# Stereoselective palladium-catalyzed carboetherification of cyclopropenes *via* a tethering strategy

**DOI:** 10.1039/d5sc09351a

**Published:** 2026-01-07

**Authors:** Duncan K. Brownsey, Alexandre A. Schoepfer, Jerome Waser

**Affiliations:** a Laboratory of Catalysis and Organic Synthesis, Institute of Chemical Sciences and Engineering, École Polytechnique Fédérale de Lausanne CH-1015 Lausanne Switzerland jerome.waser@epfl.ch https://lcso.epfl.ch/; b National Centre for Competence in Research-Catalysis (NCCR-Catalysis) Switzerland; c Laboratory for Computational Molecular Design, Institute of Chemical Sciences and Engineering, École Polytechnique Fédérale de Lausanne CH-1015 Lausanne Switzerland

## Abstract

Highly functionalized cyclopropanes are often sought after chemical motifs as building blocks in synthetic and medicinal chemistry. However, their stereoselective synthesis using catalytic methods remains a challenge. Herein we report the first carboetherification of cyclopropenes using a palladium-catalyzed tethering strategy. This reaction was compatible with various functional groups, and could be performed using aryl, alkynyl and vinyl coupling partners. The carboetherification proceeded in a stereoselective manner imparted by the trifluoromethylated tether and afforded pentasubstituted spirocyclopropanes as single diastereoisomers, extending significantly the scope of metal-catalyzed difunctionalization of strained alkenes. This process could be easily scaled up to a gram scale, and product modifications were enabled either by acid mediated ring-opening or by accessing free alcohols and amines.

## Abstract 2

We report a Pd-catalyzed carboetherification of cyclopropenes using a tethering strategy. This reaction produces single diastereomers of highly functionalized spirocyclic cyclopropanes in one step from cyclopropenes.

## Graphical abstract

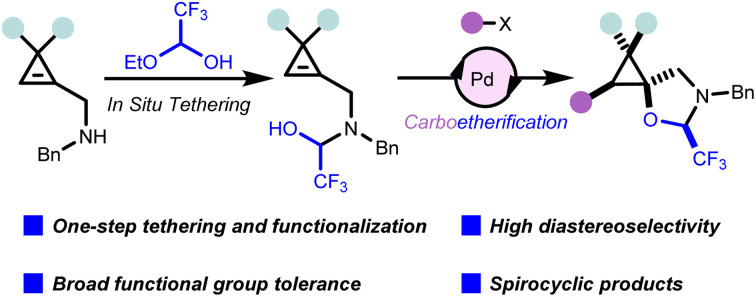

## Introduction

**Scheme 1:**
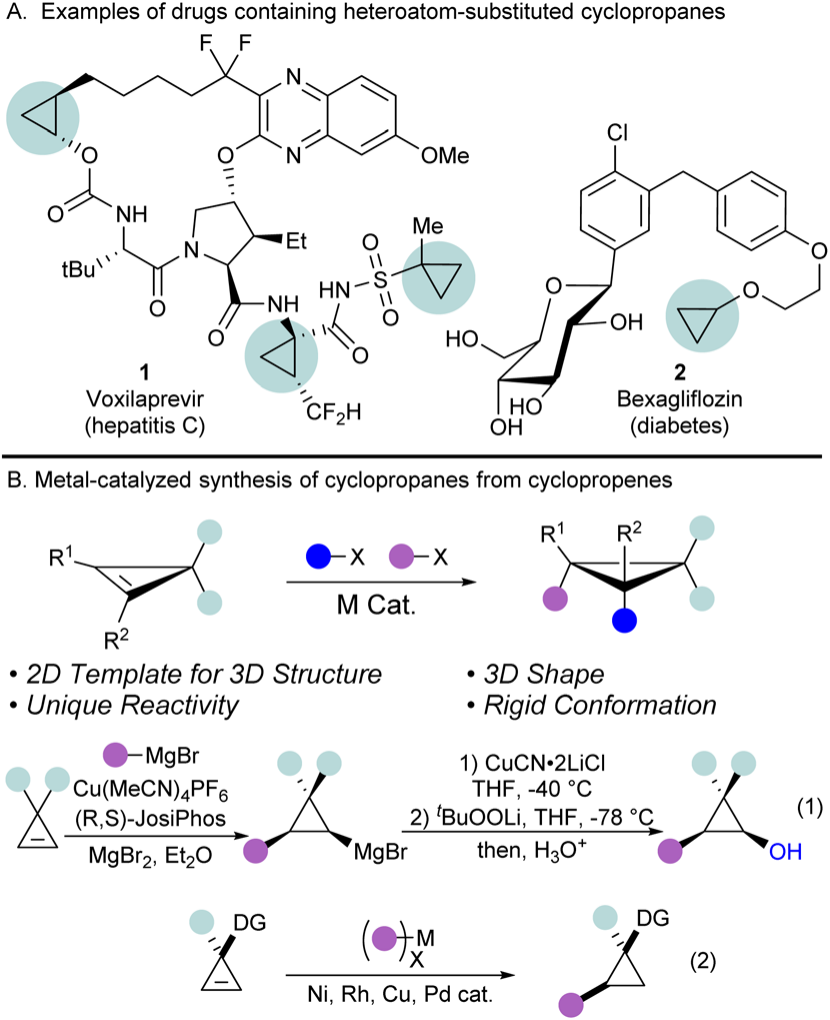
Cyclopropenes are versatile templates for the synthesis of polysubstituted cyclopropanes. (A) Heteroatom-substituted cyclopropane containing drugs. (B) Metal-catalyzed synthesis of substituted cyclopropanes from cyclopropenes.

Cyclopropanes are the smallest possible cycloalkanes and have attracted the attention of medicinal and organic chemists due to their unique properties.^1^ They are found in many natural products and drugs, and as of 2020, cyclopropanes are the 6th most frequently used ring in FDA approved drugs.^2,3^ The small size, rigid shape, and increased metabolic stability make cyclopropanes sought after moieties in drug design.^4,5^ In particular, cyclopropanes with heteroatomic substituents are of interest to chemists due to their unique reactivity and tuned properties.^6^ For example, oxygen substituted cyclopropanes, such as alkoxycyclopropanes, have been utilized in drug development (Scheme 1A, compounds 1 and 2),^7,8^ while cyclopropanols are well studied 3-carbon synthons, particularly known for their reactivity as homoenolates.^9–13^

Despite the numerous applications of oxygenated cyclopropanes, accessing them remains difficult.^14^ Traditional approaches, such as the Kulinkovich reaction, give access to a variety of cyclopropanols, but require the use of organometallic reagents, limiting functional group tolerance, and are limited to specific substitution patterns on the cyclopropane ring.^15,16^ Another strategy to prepare highly substituted cyclopropanes consists of the stereoselective functionalization of the double bond of cyclopropenes.^17^ Concerning oxygen-substituted cyclopropanes, directing group strategies have been used to guide the addition of strongly basic oxygen nucleophiles across the cyclopropene double bond.^18–21^ Another method to achieve the carbooxygenation of cyclopropenes is the use of hetero-Diels-Alder reactions between cyclopropenes and enones for the synthesis of fused bicyclic systems.^22,23^

To achieve more general double bond difunctionalizations of cyclopropenes under milder conditions, transition metal catalysis has high potential. However, controlling simultaneously regio- and stereoselectivity in difunctionalization reactions is highly challenging. Furthermore, the highly strained nature of cyclopropenes makes them prone to polymerization and ring-opening processes in the presence of transition metal catalysts, thus, methods developed for standard alkenes are often not directly applicable in this case. Seminal reports from the Marek group have demonstrated the utility of the carbometallation of cyclopropenes using Grignard or organozinc reagents with copper catalysts (Scheme 1B, eqn (1)).^21,24–28^ The formed organometallic intermediates are subsequently reacted with electrophiles, such as lithium *tert*-butyl peroxide, to access functionalized cyclopropanols. However, this strategy is limited by the compatibility of functional groups with organometallic reagents, which are still formed in stoichiometric amounts. In contrast, many reports on the hydrofunctionalization of cyclopropenes have been documented using transition metal catalysis not involving highly reactive intermediates,^29–36^ but these processes remain limited to introduction of a single functionality (Scheme 1B, eqn (2)). Therefore, there is an urgent need for further mild transition-metal catalyzed difunctionalizations of cyclopropenes not involving highly reactive intermediates.

**Scheme 2:**
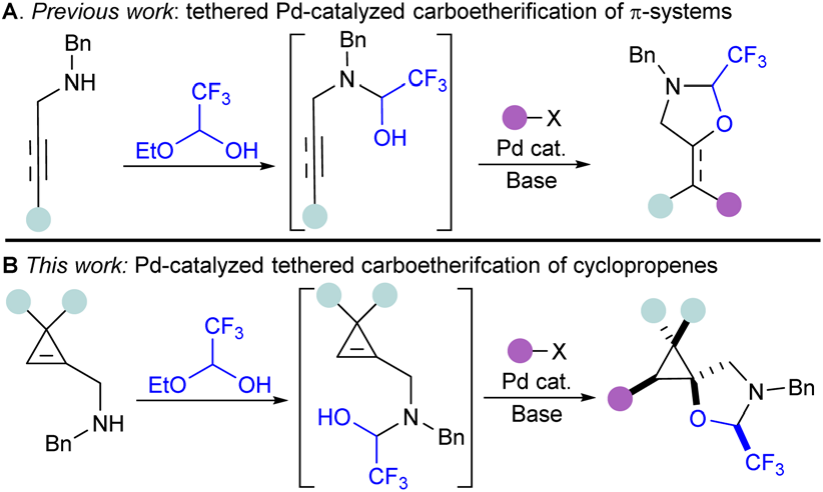
Pd-catalyzed tethering strategy for the difunctionalization of π-systems: (A) tethering strategy for the Pd-catalyzed carboetherification of alkenes and alkynes. (B) This work.

To rapidly prepare diverse oxygenated cyclopropanes without the use of organozinc or Grignard reagents, we sought to utilize the palladium-catalyzed tethering strategy that our lab has previously developed for the difunctionalization of alkenes and alkynes (Scheme 2A).^37^ This catalytic tethering strategy relies on the condensation of hemiacetal or hemiaminal tethers onto nucleophilic handles next to the π-system, followed by nucleopalladation and reductive elimination.^38,39^ Thus far, this strategy has been limited to terminal alkenes when Pd^0/II^ catalytic systems were used, and functionalization of non-terminal alkenes required the use of Pd^II/IV^ catalysis.^40^ As cyclopropenes display high reactivity, and a partial sp character, we hypothesized that a Pd^0/II^ catalytic system could still be used for their functionalization, analogously to the functionalization of internal alkynes.^41–44^

Herein, we report the first example of a catalytic carboetherification of cyclopropenes, achieved *via* a Pd-catalyzed tethering strategy. This method allows the stereoselective preparation of pentasubstituted cyclopropanes bearing a spirocyclic oxazolidine (Scheme 2B). It constitutes the first example of successful C–C bond formation at a secondary position using the Pd-catalyzed tethering strategy. Reaction optimization was guided by multivariate linear regression models for the screened monophosphine ligands. A range of carbon coupling partners could be used, including arenes, alkenes and alkynes. Notably, this carboetherification is stereoselective, and produces polysubstituted spirocyclopropanes as single diastereoisomers. The prepared pentasubstituted cyclopropanes could undergo a variety of modifications, including ring opening, reduction, and benzyl group removal.

## Results and discussion

**Fig. 1:**
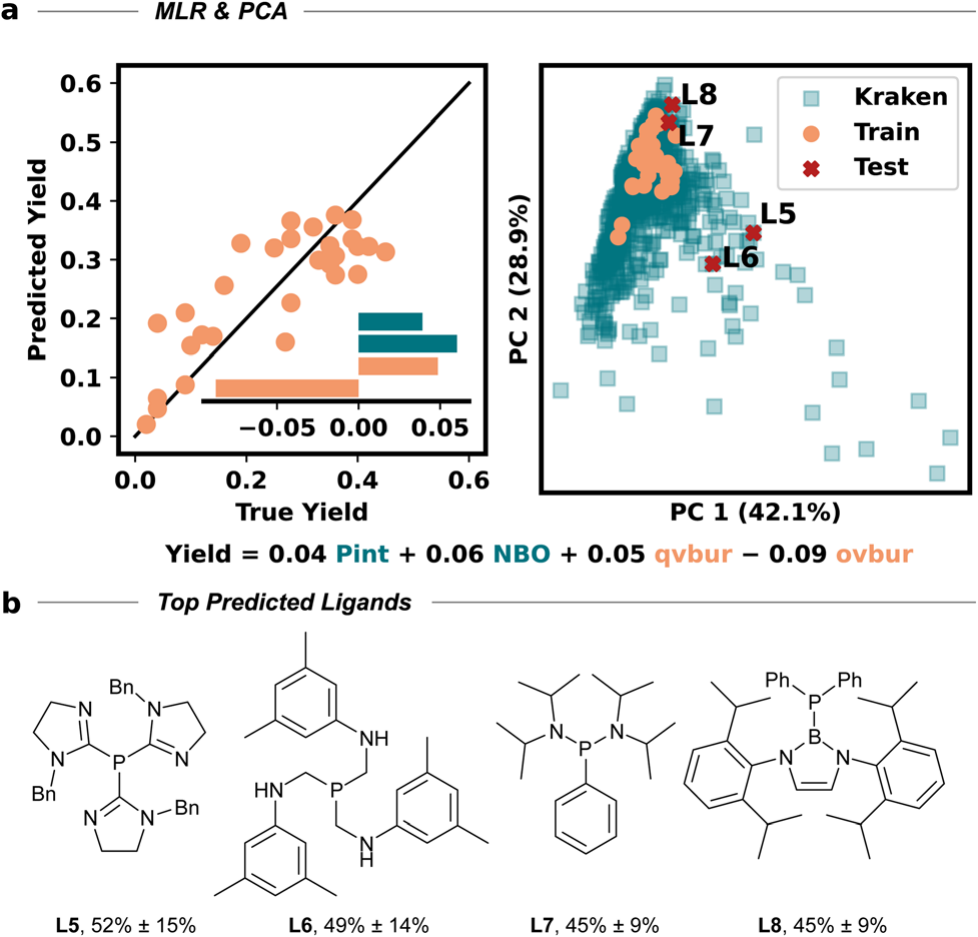
MLR and PCA guided optimization. (A, left) MLR model for ligand screening, constructed using Bayesian ridge regression and automated feature selection. The model equation is shown above, feature parameter abbreviations are as follows (see SI): molecular dispersion descriptor *P*_int_ = *P*_int_, lowest P–X antibonding orbital energy = NBO, lowest quadrant buried volume = qvbur, lowest octant buried volume = ovbur. (A, right) PCA of the selected feature subset, visualizing ligand diversity and model coverage. Teal squares indicate Kraken dataset points, orange dots represent previously tested ligands, and red crosses mark prospective high-yielding ligand predictions not yet evaluated experimentally. (B) Structures of the top four predicted ligands, as indicated in the PCA.

The amino cyclopropene 3a required for investigating our strategy was easily accessed *via* a rhodium catalyzed [2 + 1] cycloaddition of a diazoester and protected propargyl amine, followed by Boc deprotection. Carboetherification conditions were then screened using aryl iodide 4a and hemiacetal tether 5 as reaction partners (Table 1). Initial screening revealed an efficient catalytic system (see SI) when Pd_2_dba_3_·CHCl_3_ was used with L1 (SPhos) and Cs_2_CO_3_, in toluene at 80 °C, producing the desired carboetherification product 6a in 53% yield as a single diastereomer (entry 1). Other phosphine ligands were also examined, such as BrettPhos (entry 2), and ligands that have been previously used for tethered carboetherification reactions,^39,41^ including tri(2-furyl)phosphine (entry 3) and XantPhos (entry 4), which afforded the desired spirocyclic cyclopropane product, albeit less effectively than SPhos. Various other monophosphine ligands were screened, and multivariate linear regression (MLR) models were built by featurized monophosphine ligands from the Kraken data set(Fig. 1a, left), followed by principal component analysis (PCA) using either the full available features, or a subset of features selected by the MLR models (Fig. 1a, right and SI).^45,46^ The model was unable to confidently predict new ligands with greatly improved yield (*e.g.*L5–L8) compared to our best performing ligand, L1 (Fig. 1b). This important finding indicated that further ligand screening was not needed, and it was decided that other reaction parameters should be optimized to further improve the yield.

**Table 1:** Optimization of tethered carboetherification of cyclopropene 3a^a^

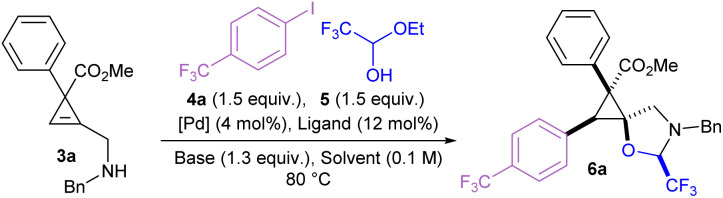				
Entry	[Pd]/ligand	Solvent	Base	Yield of 6a^b^
1	Pd_2_dba_3_·CHCl_3_/L1	Toluene	Cs_2_CO_3_	53%
2	Pd_2_dba_3_·CHCl_3_/L2	Toluene	Cs_2_CO_3_	13%
3	Pd_2_dba_3_·CHCl_3_/L3	Toluene	Cs_2_CO_3_	32%
4	Pd_2_dba_3_·CHCl_3_/L4	Toluene	Cs_2_CO_3_	19%
5	SPhos Pd G3/L1	Toluene	Cs_2_CO_3_	69%
6	SPhos Pd G3/L1	DCE	K_3_PO_4_	70%
7^c^	SPhos Pd G3/L1	DCE	K_3_PO_4_	80%
8^d^	SPhos Pd G3/L1	DCE	K_3_PO_4_	67%
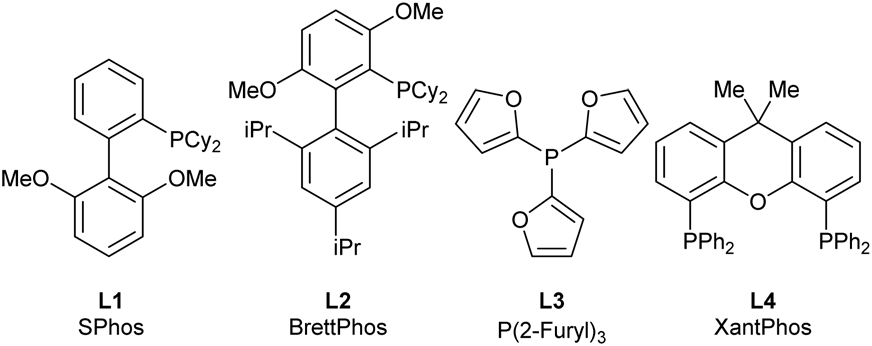				

a Reactions were performed on a 0.07 mmol scale at 0.1 M concentration for 16 h (see SI for the reaction procedure).

b Yield of 6a determined by ^1^H NMR using trichloroethylene as an internal standard.

c Reaction run at 50 °C.

d Reaction run at 23 °C.

Switching the palladium source to the Buchwald precatalyst SPhos Pd G3 improved the yield to 69% (entry 5).^47^ Using DCE as solvent and tripotassium phosphate as the base resulted in a similar yield of 70% (entry 6). The yield was improved to 80% upon reduction of the reaction temperature to 50 °C (entry 7). However, further cooling to room temperature resulted in a minor loss of yield to 67% (entry 8). The reaction was compatible with more electron rich aryl iodides to afford phenyl, *para*-methyl, and *para*-methoxy products (6b, 6c, and 6d) in moderate yields, and *para-tert*-butyl product 6e in 20% yield. Yields of the electron-rich aryl iodides were likely reduced due to slower rates of oxidative addition.^48^ Fluoro and chloro groups were well tolerated to afford 6f and 6g in 71% and 72% yield, respectively. However, brominated aryl iodides were unsuccessful as they suffered from low yields and poor selectivity. Several other *para*-substituted products with electron withdrawing groups such as trifluoromethoxy (6h), cyano (6i), and nitro groups (6j), were prepared in yields ranging from 57 to 70%, and compound 6j provided crystals which were suitable for X-ray analysis, allowing assignment of the relative configuration of these compounds.^49^ Carbonyl containing products such as an ester (6k), an aldehyde (6l) and a ketone (6m) were well tolerated. Methoxy substituted product 6n was produced in 56%, demonstrating tolerance to substitution at the *meta* position. The reaction was more sensitive to substitution at the *ortho* position, as product 6o bearing an *ortho*-methyl group was produced only in 26% yield, while a smaller and more electron deficient *ortho*-fluoro group gave 6p in 61%. Bulky *ortho*-trifluoromethyl substituted 6q was obtained in 27% yield. Trisubstituted aryl iodides were used to prepare 6r, 6s and 6t in moderate yields. Heterocyclic aryl iodides were also tolerated, affording quinoline 6u, pyridine 6v, 2-fluoropyridine 6w, and thiophene 6x in 31–59% yield.

**Scheme 3:**
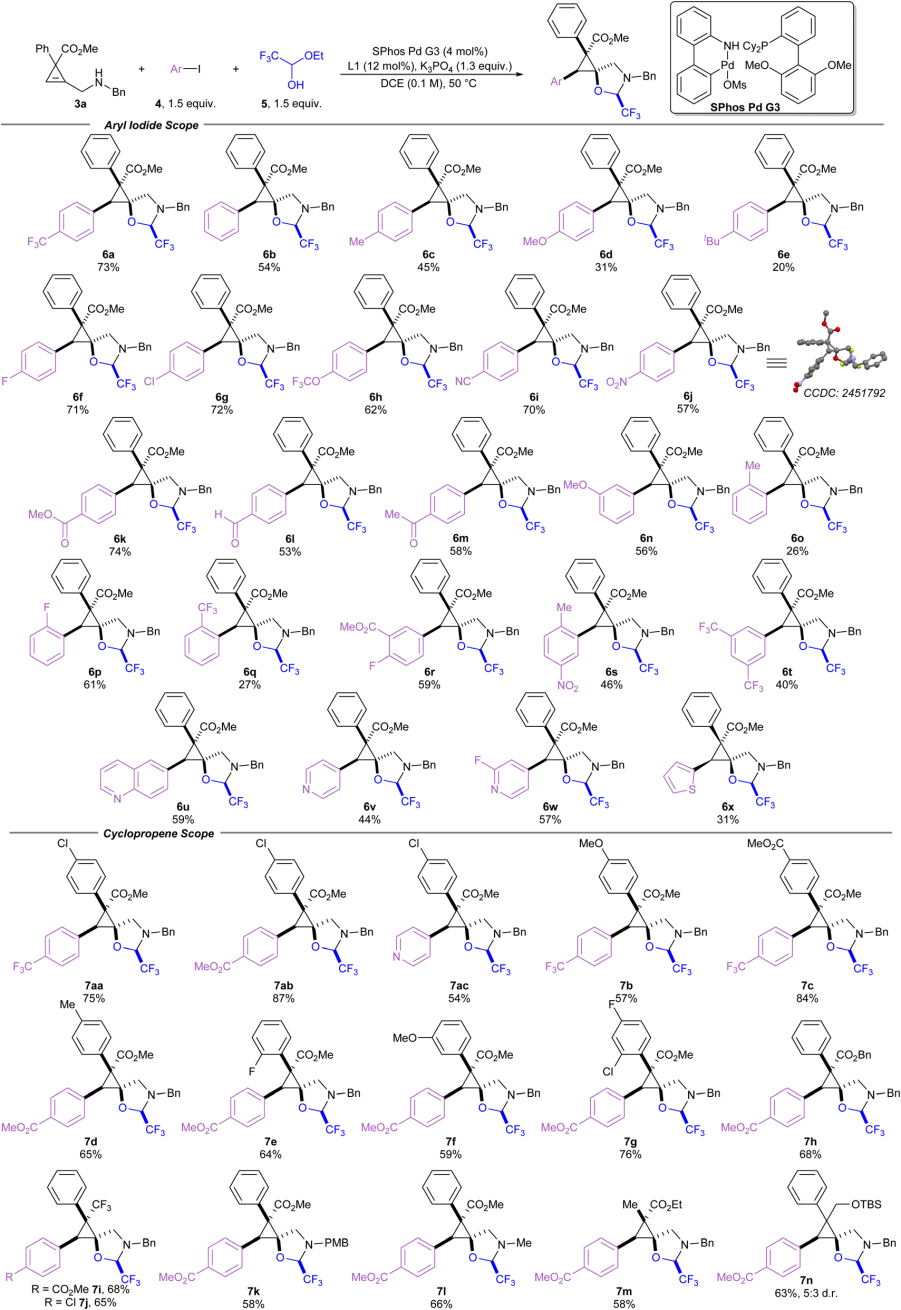
Cyclopropene carboetherification scope. Reactions were performed at a 0.2 mmol scale. Products were isolated as single (>20 : 1) diastereoisomers unless otherwise stated.

Substituent effects on the cyclopropene starting materials were then examined (Scheme 3), beginning with variations on the aryl group at the 3-position of the cyclopropene ring. Yields were improved when a chloro group was placed at the *para*-position of the arene ring (7aa–7ac) compared to the unsubstituted arene ring. Conversely, when an electron donating *para*-methoxy group is placed on the arene ring, the yield for 7b was reduced to 57% compared to 73% yield for the unsubstituted phenyl ring (6a). Generally, other substituents were well tolerated on the arene ring, including an ester (7c), a methyl group (7d), a *ortho*-fluoro group (7e), a *meta*-methoxy group (7f) and multiple halogens (7g). The methyl ester at the 3-position of the cyclopropene ring could be replaced with a benzyl ester (7h) or a trifluoromethyl group (7i and 7j). The benzyl amine could also be replaced by a *para*-methoxybenzyl amine or a methyl amine giving products 7k and 7l in 58 or 66% yield, respectively. The arene at the 3-position of the cyclopropene could be replaced by a methyl group to afford 7m in 58% yield. The reaction proceeded without an electron withdrawing group at the 3-position of the cyclopropene, affording product 7n in 63% yield, however, with an erosion of the d.r. to 5 : 3.

**Scheme 4:**
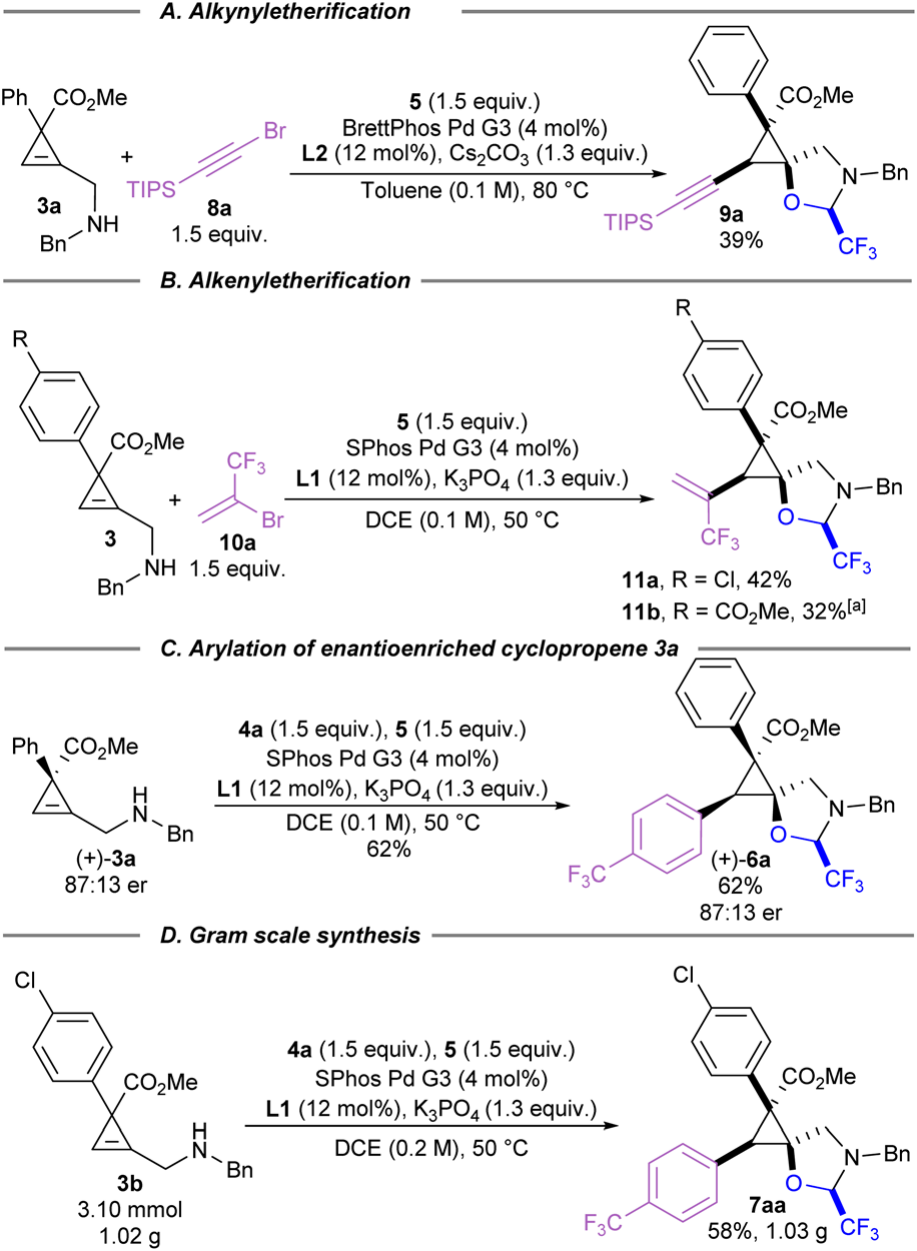
Investigation of the generality of tethered cyclopropene carboetherification. (A) Preparation of alkynyl cyclopropane 9a through reaction with alkynyl halide 8a. (B) Preparation of vinylcyclopropanes 11a and 11b through reaction with vinyl halide 10a. (C) Stereospecific reaction with enantioenriched (+)-3a. (D). Gram-scale preparation of 7aa from the carboetherification of cyclopropene 3b with aryl halide 4a. [a] Reaction performed on 0.9 mmol scale.

Carbon coupling partners beyond aryl iodides were briefly examined for their efficacy in this carboetherification reaction (Scheme 4). Alkynylation of cyclopropene 3a could be achieved using alkynyl bromide 8a (Scheme 4A). The standard reaction conditions developed for arylation did not lead to significant amounts of the desired product. A short screening of ligands and Pd precursors showed that product 9a could be isolated in 39% yield when BrettPhos Pd G3 was used as a Pd source. When vinyl bromide 10a was used as a coupling partner, the developed conditions for alkynylation did not produce any detectable amounts of desired product 11a (Scheme 4B). However, standard arylation conditions using SPhos Pd G3 afforded vinylcyclopropanes 11a and 11b in 42% and 30% yield, respectively.^50^

The stereospecificity of this tethered carboetherification was explored by preparing cyclopropene (+)-3a using a chiral rhodium catalyst (Scheme 4C).^51^ In this case, even without further optimization of the catalytic system, cyclopropane 3a could already be obtained in 87 : 13 er. After subjecting the enantioenriched starting material to the standard arylation conditions, product 6a was isolated with the same enantiomeric ratio, demonstrating the potential to prepare single enantiomers of these polysubstituted spirocyclopropanes. Next, the arylation of 3b was scaled up to gram-scale (3.1 mmol), which delivered over 1 gram of trifluoromethylated product 7aa in 58% yield (Scheme 4D).

**Scheme 5:**
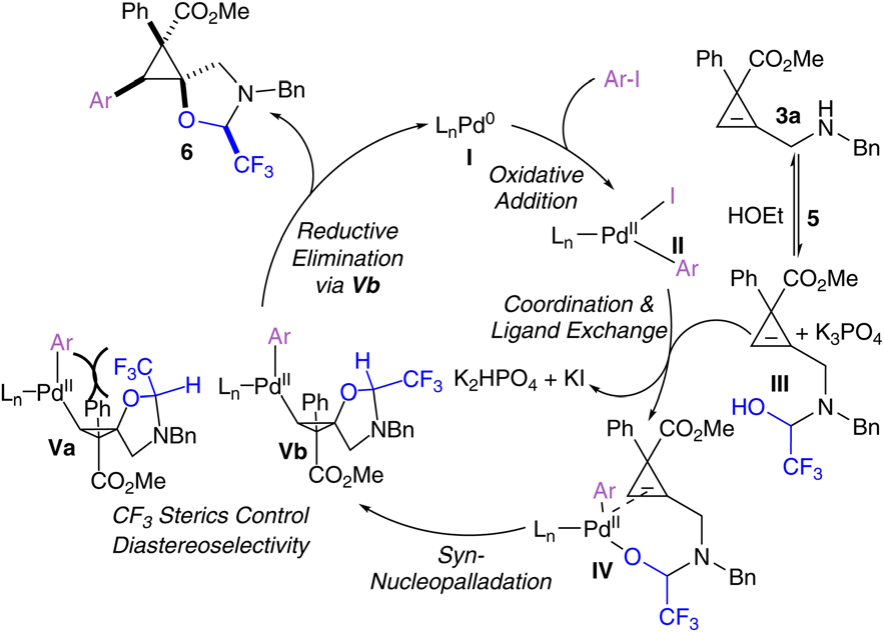
Plausible mechanism for the tethered cyclopropene carboetherification.

A plausible mechanism for the tethered carboetherification of cyclopropenes begins with the oxidative addition of Pd^0^ complex I to the aryl iodide, to give Pd^II^ intermediate II (Scheme 5). Concurrently, reversible tether condensation of the benzyl amine group onto hemiacetal tether 5 gives hemi-acetal III. Next, we hypothesize that the tethered cyclopropane III can coordinate with the activated Pd^II^ catalyst II to give complex IV through ligand exchange.^44^ Then IV undergoes nucleopalladation across the cyclopropene double bond with *syn*-selectivity, which is frequently observed in intramolecular nucleopalladation reactions.^52^ To obtain the observed product, this nucleopalladation must also occur with *anti*-facial selectivity with respect to the electron withdrawing group at the 3-position of the cyclopropene. Where no electron withdrawing group is present, such as for compound 7n, a loss of diastereoselectivity was observed, and when a CF_3_ is placed at the 3-position (7i and 7j), the same facial selectivity is observed. An *anti* attack with respect to the electron-withdrawing group has also been reported for the copper-catalyzed functionalization of 1,1-aryl-ester-substituted cyclopropenes.^53,54^ The resulting complex V then undergoes reductive elimination to afford the spirocyclic product 6 and regenerates the Pd^0^ catalyst I. We hypothesize that the stereocenter bearing the trifluoromethyl group on the tethered oxazolidine is controlled during the nucleopalladation step. The trifluoromethyl group on the oxazolidine ring can be placed either on the same (Va) or opposite (Vb) face as the palladium. The steric clash between these groups on the same face prevents efficient production of Va, leaving Vb as the dominant intermediate. However, it cannot be currently ruled out that the observed diastereoselectivity arises through different reductive elimination rates for intermediates Va and Vb (see SI).

**Scheme 6:**
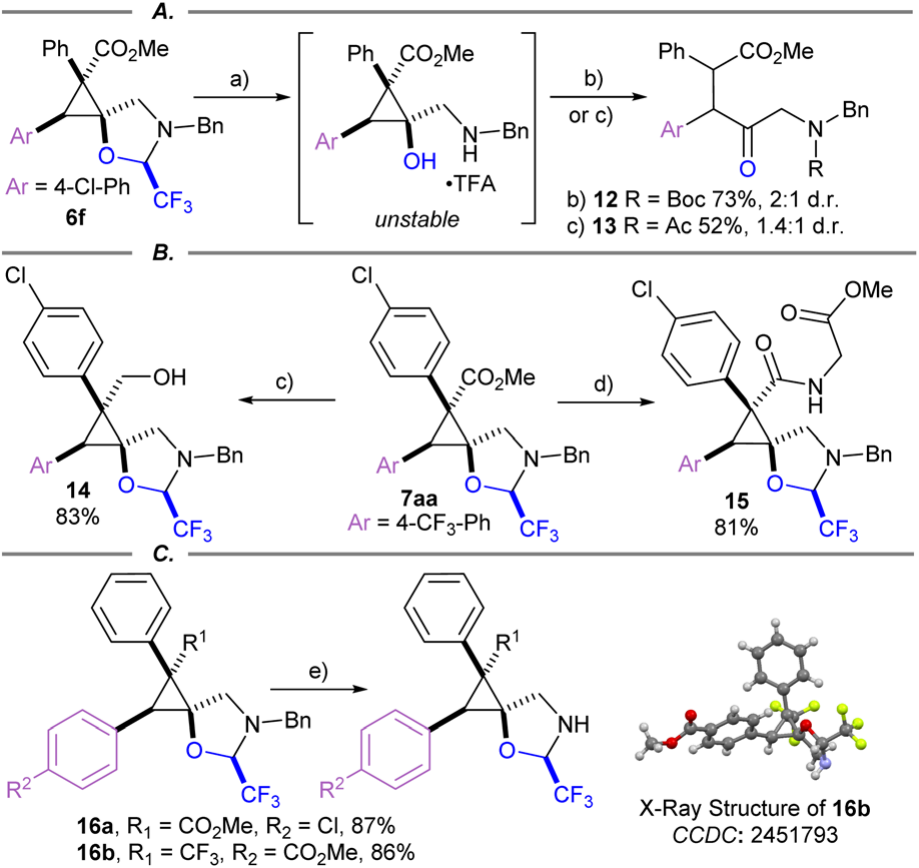
Modification of spirocyclic cyclopropanes. (A) Ring opening of cyclopropane 6f*via* acidic hydrolysis. (B) Modifications of 7aa. (C) Benzyl group deprotection. Conditions: (a) TFA (4.0 equiv.), HFIP, rt, 16 h; (b) Boc_2_O (3.0 equiv.), Et_3_N (5.0 equiv.), DCM, rt, 2 h; (c) AcCl (2.0 equiv.), Et_3_N (5.0 equiv.), DCM, rt, 2 h; (d) LiAlH_4_ (2.0 equiv.), THF, −78 °C, 2 h; (e) (i) LiOH (4.2 equiv.), THF/H_2_O (9 : 1), rt, 16 h ; (ii) glycine methyl ester HCl (1.3 equiv.), HATU (1.3 equiv.), DIPEA (3.0 equiv.), DMF, rt, 16 h; (f) H_2_ (1 atm), Pd/C (20 mol%), EtOAc, rt, 16 h.

Several product modifications were then performed to highlight the potential of the obtained spirocyclopropanes as building blocks (Scheme 6). The oxazolidine ring of 6f was cleaved successfully with several equivalents of TFA in HFIP. However, the obtained cyclopropanol intermediate was unstable, resulting in the formation of a ring-opened amino ketone. The latter could be isolated after capture with an electrophile such as Boc anhydride or acetyl chloride to afford 1,2-diarylated ketones 12 and 13 (Scheme 6A). Reduction of ester 7aa was achieved using LiAlH_4_ to give primary alcohol 14, as a single diastereomer in contrast to the direct reaction of protected cyclopropyl alcohols that gave 7n as a mixture of diastereoisomers (Scheme 6B). Unfortunately, attempts to cleave the oxazolidine on 14 resulted either in starting material recovery or full decomposition. Methyl ester 7aa was saponified, then coupled with glycine methyl ester to give amide 15. Benzyl group deprotection was readily achieved under hydrogenative conditions using Pd/C as a catalyst to give free amine 16a and 16b in good yield (Scheme 6C). The structure of 16b was then elucidated *via* X-ray crystallography, demonstrating the same relative configuration of the electron withdrawing group at the 3-position of the cyclopropane as ester containing products.^55^ Therefore, the obtained spirocycles constitute highly rigid g-amino acid derivatives, which could be included into peptide therapeutics.^56,57^

## Conclusions

In conclusion, we have developed the first palladium-catalyzed carboetherification of cyclopropenes, which was achieved using a tethering strategy. The reaction optimization was guided by multivariate linear regression models for the screened monophosphine ligands. The resulting pentasubstituted spirocyclic cyclopropanes were prepared in a highly diastereoselective manner, with good functional group tolerance, and with aryl, alkynyl and vinyl coupling partners. A variety of modifications were demonstrated for these products, allowing either to conserve the spirocyclic scaffold or open it. In particular, the spirocyclic core demonstrated high stability, allowing precise arrangement of substituents in space, including easily modifiable ester and amine groups. Our work therefore further extends the scope of metal-catalyzed cyclopropene difunctionalization for efficient access to multi-functionalized polycyclic building blocks.

## Author contributions

DKB designed the project and performed the experiments. AAS developed the MLR models used for reaction optimization and performed the DFT calculations. JW supervised the project. DKB and JW wrote the manuscript with contributions from all authors. All authors have given approval to the final version of the manuscript.

## Conflicts of interest

There are no conflicts to declare.

## Supplementary Material

SC-017-D5SC09351A-s001

SC-017-D5SC09351A-s002

## Data Availability

CCDC 2451792 (6j) and 2451793 (16b) contain the supplementary crystallographic data for this paper.^49,55^ Supplementary information (SI): experimental procedures, supplementary figures, characterization data, computational details, and copies of NMR spectra for new compounds are available as a pdf file. See DOI: https://doi.org/10.1039/d5sc09351a. Raw data for NMR, MS and IR is freely available on the platform zenodo (https://doi.org/10.5281/zenodo.17610761).
