# Supplementary material for: Stereoselective palladium-catalyzed carboetherification of cyclopropenes via a tethering strategy
Source: Chem Sci. 2026 Jan 7;17(10):5072–8. doi: 10.1039/d5sc09351a (PMC12814203; doi:10.1039/d5sc09351a)
Supplement: SC-017-D5SC09351A-s001 [file SC-017-D5SC09351A-s001.pdf]

Supplementary Information (SI) for Chemical Science.
This journal is © The Royal Society of Chemistry 2026

Supporting Information for

"Stereoselective Palladium-Catalyzed Carboetherification of Cyclopropenes via a Tethering Strategy"

Duncan K. Brownsey, Alexandre A. Schoepfer and Jerome Waser*

Laboratory of Catalysis and Organic Synthesis, École Polytechnique Fédérale de Lausanne, EPFL, SB ISIC LCSO, BCH 4306, 1015 Lausanne (Switzerland)

*Correspondence to: jerome.waser@epfl.ch

Table of Contents

General Information 3
Synthesis of Starting Materials 3
Synthesis of Diazo Compounds 4
Synthesis of alkynes 8
Synthesis of amino cyclopropenes 10
Optimization Studies 19
Procedures and Product Characterization of Cyclopropane Products 25
Product Modifications 53
DFT Calculations 58
X-ray Crystallographic Data 60
References 81
Copies of NMR Spectra 82
Cyclopropenes 82
Cyclopropane products 100
Product Modifications 164
HPLC Traces 173

General Information

The NMR spectra were recorded on a Brucker DPX-400 spectrometer at 400 MHz for 1H, 101 MHz for 13C, 376 MHz for ^{19}F. The chemical shift (δ) for ^{1}H and ^{13}C are given in ppm relative to residual signals of the solvents (CDCl3 - 7.26 ppm 1H NMR and 77.16 ppm 13C NMR). Carbon spectra have been measured using broadband {^{1}H} decoupling. Coupling constants are given in Hertz. The following abbreviations are used to indicate the multiplicity: s, singlet; d, doublet; q, quartet; m, multiplet; bs, broad signal; app, apparent. Infrared spectra were recorded on a JASCO FT-IR B4100 spectrophotometer with an ATR PRO410-S and a ZnSe prisma and are reported as cm^{-1} (w = weak, m = medium, s = strong, br = broad). High resolution mass spectrometric measurements were performed by the mass spectrometry service of ISIC at the EPFL on a MICROMASS (ESI) Q-TOF Ultima API. The raw data obtained from the Q-TOF Waters instrument does not take into account the mass of the electron for the ion, the obtained raw data has been therefore corrected by removing the mass of the electron (5 mDa). The diffraction data for crystal structures were collected by mass spectrometry service of ISIC at the EPFL at low temperature using Cu (323) or Mo (520) K_a radiation on a Rigaku SuperNova dual system in combination with Atlas type CCD detector. The data reduction and correction were carried out by CrysAlisPro (Rigaku Oxford Diffraction, release 1.171.40.68a, 2019). The solutions and refinements were performed by *SHELXT*[1] and *SHELXL*,[2] respectively. The crystal structures were refined using fullmatrix least-squares based on F^2 with all non-H atoms defined in anisotropic manner. Hydrogen atoms were placed in calculated positions by means of the “riding” model. Yields of isolated products refer to materials of >95% purity as determined by ^{1}H NMR.

The authors are indebted to the team of the research support service of ISIC at EPFL, particularly to the NMR, XRay, and the High Resolution Mass Spectrometry Units.

General Procedures. All reactions were set up under a nitrogen atmosphere in oven-dried glassware using standard Schlenk techniques, unless otherwise stated. Synthesis grade solvents were used as purchased; anhydrous solvents (THF, Et_2O, Toluene and DCM) were taken from a commercial SPS solvent dispenser (H_2O content < 10 ppm, *Karl-Fischer* titration). Chromatographic purification of products was accomplished using flash chromatography (FC) on SiliaFlash P60 silica gel (230 - 400 mesh) or using Biotage Isolera Spektra One with pre-packaged silica cartridges purchased from Buchi, models: Sepacore or GraceResolve (4 g, 12 g, 25 g, 40 g, 80 g, 120 g). For thin layer chromatography (TLC) analysis throughout this work, Merck silica gel 60 F254 TLC glass plates were employed, using UV light as the visualizing agent and basic aqueous potassium permanganate ($KMnO_4$) stain solutions, and heat as developing agents. Organic solutions were concentrated under reduced pressure on a Büchi rotatory evaporator. **Determination of Enantiomeric Ratios**. HPLC analysis on chiral stationary phase was performed on an Agilent Acquity instrument using a Daicel CHIRALPAK IB-N5 chiral column. The exact conditions for the analyses are specified within the characterization section. HPLC traces were compared to racemic samples prepared by running the reactions using racemic ligands. Optical rotations were measured on a polarimeter using a 10 cm cell with a Na 589 nm filter. The specific solvents and concentrations (in g/100 mL) are indicated.

Materials. Most of the starting materials used in this study are commercial and were purchased in the highest purity available from *Sigma-Aldrich*, *Fluka*, *Alfa Aesar*, *Fluorochem*, *Enamine* and used as received, without further purifications. Tris(dibenzylideneacetone)dipalladium was purchased from *Fluorochem* and recrystallized in 200 mg portions following a reported procedure.[3]

Synthesis of Starting Materials

Synthesis of Diazo Compounds

General procedure A: Synthesis of α-aryldiazo compounds.

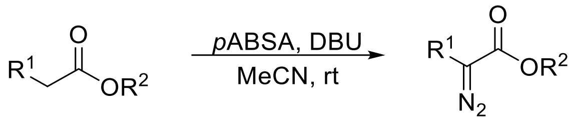

Following a slightly modified procedure,[4] DBU (1.6 equiv.) and the indicated α-aryl-acetate or were added to a solution of *p*-acetamidobenzenesulfonyl azide (1.5 equiv.) in CH_3CN (0.5 M) at room temperature and the resulting mixture was stirred for 18 h. The reaction mixture was then diluted with distilled water and extracted with diethyl ether. The combined organic layers were washed with an aqueous solution of 10% $NaHCO_3$, brine, then dried over anhydrous Na_2SO_4, filtered, and concentrated *in vacuo*. The crude residue was purified by column chromatography with the indicated solvents.

Methyl 2-diazo-2-phenylacetate (S1a)

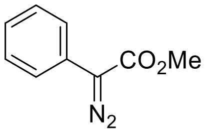

Following general procedure A, starting from methyl phenylacetate (6.50 mL, 46.3 mmol, 1.00 equiv.), the crude residue was purified by flash column chromatography (SiO_2, 40 g, 3 – 5% Et_2O in pentane) to afford **S1a** as a red oil (7.00 g, 39.8 mmol, 86%).

TLC: R_f = 0.88 (SiO_2, 5% EtOAc in pentane).

^{1}H NMR (400 MHz, $CDCl_3$) δ 7.53 – 7.46 (m, 2H, Ar*H*), 7.42 – 7.36 (m, 2H, Ar*H*), 7.23 – 7.14 (m, 1H, Ar*H*), 3.87 (s, 3H, CO_2CH_3).

^{13}C NMR (101 MHz, $CDCl_3$) δ 165.7, 129.1, 126.0, 125.6, 124.1, 52.1. One carbon is not resolved.

The characterization data correspond to previously reported values.[4]

Methyl 2-(4-chlorophenyl)-2-diazoacetate (S1b)

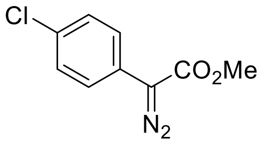

Following general procedure A, starting from methyl 2-(4-chlorophenyl)acetate (2.50 g, 13.5 mmol, 1.00 equiv.), the crude residue was purified by flash column chromatography (SiO_2, 40 g, 3 – 5% Et_2O in pentane) to afford **S1b** as an orange solid (2.65, 12.6 mmol, 93%).

TLC: R_f = 0.58 (SiO_2, 5% EtOAc in pentane).

^{1}H NMR (400 MHz, $CDCl_3$) δ 7.43 – 7.38 (m, 2H, Ar*H*), 7.37 – 7.31 (m, 2H, Ar*H*), 3.86 (s, 3H, CO_2CH_3).

^{13}C NMR (101 MHz, $CDCl_3$) δ 165.3, 131.6, 129.2, 125.1, 124.2, 52.2. One carbon is not resolved.

The characterization data correspond to previously reported values.[5]

Methyl 2-diazo-2-(4-methoxyphenyl)acetate (S1c)

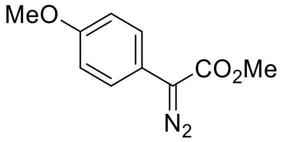

Following general procedure A, starting from methyl 4-methoxyphenylacetate (5.00 mL, 31.5 mmol, 1.00 equiv.), the crude residue was purified by flash column chromatography (SiO_2, 40 g, 3 – 5% Et_2O in pentane) to afford **S1c** as a red solid (4.30 g, 20.9 mmol, 66%).

TLC: R_f = 0.38 (SiO_2, 10% Et_2O in pentane).

^1H NMR (400 MHz, $CDCl_3$) δ 7.41 – 7.36 (m, 2H, Ar*H*), 6.96 – 6.92 (m, 2H, Ar*H*), 3.85 (s, 3H, CO_2CH_3), 3.81 (s, 3H, $ArOCH_3$).

^{13}C NMR (101 MHz, $CDCl_3$) δ 166.3, 158.2, 126.1, 117.0, 114.8, 55.5, 52.1. One carbon is not resolved.

The characterization data correspond to previously reported values.[4]

Methyl 4-(1-diazo-2-methoxy-2-oxoethyl)benzoate (S1d)

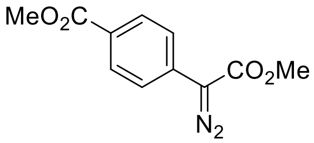

Following general procedure A, starting from methyl 4-(2-methoxy-2-oxoethyl)benzoate (3.00 g, 14.4 mmol, 1.00 equiv.), the crude residue was purified by flash column chromatography (SiO_2, 40 g, 3 – 5% Et_2O in pentane) to afford **S1d** as an orange solid (2.30 g, 11.0 mmol, 77%).

TLC: R_f = 0.29 (SiO_2, 10% Et_2O in pentane).

^1H NMR (400 MHz, $CDCl_3$) δ 8.08 – 7.99 (m, 2H, Ar*H*), 7.60 – 7.52 (m, 2H, Ar*H*), 3.91 (s, 3H, CO_2CH_3), 3.89 (s, 3H, CO_2CH_3).

^{13}C NMR (101 MHz, $CDCl_3$) δ 166.8, 165.0, 131.2, 130.3, 127.2, 123.1, 52.3, 52.2. One carbon is not resolved.

The characterization data correspond to previously reported values.[6]

Methyl 2-diazo-2-(p-tolyl)acetate (S1e)

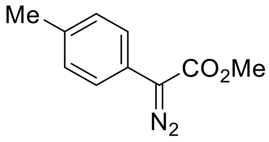

Following general procedure A, starting from methyl p-tolylacetate (2.50, 13.4 mmol, 1.00 equiv.), the crude residue was purified by flash column chromatography (SiO_2, 40 g, 3 – 5% Et_2O in pentane) to afford **S1e** as a red solid (1.66 g, 8.73 mmol, 65%).

TLC: R_f = 0.79 (SiO_2, 10% EtOAc in pentane).

^1H NMR (400 MHz, $CDCl_3$) δ 7.40 – 7.34 (m, 2H, Ar*H*), 7.23 – 7.16 (m, 2H, Ar*H*), 3.86 (s, 3H, CO_2CH_3), 2.35 (s, 3H, $ArCH_3$).

^{13}C NMR (101 MHz, $CDCl_3$) δ 166.0, 135.9, 129.8, 124.2, 122.2, 63.1, 52.0, 21.1.

The characterization data correspond to previously reported values.[7]

Methyl 2-diazo-2-(2-fluorophenyl)acetate (S1f)

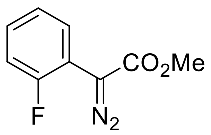

Following general procedure A, starting from methyl 2-fluorophenylacetate (2.02 g, 12.0 mmol, 1.00 equiv.), the crude residue was purified by flash column chromatography (SiO_2, 40 g, 3 – 5% Et_2O in pentane) to afford **S1f** as an orange solid (2.04 g, 10.5 mmol, 88%).

TLC: R_f = 0.77 (SiO_2, 10% Et_2O in pentane).

^1H NMR (400 MHz, $CDCl_3$) δ 7.69 (td, *J* = 7.8, 2.0 Hz, 1H, Ar*H*), 7.25 – 7.15 (m, 2H, Ar*H*), 7.08 (ddd, *J* = 11.2, 8.0, 1.7 Hz, 1H, Ar*H*), 3.86 (s, 3H, CO_2CH_3).

^{13}C NMR (101 MHz, $CDCl_3$) δ 165.8, 158.5 (d, J_{C-F} = 247.5 Hz), 129.6 (d, J_{C-F} = 2.2 Hz), 128.7 (d, J_{C-F} = 8.2 Hz), 124.8 (d, J_{C-F} = 3.5 Hz), 115.8 (d, J_{C-F} = 21.3 Hz), 113.9 (d, J_{C-F} = 11.9 Hz), 52.3. One carbon is not resolved.

^{19}F NMR (376 MHz, $CDCl_3$) δ -114.1.

The characterization data correspond to previously reported values.[4]

Methyl 2-diazo-2-(3-methoxyphenyl)acetate (S1g)

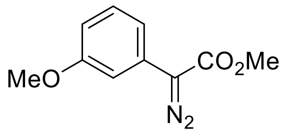

Following general procedure A, starting from methyl 3-methoxyphenylacetate (2.70 g, 15.0 mmol, 1.00 equiv.), the crude residue was purified by flash column chromatography (SiO_2, 25 g, 3 – 10% Et_2O in pentane) to afford **S1g** as a red solid (1.96 g, 9.49 mmol, 63%).

TLC: R_f = 0.54 (SiO_2, 5% Et_2O in pentane).

^1H NMR (400 MHz, $CDCl_3$) δ 7.29 (t, *J* = 8.1 Hz, 1H, Ar*H*), 7.16 (d, *J* = 4.3 Hz, 1H, Ar*H*), 6.99 (ddd, *J* = 7.9, 1.8, 0.9 Hz, 1H, Ar*H*), 6.73 (ddd, *J* = 8.3, 2.5, 0.9 Hz, 1H, Ar*H*), 3.87 (s, 3H, CO_2CH_3), 3.82 (s, 3H, ArOCH_3).

^{13}C NMR (101 MHz, $CDCl_3$) δ 165.7, 160.2, 130.0, 127.1, 116.1, 111.7, 109.8, 55.4, 52.1. One carbon is not resolved.

The characterization data correspond to previously reported values.[7]

Methyl 2-(2-chloro-4-fluorophenyl)-2-diazoacetate (S1h)

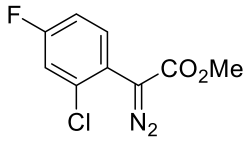

Following general procedure A, starting from methyl 2-chloro-4-fluorophenylacetate (2.43 g, 12.0 mmol, 1.00 equiv.), the crude residue was purified by flash column chromatography (SiO_2, 40 g, 3 – 5% Et_2O in pentane) to afford **S1h** as an orange solid (2.40 g, 10.5 mmol, 87%).

TLC: R_f = 0.63 (SiO_2, 10% Et_2O in pentane).

^{1}H NMR (400 MHz, $CDCl_3$) δ 7.52 (dd, *J* = 8.8, 5.9 Hz, 1H, Ar*H*), 7.19 (dd, *J* = 8.4, 2.7 Hz, 1H, Ar*H*), 7.07 (ddd, *J* = 9.0, 7.9, 2.7 Hz, 1H, Ar*H*), 3.84 (s, 3H, CO_2CH_3).

^{13}C NMR (101 MHz, $CDCl_3$) δ 166.0, 162.6 (d, J_{C-F} = 252.2 Hz), 135.1 (d, J_{C-F} = 10.4 Hz), 133.8 (d, J_{C-F} = 9.0 Hz), 120.2 (d, J_{C-F} = 3.6 Hz), 117.5 (d, J_{C-F} = 25.1 Hz), 115.0 (d, J_{C-F} = 21.7 Hz), 52.5. One carbon is not resolved.

^{19}F NMR (376 MHz, $CDCl_3$) δ -110.6.

The characterization data correspond to previously reported values.[4]

Benzyl 2-diazo-2-phenylacetate (S1i)

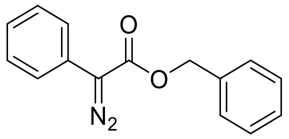

Following general procedure A, starting from benzyl 2-phenylacetate (4.20 mL, 20.4 mmol, 1.00 equiv.), the crude residue was purified by flash column chromatography (SiO_2, 80 g, 2% EtOAc in pentane) to afford **S1i** as an orange oil (4.34 g, 25.8 mmol, 85%).

TLC: R_f = 0.71 (SiO_2, 5% EtOAc in pentane).

^{1}H NMR (400 MHz, $CDCl_3$) δ 7.55 – 7.47 (m, 2H, Ar*H*), 7.45 – 7.31 (m, 7H, Ar*H*), 7.24 – 7.14 (m, 1H, Ar*H*), 5.34 (s, 2H, CH_2).

^{13}C NMR (101 MHz, $CDCl_3$) δ 165.1, 136.0, 129.1, 128.7, 128.5, 128.3, 126.0, 125.5, 124.1, 66.6. One carbon is not resolved.

The characterization data correspond to previously reported values.[8]

(1-Diazo-2,2,2-trifluoroethyl)benzene (S1j)

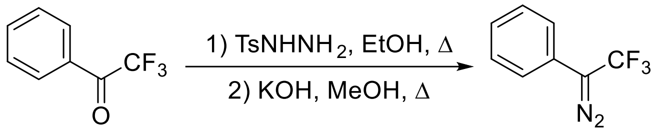

Following a reported procedure,[4] a solution of 2,2,2-trifluoro-1-phenylethanone (10.00 g, 57.43 mmol, 1.000 equiv) and 4-methylbenzenesulfonohydrazide (10.70 g, 57.43 mmol, 1.000 equiv) in ethanol (150 mL) was heated to reflux for 14 h. The mixture was cooled to rt and concentrated *in vacuo*. The resulting crude precipitate was triturated with pentane (100 mL), filtered, washed with pentane and dried under vacuum to give intermediate N-[(E)-(2,2- dimethyl-1-phenylpropylidene)amino]-4-methylbenzenesulfonamide as an off-white solid (12.05 g, 35.18 mmol, 61% yield) which was used immediately in the next step without further purification.

KOH (1.975 g, 35.20 mmol, 2.000 equiv.) was dissolved in MeOH (60 mL) by stirring at room temperature. Intermediate tosyl hydrazide (6.025 g, 17.60 mmol, 1.000 equiv.) was added in one portion and the solution was heated to reflux for 1 h, then cooled to room temperature. Water (100 mL) was added and the resulting mixture was extracted with pentane (3 x 150 mL). The combined organic layers were dried over anhydrous Na_2SO_4, filtered and concentrated *in vacuo* to give **S1j** (1.124 g, 6.039 mmol, 34% yield) as a red oil, which was used directly without further purification.

^{1}H NMR (400 MHz, $CDCl_3$) δ 7.44 – 7.38 (m, 2H, Ar*H*), 7.24 – 7.17 (m, 1H, Ar*H*), 7.14 – 7.04 (m, 2H, Ar*H*).

^{13}C NMR (101 MHz, $CDCl_3$) δ 129.6, 126.1, 125.8 (q, J_{C-F} = 269.2 Hz), 123.7, 122.4. One carbon is not resolved.

^{19}F NMR (376 MHz, $CDCl_3$) δ -57.4.

The characterization data correspond to previously reported values.[4]

Ethyl 2-diazopropanoate (S1k)

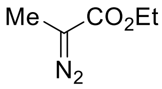

Following general procedure A, starting from ethyl 2-methylacetoacetate (5.00 mL, 35.4 mmol, 1.00 equiv.), the crude residue was purified by flash column chromatography (SiO_2, 80 g, 2% Et_2O in pentane) to afford **S1k** as a yellow oil (2.33 g, 18.2 mmol, 52%).

TLC: R_f = 0.37 (SiO_2, 10% Et_2O in pentane).

^{1}H NMR (400 MHz, $CDCl_3$) δ 4.22 (q, *J* = 7.1 Hz, 2H, $CO_2CH_2CH_3$), 1.96 (s, 3H, C*H*$_3$), 1.27 (t, *J* = 7.1 Hz, 3H, $CO_2CH_2CH_3$).

^{13}C NMR (101 MHz, $CDCl_3$) δ 168.2, 60.9, 14.7, 8.6. One carbon is not resolved.

The characterization data correspond to previously reported values.[9]

Synthesis of alkynes

tert-Butyl benzyl(prop-2-yn-1-yl)carbamate (S2)

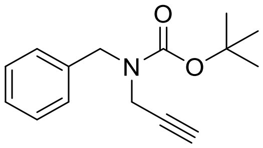

Following a modified procedure,[10] tert-butyl benzylcarbamate (7.986 g, 38.53 mmol, 1.000 equiv.) was dissolved in DMF (45 mL) and cooled to 0 °C with an ice-water bath. NaH (60% in oil dispersion, 1.695 g, 42.38 mmol, 1.100 equiv.) was then added in several portions and the solution was stirred for 30 minutes at 0 °C. A propargyl bromide solution (80% wt/v in toluene, 4.95 mL, 46.2 mmol, 1.20 equiv.) was then added dropwise, and the reaction was warmed to room temperature. After 16 h, the reaction was quenched with aqueous KOH solution (2M, 30 mL), and extracted with EtOAc (4 x 30 mL). The combined organic layers were washed with 10 wt/v LiCl solution (2 x 30 mL), dried over anhydrous Na_2SO_4, and concentrated *in vacuo*. The crude residue was then purified via flash chromatography (70 g SiO_2, 5% EtOAc in pentane) to afford **S2** as a colourless oil (8.163 g, 33.28 mmol, 86%).

TLC: R_f = 0.54 (SiO_2, 10% Et_2O in pentane).

^{1}H NMR (400 MHz, $CDCl_3$) δ 7.40 – 7.21 (m, 5H, Ar*H*), 4.55 (s, 2H, C*H*$_2$), 4.10 – 3.79 (m, 2H, C*H*$_2$), 2.21 (t, *J* = 2.5 Hz, 1H, CC-*H*), 1.49 (s, 9H, C(C*H*$_3$)$_3$).

^{13}C NMR (101 MHz, $CDCl_3$) δ 155.2, 137.6, 128.7, 127.8, 127.5, 80.7, 79.5, 71.9, 49.4, 35.4, 28.5.

The characterization data correspond to previously reported values.[11]

***tert*-Butyl (4-methoxybenzyl)carbamate (S3)**

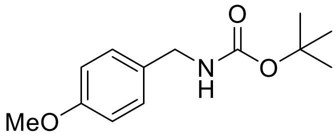

To a round bottom flask containing 4-methoxybenzylamine (4.00 mL, 4.20 g, 30.6 mmol, 1.00 equiv) in DCM (40 mL) was added a solution of di-*tert*-butyl dicarbonate (8.018 g, 36.74 mmol, 1.200 equiv.) in DCM (40 mL) dropwise at 0 °C. The solution was then stirred at room temperature for 16 h, after which the reaction was quenched with water (30 mL) and stirred at room temperature for 30 minutes. The resulting layers were separated and the aqueous fraction was extracted with DCM (3 x 30 mL). The combined organic layers were then dried over anhydrous Na_2SO_4 and the mixture was concentrated *in vacuo*. The resulting oil then precipitated upon scratching to afford a crude solid which was triturated with heptane (3 x 10 mL) and filtered to afford **S3** as a white solid (4.384 g, 18.47 mmol, 60%).

^{1}H NMR (400 MHz, $CDCl_3$) δ 7.23 – 7.17 (m, 2H, Ar*H*), 6.88 – 6.83 (m, 2H, Ar*H*), 4.76 (br s, 1H, N*H*), 4.24 (d, *J* = 5.8 Hz, 2H, C*H*$_2$), 3.80 (s, 3H, ArOC*H*$_3$), 1.46 (s, 9H, C(C*H*$_3$)$_3$).

^{13}C NMR (101 MHz, $CDCl_3$) δ 159.1, 156.0, 131.2, 129.0, 114.1, 79.5, 55.4, 44.3, 28.6.

The characterization data correspond to previously reported values.[12]

***tert*-Butyl (4-methoxybenzyl)(prop-2-yn-1-yl)carbamate (S4)**

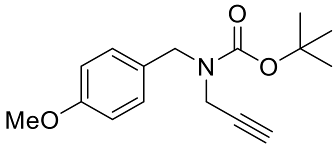

S3 (4.983 g, 21.00 mmol, 1.000 equiv.) was dissolved in DMF (25 mL) and cooled to 0 °C with an ice-water bath. NaH (60% in oil dispersion, 0.924 g, 23.1 mmol, 1.100 equiv.) was then added portionwise and the solution was stirred for 30 minutes. Propargyl bromide solution (80% wt/v in toluene, 2.70 mL, 25.2 mmol, 1.20 equiv.) was then added dropwise, and the reaction was warmed to room temperature. After 16 h, the reaction was quenched with aqueous KOH (2M, 30 mL), and extracted with EtOAc (3 x 30 mL). The combined organic layers were washed with 10 wt/v LiCl solution (50 mL), and then dried over anhydrous Na_2SO_4 and concentrated *in vacuo*. The crude residue was then purified via flash chromatography (70 g SiO_2, 5% EtOAc in pentane) to afford **S4** as a yellow oil (3.540 g, 12.86 mmol, 61%).
TLC: R_f = 0.45 (SiO_2, 10% Et_2O in pentane).

^{1}H NMR (400 MHz, $CDCl_3$) δ 7.20 (d, *J* = 8.1 Hz, 2H, Ar*H*), 6.90 – 6.82 (m, 2H, Ar*H*), 4.49 (s, 2H, C*H*$_2$), 4.07 – 3.82 (m, 2H, C*H*$_2$), 3.80 (s, 3H, ArOC*H*$_3$), 2.20 (t, *J* = 2.5 Hz, 1H, CC-H), 1.49 (s, 9H, C(C*H*$_3$)$_3$).

^{13}C NMR (101 MHz, $CDCl_3$) δ 159.1, 155.2, 129.6, 129.3, 114.1, 80.6, 79.6, 71.7, 55.4, 48.6, 35.1, 28.5.

The characterization data correspond to previously reported values.[13]

***tert*-Butyl methyl(prop-2-yn-1-yl)carbamate (S5)**

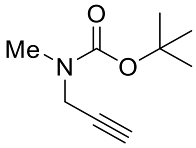

tert-Butyl N-methylcarbamate (14.90 g, 71.89 mmol, 1.000 equiv.) was dissolved in DMF (50 mL) and cooled to 0 °C with an ice bath. NaH (60% in oil dispersion, 3.163 g, 79.08 mmol, 1.100 equiv.) was then added and the solution was stirred for 30 minutes. Propargyl bromide solution (80% wt/v in toluene, 9.23 mL, 86.3 mmol, 1.20 equiv.) was then added dropwise, and the reaction was warmed to room temperature. After 16 h, the reaction was quenched with aqueous KOH (2 M, 30 mL), and extracted with EtOAc (3 x 30 mL). The combined organic layers were washed with 10% wt/v LiCl solution (50 mL), and then dried over anhydrous Na_2SO_4 and concentrated *in vacuo*. The crude residue was then purified via flash chromatography (70 g SiO_2, 5% EtOAc in pentane) to afford **S5** as a yellow oil (3.412 g, 20.17 mmol, 53%).

TLC: R_f = 0.75 (SiO_2, 10% Et_2O in pentane).

^{1}H NMR (400 MHz, $CDCl_3$) δ 4.19 – 3.87 (m, 2H), 2.91 (s, 3H), 2.20 (t, *J* = 2.5 Hz, 1H), 1.46 (s, 9H).

^{13}C NMR (101 MHz, $CDCl_3$) δ 155.4, 80.3, 79.4, 71.7, 37.8, 33.6, 28.5.

The characterization data correspond to previously reported values.[14]

Synthesis of amino cyclopropenes

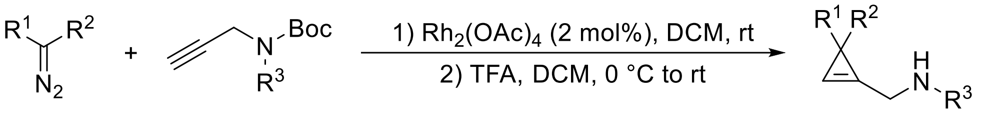

Following a modified procedure,[4] the diazo compound was dissolved in DCM (0.5 M) and added via syringe pump to a suspension of $Rh_2(OAc)_4$ (2 mol%) and the alkyne (1.5 equiv.) in DCM (0.5 M) at room temperature over 10 h. After the addition was complete, the reaction mixture was allowed to stir for another 6 hours. The reaction mixture was then concentrated under reduced pressure, and the crude residue was purified by column chromatography.

Boc-protected cyclopropene was then dissolved in DCM (0.5 M) and cooled to 0 °C. Trifluoroacetic acid (8.00 equiv.) was then added and the solution was stirred for 30 minutes at 0 °C, then warmed to room temperature and stirred for an additional hour. The reaction mixture was then concentrated in vacuo, adding toluene (5 mL) to help remove residual trifluoroacetic acid. The residue was then suspended in aqueous 1M NaOH (40 mL), and extracted with EtOAc (3 x 40 mL). The combined organic layers were then dried over anhydrous Na_2SO_4, filtered and concentrated *in vacuo*. The crude product was then purified by flash column chromatography using the indicated solvents to afford the desired cyclopropene.

Methyl 2-((benzylamino)methyl)-1-phenylcycloprop-2-ene-1-carboxylate (3a)

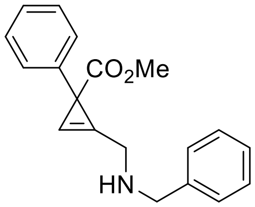

Following general procedure B, starting from diazo **S1a** (2.412 g, 13.69 mmol, 1.000 equiv.), the intermediate product was purified by flash column chromatography (SiO_2, 80 g, 10 – 20% EtOAc in pentane, R_f = 0.20 in 10% EtOAc in pentane). A portion of the Boc protected intermediate (3.307 g, 8.405 mmol, 1.000 equiv.) was immediately deprotected in TFA (8.0 equiv.) and DCM (1.0 M), then purified by flash column chromatography (SiO_2, 40 g, 25 – 55% EtOAc in pentane) to afford **3a** as an orange oil (1.769 g, 6.030 mmol, 45% over both steps).

TLC: R_f = 0.48 (SiO_2, 20% EtOAc in DCM).

^{1}H NMR (400 MHz, $CDCl_3$) δ 7.33 – 7.28 (m, 6H, Ar*H*), 7.26 – 7.19 (m, 4H, Ar*H*), 6.88 (t, *J* = 1.5 Hz, 1H, C=C-*H*), 3.90 – 3.75 (m, 4H, C*H*$_2$), 3.70 (s, 3H, CO_2CH_3).

^{13}C NMR (101 MHz, $CDCl_3$) δ 175.7, 141.3, 139.6, 128.6, 128.4, 128.4, 128.3, 127.3, 126.6, 119.7, 99.3, 53.1, 52.3, 43.3, 33.7.

HRMS (nanochip-ESI/LTQ-Orbitrap) m/z: $[M + H]^+$ Calcd for $C_{19}H_{20}NO_2^+$ 294.1489; Found 294.1488.

IR (ν_{max}, cm^{-1}) 3392 (s), 2985 (s), 2899 (s), 1717 (s), 1451 (w), 1407 (m), 1228 (s), 1053 (s).

(+)-Methyl 2-((benzylamino)methyl)-1-phenylcycloprop-2-ene-1-carboxylate ((+)-3a)

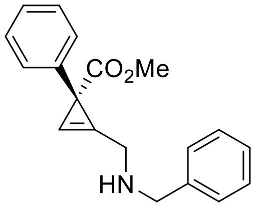

Following a modified version of general procedure B, starting from diazo **S1a** (0.505 g, 2.87 mmol, 1.00 equiv.), using alkyne **S2** (0.985 g, 4.01 mmol, 1.40 equiv.), and tetrakis[(*R*)-(+)-*N*-(p-dodecylphenylsulfonyl)prolinato]dirhodium(II) (Rh_2(*R*-DOSP)$_4$) (32 mg, 0.017 mmol, 0.60 mol%), the intermediate product was purified by flash column chromatography (SiO_2, 25 g, 10 – 20% EtOAc in pentane, R_f = 0.20 in 10% EtOAc in pentane). A portion of the Boc protected intermediate (0.400 g, 1.02 mmol, 1.00 equiv.) was immediately deprotected in TFA (0.467 mL, 6.10 mmol, 8.00 equiv.) and DCM (1.0 M), then purified by flash column chromatography (SiO_2, 40 g, 25 – 55% EtOAc in pentane) to afford **(+)-3a** as an orange oil (148 mg, 0.505 mmol, 20% over both steps). Spectra matched racemic compound. The enantiomeric ratio was determined to be 87:13 by HPLC analysis on a Daicel Chiralpak IB N-5 column: 80:20 hexane/IPA, flow rate 1 mL/min, λ= 254 nm: τ_{major} = 8.4 min, τ_{minor} = 9.2 min.

$[\alpha]_D^{20}$ = 97.4 (c = 2.43 in $CHCl_3$, 87:13 e.r.,)

Methyl 2-((benzylamino)methyl)-1-(4-chlorophenyl)cycloprop-2-ene-1-carboxylate (3b)

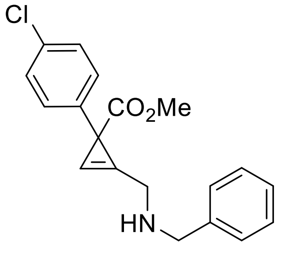

Following general procedure B, starting from diazo **S1b** (2.116 g, 10.05 mmol, 1.000 equiv.), the intermediate product was purified by flash column chromatography (SiO_2, 80 g, 15% EtOAc in pentane, R_f = 0.31 in 10% EtOAc in pentane). A portion of the Boc protected intermediate (2.102 g, 4.912 mmol, 1.000 equiv.) was immediately deprotected in TFA (8.0 equiv.) and DCM

(1.0 M), then purified by flash column chromatography (SiO_2, 25 g, 25 – 60% EtOAc in pentane) to afford **3b** as an orange oil (0.836 g, 2.55 mmol, 26% over both steps).

TLC: R_f = 0.57 (SiO_2, 40% EtOAc in pentane).

^{1}H NMR (400 MHz, $CDCl_3$) δ 7.34 – 7.26 (m, 3H, Ar*H*), 7.26 – 7.20 (m, 6H, Ar*H*), 6.85 (t, *J* = 1.5 Hz, 1H, C=C-*H*), 3.87 – 3.74 (m, 4H, C*H*$_2$, C*H*$_2$), 3.69 (s, 3H, CO_2CH_3).

^{13}C NMR (101 MHz, $CDCl_3$) δ 175.3, 139.9, 139.5, 132.4, 129.9, 128.6, 128.4, 128.4, 127.4, 119.5, 98.9, 53.1, 52.4, 43.2, 33.1.

HRMS (nanochip-ESI/LTQ-Orbitrap) m/z: [M + H]$^+$ Calcd for $C_{19}H_{19}ClNO_2^+$ 328.1099; Found 328.1102.

IR (ν_{max}, cm^{-1}) 3409 (m), 2946 (w), 2843 (w), 1718 (s), 1492 (m), 1455 (m), 1288 (m), 1223 (s), 1092 (m), 1031 (m), 1014 (m).

Methyl 2-((benzylamino)methyl)-1-(4-methoxyphenyl)cycloprop-2-ene-1-carboxylate (3c)

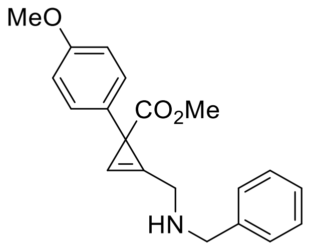

Following general procedure B, starting from diazo **S1c** (1.498 g, 7.265 mmol, 1.000 equiv.), the intermediate product was purified by flash column chromatography (SiO_2, 80 g, 10 – 20% EtOAc in pentane, R_f = 0.17 in 30% Et_2O in pentane). A portion of the Boc protected intermediate (1.458 g, 3.443 mmol, 1.000 equiv.) was immediately deprotected in TFA (8.0 equiv.) and DCM (1.0 M), then purified by flash column chromatography (SiO_2, 40 g, 25 – 55% EtOAc in pentane) to afford **3c** as an orange oil (0.830 g, 2.57 mmol, 34% over both steps).

TLC: R_f = 0.29 (SiO_2, 50% EtOAc in pentane).

^{1}H NMR (400 MHz, $CDCl_3$) δ 7.35 – 7.27 (m, 2H, Ar*H*), 7.26 – 7.17 (m, 5H, Ar*H*), 6.87 (t, *J* = 1.5 Hz, 1H, C=C-*H*), 6.85 – 6.81 (m, 2H, Ar*H*), 3.88 – 3.75 (m, 4H, C*H*$_2$, C*H*$_2$), 3.79 (s, 3H, OCH_3), 3.69 (s, 3H, CO_2CH_3).

^{13}C NMR (101 MHz, $CDCl_3$) δ 176.0, 158.4, 139.7, 133.5, 129.5, 128.6, 128.4, 127.3, 120.0, 113.7, 99.4, 55.4, 53.1, 52.3, 43.4, 33.0.

HRMS (APCI/QTOF) m/z: [M + H]$^+$ Calcd for $C_{20}H_{22}NO_3^+$ 324.1594; Found 324.1592.

IR (ν_{max}, cm^{-1}) 3354 (m), 2944 (w), 2838 (w), 1716 (s), 1612 (w), 1513 (s), 1457 (m), 1247 (s), 1029 (s), 843 (m).

Methyl 4-(2-((benzylamino)methyl)-1-(methoxycarbonyl)cycloprop-2-en-1-yl)benzoate (3d)

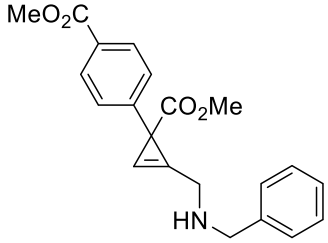

Following general procedure B, starting from diazo **S1d** (1.501 g, 6.408 mmol, 1.000 equiv.), the intermediate product was purified by flash column chromatography (SiO_2, 80 g, 20% EtOAc in pentane, R_f = 0.28 in 20% EtOAc in pentane). A portion of the Boc protected intermediate (0.948 g, 2.10 mmol, 1.00 equiv.) was immediately deprotected in TFA (8.0 equiv.) and DCM (1.0 M), then purified by flash column chromatography (SiO_2, 25 g, 25 – 60% EtOAc in pentane) to afford **3d** as a yellow oil (0.217 g, 0.614 mmol, 10% over both steps).

TLC: R_f = 0.07 (SiO_2, 50% Et_2O in pentane).

^{1}H NMR (400 MHz, $CDCl_3$) δ 7.99 – 7.93 (m, 2H, Ar*H*), 7.39 – 7.34 (m, 2H, Ar*H*), 7.32 – 7.27 (m, 2H, Ar*H*), 7.27 – 7.26 (m, 1H, Ar*H*), 7.26 – 7.22 (m, 2H, Ar*H*), 6.89 (t, *J* = 1.5 Hz, 1H, C=C-*H*), 3.90 (s, 3H, CO_2CH_3), 3.88 – 3.76 (m, 4H, CH_2, CH_2), 3.70 (s, 3H, CO_2CH_3).

^{13}C NMR (101 MHz, $CDCl_3$) δ 174.9, 167.1, 146.5, 139.1, 129.6, 128.6, 128.5, 128.4, 128.4, 127.5, 118.9, 99.1, 53.0, 52.4, 52.2, 43.0, 33.7.

HRMS (ESI/QTOF) m/z: $[M + H]^+$ Calcd for $C_{21}H_{22}NO_4^+$ 352.1543; Found 352.1542.

IR (v_{max}, cm^{-1}) 3432 (m), 2942 (m), 2844 (m), 1720 (s), 1435 (m), 1281 (s), 1116 (s), 996 (s).

Methyl 2-((benzylamino)methyl)-1-(p-tolyl)cycloprop-2-ene-1-carboxylate (3e)

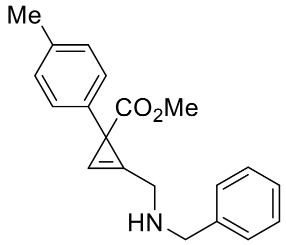

Following general procedure B, starting from diazo **S1e** (1.522 g, 8.002 mmol, 1.000 equiv.), the intermediate product was purified by flash column chromatography (SiO_2, 80 g, 10 – 20% EtOAc in pentane, R_f = 0.38 in 10% Et_2O in pentane). A portion of the Boc protected intermediate (1.126 g, 2.763 mmol, 1.000 equiv.) was immediately deprotected in TFA (8.0 equiv.) and DCM (1.0 M), then purified by flash column chromatography (SiO_2, 25 g, 20 – 60% EtOAc in pentane) to afford **3e** as an orange oil (0.521 g, 1.70 mmol, 22% over both steps).

TLC: R_f = 0.28 (SiO_2, 20% EtOAc in pentane).

^{1}H NMR (400 MHz, $CDCl_3$) δ 7.35 – 7.27 (m, 2H, Ar*H*), 7.26 – 7.22 (m, 3H, Ar*H*), 7.20 – 7.15 (m, 2H, Ar*H*), 7.11 (d, *J* = 8.1 Hz, 2H, Ar*H*), 6.87 (t, *J* = 1.5 Hz, 1H, C=C-*H*), 3.89 – 3.75 (m, 4H, CH_2, CH_2), 3.69 (s, 3H, CO_2CH_3), 2.33 (s, 3H, $ArCH_3$).

^{13}C NMR (101 MHz, $CDCl_3$) δ 175.9, 139.7, 138.3, 136.3, 129.0, 128.6, 128.4, 128.3, 127.3, 119.8, 99.4, 53.1, 52.3, 43.4, 33.4, 21.2.

HRMS (ESI/QTOF) m/z: $[M + H]^+$ Calcd for $C_{20}H_{22}NO_2^+$ 308.1645; Found 308.1648.

IR (v_{max}, cm^{-1}) 3332 (w), 3130 (w), 3032 (w), 2946 (w), 1715 (s), 1513 (m), 1453 (m), 1286 (m), 1224 (s), 1207 (s), 1030 (m).

Methyl 2-((benzylamino)methyl)-1-(2-fluorophenyl)cycloprop-2-ene-1-carboxylate (3f)

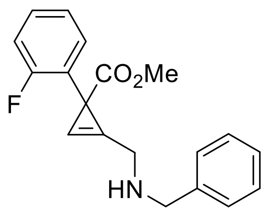

Following general procedure B, starting from diazo **S1f** (1.165 g, 6.000 mmol, 1.000 equiv.), the intermediate product was purified by flash column chromatography (SiO_2, 80 g, 10 – 20% EtOAc in pentane, R_f = 0.68 in 20% EtOAc in pentane). A portion of the Boc protected intermediate (0.874 g, 1.86 mmol, 1.00 equiv.) was immediately deprotected in TFA (8.00 equiv.) and DCM (1.0 M), then purified by flash column chromatography (SiO_2, 25 g, 10 – 50% EtOAc in pentane) to afford **3f** as a yellow oil (0.710 g, 2.28 mmol, 39% over both steps).

TLC: R_f = 0.35 (SiO_2, 25% EtOAc in pentane).

^{1}H NMR (400 MHz, $CDCl_3$) δ 7.35 – 7.23 (m, 6H, Ar*H*), 7.19 (dd, *J* = 8.5, 6.1 Hz, 1H, Ar*H*), 7.09 (dd, *J* = 8.5, 2.6 Hz, 1H, Ar*H*), 6.96 (d, *J* = 1.4 Hz, 1H, C=C-*H*), 6.95 – 6.89 (m, 1H, Ar*H*), 3.99 – 3.76 (m, 5H, CH_2, CH_2), 3.66 (s, 3H, CO_2CH_3).

^{13}C NMR (101 MHz, $CDCl_3$) δ 175.0, 161.7 (d, $J_{C\text{-}F}$ = 249.0 Hz), 139.5, 136.3 (d, $J_{C\text{-}F}$ = 3.7 Hz), 135.9 (d, $J_{C\text{-}F}$ = 10.4 Hz), 131.4 (d, $J_{C\text{-}F}$ = 8.8 Hz), 128.6, 128.4, 127.4, 120.6, 116.8 (d, $J_{C\text{-}F}$ = 24.6 Hz), 114.3 (d, $J_{C\text{-}F}$ = 21.1 Hz), 100.3, 53.1, 52.6, 43.6, 32.7.

^{19}F NMR (376 MHz, $CDCl_3$) δ -113.13 – -113.24 (m).

HRMS (ESI/QTOF) m/z: $[M + H]^+$ Calcd for $C_{19}H_{19}FNO_2^+$ 312.1394; Found 312.1396.

IR (ν_{max}, cm^{-1}) 3337 (m), 3138 (m), 2987 (m), 2954 (m), 2900 (m), 1720 (s), 1491 (s), 1453 (s), 1296 (m), 1256 (s), 1227 (s), 1122 (m), 828 (m), 759 (s).

Methyl 2-((benzylamino)methyl)-1-(3-methoxyphenyl)cycloprop-2-ene-1-carboxylate (3g)

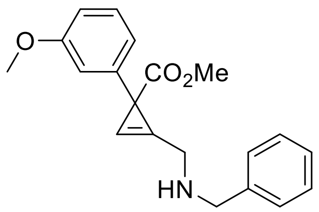

Following general procedure B, starting from diazo **S1g** (1.237 g, 6.000 mmol, 1.000 equiv.), the intermediate product was purified by flash column chromatography (SiO_2, 80 g, 10 – 35% Et_2O in pentane, R_f = 0.20 in 20% EtOAc in pentane). A portion of the Boc protected intermediate (0.852 g, 2.01 mmol, 1.00 equiv.) was immediately deprotected in TFA (8.00 equiv.) and DCM (1.0 M), then purified by flash column chromatography (SiO_2, 25 g, 20 – 60% EtOAc in pentane) to afford **3g** as an orange oil (0.442 g, 1.37 mmol, 68% over both steps).

TLC: R_f = 0.13 (SiO_2, 20% EtOAc in pentane)

^{1}H NMR (400 MHz, $CDCl_3$) δ 7.37 – 7.30 (m, 2H, Ar*H*), 7.28 – 7.21 (m, 4H, Ar*H*), 6.93 – 6.84 (m, 3H. Ar*H*, C=C-*H*), 6.80 (ddd, *J* = 8.2, 2.6, 0.9 Hz, 1H, Ar*H*), 3.91 – 3.77 (m, 4H, CH_2, CH_2), 3.81 (s, 3H, ArOCH_3), 3.72 (s, 3H, CO_2CH_3).

^{13}C NMR (101 MHz, $CDCl_3$) δ 175.6, 159.6, 143.0, 139.7, 129.3, 128.6, 128.4, 127.3, 120.8, 119.6, 114.4, 112.0, 99.3, 55.3, 53.2, 52.3, 43.4, 33.7.

HRMS (ESI/QTOF) m/z: $[M + H]^+$ Calcd for $C_{20}H_{22}NO_3^+$ 324.1594; Found 324.1601.

IR (v_{max}, cm^{-1}) 3325 (m), 3131 (m), 2959 (s), 2905 (s), 1717 (s), 1603 (m), 1583 (m), 1490 (m), 1454 (m), 1433 (m), 1290 (m), 1236 (s), 1047 (s), 767 (m).

Methyl 2-((benzylamino)methyl)-1-(2-chloro-4-fluorophenyl)cycloprop-2-ene-1-carboxylate (3h)

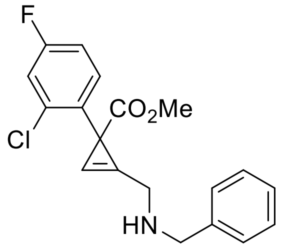

Following general procedure B, starting from diazo **S1h** (1.829 g, 8.000 mmol, 1.000 equiv.), the intermediate product was purified by flash column chromatography (SiO_2, 40 g, 15 – 35% EtOAc in pentane, R_f = 0.29 in 20% Et_2O in pentane). A portion of the Boc protected intermediate (1.490 g, 3.341 mmol, 1.000 equiv.) was immediately deprotected in TFA (8.0 equiv.) and DCM (1.0 M), then purified by flash column chromatography (SiO_2, 25 g, 20 – 60% EtOAc in pentane) to afford **3h** as an orange oil (0.519 g, 1.50 mmol, 19% over both steps).

TLC: R_f = 0.34 (SiO_2, 25% EtOAc in pentane).

^{1}H NMR (400 MHz, $CDCl_3$) δ 7.34 – 7.26 (m, 5H, Ar*H*), 7.19 (dd, *J* = 8.5, 6.0 Hz, 1H, Ar*H*), 7.09 (dd, *J* = 8.5, 2.6 Hz, 1H, Ar*H*), 6.95 (t, *J* = 1.5 Hz, 1H, C=C-*H*), 6.92 (dd, *J* = 8.3, 2.6 Hz, 1H, Ar*H*), 3.94 (dd, *J* = 17.1, 1.5 Hz, 1H, CH_2), 3.86 (dd, *J* = 14.0, 1.5 Hz, 1H, CH_2), 3.85 (d, *J* = 13.2 Hz, 1H, CH_2), 3.80 (d, *J* = 13.0 Hz, 1H, CH_2), 3.66 (s, 3H, CO_2CH_3).

^{13}C NMR (101 MHz, $CDCl_3$) δ 175.1, 161.7 (d, J_{C-F} = 249.1 Hz), 139.6, 136.3 (d, J_{C-F} = 3.6 Hz), 135.9 (d, J_{C-F} = 10.4 Hz), 131.4 (d, J_{C-F} = 8.8 Hz), 128.6, 128.4, 127.3, 120.7, 116.9 (d, J_{C-F} = 24.8 Hz), 114.3 (d, J_{C-F} = 20.9 Hz), 100.2, 53.2, 52.6, 43.7, 32.7.

^{19}F NMR (376 MHz, $CDCl_3$) δ -116.4 – -116.5 (m).

HRMS (nanochip-ESI/LTQ-Orbitrap) m/z: $[M + H]^+$ Calcd for $C_{19}H_{18}ClFNO_2^+$ 346.1005; Found 346.1006.

IR (v_{max}, cm^{-1}) 3331 (w), 3039 (w), 2951 (w), 2822 (w), 1723 (s), 1602 (m), 1490 (m), 1258 (s), 1228 (s), 1052 (m).

Benzyl 2-((benzylamino)methyl)-1-phenylcycloprop-2-ene-1-carboxylate (3i)

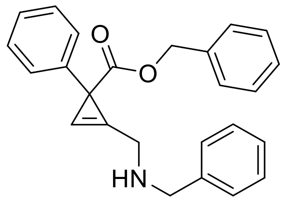

Following general procedure B, starting from diazo **S1i** (1.986 g, 7.871 mmol, 1.000 equiv.), the intermediate product was purified by flash column chromatography (SiO_2, 80 g, 10 – 20% EtOAc in pentane, R_f = 0.29 in 10% EtOAc in pentane). A portion of the Boc protected intermediate (0.874 g, 1.86 mmol, 1.00 equiv.) was immediately deprotected in TFA (8.00 equiv.) and DCM (1.0 M), then purified by flash column chromatography (SiO_2, 25 g, 20 – 60% EtOAc in pentane) to afford **3i** as an orange oil (0.485 g, 1.313 mmol, 53% over both steps).

TLC: R_f = 0.25 (SiO_2, 20% EtOAc in pentane).

^{1}H NMR (400 MHz, $CDCl_3$) δ 7.34 – 7.26 (m, 11H, Ar*H*), 7.26 – 7.18 (m, 4H, Ar*H*), 6.88 (t, *J* = 1.5 Hz, 1H, C=C-*H*), 5.15 (s, 2H, CO_2CH_2-Ar), 3.83 – 3.80 (m, 2H, C*H*$_2$), 3.79 – 3.70 (m, 2H, C*H*$_2$).

^{13}C NMR (101 MHz, $CDCl_3$) δ 174.9, 141.2, 139.6, 136.5, 128.6, 128.6, 128.4, 128.4, 128.2, 128.1, 128.0, 127.3, 126.6, 119.5, 99.2, 66.6, 53.1, 43.3, 33.8.

HRMS (ESI/QTOF) m/z: [M + H]$^+$ Calcd for $C_{25}H_{24}NO_2^+$ 370.1802; Found 370.1812.

IR (ν_{max}, cm^{-1}) 3334 (w), 3134 (w), 3061 (m), 3029 (m), 2938 (w), 1717 (s), 1602 (w), 1496 (m), 1452 (m), 1281 (m), 1209 (s), 1008 (m), 826 (m), 739 (s).

N-Benzyl-1-(3-phenyl-3-(trifluoromethyl)cycloprop-1-en-1-yl)methanamine (3j)

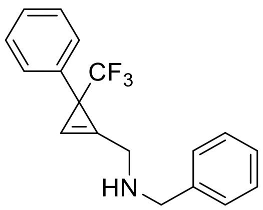

Following general procedure B, starting from diazo **S1j** (1.024 g, 5.501 mmol, 1.000 equiv.), the intermediate product was purified by flash column chromatography (SiO_2, 100 g, 5% Et_2O in pentane, R_f = 0.21 in 5% Et_2O in pentane). A portion of the Boc protected intermediate (0.954 g, 2.36 mmol, 1.00 equiv.) was immediately deprotected in TFA (8.00 equiv.) and DCM (1.0 M), then purified by flash column chromatography (SiO_2, 12 g, 20 – 50% EtOAc in pentane) to afford **3j** as a clear oil (0.555 g, 1.89 mmol, 35% over both steps).

TLC: R_f = 0.23 (SiO_2, 20% Et_2O in pentane).

^{1}H NMR (400 MHz, $CDCl_3$) δ 7.36 – 7.21 (m, 10H, Ar*H*), 6.91 (h, *J* = 1.6 Hz, 1H, C=C-*H*), 3.91 – 3.69 (m, 4H, C*H*$_2$, C*H*$_2$).

^{13}C NMR (101 MHz, $CDCl_3$) δ 139.5, 138.5, 128.6, 128.6, 128.4, 127.9 (q, J_{C-F} = 1.2 Hz), 127.4, 127.3, 126.7 (d, J_{C-F} = 277.6 Hz), 119.6 (q, J_{C-F} = 2.1 Hz), 99.5 (q, J_{C-F} = 3.1 Hz), 53.2, 43.2, 32.36 (q, J_{C-F} = 35.3 Hz).

^{19}F NMR (376 MHz, $CDCl_3$) δ -64.6.

HRMS (ESI/QTOF) m/z: [M + H]$^+$ Calcd for $C_{18}H_{17}F_3N^+$ 304.1308; Found 304.1310.

IR (ν_{max}, cm^{-1}) 3332 (w), 3138 (w), 3064 (w), 3030 (w), 2818 (w), 1496 (w), 1453 (m), 1301 (m), 1159 (s), 1121 (s), 909 (m), 740 (m).

Methyl 2-(((4-methoxybenzyl)amino)methyl)-1-phenylcycloprop-2-ene-1-carboxylate (3k)

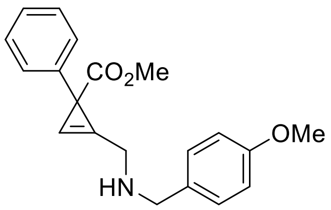

Following general procedure B, starting from diazo **S1a** (1.409 g, 8.000 mmol, 1.000 equiv.), and alkyne **S4** (2.753 g, 10.00 mmol, 1.250 equiv.) the intermediate product was purified by flash column chromatography (SiO_2, 80 g, 10 – 20% EtOAc in pentane, R_f = 0.24 in 20% Et_2O

in pentane). A portion of the Boc protected intermediate (1.802 g, 4.255 mmol, 1.000 equiv.) was immediately deprotected in TFA (8.00 equiv.) and DCM (1.0 M), then purified by flash column chromatography (SiO_2, 12 g, 25 – 55% EtOAc in pentane) to afford **3k** as an orange oil (0.813 g, 2.52 mmol, 31% over both steps).

TLC: R_f = 0.17 (SiO_2, 30% EtOAc in pentane).

^{1}H NMR (400 MHz, $CDCl_3$) δ 7.34 – 7.26 (m, 4H, Ar*H*), 7.25 – 7.20 (m, 1H, Ar*H*), 7.18 – 7.11 (m, 2H, Ar*H*), 6.88 (t, *J* = 1.5 Hz, 1H, C=C-*H*), 6.86 – 6.79 (m, 2H, Ar*H*), 3.87 – 3.76 (m, 2H, CH_2), 3.79 (s, 3H, $ArOCH_3$), 3.77 – 3.68 (m, 5H, CH_2, CO_2CH_3).

^{13}C NMR (101 MHz, $CDCl_3$) δ 175.7, 158.9, 141.3, 131.7, 129.6, 128.4, 128.3, 126.6, 119.7, 113.9, 99.2, 55.4, 52.5, 52.3, 43.2, 33.7.

HRMS (ESI/QTOF) m/z: $[M + Na]^+$ Calcd for $C_{20}H_{21}NNaO_3^+$ 346.1414; Found 346.1421.

IR (v_{max}, cm^{-1}) 3331 (w), 3131 (w), 2984 (s), 2904 (s), 1716 (s), 1611 (w), 1512 (m), 1447 (m), 1290 (m), 1245 (s), 1229 (s), 1178 (m), 1104 (m), 1070 (s), 1040 (s), 823 (m), 765 (m).

Methyl 2-((methylamino)methyl)-1-phenylcycloprop-2-ene-1-carboxylate (3l)

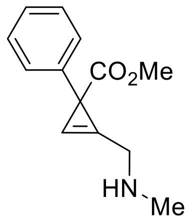

Following general procedure B, starting from diazo **S1a** (2.005 g, 11.38 mmol, 1.000 equiv.), and alkyne **S5** (2.407 g, 14.23 mmol, 1.250 equiv.) the intermediate product was purified by flash column chromatography (SiO_2, 80 g, 10 – 20% EtOAc in pentane, R_f = 0.19 in 20% Et_2O in pentane). A portion of the Boc protected intermediate (1.092 g, 3.440 mmol, 1.000 equiv.) was immediately deprotected in TFA (8.00 equiv.) and DCM (1.0 M), then purified by flash column chromatography (SiO_2, 25 g, 40 – 100% EtOAc in DCM) to afford **3l** as an orange oil (0.258 g, 1.19 mmol, 15% over both steps).

TLC: R_f = 0.06 (SiO_2, EtOAc).

^{1}H NMR (400 MHz, $CDCl_3$) δ 7.33 – 7.26 (m, 4H, Ar*H*), 7.24 – 7.18 (m, 1H, Ar*H*), 6.87 (t, *J* = 1.5 Hz, 1H, C=C-*H*), 3.85 – 3.72 (m, 2H, CH_2), 3.69 (s, 3H, CO_2CH_3), 2.46 (s, 3H. $NHCH_3$).

^{13}C NMR (101 MHz, $CDCl_3$) δ 175.7, 141.3, 128.3, 128.3, 126.6, 119.5, 99.1, 52.3, 46.0, 36.1, 33.6.

HRMS (ESI/QTOF) m/z: $[M + H]^+$ Calcd for $C_{13}H_{16}NO_2^+$ 218.1176; Found 218.1176.

IR (v_{max}, cm^{-1}) 3330 (w), 3131 (w), 2984 (s), 2901 (s), 2787 (w), 1715 (s), 1493 (m), 1437 (m), 1283 (m), 1224 (s), 1071 (s), 1042 (s), 890 (w), 824 (w), 766 (m).

Methyl 2-((benzylamino)methyl)-1-methylcycloprop-2-ene-1-carboxylate (3m)

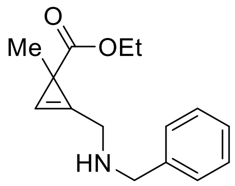

Following general procedure B, starting from diazo **S1k** (1.025 g, 8.000 mmol, 1.000 equiv.), the intermediate product was purified by flash column chromatography (SiO_2, 40 g, 10 – 20% Et_2O in pentane, R_f = 0.30 in 20% Et_2O in pentane). A portion of the Boc protected intermediate (0.190 g, 0.573 mmol, 1.00 equiv.) was immediately deprotected in TFA (8.00 equiv.) and DCM (1.0 M), then purified by flash column chromatography (SiO_2, 4 g, 20 – 50% EtOAc in pentane) to afford **3m** as a clear oil (82 mg, 0.36 mmol, 4% over both steps).

TLC: R_f = 0.07 (SiO_2, 20% Et_2O in pentane).

^{1}H NMR (400 MHz, $CDCl_3$) δ 7.33 (s, 4H, Ar*H*), 7.30 – 7.22 (m, 1H, Ar*H*), 6.64 (s, 1H, C=C-*H*), 4.15 – 4.02 (m, 2H, $CO_2CH_2CH_3$), 3.87 (s, 2H, ArCH_2), 3.74 (d, *J* = 1.5 Hz, 2H,C=C-CH_2), 1.40 (s, 3H, CH_3), 1.22 (t, *J* = 7.1 Hz, 3H, $CO_2CH_2CH_3$).

^{13}C NMR (101 MHz, $CDCl_3$) δ 177.2, 139.8, 128.6, 128.4, 127.3, 120.1, 102.4, 60.6, 53.2, 43.3, 25.0, 20.7, 14.5.

HRMS (ESI/QTOF) m/z: $[M + H]^+$ Calcd for $C_{15}H_{20}NO_2^+$ 246.1489; Found 246.1494.

IR (v_{max}, cm^{-1}) 3332 (w), 3127 (w), 2975 (m), 2928 (m), 1710 (s), 1453 (m), 1253 (s), 1112 (s), 1029 (m), 803 (m), 740 (s).

tert-Butyl benzyl((3-(hydroxymethyl)-3-phenylcycloprop-1-en-1-yl)methyl)carbamate (3n)

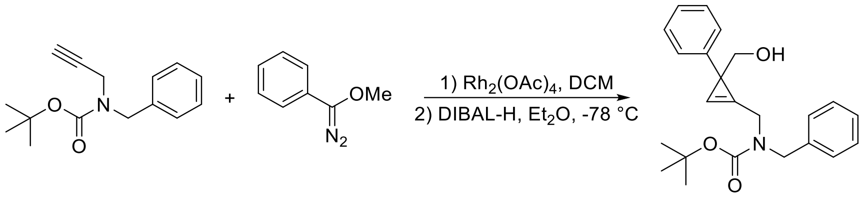

Following general procedure B, starting from diazo **S1a** (2.412 g, 13.69 mmol, 1.000 equiv.), the intermediate product was purified by flash column chromatography (SiO_2, 80 g, 10 – 20% EtOAc in pentane, R_f = 0.20 in 10% EtOAc in pentane). A portion of the Boc protected intermediate (1.023 g, 2.600 mmol, 1.000 equiv.) was then dissolved in anhydrous diethyl ether (25 mL) and cooled to -78 °C. A solution of DIBAL-H (1.0 M solution in toluene, 4.33 mL, 5.20 mmol, 2.00 equiv.) was then added to the reaction mixture dropwise. The mixture was stirred for 2 hours at -78 °C, then warmed to room temperature and stirred for an additional 1 hour. The mixture was then quenched with sat. aq. NH_4Cl (5 mL), then acidified with 1 M HCl (40 mL), and extracted with diethyl ether (3 x 40 mL). The combined organic layers were washed with brine (40 mL), dried over anhydrous Na_2SO_4, filtered and concentrated *in vacuo*. The crude residue was then purified with flash column chromatography (SiO_2, 25 g, 10 - 30% EtOAc in hexanes) to afford alcohol **3n** as a yellow oil (0.5048 g, 1.381 mmol, 53%, 38% over 2 steps).

TLC: R_f = 0.52 (SiO_2, 30% EtOAc in pentane).

^{1}H NMR (400 MHz, $CDCl_3$) δ 7.35 – 7.26 (m, 5H, Ar*H*), 7.25 – 7.10 (m, 5H, Ar*H*), 6.88 (s, 1H, C=C*H*), 4.51 (d, *J* = 15.3 Hz, 1H, HOCH_2), 4.47 – 4.32 (m, 1H, ArCH_2), 4.31 – 4.15 (m, 2H, HOCH_2, ArCH_2), 4.13 – 3.94 (m, 1H, C=C-CH_2), 3.85 (s, 1H, C=C-CH_2), 2.77 (br s, 1H, O*H*), 1.45 (s, 9H, $C(CH_3)_3$).

^{13}C NMR (101 MHz, $CDCl_3$) δ 156.3, 145.7, 137.6, 128.6, 128.1, 127.5, 126.2, 125.7, 121.6, 105.3, 81.1, 66.9, 51.5, 42.4, 32.5, 28.3.

HRMS (ESI/QTOF) m/z: $[M + Na]^+$ Calcd for $C_{23}H_{27}NNaO_3^+$ 388.1883; Found 388.1887.

IR (v_{max}, cm^{-1}) 3450 (w), 3061 (w), 2981 (m), 2944 (w), 2880 (w), 2834 (w), 1693 (s), 1494 (m), 1453 (m), 1409 (m), 1366 (m), 1281 (m), 1245 (s), 1164 (s), 1125 (m), 1069 (m), 983 (s), 914 (s), 845 (m).

***N*-Benzyl-1-(3-(((*tert*-butyldimethylsilyl)oxy)methyl)-3-phenylcycloprop-1-en-1-yl)methanamine (3o)**

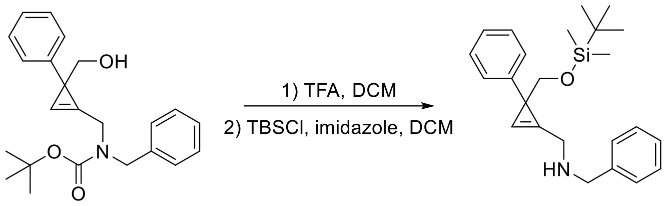

Alcohol **3n** (0.5482 g, 1.500 mmol, 1.000 equiv.) was dissolved in DCM (10 mL), cooled to 0 °C and TFA (0.919 mL, 12.0 mmol, 8.00 equiv.) was added. The reaction was then stirred at 0 °C for 30 minutes, allowed to warm to room temperature and then stirred for another 30 minutes. The reaction mixture was then concentrated *in vacuo*, and the crude residue was suspended in sat. aq. K_2CO_3 (30 mL) and extracted with DCM (3 x 30 mL). The combined organic layers were then dried over anhydrous Na_2SO_4, filtered and concentrated *in vacuo*. The crude amino alcohol was then dissolved in anhydrous DCM (20 mL) and TBSCl (339.1 mg, 2.250 mmol, 1.500 equiv.) and imidazole (204.2 mg, 3.000 mmol, 2.000 equiv.) were added, and the reaction was stirred at room temperature for 4 h. The reaction was then quenched with sat. aq. NH4Cl (30 mL), diluted with DCM (20 mL) and separated. The aqueous fraction was then extracted again with DCM (2 x 30 mL), and the combined organic layers were dried over anhydrous Na_2SO_4, filtered and concentrated *in vacuo*. The crude product was then purified by flash column chromatography (SiO2, 12 g, 5 – 20% EtOAc in pentane) to afford **3o** as a pale-yellow oil (126 mg, 0.331 mmol, 22%).

TLC: R_f = 0.37 (SiO_2, 15% EtOAc in pentane).

^{1}H NMR (400 MHz, $CDCl_3$) δ 7.33 – 7.26 (m, 5H, Ar*H*), 7.26 – 7.21 (m, 4H, Ar*H*), 7.19 – 7.13 (m, 1H, Ar*H*), 6.89 (t, *J* = 1.5 Hz, 1H, C=C*H*), 4.21 (dd, *J* = 10.4, 0.6 Hz, 1H, SiOCH_2), 3.92 (d, *J* = 10.4 Hz, 1H, SiOCH_2), 3.87 – 3.79 (m, 2H, C=C-CH_2), 3.77 (d, *J* = 1.5 Hz, 2H, ArCH_2), 0.87 (s, 9H, OSiC(CH_3)$_3$), 0.02 (d, *J* = 3.9 Hz, 6H, OSi(CH_3)$_2$).

13C NMR (101 MHz, $CDCl_3$) δ 146.6, 140.0, 128.5, 128.4, 128.1, 127.2, 126.6, 125.4, 124.2, 104.0, 69.2, 53.4, 44.1, 32.1, 26.1, 18.4, -5.1 (2 x CH_3Si).

HRMS (QTOF) m/z: $[M + H]^+$ Calcd for $C_{24}H_{34}NOSi^+$ 380.2404; Found 380.2405.

IR (ν_{max}, cm^{-1}) 3326 (w), 3085 (w), 3060 (w), 3027 (w), 2953 (m), 2929 (m), 2889 (m), 2856 (m), 1767 (w), 1712 (w), 1602 (w), 1495 (w), 1467 (m), 1254 (m), 1108 (m).

Optimization Studies

General Procedure for Ligand Optimization: An oven-dried 8 mL microwave tube equipped with a stirring bar was placed in a nitrogen-filled glovebox and charged with $Pd_2dba_3\cdot CHCl_3$ (2.9 mg, 2.8 µmol, 4.0 mol%), ligand (8.4 µmol, 12 mol%) and Cs_2CO_3 (30 mg, 91 µmol, 1.3 equiv.). The vial was sealed and taken out of the glove box, then toluene (0.35 mL) was added, and the vial was placed in a preheated aluminum block and stirred at 80 °C for 15 minutes. Then, cyclopropene **3a** (20.5 mg, 70.0 µmol, 1.00 equiv.), trifluoroacetaldehyde ethyl hemiacetal (14 µL, 0.11 mmol, 1.5 equiv.), aryl iodide (0.105 mmol, 1.50 equiv.), and remaining toluene (0.35 mL) was added, and the solution was stirred at 100 °C for 16 h. The reaction was then cooled to room temperature, filtered through a plug of silica gel eluting with EtOAc (~8 mL), and concentrated *in vacuo*. The crude reaction mixture was then dissolved in $CDCl_3$ and yield was determined by ^{1}H NMR, using trichloroethylene as an internal standard.

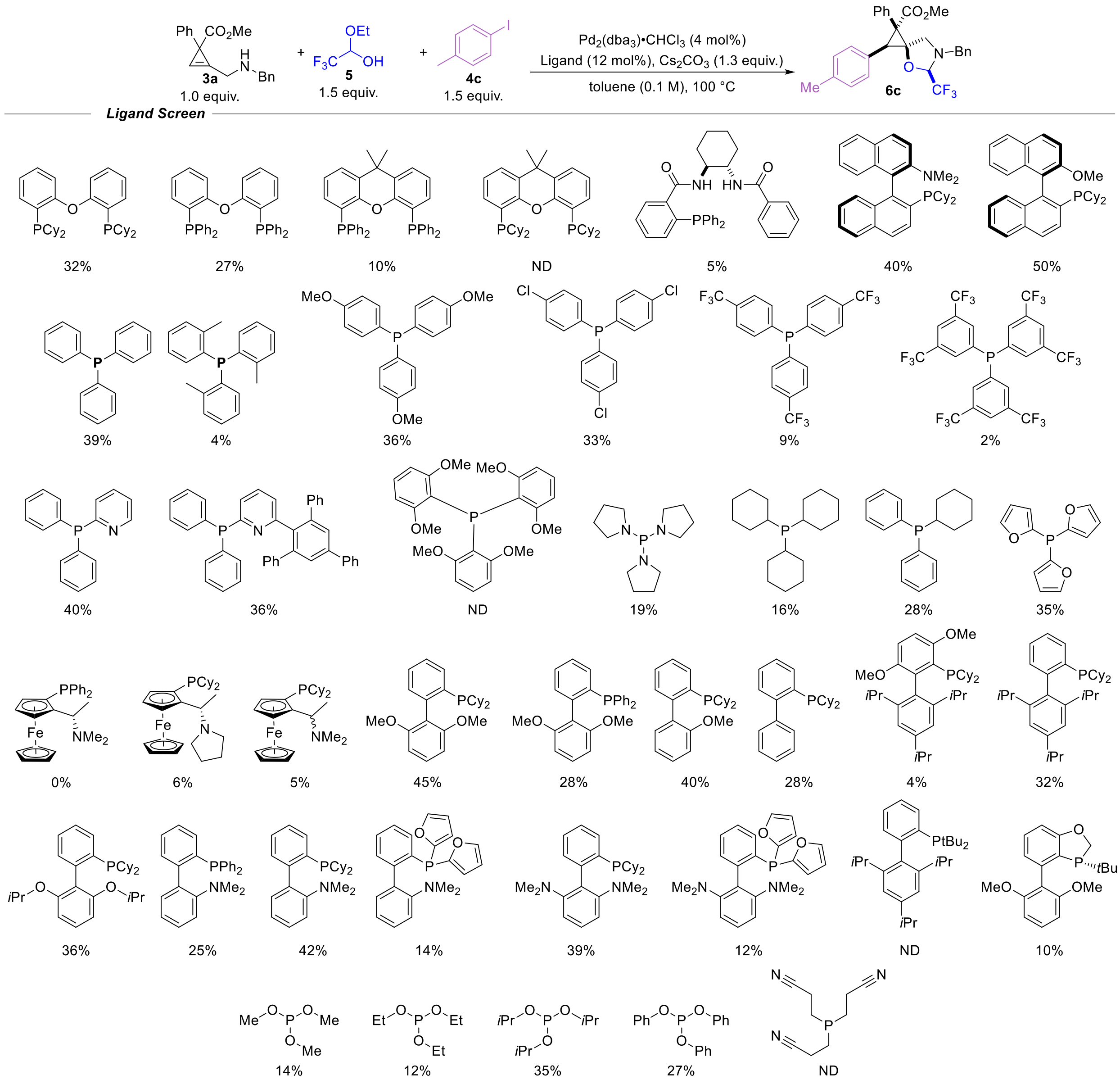

Scheme S1. Ligand screen for the tethered carboetherification of cyclopropenes. Reactions performed according to optimization procedure presented *vide supra*. N.D. = not detected.

Data-driven ligand optimization

The code to reproduce these results is available at GitHub - aa-schoepfer/carboether_of_cyclopropenes

Ligand screening for product **6c** was supported by data-driven multivariate linear regression (MLR) models. Featurized monophosphine ligands were taken from the Kraken data set.[16] For modeling, we used Bayesian ridge regression in combination with forward sequential feature selection, applying leave-one-out cross-validation with negative mean squared error as the scoring metric and a tolerance of 0.001, implemented in scikit-learn.[17,18] Entries with no yield were excluded, and percent yields were converted into fractions for improved regularization (e.g., 10% to 0.1). For visualization, principal component analysis (PCA) was performed either on the full set of available features or on the subset selected by the MLR models.

In the small-data regime, MLR models tend to overfit, yielding inaccurate predictions on out-of-sample entries. As a rule of thumb, this can be identified when the mean error approaches 0% on training data. To reduce bias, we employed automated feature selection for model construction. However, this approach was only feasible after at least 24 entries, at which point the model achieved a mean absolute error (MAE) of 7% yield on the training set (Figure S1a).

A final round of readily available ligands was tested despite not being predicted to provide major improvements (Figure S1). As expected, no significant improvement was observed, as no ligand exceeded the current best performance of 45%. The model was then retrained with the newly obtained data and yielded an MAE of 6% yield on the training data (Figure S2a).

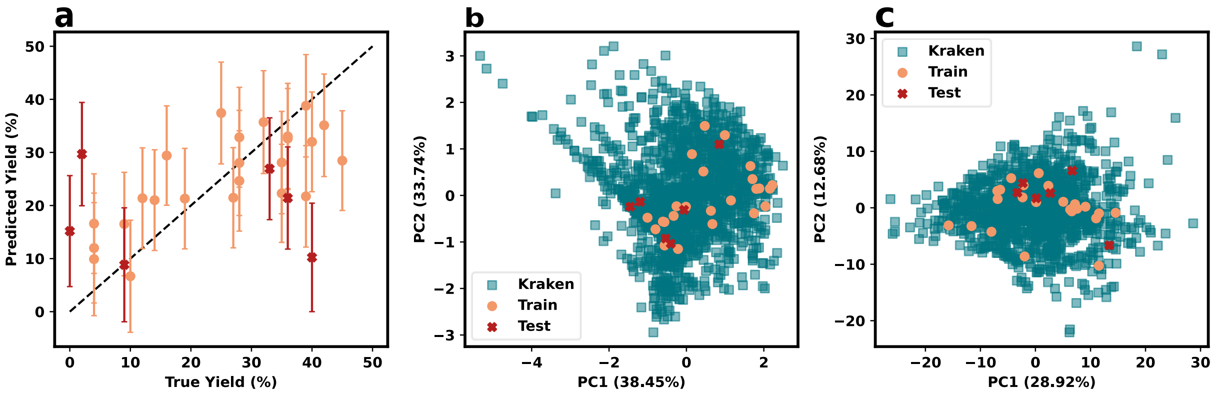

Figure S1. a) Correlation plot of the used model. Orange dots represent train points, red crosses test points. Train MAE of 7% and Test MAE of 16%. Error bars represent 1σ given by the Bayesian ride regression method. b) PCA of the model-selected feature subset. The explained variance ratio is found in the axis titles. c) PCA of the fully Kraken feature set.

We next screened the Kraken dataset for promising and easily accessible ligands (Figure S2). Among the top ten predicted candidates, only one ligand was predicted to exceed 50% yield, while the 10th-ranked candidate was already below the current best yield of 45%. PCA of the model-selected features revealed that most of the top candidates are far removed from previously tested ligands in feature space, suggesting they are likely false “better-than-best” predictions (Figure S2b), or at least, very exploratory Moreover, decomposition of the MLR predictions into the individual contribution of each feature (Scheme S2 and Scheme S3), shows that the top 2 candidates predict higher yields due to a steric feature (vbur_qvbur_min_max) for which there is no comparable precedent in the current training data. Reversely, PCA on the full feature set reveals that most of the predicted candidates are within or close to the space of already tested ligands. Taken together, these results suggest that monophosphine ligand screening within the Kraken dataset is approaching a performance plateau. This finding motivated us to shift focus away from ligand optimization toward other parameters, which ultimately proved to be key for achieving successful general optimization.

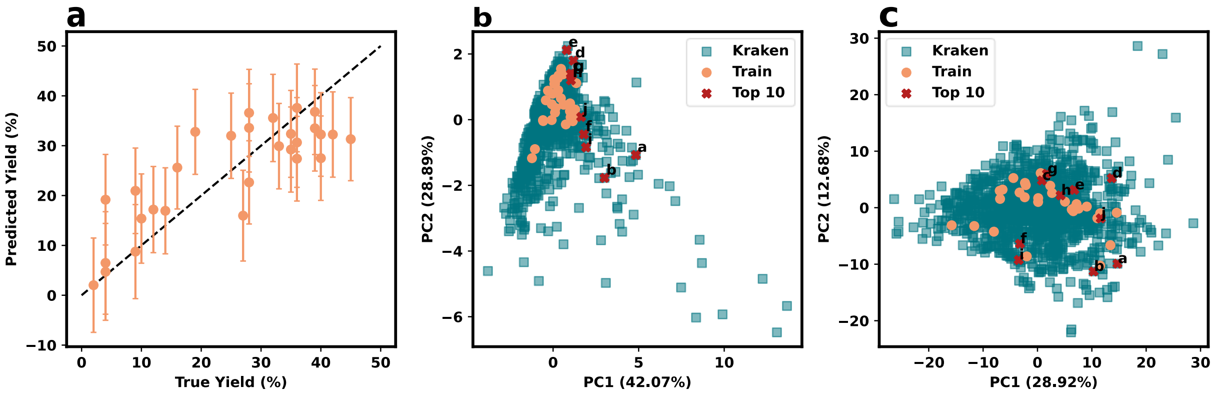

Figure S2. a) Correlation plot of the used model. Full MAE of 6%. b) PCA of the model-selected feature subset with the top 10 candidates. The explained variance ratio is found in the axis titles. c) PCA of the fully Kraken feature set with the top 10 candidates.

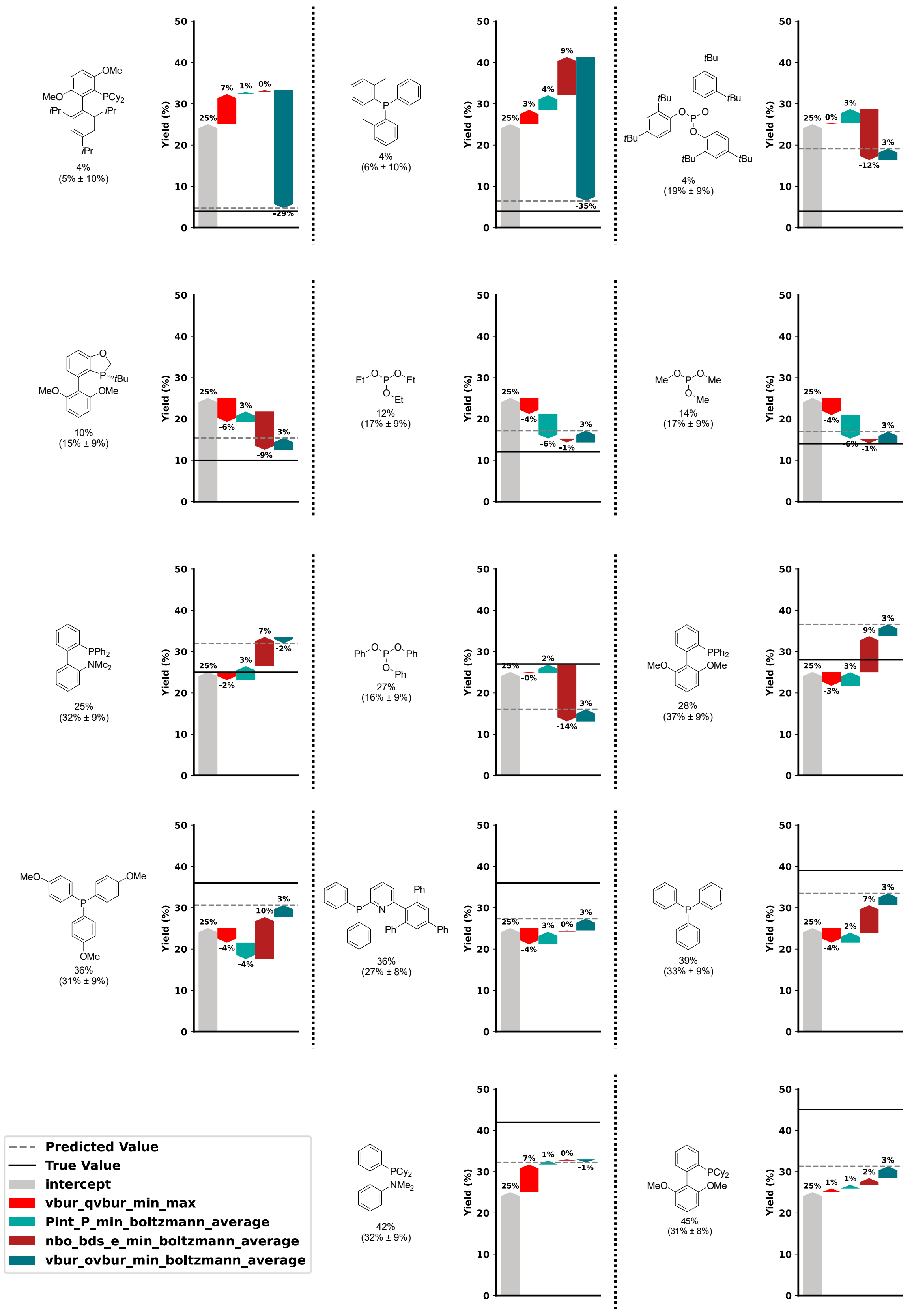

Scheme S2. Examples of ligand structures and attributions of features of training set.

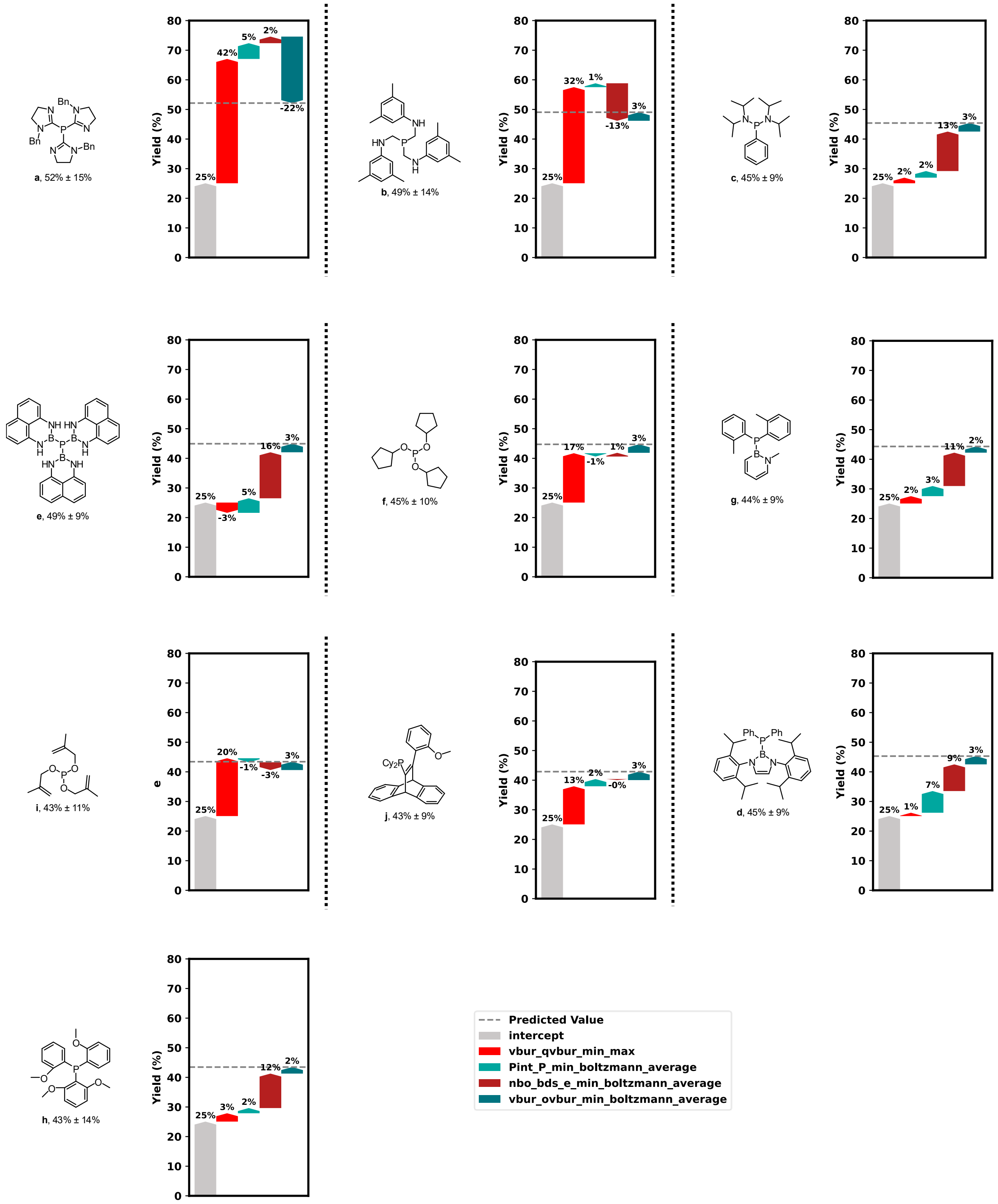

Scheme S3. Structures of top 10 predicted ligand structures within the Kraken data set.

Table S1. Optimization of catalyst system using 4-iodobenzotrifluoride.

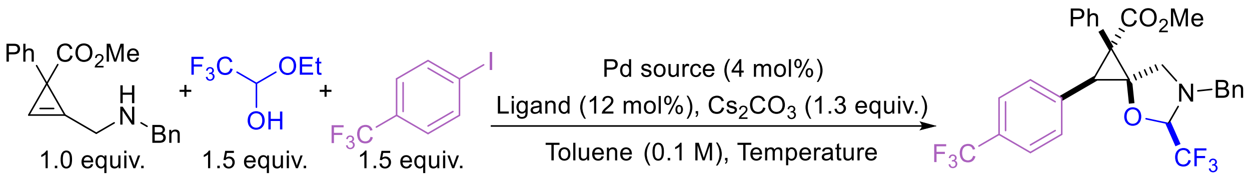

Entry	[Pd] and Ligand	Temperature	Yield[a]
1	N/A	80 °C	N.D.
2	Pd_2dba_3•$CHCl_3$/SPhos	80 °C	53%
3	Pd_2dba_3•$CHCl_3$/XantPhos	80 °C	19%
4	Pd_2dba_3•$CHCl_3$/PPh_3	80 °C	41%
5	Pd_2dba_3•$CHCl_3$/(2-Furyl)$_3$P	80 °C	32%
6	Pd_2dba_3•$CHCl_3$/BrettPhos	80 °C	13%
7	SPhos Pd G3/SPhos	80 °C	69%
8	SPhos Pd G4/SPhos	80 °C	68%
9	SPhos Pd G3/SPhos	100 °C	69%
10	SPhos Pd G3/SPhos	60 °C	68%

Reactions performed on 0.07 mmol scale for 16 h. [a] Yield determined by ^{1}H NMR using trichloroethylene as an internal standard. N.D. = not detected

Table S2. Optimization of reaction conditions.

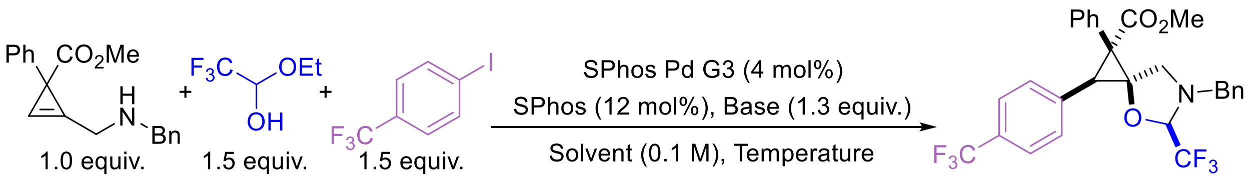

Entry	Base	Solvent (0.1 M)	Temperature	Yield[a]
1	Cs_2CO_3	DCE	80 °C	69%
2	Cs_2CO_3	CF_3Ph	80 °C	63%
3	Cs_2CO_3	EtOAc	80 °C	56%
4	Cs_2CO_3	CPME	80 °C	51%
5	Cs_2CO_3	MeCN	80 °C	36%
6	Cs_2CO_3	PhCl	80 °C	62%
7	K_3PO_4	DCE	80 °C	70%
8	K_2CO_3	DCE	80 °C	66%
9	KO^{t}Bu	DCE	80 °C	ND
10	K_3PO_4	DCE	50 °C	80%
11	K_3PO_4	DCE	23 °C	67%

Reactions performed on 0.07 mmol scale for 16 h. [a] Yield determined by ^{1}H NMR using trichloroethylene as an internal standard.

Procedures and Product Characterization of Cyclopropane Products

General procedure C: Synthesis of spirocyclopropanes.

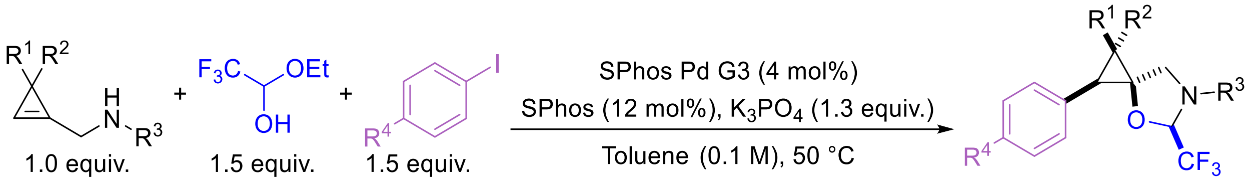

SPhos Pd G3 (7.7 mg, 0.080 mmol, 4 mol%), SPhos (13 mg, 0.024 mmol, 12 mol%) and K_3PO_4 (55 mg, 0.26 mmol, 1.3 equiv.) are weighed in an 8 mL microwave vial inside of a nitrogen filled glovebox, sealed and removed from the glovebox. The vial is then charged with aryl iodide (1.5 equiv.) and the cyclopropene starting material (0.20 mmol, 1.0 equiv.), sealed, and purged and backfilled with nitrogen three times. Anhydrous DCE (2 mL, 0.1 M), trifluoroacetaldehyde ethyl hemiacetal (90%, 39 µL, 0.30 mmol, 1.5 equiv.) and if liquid, the aryl iodide coupling partner (1.5 equiv.) are added and the reaction vial is placed into an aluminum reaction block that has been preheated to 50 °C and stirred for 16 h. The reaction mixture is then cooled to room temperature, and the crude mixture is filtered over a pad of silica gel, eluting with EtOAc, and concentrated *in vacuo*. The crude residue is then purified via flash column chromatography using the indicated solvents to afford the desired compounds.

Methyl 6-benzyl-1-phenyl-5-(trifluoromethyl)-2-(4-(trifluoromethyl)phenyl)-4-oxa-6-azaspiro[2.4]heptane-1-carboxylate (6a)

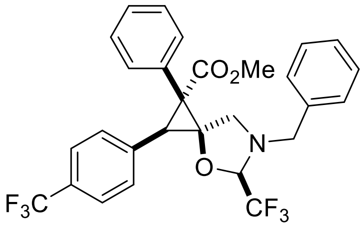

Following general procedure C, starting from **3a** (58 mg, 0.20 mmol, 1.0 equiv.), the crude product was purified by flash column chromatography (SiO_2, 12 g, 3 – 15% Et_2O in pentane) to afford **6a** as an off-white solid (77 mg, 0.14 mmol, 73%).

Melting Point: 124 – 125 °C.

TLC: R_f = 0.22 (SiO_2, 10% Et_2O in pentane).

^{1}H NMR (400 MHz, $CDCl_3$) δ 7.38 (d, *J* = 8.2 Hz, 2H, Ar*H*), 7.26 (s, 8H, Ar*H*), 7.06 – 6.97 (m, 4H, Ar*H*), 4.80 (q, *J* = 5.6 Hz, 1H, C*H*CF_3), 3.90 (d, *J* = 13.2 Hz, 1H, CH_2), 3.85 – 3.77 (m, 2H, CH_2), 3.62 (s, 3H, CO_2CH_3), 3.49 – 3.42 (m, 1H, CH_2), 3.25 (s, 1H, C*H*).

^{13}C NMR (101 MHz, $CDCl_3$) δ 172.4, 138.2, 137.0, 132.8, 130.5, 130.2, 128.8, 128.8, 128.7 (q, $J_{C\text{-}F}$ = 32.4 Hz), 128.1, 127.9, 127.9, 124.4 (q, *J* = 3.8 Hz), 124.40 (q, $J_{C\text{-}F}$ = 271.7 Hz), 123.1 (q, $J_{C\text{-}F}$ = 283.5 Hz), 93.6 (q, $J_{C\text{-}F}$ = 34.3 Hz), 73.5, 60.6, 57.5, 57.5, 53.3, 39.5.

^{19}F NMR (376 MHz, $CDCl_3$) δ -62.44, -79.40 (d, *J* = 5.6 Hz).

HRMS (nanochip-ESI/LTQ-Orbitrap) m/z: $[M + H]^+$ Calcd for $C_{28}H_{24}F_6NO_3^+$ 536.1655; Found 536.1654.

IR (ν_{max}, cm^{-1}) 3065 (w), 3034 (w), 2953 (w), 2849 (w), 1718 (m), 1619 (w), 1326 (s), 1256 (m), 1235 (m), 1169 (s), 1119 (s), 1070 (m), 1016 (m), 990 (m), 847 (m).

(+)-Methyl 6-benzyl-1-phenyl-5-(trifluoromethyl)-2-(4-(trifluoromethyl)phenyl)-4-oxa-6-azaspiro[2.4]heptane-1-carboxylate ((+)-6a)

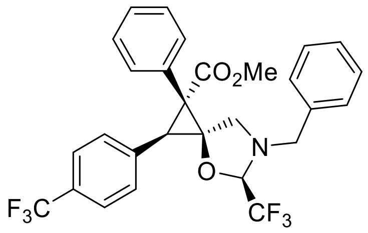

Following general procedure C, starting from **(+)-3a** (29 mg, 0.10 mmol, 1.0 equiv.), the crude product was purified by flash column chromatography (SiO_2, 12 g, 3 – 15% Et_2O in pentane) to afford **(+)-6a** as an off-white solid (33 mg, 0.062 mmol, 62%) with identical spectral properties to racemic **6a**. The enantiomeric ratio was determined to be 87:13 by HPLC analysis on a Daicel Chiralpak IB N-5 column: 99:1 hexane/IPA, flow rate 1 mL/min, λ= 254 nm: τ_{major} = 8.0 min, τ_{minor} = 8.9 min.

$[\alpha]_D^{20}$ = 166.8 (c = 1.17 in $CHCl_3$, 87:13 e.r.,)

Methyl 6-benzyl-1,2-diphenyl-5-(trifluoromethyl)-4-oxa-6-azaspiro[2.4]heptane-1-carboxylate (6b)

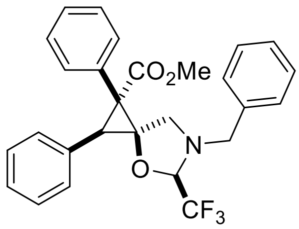

Following general procedure C, starting from **3a** (59 mg, 0.20 mmol, 1.0 equiv.), the crude product was purified by flash column chromatography (SiO_2, 12 g, 3 – 10% Et_2O in pentane, then SiO_2, 12 g 20 – 45% DCM in pentane) to afford **6b** as an off-white solid (51 mg, 0.11 mmol, 54%).

Melting Point: 137 – 139 °C.

TLC: R_f = 0.19 (SiO_2, 10% Et_2O in pentane).

^1H NMR (400 MHz, $CDCl_3$) δ 7.33 – 7.26 (m, 5H, Ar*H*), 7.25 – 7.11 (m, 6H, Ar*H*), 7.06 – 7.00 (m, 2H, Ar*H*), 7.00 – 6.94 (m, 2H, Ar*H*), 4.79 (q, *J* = 5.7 Hz, 1H, C*H*CF_3), 3.92 (d, *J* = 13.2 Hz, 1H, CH_2), 3.86 – 3.75 (m, 2H, CH_2), 3.61 (s, 3H, CO_2CH_3), 3.48 – 3.40 (m, 1H, CH_2), 3.23 (s, 1H, C*H*).

^{13}C NMR (101 MHz, $CDCl_3$) δ 172.8, 137.3, 134.0, 132.9, 130.8, 130.3, 128.8, 128.7, 127.9, 127.7, 127.6, 127.5, 126.7, 123.0 (q, $J_{C\text{-}F}$ = 283.4 Hz), 93.5 (q, $J_{C\text{-}F}$ = 34.2 Hz), 73.5, 60.6, 57.6, 53.1, 40.2, 38.9.

^{19}F NMR (376 MHz, $CDCl_3$) δ -79.42 (d, *J* = 5.6 Hz).

HRMS (ESI/QTOF) m/z: $[M + H]^+$ Calcd for $C_{27}H_{25}F_3NO_3^+$ 468.1781; Found 468.1783.

IR (v_{max}, cm^{-1}) 3089 (w), 3063 (w), 3032 (w), 2952 (w), 2846 (w), 1955 (w), 1881 (w), 1809 (w), 1715 (s), 1498 (m), 1293 (s), 1255 (s), 1233 (s), 1174 (s), 1147 (s), 1123 (s), 992 (s), 760 (s).

Methyl 6-benzyl-1-phenyl-2-(p-tolyl)-5-(trifluoromethyl)-4-oxa-6-azaspiro[2.4]heptane-1-carboxylate (6c)

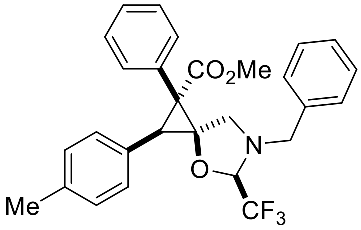

Following general procedure C, starting from **3a** (60 mg, 0.20 mmol, 1.0 equiv.), the crude product was purified by flash column chromatography (SiO_2, 12 g, 3 – 15% Et_2O in pentane) to afford **6c** as an off-white solid (44 mg, 0.091 mmol, 45%).

Melting Point: 137 – 138 °C.

TLC: R_f = 0.49 (SiO_2, 10% Et_2O in pentane).

^{1}H NMR (400 MHz, $CDCl_3$) δ 7.26 – 7.19 (m, 5H, Ar*H*), 7.18 – 7.10 (m, 3H, Ar*H*), 6.98 – 6.92 (m, 2H, Ar*H*), 6.88 (d, *J* = 8.1 Hz, 2H, Ar*H*), 6.77 (d, *J* = 8.2 Hz, 2H, Ar*H*), 4.69 (q, *J* = 5.7 Hz, 1H, C*H*CF_3), 3.83 (d, *J* = 13.2 Hz, 1H, CH_2), 3.75 (d, *J* = 13.2 Hz, 1H, CH_2), 3.69 (d, *J* = 13.2 Hz, 1H, CH_2), 3.53 (s, 3H, CO_2CH_3), 3.38 – 3.32 (m, 1H, CH_2), 3.12 (s, 1H, C*H*), 2.22 (s, 3H, ArCH_3).

^{13}C NMR (101 MHz, $CDCl_3$) δ 172.7, 137.2, 136.1, 132.8, 130.8, 130.6, 130.0, 128.7, 128.6, 128.3, 127.8, 127.5, 127.5, 127.4, 121.7 (q, $J_{C\text{-}F}$ = 283.4 Hz), 93.4 (q, $J_{C\text{-}F}$ = 34.3 Hz), 73.3, 60.5, 57.5, 52.9, 39.9, 38.6, 21.1.

^{19}F NMR (376 MHz, $CDCl_3$) δ -79.43 (d, *J* = 5.7 Hz, 1F).

HRMS (nanochip-ESI/LTQ-Orbitrap) m/z: $[M + H]^+$ Calcd for $C_{28}H_{27}F_3NO_3^+$ 482.1938; Found 482.1935.

IR (ν_{max}, cm^{-1}) 3060 (w), 3029 (w), 2954 (w), 2902 (w), 1715 (m), 1517 (w), 1497 (w), 1452 (w), 1436 (w), 1293 (m), 1255 (m), 1232 (s), 1177 (m), 1147 (m), 1123 (m), 996 (m), 771 (s).

Methyl 6-benzyl-2-(4-methoxyphenyl)-1-phenyl-5-(trifluoromethyl)-4-oxa-6-azaspiro[2.4]heptane-1-carboxylate (6d)

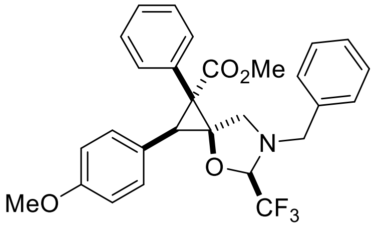

Following general procedure C, starting from **3a** (58 mg, 0.20 mmol, 1.0 equiv.), the crude product was purified by flash column chromatography (SiO_2, 12 g, 5 – 18% Et_2O in pentane) to afford **6d** as an off-white solid (31 mg, 0.061 mmol, 31%).

Melting Point: 107 – 108 °C.

TLC: R_f = 0.21 (SiO_2, 10% Et_2O in pentane).

^{1}H NMR (400 MHz, $CDCl_3$) δ 7.35 – 7.26 (m, 5H, Ar*H*), 7.26 – 7.18 (m, 3H, Ar*H*), 7.05 – 6.98 (m, 2H, Ar*H*), 6.92 – 6.85 (m, 2H, Ar*H*), 6.72 – 6.65 (m, 2H, Ar*H*), 4.77 (q, *J* = 5.6 Hz, 1H, C*H*CF_3), 3.95 – 3.80 (m, 2H, CH_2), 3.76 (s, 4H, CH2, ArOCH_3), 3.60 (s, 3H, CO_2CH_3), 3.49 – 3.38 (m, 1H, CH_2), 3.18 (s, 1H, C*H*).

^{13}C NMR (101 MHz, $CDCl_3$) δ 172.9, 158.4, 137.3, 133.0, 131.3, 130.9, 128.9, 128.7, 127.9, 127.6, 127.5, 125.9, 123.2 (q, J_{C-F} = 283.5 Hz), 113.2, 93.4 (q, J_{C-F} = 34.2 Hz), 73.4, 60.6, 57.7, 55.3, 53.0, 39.8, 38.5.

^{19}F NMR (376 MHz, $CDCl_3$) δ -79.4 (d, *J* = 5.5 Hz).

HRMS (APCI/QTOF) m/z: [M + Na]$^+$ Calcd for $C_{28}H_{26}F_3NNaO_4^+$ 520.1706; Found 520.1705.

IR (v_{max}, cm^{-1}) 3061 (w), 3028 (w), 2955 (w), 2903 (w), 2840 (w), 1714 (m), 1515 (m), 1296 (m), 1254 (s), 1235 (s), 1177 (s), 1147 (s), 1123 (m), 1034 (m), 994 (m), 770 (s).

Methyl 6-benzyl-2-(4-(*tert*-butyl)phenyl)-1-phenyl-5-(trifluoromethyl)-4-oxa-6-azaspiro[2.4]heptane-1-carboxylate (6e)

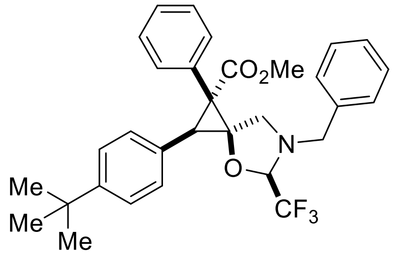

Following general procedure C, starting from **3a** (57 mg, 0.19 mmol, 1.0 equiv.), the crude product was purified by flash column chromatography (SiO_2, 12 g, 3 – 15% Et_2O in pentane) to afford **6e** as a white solid (20 mg, 0.038 mmol, 20%).

Melting Point: 135 – 137 °C.

TLC: R_f = 0.32 (SiO_2, 10% Et_2O in pentane).

^{1}H NMR (400 MHz, $CDCl_3$) δ 7.33 – 7.27 (m, 5H, Ar*H*), 7.25 – 7.17 (m, 3H, Ar*H*), 7.18 – 7.13 (m, 2H, Ar*H*), 7.06 – 7.00 (m, 2H, Ar*H*), 6.93 – 6.86 (m, 2H, Ar*H*), 4.78 (q, *J* = 5.6 Hz, 1H, C*H*F_3), 3.93 (d, *J* = 13.2 Hz, 1H, CH_2), 3.84 (d, *J* = 13.2 Hz, 1H , CH_2), 3.81 – 3.73 (m, 1H, CH_2), 3.61 (s, 3H, CO_2CH_3), 3.47 – 3.41 (m, 1H, CH_2), 3.21 (s, 1H, C*H*), 1.29 (s, 9H, $C(CH_3)_3$).

^{13}C NMR (101 MHz, $CDCl_3$) δ 172.9, 149.4, 137.4, 133.0, 130.9, 130.7, 129.9, 128.9, 128.7, 127.9, 127.6, 127.4, 124.5, 123.2 (q, *J* = 283.5 Hz), 93.4 (q, *J* = 34.1 Hz), 73.4, 60.5, 57.6, 53.0, 39.9, 38.7, 34.5, 31.4.

^{19}F NMR (376 MHz, $CDCl_3$) δ -79.4 (d, *J* = 5.6 Hz).

HRMS (ESI/QTOF) m/z: [M + H]$^+$ Calcd for $C_{31}H_{33}F_3NO_3^+$ 524.2407; Found 524.2421.

IR (v_{max}, cm^{-1}) 2970 (s), 2901 (s), 1713 (m), 1406 (m), 1254 (m), 1217 (s), 1061 (s), 912 (m)

Methyl 6-benzyl-2-(4-fluorophenyl)-1-phenyl-5-(trifluoromethyl)-4-oxa-6-azaspiro[2.4]heptane-1-carboxylate (6f)

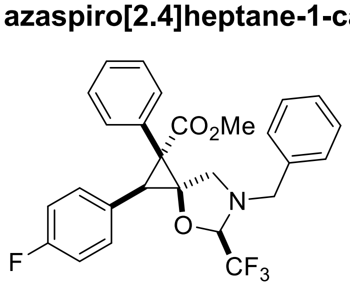

Following general procedure C, starting from **3a** (59 mg, 0.20 mmol, 1.0 equiv.), the crude product was purified by flash column chromatography (SiO_2, 12 g, 5 – 15% Et_2O in pentane) to afford **6f** as an off-white solid (69 mg, 0.14 mmol, 71%).

Melting Point: 126 – 127 °C.

TLC: R_f = 0.28 (SiO_2, 10% Et_2O in pentane).

^{1}H NMR (400 MHz, $CDCl_3$) δ 7.37 – 7.26 (m, 5H, Ar*H*), 7.26 – 7.17 (m, 3H, Ar*H*), 7.04 – 6.98 (m, 2H, Ar*H*), 6.96 – 6.89 (m, 2H, Ar*H*), 6.87 – 6.79 (m, 2H, Ar*H*), 4.79 (q, *J* = 5.7 Hz, 1H, C*H*CF_3), 3.92 (d, *J* = 13.2 Hz, 1H, CH_2), 3.85 – 3.76 (m, 2H, CH_2), 3.61 (s, 3H, CO_2CH_3), 3.49 – 3.42 (m, 1H, CH_2), 3.21 (s, 1H, C*H*).

^{13}C NMR (101 MHz, $CDCl_3$) δ 172.7, 161.8 (d, $J_{C\text{-}F}$ = 245.6 Hz), 137.1, 132.9, 131.7 (d, $J_{C\text{-}F}$ = 7.7 Hz), 130.5, 129.7 (d, $J_{C\text{-}F}$ = 3.3 Hz), 128.8, 128.7, 128.0, 127.7, 127.7, 123.2 (q, $J_{C\text{-}F}$ = 283.2 Hz), 114.6 (d, $J_{C\text{-}F}$ = 21.1 Hz), 93.5 (q, $J_{C\text{-}F}$ = 34.2 Hz), 73.3, 60.6, 57.6, 53.1, 39.3, 38.8.

^{19}F NMR (376 MHz, $CDCl_3$) δ -79.4 (d, *J* = 5.6 Hz), -115.9 (tt, *J* = 8.9, 5.6 Hz).

HRMS (ESI/QTOF) m/z: $[M + Na]^+$ Calcd for $C_{27}H_{23}F_4NNaO_3^+$ 508.1506; Found 508.1504.

IR (v_{max}, cm^{-1}) 3087 (w), 3064 (w), 3032 (m), 2952 (w), 2845 (w), 1955 (w), 1890 (w), 1715 (s), 1511 (s), 1293 (s), 1256 (s), 1224 (s), 1173 (s), 1124 (s), 993 (s), 757 (s).

Methyl 6-benzyl-2-(4-chlorophenyl)-1-phenyl-5-(trifluoromethyl)-4-oxa-6-azaspiro[2.4]heptane-1-carboxylate (6g)

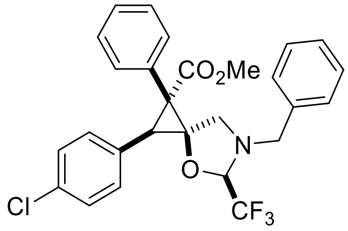

Following general procedure C, starting from **3a** (58 mg, 0.20 mmol, 1.0 equiv.), the crude product was purified by flash column chromatography (SiO_2, 12 g, 3 – 15% Et_2O in pentane), afford **6g** as an off-white solid (72 mg, 0.14 mmol, 72%).

Melting Point: 131 – 133 °C.

TLC: R_f = 0.34 (SiO_2, 10% Et_2O in pentane).

^{1}H NMR (400 MHz, $CDCl_3$) δ 7.33 – 7.26 (m, 4H, Ar*H*), 7.26 – 7.19 (m, 4H, Ar*H*), 7.13 – 7.08 (m, 2H, Ar*H*), 7.03 – 6.98 (m, 2H, Ar*H*), 6.90 – 6.85 (m, 2H, Ar*H*), 4.78 (q, *J* = 5.7 Hz, 1H, C*H*CF_3), 3.90 (d, *J* = 13.2 Hz, 1H, CH_2), 3.85 – 3.75 (m, 2H, CH_2), 3.61 (s, 3H, CO_2CH_3), 3.48 – 3.40 (m, 1H, CH_2), 3.18 (s, 1H, C*H*).

^{13}C NMR (101 MHz, $CDCl_3$) δ 172.6, 137.1, 132.9, 132.6, 132.5, 131.5, 130.4, 128.8, 128.8, 128.0, 127.8, 127.8, 127.7, 123.2 (q, $J_{C\text{-}F}$ = 283.8 Hz), 93.5 (q, $J_{C\text{-}F}$ = 34.4 Hz), 73.3, 60.6, 57.6, 53.2, 39.4, 39.0.

^{19}F NMR (376 MHz, $CDCl_3$) δ -79.39 (d, *J* = 5.6 Hz).

HRMS (nanochip-ESI/LTQ-Orbitrap) m/z: $[M + H]^+$ Calcd for $C_{27}H_{24}ClF_3NO_3^+$ 502.1391; Found 502.1389.

IR (v_{max}, cm^{-1}) 3088 (w), 3063 (w), 3030 (w), 2954 (w), 2900 (w), 1962 (w), 1904 (w), 1715 (s), 1495 (m), 1292 (s), 1254 (s), 1233 (s), 1174 (s), 1147 (s), 1123 (s), 992 (s), 764 (s).

Methyl 6-benzyl-1-phenyl-2-(4-(trifluoromethoxy)phenyl)-5-(trifluoromethyl)-4-oxa-6-azaspiro[2.4]heptane-1-carboxylate (6h)

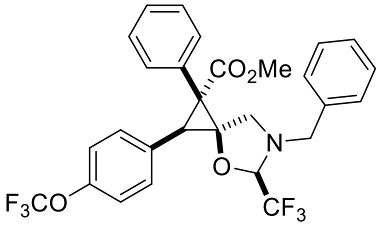

Following general procedure C, starting from **3a** (60 mg, 0.20 mmol, 1.0 equiv.), the crude product was purified by flash column chromatography (SiO_2, 12 g, 5 – 10% Et_2O in pentane, then SiO_2, 12 g, 25-50% DCM in pentane) to afford **6h** as an off-white solid (69 mg, 0.13 mmol, 62%).

Melting Point: 144 – 146 °C.

TLC: R_f = 0.22 (SiO_2, 10% Et_2O in pentane).

^{1}H NMR (400 MHz, $CDCl_3$) δ 7.31 – 7.26 (m, 4H, Ar*H*), 7.26 – 7.18 (m, 4H, Ar*H*), 7.03 – 6.92 (m, 6H, Ar*H*), 4.81 (q, *J* = 5.7 Hz, 1H, C*H*CF$_3$), 3.92 (d, *J* = 13.3 Hz, 1H, C*H*$_2$), 3.86 – 3.76 (m, 2H, C*H*$_2$, C*H*$_2$), 3.62 (s, 3H, CO$_2$C*H*$_3$), 3.49 – 3.41 (m, 1H, C*H*$_2$), 3.21 (s, 1H, C*H*).

^{13}C NMR (101 MHz, $CDCl_3$) δ 172.4, 147.9 (q, J_{C-F} = 1.9 Hz), 136.9, 132.7, 132.6, 131.4, 130.2, 128.7, 128.6, 127.9, 127.7, 127.6, 125.9 (q, J_{C-F} = 283.3 Hz), 120.5 (q, J_{C-F} = 257.0 Hz), 119.9, 93.4 (q, J_{C-F} = 34.2 Hz), 73.3, 60.4, 57.4, 53.0, 39.1, 39.0.

^{19}F NMR (376 MHz, $CDCl_3$) δ -57.82, -79.42 (d, *J* = 5.9 Hz).

HRMS (ESI/QTOF) m/z: [M + Na]$^+$ Calcd for $C_{28}H_{23}F_6NNaO_4^+$ 574.1423; Found 574.1437.

IR (ν_{max}, cm^{-1}) 3060 (w), 3033 (w), 2952 (w), 2843 (w), 1717 (m), 1510 (m), 1292 (m), 1255 (s), 1223 (s), 1167 (s), 1147 (s), 992 (m), 758 (s).

Methyl 6-benzyl-2-(4-cyanophenyl)-1-phenyl-5-(trifluoromethyl)-4-oxa-6-azaspiro[2.4]heptane-1-carboxylate (6i)

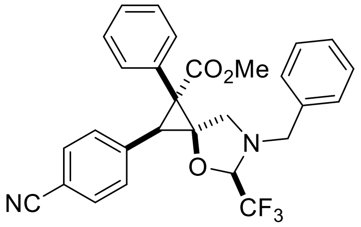

Following general procedure C, starting from **3a** (60 mg, 0.20 mmol, 1.0 equiv.), the crude product was purified by flash column chromatography (SiO_2, 12 g, 2 – 15% Et_2O in pentane) to afford **6i** as an off-white solid (70 mg, 0.14 mmol, 70%).

Melting Point: 128 – 129 °C.

TLC: R_f = 0.24 (SiO_2, 10% EtOAc in pentane).

^{1}H NMR (400 MHz, $CDCl_3$) δ 7.44 – 7.37 (m, 2H, Ar*H*), 7.32 – 7.26 (m, 3H, Ar*H*), 7.26 – 7.19 (m, 5H, Ar*H*), 7.04 – 6.94 (m, 4H, Ar*H*), 4.81 (q, *J* = 5.6 Hz, 1H, C*H*CF$_3$), 3.90 (d, *J* = 13.3 Hz, 1H, C*H*$_2$), 3.83 – 3.77 (m, 2H, C*H*$_2$), 3.62 (s, 3H, CO$_2$C*H*$_3$), 3.50 – 3.41 (m, 1H, C*H*$_2$), 3.22 (s, 1H, C*H*).

^{13}C NMR (101 MHz, $CDCl_3$) δ 172.1, 139.9, 136.8, 132.7, 131.3, 130.8, 129.9, 128.8, 128.2, 128.0, 128.0, 127.3, 123.2 (q, $J_{C\text{-}F}$ = 285.2 Hz), 119.1, 110.3, 93.7 (q, $J_{C\text{-}F}$ = 34.3 Hz), 73.7, 60.6, 57.5, 53.3, 40.0, 39.6.

^{19}F NMR (376 MHz, $CDCl_3$) δ -79.36 (d, J = 5.5 Hz).

HRMS (nanochip-ESI/LTQ-Orbitrap) m/z: $[M + H]^+$ Calcd for $C_{28}H_{24}F_3N_2O_3^+$ 493.1734; Found 493.1730.

IR (ν_{max}, cm^{-1}) 3087 (w), 3063 (w), 3029 (w), 2954 (w), 2847 (w), 2228 (m), 1719 (s), 1293 (s), 1256 (s), 1175 (s), 1148 (s), 1124 (s), 992 (m), 757 (s).

Methyl 6-benzyl-2-(4-nitrophenyl)-1-phenyl-5-(trifluoromethyl)-4-oxa-6-azaspiro[2.4]heptane-1-carboxylate (6j)

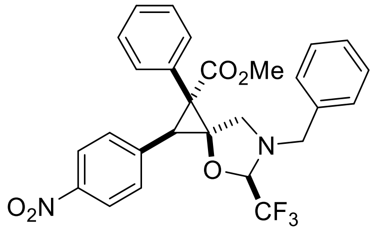

Following general procedure C, starting from **3a** (67 mg, 0.23 mmol, 1.0 equiv.), the crude product was purified by flash column chromatography (SiO_2, 12 g, 10 – 35% Et_2O in pentane) to afford **6j** as an off-white solid (67 mg, 0.13 mmol, 57%).

Melting Point: 172 – 174 °C.

TLC: R_f = 0.29 (SiO_2, 30% Et_2O in pentane).

^{1}H NMR (400 MHz, $CDCl_3$) δ 8.04 – 7.90 (m, 2H, Ar*H*), 7.31 – 7.26 (m, 3H, Ar*H*), 7.26 – 7.19 (m, 5H, Ar*H*), 7.10 – 7.04 (m, 2H, Ar*H*), 7.02 – 6.95 (m, 2H, Ar*H*), 4.82 (q, J = 5.6 Hz, 1H, C*H*CF_3), 3.90 (d, J = 13.2 Hz, 1H, CH_2), 3.86 – 3.76 (m, 2H, CH_2), 3.63 (s, 3H, CO_2CH_3), 3.53 – 3.43 (m, 1H, CH_2), 3.29 (s, 1H, C*H*).

^{13}C NMR (101 MHz, $CDCl_3$) δ 172.1, 146.6, 142.1, 136.8, 132.6, 130.9, 129.8, 128.8 (2C), 128.2, 128.1, 128.1, 123.1 (d, $J_{C\text{-}F}$ = 283.8 Hz), 122.7, 93.7 (q, $J_{C\text{-}F}$ = 34.4 Hz), 73.8, 60.6, 57.5, 53.4, 40.3, 39.4.

^{19}F NMR (376 MHz, $CDCl_3$) δ -79.34 (d, J = 5.6 Hz).

HRMS (ESI/QTOF) m/z: $[M + H]^+$ Calcd for $C_{27}H_{24}F_3N_2O_5^+$ 513.1632; Found 513.1646.

IR (ν_{max}, cm^{-1}) 3065 (w), 3028 (w), 2953 (w), 2849 (w), 1718 (m), 1600 (w), 1520 (m), 1345 (m), 1293 (m), 1257 (m), 1221 (m), 1176 (m), 991 (m), 858 (m), 769 (s).

Methyl 2-(4-acetoxyphenyl)-6-benzyl-1-phenyl-5-(trifluoromethyl)-4-oxa-6-azaspiro[2.4]heptane-1-carboxylate (6k)

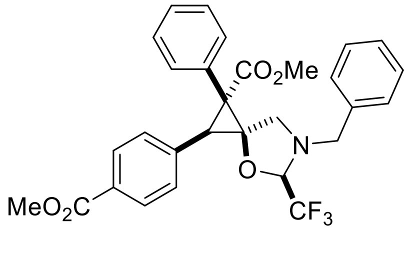

Following general procedure C, starting from **3a** (59 mg, 0.20 mmol, 1.0 equiv.), the crude product was purified by flash column chromatography (SiO_2, 12 g, 5 – 15% Et_2O in pentane) to afford **6k** as an off-white solid (78 mg, 0.15 mmol, 74%).

Melting Point: 130 – 131 °C.

TLC: R_f = 0.25 (SiO_2, 10% EtOAc in pentane).

^{1}H NMR (400 MHz, $CDCl_3$) δ 7.84 – 7.76 (m, 2H, Ar*H*), 7.27 (d, *J* = 5.3 Hz, 2H, Ar*H*), 7.26 – 7.17 (m, 6H, Ar*H*), 7.06 – 6.95 (m, 4H, Ar*H*), 4.79 (q, *J* = 5.6 Hz, 1H, C*H*CF_3), 3.93 – 3.86 (m, 4H, CH_2, CO_2CH_3), 3.80 (d, *J* = 13.2 Hz, 2H, CH_2), 3.62 (s, 3H, CO_2CH_3), 3.48 (d, *J* = 1.5 Hz, 1H, CH_2), 3.25 (s, 1H, C*H*).

^{13}C NMR (101 MHz, $CDCl_3$) δ 172.4, 167.2, 139.6, 137.0, 132.8, 130.3, 130.2, 128.8, 128.8, 128.8, 128.3, 128.1, 127.8, 127.8, 123.2 (q, $J_{C\text{-}F}$ = 283.8 Hz), 93.6 (q, $J_{C\text{-}F}$ = 34.1 Hz), 73.7, 60.6, 57.6, 53.2, 52.2, 39.9, 39.7.

^{19}F NMR (376 MHz, $CDCl_3$) δ -79.39 (d, *J* = 5.7 Hz).

HRMS (nanochip-ESI/LTQ-Orbitrap) m/z: $[M + H]^+$ Calcd for $C_{29}H_{27}F_3NO_5^+$ 526.1836; Found 526.1830.

IR (ν_{max}, cm^{-1}) 3029 (m), 2954 (m), 2847 (w), 1717 (s), 1610 (m), 1436 (m), 1282 (s), 1175 (s), 1119 (s), 993 (s), 756 (s).

Methyl 6-benzyl-2-(4-formylphenyl)-1-phenyl-5-(trifluoromethyl)-4-oxa-6-azaspiro[2.4]heptane-1-carboxylate (6l)

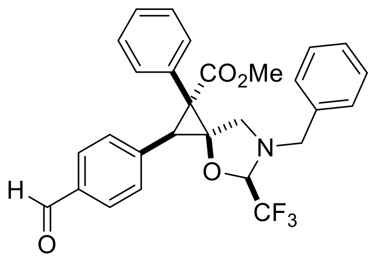

Following general procedure C, starting from **3a** (59 mg, 0.20 mmol, 1.0 equiv.), the crude product was purified by flash column chromatography (SiO_2, 12 g, 5 – 15% Et_2O in pentane) to afford **6l** as an off-white solid (53 mg, 0.11 mmol, 53%).

Melting Point: 133 – 134 °C.

TLC: R_f = 0.27 (SiO_2, 10% Et_2O in pentane).

^{1}H NMR (400 MHz, $CDCl_3$) δ 9.94 (s, 1H, CO*H*), 7.72 – 7.56 (m, 2H, Ar*H*), 7.32 – 7.26 (m, 3H, Ar*H*), 7.26 – 7.18 (m, 5H, Ar*H*), 7.13 – 7.06 (m, 2H, Ar*H*), 7.03 – 6.98 (m, 2H, Ar*H*), 4.81 (q, *J* = 5.6 Hz, 1H, C*H*CF_3), 3.95 – 3.77 (m, 3H, CH_2, CH_2), 3.63 (s, 3H, CO_2CH_3), 3.53 – 3.41 (m, 1H, CH_2), 3.28 (s, 1H, C*H*).

^{13}C NMR (101 MHz, $CDCl_3$) δ 192.2, 172.3, 141.5, 136.9, 134.7, 132.7, 130.8, 130.2, 128.9, 128.8, 128.8, 128.1, 127.9, 127.9, 123.1 (q, $J_{C\text{-}F}$ = 283.6 Hz), 93.6 (q, $J_{C\text{-}F}$ = 34.3 Hz), 73.8, 60.6, 57.6, 53.3, 40.0, 40.0.

^{19}F NMR (376 MHz, $CDCl_3$) δ -79.36 (d, *J* = 5.6 Hz).

HRMS (ESI/QTOF) m/z: $[M + Na]^+$ Calcd for $C_{28}H_{24}F_3NNaO_4^+$ 518.1550; Found 518.1553.

IR (ν_{max}, cm^{-1}) 3046 (w), 2984 (s), 2903 (s), 1713 (m), 1701 (s), 1606 (m), 1391 (m), 1232 (s), 1057 (s), 760 (m).

Methyl 2-(4-acetylphenyl)-6-benzyl-1-phenyl-5-(trifluoromethyl)-4-oxa-6-azaspiro[2.4]heptane-1-carboxylate (6m)

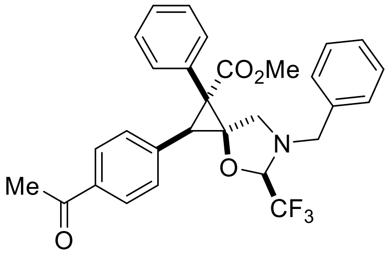

Following general procedure C, starting from **3a** (61 mg, 0.21 mmol, 1.0 equiv.), the crude product was purified by flash column chromatography (SiO_2, 12 g, 5 – 15% Et_2O in pentane) to afford **6m** as an off-white solid (62 mg, 0.12 mmol, 58%).

Melting Point: 143 – 144 °C.

TLC: R_f = 0.37 (SiO_2, 10% Et_2O in pentane).

^{1}H NMR (400 MHz, $CDCl_3$) δ 7.77 – 7.69 (m, 2H, Ar*H*), 7.31 – 7.18 (m, 8H, Ar*H*), 7.06 – 6.97 (m, 4H, Ar*H*), 4.79 (q, *J* = 5.6 Hz, 1H, C*H*CF_3), 3.90 (d, *J* = 13.2 Hz, 1H, CH_2), 3.80 (d, *J* = 13.2 Hz, 2H, C*H*$_2$, C*H*$_2$), 3.62 (s, 3H, CO_2C*H*$_3$), 3.46 (dt, *J* = 13.3, 1.6 Hz, 1H, C*H*$_2$), 3.27 (s, 1H, C*H*), 2.55 (s, 3H, COC*H*$_3$).

^{13}C NMR (101 MHz, $CDCl_3$) δ 198.1, 172.4, 139.8, 137.0, 135.4, 132.7, 130.4, 130.3, 128.8, 128.8, 128.1, 127.9, 127.8, 127.6, 123.1 (q, $J_{C\text{-}F}$ = 283.1 Hz), 93.6 (q, $J_{C\text{-}F}$ = 34.3 Hz), 73.7, 60.6, 57.6, 53.3, 39.9, 39.8, 26.7.

^{19}F NMR (376 MHz, $CDCl_3$) δ -79.39 (d, *J* = 5.5 Hz).

HRMS (nanochip-ESI/LTQ-Orbitrap) m/z: $[M + H]^+$ Calcd for $C_{29}H_{27}F_3NO_4^+$ 510.1887; Found 510.1880.

IR (ν_{max}, cm^{-1}) 3064 (w), 3028 (w), 2954 (w), 2849 (w), 1717 (m), 1681 (m), 1606 (m), 1255 (s), 1232 (s), 1174 (s), 1147 (s), 1123 (m), 992 (m), 844 (w), 754 (s).

Methyl 6-benzyl-2-(3-methoxyphenyl)-1-phenyl-5-(trifluoromethyl)-4-oxa-6-azaspiro[2.4]heptane-1-carboxylate (6n)

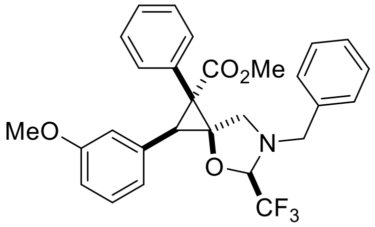

Following general procedure C, starting from **3a** (61 mg, 0.21 mmol, 1.0 equiv.), the crude product was purified by flash column chromatography (SiO_2, 12 g, 5 – 15% Et_2O in pentane), afford **6n** as an off-white solid (58 mg, 0.12 mmol, 56%).

Melting Point: 110 – 112 °C.

TLC: R_f = 0.26 (SiO_2, 10% Et_2O in pentane).

^{1}H NMR (400 MHz, $CDCl_3$) δ 7.37 – 7.27 (m, 5H, Ar*H*), 7.25 – 7.18 (m, 3H, Ar*H*), 7.11 – 7.02 (m, 3H, Ar*H*), 6.76 – 6.64 (m, 2H, Ar*H*), 6.38 (dd, *J* = 2.6, 1.6 Hz, 1H, Ar*H*), 4.78 (q, *J* = 5.6 Hz, 1H, C*H*CF_3), 3.92 (d, *J* = 13.2 Hz, 1H, CH_2), 3.84 (d, *J* = 13.2 Hz, 1H, CH_2), 3.78 (dd, *J* = 13.2, 1.4 Hz, 1H, CH_2), 3.61 (s, 3H, CO_2CH_3), 3.50 (s, 3H, ArOCH_3), 3.47 – 3.40 (m, 1H, CH_2), 3.20 (s, 1H, C*H*).

^{13}C NMR (101 MHz, $CDCl_3$) δ 172.7, 158.8, 137.3, 135.3, 132.9, 130.8, 128.8, 128.7, 128.5, 127.9, 127.7, 127.5, 123.2 (q, $J_{C\text{-}F}$ = 283.0 Hz), 123.0, 115.3, 113.0, 93.5 (q, $J_{C\text{-}F}$ = 34.1 Hz), 73.4, 60.6, 57.6, 55.0, 53.1, 40.1, 39.0.

^{19}F NMR (376 MHz, $CDCl_3$) δ -79.54 (d, *J* = 5.4 Hz).

HRMS (nanochip-ESI/LTQ-Orbitrap) m/z: $[M + H]^+$ Calcd for $C_{28}H_{27}F_3NO_4^+$ 498.1887; Found 498.1890.

IR (v_{max}, cm^{-1}) 3088 (w), 3061 (w), 3029 (w), 3000 (w), 2956 (w), 2898 (w), 2840 (w), 1716 (s), 1600 (m), 1494 (m), 1454 (m), 1292 (s), 1232 (s), 1173 (s), 1148 (s), 1123 (s), 734 (s).

Methyl 6-benzyl-1-phenyl-2-(o-tolyl)-5-(trifluoromethyl)-4-oxa-6-azaspiro[2.4]heptane-1-carboxylate (6o)

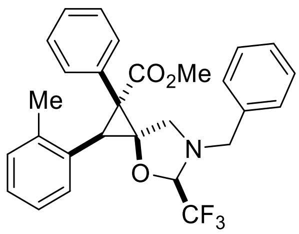

Following general procedure C, starting from **3a** (60 mg, 0.20 mmol, 1.0 equiv.), the crude product was purified by flash column chromatography (SiO_2, 12 g, 4 – 15% Et_2O in pentane) to afford **6o** as a colourless amorphous foam (26 mg, 0.053 mmol, 26%).

TLC: R_f = 0.38 (SiO_2, 10% Et_2O in pentane).

^{1}H NMR (400 MHz, $CDCl_3$) δ 7.36 – 7.27 (m, 5H, Ar*H*), 7.25 – 7.20 (m, 1H, Ar*H*), 7.20 – 7.07 (m, 4H, Ar*H*), 7.05 – 7.01 (m, 1H, Ar*H*), 6.96 – 6.85 (m, 3H, Ar*H*), 4.93 (q, *J* = 5.7 Hz, 1H, C*H*CF_3), 4.02 (d, *J* = 13.2 Hz, 1H, CH_2), 3.96 (d, *J* = 13.2 Hz, 1H, CH_2), 3.88 – 3.78 (m, 1H, CH_2), 3.64 (s, 3H, CO_2CH_3), 3.50 – 3.39 (m, 1H, CH_2), 3.25 (s, 1H, C*H*), 2.50 (s, 3H, ArCH_3).

^{13}C NMR (101 MHz, $CDCl_3$) δ 172.9, 138.1, 137.3, 133.4, 132.4, 131.4, 129.9, 128.9, 128.8, 128.3, 128.0, 127.6, 127.4, 126.8, 125.8, 123.2 (q, $J_{C\text{-}F}$ = 283.7 Hz), 93.5 (q, *J* = 34.1 Hz), 74.4, 60.6, 58.4, 53.1, 38.6, 36.0, 20.8.

^{19}F NMR (376 MHz, $CDCl_3$) δ -79.2 (d, *J* = 5.7 Hz).

HRMS (ESI/QTOF) m/z: $[M + H]^+$ Calcd for $C_{28}H_{27}F_3NO_3^+$ 482.1938; Found 482.1947.

IR (v_{max}, cm^{-1}) 2973 (s), 2904 (m), 1714 (s), 1496 (w), 1452 (m), 1408 (m), 1292 (s), 1236 (s), 1174 (s), 1146 (s), 1061 (s).

Methyl 6-benzyl-2-(2-fluorophenyl)-1-phenyl-5-(trifluoromethyl)-4-oxa-6-azaspiro[2.4]heptane-1-carboxylate (6p)

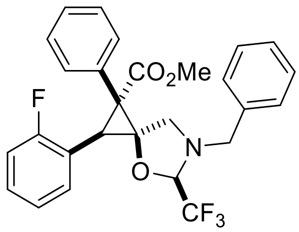

Following general procedure C, starting from **3a** (58 mg, 0.20 mmol, 1.0 equiv.), the crude product was purified by flash column chromatography (SiO_2, 12 g, 4 – 15% Et_2O in pentane) to afford **6p** as a white solid (59 mg, 0.12 mmol, 61%).

Melting Point: 159 – 161 °C.

TLC: R_f = 0.44 (SiO_2, 10% Et_2O in pentane).

^{1}H NMR (400 MHz, $CDCl_3$) δ 7.29 – 7.25 (m, 4H, Ar*H*), 7.25 – 7.14 (m, 4H, Ar*H*), 7.12 – 7.00 (m, 4H, Ar*H*), 6.72 (td, *J* = 7.5, 1.5 Hz, 1H, Ar*H*), 6.62 (td, *J* = 7.8, 1.7 Hz, 1H, Ar*H*), 4.82 (q, *J* = 5.6 Hz, 1H, C*H*CF_3), 3.93 (d, *J* = 13.2 Hz, 1H, C*H*$_2$), 3.84 (d, *J* = 13.2 Hz, 1H, C*H*$_2$), 3.82 – 3.74 (m, 1H, C*H*$_2$), 3.59 (s, 3H, CO_2CH_3), 3.51 (s, 1H, C*H*), 3.50 – 3.43 (m, 1H, C*H*$_2$).

^{13}C NMR (101 MHz, $CDCl_3$) δ 172.4, 162.3 (d, $J_{C\text{-}F}$ = 245.7 Hz), 137.1, 132.6, 130.8, 130.6 (d, $J_{C\text{-}F}$ = 2.4 Hz), 128.9, 128.7, 128.0, 127.9, 127.7, 127.6, 123.2 (d, $J_{C\text{-}F}$ = 3.6 Hz), 123.2 (q, $J_{C\text{-}F}$ = 283.7 Hz), 121.6 (d, $J_{C\text{-}F}$ = 12.1 Hz), 114.6 (d, $J_{C\text{-}F}$ = 22.6 Hz), 93.6 (q, $J_{C\text{-}F}$ = 34.2 Hz), 73.6, 60.6, 57.7, 53.1, 38.6, 31.3 (d, $J_{C\text{-}F}$ = 7.2 Hz).

^{19}F NMR (376 MHz, $CDCl_3$) δ -79.3 (d, *J* = 5.6 Hz), -117.2 – -117.9 (m).

HRMS (Sicrit plasma/LTQ-Orbitrap) m/z: $[M + H]^+$ Calcd for $C_{27}H_{24}F_4NO_3^+$ 486.1687; Found 486.1688.

IR (ν_{max}, cm^{-1}) 2975 (s), 2901 (m), 1717 (m), 1496 (m), 1453 (m), 1293 (m), 1237 (s), 1176 (s), 1147 (s), 1122 (s), 1058 (s), 912 (m).

Methyl 6-benzyl-1-phenyl-5-(trifluoromethyl)-2-(2-(trifluoromethyl)phenyl)-4-oxa-6-azaspiro[2.4]heptane-1-carboxylate (6q)

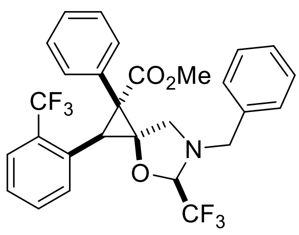

Following general procedure C, starting from **3a** (59 mg, 0.20, 1.0 equiv.), the crude product was purified by flash column chromatography (SiO_2, 12 g, 5 – 10% Et_2O in pentane, then SiO_2, 12 g, 25 – 50% DCM in pentane) to afford **6q** as an off-white solid (29 mg, 0.054 mmol, 27%).

Melting Point: 138 – 139 °C.

TLC: R_f = 0.43 (SiO_2, 10% DCM in pentane).

^{1}H NMR (400 MHz, $CDCl_3$) δ 7.70 (dd, *J* = 7.9, 1.4 Hz, 1H, Ar*H*), 7.31 – 7.27 (m, 4H, Ar*H*), 7.26 – 7.08 (m, 6H, Ar*H*), 7.00 – 6.92 (m, 3H, Ar*H*), 4.89 (q, *J* = 5.7 Hz, 1H, C*H*CF_3), 3.97 – 3.84 (m, 2H, C*H*$_2$), 3.84 – 3.78 (m, 1H, C*H*$_2$), 3.62 (s, 3H, CO_2CH_3), 3.54 (s, 1H, C*H*), 3.53 – 3.47 (m, 1H. C*H*$_2$).

^{13}C NMR (101 MHz, $CDCl_3$) δ 172.0, 137.1, 133.3, 132.6, 131.0, 130.7, 130.4 (q, $J_{C\text{-}F}$ = 29.2 Hz), 130.4, 128.9, 128.8, 128.0, 127.7, 127.7, 126.4, 125.7 (q, $J_{C\text{-}F}$ = 5.9 Hz), 124.8 (q, $J_{C\text{-}F}$ =

273.9 Hz), 123.2 (q, $J_{C\text{-}F}$ = 283.7 Hz), 93.8 (q, $J_{C\text{-}F}$ = 34.4 Hz), 74.2, 60.6, 58.1, 53.2, 39.7, 34.9 (q, $J_{C\text{-}F}$ = 2.2 Hz).

^{19}F NMR (376 MHz, $CDCl_3$) δ -59.4, -79.4 (d, J = 5.4 Hz).

HRMS (APCI/QTOF) m/z: [M + H]$^+$ Calcd for $C_{28}H_{24}F_6NO_3^+$ 536.1655; Found 536.1648.

IR (ν_{max}, cm^{-1}) 3068 (w), 3031 (w), 2956 (w), 2902 (w), 2848 (w), 1721 (m), 1313 (s), 1294 (s), 1236 (s), 1159 (s), 1120 (s), 1062 (m), 1036 (m), 991 (m), 770 (s).

Methyl 6-benzyl-2-(4-fluoro-3-(methoxycarbonyl)phenyl)-1-phenyl-5-(trifluoromethyl)-4-oxa-6-azaspiro[2.4]heptane-1-carboxylate (6r)

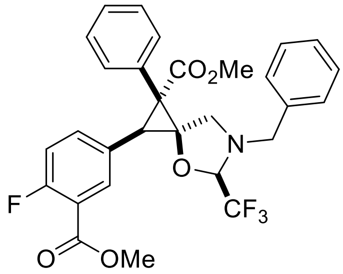

Following general procedure C, starting from **3a** (57 mg, 0.19 mmol, 1.0 equiv.), the crude product was purified by flash column chromatography (SiO_2, 12 g, 3 – 15% Et_2O in pentane), afford **6r** as an off-white solid (62 mg, 0.11 mmol, 59%).

Melting Point: 54 – 55 °C.

TLC: R_f = 0.10 (SiO_2, 10% Et_2O in pentane).

^{1}H NMR (400 MHz, $CDCl_3$) δ 7.79 – 7.71 (m, 1H, Ar*H*), 7.32 – 7.26 (m, 4H, Ar*H*), 7.26 – 7.18 (m, 4H, Ar*H*), 7.02 – 6.94 (m, 2H, Ar*H*), 6.82 – 6.76 (m, 2H, Ar*H*), 4.81 (q, J = 5.7 Hz, 1H, C*H*CF_3), 3.95 – 3.88 (m, 4H, CH_2, CO_2CH_3), 3.86 – 3.77 (m, 2H, C*H*$_2$), 3.62 (s, 3H, CO_2CH_3), 3.51 – 3.40 (m, 1H, C*H*$_2$), 3.23 (s, 1H, C*H*).

^{13}C NMR (101 MHz, $CDCl_3$) δ 172.4, 165.0 (d, $J_{C\text{-}F}$ = 3.8 Hz), 160.8 (d, $J_{C\text{-}F}$ = 259.8 Hz), 137.0, 135.4 (d, $J_{C\text{-}F}$ = 8.8 Hz), 134.3, 132.9, 130.2, 130.1 (d, $J_{C\text{-}F}$ = 3.8 Hz), 128.9, 128.8, 128.1, 127.9, 127.8, 123.1 (q, $J_{C\text{-}F}$ = 283.8 Hz), 117.8 (d, $J_{C\text{-}F}$ = 10.2 Hz), 116.2 (d, $J_{C\text{-}F}$ = 22.5 Hz), 93.6 (q, $J_{C\text{-}F}$ = 34.3 Hz), 73.3, 60.6, 57.6, 53.2, 52.5, 39.0, 38.8.

^{19}F NMR (376 MHz, $CDCl_3$) δ -79.4 (d, J = 5.6 Hz), -112.6 (q, J = 7.3 Hz).

HRMS (ESI/QTOF) m/z: [M + Na]$^+$ Calcd for $C_{29}H_{25}F_4NNaO_5^+$ 566.1561; Found 566.1572.

IR (ν_{max}, cm^{-1}) 3063 (w), 3032 (w), 2953 (w), 2847 (w), 1718 (s), 1612 (w), 1501 (m), 1441 (m), 1296 (s), 1230 (s), 1146 (s), 988 (m), 765 (s).

Methyl 6-benzyl-2-(2-methyl-5-nitrophenyl)-1-phenyl-5-(trifluoromethyl)-4-oxa-6-azaspiro[2.4]heptane-1-carboxylate (6s)

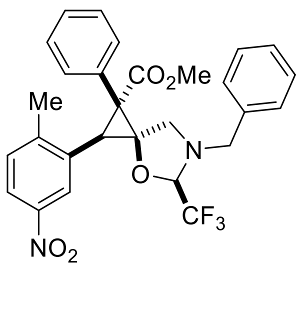

Following general procedure C, starting from **3a** (58 mg, 0.20 mmol, 1.0 equiv.), the crude product was purified by flash column chromatography (SiO_2, 12 g, 5 – 25% Et_2O in pentane) to afford **6s** as an off-white solid (49 mg, 0.092 mmol, 46%).

Melting Point: 127 – 128 °C.

TLC: R_f = 0.13 (SiO_2, 10% Et_2O in pentane).

^{1}H NMR (400 MHz, $CDCl_3$) δ 7.93 (dd, *J* = 8.4, 2.4 Hz, 1H, Ar*H*), 7.80 (d, *J* = 2.4 Hz, 1H, Ar*H*), 7.35 – 7.26 (m, 5H, Ar*H*), 7.25 – 7.10 (m, 4H, Ar*H*), 6.94 – 6.87 (m, 2H, Ar*H*), 5.05 (q, *J* = 5.7 Hz, 1H, C*H*CF_3), 4.06 – 3.92 (m, 2H, CH_2), 3.90 – 3.80 (m, 1H, CH_2), 3.66 (s, 3H, CO_2CH_3), 3.53 – 3.39 (m, 1H. CH_2), 3.21 (s, 1H, C*H*), 2.57 (s, 3H, ArCH_3).

^{13}C NMR (101 MHz, $CDCl_3$) δ 172.4, 146.1, 145.7, 136.8, 135.2, 132.0, 130.4, 130.3, 129.0, 128.8, 128.1, 127.9, 127.9, 123.5, 123.1 (q, J_{C-F} = 283.6 Hz), 121.6, 93.7 (q, J_{C-F} = 34.3 Hz), 74.3, 60.6, 58.2, 53.3, 39.5, 34.7, 21.1.

^{19}F NMR (376 MHz, $CDCl_3$) δ -79.1 (d, *J* = 5.6 Hz).

HRMS (ESI/QTOF) m/z: $[M + Na]^+$ Calcd for $C_{28}H_{25}F_3N_2NaO_5^+$ 549.1608; Found 549.1613.

IR (ν_{max}, cm^{-1}) 3062 (w), 2984 (s), 2903 (s), 1716 (s), 1521 (s), 1347 (s), 1238 (s), 1061 (s), 756 (s).

Methyl 6-benzyl-2-(3,5-bis(trifluoromethyl)phenyl)-1-phenyl-5-(trifluoromethyl)-4-oxa-6-azaspiro[2.4]heptane-1-carboxylate (6t)

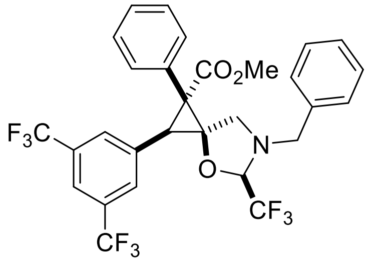

Following general procedure C, starting from **3a** (61 mg, 0.21 mmol, 1.0 equiv.), the crude product was purified by flash column chromatography (SiO_2, 12 g, 3 – 10% Et_2O in pentane, then SiO_2, 12 g 25 – 45% DCM in pentane) to afford **6t** as an off-white solid (51 mg, 0.084 mmol, 40%).

Melting Point: 85 – 86 °C.

TLC: R_f = 0.38 (SiO_2, 10% Et_2O in pentane).

^{1}H NMR (400 MHz, $CDCl_3$) δ 7.68 – 7.62 (m, 1H, Ar*H*), 7.30 – 7.16 (m, 10H, Ar*H*), 6.97 – 6.91 (m, 2H, Ar*H*), 4.88 (q, *J* = 5.6 Hz, 1H, C*H*CF_3), 3.93 (d, *J* = 13.5 Hz, 1H, CH_2), 3.88 – 3.78 (m, 2H, CH_2), 3.64 (s, 3H, CO_2CH_3), 3.54 – 3.47 (m, 1H, CH_2), 3.24 (s, 1H, C*H*).

^{13}C NMR (101 MHz, $CDCl_3$) δ 172.0, 136.8, 136.7, 132.6, 130.6 (q, J_{C-F} = 33.2 Hz), 130.2 (q, J_{C-F} = 3.7 Hz), 129.3, 128.8, 128.8, 128.2, 128.1, 128.1, 123.3 (q, J_{C-F} = 272.8 Hz), 123.1 (q, J_{C-F} = 283.0 Hz), 120.4 – 120.1 (m), 93.8 (q, J_{C-F} = 34.5 Hz), 73.6, 60.5, 57.4, 53.4, 40.0, 39.0.

^{19}F NMR (376 MHz, $CDCl_3$) δ -63.1, -79.4 (d, *J* = 5.5 Hz).

HRMS (ESI/QTOF) m/z: $[M + Na]^+$ Calcd for $C_{29}H_{22}F_9NNaO_3^+$ 626.1348; Found 626.1349.

IR (ν_{max}, cm^{-1}) 3031 (w), 2848 (w), 1719 (m), 1369 (m), 1279 (s), 1258 (m), 1236 (m), 1176 (s), 1132 (s), 983 (m).

Methyl 6-benzyl-1-phenyl-2-(quinolin-6-yl)-5-(trifluoromethyl)-4-oxa-6-azaspiro[2.4]heptane-1-carboxylate (6u)

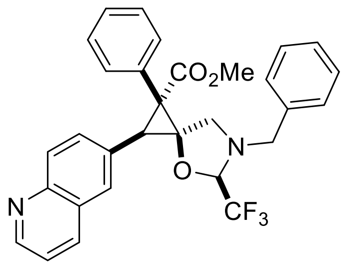

Following general procedure C, starting from **3a** (59 mg, 0.20 mmol, 1.0 equiv.), the crude product was purified by flash column chromatography (SiO_2, 12 g, 15 – 40% Et_2O in pentane) to afford **6u** as an off-white solid (61 mg, 0.12 mmol, 59%).

Melting Point: 165 – 167 °C.

TLC: R_f = 0.27 (SiO_2, 30% EtOAc in pentane).

^{1}H NMR (400 MHz, $CDCl_3$) δ 8.86 (dd, *J* = 4.3, 1.7 Hz, 1H, Ar*H*), 7.94 (dd, *J* = 8.5, 1.7 Hz, 1H, Ar*H*), 7.88 (d, *J* = 8.8 Hz, 1H, Ar*H*), 7.41 – 7.30 (m, 3H, Ar*H*), 7.27 – 7.15 (m, 8H, Ar*H*), 7.08 – 6.99 (m, 2H, Ar*H*), 4.84 (q, *J* = 5.6 Hz, 1H, C*H*CF_3), 3.98 – 3.78 (m, 3H, CH_2, CH_2), 3.64 (s, 3H, CO_2CH_3), 3.57 – 3.49 (m, 1H, CH_2), 3.41 (s, 1H, C*H*).

^{13}C NMR (101 MHz, $CDCl_3$) δ 172.5, 150.2, 147.2, 137.0, 136.1, 132.9, 132.7, 132.0, 130.4, 129.0, 128.8, 128.7, 128.3, 128.0, 127.8, 127.7, 127.7, 123.2 (q, J_{C-F} = 283.7 Hz), 121.2, 93.6 (q, J_{C-F} = 34.2 Hz), 73.7, 60.6, 57.7, 53.2, 39.9, 39.5.

^{19}F NMR (376 MHz, $CDCl_3$) δ -79.3 (d, *J* = 5.6 Hz).

HRMS (nanochip-ESI/LTQ-Orbitrap) m/z: $[M + H]^+$ Calcd for $C_{30}H_{26}F_3N_2O_3^+$ 519.1890; Found 519.1884.

IR (ν_{max}, cm^{-1}) 3033 (w), 2957 (w), 2901 (w), 1715 (s), 1501 (m), 1291 (m), 1234 (s), 1174 (s), 1147 (s), 1123 (s), 994 (m).

Methyl 6-benzyl-1-phenyl-2-(pyridin-4-yl)-5-(trifluoromethyl)-4-oxa-6-azaspiro[2.4]heptane-1-carboxylate (6v)

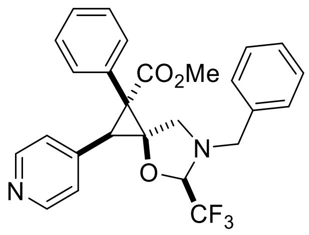

Following general procedure C, starting from **3a** (59 mg, 0.20 mmol, 1.0 equiv.), the crude product was purified by flash column chromatography (SiO_2, 12 g, 15 – 50% EtOAc in pentane) to afford **6v** as an off-white solid (41 mg, 0.088 mmol, 44%).

Melting Point: 143 – 144 °C.

TLC: R_f = 0.30 (SiO_2, 30% EtOAc in pentane).

^{1}H NMR (400 MHz, $CDCl_3$) δ 8.39 – 8.28 (m, 2H, Ar*H*), 7.31 – 7.20 (m, 8H, Ar*H*), 7.05 – 6.93 (m, 2H, Ar*H*), 6.86 – 6.74 (m, 2H, Ar*H*), 4.80 (q, *J* = 5.6 Hz, 1H, C*H*CF_3), 3.94 – 3.75 (m, 3H, CH_2, CH_2), 3.62 (s, 3H, CO_2CH_3), 3.50 – 3.41 (m, 1H, CH_2), 3.14 (s, 1H, C*H*).

^{13}C NMR (101 MHz, $CDCl_3$) δ 172.1, 149.0, 143.2, 136.9, 132.6, 129.9, 129.8, 128.8, 128.8, 128.1, 128.0, 125.2, 123.1 (q, $J_{C\text{-}F}$ = 284.4 Hz), 93.6 (q, $J_{C\text{-}F}$ = 33.3 Hz), 73.6, 60.6, 57.4, 53.3, 39.8, 38.8.

^{19}F NMR (376 MHz, $CDCl_3$) δ -79.4 (d, J = 6.8 Hz).

HRMS (APCI/QTOF) m/z: $[M + H]^+$ Calcd for $C_{26}H_{24}F_3N_2O_3^+$ 469.1734; Found 469.1729.

IR (v_{max}, cm^{-1}) 3028 (w), 2955 (m), 1718 (s), 1599 (m), 1498 (w), 1292 (m), 1238 (s), 1175 (s), 993 (m), 770 (s).

Methyl 6-benzyl-2-(2-fluoropyridin-4-yl)-1-phenyl-5-(trifluoromethyl)-4-oxa-6-azaspiro[2.4]heptane-1-carboxylate (6w)

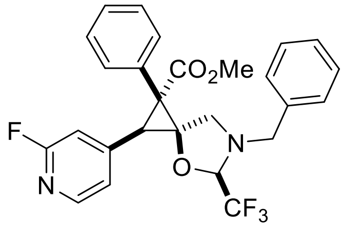

Following general procedure C, starting from **3a** (60 mg, 0.20 mmol, 1.0 equiv.), the crude product was purified by flash column chromatography (SiO_2, 12 g, 5 – 15% Et_2O in pentane), afford **6w** as an off-white solid (57 mg, 0.12 mmol, 57%).

Melting Point: 171 – 172 °C.

TLC: R_f = 0.28 (SiO_2, 10% Et_2O in pentane).

^1H NMR (400 MHz, $CDCl_3$) δ 7.94 (d, J = 5.3 Hz, 1H, Ar*H*), 7.32 – 7.18 (m, 8H, Ar*H*), 7.04 – 6.92 (m, 2H, Ar*H*), 6.72 (dt, J = 5.3, 1.6 Hz, 1H, Ar*H*), 6.41 (d, J = 1.3 Hz, 1H, Ar*H*), 4.84 (q, J = 5.6 Hz, 1H, C*H*CF_3), 3.90 (d, J = 13.3 Hz, 1H, CH_2), 3.85 – 3.76 (m, 2H, C*H2*, C*H2*), 3.62 (s, 3H, CO_2CH_3), 3.49 – 3.41 (m, 1H, CH_2), 3.16 (s, 1H, C*H*).

^{13}C NMR (101 MHz, $CDCl_3$) δ 171.8, 163.4 (d, $J_{C\text{-}F}$ = 237.2 Hz), 149.1 (d, $J_{C\text{-}F}$ = 8.7 Hz), 146.4 (d, $J_{C\text{-}F}$ = 15.3 Hz), 136.7, 132.4, 129.5, 128.8 (2C), 128.2, 128.1, 123.0 (q, $J_{C\text{-}F}$ = 283.7 Hz), 122.9, 122.9, 110.6 (d, $J_{C\text{-}F}$ = 38.9 Hz), 110.4, 93.8 (q, $J_{C\text{-}F}$ = 34.5 Hz), 73.7, 60.6, 57.3, 53.4, 40.3, 38.5 (d, $J_{C\text{-}F}$ = 3.5 Hz).

^{19}F NMR (376 MHz, $CDCl_3$) δ -68.9, -79.4 (d, J = 5.6 Hz).

HRMS (ESI/QTOF) m/z: $[M + H]^+$ Calcd for $C_{26}H_{23}F_4N_2O_3^+$ 487.1639; Found 487.1647.

IR (v_{max}, cm^{-1}) 3068 (w), 3032 (w), 2954 (w), 2918 (w), 2848 (w), 1719 (m), 1611 (m), 1553 (m), 1292 (m), 1220 (s), 1150 (s), 1124 (m), 995 (m), 770 (s).

Methyl 6-benzyl-1-phenyl-2-(thiophen-2-yl)-5-(trifluoromethyl)-4-oxa-6-azaspiro[2.4]heptane-1-carboxylate (6x)

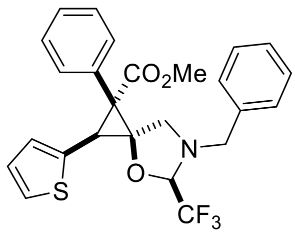

Following general procedure C, starting from **3a** (61 mg, 0.21 mmol, 1.0 equiv.), the crude product was purified by flash column chromatography (SiO_2, 12 g, 5 – 15% Et_2O in pentane) to afford **6x** as an off-white solid (31 mg, 0.065 mmol, 31%).

Melting Point: 109 – 110 °C.

TLC: R_f = 0.26 (SiO_2, 10% Et_2O in pentane).

^1H NMR (400 MHz, $CDCl_3$) δ 7.35 – 7.27 (m, 5H, Ar*H*), 7.24 – 7.16 (m, 3H, Ar*H*), 7.11 (dd, *J* = 5.2, 1.2 Hz, 1H, Ar*H*), 7.09 – 7.04 (m, 2H, Ar*H*), 6.89 (dd, *J* = 5.1, 3.6 Hz, 1H, Ar*H*), 6.81 (dd, *J* = 3.7, 1.2 Hz, 1H, Ar*H*), 4.86 (q, *J* = 5.6 Hz, 1H, C*H*CF_3), 3.97 (d, *J* = 13.1 Hz, 1H, CH_2), 3.89 (d, *J* = 13.1 Hz, 1H, CH_2), 3.84 – 3.77 (m, 1H, CH_2), 3.62 (s, 3H, CO_2CH_3), 3.56 (s, 1H, C*H*), 3.52 – 3.45 (m, 1H, CH_2).

^{13}C NMR (101 MHz, $CDCl_3$) δ 172.3, 137.2, 135.0, 132.6, 130.7, 128.9, 128.7, 128.0, 127.9, 127.6, 127.6, 126.3, 125.2, 123.1 (q, $J_{C\text{-}F}$ = 283.5 Hz), 93.4 (q, $J_{C\text{-}F}$ = 34.3 Hz), 73.1, 60.6, 57.2, 53.1, 39.0, 36.1.

^{19}F NMR (376 MHz, $CDCl_3$) δ -79.5 (d, *J* = 5.5 Hz).

HRMS (nanochip-ESI/LTQ-Orbitrap) m/z: $[M + H]^+$ Calcd for $C_{25}H_{23}F_3NO_3S^+$ 474.1345; Found 474.1342.

IR (ν_{max}, cm^{-1}) 3085 (m), 3064 (m), 3031 (m), 2952 (m), 2845 (m), 1956 (w), 1880 (w), 1805 (w), 1714 (s), 1437 (m), 1292 (s), 1233 (s), 1174 (s), 1146 (s), 1122 (s), 991 (s), 758 (s).

Methyl 6-benzyl-1-(4-chlorophenyl)-5-(trifluoromethyl)-2-(4-(trifluoromethyl)phenyl)-4-oxa-6-azaspiro[2.4]heptane-1-carboxylate (7aa)

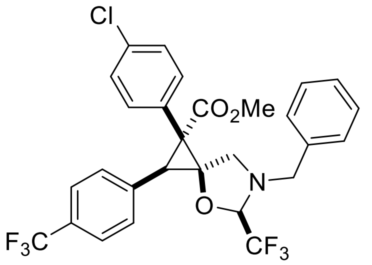

Following general procedure C, starting from **3b** (66 mg, 0.20 mmol, 1.0 equiv.), the crude product was purified by flash column chromatography (SiO_2, 12 g, 5 – 15% Et_2O in pentane) to afford **7aa** as an off-white solid (85 mg, 0.15 mmol, 75%).

Gram scale procedure: prepared according to the general procedure C using **3b** (1.02 g, 3.10 mmol, 1.00 equiv.), in anhydrous DCE (15.5 mL, 0.2 M). The crude material was purified by flash column chromatography (SiO_2, 40 g, 3 – 15% Et2O in pentane) to afford **7aa** as an off-white solid (1.03 g, 1.81 mmol, 58%).

Melting Point: 149 – 151 °C.

TLC: R_f = 0.29 (SiO_2, 10% Et_2O in pentane).

^1H NMR (400 MHz, $CDCl_3$) δ 7.41 (d, *J* = 8.2 Hz, 2H, Ar*H*), 7.30 – 7.22 (m, 5H, Ar*H*), 7.22 – 7.17 (m, 2H, Ar*H*), 7.05 (d, *J* = 8.2 Hz, 2H, Ar*H*), 6.94 – 6.87 (m, 2H, Ar*H*), 4.81 (q, *J* = 5.6 Hz, 1H, C*H*CF_3), 3.89 (d, *J* = 13.3 Hz, 1H, CH_2), 3.84 – 3.75 (m, 2H, CH_2, CH_2), 3.62 (s, 3H, CO_2CH_3), 3.50 – 3.41 (m, 1H, CH_2), 3.24 (s, 1H, C*H*).

^{13}C NMR (101 MHz, $CDCl_3$) δ 171.9, 137.9 (q, $J_{C\text{-}F}$ = 1.5 Hz), 136.9, 134.1, 134.0, 130.5, 129.3 (q, $J_{C\text{-}F}$ = 32.2 Hz), 128.8, 128.8, 128.8, 128.3, 128.1, 124.6 (q, $J_{C\text{-}F}$ = 3.8 Hz), 124.3 (q, $J_{C\text{-}F}$ = 271.9 Hz), 123.1 (q, $J_{C\text{-}F}$ = 283.6 Hz), 93.7 (q, $J_{C\text{-}F}$ = 34.3 Hz), 73.5, 60.6, 57.5, 53.3, 39.5, 38.9.

^{19}F NMR (376 MHz, $CDCl_3$) δ -62.5, -79.4 (d, *J* = 5.6 Hz).

HRMS (nanochip-ESI/LTQ-Orbitrap) m/z: $[M + H]^+$ Calcd for $C_{28}H_{23}ClF_6NO_3^+$ 570.1265; Found 570.1261.

IR (v_{max}, cm^{-1}) 3066 (w), 3032 (w), 2954 (w), 2849 (w), 1720 (m), 1619 (m), 1495 (m), 1326 (s), 1256 (s), 1236 (s), 1169 (s), 1120 (s), 1015 (m), 912 (m), 732 (s).

Methyl 6-benzyl-1-(4-chlorophenyl)-2-(4-(methoxycarbonyl)phenyl)-5-(trifluoromethyl)-4-oxa-6-azaspiro[2.4]heptane-1-carboxylate (7ab)

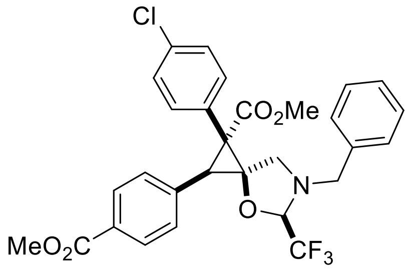

Following general procedure C, starting from **3b** (66 mg, 0.20 mmol, 1.0 equiv.), the crude product was purified by flash column chromatography (SiO_2, 12 g, 5 – 20% Et_2O in pentane) to afford **7ab** as an off-white solid (99 mg, 0.18 mmol, 87%).

Melting Point: 161 – 163 °C.

TLC: R_f = 0.14 (SiO_2, 10% Et_2O in pentane).

^1H NMR (400 MHz, $CDCl_3$) δ 7.86 – 7.78 (m, 2H, Ar*H*), 7.32 – 7.21 (m, 5H, Ar*H*), 7.21 – 7.15 (m, 2H, Ar*H*), 7.06 – 6.99 (m, 2H, Ar*H*), 6.94 – 6.88 (m, 2H, Ar*H*), 4.82 (q, *J* = 5.6 Hz, 1H, C*H*CF_3), 3.94 – 3.87 (m, 4H, CO_2CH_3, CH_2), 3.84 – 3.76 (m, 2H, CH_2, CH_2), 3.62 (s, 3H, CO_2CH_3), 3.51 – 3.42 (m, 1H, CH_2), 3.25 (s, 1H, C*H*).

^{13}C NMR (101 MHz, $CDCl_3$) δ 171.9, 167.1, 139.2, 136.9, 134.1, 133.9, 130.2, 128.9, 128.9, 128.8, 128.8, 128.6, 128.2, 128.1, 123.1 (q, J_{C-F} = 283.6 Hz), 93.6 (q, J_{C-F} = 34.3 Hz), 73.7, 60.5, 57.5, 53.3, 52.2, 39.8, 39.0.

^{19}F NMR (376 MHz, $CDCl_3$) δ -79.3 (d, *J* = 5.7 Hz).

HRMS (nanochip-ESI/LTQ-Orbitrap) m/z: $[M + H]^+$ Calcd for $C_{29}H_{26}ClF_3NO_5^+$ 560.1446; Found 560.1439.

IR (v_{max}, cm^{-1}) 3030 (w), 2953 (w), 2846 (w), 1718 (s), 1610 (w), 1495 (m), 1436 (m), 1282 (s), 1176 (s), 990 (m), 758 (s), 729 (s).

Methyl 6-benzyl-1-(4-chlorophenyl)-2-(pyridin-4-yl)-5-(trifluoromethyl)-4-oxa-6-azaspiro[2.4]heptane-1-carboxylate (7ac)

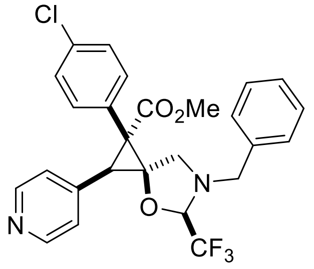

Following general procedure C, starting from **3b** (67 mg, 0.20 mmol, 1.0 equiv.), the crude product was purified by flash column chromatography (SiO_2, 12 g, 15 – 50% Et_2O in pentane), afford **7ac** as an off-white solid (56 mg, 0.11 mmol, 54%).

Melting Point: 153 – 155 °C.

TLC: R_f = 0.22 (SiO_2, 50% Et_2O in pentane).

^{1}H NMR (400 MHz, $CDCl_3$) δ 8.41 – 8.32 (m, 2H, Ar*H*), 7.31 – 7.27 (m, 1H, Ar*H*), 7.25 – 7.18 (m, 4H, Ar*H*), 6.94 – 6.88 (m, 2H, Ar*H*), 6.87 – 6.78 (m, 2H, Ar*H*), 4.82 (q, *J* = 5.6 Hz, 1H, C*H*CF_3), 3.89 (d, *J* = 13.2 Hz, 1H, CH_2), 3.83 – 3.75 (m, 2H, CH_2, CH_2), 3.63 (s, 3H, CO_2CH_3), 3.50 – 3.41 (m, 1H, CH_2), 3.13 (s, 1H, C*H*).

^{13}C NMR (101 MHz, $CDCl_3$) δ 171.6, 149.1, 142.9, 136.7, 134.1, 133.9, 128.8, 128.8, 128.5, 128.4, 128.2, 125.1, 123.1 (q, $J_{C\text{-}F}$ = 283.9 Hz), 93.7 (q, $J_{C\text{-}F}$ = 34.6 Hz), 73.6, 60.5, 57.4, 53.4, 39.2, 38.8.

^{19}F NMR (376 MHz, $CDCl_3$) δ -79.4 (d, *J* = 5.5 Hz).

HRMS (ESI/QTOF) m/z: $[M + H]^+$ Calcd for $C_{26}H_{23}ClF_3N_2O_3^+$ 503.1344; Found 503.1357.

IR (v_{max}, cm^{-1}) 3060 (w), 2976 (s), 2902 (s), 1721 (m), 1598 (m), 1406 (m), 1239 (s), 1176 (s), 1058 (s), 993 (m), 768 (s).

Methyl 6-benzyl-1-(4-methoxyphenyl)-5-(trifluoromethyl)-2-(4-(trifluoromethyl)phenyl)-4-oxa-6-azaspiro[2.4]heptane-1-carboxylate (7b)

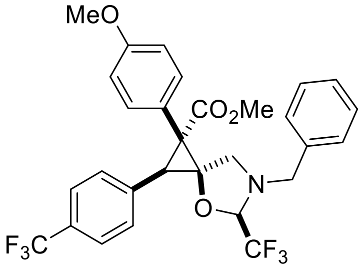

Following general procedure C, starting from **3c** (62 mg, 0.19 mmol, 1.0 equiv.), the crude product was purified by flash column chromatography (SiO_2, 12 g, 3 – 10% Et_2O in pentane) to afford **7b** as an off-white solid (62 mg, 0.11 mmol, 57%).

Melting Point: 125 – 126 °C.

TLC: R_f = 0.17 (SiO_2, 10% Et_2O in pentane).

^{1}H NMR (400 MHz, $CDCl_3$) δ 7.42 – 7.35 (m, 2H, Ar*H*), 7.31 – 7.26 (m, 2H, Ar*H*), 7.25 (d, *J* = 7.0 Hz, 3H, Ar*H*), 7.08 – 7.02 (m, 2H, Ar*H*), 6.94 – 6.87 (m, 2H, Ar*H*), 6.79 – 6.72 (m, 2H, Ar*H*), 4.79 (q, *J* = 5.6 Hz, 1H, C*H*CF_3), 3.92 – 3.75 (m, 6H, ArOCH_3, CH_2, CH_2), 3.62 (s, 3H, CO_2CH_3), 3.48 – 3.40 (m, 1H, CH_2), 3.22 (s, 1H, C*H*).

^{13}C NMR (101 MHz, $CDCl_3$) δ 172.7, 159.1, 138.3, 137.0, 133.8, 130.5, 128.9 (q, *J* = 32.4 Hz), 128.8, 128.8, 128.5, 128.1, 124.45 (q, $J_{C\text{-}F}$ = 3.7 Hz), 124.43 (q, $J_{C\text{-}F}$ = 271.9 Hz), 123.15 (q, $J_{C\text{-}F}$ = 283.8 Hz), 122.1, 113.4, 93.6 (q, $J_{C\text{-}F}$ = 34.5 Hz), 73.6, 60.6, 57.5, 55.2, 53.2, 39.6, 38.9.

^{19}F NMR (376 MHz, $CDCl_3$) δ -62.4, -79.4 (d, *J* = 5.6 Hz).

HRMS (APCI/QTOF) m/z: $[M + H]^+$ Calcd for $C_{29}H_{26}F_6NO_4^+$ 566.1761; Found 566.1764.

IR (v_{max}, cm^{-1}) 3065 (w), 3031 (w), 2956 (w), 2901 (w), 2848 (w), 1721 (m), 1498 (w), 1457 (w), 1311 (s), 1297 (s), 1236 (s), 1159 (s), 1120 (s), 770 (s).

Methyl 6-benzyl-1-(4-(methoxycarbonyl)phenyl)-5-(trifluoromethyl)-2-(4-(trifluoromethyl)phenyl)-4-oxa-6-azaspiro[2.4]heptane-1-carboxylate (7c)

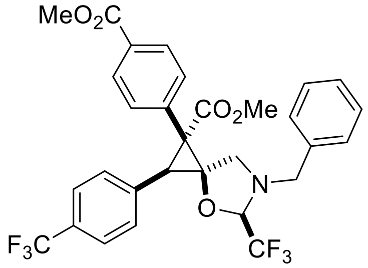

Following general procedure C, starting from **3d** (72, 0.20 mmol, 1.0 equiv.), the crude product was purified by flash column chromatography (SiO_2, 12 g, 5 – 15% Et_2O in pentane) to afford **7c** as an off-white amorphous foam (102 mg, 0.17 mmol, 84%).

TLC: R_f = 0.37 (SiO_2, 10% Et_2O in pentane).

^{1}H NMR (400 MHz, $CDCl_3$) δ 7.93 – 7.85 (m, 2H, Ar*H*), 7.42 – 7.36 (m, 2H, Ar*H*), 7.31 – 7.26 (m, 2H, Ar*H*), 7.26 – 7.21 (m, 3H, Ar*H*), 7.10 – 7.05 (m, 2H, Ar*H*), 7.04 – 6.99 (m, 2H, Ar*H*), 4.82 (q, *J* = 5.6 Hz, 1H, C*H*CF_3), 3.89 (d, *J* = 8.4 Hz, 4H, C*H*$_2$, C*H*$_3$), 3.85 – 3.77 (m, 2H, C*H*$_2$), 3.62 (s, 3H, CO_2C*H*$_3$), 3.53 – 3.45 (m, 1H, C*H*$_2$), 3.27 (s, 1H, C*H*).

^{13}C NMR (101 MHz, $CDCl_3$) δ 171.7, 167.0, 137.7 (q, J_{C-F} = 1.1 Hz), 136.8, 135.5, 132.8, 130.4, 129.6, 129.1, 129.0 (q, *J* = 32.4 Hz), 128.8, 128.8, 128.1, 124.6 (q, J_{C-F} = 3.8 Hz), 124.3 (q, J_{C-F} = 272.0 Hz), 123.1 (q, J_{C-F} = 283.6 Hz), 93.7 (q, J_{C-F} = 34.3 Hz), 73.5, 60.6, 57.5, 53.3, 52.2, 39.6, 39.3.

^{19}F NMR (376 MHz, $CDCl_3$) δ -62.5, -79.4 (d, *J* = 5.7 Hz).

HRMS (ESI/QTOF) m/z: [M + H]$^+$ Calcd for $C_{30}H_{26}F_6NO_5^+$ 594.1710; Found 594.1717.

IR (ν_{max}, cm^{-1}) 3033 (w), 2952 (w), 2845 (w), 1718 (m), 1615 (w), 1437 (w), 1326 (m), 1282 (m), 1218 (m), 1170 (m), 1119 (s), 990 (m), 849 (w), 752 (s).

Methyl 6-benzyl-2-(4-(methoxycarbonyl)phenyl)-1-(p-tolyl)-5-(trifluoromethyl)-4-oxa-6-azaspiro[2.4]heptane-1-carboxylate (7d)

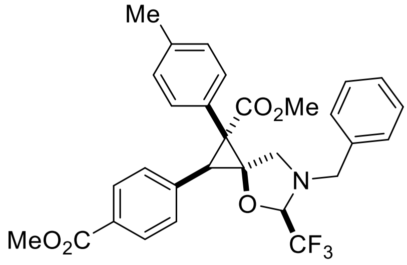

Following general procedure C, starting from **3e** (61 mg, 0.20 mmol, 1.0 equiv.), the crude product was purified by flash column chromatography (SiO_2, 12 g, 5 – 15% Et_2O in pentane) to afford **7d** as an off-white solid (70 mg, 0.13 mmol, 65%).

Melting Point: 154 – 156 °C.

TLC: R_f = 0.29 (SiO_2, 10% Et_2O in pentane).

^{1}H NMR (400 MHz, $CDCl_3$) δ 7.85 – 7.76 (m, 2H, Ar*H*), 7.31 – 7.26 (m, 2H, Ar*H*), 7.26 – 7.20 (m, 3H, Ar*H*), 7.07 – 6.96 (m, 4H, Ar*H*), 6.92 – 6.83 (m, 2H, Ar*H*), 4.78 (q, *J* = 5.6 Hz, 1H,

C*H*CF_3), 3.92 – 3.86 (m, 4H, C*H*$_2$, CO_2CH_3), 3.84 – 3.75 (m, 2H, C*H*$_2$), 3.62 (s, 3H, CO_2CH_3), 3.49 – 3.40 (m, 1H, C*H*$_2$), 3.23 (s, 1H, C*H*), 2.31 (s, 3H, ArC*H*$_3$).

^{13}C NMR (101 MHz, $CDCl_3$) δ 172.5, 167.2, 139.6, 137.4, 136.9, 132.4, 130.1, 128.7, 128.7, 128.6, 128.5, 128.1, 127.9, 127.0, 123.0 (q, J_{C-F} = 283.8 Hz), 93.4 (q, J_{C-F} = 34.2 Hz), 73.6, 60.4, 57.4, 53.1, 52.0, 39.7, 39.3, 21.3.

^{19}F NMR (376 MHz, $CDCl_3$) δ -79.4 (d, J = 5.6 Hz).

HRMS (ESI/QTOF) m/z: [M + H]$^+$ Calcd for $C_{30}H_{29}F_3NO_5^+$ 540.1992; Found 540.2002.

IR (v_{max}, cm^{-1}) 3060 (w), 3030 (w), 2953 (w), 2846 (w), 1717 (s), 1611 (w), 1436 (m), 1281 (s), 1233 (s), 1175 (s), 1147 (s), 1119 (s), 1018 (m), 992 (m), 756 (s).

Methyl 6-benzyl-1-(2-fluorophenyl)-2-(4-(methoxycarbonyl)phenyl)-5-(trifluoromethyl)-4-oxa-6-azaspiro[2.4]heptane-1-carboxylate (7e)

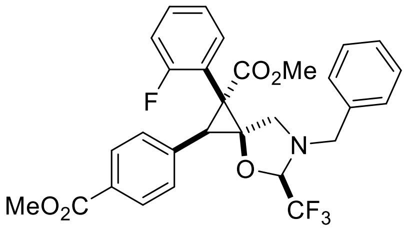

Following general procedure C, starting from **3f** (62 mg, 0.20 mmol, 1.0 equiv.), the crude product was purified by flash column chromatography (SiO_2, 12 g, 5 – 15% Et_2O in pentane) to afford **7e** as an off-white solid (70 mg, 0.13 mmol, 64%).

Melting Point: 139 – 139 °C.

TLC: R_f = 0.32 (SiO_2, 10% Et_2O in pentane).

^{1}H NMR (400 MHz, $CDCl_3$) δ 7.81 – 7.72 (m, 2H, Ar*H*), 7.35 – 7.27 (m, 1H, Ar*H*), 7.26 – 7.18 (m, 6H, Ar*H*), 7.10 – 6.98 (m, 3H, Ar*H*), 6.86 – 6.74 (m, 1H, Ar*H*), 4.79 (q, J = 5.6 Hz, 1H, C*H*CF_3), 3.90 (d, J = 13.2 Hz, 1H, C*H*$_2$), 3.86 (s, 3H, CO_2CH_3), 3.82 – 3.73 (m, 2H, C*H*$_2$, C*H*$_2$), 3.62 (s, 3H, CO_2CH_3), 3.54 – 3.46 (m, 1H, C*H*$_2$), 3.29 (s, 1H, C*H*).

^{13}C NMR (101 MHz, $CDCl_3$) δ 171.6, 167.2, 162.4 (d, J_{C-F} = 249.2 Hz), 139.4, 137.0, 133.4 (d, J_{C-F} = 3.3 Hz), 130.1 (d, J_{C-F} = 8.6 Hz), 129.6, 128.8, 128.8, 128.5, 128.1, 123.6 (d, J_{C-F} = 3.5 Hz), 123.1 (q, J_{C-F} = 283.6 Hz), 118.9 (d, J_{C-F} = 14.7 Hz), 115.2 (d, J_{C-F} = 21.5 Hz), 93.7 (q, J_{C-F} = 34.3 Hz), 73.7, 60.6, 57.5, 53.3, 52.2, 40.3, 35.4. One carbon is not resolved.

^{19}F NMR (376 MHz, $CDCl_3$) δ -79.4 – -79.5 (m), -112.0 – -112.2 (m).

HRMS (ESI/QTOF) m/z: [M + H]$^+$ Calcd for $C_{29}H_{26}F_4NO_5^+$ 544.1742; Found 544.1745.

IR (v_{max}, cm^{-1}) 3064 (w), 2984 (s), 2969 (s), 2903 (s), 1720 (s), 1612 (w), 1496 (m), 1437 (m), 1282 (s), 1254 (s), 1234 (s), 1061 (s).

Methyl 6-benzyl-2-(4-(methoxycarbonyl)phenyl)-1-(3-methoxyphenyl)-5-(trifluoromethyl)-4-oxa-6-azaspiro[2.4]heptane-1-carboxylate (7f)

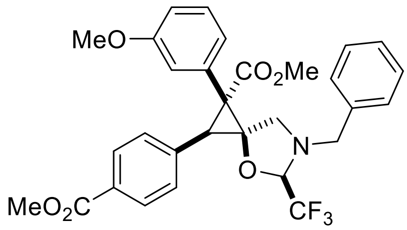

Following general procedure C, starting from **3g** (65 mg, 0.20 mmol, 1.0 equiv.), the crude product was purified by flash column chromatography (SiO_2, 12 g, 10 – 25% Et_2O in pentane) to afford **7f** as a colourless amorphous foam (65 mg, 0.12 mmol, 59%).

TLC: R_f = 0.34 (SiO_2, 20% Et_2O in pentane).

^{1}H NMR (400 MHz, $CDCl_3$) δ 7.85 – 7.76 (m, 2H, Ar*H*), 7.31 – 7.26 (m, 2H, Ar*H*), 7.26 – 7.21 (m, 3H, Ar*H*), 7.11 – 7.01 (m, 3H, Ar*H*), 6.80 (ddd, *J* = 8.3, 2.6, 1.0 Hz, 1H, Ar*H*), 6.65 (dd, *J* = 2.6, 1.5 Hz, 1H, Ar*H*), 6.50 (ddd, *J* = 7.5, 1.5, 1.0 Hz, 1H, Ar*H*), 4.81 (q, *J* = 5.6 Hz, 1H, C*H*CF_3), 3.89 (s, 4H, CO_2CH_3, CH_2), 3.83 – 3.76 (m, 2H, CH_2, CH_2), 3.66 (s, 3H, ArOCH_3), 3.63 (s, 3H, CO_2CH_3), 3.49 – 3.42 (m, 1H, CH_2), 3.24 (s, 1H, C*H*).

^{13}C NMR (101 MHz, $CDCl_3$) δ 172.3, 167.2, 159.1, 139.6, 137.0, 131.6, 130.2, 128.8, 128.8, 128.8, 128.7, 128.3, 128.1, 125.2, 123.2 (q, $J_{C\text{-}F}$ = 283.2 Hz), 117.4, 114.4, 93.6 (q, $J_{C\text{-}F}$ = 34.2 Hz), 73.7, 60.6, 57.6, 55.1, 53.2, 52.2, 39.8, 39.7.

^{19}F NMR (376 MHz, $CDCl_3$) δ -79.3 (d, *J* = 5.6 Hz).

HRMS (ESI/QTOF) m/z: $[M + Na]^+$ Calcd for $C_{30}H_{28}F_3NNaO_6^+$ 578.1761; Found 578.1764.

IR ($ν_{max}$, cm^{-1}) 3031 (w), 2953 (w), 2842 (w), 1720 (s), 1608 (m), 1435 (m), 1284 (s), 1249 (s), 1178 (s), 993 (m), 864 (m).

Methyl 6-benzyl-1-(2-chloro-4-fluorophenyl)-2-(4-(methoxycarbonyl)phenyl)-5-(trifluoromethyl)-4-oxa-6-azaspiro[2.4]heptane-1-carboxylate (7g)

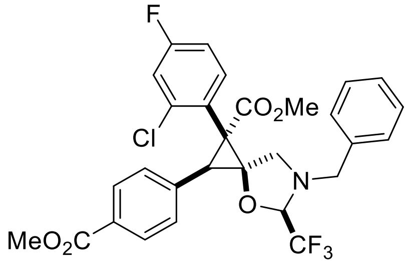

Following general procedure C, starting from **3h** (69 mg, 0.20 mmol, 1.0 equiv.), the crude product was purified by flash column chromatography (SiO_2, 12 g, 5 – 15% Et_2O in pentane), afford **7g** as an off-white amorphous foam (88 mg, 0.15 mmol, 76%).

TLC: R_f = 0.23 (SiO_2, 10% Et_2O in pentane).

^{1}H NMR (400 MHz, $CDCl_3$) δ 7.78 (d, *J* = 8.1 Hz, 2H, Ar*H*), 7.64 (s, 1H, Ar*H*), 7.31 – 7.18 (m, 5H, Ar*H*), 7.13 – 6.82 (m, 4H, Ar*H*), 4.92 – 4.71 (m, 1H, C*H*CF_3), 3.93 – 3.85 (m, 4H, CH_2, CO_2CH_3), 3.79 (d, *J* = 13.2 Hz, 1H, CH_2), 3.73 (d, *J* = 13.3 Hz, 1H, CH_2), 3.64 (s, 3H, CO_2CH_3), 3.49 (d, *J* = 13.3 Hz, 1H, CH_2), 3.41 (s, 1H, C*H*).

^{13}C NMR (101 MHz, $CDCl_3$) δ 171.1, 167.1, 162.1 (d, $J_{C\text{-}F}$ = 251.0 Hz), 138.7, 138.0 (d, $J_{C\text{-}F}$ = 10.3 Hz), 136.7, 134.4 (d, $J_{C\text{-}F}$ = 8.8 Hz), 129.4, 128.7, 128.7, 128.4, 128.0, 125.9, 123.0 (q, $J_{C\text{-}F}$ = 283.6 Hz), 116.9 (d, $J_{C\text{-}F}$ = 24.7 Hz), 113.7 (d, $J_{C\text{-}F}$ = 20.7 Hz), 93.7 (q, $J_{C\text{-}F}$ = 34.3 Hz), 73.7, 60.4, 57.4, 53.2, 52.0, 40.8, 37.8. One carbon is not resolved.

^{19}F NMR (376 MHz, $CDCl_3$) δ -79.3, -111.2.

HRMS (ESI/QTOF) m/z: $[M + H]^+$ Calcd for $C_{29}H_{25}ClF_4NO_5^+$ 578.1352; Found 578.1359.

IR (v_{max}, cm^{-1}) 3070 (w), 3031 (w), 2953 (w), 2846 (w), 1720 (s), 1607 (m), 1495 (m), 1437 (m), 1282 (s), 1234 (s), 1179 (s), 1120 (s), 988 (m).

Benzyl 6-benzyl-2-(4-(methoxycarbonyl)phenyl)-1-phenyl-5-(trifluoromethyl)-4-oxa-6-azaspiro[2.4]heptane-1-carboxylate (7h)

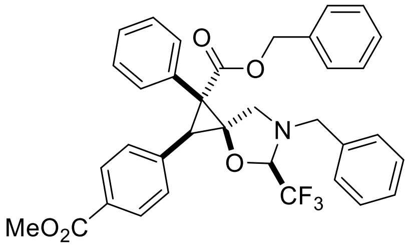

Following general procedure C, starting from **3i** (74 mg, 0.20 mmol, 1.0 equiv.), the crude product was purified by flash column chromatography (SiO_2, 12 g, 10 – 25% Et_2O in pentane) to afford **7h** as a colourless amorphous foam (82 mg, 0.14 mmol, 68%).

TLC: R_f = 0.12 (SiO_2, 10% Et_2O in pentane).

^{1}H NMR (400 MHz, $CDCl_3$) δ 7.84 – 7.74 (m, 2H, Ar*H*), 7.31 – 7.26 (m, 2H, Ar*H*), 7.26 – 7.18 (m, 9H, Ar*H*), 7.09 – 6.97 (m, 6H, Ar*H*), 5.12 – 5.01 (m, 2H, ArC*H*$_2$), 4.79 (q, *J* = 5.6 Hz, 1H, C*H*CF_3), 3.91 – 3.84 (m, 4H, CO_2CH_3, CH_2), 3.78 (d, *J* = 13.2 Hz, 1H, C*H*$_2$), 3.75 – 3.67 (m, 1H. C*H*$_2$), 3.43 – 3.33 (m, 1H, C*H*$_2$), 3.25 (s, 1H, C*H*).

^{13}C NMR (101 MHz, $CDCl_3$) δ 171.6, 167.2, 139.5, 137.0, 135.8, 132.7, 130.3, 130.2, 128.8, 128.8, 128.8, 128.6, 128.3, 128.1, 128.0, 127.9, 127.8, 127.1, 123.1 (q, $J_{C\text{-}F}$ = 283.6 Hz), 93.6 (q, $J_{C\text{-}F}$ = 34.3 Hz), 73.7, 67.3, 60.5, 57.5, 52.2, 39.9, 39.6.

^{19}F NMR (376 MHz, $CDCl_3$) δ -79.3 (d, *J* = 5.8 Hz).

HRMS (ESI/QTOF) m/z: $[M + H]^+$ Calcd for $C_{35}H_{31}F_3NO_5^+$ 602.2149; Found 602.2149.

IR (v_{max}, cm^{-1}) 3063 (w), 3030 (m), 2954 (m), 2902 (m), 2847 (w), 1718 (s), 1610 (m), 1438 (m), 1281 (s), 1219 (s), 1176 (s), 1119 (s), 992 (s), 862 (m), 769 (s).

Methyl 4-(-6-benzyl-2-phenyl-2,5-bis(trifluoromethyl)-4-oxa-6-azaspiro[2.4]heptan-1-yl)benzoate (7i)

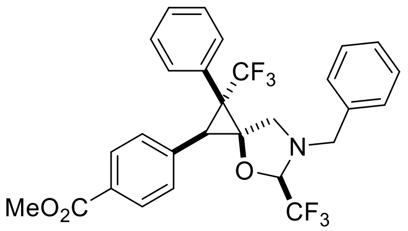

Following general procedure C, starting from **3j** (61 mg, 0.20 mmol, 1.0 equiv.), the crude product was purified by flash column chromatography (SiO_2, 12 g, 5 – 15% Et_2O in pentane) to afford **7i** as a colourless amorphous foam (73 mg, 0.14 mmol, 68%).

TLC: R_f = 0.27 (SiO_2, 5% EtOAc in pentane).

^{1}H NMR (400 MHz, $CDCl_3$) δ 7.86 – 7.80 (m, 2H, Ar*H*), 7.35 – 7.26 (m, 4H, Ar*H*), 7.26 – 7.19 (m, 4H, Ar*H*), 7.10 – 7.04 (m, 2H, Ar*H*), 7.04 – 6.98 (m, 2H, Ar*H*), 4.74 (q, *J* = 5.5 Hz, 1H,

C*H*CF_3), 4.04 (d, *J* = 13.2 Hz, 1H, C*H*$_2$), 3.92 – 3.84 (m, 4H, CO_2CH_3, C*H*$_2$), 3.78 (d, *J* = 13.2 Hz, 1H, C*H*$_2$), 3.30 (d, *J* = 12.9 Hz, 1H, C*H*$_2$), 2.84 (s, 1H. C*H*).

^{13}C NMR (101 MHz, $CDCl_3$) δ 167.1, 138.4, 136.8, 133.7, 130.2, 128.9, 128.9, 128.8, 128.8, 128.7, 128.2, 127.9, 126.8, 125.1 (d, J_{C-F} = 275.9 Hz), 123.0 (d, J_{C-F} = 283.4 Hz), 93.0 (q, J_{C-F} = 34.5 Hz), 70.1, 60.5, 56.9, 52.2, 37.1 (q, J_{C-F} = 32.5 Hz), 34.8 (q, J_{C-F} = 2.5 Hz).

^{19}F NMR (376 MHz, $CDCl_3$) δ -66.8, -79.5 (d, *J* = 5.5 Hz).

HRMS (ESI/QTOF) m/z: [M + H]$^+$ Calcd for $C_{28}H_{24}F_6NO_3^+$ 536.1655; Found 536.1660.

IR (v_{max}, cm^{-1}) 3065 (w), 2959 (m), 2899 (w), 1721 (s), 1612 (w), 1280 (s), 1139 (s), 1049 (m), 861 (w), 765 (s).

6-Benzyl-2-(4-chlorophenyl)-1-phenyl-1,5-bis(trifluoromethyl)-4-oxa-6-azaspiro[2.4]heptane (7j)

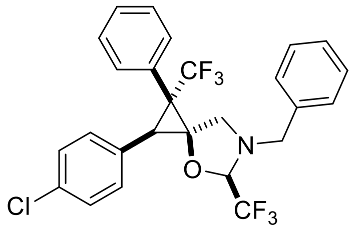

Following general procedure C, starting from **3j** (150 mg, 0.495 mmol, 1.00 equiv.), the crude product was purified by flash column chromatography (SiO_2, 12 g, 3 – 12% EtOAc in pentane) to afford **7j** as a colourless amorphous foam (165 mg, 0.322 mmol, 65%).

TLC: R_f = 0.65 (SiO_2, 5% EtOAc in pentane).

^{1}H NMR (400 MHz, $CDCl_3$) δ 7.36 – 7.20 (m, 8H, Ar*H*), 7.15 – 7.11 (m, 2H, Ar*H*), 7.10 – 7.01 (m, 2H, Ar*H*), 6.90 – 6.83 (m, 2H, Ar*H*), 4.72 (q, *J* = 5.6 Hz, 1H, C*H*CF_3), 4.01 (d, *J* = 13.2 Hz, 1H, C*H*$_2$), 3.87 (d, *J* = 13.2 Hz, 1H, C*H*$_2$), 3.78 (d, *J* = 13.2 Hz, 1H, C*H*$_2$), 3.30 – 3.23 (m, 1H, C*H*$_2$), 2.76 (s, 1H, C*H*).

^{13}C NMR (101 MHz, $CDCl_3$) δ 136.9, 133.8, 133.0, 131.5, 131.4, 128.9, 128.8, 128.8, 128.2, 128.0, 127.9, 126.9, 125.2 (q, J_{C-F} = 275.4 Hz), 123.0 (q, J_{C-F} = 283.4 Hz), 93.0 (q, J_{C-F} = 34.4 Hz), 69.7 (q, J_{C-F} = 1.8 Hz), 60.5, 56.8, 36.5 (q, J_{C-F} = 32.4 Hz), 34.3 (q, J_{C-F} = 2.8 Hz).

^{19}F NMR (376 MHz, $CDCl_3$) δ -66.7, -79.5 (d, *J* = 5.5 Hz).

HRMS (ESI/QTOF) m/z: [M + H]$^+$ Calcd for $C_{26}H_{21}ClF_6NO^+$ 512.1210; Found 512.1221.

IR (v_{max}, cm^{-1}) 3064 (w), 3031 (w), 2842 (w), 1496 (m), 1294 (m), 1217 (s), 1179 (s), 1141 (s), 1004 (m), 934 (m).

Methyl 6-(4-methoxybenzyl)-2-(4-(methoxycarbonyl)phenyl)-1-phenyl-5-(trifluoromethyl)-4-oxa-6-azaspiro[2.4]heptane-1-carboxylate (7k)

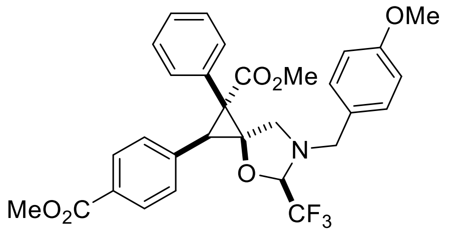

Following general procedure C, starting from **3k** (65 mg, 0.20 mmol, 1.0 equiv.), the crude product was purified by flash column chromatography (SiO_2, 12 g, 5 – 20% Et_2O in pentane), to afford **7k** as an off-white solid (64 mg, 0.12 mmol, 58%).

Melting Point: 137 – 139 °C.

TLC: R_f = 0.32 (SiO_2, 20% Et_2O in pentane).

^{1}H NMR (400 MHz, $CDCl_3$) δ 7.82 – 7.75 (m, 2H, Ar*H*), 7.25 – 7.16 (m, 3H, Ar*H*), 7.16 – 7.10 (m, 2H, Ar*H*), 7.02 – 6.95 (m, 4H, Ar*H*), 6.80 – 6.75 (m, 2H, Ar*H*), 4.79 (q, *J* = 5.7 Hz, 1H, C*H*CF_3), 3.88 (s, 3H, CO_2CH_3), 3.84 (d, *J* = 13.0 Hz, 1H, C*H*$_2$), 3.81 – 3.76 (m, 1H, C*H*$_2$), 3.75 (s, 3H, ArOC*H*$_3$), 3.73 (d, *J* = 13.0 Hz, 1H, C*H*$_2$), 3.62 (s, 3H, CO_2CH_3), 3.50 – 3.44 (m, 1H, C*H*$_2$), 3.22 (s, 1H, C*H*).

^{13}C NMR (101 MHz, $CDCl_3$) δ 172.4, 167.3, 159.4, 139.6, 132.7, 130.3, 130.2, 130.2, 128.9, 128.8, 128.3, 127.8, 127.8, 123.2 (d, $J_{C\text{-}F}$ = 283.6 Hz), 114.1, 93.3 (q, $J_{C\text{-}F}$ = 34.2 Hz), 73.7, 59.9, 57.4, 55.3, 53.2, 52.2, 39.9, 39.8.

^{19}F NMR (376 MHz, $CDCl_3$) δ -79.4 (d, *J* = 5.6 Hz).

HRMS (ESI/QTOF) m/z: $[M + Na]^+$ Calcd for $C_{30}H_{28}F_3NNaO_6^+$ 578.1761; Found 578.1771.

IR (ν_{max}, cm^{-1}) 3031 (w), 2952 (w), 2839 (w), 1718 (s), 1611 (m), 1513 (m), 1437 (m), 1283 (s), 1253 (s), 1175 (s), 1147 (s), 1121 (s), 992 (m), 862 (m).

Methyl 2-(4-(methoxycarbonyl)phenyl)-6-methyl-1-phenyl-5-(trifluoromethyl)-4-oxa-6-azaspiro[2.4]heptane-1-carboxylate (7l)

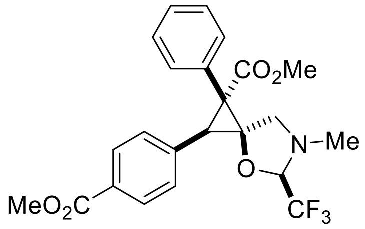

Following general procedure C, starting from **3l** (43 mg, 0.20 mmol, 1.0 equiv.), the crude product was purified by flash column chromatography (SiO_2, 12 g, 15 – 45% Et_2O in pentane), to afford **7l** as an off-white solid (59 mg, 0.13 mmol, 66%).

Melting Point: 101 – 102 °C.

TLC: R_f = 0.31 (SiO_2, 30% Et_2O in pentane).

^{1}H NMR (400 MHz, $CDCl_3$) δ 7.82 – 7.69 (m, 2H, Ar*H*), 7.30 – 7.16 (m, 3H, Ar*H*), 7.03 – 6.94 (m, 4H, Ar*H*), 4.59 (q, *J* = 5.5 Hz, 1H, C*H*CF_3), 3.87 (s, 3H, CO_2CH_3), 3.82 (d, *J* = 1.4 Hz, 1H, C*H*$_2$), 3.64 (s, 3H, CO_2CH_3), 3.37 – 3.30 (m, 1H, C*H*$_2$), 3.29 (s, 1H, C*H*), 2.57 (s, 3H, NC*H*$_3$).

^{13}C NMR (101 MHz, $CDCl_3$) δ 172.4, 167.2, 139.4, 132.8, 130.2, 128.8, 128.4, 127.9, 127.8, 123.0 (q, $J_{C\text{-}F}$ = 283.7 Hz), 95.4 (q, $J_{C\text{-}F}$ = 34.1 Hz), 73.4, 59.7, 53.3, 52.2, 44.4, 39.8, 39.5. One carbon is not resolved.

^{19}F NMR (376 MHz, $CDCl_3$) δ -79.9 (d, *J* = 5.6 Hz).

HRMS (ESI/QTOF) m/z: $[M + Na]^+$ Calcd for $C_{23}H_{22}F_3NNaO_5^+$ 472.1342; Found 472.1347.

IR (ν_{max}, cm^{-1}) 3059 (w), 2987 (m), 2968 (m), 2902 (m), 1717 (s), 1610 (w), 1436 (m), 1281 (s), 1254 (s), 1228 (s), 1148 (s), 1069 (s), 860 (m), 770 (s).

Ethyl 6-benzyl-2-(4-(methoxycarbonyl)phenyl)-1-methyl-5-(trifluoromethyl)-4-oxa-6-azaspiro[2.4]heptane-1-carboxylate (7m)

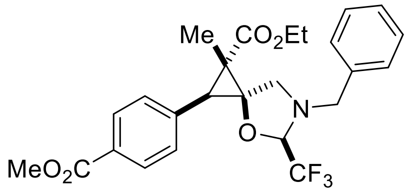

Following general procedure C, starting from **3m** (46 mg, 0.20 mmol, 1.0 equiv.), the crude product was purified by flash column chromatography (SiO_2, 12 g, 5 – 15% Et_2O in pentane) to afford **7m** as beige solid (62 mg, 0.12 mmol, 58%).

Melting Point: 100 – 102 °C.

TLC: R_f = 0.27 (SiO_2, 5% EtOAc in pentane).

^{1}H NMR (400 MHz, $CDCl_3$) δ 8.02 – 7.95 (m, 2H, Ar*H*), 7.40 – 7.28 (m, 7H, Ar*H*), 4.94 (q, *J* = 5.6 Hz, 1H, C*H*CF$_3$), 4.18 (dddd, *J* = 17.9, 10.7, 7.1, 3.6 Hz, 2H, C*H*$_2$CH$_3$), 4.10 (d, *J* = 13.2 Hz, 1H, C*H*$_2$), 4.03 (d, *J* = 13.2 Hz, 1H, C*H*$_2$), 3.92 (s, 3H, CO$_2$C*H*$_3$), 3.60 – 3.51 (m, 1H, C*H*$_2$), 3.34 – 3.24 (m, 1H, C*H*$_2$), 2.97 (s, 1H, C*H*), 1.27 (t, *J* = 7.1 Hz, 3H, CH$_2$C*H*$_3$), 1.16 (s, 3H, C-C*H*$_3$).

^{13}C NMR (101 MHz, $CDCl_3$) δ 172.8, 167.1, 139.5, 137.3, 130.4, 129.5, 128.8, 128.8, 128.8, 128.0, 123.2 (q, $J_{C\text{-}F}$ = 285.0 Hz), 92.6 (q, $J_{C\text{-}F}$ = 34.1 Hz), 73.8, 61.4, 60.7, 56.4, 52.2, 34.2, 32.3, 14.4, 9.9.

^{19}F NMR (376 MHz, $CDCl_3$) δ -79.8 (d, *J* = 5.7 Hz).

HRMS (ESI/QTOF) m/z: $[M + Na]^+$ Calcd for $C_{25}H_{26}F_3NNaO_5^+$ 500.1655; Found 500.1663.

IR (ν_{max}, cm^{-1}) 3075 (w), 2985 (w), 2902 (w), 2841 (w), 1717 (s), 1611 (w), 1440 (m), 1279 (s), 1175 (s), 1115 (s), 1022 (m), 867 (m).

Methyl 4-(-6-benzyl-2-(((*tert*-butyldimethylsilyl)oxy)methyl)-2-phenyl-5-(trifluoromethyl)-4-oxa-6-azaspiro[2.4]heptan-1-yl)benzoate (7n)

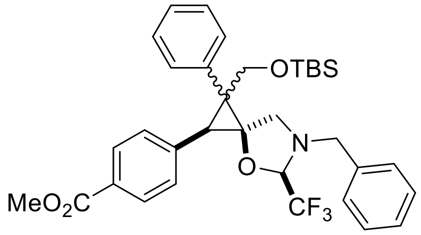

Following general procedure C, starting from **3o** (76 mg, 0.20 mmol, 1.0 equiv.), the crude product was purified by flash column chromatography (SiO_2, 12 g, 2 – 10% EtOAc in pentane) to afford **7n** as a colorless amorphous solid and as an inseparable mixture of diastereomers (70 mg, 0.13 mmol, 63%, 5:3 d.r.). The d.r. was determined by comparison of the integrals of silyl group $Si(CH_3)_2$ signal.

TLC: R_f = 0.70 (SiO_2, 10% EtOAc in pentane).

^{1}H NMR (400 MHz, $CDCl_3$) δ 8.06 – 8.01 (m, 2H minor, Ar*H*), 7.83 – 7.78 (m, 2H major, Ar*H*), 7.74 – 7.66 (m, 2H minor, Ar*H*), 7.40 – 7.27 (m, 4H major, 10H minor, Ar*H*), 7.25 – 7.20 (m, 4H major, Ar*H*), 7.08 – 7.02 (m, 2H major, Ar*H*), 7.00 – 6.94 (m, 2H major, Ar*H*), 4.95 (q, *J* = 5.1 Hz, 1H minor, C*H*CF$_3$), 4.63 (q, *J* = 5.5 Hz, 1H major, C*H*CF$_3$), 4.20 (d, *J* = 13.2 Hz, 1H minor, C*H*$_2$), 3.95 (s, 3H minor, CO$_2$C*H*$_3$), 3.92 (d, *J* = 8.3 Hz, 1H major, C*H*$_2$), 3.91 – 3.88 (m, 3H major, 2H minor CO$_2$C*H*$_3$, C*H*$_2$), 3.88 – 3.85 (m, 1H major, C*H*$_2$), 3.81 (d, *J* = 3.4 Hz, 2H major OC*H*$_2$),

3.71 (d, J = 10.9 Hz, 1H minor, CH_2), 3.64 (d, J = 10.5 Hz, 1H major, CH_2), 3.30 (d, J = 11.3 Hz, 1H minor, CH_2), 3.24 (d, J = 13.1 Hz, 1H major, CH_2), 2.69 (d, J = 11.3 Hz, 1H minor, CH_2), 2.58 (s, 1H minor, CH), 2.54 (s, 1H major, CH), 0.82 (s, 9H major, SiC(CH_3)$_3$), 0.68 (s, 9H minor, SiC(CH_3)$_3$), -0.11 (d, J = 38.6 Hz, 6H major Si(CH_3)$_2$), -0.35 (d, J = 38.1 Hz, 6H minor, Si(CH_3)$_2$).

^{13}C NMR (101 MHz, CDCl$_3$) δ 167.5 (major), 167.3 (minor), 142.0 (major), 140.9 (minor), 140.3 (minor), 137.5 (major), 137.5 (minor), 134.2 (major), 132.2 (major), 130.9 (major), 129.9 (minor), 129.7 (major), 129.3 (minor), 128.9 (major), 128.7 (major), 128.7 (major), 128.7 (minor), 128.5 (minor), 128.5 (minor), 128.3 (minor), 127.9 (major), 127.9 (major), 127.3 (minor), 127.2 (major), 127.0 (minor), 123.5 (q, J_{C-F} = 284.1 Hz, minor), 123.3 (q, J_{C-F} = 283.4 Hz, major), 92.5 (q, J_{C-F} = 33.8 Hz, major), 92.5 (q, J_{C-F} = 33.7 Hz, minor), 72.2 (minor), 71.6 (major), 68.2 (major), 63.0 (minor), 60.4 (major), 59.9 (minor), 56.1 (minor), 55.9 (major), 52.2 (minor), 52.0 (major), 39.1 (minor), 37.8 (major), 34.4 (major), 33.4 (minor), 25.9 (major), 25.7 (minor), 18.2 (major), 18.1 (minor), -5.7 (2 x CH$_3$Si, major), -6.0 (2 x CH3Si, minor). 1C of minor diastereomer not resolved.

^{19}F NMR (376 MHz, CDCl$_3$) δ -79.83 (d, J = 5.5 Hz, major), -80.11 (d, J = 5.2 Hz, minor).

IR (ν_{max}, cm^{-1}) 3060 (w), 3028 (w), 2954 (m), 2898 (m), 2858 (m), 1722 (s), 1610 (w), 1497 (w), 1467 (w), 1439 (w), 1281 (s), 1174 (s), 1154 (s), 1110 (s), 1023 (m), 840 (s).

HRMS (ESI/QTOF) m/z: [M + Na]$^+$ Calcd for $C_{34}H_{40}F_3NNaO_4Si^+$ 634.2571; Found 634.2570.

Methyl 6-benzyl-1-phenyl-5-(trifluoromethyl)-2-((triisopropylsilyl)ethynyl)-4-oxa-6-azaspiro[2.4]heptane-1-carboxylate (9a)

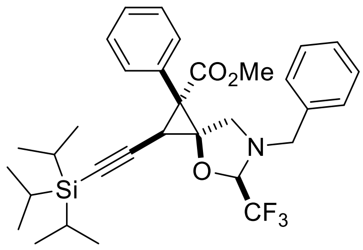

Following a modified version of general procedure C, starting from **3a** (59 mg, 0.20 mmol, 1.0 equiv.) using (bromoethynyl)triisopropylsilane (72 µL, 0.30 mmol, 1.5 equiv.), Cs_2CO_3, (85 mg, 0.26 mmol, 1.3 equiv.), BrettPhos Pd G3 (7.7 mg, 8.0 µmol, 4.0 mol%) and BrettPhos (8.6 mg, 16 µmol, 8.0 mol%). The crude product was purified by flash column chromatography (SiO_2, 12 g, 3 – 12% Et_2O in pentane) to afford **9a** as a colourless amorphous foam (45 mg, 0.079 mmol, 39%).

TLC: R_f = 0.44 (SiO_2, 5% Et_2O in pentane).

^{1}H NMR (400 MHz, CDCl$_3$) δ 7.42 – 7.37 (m, 4H, ArH), 7.37 – 7.23 (m, 6H, ArH), 4.77 (q, J = 5.6 Hz, 1H, CHCF$_3$), 4.09 (d, J = 13.1 Hz, 1H, CH_2), 4.03 (d, J = 13.1 Hz, 1H, CH_2), 3.77 – 3.70 (m, 1H, CH_2), 3.60 (s, 3H, CO$_2$CH_3), 3.39 – 3.31 (m, 1H, CH_2), 2.78 (s, 1H, CH), 1.01 – 0.86 (m, 21H, Si-(CH-(CH_3)$_2$)$_3$).

^{13}C NMR (101 MHz, CDCl$_3$) δ 171.3, 137.2, 132.1, 131.2, 129.0, 128.8, 128.0, 127.7, 127.6, 123.1 (q, J_{C-F} = 283.3 Hz), 100.4, 92.9 (q, J_{C-F} = 34.2 Hz), 88.0, 72.5, 60.7, 56.6, 56.6, 53.2, 38.1, 27.7, 18.6, 18.5, 11.2.

^{19}F NMR (376 MHz, CDCl$_3$) δ -79.7 (d, J = 5.6 Hz).

HRMS (ESI/QTOF) m/z: [M + H]$^+$ Calcd for $C_{32}H_{41}F_3NO_3Si^+$ 572.2802; Found 572.2809.

IR (ν_{max}, cm^{-1}) 3065 (w), 3033 (w), 2944 (m), 2865 (m), 2166 (w), 1722 (s), 1461 (m), 1293 (m), 1254 (s), 1176 (s), 1150 (s).

Methyl 6-benzyl-1-(4-chlorophenyl)-5-(trifluoromethyl)-2-(3,3,3-trifluoroprop-1-en-2-yl)-4-oxa-6-azaspiro[2.4]heptane-1-carboxylate (11a)

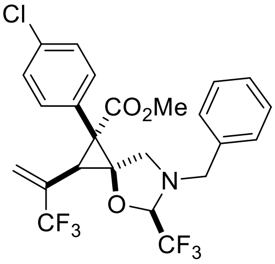

Following general procedure C, starting from **3b** (65 mg, 0.20 mmol, 1.0 equiv.), the crude product was purified by flash column chromatography (SiO_2, 12 g, 5 – 15% Et_2O in pentane) to afford **11a** as white solid (44 mg, 0.084 mmol, 42%).

Melting Point: 81 – 82 °C.

TLC: R_f = 0.61 (SiO_2, 10% Et_2O in pentane).

^{1}H NMR (400 MHz, $CDCl_3$) δ 7.40 – 7.33 (m, 4H, Ar*H*), 7.33 – 7.28 (m, 3H, Ar*H*), 7.14 – 7.08 (m, 2H, Ar*H*), 5.68 – 5.61 (m, 1H, C=C*H*$_2$), 4.83 (q, *J* = 5.6 Hz, 1H, C*H*CF_3), 4.68 (q, *J* = 1.5 Hz, 1H, C=C*H*$_2$), 3.98 (d, *J* = 13.0 Hz, 1H, C*H*$_2$), 3.91 (d, *J* = 13.0 Hz, 1H, C*H*$_2$), 3.75 – 3.68 (m, 1H, C*H*$_2$), 3.62 (s, 3H, CO_2C*H*$_3$), 3.43 – 3.35 (m, 1H, C*H*$_2$), 2.79 (s, 1H, C*H*).

^{13}C NMR (101 MHz, $CDCl_3$) δ 171.4, 136.8, 134.1, 134.0, 129.7 (q, J_{C-F} = 29.7 Hz), 128.9, 128.9, 128.4, 128.2, 128.1, 123.6 (q, J_{C-F} = 274.4 Hz), 123.0 (q, J_{C-F} = 283.4 Hz), 122.1 (q, J_{C-F} = 4.7 Hz), 93.8 (q, J_{C-F} = 34.4 Hz), 72.7, 60.6, 57.1, 53.4, 38.0, 31.7.

^{19}F NMR (376 MHz, $CDCl_3$) δ -68.8 (s), -79.4 (d, *J* = 5.5 Hz).

HRMS (ESI/QTOF) m/z: $[M + H]^+$ Calcd for $C_{24}H_{21}ClF_6NO_3^+$ 520.1109; Found 520.1114.

IR (ν_{max}, cm^{-1}) 3066 (w), 3033 (w), 2956 (w), 2851 (w), 1725 (m), 1496 (w), 1293 (m), 1238 (m), 1173 (s), 1123 (s), 988 (m), 740 (m).

Methyl 6-benzyl-1-(4-(methoxycarbonyl)phenyl)-5-(trifluoromethyl)-2-(3,3,3-trifluoroprop-1-en-2-yl)-4-oxa-6-azaspiro[2.4]heptane-1-carboxylate (11b)

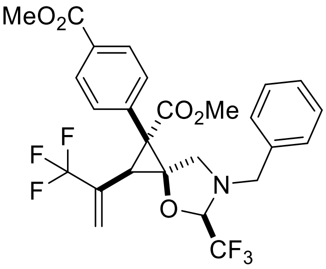

Following general procedure C, starting from **3d** (328 mg, 0.933 mmol, 1.00 equiv.), the crude product was purified by flash column chromatography (SiO_2, 12 g, 3 – 15% Et_2O in pentane), afford **11b** as a white solid (164 mg, 0.302 mmol, 32%).

TLC: R_f = 0.36 (SiO_2, 10% Et_2O in pentane).

^1H NMR (400 MHz, $CDCl_3$) δ 8.03 – 7.97 (m, 2H, Ar*H*), 7.44 – 7.28 (m, 5H, Ar*H*), 7.28 – 7.21 (m, 2H, Ar*H*), 5.65 – 5.54 (m, 1H, C=CH_2), 4.82 (q, *J* = 5.6 Hz, 1H, C*H*CF_3), 4.65 – 4.55 (m, 1H, C=CH_2), 3.97 (d, *J* = 13.0 Hz, 1H, CH_2), 3.93 – 3.86 (m, 4H CH_2, ArCO_2CH_3), 3.77 – 3.68 (m, 1H CH_2), 3.60 (s, 3H, CO_2CH_3), 3.45 – 3.36 (m, 1H, CH_2), 2.81 (s, 1H, C*H*).

^{13}C NMR (101 MHz, $CDCl_3$) δ 171.1, 167.0, 136.8, 134.8, 132.7, 129.8, 129.7 (q, $J_{C\text{-}F}$ = 29.8 Hz), 129.3, 128.9, 128.9, 128.2, 123.5 (q, $J_{C\text{-}F}$ = 273.5 Hz), 123.0 (q, $J_{C\text{-}F}$ = 283.3 Hz), 122.1 (q, $J_{C\text{-}F}$ = 5.6 Hz), 93.8 (q, $J_{C\text{-}F}$ = 34.5 Hz), 72.7, 60.6, 57.2, 53.4, 52.3, 38.5, 31.8.

^{19}F NMR (376 MHz, $CDCl_3$) δ -68.8, -79.4 (d, *J* = 5.5 Hz).

HRMS (ESI/QTOF) m/z: $[M + H]^+$ Calcd for $C_{26}H_{24}F_6NO_5^+$ 544.1553; Found 544.1559.

IR (ν_{max}, cm^{-1}) 3037 (w), 2955 (w), 2852 (w), 1724 (s), 1607 (w), 1438 (m), 1282 (s), 1177 (s), 1124 (s), 992 (m).

Product Modifications

Methyl 5-(benzyl(*tert*-butoxycarbonyl)amino)-3-(4-chlorophenyl)-4-oxo-2-phenylpentanoate (12)

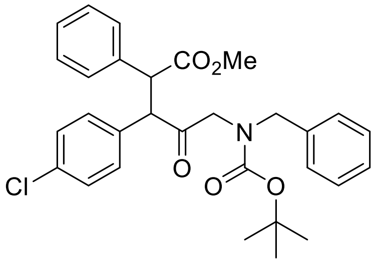

To a vial containing tethered product **6g** (50 mg, 0.10 mmol, 1.0 equiv.) was added HFIP (1.0 mL) and trifluoroacetic acid (31 µL, 0.40 mmol, 4.0 equiv.). The reaction is stirred at room temperature for 16 h, then concentrated *in vacuo*, The crude mixture is then dissolved in DCM (1 mL), cooled to 0 °C, and Et_3N (70 µL, 0.50 mmol, 5.0 equiv.) and Boc_2O (65 mg, 0.3 mmol, 3.0 equiv.) are added sequentially. The reaction was then stirred at room temperature for 2 h, then quenched with water (3 mL) and extracted with DCM (3 x 3 mL). The combined organic fractions were combined and dried over anhydrous Na_2SO_4, filtered and concentrated *in vacuo*. The crude residue was then purified via preparatory thin layer chromatography (SiO_2, 50% Et_2O in pentane) to afford **12** as an amorphous solid and as an inseparable mixture of diastereoisomers (38 mg, 0.073 mmol, 73%, 2:1 d.r.). The d.r. was determined by comparison of the integrals of Boc group C(CH_3)$_3$ signal.

TLC: R_f = 0.50 (SiO_2, 30% Et_2O in pentane).

^{1}H NMR (400 MHz, $CDCl_3$) δ 7.48 – 7.40 (m, 2H major, 2H minor, Ar*H*), 7.37 – 7.28 (m, 7H major, 7H minor, Ar*H*), 7.22 – 7.14 (m, 3H major, 3H minor, Ar*H*), 6.80 – 6.70 (m, 2H major, 2H minor, Ar*H*), 4.55 (d, *J* = 11.7 Hz, 1H minor, ArC*H*CO), 4.46 – 4.35 (m, 2H major, 1H minor, ArC*H*CO), 4.26 (d, *J* = 14.9 Hz, 1H major, CH_2), 3.91 (d, *J* = 15.6 Hz, 1H minor), 3.84 – 3.75 (m, 1H major, 1H minor, CH_2), 3.75 – 3.67 (m, 1H major, 1H minor, CH_2), 3.66 – 3.58 (m, 1H minor, CH_2), 3.41 (s, 3H major, 3H minor, CO_2CH_3) 3.32 (d, *J* = 18.7 Hz, 1H major, CH_2), 1.42 (s, 9H minor, C(CH_3)$_3$), 1.10 (s, 9H major, C(CH_3)$_3$)

^{13}C NMR (101 MHz, $CDCl_3$) δ 203.5 (minor), 202.5 (major), 171.9 (minor), 171.9 (major), 155.4 (major), 155.3 (minor), 137.1 (minor), 136.9 (major), 136.2 (minor), 136.2 (major), 134.2 (major), 134.1 (minor), 133.8 (major), 133.7 (minor), 130.2 (minor), 130.1 (major), 129.2 (major), 128.9 (major), 128.9 (minor), 128.6 (minor), 128.6 (major), 128.4 (major), 128.3 (minor), 128.1 (major), 128.0 (minor), 127.5 (minor), 127.4 (major), 127.3 (minor), 80.6 (minor), 80.2 (major), 57.7 (major), 57.7 (minor), 55.4 (major), 54.8 (minor), 54.2 (minor), 54.1 (major), 52.1 (major), 52.1 (minor), 50.3 (minor), 50.2 (major), 28.3 (minor), 27.8 (major). 2C not resolved.

HRMS (ESI/QTOF) m/z: $[M + Na]^+$ Calcd for $C_{30}H_{32}ClNNaO_5^+$ 544.1861; Found 544.1844.

IR (v_{max}, cm^{-1}) 2975 (m), 1730 (s), 1697 (s), 1491 (m), 1453 (m), 1429 (m), 1392 (m), 1366 (m), 1238 (s), 1159 (s), 912 (m).

Methyl 5-(*N*-benzylacetamido)-3-(4-chlorophenyl)-4-oxo-2-phenylpentanoate (13)

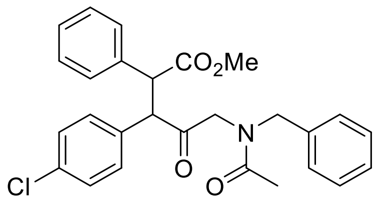

To a vial containing tethered product **6g** (50 mg, 0.10 mmol, 1.0 equiv.) was added HFIP (1.0 mL) and trifluoroacetic acid (31 µL, 0.40 mmol, 4.0 equiv.). The reaction is stirred at room temperature for 16 h, then concentrated *in vacuo*, The crude mixture is then dissolved in DCM (1 mL), cooled to 0 °C, and Et_3N (42 µL, 0.30 mmol, 3.0 equiv.) and AcCl (14 µL, 0.2 mmol, 2.0 equiv.) are added sequentially. The reaction was then stirred at room temperature for 1 h, then quenched with water (3 mL) and extracted with DCM (3 x 3 mL). The combined organic fractions were combined and dried over anhydrous Na_2SO_4, filtered and concentrated *in vacuo*. The crude residue was then purified via preparatory thin layer chromatography (SiO_2, 50% Et_2O in pentane) to afford **13** as a white amorphous solid and as an inseparable mixture of diastereoisomers (24 mg, 0.052 mmol, 52%, 1.4:1 d.r.). The d.r. was determined by comparison of the integrals of Ac group COCH_3 signal.

TLC: R_f = 0.44 (SiO_2, 50% Et_2O in pentane)

^{1}H NMR (400 MHz, $CDCl_3$) δ 7.47 – 7.40 (m, 2H major, 2H minor, Ar*H*), 7.37 – 7.26 (m, 7H major, 7H minor, Ar*H*), 7.25 – 7.09 (m, 3H major, 3H minor, Ar*H*), 6.82 – 6.76 (m, 2H major, Ar*H*), 6.76 – 6.72 (m, 2H minor, Ar*H*), 4.51 (d, *J* = 11.7 Hz, 1H major, ArC*H*CO), 4.42 – 4.37 (m, 1H major, 2H minor, ArC*H*CO), 4.23 – 4.12 (m, 2H minor, C*H*$_2$), 4.05 (d, *J* = 7.1 Hz, 1H major, C*H*$_2$), 4.01 (d, *J* = 8.0 Hz, 1H major, C*H*$_2$), 3.84 (d, *J* = 13.1 Hz, 1H major, C*H*$_2$), 3.79 (d, *J* = 16.0 Hz, 1H minor, C*H*$_2$), 3.73 (d, *J* = 17.4 Hz, 1H major, C*H*$_2$), 3.56 (d, *J* = 19.4 Hz, 1H minor, C*H*$_2$), 3.43 (s, 3H minor, CO_2C*H*$_3$), 3.41 (s, 3H major, CO_2C*H*$_3$), 2.08 (s, 3H major, NHCOC*H*$_3$), 1.35 (s, 3H minor, CO_2C*H*$_3$).

^{13}C NMR (101 MHz, $CDCl_3$) δ 202.6 (major), 202.2 (minor), 171.9 (major), 171.8 (minor), 171.0 (minor), 170.9 (major), 136.4 (minor), 136.3 (major), 136.1 (minor), 135.7 (major), 134.6 (minor), 134.3 (major), 133.7 (major), 133.1 (minor), 130.3 (major), 130.1 (minor), 129.5 (minor), 129.3 (major), 129.2 (minor), 129.0 (major), 128.9 (major), 128.7 (major 2C), 128.6 (minor 2C), 128.5 (minor), 128.2 (minor), 127.9 (major), 127.7 (minor), 126.7 (major), 58.2 (minor), 58.1 (major), 57.0 (minor), 54.3 (major), 54.2 (major), 54.1 (minor), 52.3 (minor), 52.2 (major), 52.0 (major), 49.2 (minor), 21.2 (major), 20.6 (minor).

HRMS (Sicrit plasma/LTQ-Orbitrap) m/z: $[M + H]^+$ Calcd for $C_{27}H_{27}ClNO_4^+$ 464.1623; Found 464.1620.

IR (v_{max}, cm^{-1}) 3030 (w), 2953 (w), 1729 (s), 1650 (s), 1491 (m), 1431 (s), 1283 (s), 1234 (s), 1159 (s), 1092 (m).

6-Benzyl-1-(4-chlorophenyl)-5-(trifluoromethyl)-2-(4-(trifluoromethyl)phenyl)-4-oxa-6-azaspiro[2.4]heptan-1-yl)methanol (14)

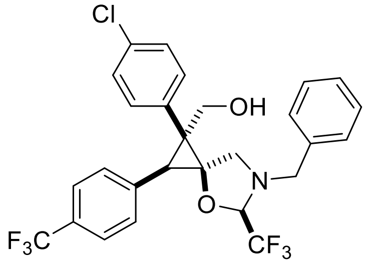

Product **7aa** (114 mg, 0.200 mmol, 1.00 equiv.) was added to a dried flask containing anhydrous THF (2 mL) and cooled to 0 °C. A solution of $LiAlH_4$ in THF (1 M, 0.40 mL, 0.40 mmol, 2.0 equiv.) was then added to the flask dropwise, and the reaction mixture was warmed to room temperature and stirred for 2 hours. The reaction mixture was then cooled to 0 °C and water was added slowly dropwise, followed by the addition of EtOAc (10 mL) to destroy excess $LiAlH_4$. Water (1 mL) was then added to the solution and a white precipitate formed. The solution was decanted into a flask, and the precipitate was washed with Et_2O (3 x 8 mL). The combined organic fractions were dried over anhydrous Na_2SO_4, filtered and concentrated *in vacuo*. The crude residue was then purified by flash column chromatography (SiO_2, 4 g, 10 - 35% Et_2O in pentane) to afford **14** as a white solid (90 mg, 0.17 mmol, 83%).

Melting Point: 136 – 138 °C.

TLC: R_f = 0.143 (SiO_2, 20% Et_2O in pentane).

^{1}H NMR (400 MHz, $CDCl_3$) δ 7.40 (d, *J* = 8.3 Hz, 2H, Ar*H*), 7.34 – 7.26 (m, 5H, Ar*H*), 7.26 – 7.23 (m, 2H, Ar*H*), 7.03 – 6.94 (m, 4H, Ar*H*), 4.68 (q, *J* = 5.5 Hz, 1H, C*H*CF_3), 4.00 (d, *J* = 12.6 Hz, 1H, C*H*$_2$), 3.90 – 3.76 (m, 3H, C*H*$_2$OH, C*H*$_2$), 3.56 (dd, *J* = 11.6, 4.5 Hz, 1H, C*H*$_2$), 3.21 – 3.07 (m, 1H, C*H*$_2$), 2.37 (s, 1H, C*H*), 1.52 (br s, 1H, OH).

^{13}C NMR (101 MHz, $CDCl_3$) δ 139.5, 137.2, 133.8, 133.4, 131.8, 129.8, 128.8, 128.8, 128.7, 128.1 (q, J_{C-F} = 32.5 Hz), 128.0, 124.5 (q, J_{C-F} = 3.8 Hz), 124.5 (d, J_{C-F} = 272.0 Hz), 123.2 (d, J_{C-F} = 283.5 Hz), 93.0 (q, J_{C-F} = 34.0 Hz), 71.9, 69.6, 60.4, 55.9, 37.0, 36.4.

^{19}F NMR (376 MHz, $CDCl_3$) δ -62.3 (s, 1F), -79.8 (d, *J* = 5.4 Hz, 1F).

HRMS (ESI/QTOF) m/z: $[M + H]^+$ Calcd for $C_{27}H_{23}ClF_6NO_2^+$ 542.1316; Found 542.1324.

IR (v_{max}, cm^{-1}) 3380 (w), 3063 (w), 3031 (w), 2946 (w), 2885 (w), 1618 (w), 1494 (m), 1327 (s), 1292 (m), 1169 (s), 1118 (s), 1016 (m), 844 (m), 735 (m).

Methyl (-6-benzyl-1-(4-chlorophenyl)-5-(trifluoromethyl)-2-(4-(trifluoromethyl)phenyl)-4-oxa-6-azaspiro[2.4]heptane-1-carbonyl)glycinate (15)

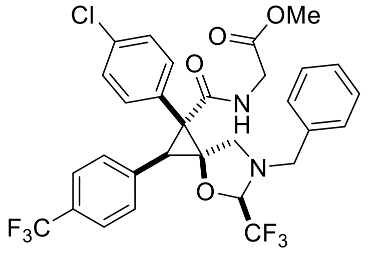

Product **7aa** (57 mg, 0.10 mmol, 1.0 equiv.) was dissolved in a mixture of THF/water (9:1, 1 mL) containing LiOH (10 mg, 0.42 mmol, 4.2 equiv.). The reaction mixture was stirred for 16 h at room temperature, then diluted with water (3 mL) and acidified with 2 M HCl until pH = 1. The aqueous layer was then extracted with DCM (3 x 15 mL), and the combined organic fractions were dried over anhydrous Na_2SO_4, filtered and concentrated *in vacuo*. The resulting acid was used immediately in the next step without further purification.

The intermediate carboxylic acid (0.1 mmol) was used directly from previous reaction and dissolved in anhydrous DMF (1 mL). HATU (49 mg, 0.13 mmol, 1.3 equiv.) and DIPEA (52 uL, 0.30 mmol, 3.0 equiv.) were added to this solution and stirred at room temperature for 5 minutes before adding glycine methyl ester HCl (16 mg, 0.13 mmol, 1.3 equiv.). The reaction was stirred for 16 h at room temperature, and then diluted with EtOAc (15 mL), and washed with 10% LiCl (3 x 15 mL). The combined organic fractions were dried over anhydrous Na_2SO_4, filtered and concentrated *in vacuo*. The crude residue was then purified by flash column chromatography

(SiO_2, 4 g, 15-35% Et_2O in pentane) to afford **15** as a colourless amorphous foam (51 mg, 0.081 mmol, 81%).

TLC: R_f = 0.14 (SiO_2, 20% Et_2O in pentane).

^{1}H NMR (400 MHz, $CDCl_3$) δ 7.41 (d, *J* = 8.2 Hz, 2H, Ar*H*), 7.34 – 7.28 (m, 2H, Ar*H*), 7.27 – 7.19 (m, 5H, Ar*H*), 7.04 (dd, *J* = 10.4, 8.1 Hz, 4H, Ar*H*), 5.76 (t, *J* = 5.5 Hz, 1H, CON*H*), 4.81 (q, *J* = 5.7 Hz, 1H, C*H*CF_3), 3.99 – 3.83 (m, 3H, CH_2, CH_2), 3.82 – 3.73 (m, 2H, CH_2, CH_2), 3.69 (s, 3H, CO_2CH_3), 3.57 – 3.49 (m, 1H, CH_2), 3.40 (s, 1H, C*H*).

^{13}C NMR (101 MHz, $CDCl_3$) δ 170.5, 169.8, 138.4, 137.0, 135.1, 134.3, 130.4, 129.3, 128.8, 128.8, 128.8 (q, J_{C-F} = 32.4 Hz), 128.7, 128.0, 124.6 (q, J_{C-F} = 3.6 Hz), 124.4 (d, J_{C-F} = 271.8 Hz), 123.2 (q, J_{C-F} = 283.9 Hz), 73.5, 60.6, 57.1, 52.5, 42.0, 40.1, 38.2.

^{19}F NMR (376 MHz, $CDCl_3$) δ -62.4, -79.3 (d, *J* = 6.0 Hz).

HRMS (ESI/QTOF) m/z: $[M + Na]^+$ Calcd for $C_{30}H_{25}ClF_6N_2NaO_4^+$ 649.1299; Found 649.1305.

IR (ν_{max}, cm^{-1}) 3652 (w), 3426 (w), 3066 (w), 2974 (m), 2903 (m), 1751 (m), 1665 (m), 1511 (m), 1497 (m), 1326 (s), 1218 (m), 1171 (s), 1120 (s), 768 (s).

Methyl 1-(4-chlorophenyl)-5-(trifluoromethyl)-2-(4-(trifluoromethyl)phenyl)-4-oxa-6-azaspiro[2.4]heptane-1-carboxylate (16a)

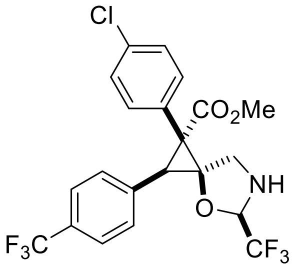

An 8 mL microwave vial equipped with a stirring bar was charged with Pd/C (20 mol%) and **7aa** (57 mg, 0.10 mmol, 1.0 equiv.). The flask was sealed, evacuated and back-filled with N_2 three times. EtOAc (2 mL) was then added and the resulting suspension was stirred at room temperature for 10 minutes under a nitrogen flow. Then, a balloon containing hydrogen was connected to the flask through a needle and the mixture was vigorously stirred at room temperature for 16 h. Then, the reaction mixture was degassed by bubbling nitrogen for 10 minutes and the suspension was filtered through syringe filter and concentrated *in vacuo*. The crude material was then purified by preparatory thin layer chromatography (SiO_2, 10% EtOAc in pentane) to afford **16a** as an off-white powder (42 mg, 0.087 mmol, 87%).

Melting Point: 189 – 191 °C.

TLC: R_f = 0.50 (SiO_2, 15% EtOAc in pentane).

^{1}H NMR (400 MHz, $CDCl_3$) δ 7.38 (d, *J* = 8.1 Hz, 2H, Ar*H*), 7.25 – 7.16 (m, 2H, Ar*H*), 7.03 (d, *J* = 8.1 Hz, 2H, Ar*H*), 6.95 – 6.87 (m, 2H, Ar*H*), 5.05 (q, *J* = 5.6 Hz, 1H, C*H*CF_3), 3.70 (d, *J* = 13.0 Hz, 1H, CH_2), 3.66 (s, 3H, CO_2CH_3), 3.62 (d, *J* = 12.5 Hz, 1H, CH_2), 3.25 (s, 1H, C*H*).

^{13}C NMR (101 MHz, $CDCl_3$) δ 172.0, 138.4, 134.0, 134.0, 130.5, 128.9 (q, J_{C-F} = 32.5 Hz), 128.9, 128.3, 124.6 (q, J_{C-F} = 3.0 Hz), 124.3 (d, J_{C-F} = 271.9 Hz), 121.8 (q, J_{C-F} = 283.3 Hz), 75.0, 53.4, 51.7, 40.0, 39.2.

^{19}F NMR (376 MHz, $CDCl_3$) δ -62.5, -80.4 (d, *J* = 5.6 Hz).

HRMS (APCI/QTOF) m/z: $[M + Na]^+$ Calcd for $C_{21}H_{16}ClF_6NNaO_3^+$ 502.0615; Found 502.0608.

IR (v_{max}, cm^{-1}) 3349 (w), 3033 (w), 2956 (w), 2892 (w), 2847 (w), 1719 (m), 1619 (w), 1495 (m), 1326 (s), 1236 (m), 1168 (s), 1146 (s), 1117 (s), 1071 (s), 911 (m), 736 (s).

Methyl 4-(-2-phenyl-2,5-bis(trifluoromethyl)-4-oxa-6-azaspiro[2.4]heptan-1-yl)benzoate (16b)

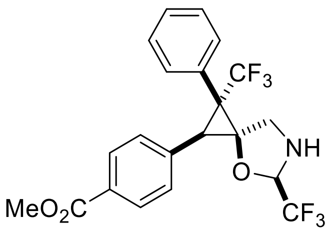

An 8 mL microwave vial equipped with a stirring bar was charged with Pd/C (20 mol%) and **7i** (102 mg, 0.191 mmol, 1.00 equiv.). The flask was sealed, evacuated and back-filled with N_2 three times. EtOAc (2 mL) was then added and the resulting suspension was stirred at room temperature for 10 minutes under a nitrogen flow. Then, a balloon containing hydrogen was connected to the flask through a needle and the mixture was vigorously stirred at room temperature for 16 h. Then, the reaction mixture was degassed by bubbling nitrogen for 10 minutes and the suspension was filtered through syringe filter and concentrated *in vacuo*. The crude material was then purified by preparatory thin layer chromatography (SiO_2, 30% Et_2O in pentane) to afford **16b** as a white solid (72 mg, 0.15 mmol, 85%).

Melting Point: 133 – 135 °C.

TLC: R_f = 0.31 (SiO_2, 30% Et_2O in pentane).

^{1}H NMR (400 MHz, $CDCl_3$) δ 7.81 – 7.74 (m, 2H, Ar*H*), 7.36 – 7.28 (m, 1H, Ar*H*), 7.27 – 7.20 (m, 2H, Ar*H*), 7.11 – 7.02 (m, 2H, Ar*H*), 7.01 – 6.91 (m, 2H, Ar*H*), 5.00 (q, *J* = 5.5 Hz, 1H, C*H*CF_3), 3.91 – 3.79 (m, 4H, CH_2, CO_2CH_3), 3.60 – 3.45 (m, 1H, CH_2), 2.84 (s, 1H, C*H*).

^{13}C NMR (101 MHz, $CDCl_3$) δ 166.9, 138.9, 133.5, 130.1, 128.8, 128.7, 128.5, 127.8, 126.7, 125.1 (q, J_{C-F} = 275.6 Hz), 123.0 (q, J_{C-F} = 283.1 Hz), 88.3 (q, J_{C-F} = 34.7 Hz), 71.1 (q, J_{C-F} = 1.5 Hz), 52.1, 51.0, 38.2 (q, J_{C-F} = 32.4 Hz), 34.4 (q, J_{C-F} = 2.6 Hz).

^{19}F NMR (376 MHz, $CDCl_3$) δ -66.8, -80.7 (d, *J* = 5.5 Hz).

HRMS (Nanochip-based ESI/LTQ-Orbitrap) m/z: $[M + H]^+$ Calcd for $C_{21}H_{18}F_6NO_3^+$ 446.1185; Found 446.1188.

IR (v_{max}, cm^{-1}) 3345 (w), 3062 (w), 3013 (w), 2956 (w), 2893 (w), 2849 (w), 1713 (m), 1611 (w), 1439 (w), 1283 (s), 1140 (s), 1115 (s), 1041 (m), 757 (m).

DFT Calculations

SMILES were generated for all possible diastereomers of compound **6b**. Rough 3D geometries were obtained with Open Babel,[19] followed by a first GFN2-xTB geometry optimization. Conformers for each isomers were generated with Crest also at the GFN2-xTB level.[20,21] Final energies were then computed with DFT using Gaussian16[22] at the ωB97X-D/def2-TZVP//ωB97X-D/def2-SVP level.[23–25]

Table S3. SMILES for all 8 possible diastereomers of cyclopropane **6b**.

Isomer	SMILES
D1	O=C(OC)[C@@]1(C2=CC=CC=C2)[C@H](C3=CC=CC=C3)[C@]14O[C@@H](C(F)(F)F)N(CC5=CC=CC=C5)C4
D2	O=C(OC)[C@]1(C2=CC=CC=C2)[C@H](C3=CC=CC=C3)[C@]14O[C@@H](C(F)(F)F)N(CC5=CC=CC=C5)C4
D3	O=C(OC)[C@@]1(C2=CC=CC=C2)[C@@H](C3=CC=CC=C3)[C@]14O[C@@H](C(F)(F)F)N(CC5=CC=CC=C5)C4
D4	O=C(OC)[C@@]1(C2=CC=CC=C2)[C@H](C3=CC=CC=C3)[C@@]14O[C@@H](C(F)(F)F)N(CC5=CC=CC=C5)C4
D5	O=C(OC)[C@@]1(C2=CC=CC=C2)[C@H](C3=CC=CC=C3)[C@]14O[C@H](C(F)(F)F)N(CC5=CC=CC=C5)C4
D6	O=C(OC)[C@]1(C2=CC=CC=C2)[C@@H](C3=CC=CC=C3)[C@]14O[C@@H](C(F)(F)F)N(CC5=CC=CC=C5)C4
D7	O=C(OC)[C@]1(C2=CC=CC=C2)[C@H](C3=CC=CC=C3)[C@@]14O[C@@H](C(F)(F)F)N(CC5=CC=CC=C5)C4
D8	[C@@]1([C@]2([C@H]1c1ccccc1)O[C@@H](N(C2)Cc1ccccc1)C(F)(F)F)(c1ccccc1)C(=O)OC

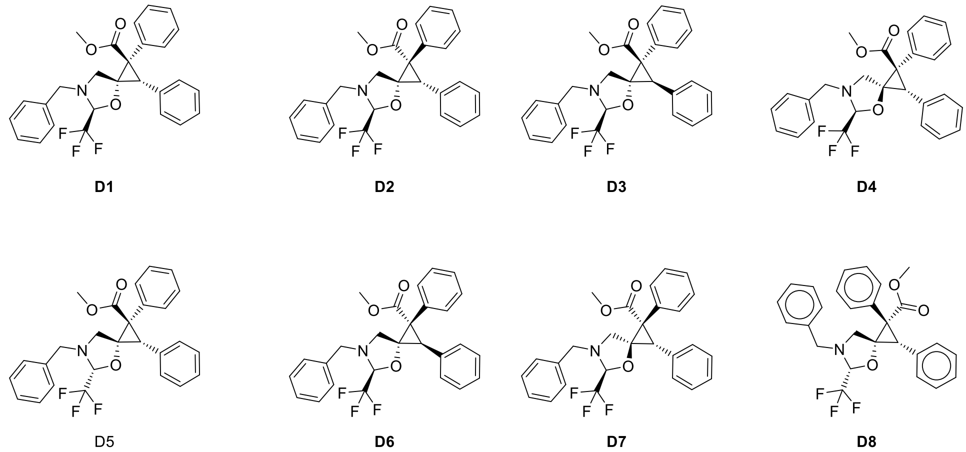

Figure S3. Structures of 8 possible diastereomers of spirocyclopropane **6b**.

Table S4. Gibbs free energies of diastereomers of **6b**. Isomers D1 and D5 are most stable and degenerate in energy.

**Isomer**	**G (Ha)**	**ΔG (kcal/mol)**
D1	-1622.965877	0
D2	-1622.959335	4.105166979
D3	-1622.962443	2.154867534
D4	-1622.962204	2.304842298

D5	-1622.965769	0.067771023
D6	-1622.961471	2.764806743
D7	-1622.960892	3.128134728
D8	-1622.957338	5.358303399

X-ray Crystallographic Data

Methyl 6-benzyl-2-(4-nitrophenyl)-1-phenyl-5-(trifluoromethyl)-4-oxa-6-azaspiro[2.4]heptane-1-carboxylate 6j

CCDC deposition number: 2451792

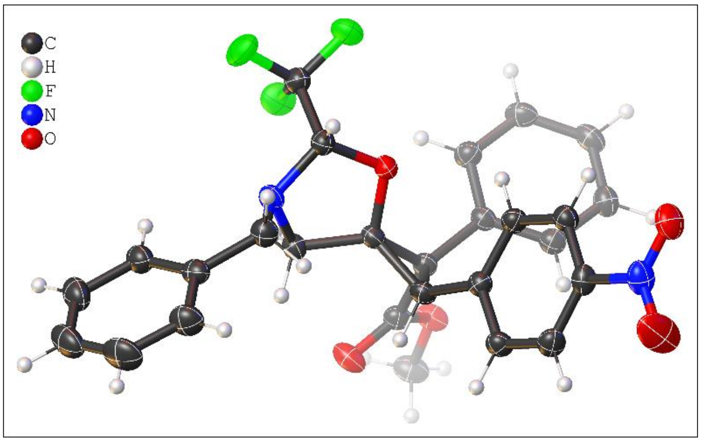

Crystals of compound **6j** were collected upon crystallization via slow evaporation with EtOH in heptane.

Experimental. Single colourless prism-shaped crystals of **6j** were used as supplied. A suitable crystal with dimensions 0.13 × 0.09 × 0.05 mm^3 was selected and mounted on an XtaLAB Synergy R, DW system, HyPix-Arc 150 diffractometer. The crystal was kept at a steady T = 140.00(10) K during data collection. The structure was solved with the ShelXT 2018/2 (Sheldrick, 2015) solution program using dual methods and by using Olex2 1.5 (Dolomanov et al., 2009) as the graphical interface. The model was refined with ShelXL 2019/3 (Sheldrick, 2015) using full matrix least squares minimisation on $\boldsymbol{F}^2$.

Crystal Data. $C_{27}H_{23}N_2O_5F_3$, M_r = 512.47, monoclinic, $C2/c$ (No. 15), a = 25.4150(5) Å, b = 9.44890(15) Å, c = 21.1616(3) Å, β = 110.432(2)°, α = γ = 90°, V = 4762.11(15) Å^3, T = 140.00(10) K, Z = 8, Z' = 1, μ(Cu K$_\alpha$) = 0.975, 23714 reflections measured, 4842 unique (R_{int} = 0.0349) which were used in all calculations. The final wR_2 was 0.1121 (all data) and R_1 was 0.0413 (I≥σ(I)).

Compound	6j
Formula	$C_{27}H_{23}N_2O_5F_3$
$D_{calc.}$/ g cm^{-3}	1.430
μ/mm^{-1}	0.975
Formula Weight	512.47
Colour	colourless
Shape	prism-shaped
Size/mm^3	0.13×0.09×0.05
T/K	140.00(10)
Crystal System	monoclinic
Space Group	$C2/c$
a/Å	25.4150(5)
b/Å	9.44890(15)
c/Å	21.1616(3)
α/°	90
β/°	110.432(2)
γ/°	90
V/Å^3	4762.11(15)
Z	8
Z'	1
Wavelength/Å	1.54184
Radiation type	Cu$K\alpha$
Θ_{min}/°	3.712
Θ_{max}/°	75.535
Measured Refl's.	23714
Indep't Refl's	4842
Refl's I≥2 σ(I)	3952
R_{int}	0.0349
Parameters	335
Restraints	0
Largest Peak/e Å^{-3}	0.320
Deepest Hole/e Å^{-3}	-0.390
GooF	1.050
wR_2 (all data)	0.1121
wR_2	0.1062
R_1 (all data)	0.0514
R_1	0.0413
CCDC number	2451792

Structure Quality Indicators

**Reflections:**	d min (CuKα) 2Θ=151.1° 0.80	I/σ(I) 37.0	Rint m=5.11 3.49%	Full 135.4° 98% to 151.1° 100
**Refinement:**	Shift 0.001	Max Peak 0.3	Min Peak -0.4	GooF 1.050

A colourless prism-shaped crystal with dimensions 0.13 × 0.09 × 0.05 mm^3 was mounted. Data were collected using an XtaLAB Synergy R, DW system, HyPix-Arc 150 diffractometer operating at T = 140.00(10) K.

Data were measured using ω scans with CuK_α radiation. The diffraction pattern was indexed and the total number of runs and images was based on the strategy calculation from the program CrysAlisPro system (CCD 43.94a 64-bit (release 20-10-2023)). The maximum resolution achieved was Θ = 75.535° (0.80 Å).

The unit cell was refined using CrysAlisPro 1.171.43.94a (Rigaku OD, 2023) on 13170 reflections, 56% of the observed reflections.

Data reduction, scaling and absorption corrections were performed using CrysAlisPro 1.171.43.94a (Rigaku OD, 2023). The final completeness is 100.00 % out to 75.535° in Θ. A Gaussian absorption correction was performed using CrysAlisPro 1.171.43.94a (Rigaku Oxford Diffraction, 2024) Numerical absorption correction based on Gaussian integration over a multifaceted crystal model. Empirical absorption correction using spherical harmonics as implemented in SCALE3 ABSPACK scaling algorithm. The absorption coefficient μ of this material is 0.975 mm^{-1} at this wavelength (λ = 1.54184Å) and the minimum and maximum transmissions are 0.904 and 1.000.

The structure was solved and in space group $C2/c$ (# 15) by the ShelXT 2018/2 (Sheldrick, 2015) structure solution program using dual methods and refined by full matrix least squares minimisation on $\boldsymbol{F}^2$ using version 2019/3 of ShelXL 2019/3 (Sheldrick, 2015). All non-hydrogen atoms were refined anisotropically. Hydrogen atom positions were calculated geometrically and refined using the riding model.

There is a single formula unit in the asymmetric unit, which is represented by the reported sum formula. In other words: Z is 8 and Z' is 1. The moiety formula is C27 H23 F3 N2 O5.

Citations

CrysAlis^{Pro} Software System, Rigaku Oxford Diffraction, (2023).

O.V. Dolomanov and L.J. Bourhis and R.J. Gildea and J.A.K. Howard and H. Puschmann, Olex2: A complete structure solution, refinement and analysis program, *J. Appl. Cryst.*, (2009), **42**, 339-341.

Sheldrick, G.M., Crystal structure refinement with ShelXL, *Acta Cryst.*, (2015), **C71**, 3-8.

Sheldrick, G.M., ShelXT-Integrated space-group and crystal-structure determination, *Acta Cryst.*, (2015), **A71**, 3-8.

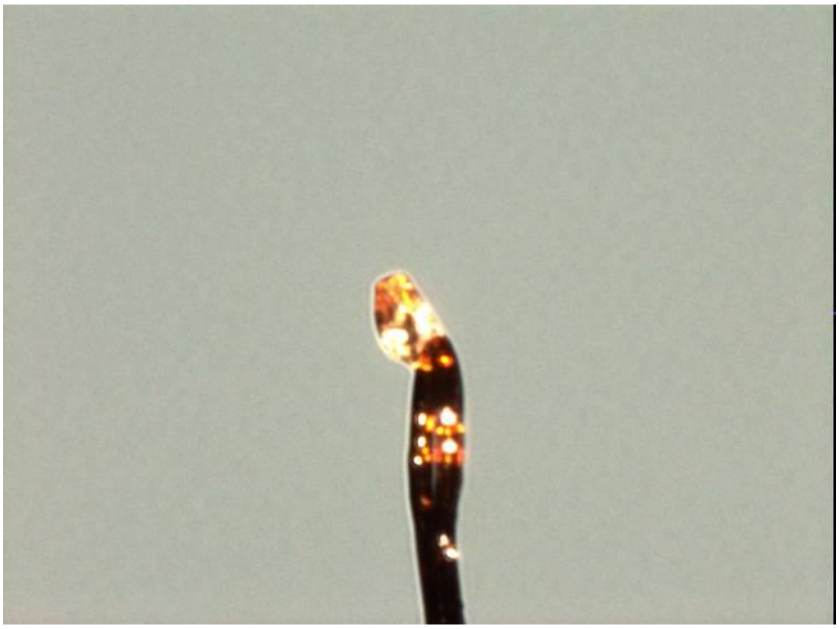

Figure S4 Image of the **6j** Crystal on the Diffractometer.

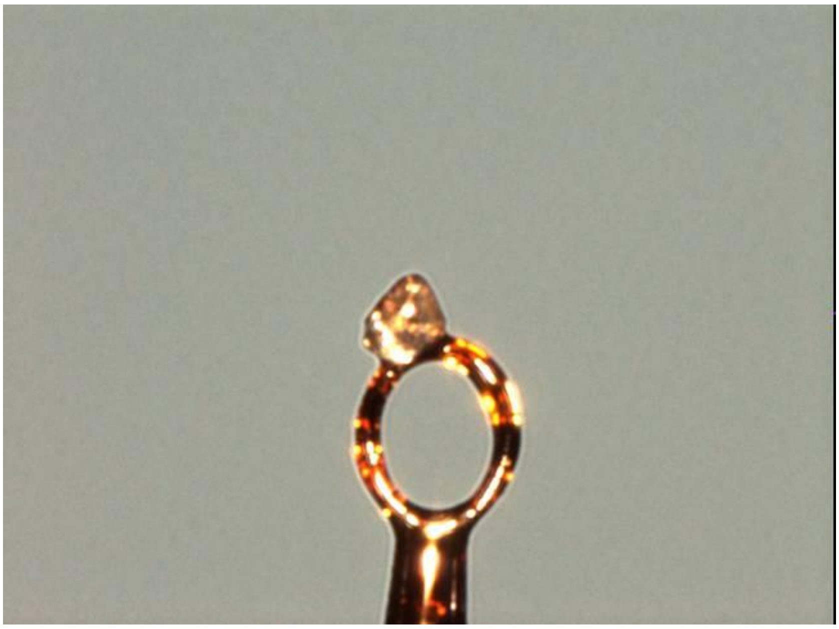

Figure S5 Image of the **6j** Crystal on the Diffractometer.

Data Plots: Diffraction Data

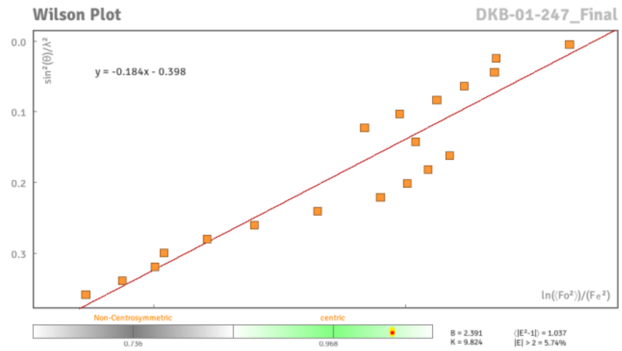

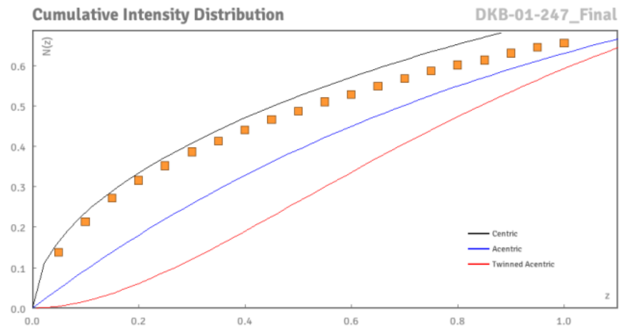

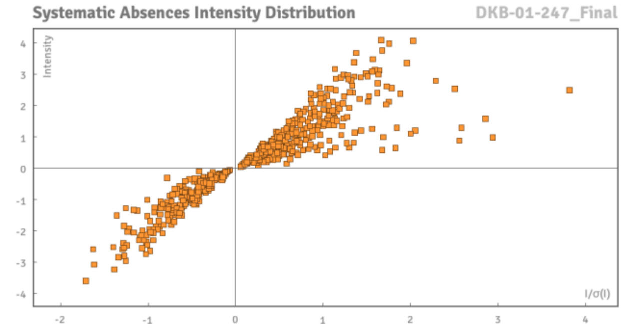

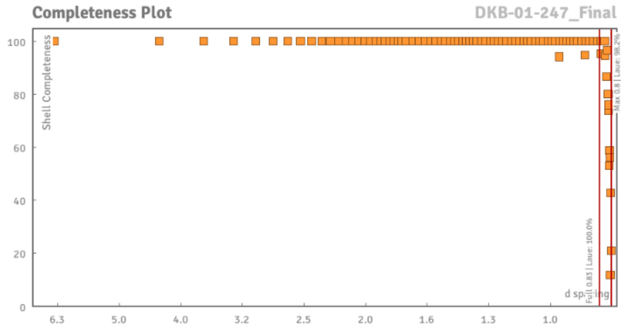

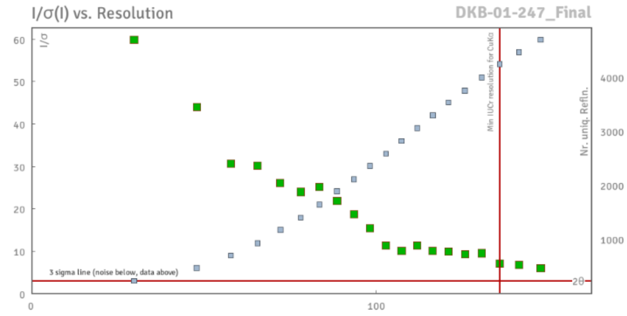

Data Plots: Refinement and Data

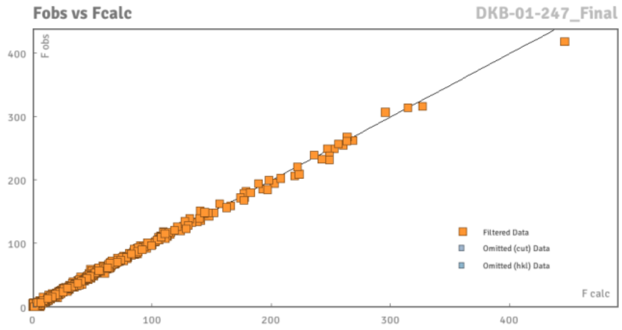

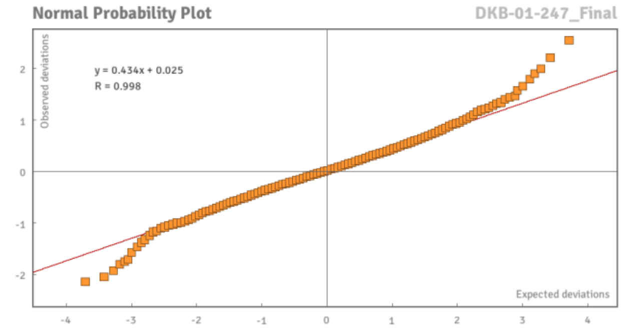

Reflection Statistics

Total reflections (after filtering)	24764	Unique reflections	4842
Completeness	0.982	Mean I/σ	19.65
hkl_{max} collected	(31, 11, 26)	hkl_{min} collected	(-31, -11, -24)
hkl_{max} used	(29, 11, 26)	hkl_{min} used	(-31, 0, 0)
Lim d_{max} collected	100.0	Lim d_{min} collected	0.77
d_{max} used	19.83	d_{min} used	0.8
Friedel pairs	3887	Friedel pairs merged	1

Inconsistent equivalents	0	R_{int}	0.0349
R_{sigma}	0.027	Intensity transformed	0
Omitted reflections	0	Omitted by user (OMIT hkl)	0
Multiplicity	(5848, 3885, 1553, 486, 268, 168, 106, 67, 39, 32, 18, 4)	Maximum multiplicity	19
Removed systematic absences	1050	Filtered off (Shel/OMIT)	0

Table S5: Fractional Atomic Coordinates (×10^4) and Equivalent Isotropic Displacement Parameters (Å^2×10^3) for **6j.** U_{eq} is defined as 1/3 of the trace of the orthogonalised U_{ij}.

Atom	x	y	z	U_{eq}
F1	7185.0(4)	-621.8(11)	4845.8(4)	38.9(2)
F2	6868.9(5)	-2268.8(11)	4109.6(5)	48.5(3)
F3	6306.4(4)	-1125.9(12)	4484.3(5)	43.2(3)
O1	6602.8(4)	1430.9(11)	3995.5(5)	26.6(2)
O2	4880.3(5)	2752.1(14)	3276.4(6)	36.0(3)
O3	5094.9(4)	3797.5(13)	4286.5(5)	30.7(3)
O4	8192.6(5)	6886.6(14)	3434.7(6)	40.7(3)
O5	7568.9(7)	7828(2)	2569.2(8)	75.0(6)
N1	6169.4(5)	-252.5(14)	3163.3(6)	25.9(3)
N2	7713.9(7)	6961.0(16)	3030.8(7)	36.5(3)
C1	6056.9(6)	1923.5(16)	3623.1(7)	24.0(3)
C2	5848.5(6)	3115.7(16)	3952.4(7)	23.4(3)
C3	6004.5(6)	3356.8(16)	3306.5(7)	24.1(3)
C4	5727.6(6)	649.7(16)	3257.8(7)	26.3(3)
C5	6665.2(6)	74.7(16)	3730.8(7)	25.0(3)
C6	6232.0(7)	-32.3(18)	2504.4(7)	30.2(3)
C7	5793.2(7)	-828.6(17)	1946.0(8)	29.3(3)
C8	5707.9(8)	-502(2)	1275.4(8)	41.4(4)
C9	5325.7(9)	-1272(3)	757.7(9)	51.1(5)
C10	5019.1(9)	-2350(2)	898.8(10)	48.4(5)
C11	5096.6(8)	-2672(2)	1558.1(9)	41.8(4)
C12	5483.9(7)	-1922.0(18)	2080.7(8)	33.6(4)
C13	6753.5(7)	-989.0(17)	4296.2(8)	30.3(3)
C14	6211.5(6)	3665.9(16)	4629.5(7)	23.4(3)
C15	6396.9(7)	2753.7(17)	5179.9(8)	29.4(3)
C16	6688.7(7)	3277.8(19)	5819.3(8)	32.9(4)
C17	6796.3(7)	4716.3(19)	5914.6(8)	31.6(4)
C18	6622.7(7)	5627.0(18)	5367.4(8)	30.5(3)
C19	6329.9(6)	5101.7(17)	4725.6(7)	26.4(3)
C20	5226.4(6)	3177.3(17)	3794.7(7)	25.7(3)
C21	4503.1(7)	3966(2)	4165.1(8)	35.8(4)
C22	6474.9(6)	4249.4(16)	3275.2(7)	23.4(3)
C23	6345.8(7)	5197.5(17)	2735.0(7)	27.7(3)
C24	6747.3(7)	6080.3(17)	2647.0(8)	30.6(3)
C25	7286.0(7)	5998.6(17)	3103.4(8)	27.7(3)
C26	7435.1(7)	5064.6(16)	3641.5(7)	26.8(3)
C27	7027.2(7)	4197.7(16)	3729.0(7)	26.0(3)

Table S6: Anisotropic Displacement Parameters (×10^4) for **6j.** The anisotropic displacement factor exponent takes the form: $-2\pi^2[h^2a^{*2} \times U_{11} + ... + 2hka^* \times b^* \times U_{12}]$

Atom	U_{11}	U_{22}	U_{33}	U_{23}	U_{13}	U_{12}
F1	39.6(6)	44.0(6)	24.9(5)	1.7(4)	1.1(4)	2.0(4)
F2	72.2(8)	25.8(5)	41.6(6)	-1.0(4)	12.5(6)	11.6(5)
F3	43.8(6)	48.6(6)	39.1(5)	10.5(5)	16.9(5)	-4.8(5)
O1	23.9(5)	23.7(5)	27.3(5)	-3.8(4)	2.7(4)	1.0(4)

Atom	U_{11}	U_{22}	U_{33}	U_{23}	U_{13}	U_{12}
O2	24.8(6)	53.6(8)	26.8(6)	-7.6(5)	5.4(5)	-1.6(5)
O3	24.8(6)	43.9(7)	24.3(5)	-1.6(5)	9.6(4)	2.8(5)
O4	35.6(7)	42.7(7)	45.9(7)	-6.6(6)	17.1(6)	-10.1(6)
O5	70.2(11)	84.0(12)	57.8(9)	36.1(9)	6.0(8)	-33.8(9)
N1	26.9(7)	28.8(7)	21.2(6)	-3.2(5)	7.1(5)	-0.5(5)
N2	43.2(9)	36.9(8)	32.7(7)	-3.3(6)	17.1(7)	-10.6(7)
C1	21.1(7)	26.9(8)	21.4(7)	-2.0(6)	4.2(6)	0.0(6)
C2	25.1(7)	24.7(7)	19.7(7)	0.4(6)	6.9(6)	-0.2(6)
C3	23.4(7)	29.2(8)	18.1(7)	0.0(6)	5.2(6)	1.2(6)
C4	25.3(8)	27.7(8)	24.4(7)	-3.3(6)	6.9(6)	-2.5(6)
C5	26.2(8)	23.9(7)	24.8(7)	-2.8(6)	8.6(6)	0.4(6)
C6	34.5(9)	32.2(8)	25.2(8)	-1.6(6)	12.1(7)	-1.1(7)
C7	31.4(8)	30.7(8)	24.1(7)	-3.7(6)	7.7(6)	6.3(7)
C8	47.5(11)	48.3(11)	27.0(8)	-0.1(8)	11.4(8)	3.7(9)
C9	57.9(13)	67.8(14)	22.1(8)	-6.6(9)	7.1(8)	10.3(11)
C10	42.7(11)	57.7(13)	35.5(10)	-20.9(9)	2.2(8)	5.6(9)
C11	39.3(10)	39.3(10)	41.8(10)	-14.2(8)	7.9(8)	-1.7(8)
C12	35.9(9)	32.6(9)	29.0(8)	-5.0(7)	7.2(7)	1.4(7)
C13	34.3(9)	27.1(8)	26.8(8)	-1.7(6)	7.2(7)	2.0(6)
C14	21.3(7)	28.2(8)	20.7(7)	-1.2(6)	7.2(6)	0.2(6)
C15	34.7(9)	28.3(8)	24.6(7)	0.9(6)	9.6(7)	3.0(7)
C16	34.9(9)	40.0(9)	21.6(7)	2.8(7)	7.1(7)	8.0(7)
C17	26.5(8)	44.1(10)	22.6(7)	-7.0(7)	6.5(6)	-0.4(7)
C18	31.1(8)	31.8(8)	30.4(8)	-7.1(7)	13.0(7)	-5.3(7)
C19	26.8(8)	29.7(8)	23.3(7)	-0.2(6)	9.3(6)	0.4(6)
C20	27.3(8)	28.6(8)	21.3(7)	3.1(6)	8.6(6)	0.9(6)
C21	25.7(8)	51.8(11)	31.1(8)	2.2(8)	11.4(7)	5.1(8)
C22	26.4(8)	24.4(7)	20.4(7)	-2.2(6)	9.4(6)	0.5(6)
C23	29.6(8)	31.0(8)	20.7(7)	0.9(6)	6.4(6)	0.6(6)
C24	39.0(9)	29.6(8)	23.8(7)	2.6(6)	11.6(7)	-1.5(7)
C25	33.9(8)	27.6(8)	26.3(7)	-4.5(6)	16.4(7)	-5.4(6)
C26	26.8(8)	28.5(8)	24.5(7)	-3.1(6)	8.2(6)	0.0(6)
C27	29.0(8)	26.1(7)	22.0(7)	1.0(6)	7.8(6)	0.7(6)

Table S7: Bond Lengths in Å for **6j**.

Atom	Atom	Length/Å
F1	C13	1.3362(18)
F2	C13	1.3360(18)
F3	C13	1.335(2)
O1	C1	1.4140(17)
O1	C5	1.4294(17)
O2	C20	1.2102(18)
O3	C20	1.3346(18)
O3	C21	1.4435(19)
O4	N2	1.2203(19)
O5	N2	1.228(2)
N1	C4	1.478(2)
N1	C5	1.4384(18)
N1	C6	1.4724(19)
N2	C25	1.466(2)
C1	C2	1.514(2)
C1	C3	1.496(2)
C1	C4	1.515(2)
C2	C3	1.567(2)
C2	C14	1.5004(19)
C2	C20	1.499(2)
C3	C22	1.483(2)

Atom	Atom	Length/Å
C5	C13	1.518(2)
C6	C7	1.512(2)
C7	C8	1.393(2)
C7	C12	1.387(2)
C8	C9	1.389(3)
C9	C10	1.377(3)
C10	C11	1.373(3)
C11	C12	1.391(2)
C14	C15	1.392(2)
C14	C19	1.389(2)
C15	C16	1.388(2)
C16	C17	1.387(2)
C17	C18	1.385(2)
C18	C19	1.393(2)
C22	C23	1.398(2)
C22	C27	1.398(2)
C23	C24	1.380(2)
C24	C25	1.375(2)
C25	C26	1.385(2)
C26	C27	1.383(2)

Table S8: Bond Angles in ° for **6j.**

Atom	Atom	Atom	Angle/°
C1	O1	C5	107.35(11)
C20	O3	C21	116.01(12)
C5	N1	C4	104.40(11)
C5	N1	C6	114.05(12)
C6	N1	C4	112.64(12)
O4	N2	O5	122.98(15)
O4	N2	C25	119.03(14)
O5	N2	C25	117.97(15)
O1	C1	C2	114.90(12)
O1	C1	C3	117.65(12)
O1	C1	C4	106.42(12)
C2	C1	C4	127.32(13)
C3	C1	C2	62.74(10)
C3	C1	C4	122.94(12)
C1	C2	C3	58.06(9)
C14	C2	C1	120.00(13)
C14	C2	C3	121.60(12)
C20	C2	C1	115.95(12)
C20	C2	C3	111.63(12)
C20	C2	C14	116.65(12)
C1	C3	C2	59.19(9)
C22	C3	C1	125.99(13)
C22	C3	C2	125.09(12)
N1	C4	C1	102.52(12)
O1	C5	N1	109.58(12)
O1	C5	C13	106.91(12)
N1	C5	C13	111.17(13)
N1	C6	C7	112.07(13)
C8	C7	C6	119.72(16)
C12	C7	C6	121.77(14)
C12	C7	C8	118.47(16)
C9	C8	C7	120.29(19)
C10	C9	C8	120.61(18)
C11	C10	C9	119.53(18)
C10	C11	C12	120.38(19)
C7	C12	C11	120.71(17)
F1	C13	C5	111.39(13)
F2	C13	F1	107.10(13)
F2	C13	C5	110.72(13)
F3	C13	F1	106.91(13)
F3	C13	F2	107.33(14)
F3	C13	C5	113.09(13)
C15	C14	C2	120.00(14)
C19	C14	C2	120.52(13)
C19	C14	C15	119.30(14)
C16	C15	C14	120.29(15)
C17	C16	C15	120.17(15)
C18	C17	C16	119.87(15)
C17	C18	C19	119.97(15)
C14	C19	C18	120.37(15)
O2	C20	O3	123.50(14)
O2	C20	C2	124.43(14)
O3	C20	C2	112.03(12)
C23	C22	C3	115.99(13)
C27	C22	C3	125.53(13)
C27	C22	C23	118.48(14)
C24	C23	C22	121.61(15)
C25	C24	C23	118.24(14)
C24	C25	N2	118.99(14)
C24	C25	C26	122.21(15)
C26	C25	N2	118.78(15)
C27	C26	C25	119.00(15)
C26	C27	C22	120.46(14)

Table S9: Torsion Angles in ° for **6j.**

Atom	Atom	Atom	Atom	Angle/°
O1	C1	C2	C3	109.66(14)
O1	C1	C2	C14	-1.0(2)
O1	C1	C2	C20	-149.99(13)
O1	C1	C3	C2	-105.36(14)
O1	C1	C3	C22	7.9(2)
O1	C1	C4	N1	-28.90(14)
O1	C5	C13	F1	-55.05(17)
O1	C5	C13	F2	-174.11(12)
O1	C5	C13	F3	65.39(16)
O4	N2	C25	C24	-179.80(15)
O4	N2	C25	C26	2.0(2)
O5	N2	C25	C24	1.5(2)
O5	N2	C25	C26	-176.69(17)
N1	C5	C13	F1	-174.61(12)
N1	C5	C13	F2	66.32(17)
N1	C5	C13	F3	-54.17(17)
N1	C6	C7	C8	165.67(15)
N1	C6	C7	C12	-16.8(2)
N2	C25	C26	C27	177.20(14)
C1	O1	C5	N1	3.42(15)
C1	O1	C5	C13	-117.16(13)
C1	C2	C3	C22	-114.67(16)

Atom	Atom	Atom	Atom	Angle/°
C1	C2	C14	C15	-58.1(2)
C1	C2	C14	C19	126.85(16)
C1	C2	C20	O2	-28.6(2)
C1	C2	C20	O3	153.27(13)
C1	C3	C22	C23	152.89(14)
C1	C3	C22	C27	-26.0(2)
C2	C1	C3	C22	113.21(16)
C2	C1	C4	N1	-169.86(13)
C2	C3	C22	C23	-132.38(15)
C2	C3	C22	C27	48.7(2)
C2	C14	C15	C16	-174.02(15)
C2	C14	C19	C18	173.99(14)
C3	C1	C2	C14	-110.67(15)
C3	C1	C2	C20	100.35(14)
C3	C1	C4	N1	111.22(15)
C3	C2	C14	C15	-126.90(15)
C3	C2	C14	C19	58.1(2)
C3	C2	C20	O2	35.3(2)
C3	C2	C20	O3	-142.82(13)
C3	C22	C23	C24	-179.60(14)
C3	C22	C27	C26	178.67(14)
C4	N1	C5	O1	-21.86(15)
C4	N1	C5	C13	96.09(14)
C4	N1	C6	C7	-80.59(16)
C4	C1	C2	C3	-112.11(17)
C4	C1	C2	C14	137.22(15)
C4	C1	C2	C20	-11.8(2)
C4	C1	C3	C2	118.61(16)
C4	C1	C3	C22	-128.17(16)
C5	O1	C1	C2	162.63(12)
C5	O1	C1	C3	-126.45(13)
C5	O1	C1	C4	16.15(15)
C5	N1	C4	C1	30.16(14)
C5	N1	C6	C7	160.65(13)
C6	N1	C4	C1	-94.10(14)
C6	N1	C5	O1	101.49(14)
C6	N1	C5	C13	-140.56(13)
C6	C7	C8	C9	176.84(17)
C6	C7	C12	C11	-177.71(16)
C7	C8	C9	C10	1.1(3)
C8	C7	C12	C11	-0.2(3)
C8	C9	C10	C11	-0.5(3)
C9	C10	C11	C12	-0.4(3)
C10	C11	C12	C7	0.7(3)
C12	C7	C8	C9	-0.8(3)
C14	C2	C3	C1	107.95(15)
C14	C2	C3	C22	-6.7(2)
C14	C2	C20	O2	-178.61(15)
C14	C2	C20	O3	3.23(19)
C14	C15	C16	C17	0.2(2)
C15	C14	C19	C18	-1.1(2)
C15	C16	C17	C18	-1.4(3)
C16	C17	C18	C19	1.4(2)
C17	C18	C19	C14	-0.2(2)
C19	C14	C15	C16	1.1(2)
C20	C2	C3	C1	-107.91(14)
C20	C2	C3	C22	137.42(15)
C20	C2	C14	C15	90.64(18)
C20	C2	C14	C19	-84.38(18)
C21	O3	C20	O2	-1.0(2)
C21	O3	C20	C2	177.14(13)
C22	C23	C24	C25	0.7(2)

Atom	Atom	Atom	Atom	Angle/°
C23	C22	C27	C26	-0.2(2)
C23	C24	C25	N2	-178.00(14)
C23	C24	C25	C26	0.1(2)
C24	C25	C26	C27	-0.9(2)
C25	C26	C27	C22	1.0(2)
C27	C22	C23	C24	-0.6(2)

Table S10: Hydrogen Fractional Atomic Coordinates (×10^4) and Equivalent Isotropic Displacement Parameters (Å^2×10^3) for **6j.** U_{eq} is defined as 1/3 of the trace of the orthogonalised U_{ij}.

Atom	x	y	z	U_{eq}
H3	5664.7	3436.74	2888.75	29
H4A	5537.78	165.02	3533.69	32
H4B	5444.04	923.09	2819.3	32
H5	7000.21	80.93	3586.44	30
H6A	6610.09	-350.37	2530.07	36
H6B	6200.95	991.01	2397.13	36
H8	5912.08	250.71	1171.71	50
H9	5275.26	-1052.08	302.17	61
H10	4755.97	-2867.41	542.63	58
H11	4884.38	-3411.1	1657.73	50
H12	5537.11	-2161.33	2534.92	40
H15	6323.39	1768.1	5117.4	35
H16	6815.06	2649.94	6192.78	39
H17	6988.62	5076.2	6354.18	38
H18	6703.34	6609.56	5430.06	37
H19	6210.43	5728.8	4351.38	32
H21A	4323.61	4443.74	3731.76	54
H21B	4452.9	4535.46	4526.95	54
H21C	4331.13	3033.98	4153.95	54
H23	5972.61	5234.83	2420.82	33
H24	6653.97	6727.3	2280.68	37
H26	7811.7	5019.83	3945.83	32
H27	7123.14	3562.88	4100.18	31

Methyl 4-(-2-phenyl-2,5-bis(trifluoromethyl)-4-oxa-6-azaspiro[2.4]heptan-1-yl)benzoate (16b)

CCDC deposition number: 2451793

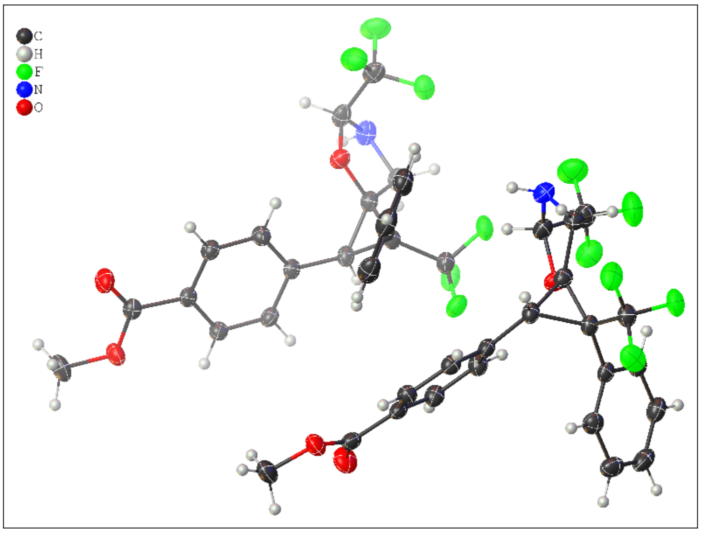

Crystals of compound **16b** were collected upon crystallization via slow evaporation with pentane in toluene.

Experimental. Single colourless plate-shaped crystals of **16b** were used as supplied. A suitable crystal with dimensions 0.25 × 0.16 × 0.06 mm³ was selected and mounted on an XtaLAB Synergy R, DW system, HyPix-Arc 150 diffractometer. The crystal was kept at a steady T = 140.00(10) K during data collection. The structure was solved with the ShelXT 2018/2 (Sheldrick, 2015) solution program using dual methods and by using Olex2 1.5 (Dolomanov et al., 2009) as the graphical interface. The model was refined with ShelXL 2019/3 (Sheldrick, 2015) using full matrix least squares minimisation on $\boldsymbol{F}^2$.

Crystal Data. $C_{21}H_{17}NO_3F_6$, M_r = 445.36, monoclinic, $P2_1/c$ (No. 14), a = 14.77504(10) Å, b = 24.62736(17) Å, c = 11.15008(6) Å, β = 90.3773(6)°, α = γ = 90°, V = 4057.09(5) Å³, T = 140.00(10) K, Z = 8, Z' = 2, μ(Cu K_α) = 1.178, 54330 reflections measured, 8257 unique (R_{int} = 0.0170) which were used in all calculations. The final wR_2 was 0.0786 (all data) and R_1 was 0.0304 (I≥2σ(I)).

**Compound**	**16b**
Formula	$C_{21}H_{17}NO_3F_6$
$D_{calc.}$/ g cm^{-3}	1.458
μ/mm^{-1}	1.178
Formula Weight	445.36
Colour	colourless
Shape	plate-shaped
Size/mm³	0.25×0.16×0.06
T/K	140.00(10)
Crystal System	monoclinic
Space Group	$P2_1/c$
a/Å	14.77504(10)
b/Å	24.62736(17)
c/Å	11.15008(6)
α/°	90
β/°	90.3773(6)
γ/°	90
V/Å³	4057.09(5)
Z	8
Z'	2
Wavelength/Å	1.54184
Radiation type	Cu$K\alpha$
Θ_{min}/°	2.991
Θ_{max}/°	75.363
Measured Refl's.	54330
Indep't Refl's	8257
Refl's I≥2σ(I)	7455
R_{int}	0.0170
Parameters	570
Restraints	0
Largest Peak/e Å^{-3}	0.290
Deepest Hole/e Å^{-3}	-0.200
GooF	1.013
wR_2 (all data)	0.0786
wR_2	0.0768
R_1 (all data)	0.0336
R_1	0.0304
CCDC number	2451793

Structure Quality Indicators

**Reflections:**	d min (CuKα) 2Θ=150.7° 0.80	I/σ(I) 90.9	Rint m=6.71 1.70%	Full 135.4° 98% to 150.7° 99.9
**Refinement:**	Shift 0.001	Max Peak 0.3	Min Peak -0.2	GooF 1.013

A colourless plate-shaped crystal with dimensions 0.25 × 0.16 × 0.06 mm^3 was mounted. Data were collected using an XtaLAB Synergy R, DW system, HyPix-Arc 150 diffractometer operating at T = 140.00(10) K.

Data were measured using ω scans with Cu K_α radiation. The diffraction pattern was indexed and the total number of runs and images was based on the strategy calculation from the program CrysAlisPro system (CCD 43.143a 64-bit (release 25-10-2024)). The maximum resolution achieved was Θ = 75.363° (0.80 Å).

The unit cell was refined using CrysAlisPro 1.171.43.143a (Rigaku OD, 2024) on 37169 reflections, 68% of the observed reflections.

Data reduction, scaling and absorption corrections were performed using CrysAlisPro 1.171.43.143a (Rigaku OD, 2024). The final completeness is 99.90 % out to 75.363° in Θ. A Gaussian absorption correction was performed using CrysAlisPro 1.171.43.143a (Rigaku Oxford Diffraction, 2024) Numerical absorption correction based on Gaussian integration over a multifaceted crystal model. Empirical absorption correction using spherical harmonics as implemented in SCALE3 ABSPACK scaling algorithm. The absorption coefficient μ of this material is 1.178 mm^{-1} at this wavelength (λ = 1.54184Å) and the minimum and maximum transmissions are 0.591 and 1.000.

The structure was solved in the space group $P2_1/c$ (# 14) by the ShelXT 2018/2 (Sheldrick, 2015) structure solution program using dual methods and refined by full matrix least squares minimisation on $\boldsymbol{F^2}$ using version 2019/3 of ShelXL 2019/3 (Sheldrick, 2015). All non-hydrogen atoms were refined anisotropically. Hydrogen atom positions were calculated geometrically and refined using the riding model, but hydrogen atoms bound to nitrogen atoms were refined freely.

The value of Z' is 2. This means that there are two independent molecules in the asymmetric unit. The moiety formula is C21 H17 F6 N O3.

Citations

CrysAlis^{Pro} Software System, Rigaku Oxford Diffraction, (2024).

O.V. Dolomanov and L.J. Bourhis and R.J. Gildea and J.A.K. Howard and H. Puschmann, Olex2: A complete structure solution, refinement and analysis program, *J. Appl. Cryst.*, (2009), **42**, 339-341.

Sheldrick, G.M., Crystal structure refinement with ShelXL, *Acta Cryst.*, (2015), **C71**, 3-8.

Sheldrick, G.M., ShelXT-Integrated space-group and crystal-structure determination, *Acta Cryst.*, (2015), **A71**, 3-8.

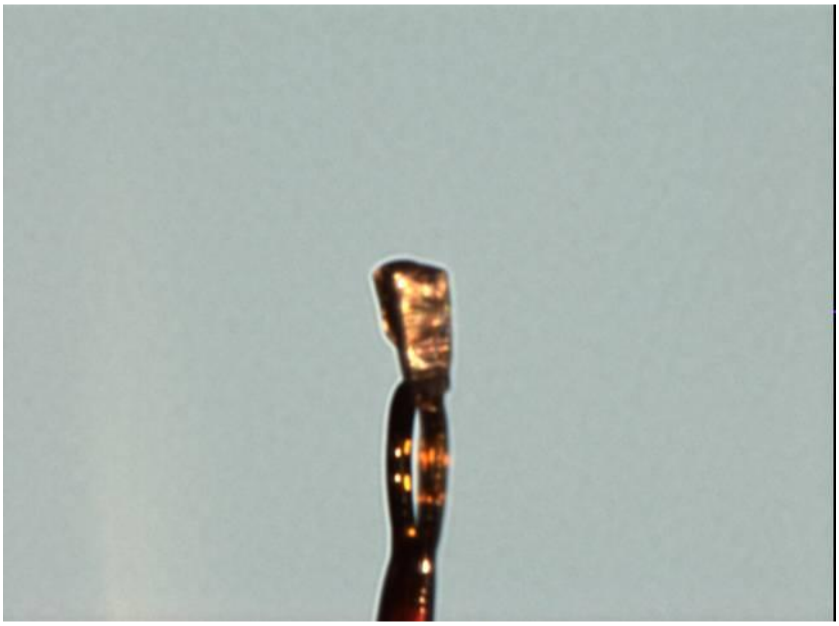

Figure S6 Image of the **16b** Crystal on the Diffractometer.

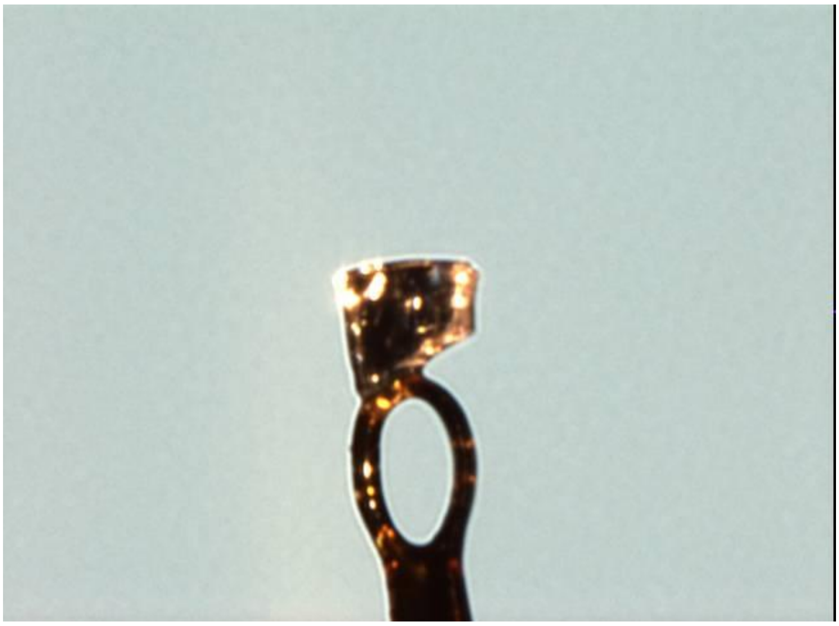

Figure S7 Image of the **16b** Crystal on the Diffractometer.

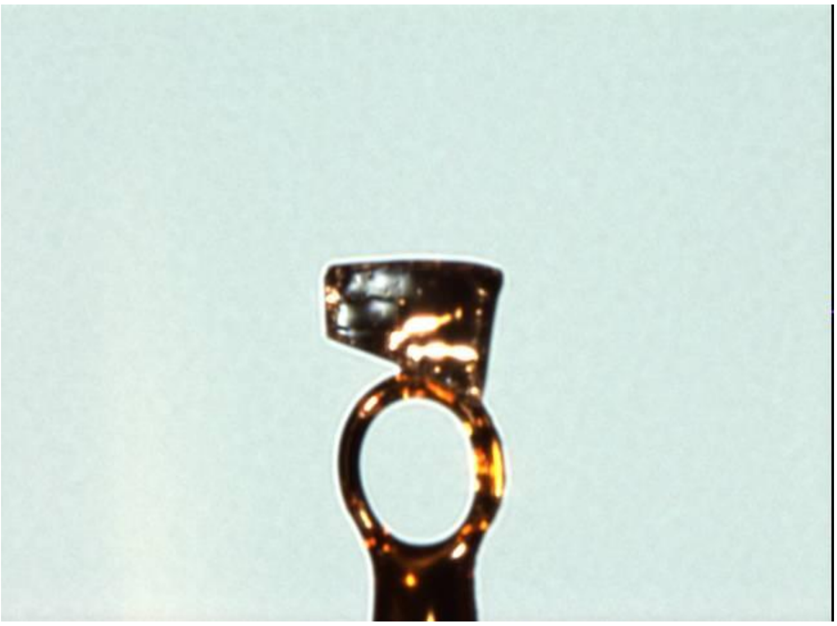

Figure S8 Image of the **16b** Crystal on the Diffractometer.

Data Plots: Diffraction Data

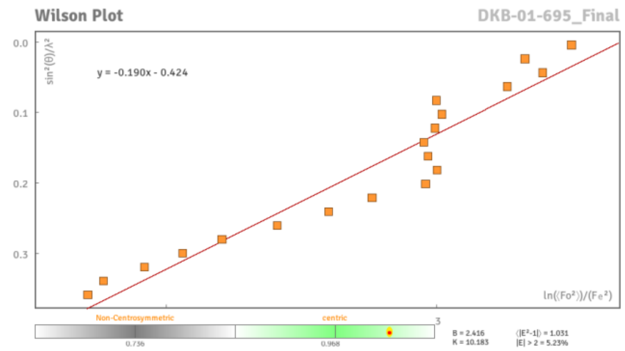

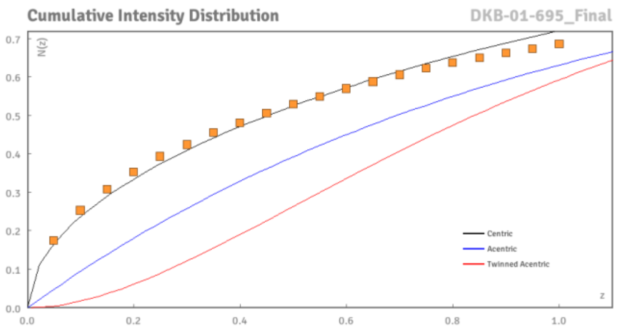

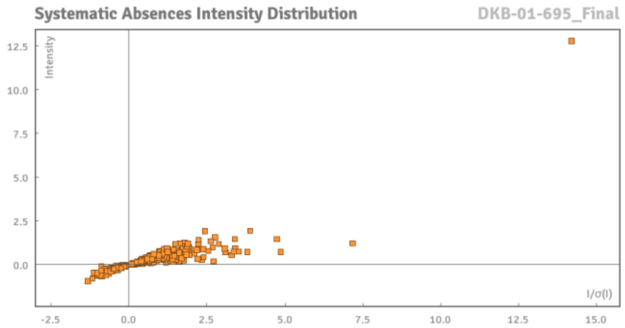

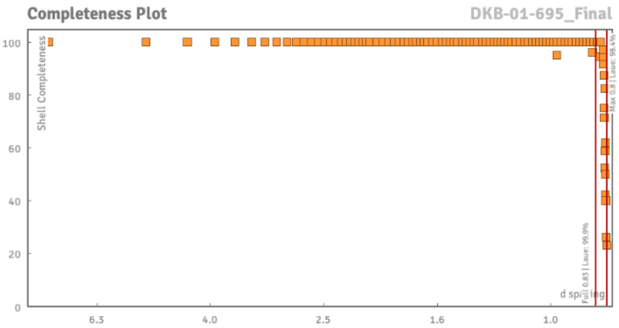

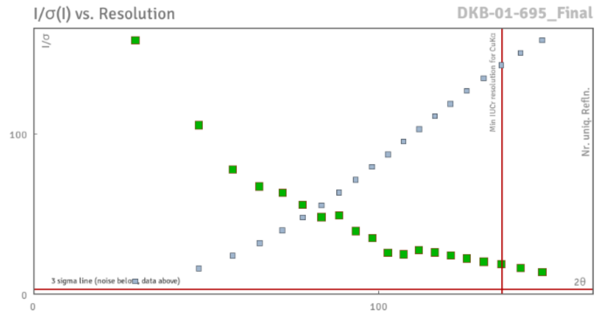

Data Plots: Refinement and Data

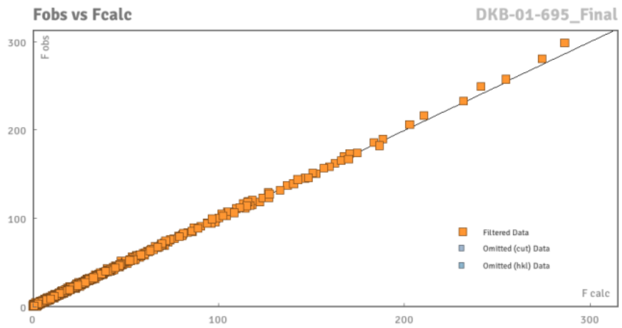

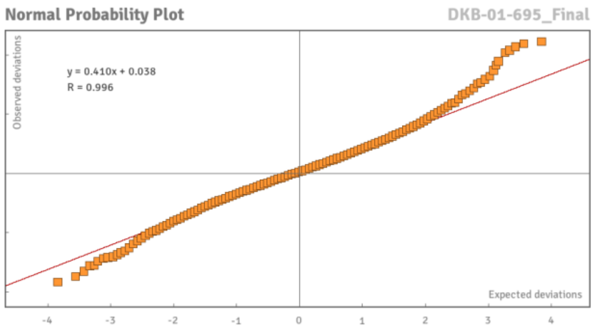

Reflection Statistics

Total reflections (after filtering)	55378	Unique reflections	8257
Completeness	0.984	Mean I/σ	46.44
hkl_{max} collected	(18, 30, 12)	hkl_{min} collected	(-18, -30, -13)
hkl_{max} used	(18, 30, 13)	hkl_{min} used	(-18, 0, 0)
Lim d_{max} collected	100.0	Lim d_{min} collected	0.77
d_{max} used	24.63	d_{min} used	0.8
Friedel pairs	6440	Friedel pairs merged	1
Inconsistent equivalents	0	R_{int}	0.017
R_{sigma}	0.011	Intensity transformed	0
Omitted reflections	0	Omitted by user (OMIT hkl)	0
Multiplicity	(8166, 6212, 3339, 1600, 828, 496, 282, 193, 161, 120, 110, 79, 47, 47, 35, 22, 22, 12, 7, 2, 1)	Maximum multiplicity	31
Removed systematic absences	1048	Filtered off (Shel/OMIT)	0

Table S11: Fractional Atomic Coordinates (×10^4) and Equivalent Isotropic Displacement Parameters (Å^2×10^3) for **16b**. U_{eq} is defined as 1/3 of the trace of the orthogonalised U_{ij}.

Atom	x	y	z	U_{eq}
F1	11289.2(6)	5042.4(3)	2852.2(8)	50.0(2)
F2	9879.0(5)	4846.6(3)	3015.5(6)	36.25(16)
F3	10670.3(6)	4478.8(3)	1620.0(6)	42.31(18)
F4	7802.8(5)	4187.1(3)	4559.7(7)	37.29(17)
F5	8298.3(5)	3752.8(3)	6104.8(6)	36.82(17)
F6	7326.4(5)	3379.2(3)	4924.9(6)	36.48(17)
O1	10294.6(5)	3750.9(3)	3368.9(7)	27.66(17)
O2	11607.2(6)	1094.9(4)	2267.1(7)	36.1(2)
O3	11233.2(6)	755.6(3)	4058.7(7)	33.28(19)
N1	10886.9(7)	4333.7(4)	4830.1(9)	32.5(2)
C1	9747.4(7)	3695.0(4)	4396.8(9)	24.5(2)
C2	8815.9(7)	3469.7(4)	4180.5(9)	23.1(2)
C3	9545.3(7)	3127.7(5)	4834.4(9)	24.7(2)
C4	10942.2(7)	4171.7(5)	3599.6(11)	29.8(2)
C5	9979.2(8)	4171.0(5)	5220.0(10)	29.4(2)
C6	10693.3(8)	4636.3(5)	2767.2(11)	32.5(3)
C7	8066.6(8)	3694.3(5)	4944.9(10)	28.1(2)
C8	8478.7(7)	3334.0(4)	2943.8(9)	23.2(2)
C9	8473.3(7)	3728.7(5)	2048.7(10)	26.5(2)
C10	8068.3(8)	3621.6(5)	945.2(11)	32.5(3)
C11	7667.8(8)	3123.5(6)	735.6(11)	36.1(3)
C12	7677.9(8)	2727.4(5)	1614.0(12)	36.3(3)

Atom	x	y	z	U_{eq}
C13	8085.0(8)	2831.2(5)	2717.6(11)	29.8(2)
C14	9945.7(7)	2618.4(4)	4343.6(9)	23.2(2)
C15	9943.7(7)	2162.0(5)	5084.5(10)	25.4(2)
C16	10348.1(7)	1680.2(5)	4741.5(10)	26.0(2)
C17	10759.5(7)	1642.1(4)	3622.0(9)	23.2(2)
C18	10742.7(7)	2090.3(5)	2861.7(9)	25.8(2)
C19	10347.7(8)	2574.0(5)	3213.6(10)	26.5(2)
C20	11241.8(7)	1143.1(5)	3225.7(10)	25.3(2)
C21	11721.7(10)	263.5(5)	3783.9(13)	39.9(3)
F7	5865.3(6)	4572.6(4)	4352.2(7)	47.0(2)
F8	5234.9(6)	5039.2(4)	2951.6(8)	55.9(2)
F9	6686.6(6)	5034.4(4)	3143.4(9)	55.7(2)
F10	2717.2(5)	4271.7(3)	1387.9(7)	38.36(17)
F11	3330.7(5)	3907.0(3)	-157.5(6)	42.31(19)
F12	2378.0(5)	3454.4(3)	882.2(7)	43.12(19)
O4	5262.3(5)	3895.9(3)	2647.9(7)	30.59(18)
O5	6432.8(7)	1198.3(4)	3841.0(8)	41.7(2)
O6	6261.7(6)	899.8(3)	1958.7(8)	33.22(19)
N2	5959.2(7)	4400.0(5)	1135.1(10)	34.9(2)
C22	4743.6(7)	3830.8(5)	1589.2(10)	26.7(2)
C23	3813.0(7)	3598.0(5)	1765.7(10)	25.1(2)
C24	4558.1(7)	3262.9(5)	1136.4(10)	26.2(2)
C25	6016.7(8)	4240.8(5)	2373.1(11)	31.4(3)
C26	5013.6(8)	4298.4(5)	776.9(11)	32.5(3)
C27	5946.7(9)	4724.6(6)	3211.2(12)	38.5(3)
C28	3065.4(8)	3805.4(5)	966.8(11)	29.8(2)
C29	3473.0(7)	3448.9(5)	2989.5(10)	25.3(2)
C30	3500.2(8)	3825.6(5)	3922.8(10)	28.6(2)
C31	3117.2(8)	3700.9(5)	5026.8(11)	33.7(3)
C32	2707.4(8)	3204.0(6)	5198.7(11)	36.9(3)
C33	2679.0(8)	2826.1(6)	4280.5(12)	37.1(3)
C34	3064.6(8)	2948.4(5)	3179.2(11)	31.2(2)
C35	4941.2(7)	2751.1(5)	1636.3(9)	24.6(2)
C36	4923.3(7)	2291.6(5)	905.2(10)	26.0(2)
C37	5309.9(7)	1805.6(5)	1265.4(10)	26.7(2)
C38	5711.5(7)	1768.7(5)	2397.7(10)	24.9(2)
C39	5704.4(8)	2218.6(5)	3150.8(10)	28.4(2)
C40	5331.3(8)	2706.3(5)	2779.6(10)	29.0(2)
C41	6165.5(7)	1266.3(5)	2823.9(10)	27.1(2)
C42	6742.2(9)	412.9(5)	2293.9(14)	41.2(3)

Table S12: Anisotropic Displacement Parameters (×10⁴) for **16b**. The anisotropic displacement factor exponent takes the form: $-2\pi^2[h^2a^{*2} \times U_{11} + \ldots + 2hka^* \times b^* \times U_{12}]$

Atom	U_{11}	U_{22}	U_{33}	U_{23}	U_{13}	U_{12}
F1	47.9(5)	42.7(4)	59.6(5)	-5.0(4)	6.0(4)	-22.9(4)
F2	34.8(4)	33.2(4)	40.7(4)	-4.0(3)	-2.4(3)	4.5(3)
F3	55.1(5)	40.1(4)	31.9(4)	-4.5(3)	7.6(3)	-6.0(4)
F4	38.8(4)	32.0(4)	41.1(4)	1.5(3)	5.1(3)	15.6(3)
F5	37.0(4)	48.2(4)	25.3(3)	-6.8(3)	3.8(3)	12.6(3)
F6	25.7(3)	43.4(4)	40.5(4)	-1.9(3)	8.8(3)	0.7(3)
O1	27.2(4)	28.6(4)	27.3(4)	-6.4(3)	3.7(3)	-3.7(3)
O2	43.2(5)	36.7(5)	28.5(4)	-4.1(4)	6.7(4)	10.6(4)
O3	42.8(5)	23.7(4)	33.4(4)	-0.1(3)	4.3(4)	7.9(3)
N1	25.6(5)	38.2(6)	33.5(5)	-7.8(4)	-6.3(4)	0.8(4)
C1	23.3(5)	28.0(5)	22.2(5)	-3.9(4)	0.0(4)	3.6(4)
C2	22.6(5)	24.2(5)	22.5(5)	-0.6(4)	0.4(4)	3.8(4)
C3	25.3(5)	28.8(6)	20.1(5)	-1.4(4)	-0.4(4)	6.2(4)
C4	20.1(5)	33.3(6)	35.9(6)	-8.6(5)	0.1(4)	-1.5(4)

Atom	U_{11}	U_{22}	U_{33}	U_{23}	U_{13}	U_{12}
C5	28.5(6)	31.3(6)	28.5(6)	-8.2(5)	-1.5(4)	1.3(5)
C6	29.5(6)	32.5(6)	35.6(6)	-7.6(5)	3.5(5)	-7.9(5)
C7	26.7(5)	30.4(6)	27.4(6)	-0.5(4)	1.5(4)	5.8(4)
C8	18.3(5)	26.5(5)	24.8(5)	-2.4(4)	1.4(4)	2.2(4)
C9	24.3(5)	27.2(5)	28.0(5)	-1.2(4)	-1.0(4)	2.7(4)
C10	29.3(6)	42.4(7)	25.8(6)	0.6(5)	-1.5(5)	7.4(5)
C11	25.3(6)	54.2(8)	28.8(6)	-12.0(5)	-3.6(5)	2.7(5)
C12	27.0(6)	40.5(7)	41.4(7)	-14.0(6)	2.7(5)	-8.2(5)
C13	26.7(5)	30.0(6)	32.9(6)	-2.0(5)	4.4(5)	-3.4(4)
C14	20.8(5)	27.0(5)	21.9(5)	-2.1(4)	-2.1(4)	4.0(4)
C15	23.4(5)	31.5(6)	21.3(5)	0.4(4)	3.3(4)	2.9(4)
C16	26.7(5)	26.7(5)	24.4(5)	2.8(4)	1.1(4)	2.1(4)
C17	20.0(5)	26.0(5)	23.5(5)	-2.4(4)	-1.7(4)	1.7(4)
C18	26.7(5)	30.3(6)	20.3(5)	-1.4(4)	2.1(4)	2.5(4)
C19	30.5(6)	27.6(6)	21.3(5)	1.6(4)	0.3(4)	4.1(4)
C20	23.4(5)	27.6(6)	24.8(5)	-3.9(4)	-3.2(4)	0.6(4)
C21	49.6(8)	24.7(6)	45.2(7)	-6.9(5)	-3.6(6)	10.2(5)
F7	44.7(4)	58.0(5)	38.2(4)	-8.4(4)	1.3(3)	-3.4(4)
F8	56.0(5)	51.3(5)	60.3(5)	-13.1(4)	-4.6(4)	22.6(4)
F9	52.2(5)	49.2(5)	65.8(6)	-10.3(4)	5.8(4)	-20.4(4)
F10	37.3(4)	35.2(4)	42.6(4)	-3.1(3)	-4.8(3)	15.0(3)
F11	43.0(4)	56.7(5)	27.2(4)	6.7(3)	-4.0(3)	17.3(4)
F12	34.3(4)	43.3(4)	51.5(5)	1.1(4)	-18.8(3)	-1.7(3)
O4	25.0(4)	38.3(5)	28.4(4)	3.4(3)	-1.7(3)	-5.1(3)
O5	49.0(5)	43.9(5)	32.1(5)	8.6(4)	-6.3(4)	11.1(4)
O6	35.6(4)	25.8(4)	38.2(5)	3.3(3)	-1.8(4)	4.6(3)
N2	31.7(5)	36.1(6)	37.0(6)	1.0(5)	9.2(4)	-0.9(4)
C22	23.6(5)	32.0(6)	24.7(5)	2.5(4)	0.8(4)	3.2(4)
C23	22.7(5)	28.5(5)	24.2(5)	-1.1(4)	-0.6(4)	3.7(4)
C24	26.2(5)	32.2(6)	20.2(5)	1.4(4)	1.0(4)	7.2(4)
C25	22.5(5)	34.3(6)	37.6(6)	0.2(5)	3.9(5)	-0.5(5)
C26	32.8(6)	32.6(6)	32.2(6)	5.6(5)	4.3(5)	2.9(5)
C27	30.3(6)	40.0(7)	45.3(7)	-2.9(6)	1.3(5)	0.0(5)
C28	27.7(6)	31.8(6)	30.0(6)	-1.5(5)	-2.9(5)	5.7(5)
C29	18.4(5)	31.1(6)	26.3(5)	-0.1(4)	-0.1(4)	3.8(4)
C30	24.5(5)	31.2(6)	30.0(6)	-1.4(5)	0.5(4)	3.8(4)
C31	29.3(6)	44.5(7)	27.4(6)	-3.8(5)	1.6(5)	7.9(5)
C32	26.1(6)	54.0(8)	30.8(6)	7.8(6)	5.3(5)	5.0(5)
C33	26.7(6)	41.8(7)	42.9(7)	8.5(6)	1.1(5)	-4.8(5)
C34	25.6(5)	33.9(6)	34.0(6)	-2.0(5)	-2.1(5)	-1.9(5)
C35	20.4(5)	31.1(6)	22.4(5)	2.3(4)	3.2(4)	4.0(4)
C36	23.1(5)	32.8(6)	22.0(5)	1.8(4)	-1.7(4)	0.7(4)
C37	25.3(5)	28.3(6)	26.5(5)	-0.1(4)	0.1(4)	-0.8(4)
C38	19.5(5)	29.9(6)	25.4(5)	4.7(4)	3.0(4)	1.2(4)
C39	27.6(5)	37.1(6)	20.6(5)	2.4(4)	-1.3(4)	4.9(5)
C40	31.7(6)	33.5(6)	21.7(5)	-1.8(4)	0.1(4)	5.7(5)
C41	21.2(5)	30.7(6)	29.4(6)	6.2(5)	2.7(4)	-1.3(4)
C42	37.9(7)	27.7(6)	58.0(8)	8.2(6)	0.1(6)	7.0(5)

Table S13: Bond Lengths in Å for **16b**.

Atom	Atom	Length/Å
F1	C6	1.3353(14)
F2	C6	1.3404(14)
F3	C6	1.3368(14)
F4	C7	1.3442(13)
F5	C7	1.3434(13)
F6	C7	1.3412(14)
O1	C1	1.4137(13)
O1	C4	1.4325(13)
O2	C20	1.2064(14)
O3	C20	1.3318(14)
O3	C21	1.4444(14)
N1	C4	1.4318(15)
N1	C5	1.4683(15)
C1	C2	1.5019(15)

Atom	Atom	Length/Å
C1	C3	1.5102(16)
C1	C5	1.5264(15)
C2	C3	1.5466(14)
C2	C7	1.5068(15)
C2	C8	1.5007(14)
C3	C14	1.4923(14)
C4	C6	1.5170(18)
C8	C9	1.3930(16)
C8	C13	1.3905(16)
C9	C10	1.3899(16)
C10	C11	1.3812(19)
C11	C12	1.382(2)
C12	C13	1.3898(17)
C14	C15	1.3950(15)
C14	C19	1.4007(15)
C15	C16	1.3835(15)
C16	C17	1.3952(15)
C17	C18	1.3920(15)
C17	C20	1.4891(15)
C18	C19	1.3844(15)
F7	C27	1.3323(16)
F8	C27	1.3364(15)
F9	C27	1.3358(15)
F10	C28	1.3443(13)
F11	C28	1.3395(14)
F12	C28	1.3365(14)
O4	C22	1.4122(13)
O4	C25	1.4360(14)
O5	C41	1.2101(14)
O6	C41	1.3294(14)
O6	C42	1.4417(14)
N2	C25	1.4370(16)
N2	C26	1.4719(16)
C22	C23	1.5038(15)
C22	C24	1.5114(16)
C22	C26	1.5200(16)
C23	C24	1.5477(15)
C23	C28	1.5039(15)
C23	C29	1.5027(15)
C24	C35	1.4886(15)
C25	C27	1.5181(18)
C29	C30	1.3944(16)
C29	C34	1.3893(17)
C30	C31	1.3925(17)
C31	C32	1.3793(19)
C32	C33	1.3840(19)
C33	C34	1.3900(17)
C35	C36	1.3948(16)
C35	C40	1.3999(15)
C36	C37	1.3844(16)
C37	C38	1.3944(16)
C38	C39	1.3905(16)
C38	C41	1.4840(15)
C39	C40	1.3838(16)

Table S14: Bond Angles in ° for **16b**.

Atom	Atom	Atom	Angle/°
C1	O1	C4	108.05(8)
C20	O3	C21	116.50(9)
C4	N1	C5	105.40(9)
O1	C1	C2	115.73(9)
O1	C1	C3	117.86(9)
O1	C1	C5	106.59(9)
C2	C1	C3	61.79(7)
C2	C1	C5	125.60(9)
C3	C1	C5	124.07(9)
C1	C2	C3	59.37(7)
C1	C2	C7	116.71(9)
C7	C2	C3	116.45(9)
C8	C2	C1	121.88(9)
C8	C2	C3	122.55(9)
C8	C2	C7	111.15(9)
C1	C3	C2	58.84(7)
C14	C3	C1	125.40(9)
C14	C3	C2	124.16(9)
O1	C4	C6	106.07(9)
N1	C4	O1	109.36(9)
N1	C4	C6	111.16(10)
N1	C5	C1	103.50(9)
F1	C6	F2	106.75(10)
F1	C6	F3	107.32(10)
F1	C6	C4	111.37(10)
F2	C6	C4	112.29(10)
F3	C6	F2	107.01(10)
F3	C6	C4	111.79(10)
F4	C7	C2	111.30(9)
F5	C7	F4	106.40(9)
F5	C7	C2	113.61(9)
F6	C7	F4	106.40(9)
F6	C7	F5	106.30(9)
F6	C7	C2	112.35(9)
C9	C8	C2	120.22(10)
C13	C8	C2	120.06(10)
C13	C8	C9	119.40(10)
C10	C9	C8	120.15(11)
C11	C10	C9	119.99(12)
C10	C11	C12	120.28(11)
C11	C12	C13	120.01(12)
C12	C13	C8	120.17(11)
C15	C14	C3	117.23(9)
C15	C14	C19	118.19(10)
C19	C14	C3	124.54(10)
C16	C15	C14	121.65(10)
C15	C16	C17	119.77(10)
C16	C17	C20	122.22(10)
C18	C17	C16	119.04(10)
C18	C17	C20	118.71(9)
C19	C18	C17	121.04(10)
C18	C19	C14	120.28(10)
O2	C20	O3	123.62(10)
O2	C20	C17	124.18(10)
O3	C20	C17	112.19(9)
C22	O4	C25	107.85(9)

Atom	Atom	Atom	Angle/°
C41	O6	C42	115.56(10)
C25	N2	C26	105.32(9)
O4	C22	C23	115.18(9)
O4	C22	C24	118.73(9)
O4	C22	C26	105.60(9)
C23	C22	C24	61.77(7)
C23	C22	C26	127.63(10)
C24	C22	C26	123.35(10)
C22	C23	C24	59.36(7)
C22	C23	C28	117.50(10)
C28	C23	C24	115.75(9)
C29	C23	C22	121.56(9)
C29	C23	C24	121.63(9)
C29	C23	C28	111.85(9)
C22	C24	C23	58.88(7)
C35	C24	C22	126.21(10)
C35	C24	C23	123.47(9)
O4	C25	N2	108.98(10)
O4	C25	C27	106.06(9)
N2	C25	C27	111.92(11)
N2	C26	C22	102.68(9)
F7	C27	F8	107.10(11)
F7	C27	F9	107.07(11)
F7	C27	C25	111.98(11)
F8	C27	C25	112.23(11)
F9	C27	F8	107.44(11)
F9	C27	C25	110.75(11)

Atom	Atom	Atom	Angle/°
F10	C28	C23	111.38(9)
F11	C28	F10	106.39(10)
F11	C28	C23	113.55(9)
F12	C28	F10	106.54(9)
F12	C28	F11	106.36(10)
F12	C28	C23	112.15(10)
C30	C29	C23	120.46(10)
C34	C29	C23	120.21(10)
C34	C29	C30	119.15(10)
C31	C30	C29	120.21(11)
C32	C31	C30	119.99(12)
C31	C32	C33	120.32(11)
C32	C33	C34	119.82(12)
C29	C34	C33	120.51(11)
C36	C35	C24	117.54(9)
C36	C35	C40	118.32(10)
C40	C35	C24	124.12(10)
C37	C36	C35	121.71(10)
C36	C37	C38	119.43(11)
C37	C38	C41	122.21(10)
C39	C38	C37	119.34(10)
C39	C38	C41	118.44(10)
C40	C39	C38	121.04(10)
C39	C40	C35	120.10(11)
O5	C41	O6	123.39(11)
O5	C41	C38	124.02(11)
O6	C41	C38	112.58(9)

Table S15: Torsion Angles in ° for **16b**.

Atom	Atom	Atom	Atom	Angle/°
O1	C1	C2	C3	109.28(10)
O1	C1	C2	C7	-144.36(10)
O1	C1	C2	C8	-2.32(14)
O1	C1	C3	C2	-105.88(10)
O1	C1	C3	C14	6.16(15)
O1	C1	C5	N1	-22.40(11)
O1	C4	C6	F1	-176.19(9)
O1	C4	C6	F2	64.14(12)
O1	C4	C6	F3	-56.14(12)
N1	C4	C6	F1	65.03(12)
N1	C4	C6	F2	-54.64(12)
N1	C4	C6	F3	-174.92(9)
C1	O1	C4	N1	7.70(12)
C1	O1	C4	C6	-112.26(10)
C1	C2	C3	C14	-114.07(12)
C1	C2	C7	F4	77.38(12)
C1	C2	C7	F5	-42.70(14)
C1	C2	C7	F6	-163.42(9)
C1	C2	C8	C9	-56.26(14)
C1	C2	C8	C13	130.31(11)
C1	C3	C14	C15	158.17(10)
C1	C3	C14	C19	-19.55(17)
C2	C1	C3	C14	112.04(11)
C2	C1	C5	N1	-162.77(10)
C2	C3	C14	C15	-128.38(11)
C2	C3	C14	C19	53.90(16)
C2	C8	C9	C10	-172.75(10)
C2	C8	C13	C12	172.50(10)

Atom	Atom	Atom	Atom	Angle/˚
C3	C1	C2	C7	106.36(10)
C3	C1	C2	C8	-111.60(11)
C3	C1	C5	N1	119.80(11)
C3	C2	C7	F4	144.63(10)
C3	C2	C7	F5	24.55(14)
C3	C2	C7	F6	-96.17(11)
C3	C2	C8	C9	-127.91(11)
C3	C2	C8	C13	58.66(14)
C3	C14	C15	C16	-175.98(10)
C3	C14	C19	C18	176.66(10)
C4	O1	C1	C2	154.30(9)
C4	O1	C1	C3	-135.51(10)
C4	O1	C1	C5	9.44(11)
C4	N1	C5	C1	26.59(12)
C5	N1	C4	O1	-22.13(13)
C5	N1	C4	C6	94.66(11)
C5	C1	C2	C3	-113.44(12)
C5	C1	C2	C7	-7.08(16)
C5	C1	C2	C8	134.96(11)
C5	C1	C3	C2	115.76(12)
C5	C1	C3	C14	-132.20(11)
C7	C2	C3	C1	-106.78(11)
C7	C2	C3	C14	139.15(11)
C7	C2	C8	C9	87.64(12)
C7	C2	C8	C13	-85.79(12)
C8	C2	C3	C1	110.50(11)
C8	C2	C3	C14	-3.57(16)
C8	C2	C7	F4	-68.57(12)
C8	C2	C7	F5	171.36(9)
C8	C2	C7	F6	50.64(12)
C8	C9	C10	C11	0.18(17)
C9	C8	C13	C12	-0.98(16)
C9	C10	C11	C12	-0.84(18)
C10	C11	C12	C13	0.58(18)
C11	C12	C13	C8	0.34(18)
C13	C8	C9	C10	0.73(16)
C14	C15	C16	C17	-0.92(17)
C15	C14	C19	C18	-1.04(16)
C15	C16	C17	C18	-0.92(16)
C15	C16	C17	C20	177.14(10)
C16	C17	C18	C19	1.77(16)
C16	C17	C20	O2	-179.83(11)
C16	C17	C20	O3	-0.53(15)
C17	C18	C19	C14	-0.78(17)
C18	C17	C20	O2	-1.76(16)
C18	C17	C20	O3	177.54(10)
C19	C14	C15	C16	1.89(16)
C20	C17	C18	C19	-176.37(10)
C21	O3	C20	O2	2.19(16)
C21	O3	C20	C17	-177.11(10)
O4	C22	C23	C24	-110.51(11)
O4	C22	C23	C28	144.37(10)
O4	C22	C23	C29	0.07(15)
O4	C22	C24	C23	104.86(11)
O4	C22	C24	C35	-6.02(16)
O4	C22	C26	N2	29.98(11)
O4	C25	C27	F7	52.01(13)
O4	C25	C27	F8	-68.48(13)
O4	C25	C27	F9	171.45(10)
N2	C25	C27	F7	170.75(10)
N2	C25	C27	F8	50.25(14)
N2	C25	C27	F9	-69.82(14)

Atom	Atom	Atom	Atom	Angle/°
C22	O4	C25	N2	1.00(12)
C22	O4	C25	C27	121.66(10)
C22	C23	C24	C35	115.34(12)
C22	C23	C28	F10	-83.33(12)
C22	C23	C28	F11	36.75(14)
C22	C23	C28	F12	157.36(10)
C22	C23	C29	C30	52.89(15)
C22	C23	C29	C34	-132.05(11)
C22	C24	C35	C36	-160.77(10)
C22	C24	C35	C40	17.94(17)
C23	C22	C24	C35	-110.88(12)
C23	C22	C26	N2	170.60(11)
C23	C24	C35	C36	125.72(11)
C23	C24	C35	C40	-55.57(16)
C23	C29	C30	C31	174.73(10)
C23	C29	C34	C33	-174.43(10)
C24	C22	C23	C28	-105.12(11)
C24	C22	C23	C29	110.58(11)
C24	C22	C26	N2	-111.38(12)
C24	C23	C28	F10	-150.57(10)
C24	C23	C28	F11	-30.49(14)
C24	C23	C28	F12	90.12(12)
C24	C23	C29	C30	123.97(12)
C24	C23	C29	C34	-60.96(14)
C24	C35	C36	C37	176.25(10)
C24	C35	C40	C39	-177.33(10)
C25	O4	C22	C23	-165.67(9)
C25	O4	C22	C24	124.11(10)
C25	O4	C22	C26	-19.40(11)
C25	N2	C26	C22	-28.99(12)
C26	N2	C25	O4	18.41(13)
C26	N2	C25	C27	-98.58(11)
C26	C22	C23	C24	111.96(13)
C26	C22	C23	C28	6.84(17)
C26	C22	C23	C29	-137.46(12)
C26	C22	C24	C23	-118.44(12)
C26	C22	C24	C35	130.68(12)
C28	C23	C24	C22	108.06(11)
C28	C23	C24	C35	-136.59(11)
C28	C23	C29	C30	-93.22(12)
C28	C23	C29	C34	81.85(13)
C29	C23	C24	C22	-110.47(11)
C29	C23	C24	C35	4.88(16)
C29	C23	C28	F10	64.28(13)
C29	C23	C28	F11	-175.64(10)
C29	C23	C28	F12	-55.03(13)
C29	C30	C31	C32	-0.15(17)
C30	C29	C34	C33	0.70(17)
C30	C31	C32	C33	0.39(18)
C31	C32	C33	C34	-0.08(19)
C32	C33	C34	C29	-0.47(18)
C34	C29	C30	C31	-0.38(16)
C35	C36	C37	C38	1.40(16)
C36	C35	C40	C39	1.38(17)
C36	C37	C38	C39	0.93(16)
C36	C37	C38	C41	-177.86(10)
C37	C38	C39	C40	-2.08(17)
C37	C38	C41	O5	-171.55(11)
C37	C38	C41	O6	9.51(15)
C38	C39	C40	C35	0.91(18)
C39	C38	C41	O5	9.65(17)
C39	C38	C41	O6	-169.28(10)

Atom	Atom	Atom	Atom	Angle/°
C40	C35	C36	C37	-2.54(16)
C41	C38	C39	C40	176.75(10)
C42	O6	C41	O5	-2.19(16)
C42	O6	C41	C38	176.75(10)

Table S16: Hydrogen Fractional Atomic Coordinates (×10[4]) and Equivalent Isotropic Displacement Parameters (Å^2×10^3) for **16b.** U_{eq} is defined as 1/3 of the trace of the orthogonalised U_{ij}.

Atom	x	y	z	U_{eq}
H1	11297(11)	4173(6)	5256(14)	42(4)
H3	9450.46	3111.1	5720.83	30
H4	11566.17	4039.01	3418.5	36
H5A	9540.75	4471.53	5115.61	35
H5B	9984.88	4057.01	6071.5	35
H9	8747.04	4071.75	2192.88	32
H10	8066.88	3890.94	335.33	39
H11	7384.32	3052.76	-15.47	43
H12	7406.56	2384.23	1463.35	44
H13	8094.19	2557.98	3318.97	36
H15	9657.51	2182.55	5843.62	30
H16	10345.64	1376.42	5266.54	31
H18	11006.57	2063.95	2089.24	31
H19	10349.29	2877.08	2686.49	32
H21A	11550.89	137.62	2979.96	60
H21B	11571.94	-16.86	4373.99	60
H21C	12373.76	335.81	3811.44	60
H2	6298(11)	4192(7)	690(15)	48(4)
H24	4474.56	3248.38	246.21	31
H25	6596.9	4042.55	2524.77	38
H26A	4969.43	4194.15	-78.97	39
H26B	4630.85	4621.73	918.38	39
H30	3780.93	4168.31	3805.04	34
H31	3138.15	3958.02	5661.43	40
H32	2443.26	3120.68	5951.1	44
H33	2396.85	2484.12	4402.46	45
H34	3048.76	2687.63	2551.53	37
H36	4638.56	2312.63	140.27	31
H37	5301.64	1500	745.57	32
H39	5959.76	2190.78	3932.58	34
H40	5339.96	3011.27	3301.39	35
H42A	6827.04	184.46	1583.74	62
H42B	6392.19	213	2892.96	62
H42C	7333.99	510.1	2632.88	62

References

(1) Sheldrick, G. M. SHELXT – Integrated Space-Group and Crystal-Structure Determination. *Acta Crystallogr. Sect. Found. Adv.* **2015**, *71* (1), 3–8. https://doi.org/10.1107/S2053273314026370.

(2) Sheldrick, G. M. Crystal Structure Refinement with SHELXL. *Acta Crystallogr. Sect. C Struct. Chem.* **2015**, *71* (1), 3–8. https://doi.org/10.1107/S2053229614024218.

(3) Zalesskiy, S. S.; Ananikov, V. P. Pd2(Dba)3 as a Precursor of Soluble Metal Complexes and Nanoparticles: Determination of Palladium Active Species for Catalysis and Synthesis. *Organometallics* **2012**, *31* (6), 2302–2309. https://doi.org/10.1021/om201217r.

(4) Muriel, B.; Waser, J. Azide Radical Initiated Ring Opening of Cyclopropenes Leading to Alkenyl Nitriles and Polycyclic Aromatic Compounds. *Angew. Chem. Int. Ed.* **2021**, *60* (8), 4075–4079. https://doi.org/10.1002/anie.202013516.

(5) Biswas, S.; Bolm, C. Rhodium(II)-Catalyzed N–H Insertions of Carbenes under Mechanochemical Conditions. *Org. Lett.* **2024**, *26* (7), 1511–1516. https://doi.org/10.1021/acs.orglett.4c00216.

(6) Ye, F.; Wang, C.; Zhang, Y.; Wang, J. Synthesis of Aryldiazoacetates through Palladium(0)-Catalyzed Deacylative Cross-Coupling of Aryl Iodides with Acyldiazoacetates. *Angew. Chem. Int. Ed.* **2014**, *53* (43), 11625–11628. https://doi.org/10.1002/anie.201407653.

(7) Kidonakis, M.; Stratakis, M. Au Nanoparticle-Catalyzed Insertion of Carbenes from α-Diazocarbonyl Compounds into Hydrosilanes. *Org. Lett.* **2018**, *20* (13), 4086–4089. https://doi.org/10.1021/acs.orglett.8b01638.

(8) Smyrnov, V.; Muriel, B.; Waser, J. Synthesis of Quinolines via the Metal-Free Visible-Light-Mediated Radical Azidation of Cyclopropenes. *Org. Lett.* **2021**, *23* (14), 5435–5439. https://doi.org/10.1021/acs.orglett.1c01775.

(9) Pisella, G.; Gagnebin, A.; Waser, J. Copper-Catalyzed Oxyvinylation of Diazo Compounds. *Org. Lett.* **2020**, *22* (10), 3884–3889. https://doi.org/10.1021/acs.orglett.0c01150.

(10) Liu, Y.; De Nisi, A.; Cerveri, A.; Monari, M.; Bandini, M. Nickel-Catalyzed Synthesis of Stereochemically Defined Enamides via Bi- and Tricomponent Coupling Reaction. *Org. Lett.* **2017**, *19* (19), 5034–5037. https://doi.org/10.1021/acs.orglett.7b02166.

(11) Robles-Machín, R.; Adrio, J.; Carretero, J. C. Gold-Catalyzed Synthesis of Alkylidene 2-Oxazolidinones and 1,3-Oxazin-2-Ones. *J. Org. Chem.* **2006**, *71* (13), 5023–5026. https://doi.org/10.1021/jo060520y.

(12) Molander, G. A.; Shin, I. Suzuki–Miyaura Cross-Coupling Reactions of Potassium Boc-Protected Aminomethyltrifluoroborate with Aryl and Hetaryl Mesylates. *Org. Lett.* **2012**, *14* (12), 3138–3141. https://doi.org/10.1021/ol301221p.

(13) Charlesworth, N. G.; Arunprasath, D.; Graham, M. A.; Argent, S. P.; Datsenko, O. P.; Mykhailiuk, P. K.; Denton, R. M. Modular Synthesis of Cyclic β-Difluoroamines. *Chem. Commun.* **2024**, *60* (60), 7701–7704. https://doi.org/10.1039/D4CC00640B.

(14) Wang, H.-Y.; Qin, Y.; Li, H.; Roman, L. J.; Martásek, P.; Poulos, T. L.; Silverman, R. B. Potent and Selective Human Neuronal Nitric Oxide Synthase Inhibition by Optimization of the 2-Aminopyridine-Based Scaffold with a Pyridine Linker. *J. Med. Chem.* **2016**, *59* (10), 4913–4925. https://doi.org/10.1021/acs.jmedchem.6b00273.

(15) Das, S.; Goswami, R. K. Total Synthesis of Marine Natural Products Separacenes A and B. *Org. Biomol. Chem.* **2017**, *15* (22), 4842–4850. https://doi.org/10.1039/C7OB00345E.

(16) Gensch, T.; dos Passos Gomes, G.; Friederich, P.; Peters, E.; Gaudin, T.; Pollice, R.; Jorner, K.; Nigam, A.; Lindner-D'Addario, M.; Sigman, M. S.; Aspuru-Guzik, A. A

Comprehensive Discovery Platform for Organophosphorus Ligands for Catalysis. *J. Am. Chem. Soc.* **2022**, *144* (3), 1205–1217. https://doi.org/10.1021/jacs.1c09718.
(17) Pedregosa, F.; Varoquaux, G.; Gramfort, A.; Michel, V.; Thirion, B.; Grisel, O.; Blondel, M.; Prettenhofer, P.; Weiss, R.; Dubourg, V.; Vanderplas, J.; Passos, A.; Cournapeau, D.; Brucher, M.; Perrot, M.; Duchesnay, É. Scikit-Learn: Machine Learning in Python. *J. Mach. Learn. Res.* **2011**, *12* (85), 2825–2830.
(18) Schoepfer, A. A.; Laplaza, R.; Wodrich, M. D.; Waser, J.; Corminboeuf, C. Reaction-Agnostic Featurization of Bidentate Ligands for Bayesian Ridge Regression of Enantioselectivity. *ACS Catal.* **2024**, *14* (12), 9302–9312. https://doi.org/10.1021/acscatal.4c02452.
(19) O'Boyle, N. M.; Banck, M.; James, C. A.; Morley, C.; Vandermeersch, T.; Hutchison, G. R. Open Babel: An Open Chemical Toolbox. *J. Cheminformatics* **2011**, *3* (1), 33. https://doi.org/10.1186/1758-2946-3-33.
(20) Pracht, P.; Grimme, S.; Bannwarth, C.; Bohle, F.; Ehlert, S.; Feldmann, G.; Gorges, J.; Müller, M.; Neudecker, T.; Plett, C.; Spicher, S.; Steinbach, P.; Wesołowski, P. A.; Zeller, F. CREST—A Program for the Exploration of Low-Energy Molecular Chemical Space. *J. Chem. Phys.* **2024**, *160* (11), 114110. https://doi.org/10.1063/5.0197592.
(21) Bannwarth, C.; Ehlert, S.; Grimme, S. GFN2-xTB—An Accurate and Broadly Parametrized Self-Consistent Tight-Binding Quantum Chemical Method with Multipole Electrostatics and Density-Dependent Dispersion Contributions. *J. Chem. Theory Comput.* **2019**, *15* (3), 1652–1671. https://doi.org/10.1021/acs.jctc.8b01176.
(22) Frisch, M. J.; Trucks, G. W.; Schlegel, H. B.; Scuseria, G. E.; Robb, M. A.; Cheeseman, J. R.; Scalmani, G.; Barone, V.; Petersson, G. A.; Nakatsuji, H.; Li, X.; Caricato, M.; Marenich, A. V.; Bloino, J.; Janesko, B. G.; Gomperts, R.; Mennucci, B.; Hratchian, H. P.; Ortiz, J. V.; Izmaylov, A. F.; Sonnenberg, J. L.; Williams; Ding, F.; Lipparini, F.; Egidi, F.; Goings, J.; Peng, B.; Petrone, A.; Henderson, T.; Ranasinghe, D.; Zakrzewski, V. G.; Gao, J.; Rega, N.; Zheng, G.; Liang, W.; Hada, M.; Ehara, M.; Toyota, K.; Fukuda, R.; Hasegawa, J.; Ishida, M.; Nakajima, T.; Honda, Y.; Kitao, O.; Nakai, H.; Vreven, T.; Throssell, K.; Montgomery Jr., J. A.; Peralta, J. E.; Ogliaro, F.; Bearpark, M. J.; Heyd, J. J.; Brothers, E. N.; Kudin, K. N.; Staroverov, V. N.; Keith, T. A.; Kobayashi, R.; Normand, J.; Raghavachari, K.; Rendell, A. P.; Burant, J. C.; Iyengar, S. S.; Tomasi, J.; Cossi, M.; Millam, J. M.; Klene, M.; Adamo, C.; Cammi, R.; Ochterski, J. W.; Martin, R. L.; Morokuma, K.; Farkas, O.; Foresman, J. B.; Fox, D. J. Gaussian 16 Rev. C.01, 2016.
(23) Chai, J.-D.; Head-Gordon, M. Systematic Optimization of Long-Range Corrected Hybrid Density Functionals. *J. Chem. Phys.* **2008**, *128* (8), 084106. https://doi.org/10.1063/1.2834918.
(24) Weigend, F.; Ahlrichs, R. Balanced Basis Sets of Split Valence, Triple Zeta Valence and Quadruple Zeta Valence Quality for H to Rn: Design and Assessment of Accuracy. *Phys. Chem. Chem. Phys.* **2005**, *7* (18), 3297–3305. https://doi.org/10.1039/B508541A.
(25) Weigend, F. Accurate Coulomb-Fitting Basis Sets for H to Rn. *Phys. Chem. Chem. Phys.* **2006**, *8* (9), 1057–1065. https://doi.org/10.1039/B515623H.

Copies of NMR Spectra

Cyclopropenes

^{1}H NMR Spectrum of 3a (400 MHz, $CDCl_3$)

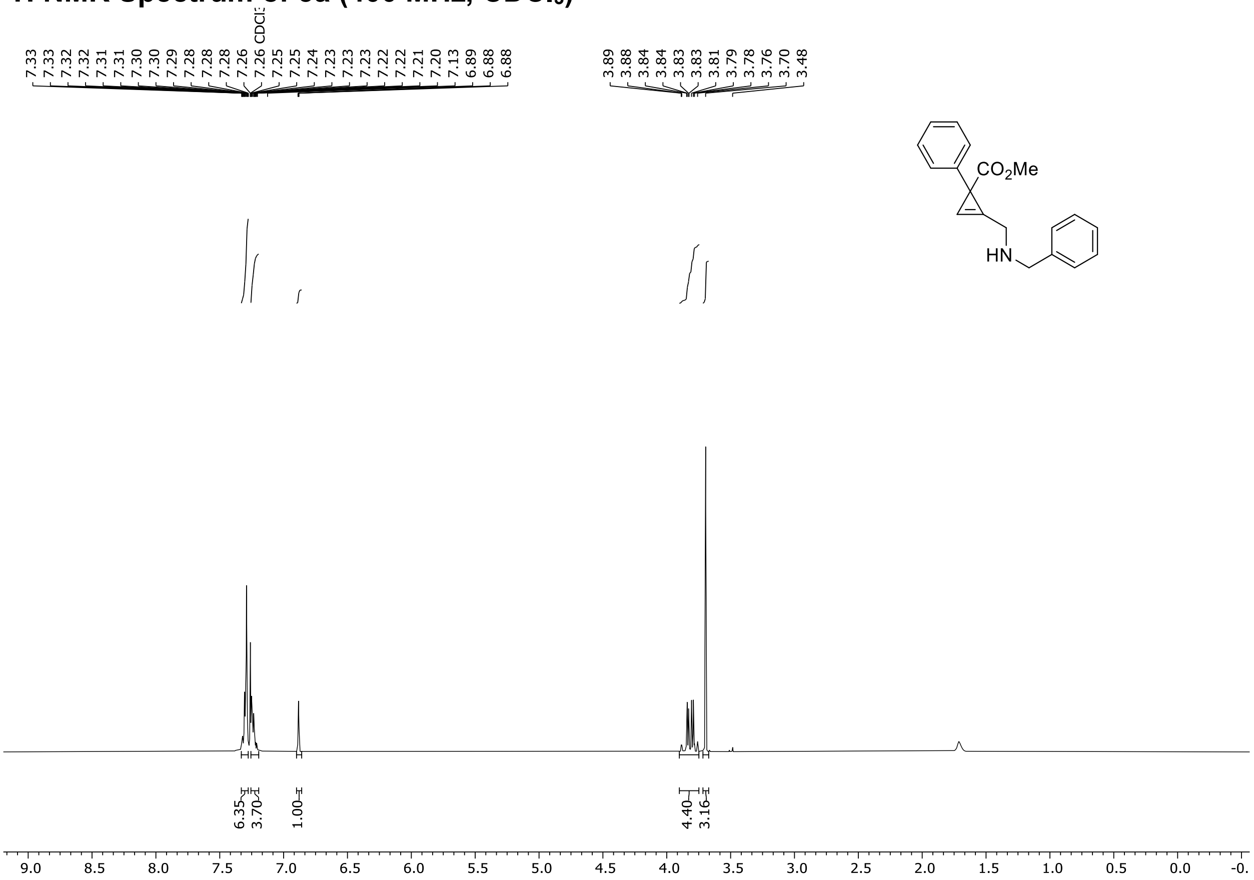

^{13}C NMR Spectrum of 3a (101 MHz, $CDCl_3$)

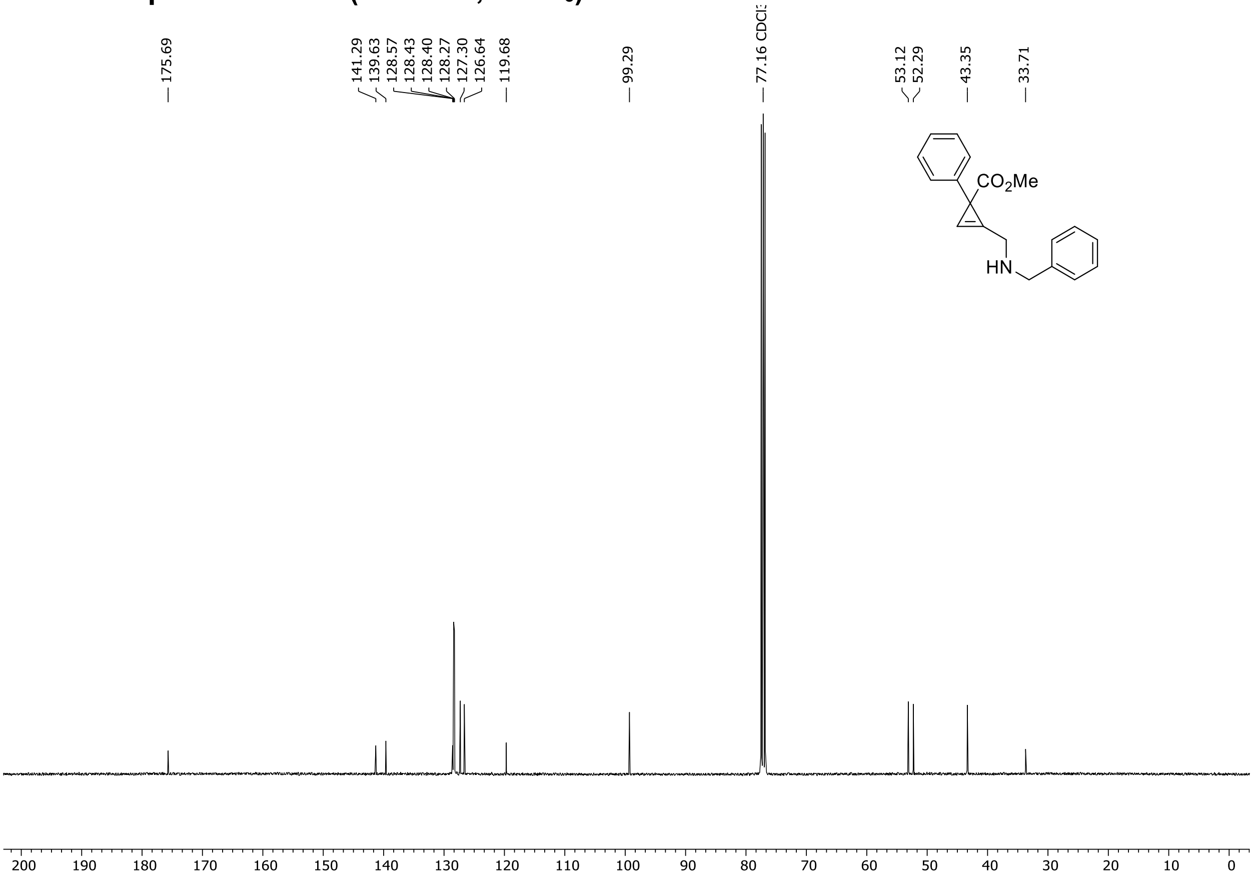

^{1}H NMR Spectrum of 3b (400 MHz, $CDCl_3$)

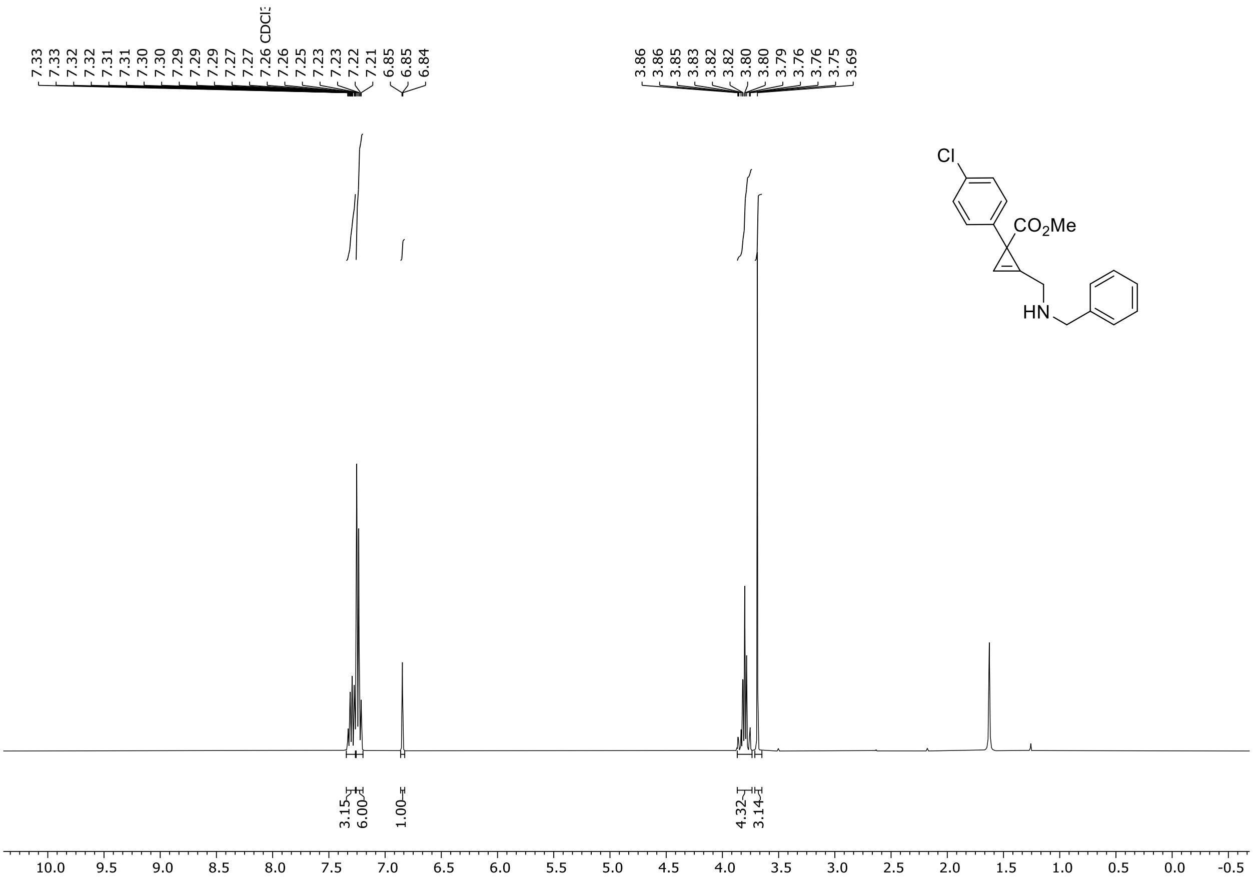

^{13}C NMR Spectrum of 3b (101 MHz, $CDCl_3$)

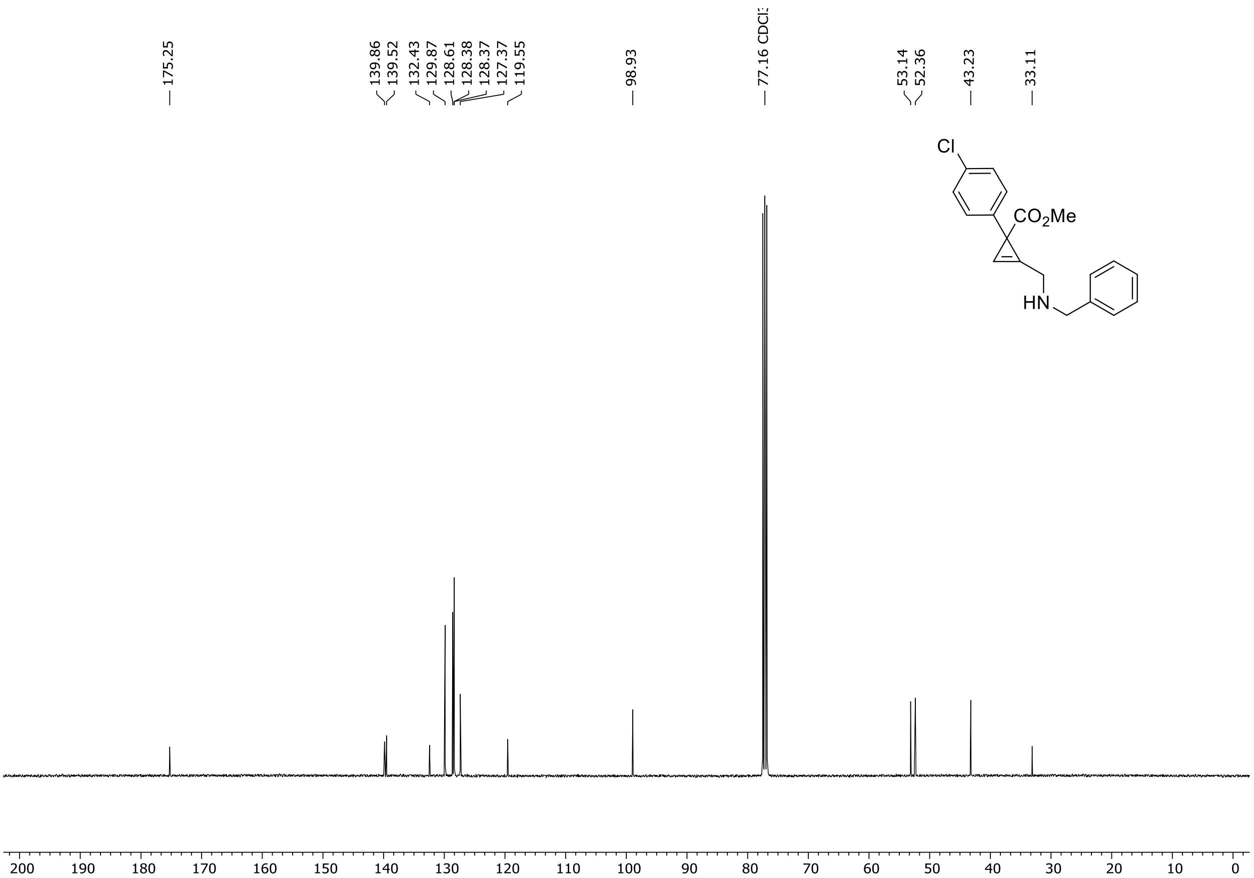

^{1}H NMR Spectrum of 3c (400 MHz, $CDCl_3$)

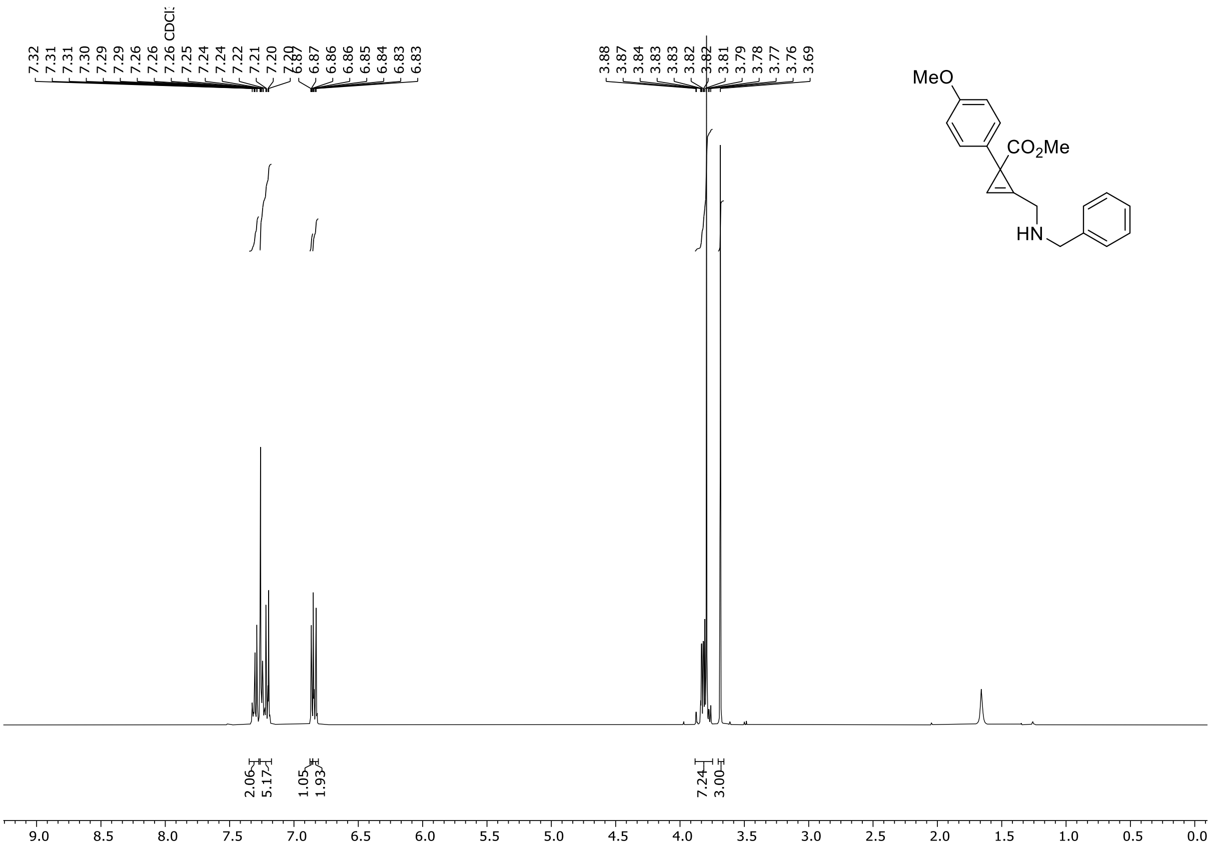

^{13}C NMR Spectrum of 3c (101 MHz, $CDCl_3$)

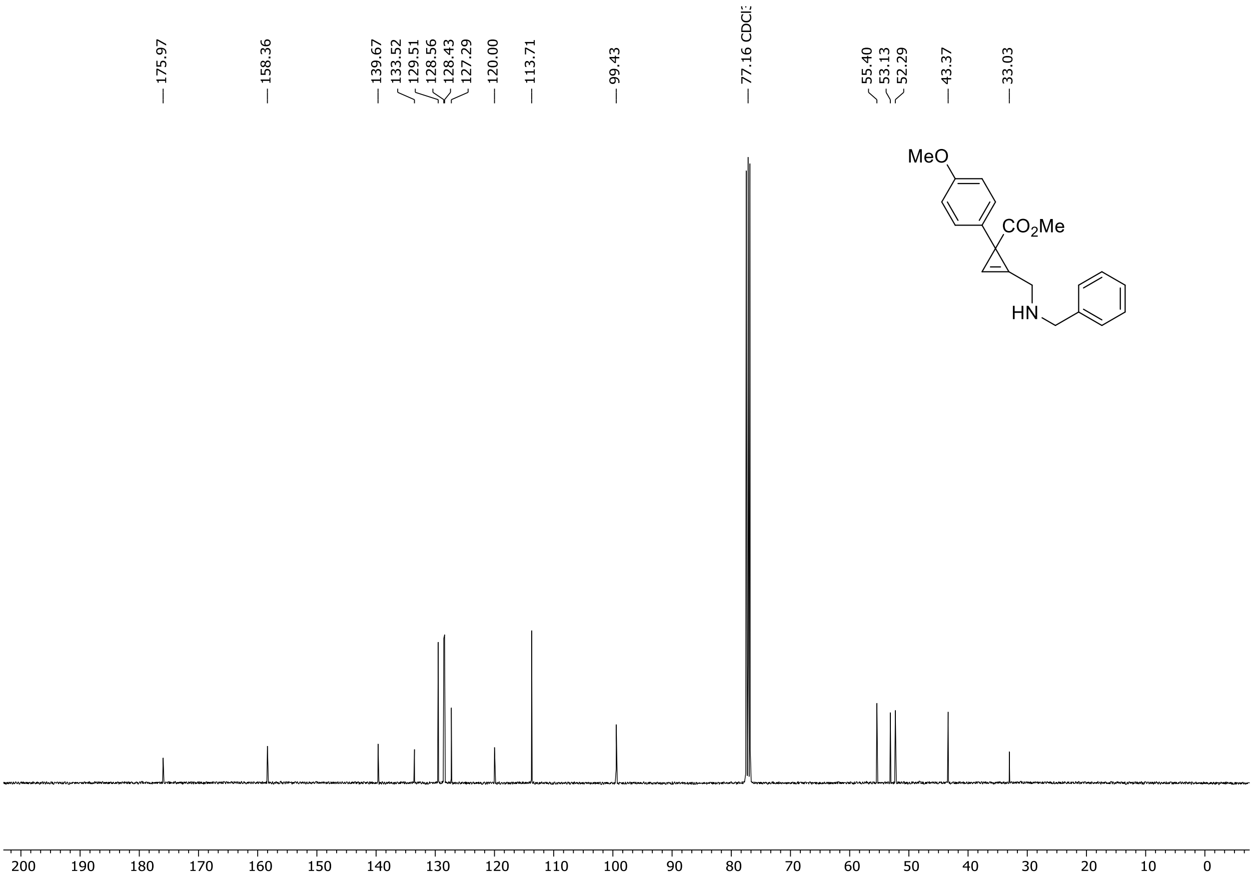

^{1}H NMR Spectrum of 3d (400 MHz, CDCl$_3$)

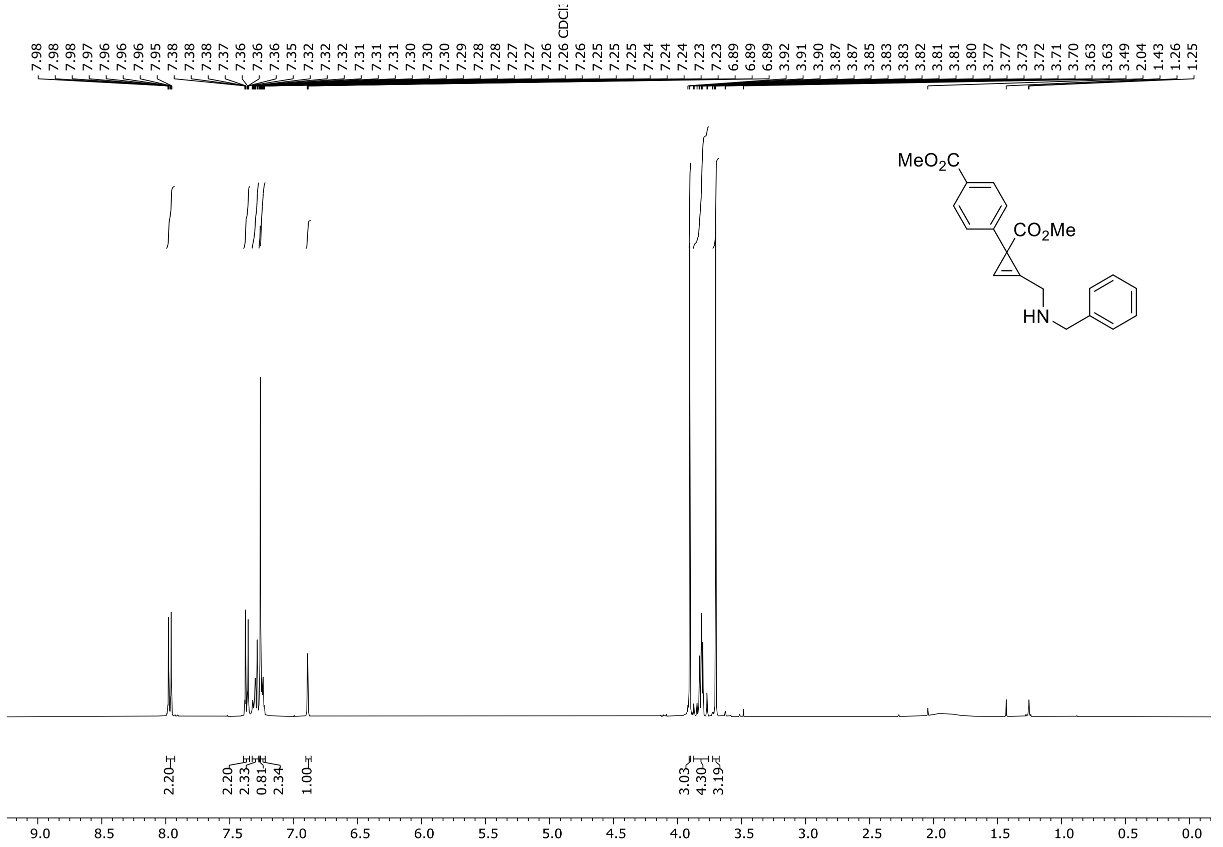

^{13}C NMR Spectrum of 3d (101 MHz, CDCl$_3$)

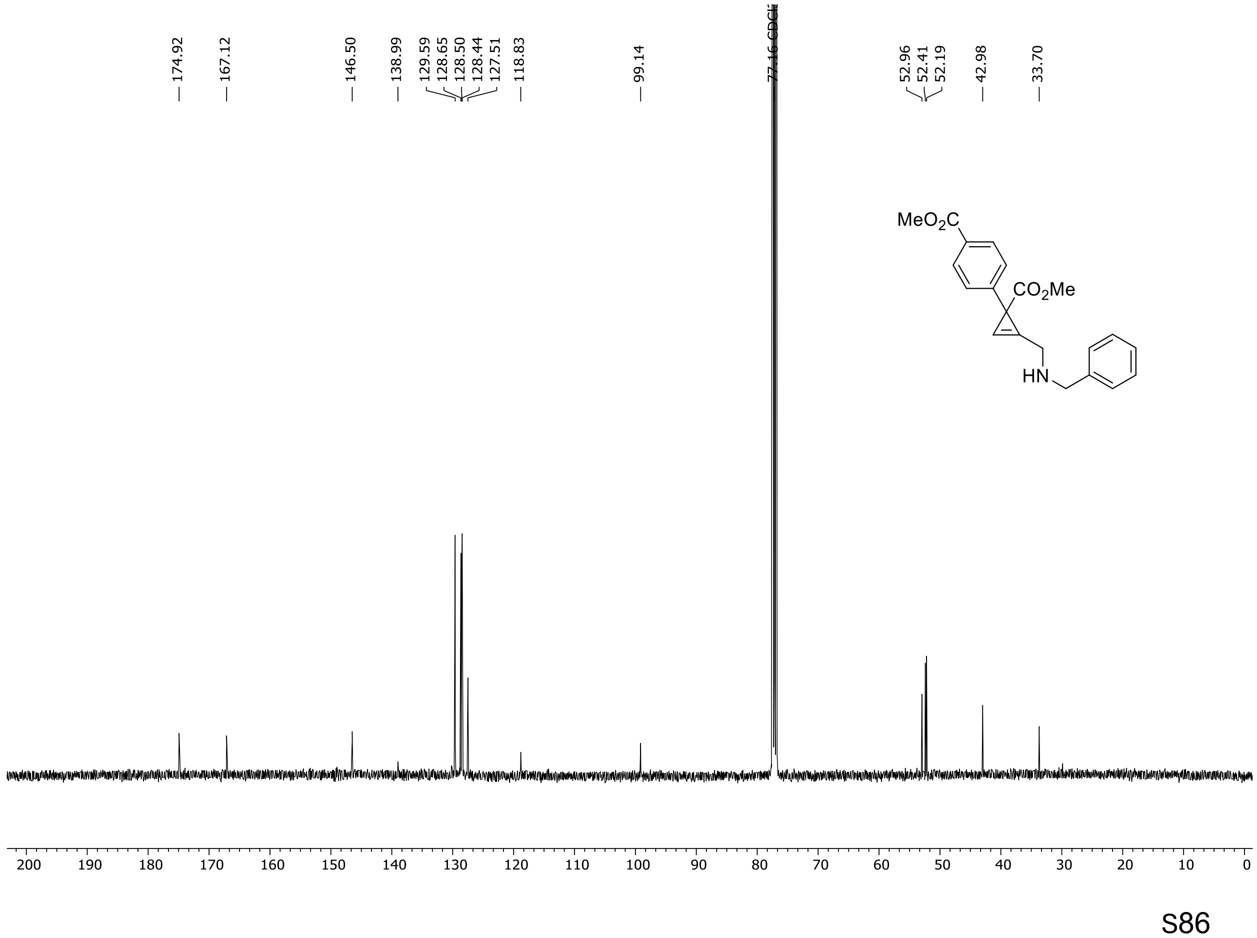

^1H NMR Spectrum of 3e (400 MHz, $CDCl_3$)

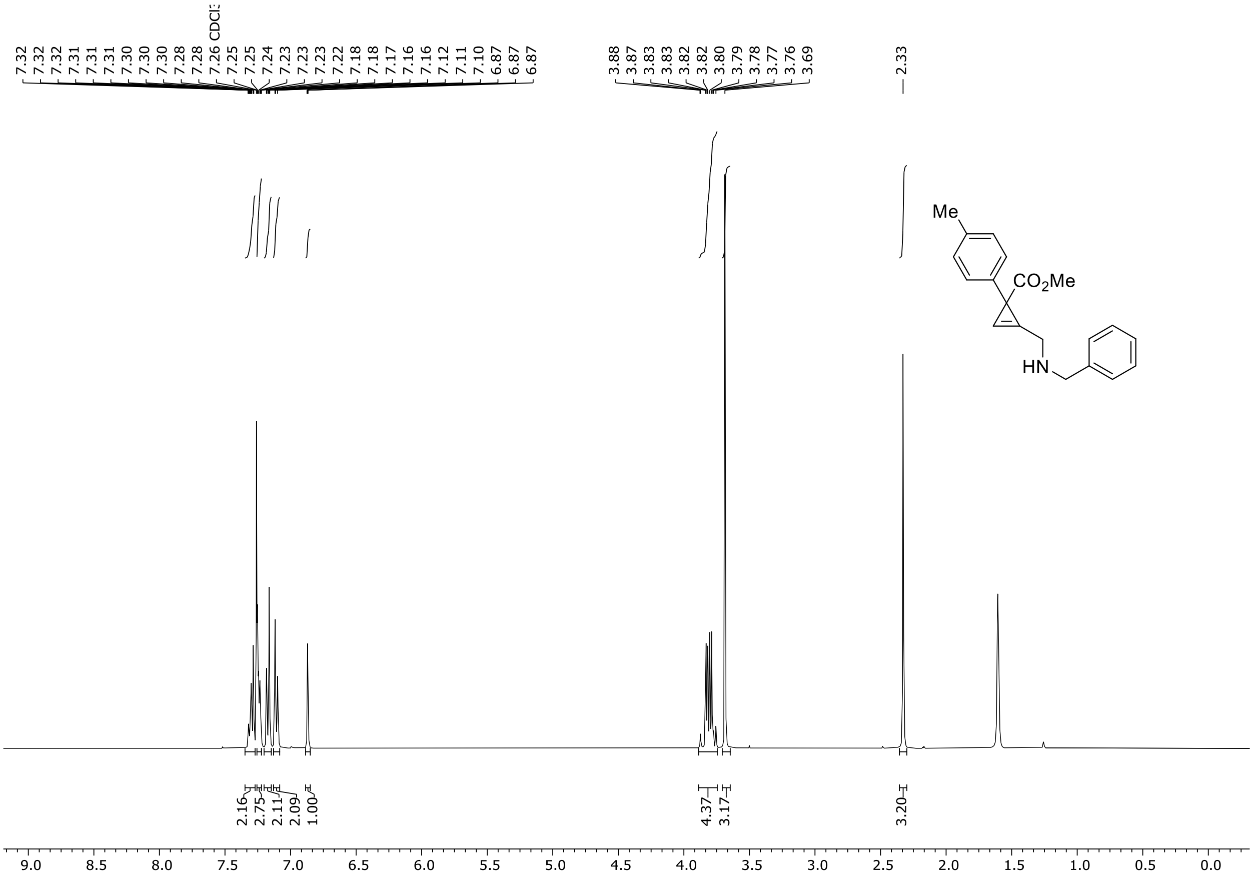

^{13}C NMR Spectrum of 3e (101 MHz, $CDCl_3$)

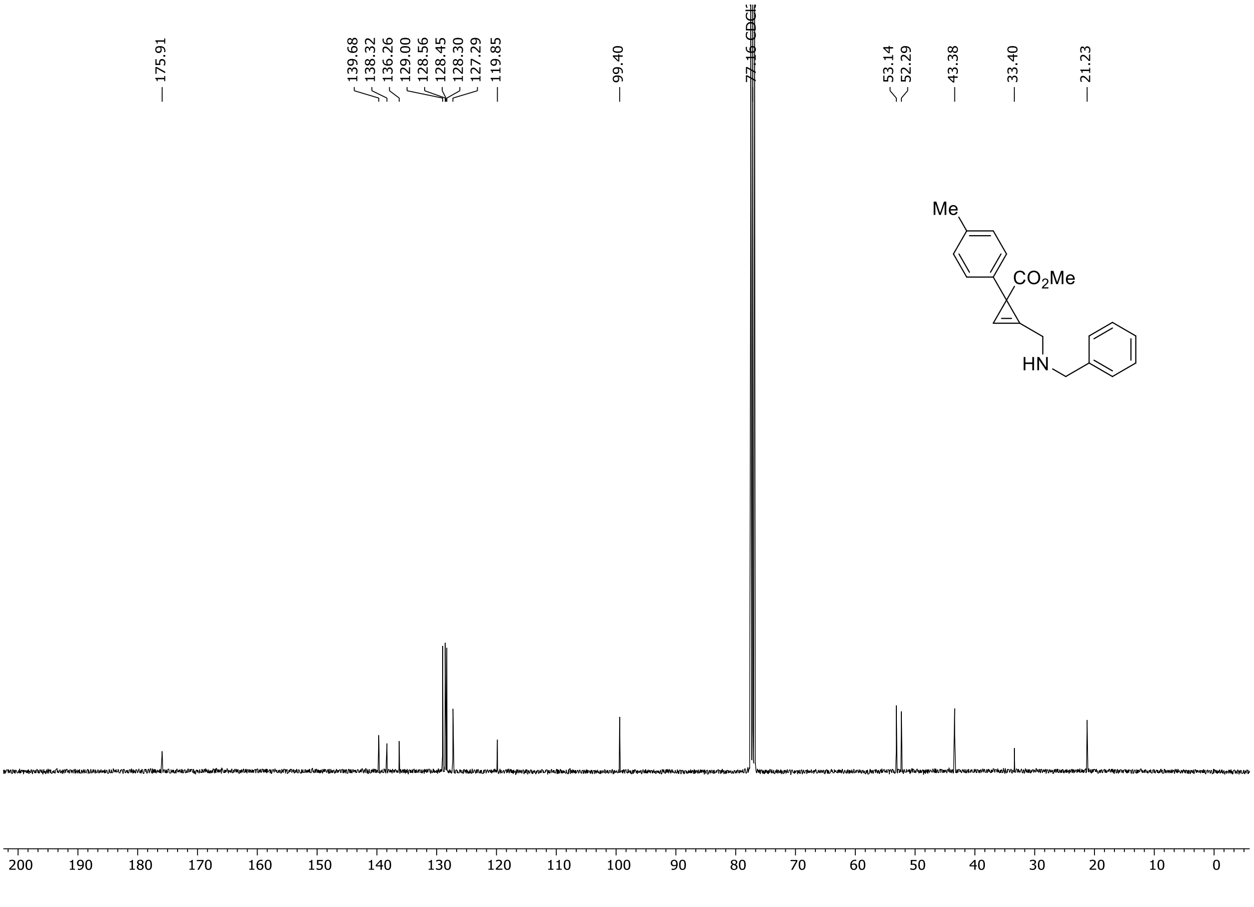

^{1}H NMR Spectrum of 3f (400 MHz, $CDCl_3$)

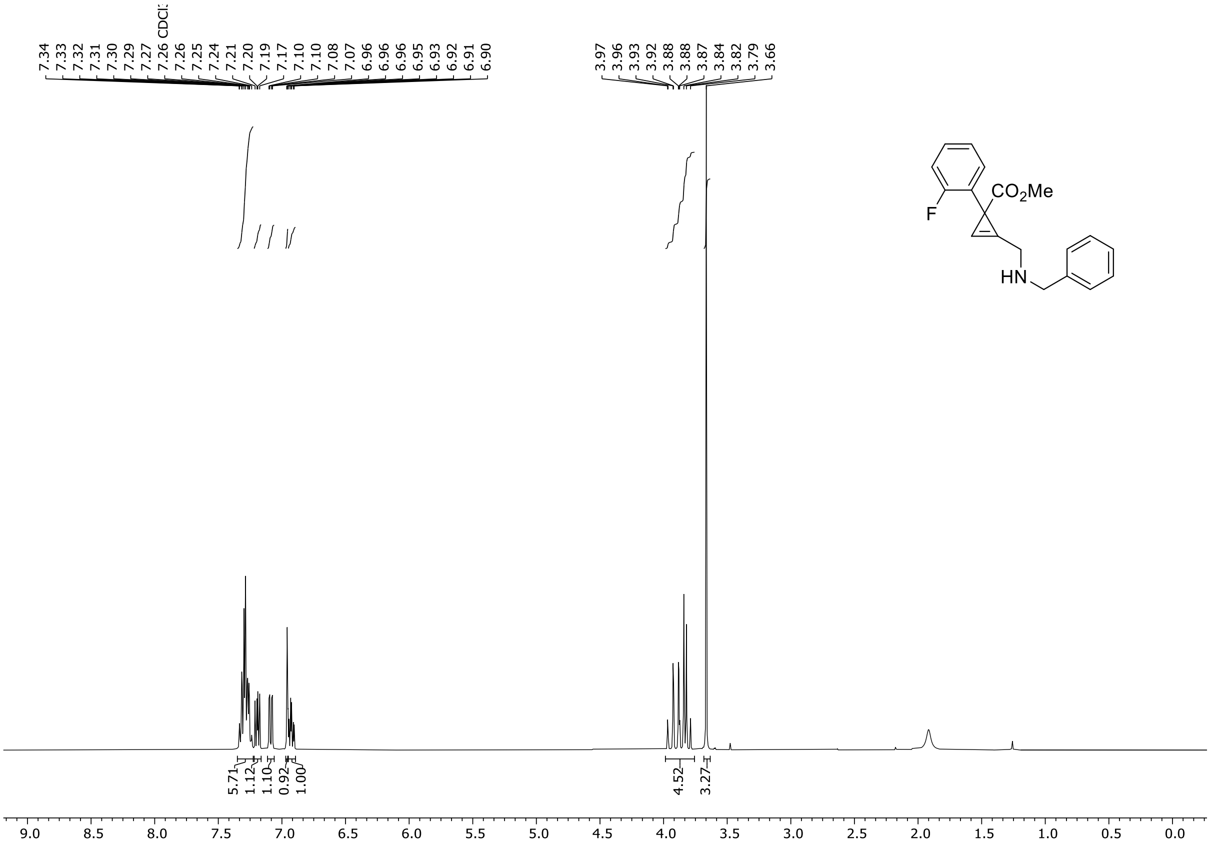

^{13}C NMR Spectrum of 3f (101 MHz, $CDCl_3$)

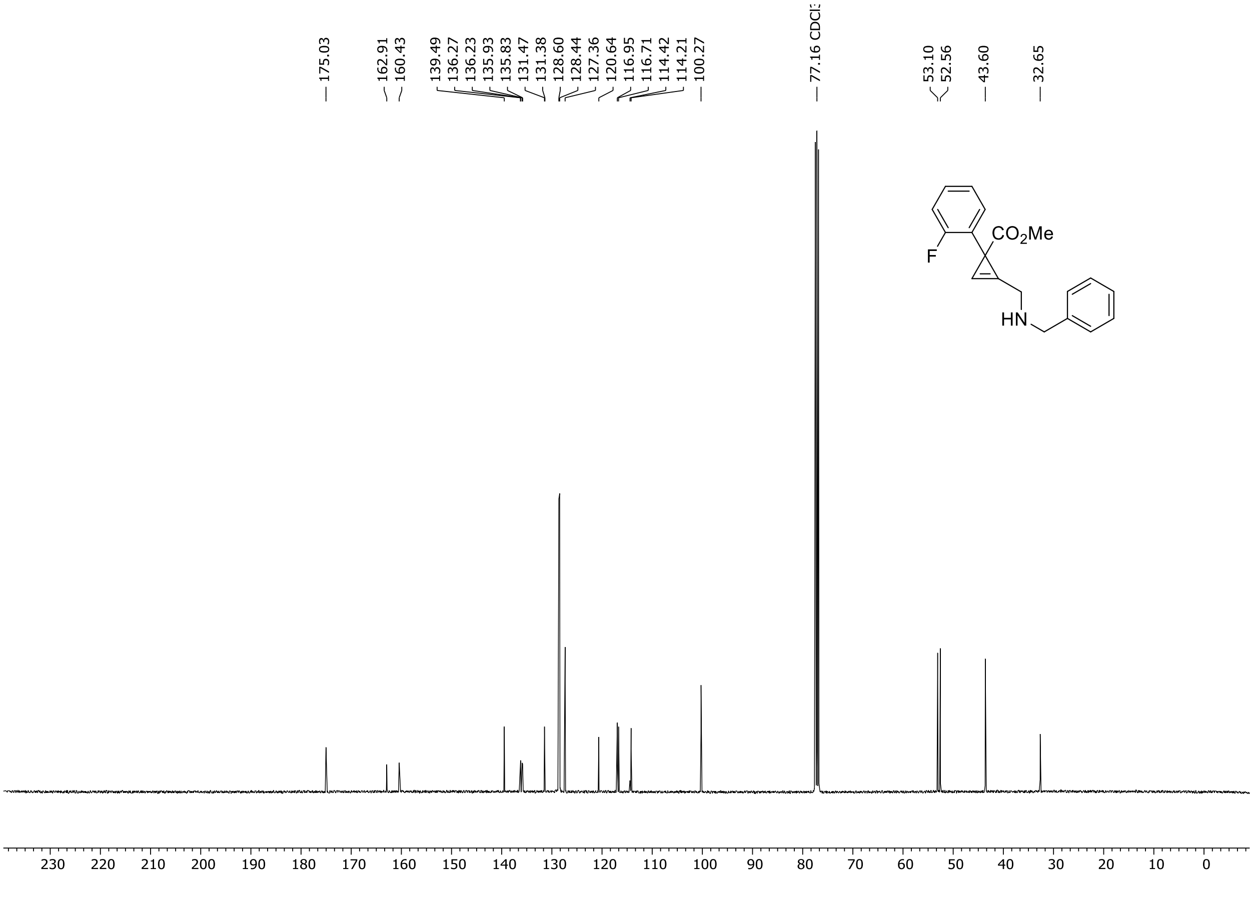

^{19}F NMR Spectrum of 3g (376 MHz, $CDCl_3$)

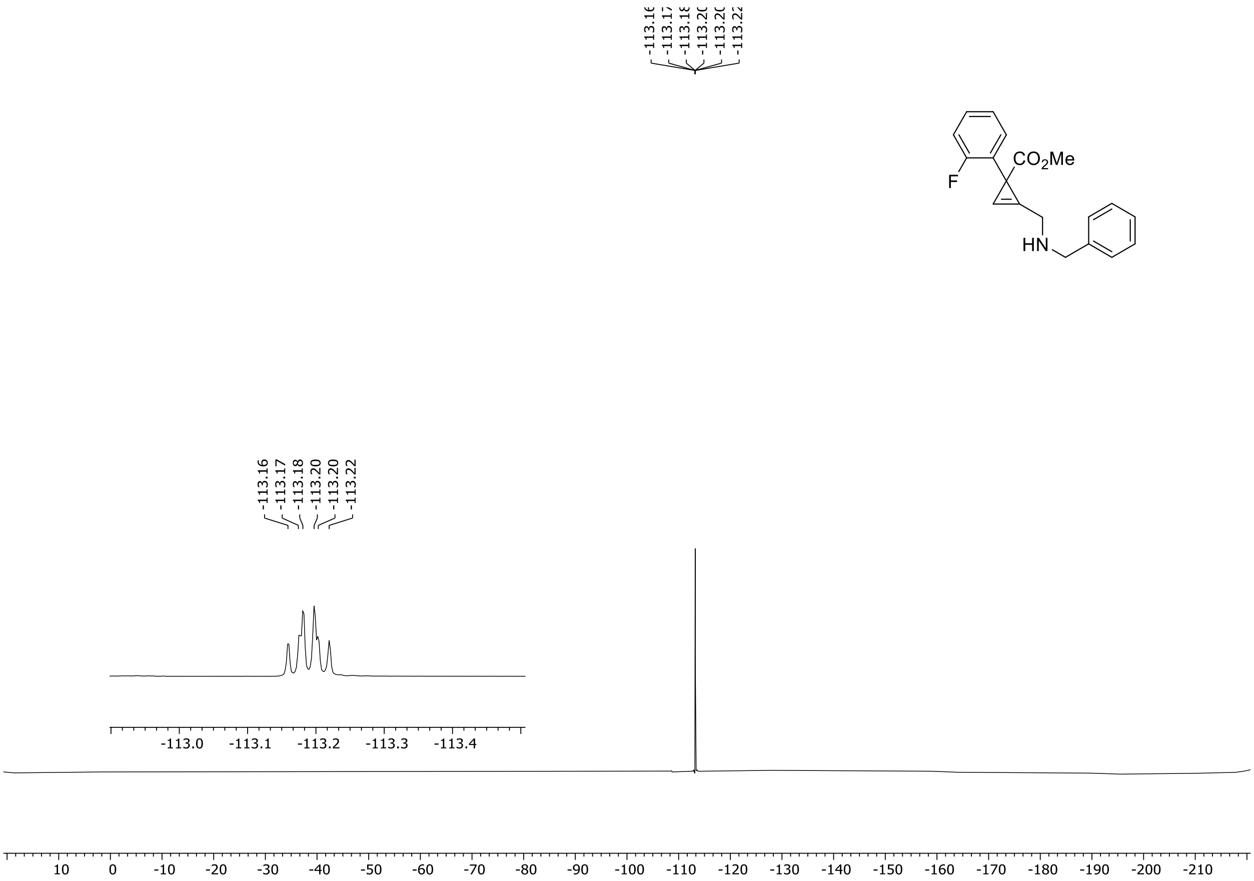

^{1}H NMR Spectrum of 3g (400 MHz, $CDCl_3$)

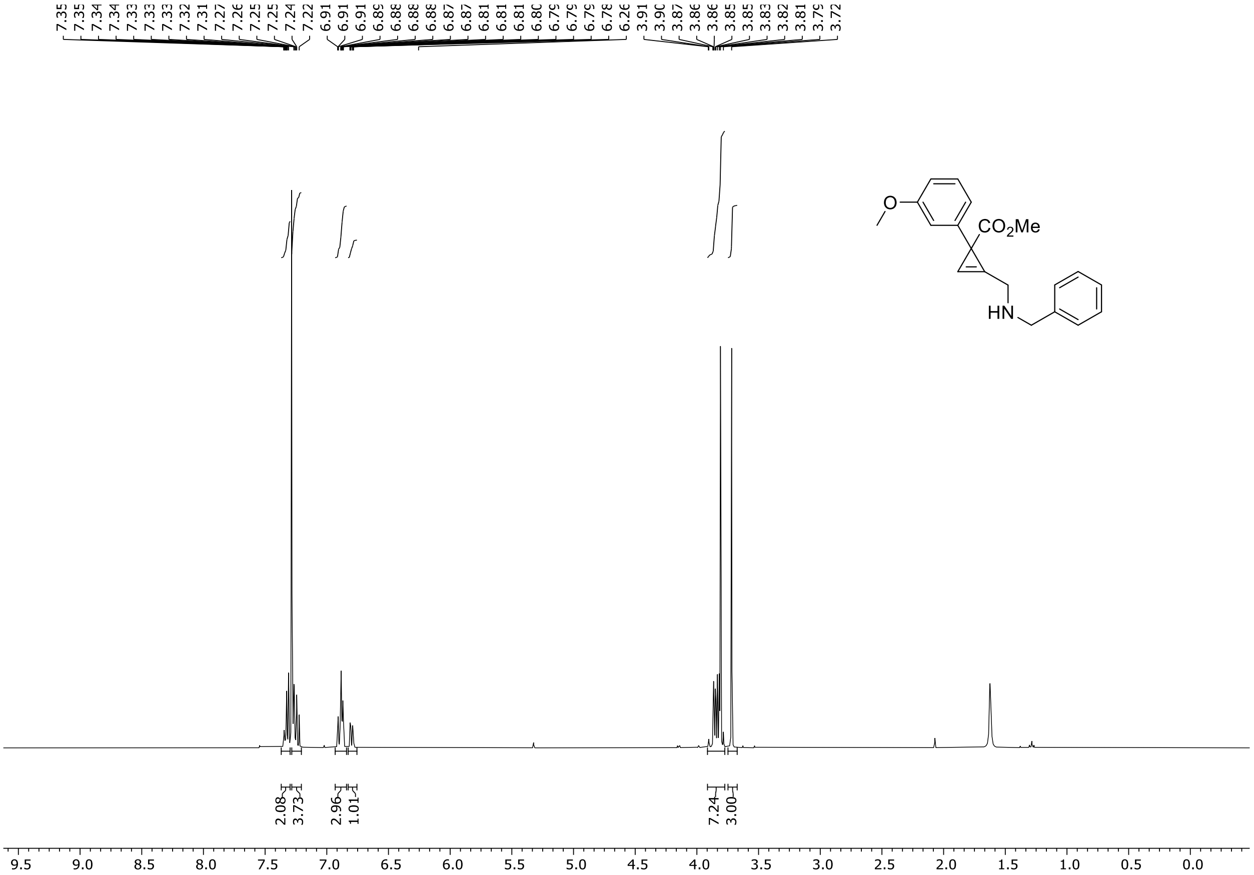

^{13}C NMR Spectrum of 3g (101 MHz, $CDCl_3$)

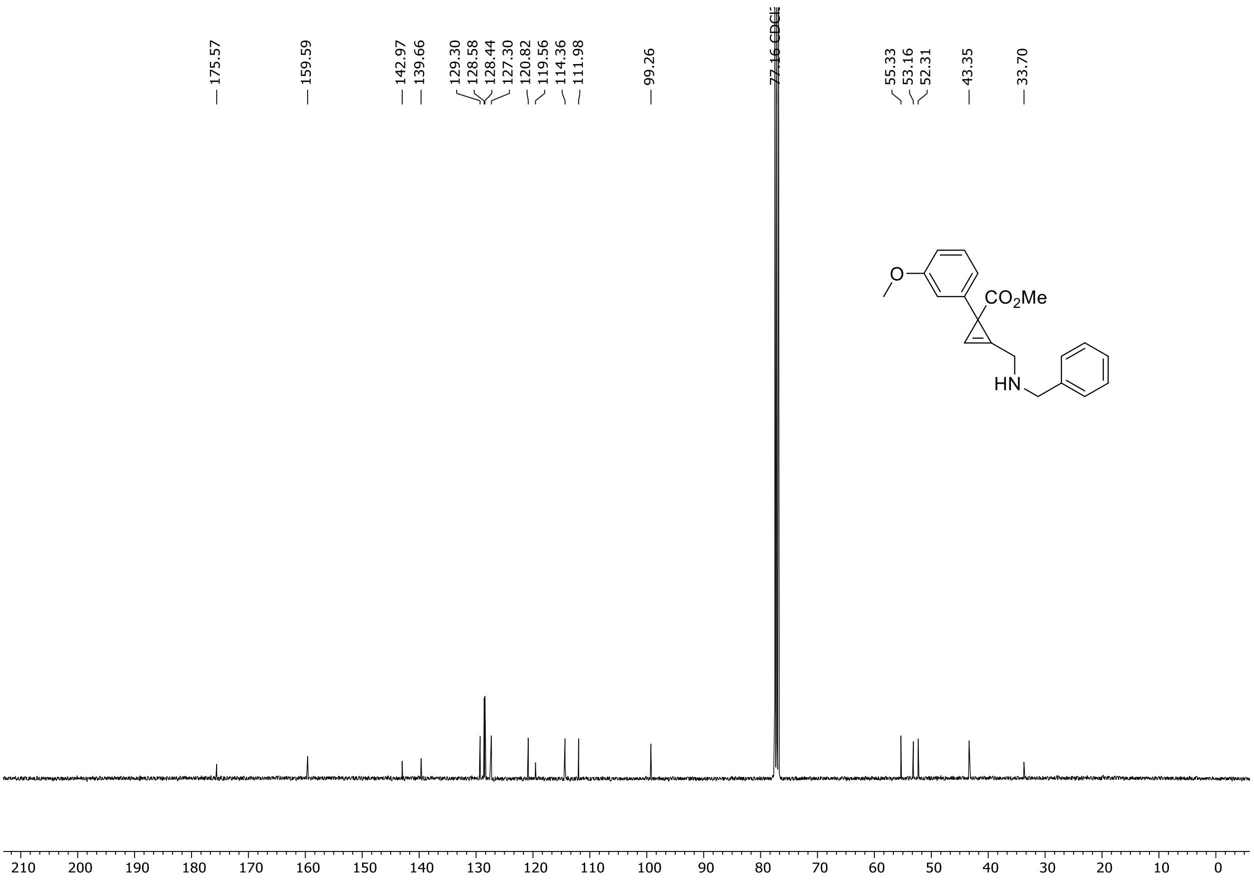

^{1}H NMR Spectrum of 3h (400 MHz, $CDCl_3$)

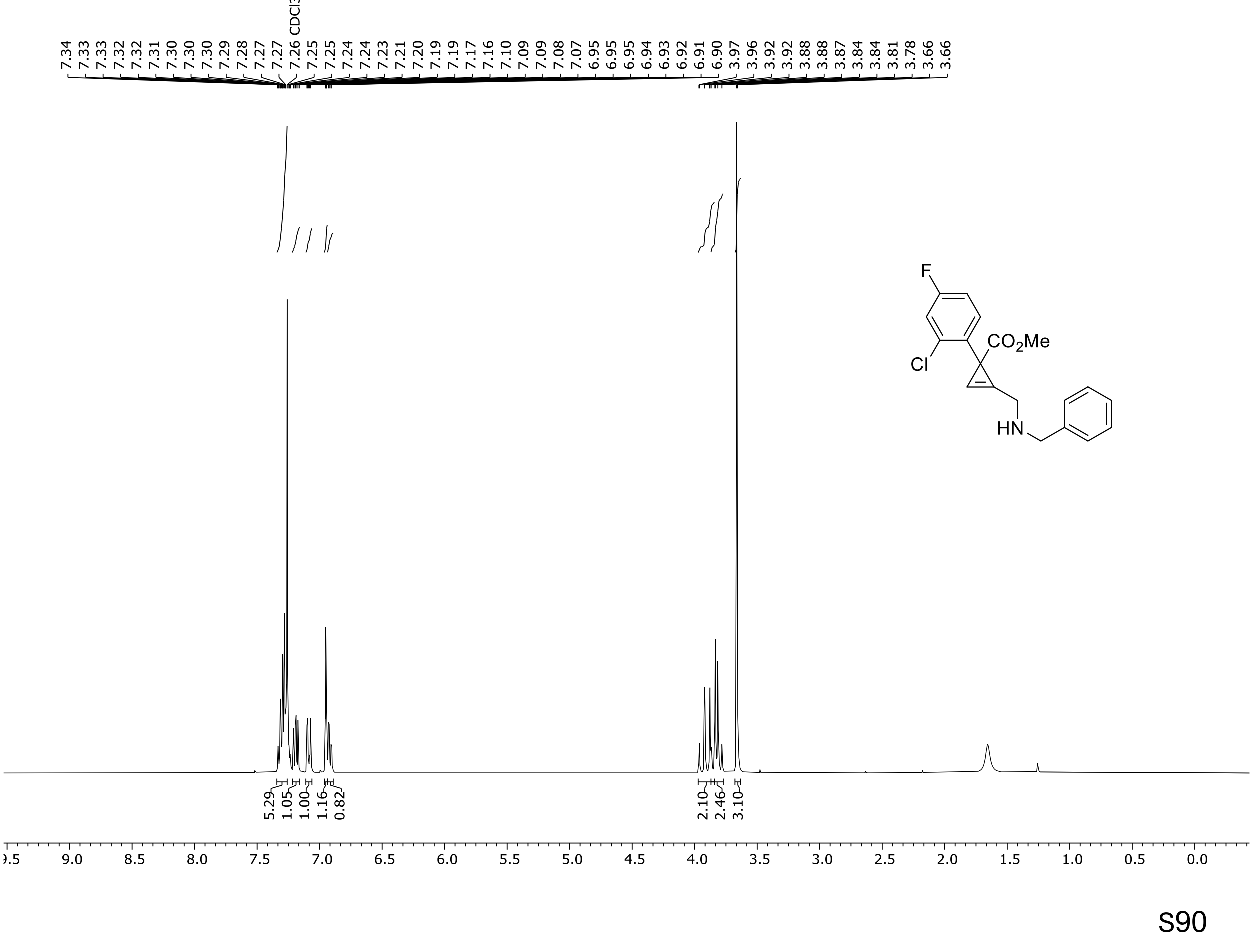

^{13}C NMR Spectrum of 3h (101 MHz, $CDCl_3$)

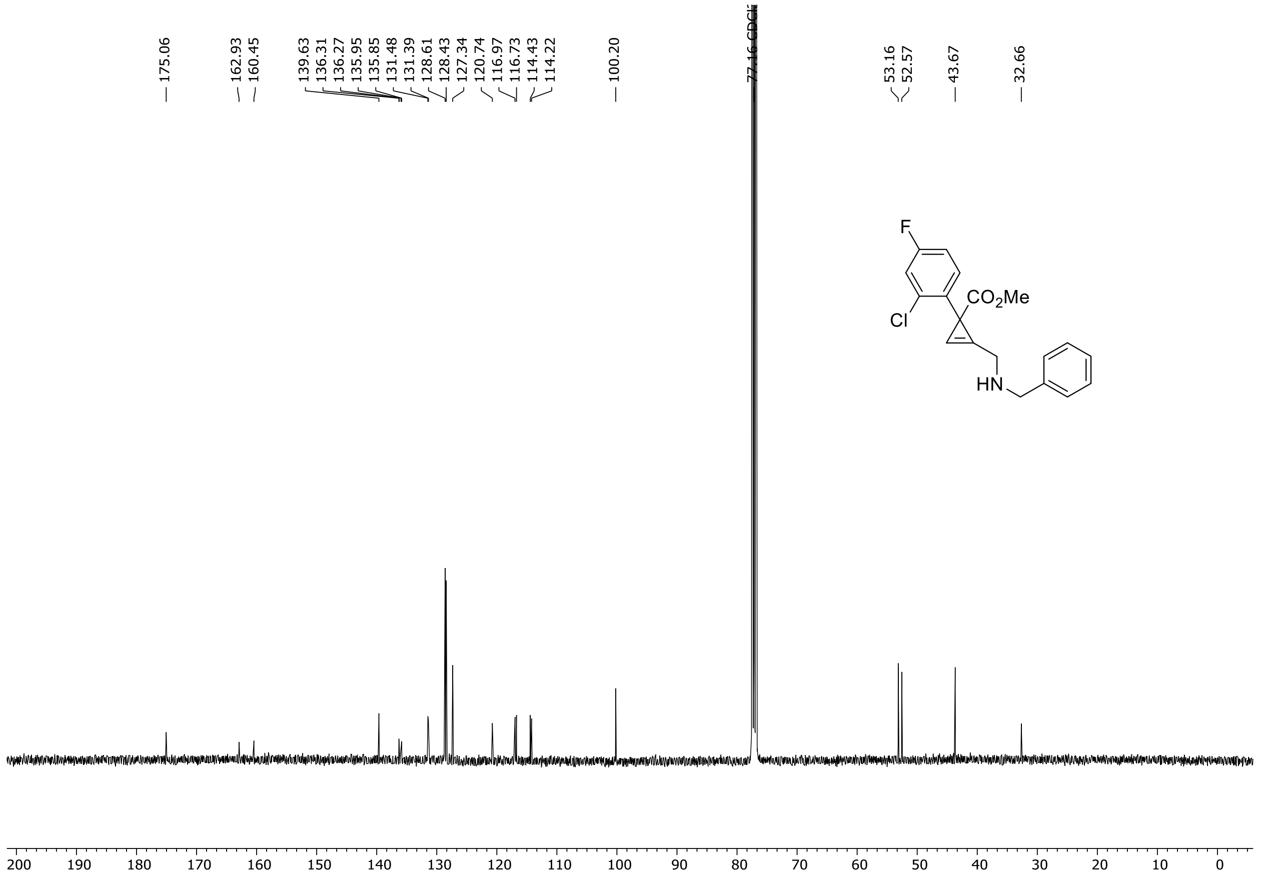

^{19}F NMR Spectrum of 3h (376 MHz, $CDCl_3$)

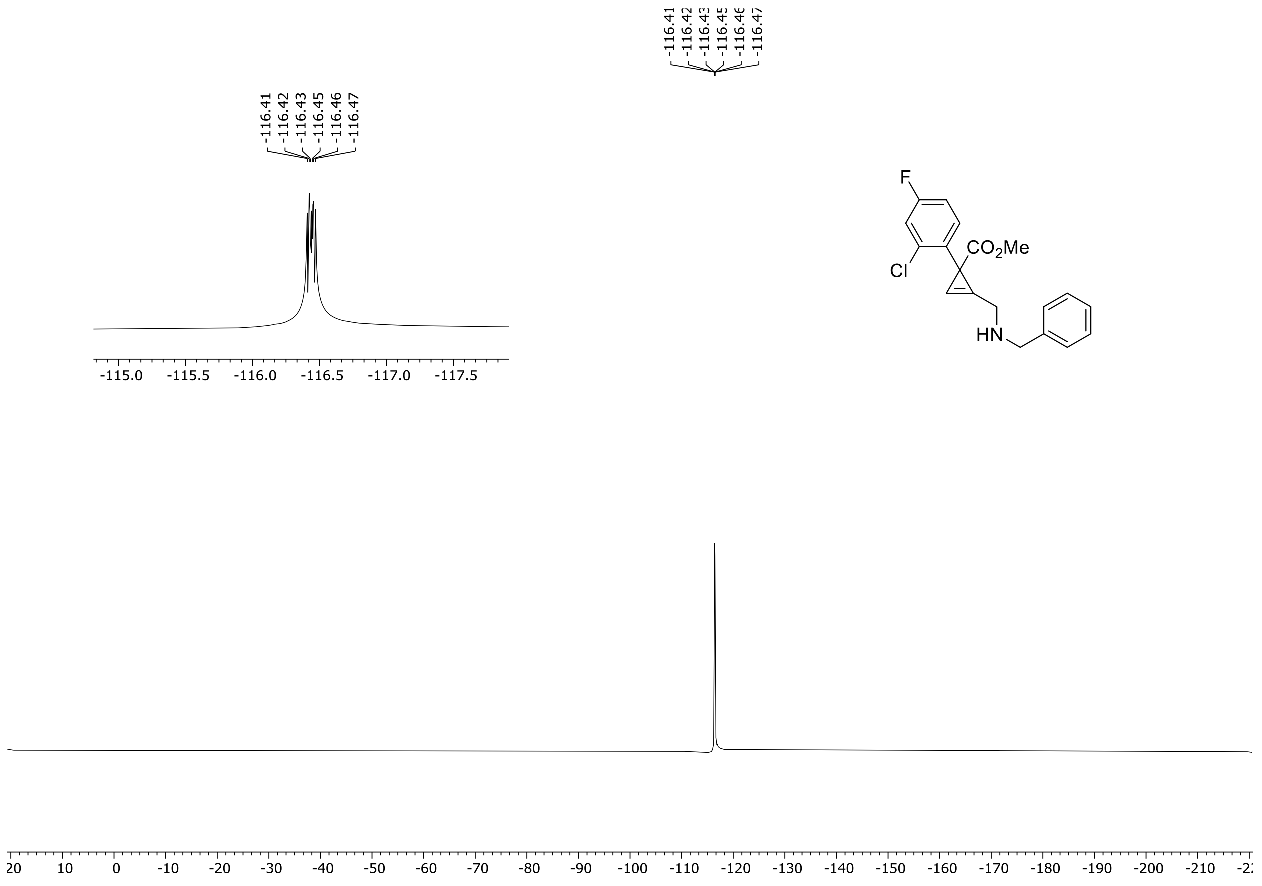

^{1}H NMR Spectrum of 3i (400 MHz, $CDCl_3$)

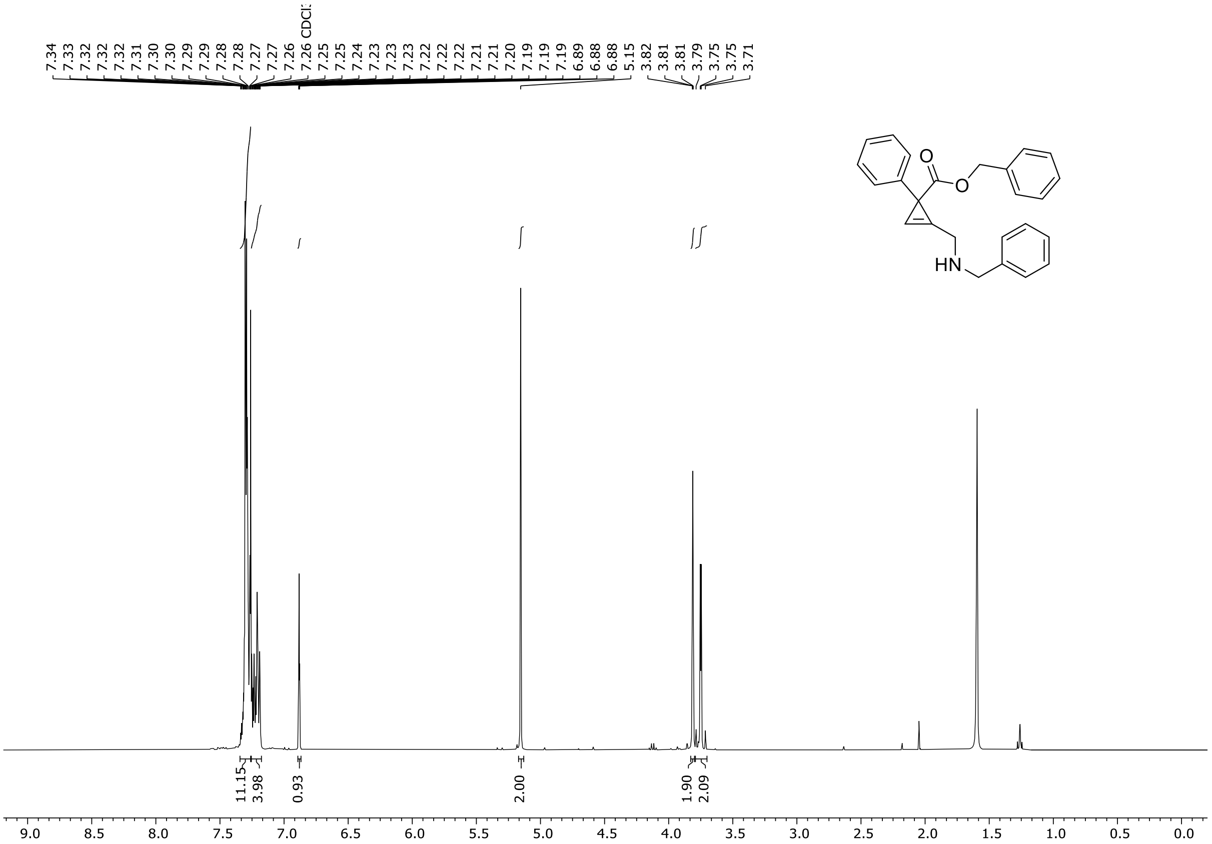

^{13}C NMR Spectrum of 3i (101 MHz, $CDCl_3$)

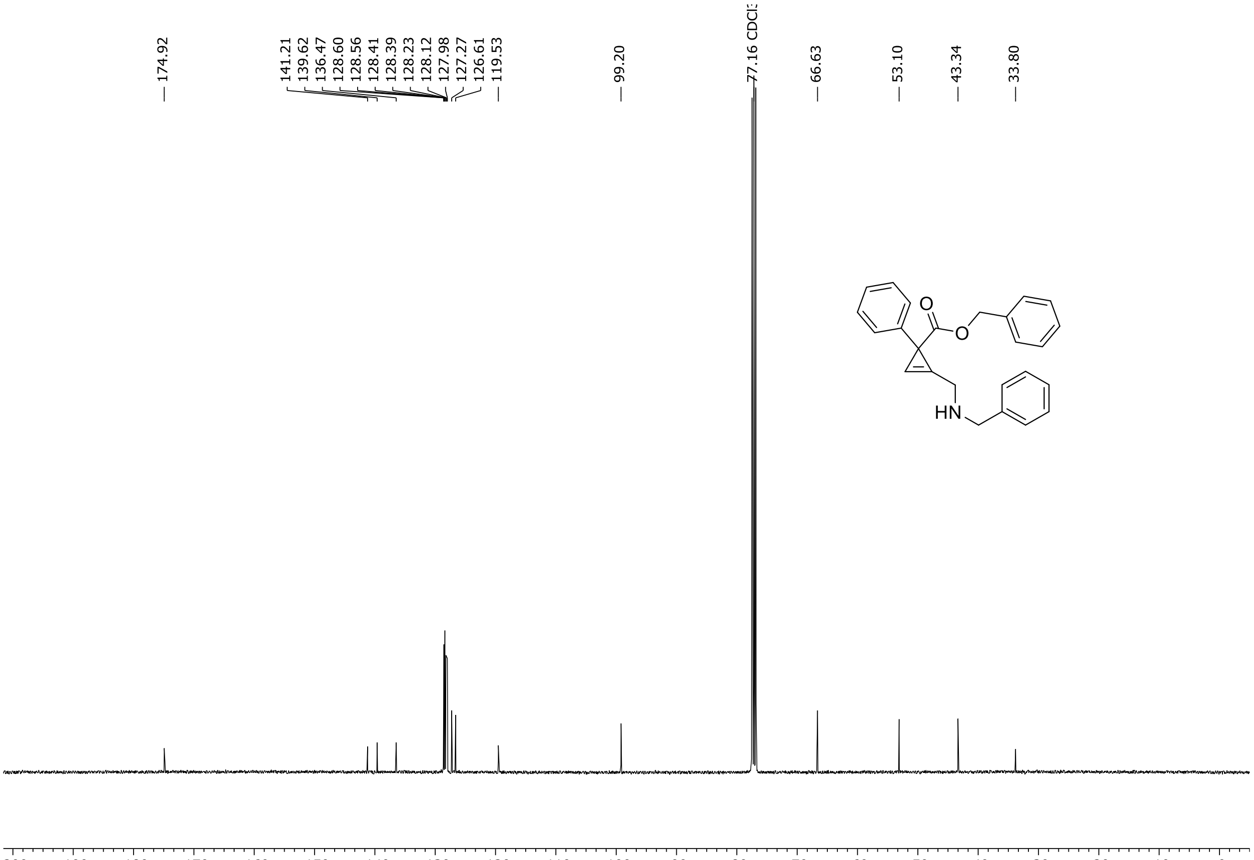

^{1}H NMR Spectrum of 3j (400 MHz, CDCl$_3$)

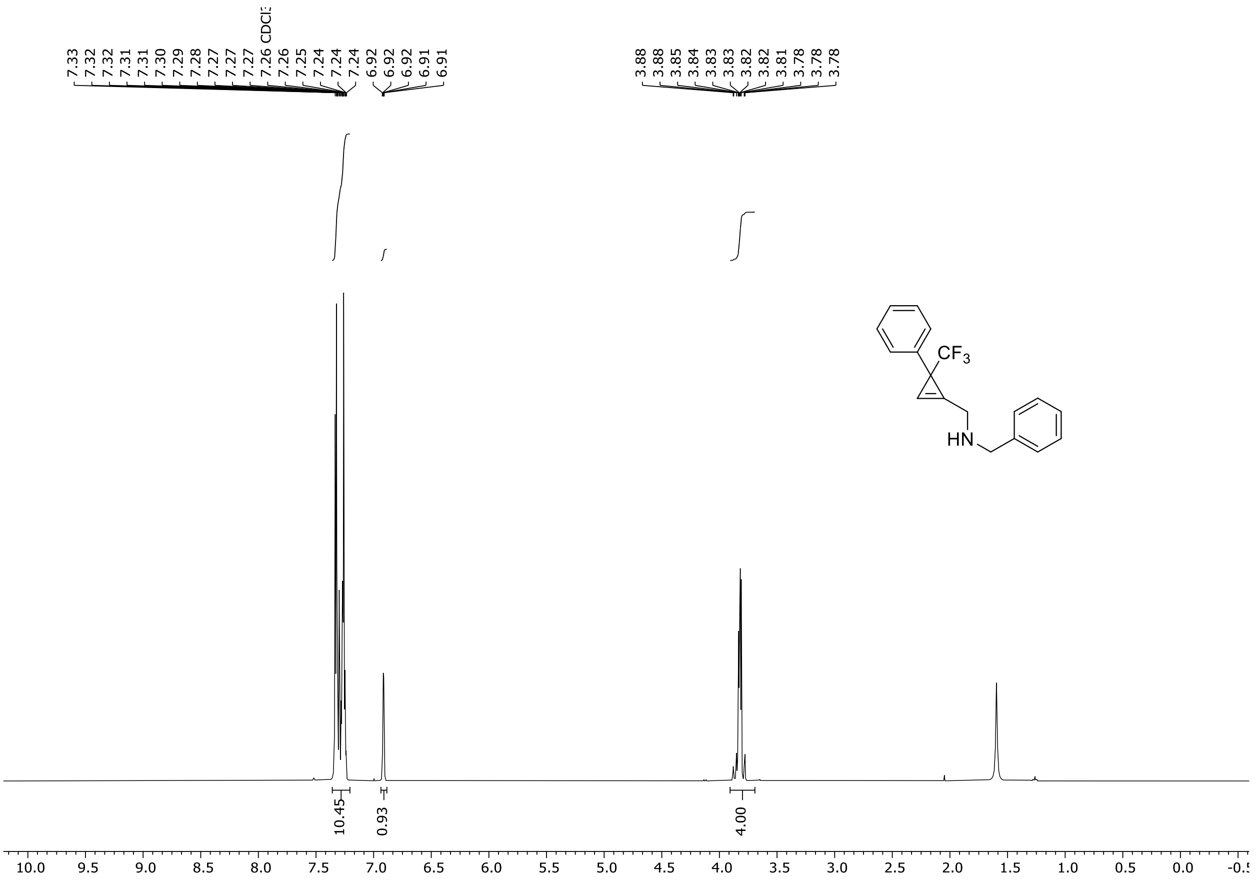

^{13}C NMR Spectrum of 3j (101 MHz, CDCl$_3$)

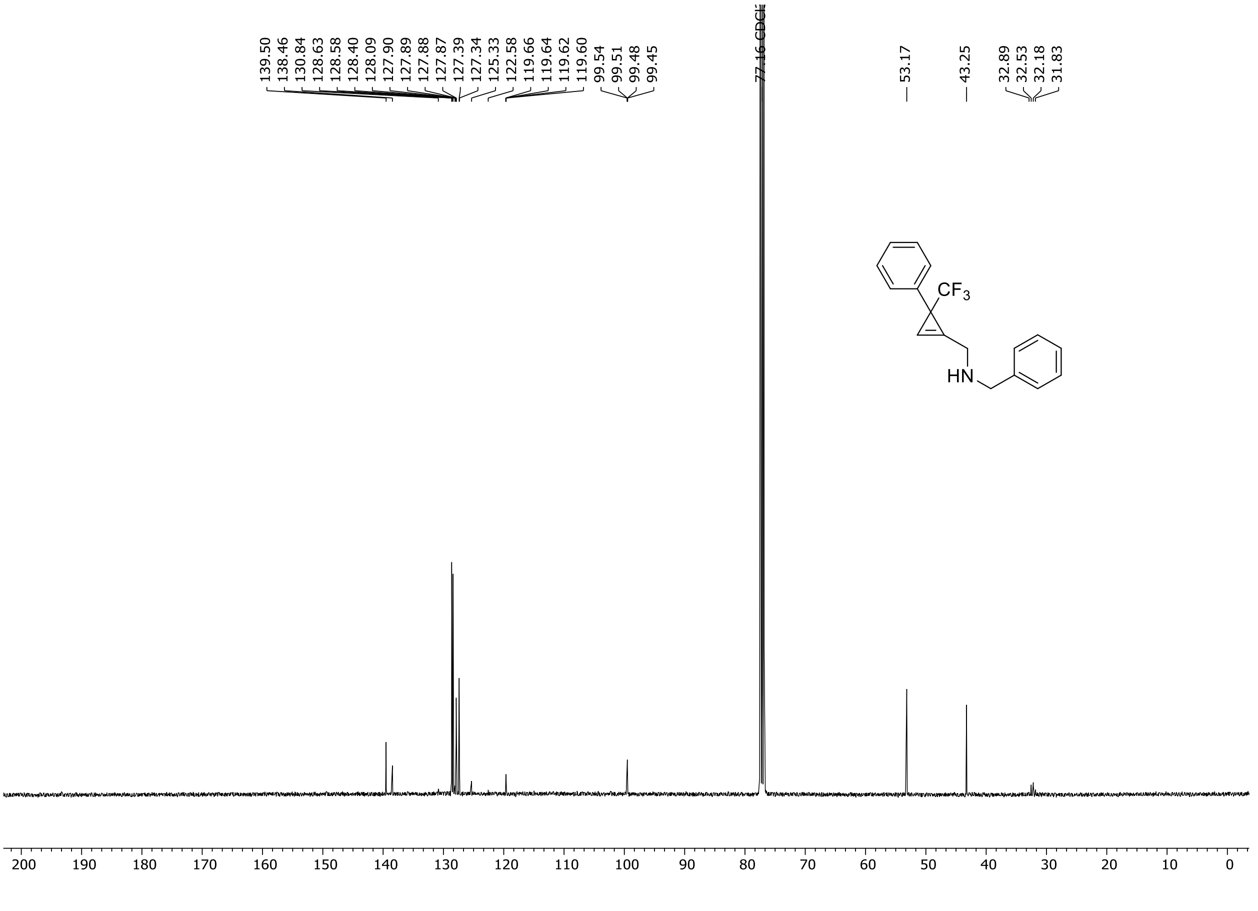

^{19}F NMR Spectrum of 3j (376 MHz, $CDCl_3$)

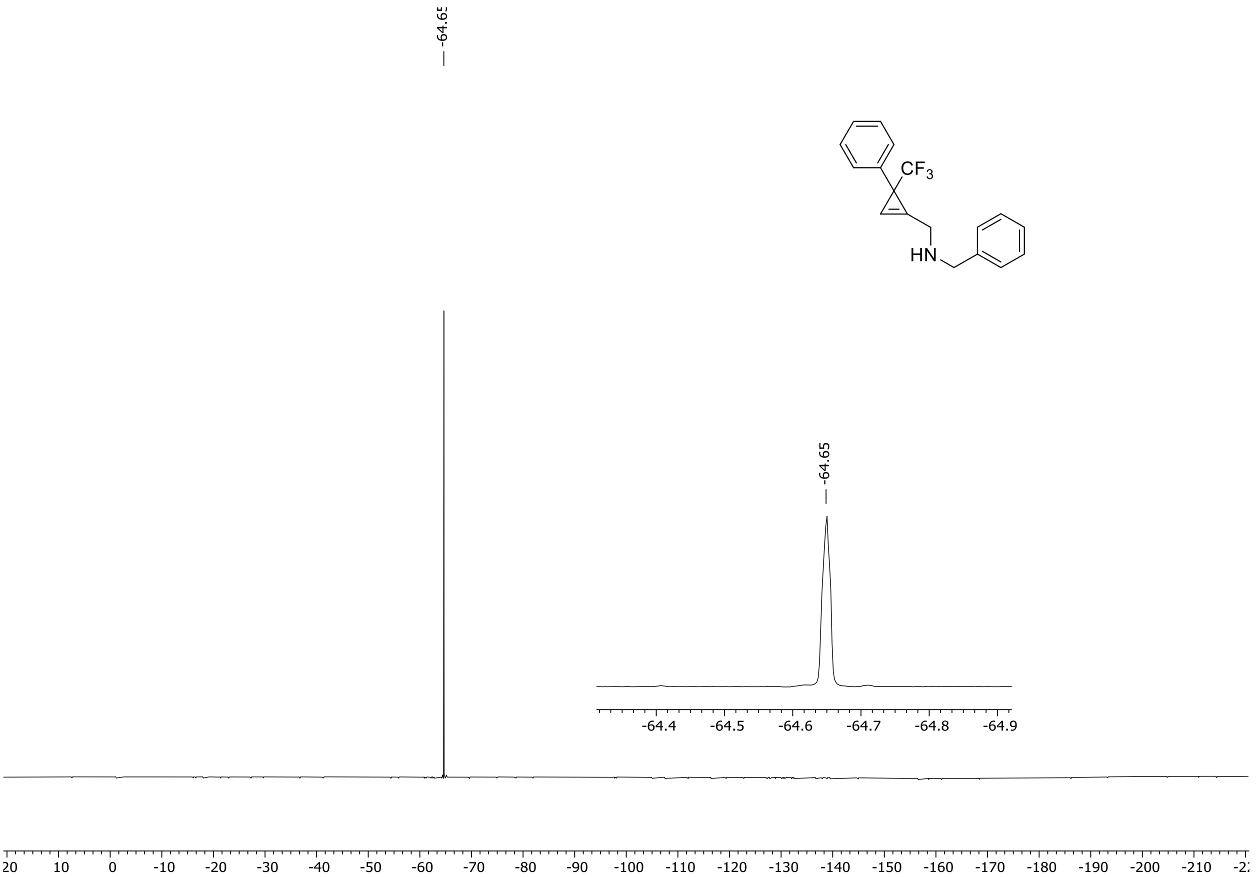

^{1}H NMR Spectrum of 3k (400 MHz, $CDCl_3$)

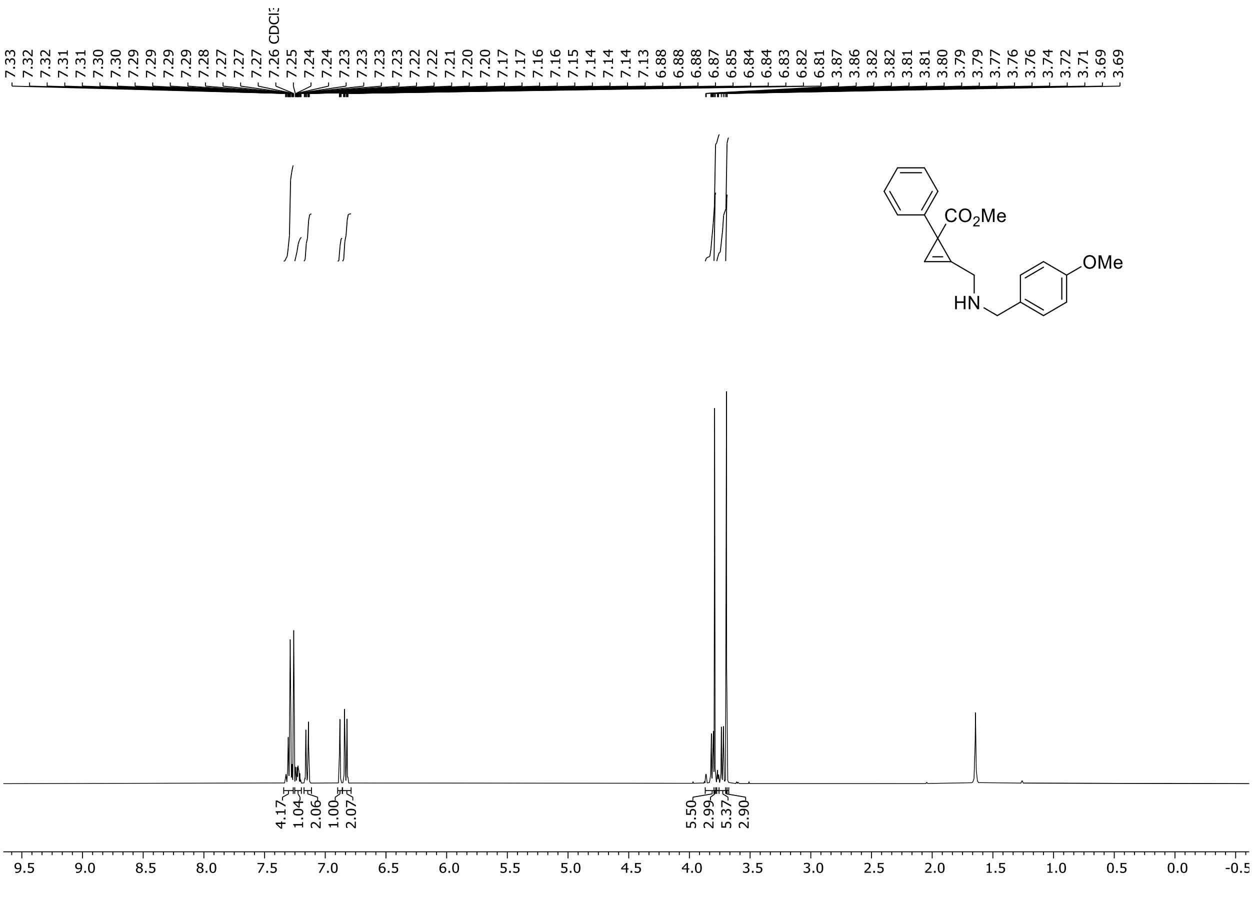

^{13}C NMR Spectrum of 3k (101 MHz, $CDCl_3$)

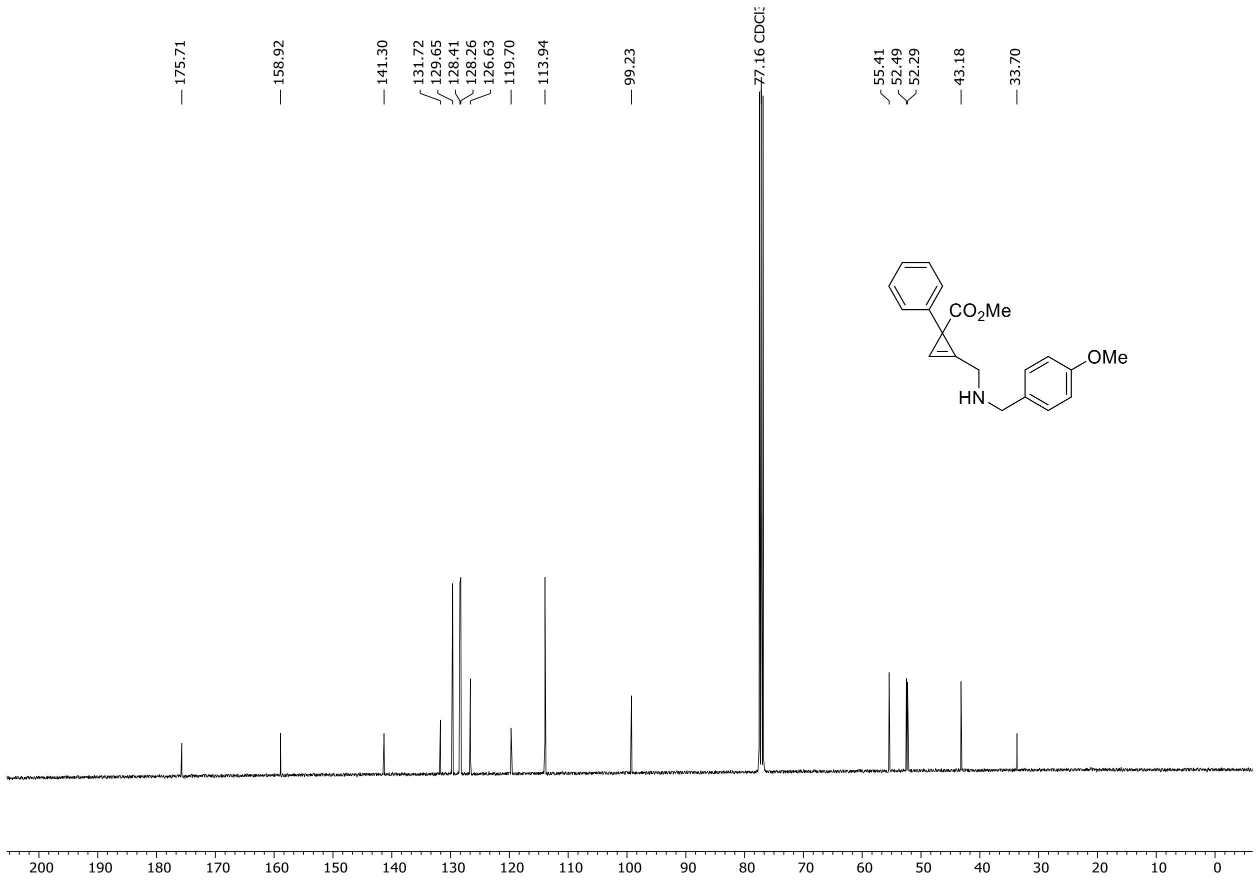

^{1}H NMR Spectrum of 3l (400 MHz, $CDCl_3$)

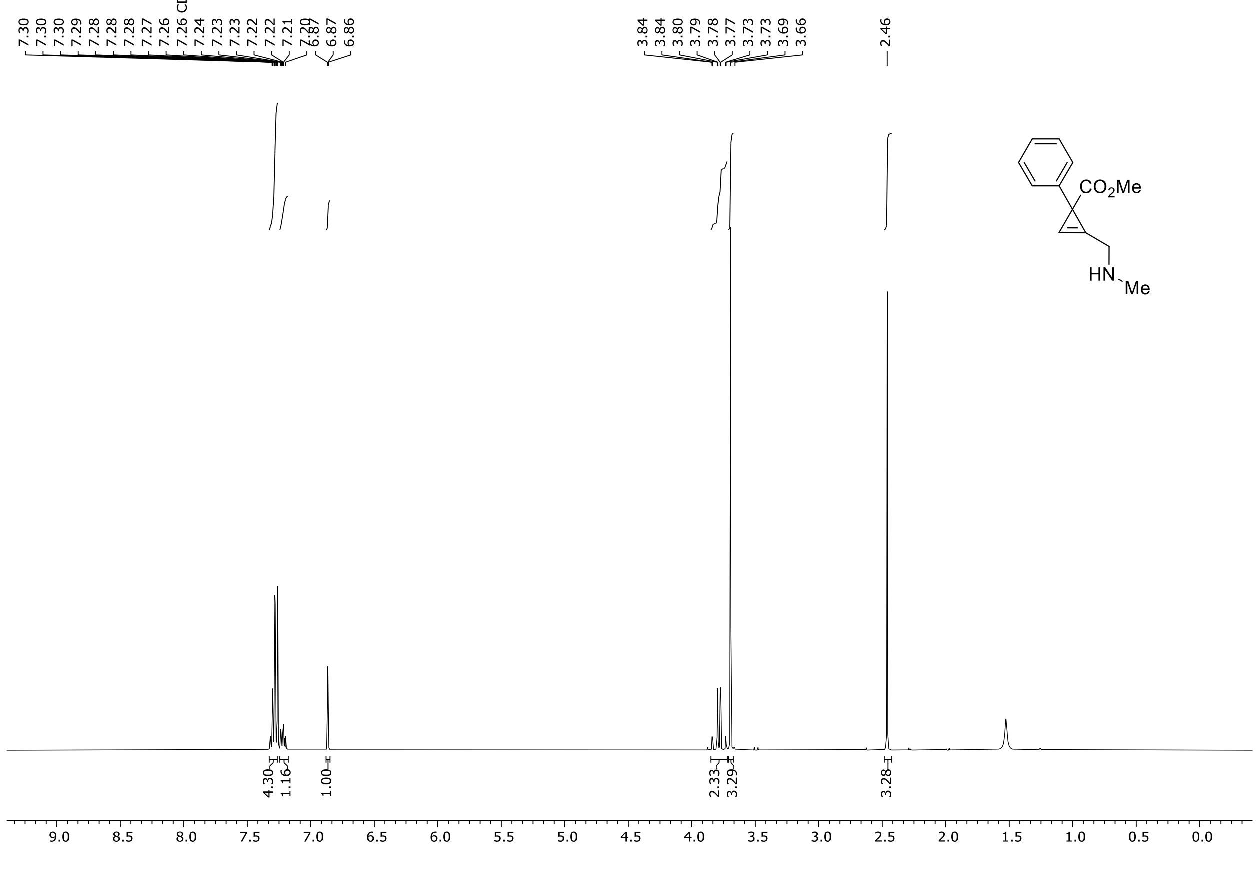

^{13}C NMR Spectrum of 3l (101 MHz, $CDCl_3$)

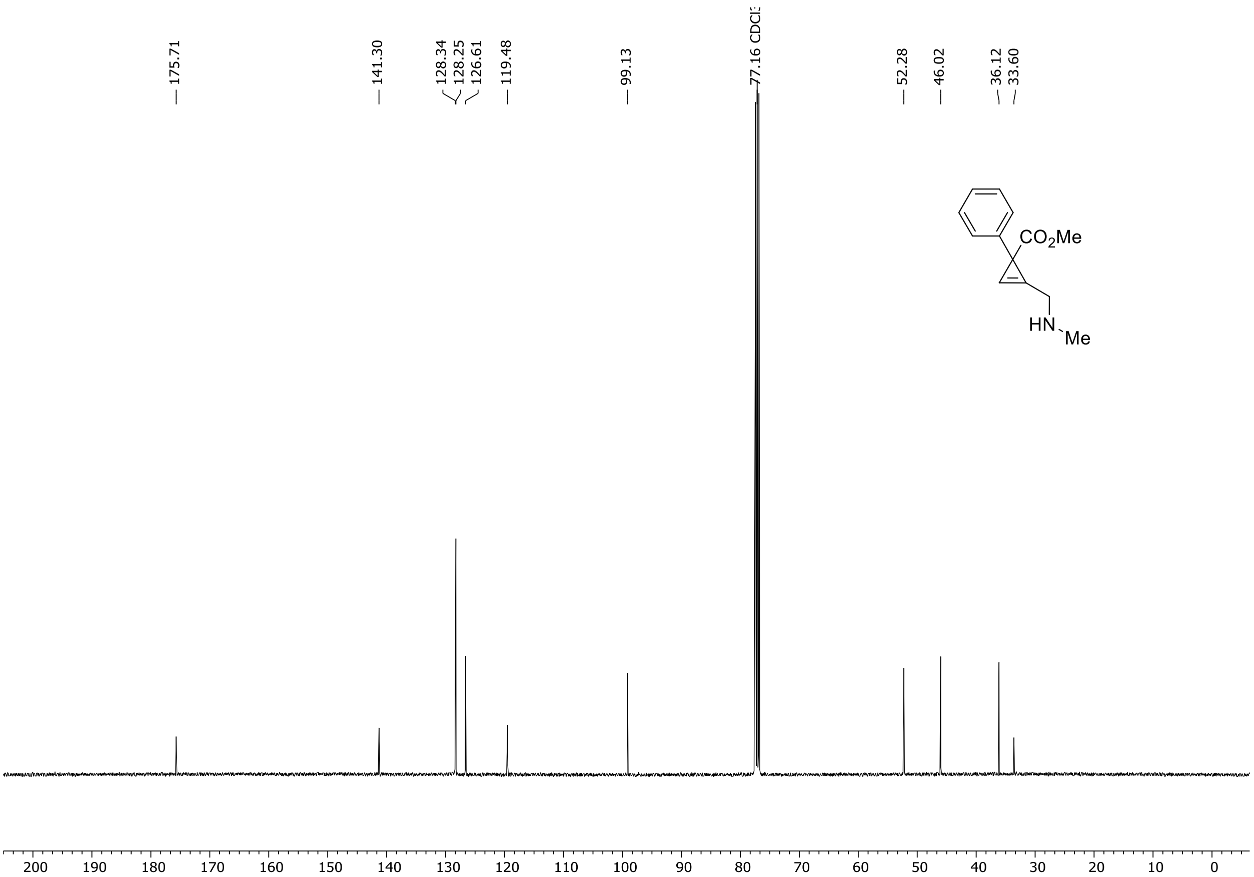

^{1}H NMR Spectrum of 3m (400 MHz, $CDCl_3$)

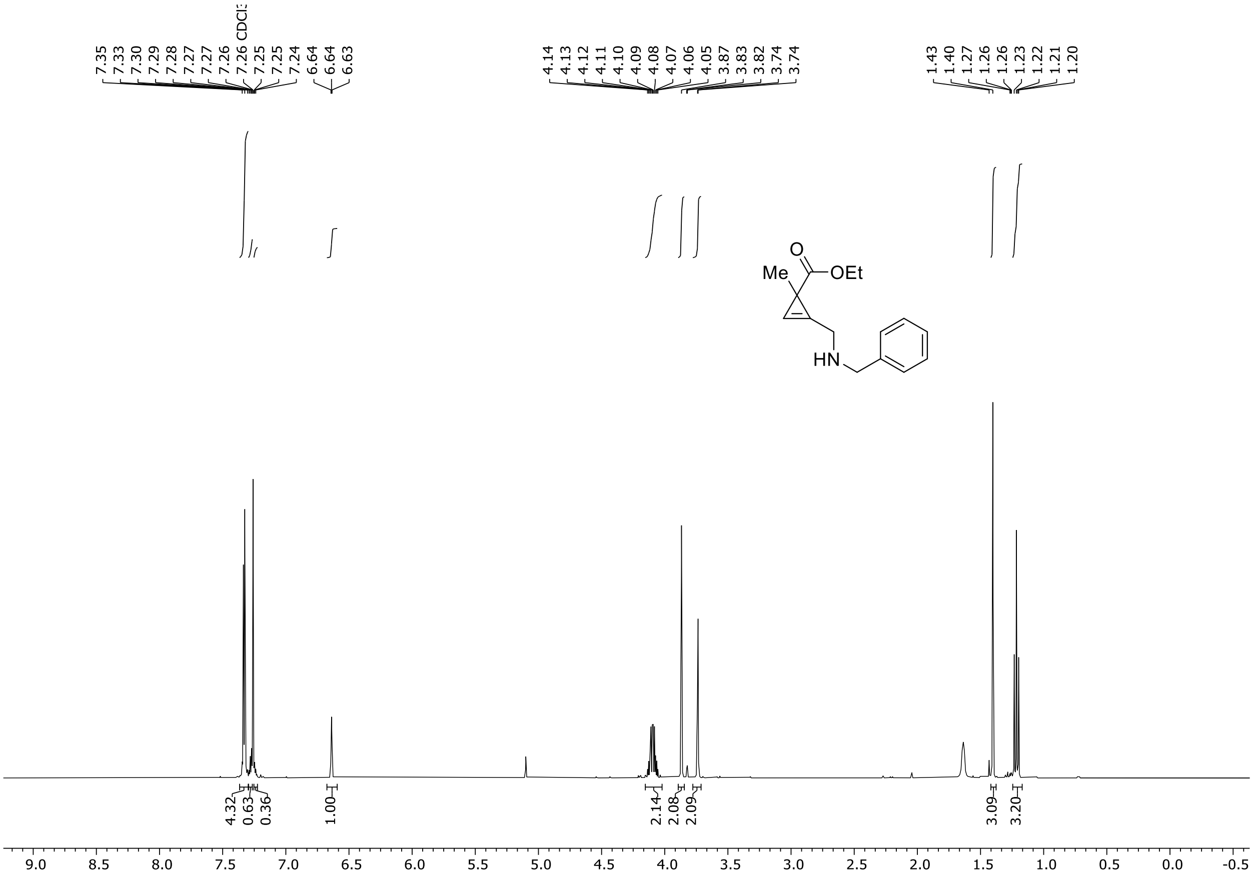

^{13}C NMR Spectrum of 3m (101 MHz, CDCl$_3$)

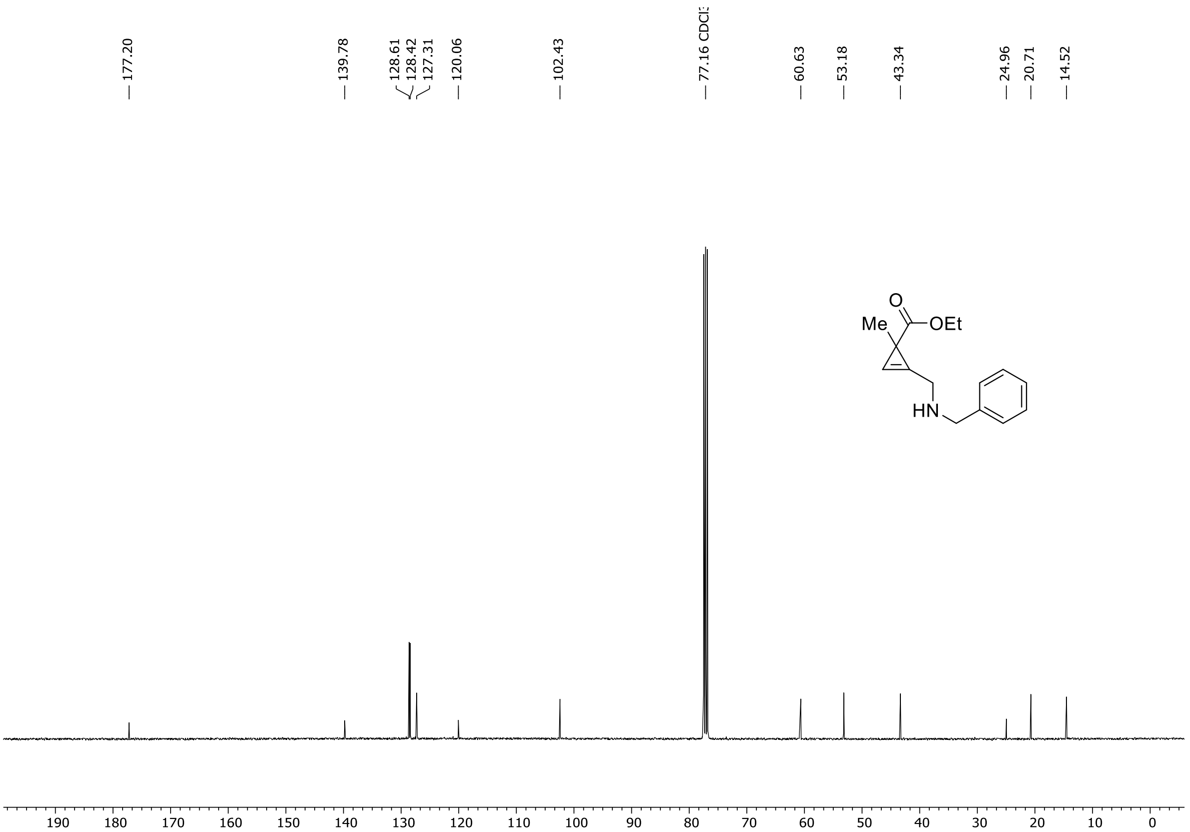

^{1}H NMR Spectrum of 3n (400 MHz, CDCl$_3$)

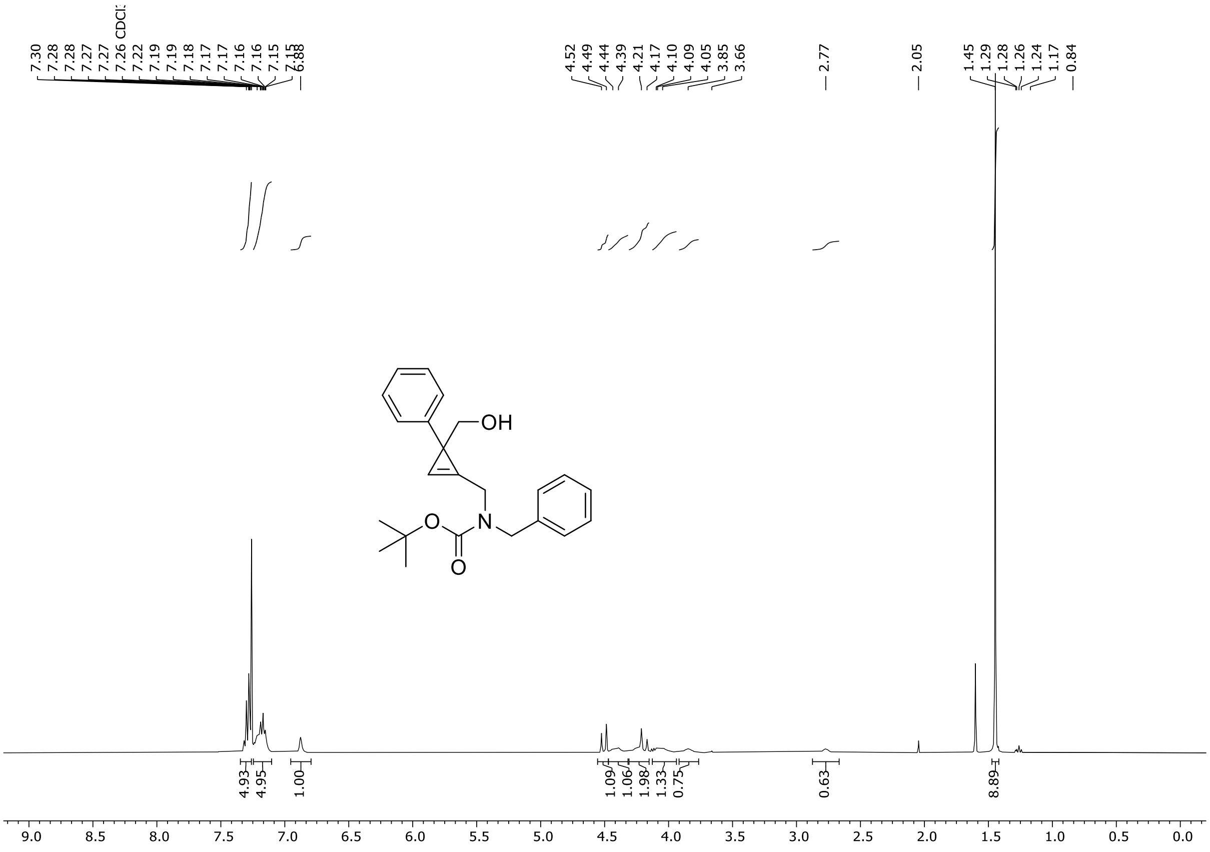

^{13}C NMR Spectrum of 3n (101 MHz, $CDCl_3$)

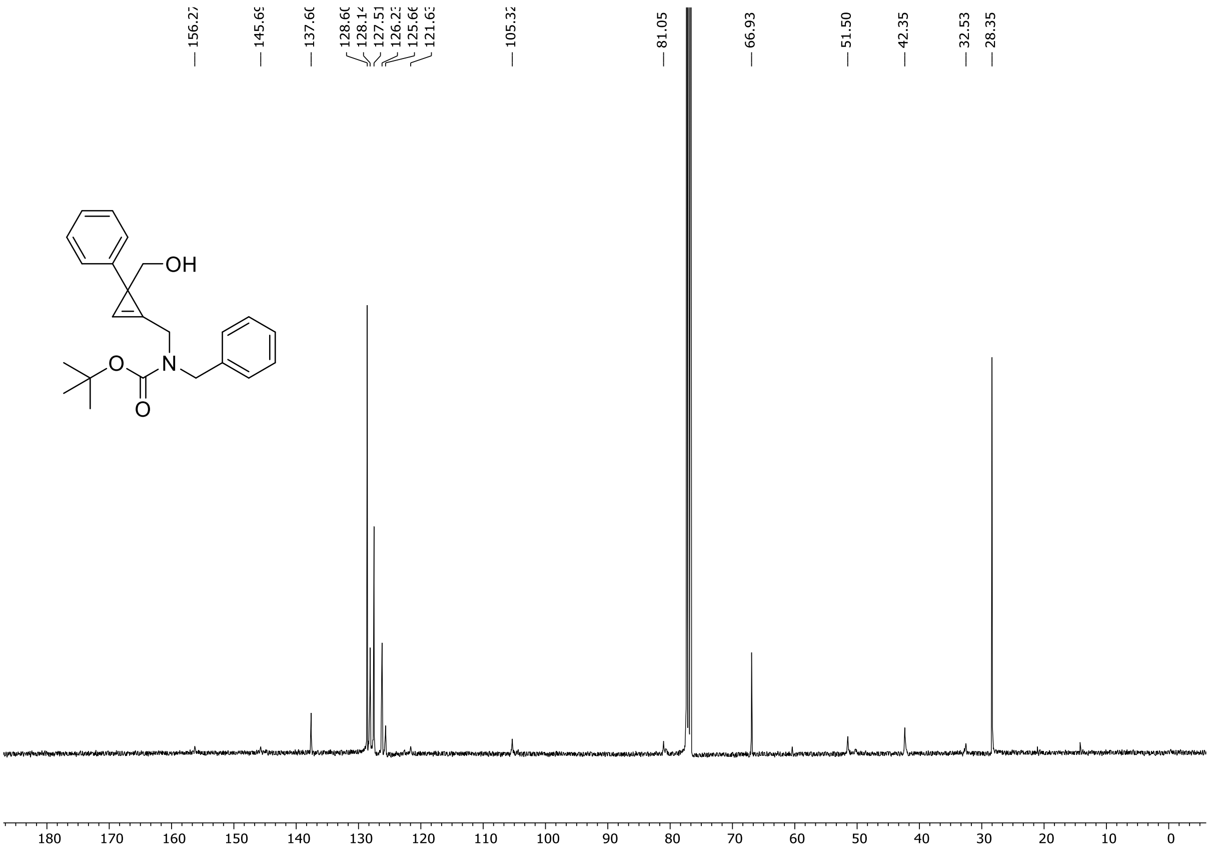

^{1}H NMR Spectrum of 3o (400 MHz, $CDCl_3$)

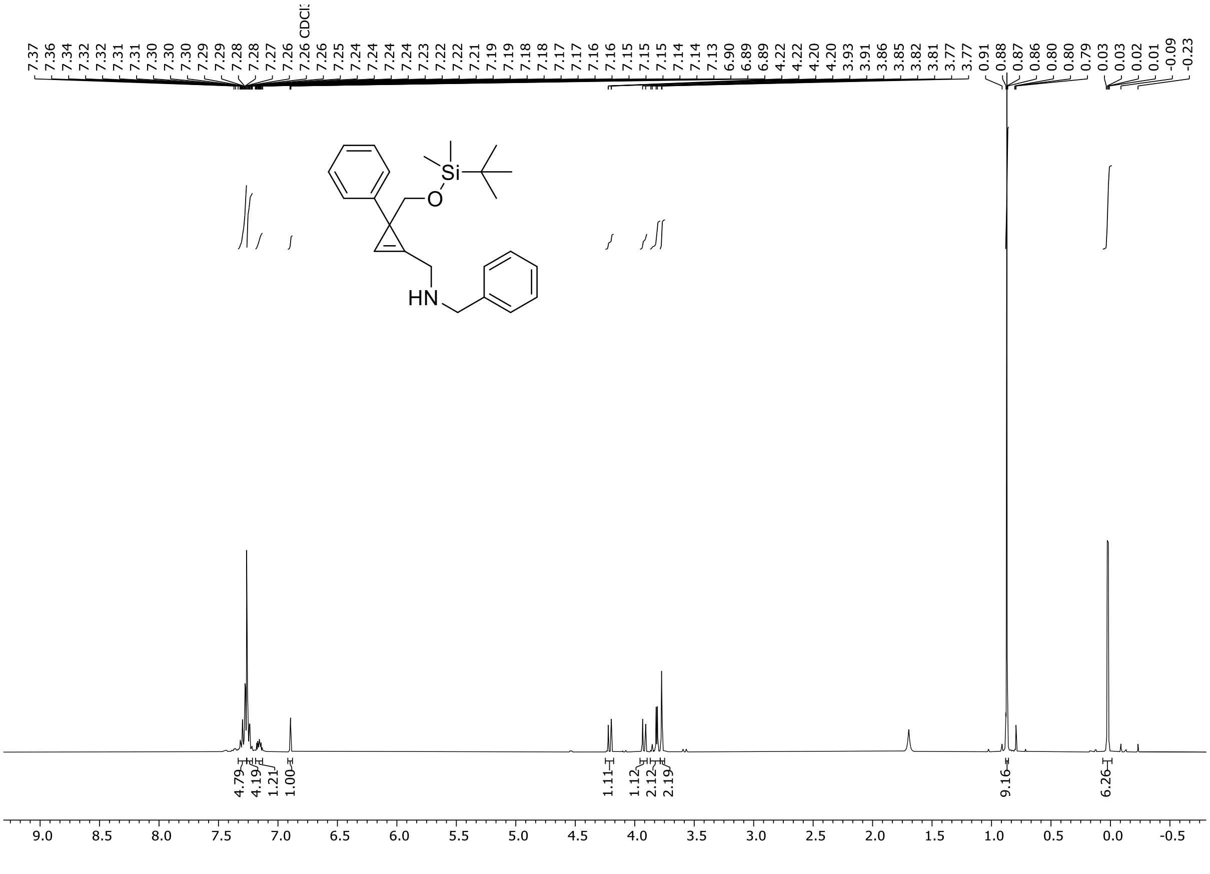

^{13}C NMR Spectrum of 3n (101 MHz, $CDCl_3$)

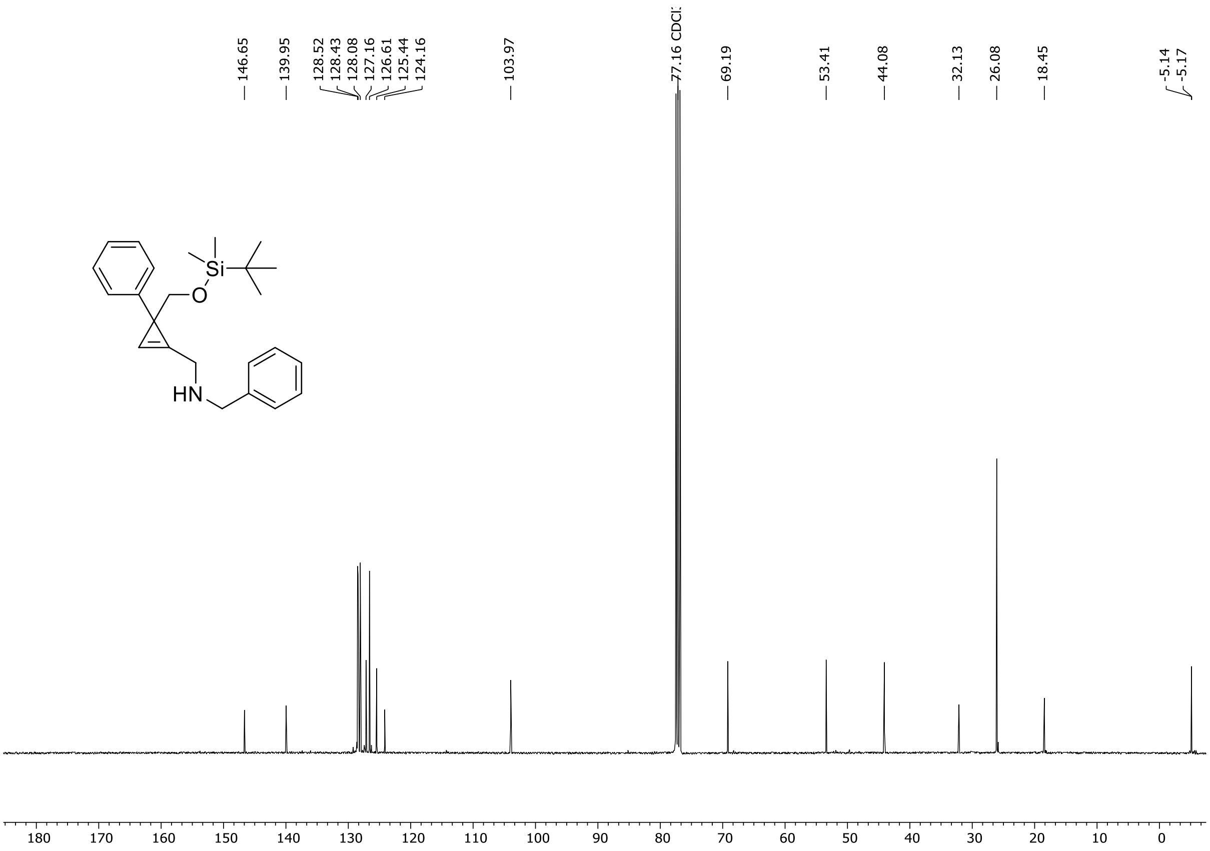

Cyclopropane products

^{1}H NMR Spectrum of 6a (400 MHz, $CDCl_3$)

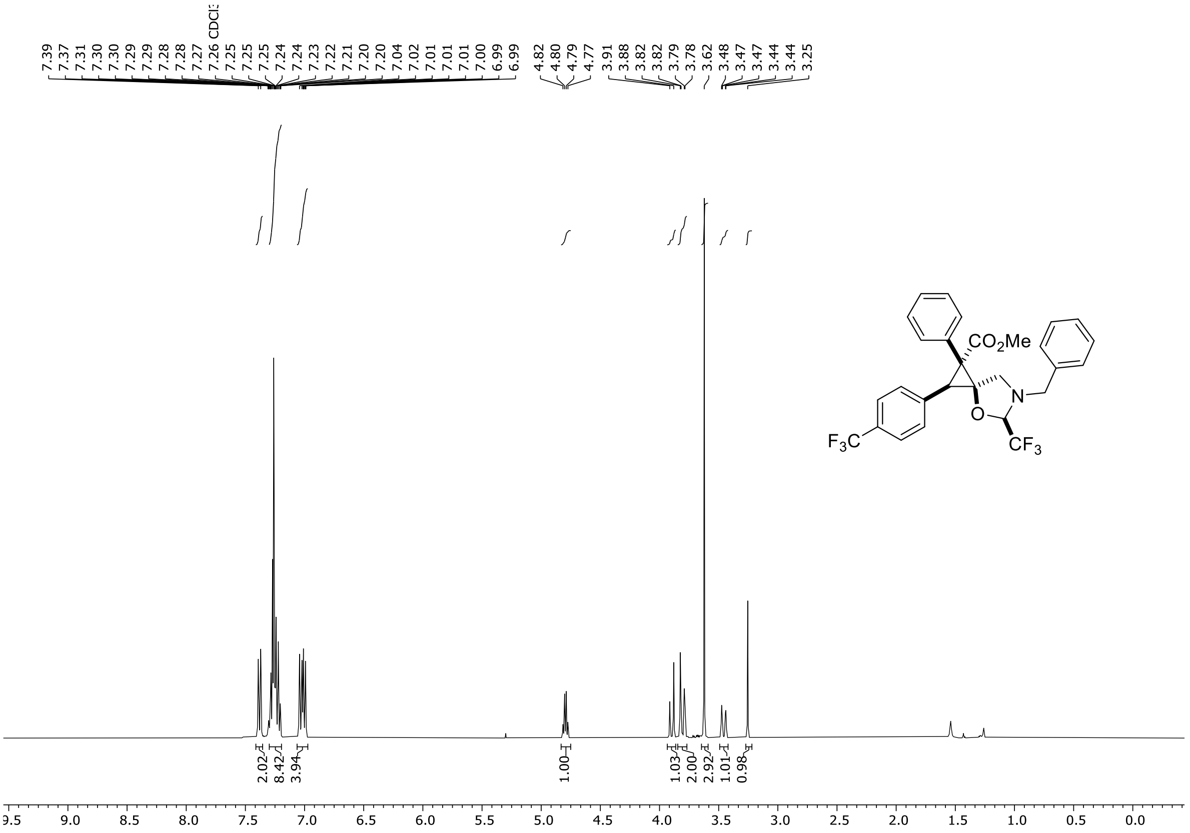

^{13}C NMR Spectrum of 6a (101 MHz, $CDCl_3$)

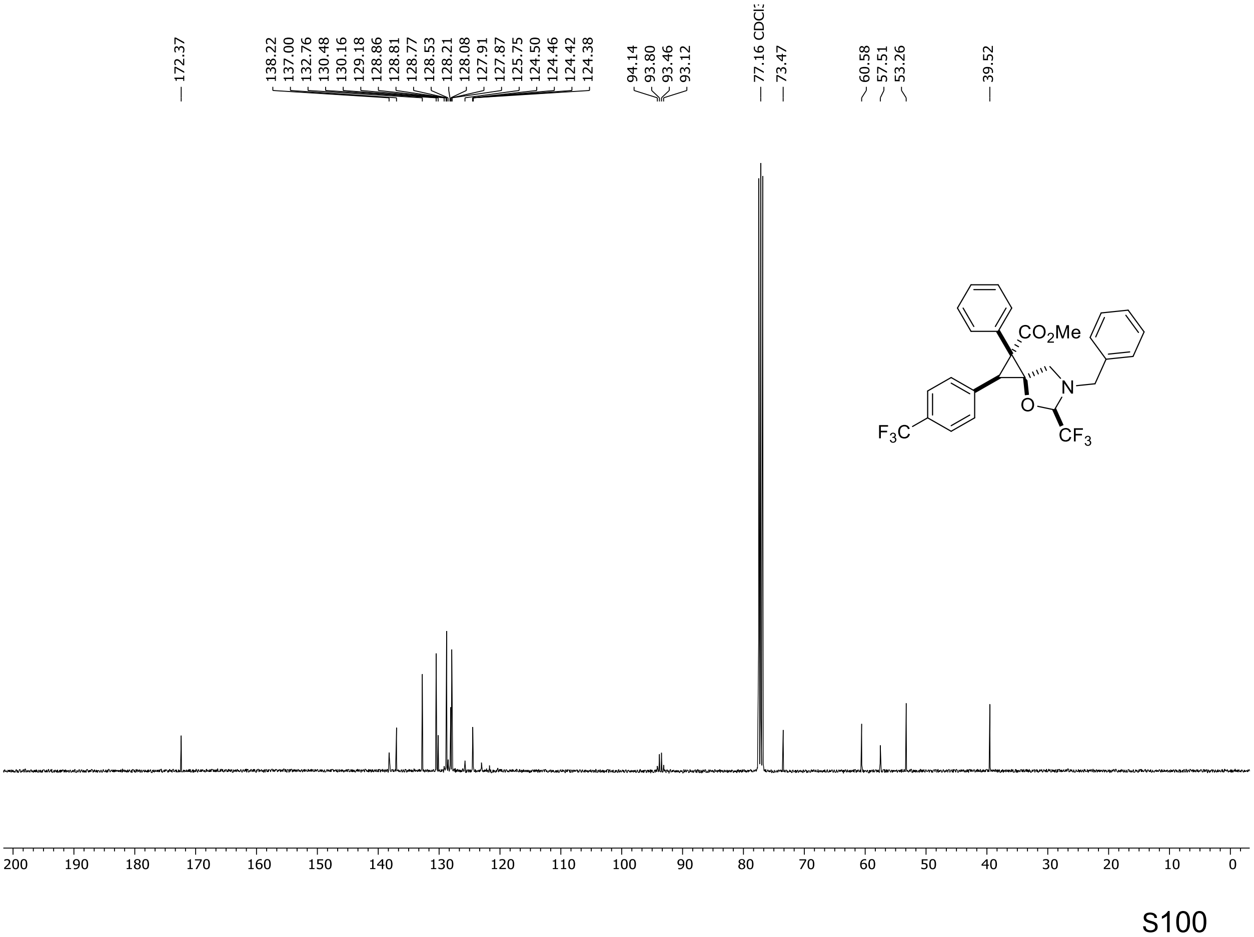

^{19}F NMR Spectrum of 6a (376 MHz, $CDCl_3$)

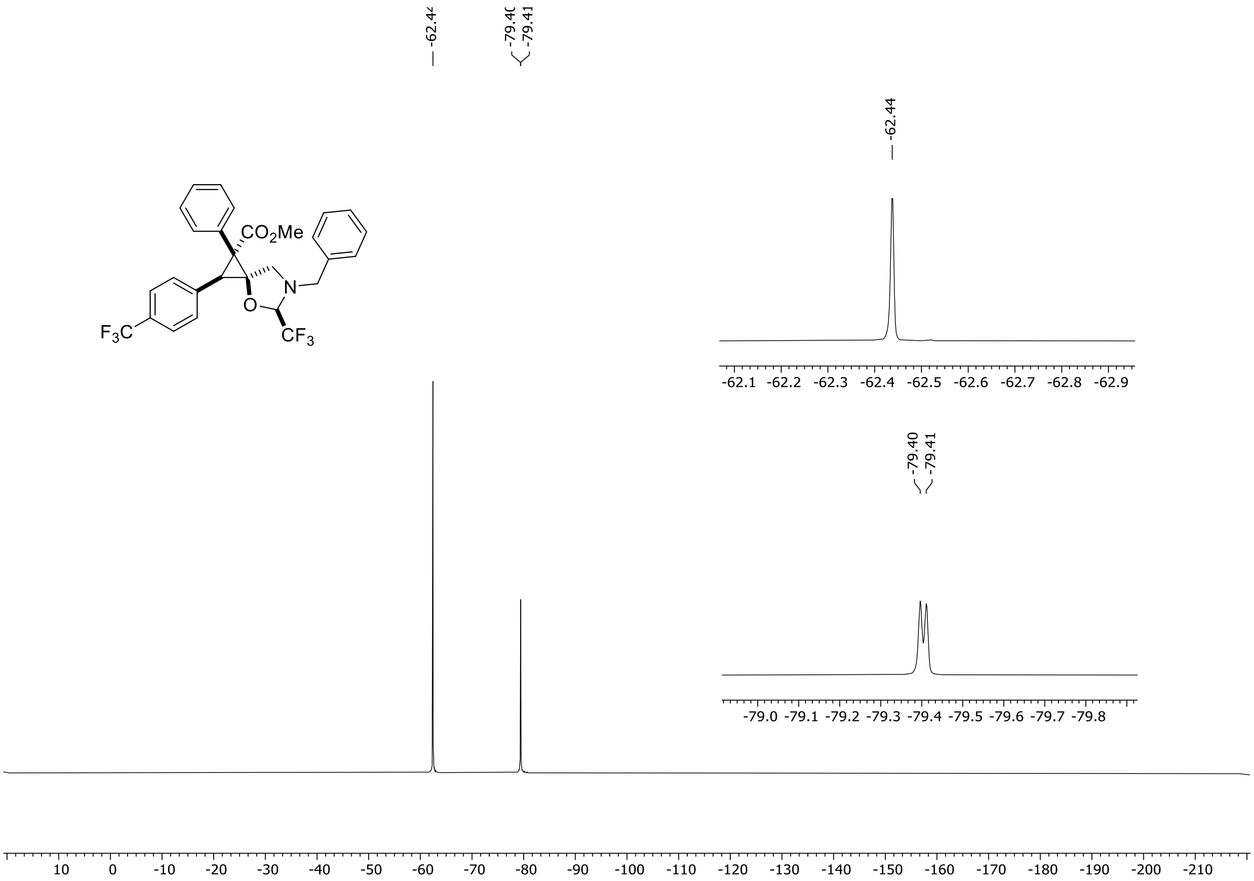

^{1}H NMR Spectrum of 6b (400 MHz, $CDCl_3$)

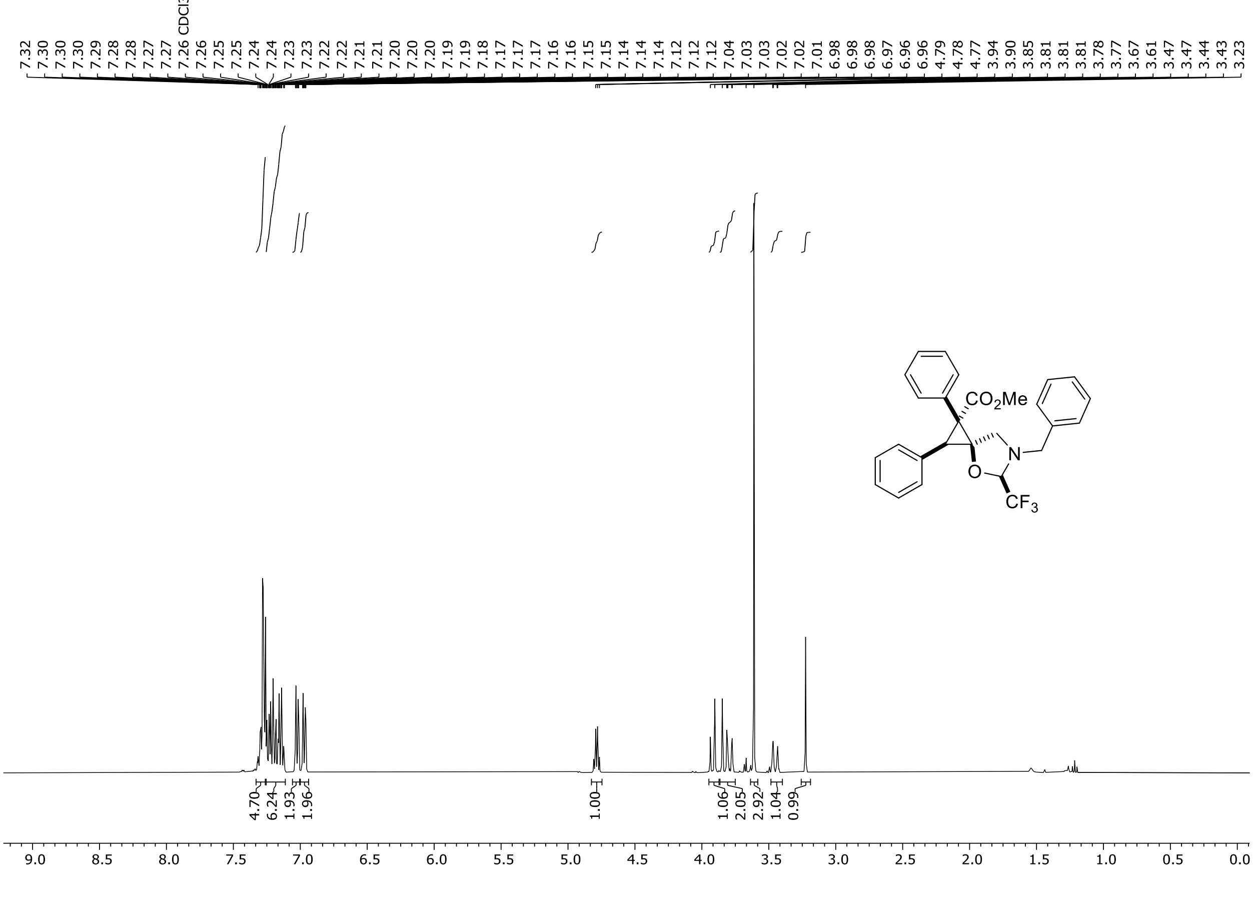

^{13}C NMR Spectrum of 6b (101 MHz, $CDCl_3$)

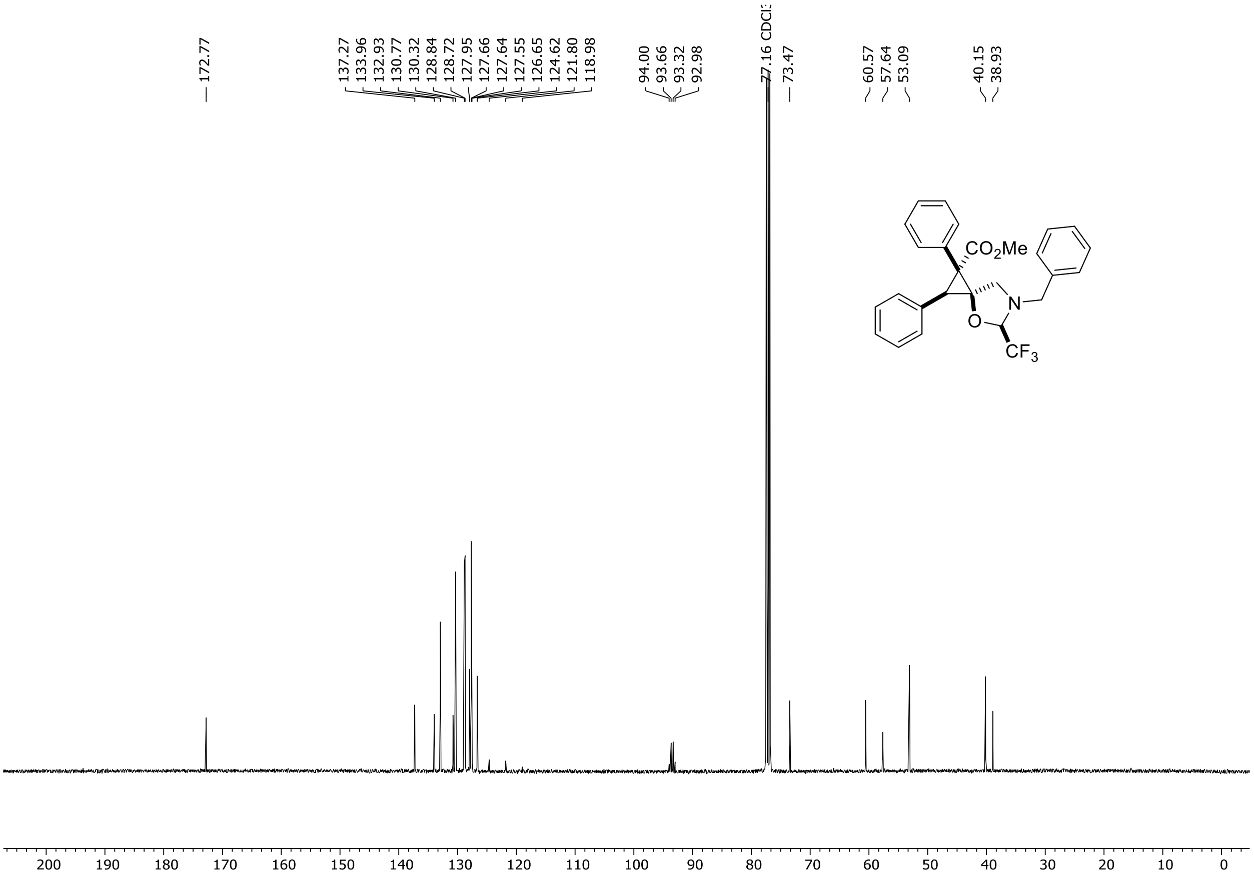

^{19}F NMR Spectrum of 6b (376 MHz, $CDCl_3$)

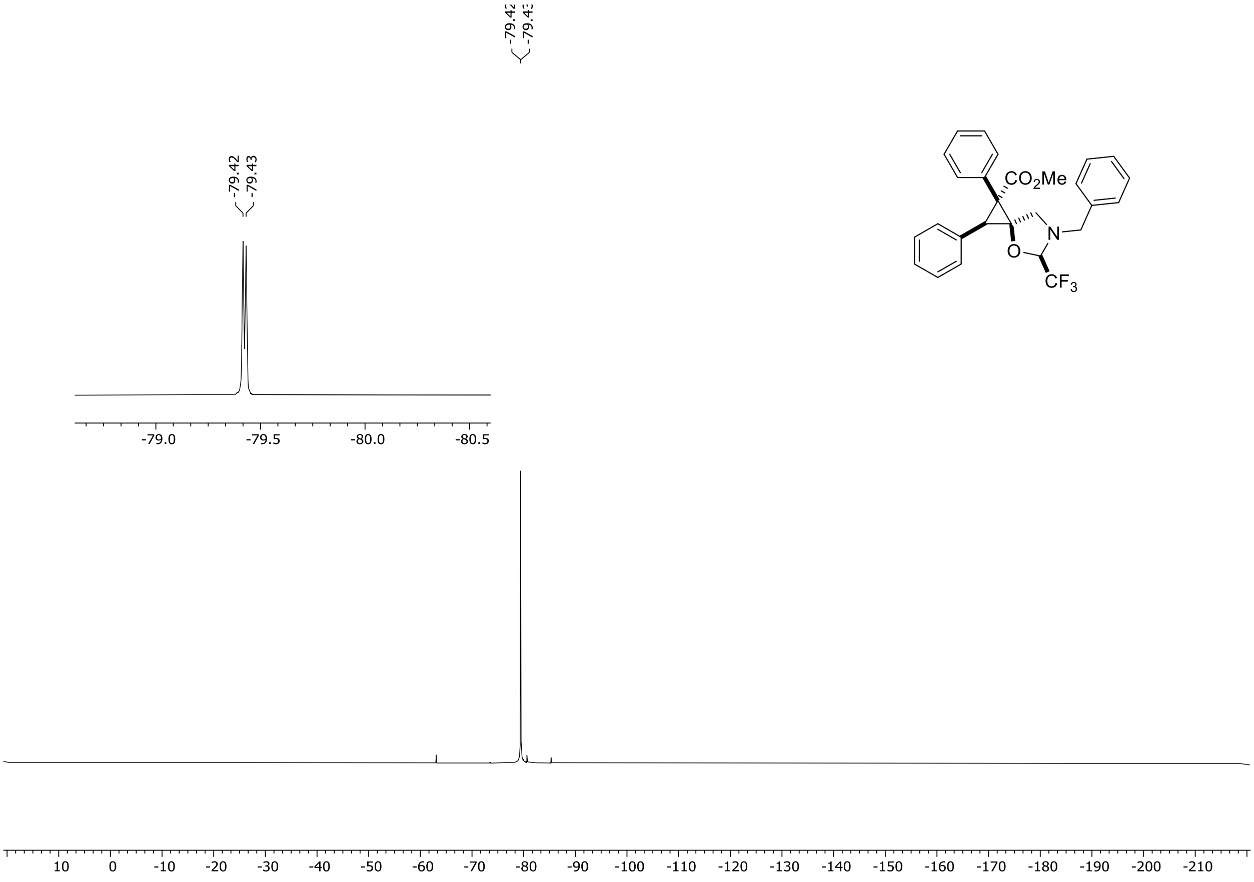

^{1}H NMR Spectrum of 6c (400 MHz, $CDCl_3$)

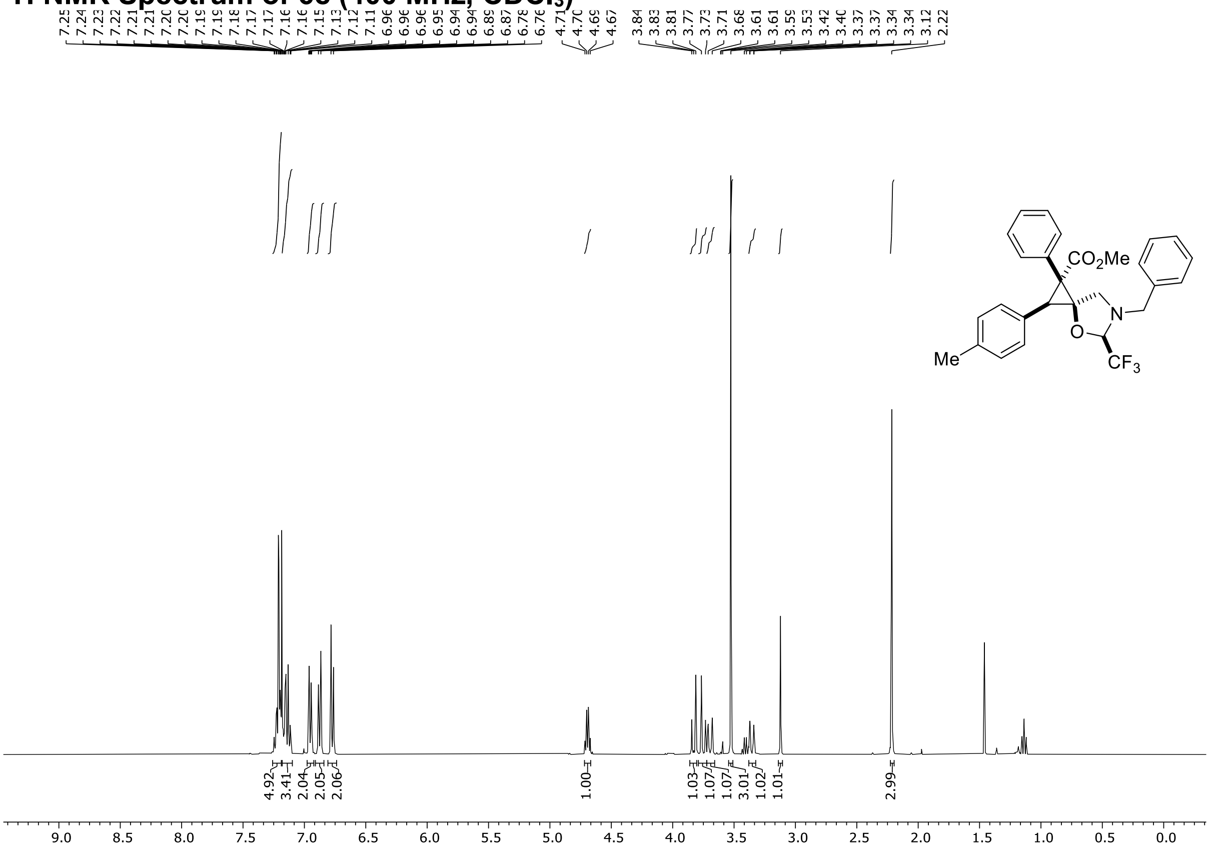

^{13}C NMR Spectrum of 6c (101 MHz, $CDCl_3$)

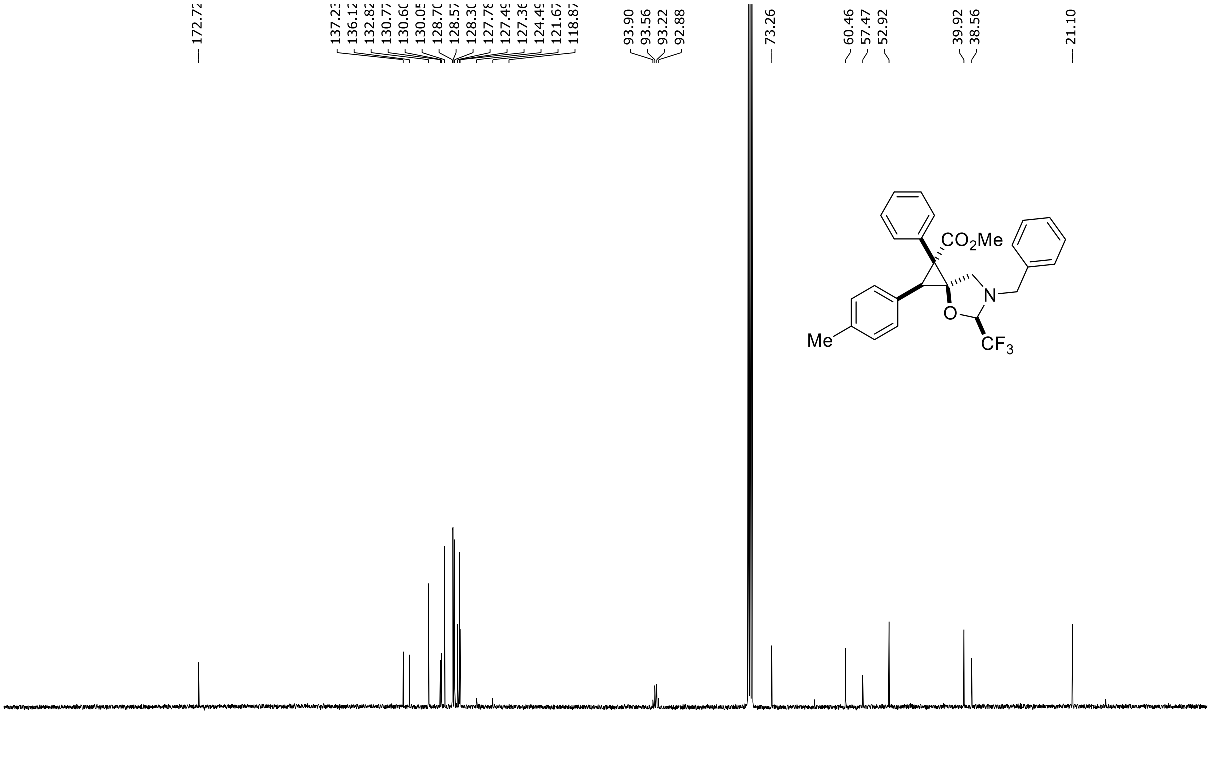

^{19}F NMR Spectrum of 6c (376 MHz, $CDCl_3$)

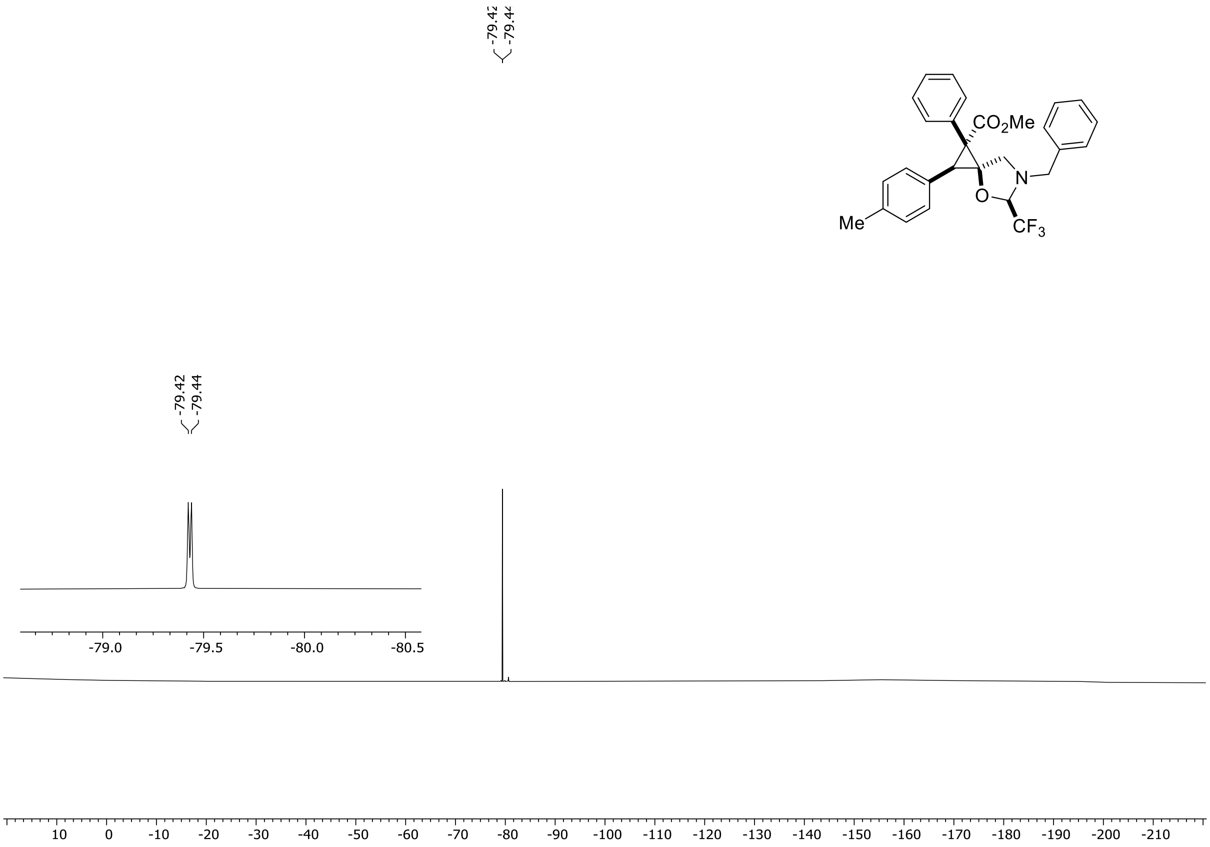

^{1}H NMR Spectrum of 6d (400 MHz, $CDCl_3$)

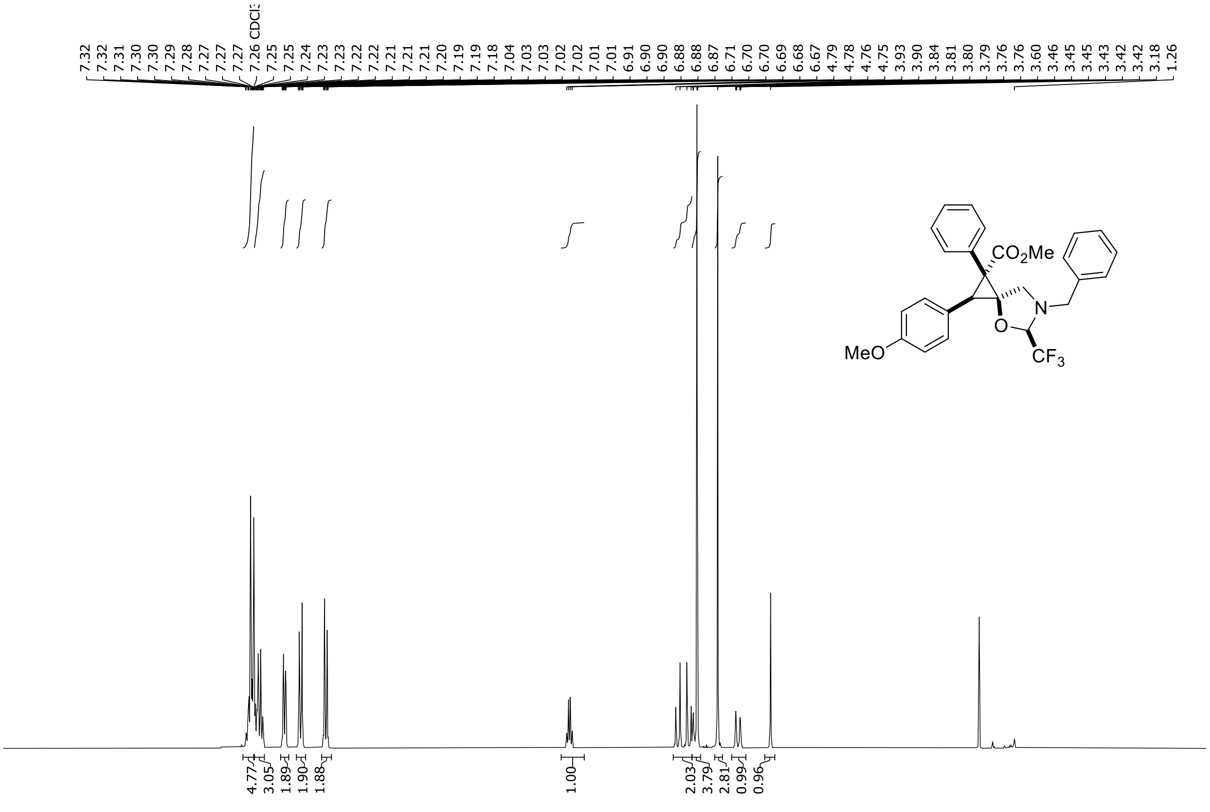

^{13}C NMR Spectrum of 6d (101 MHz, $CDCl_3$)

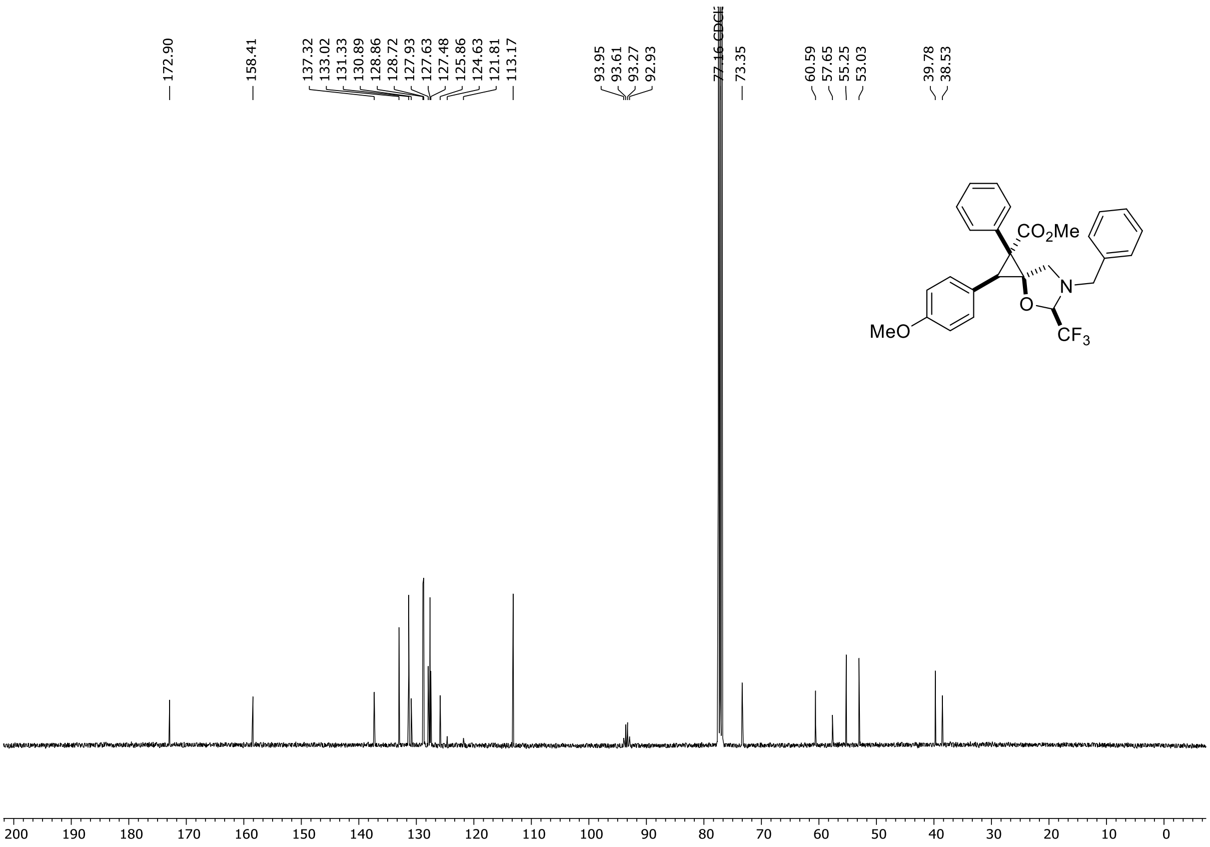

^{19}F NMR Spectrum of 6d (376 MHz, $CDCl_3$)

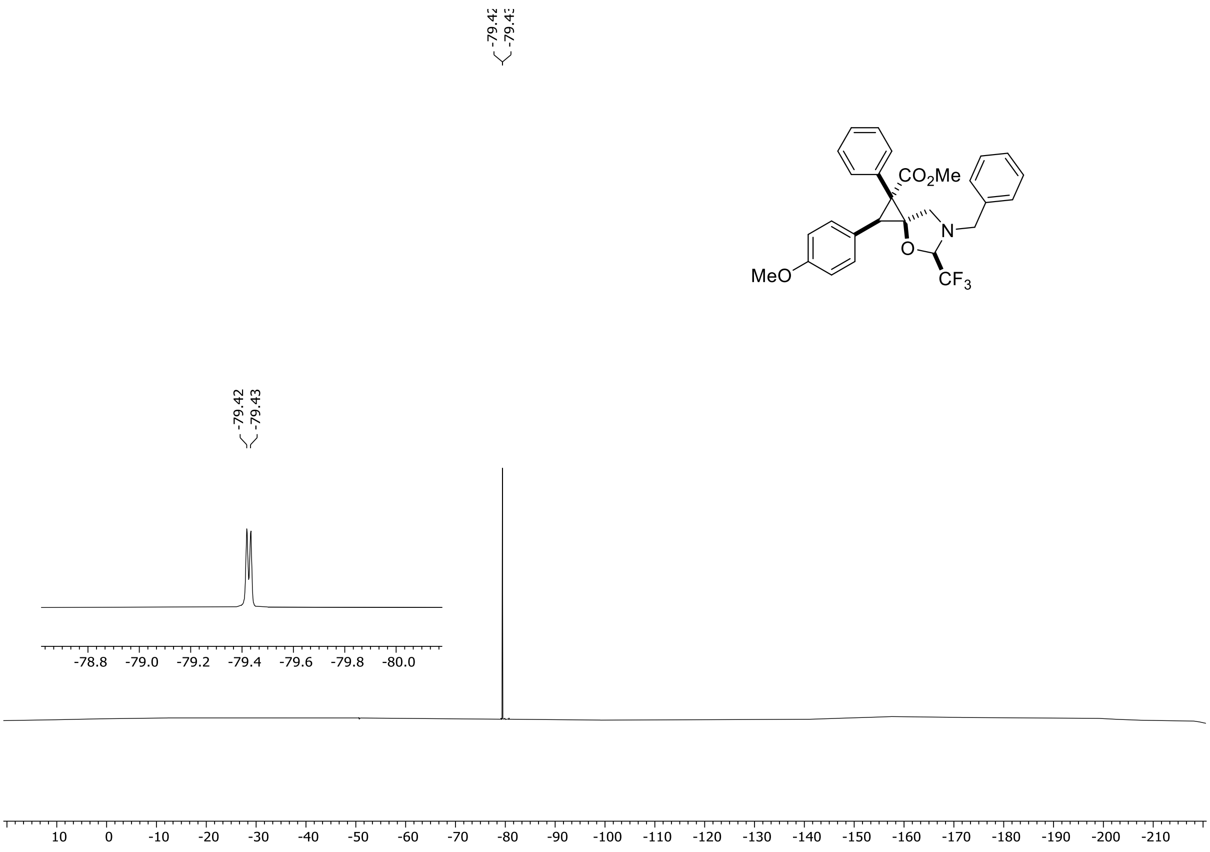

^{1}H NMR Spectrum of 6e (400 MHz, $CDCl_3$)

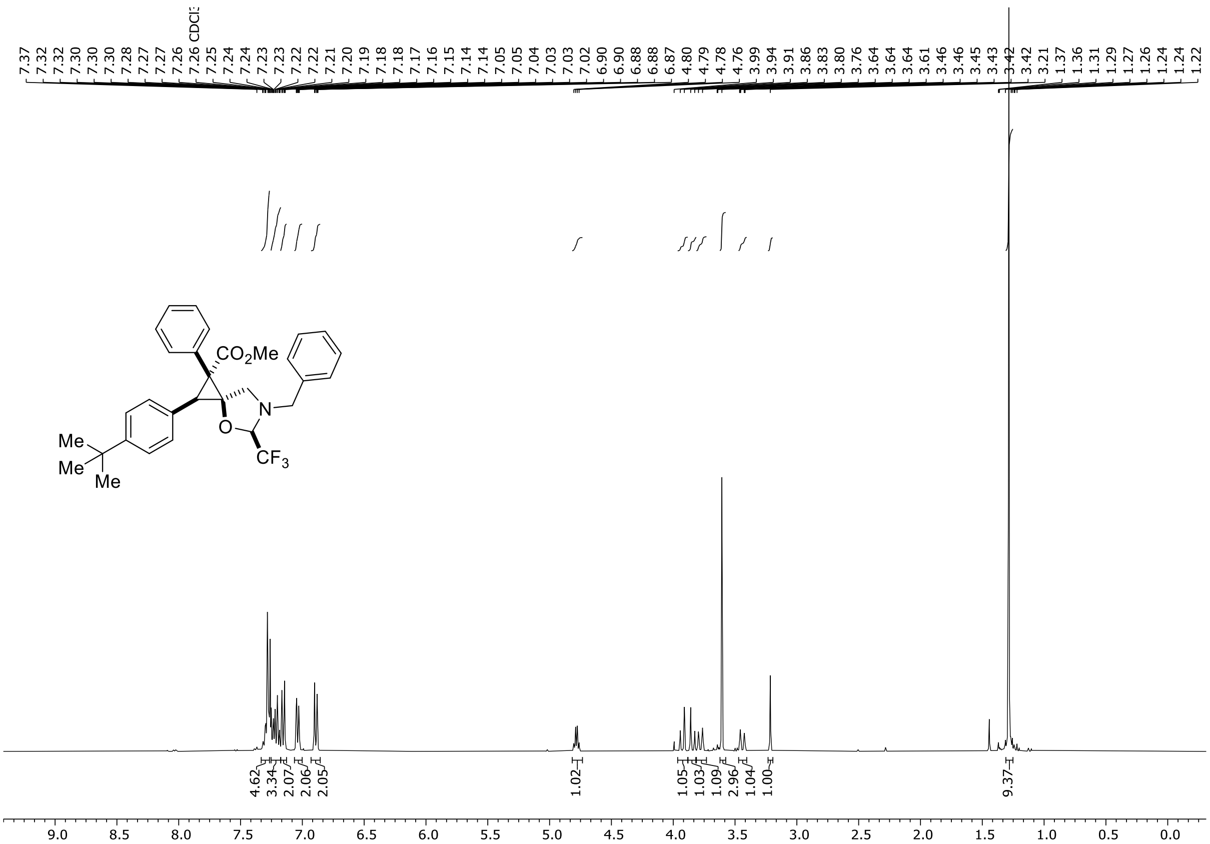

^{13}C NMR Spectrum of 6e (101 MHz, $CDCl_3$)

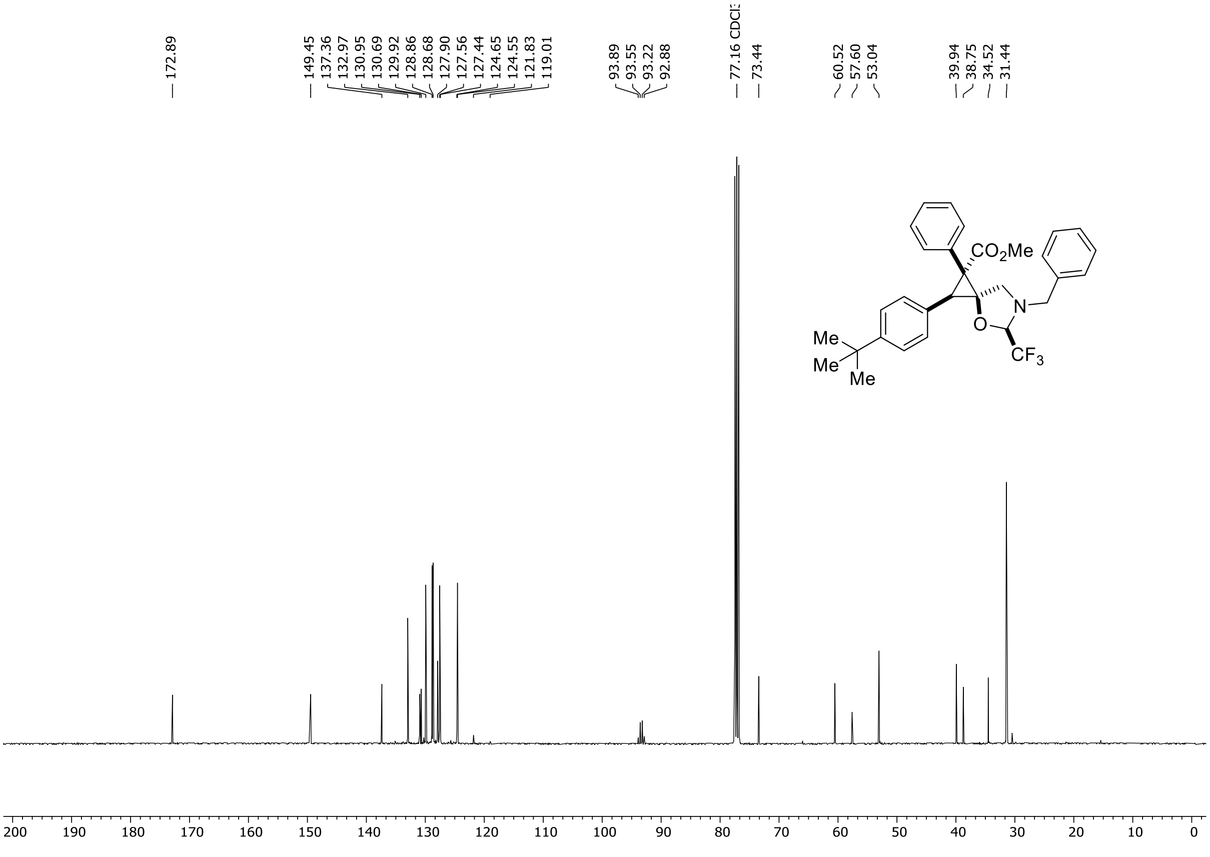

^{19}F NMR Spectrum of 6e (376 MHz, $CDCl_3$)

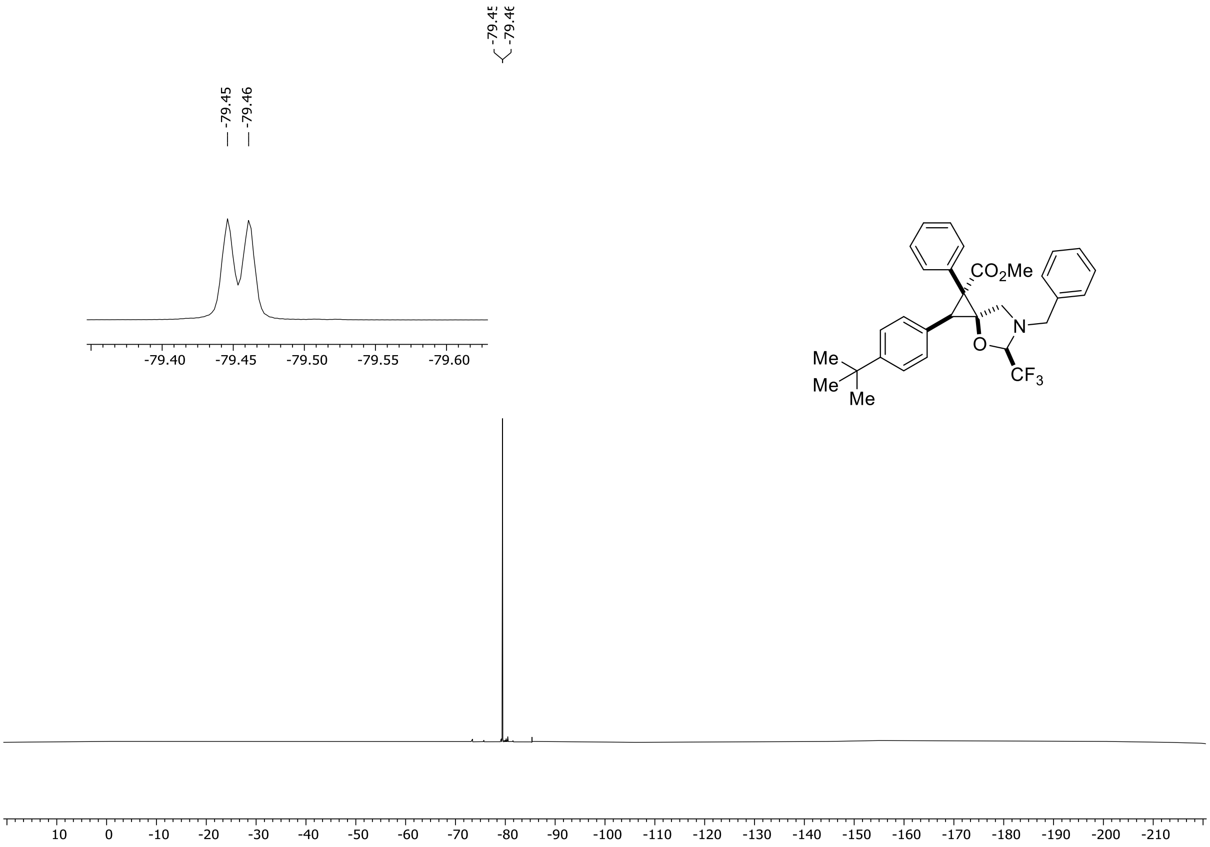

^{1}H NMR Spectrum of 6f (400 MHz, $CDCl_3$)

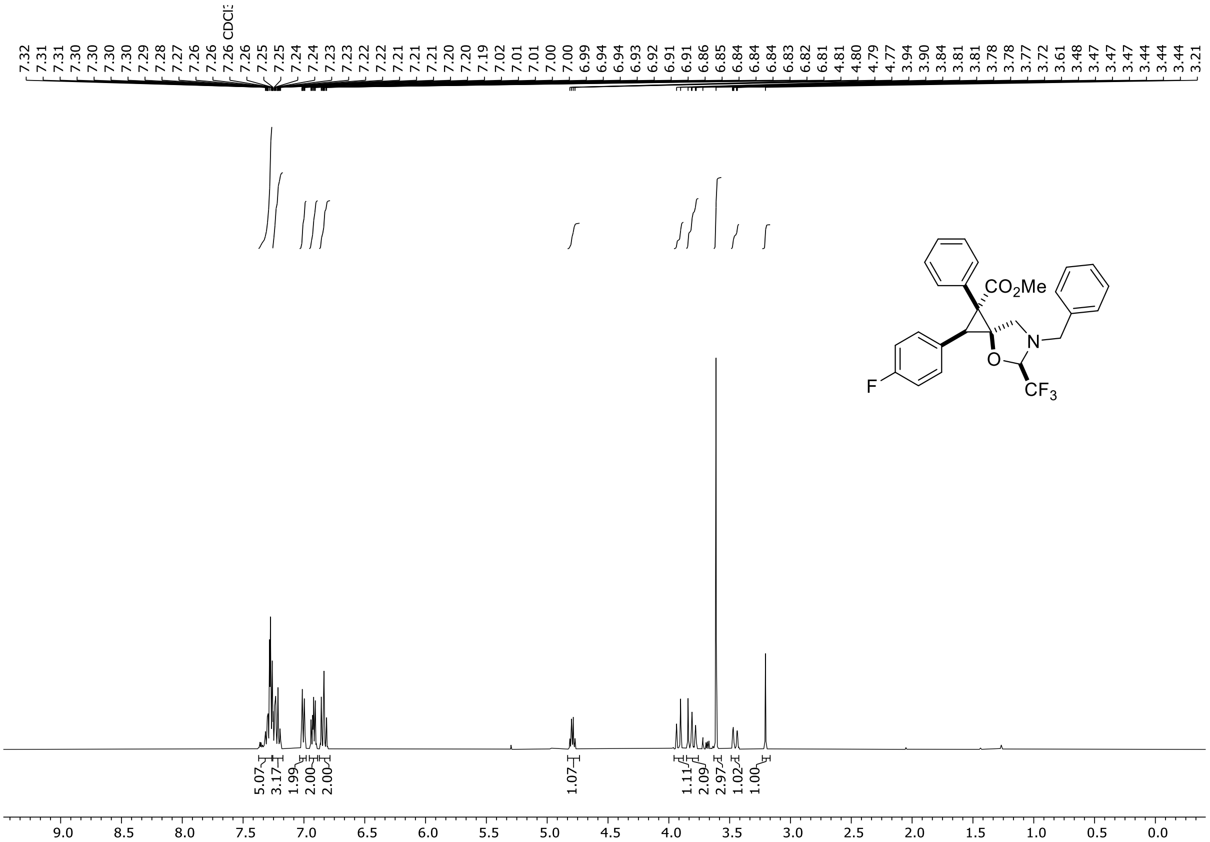

^{13}C NMR Spectrum of 6f (101 MHz, $CDCl_3$)

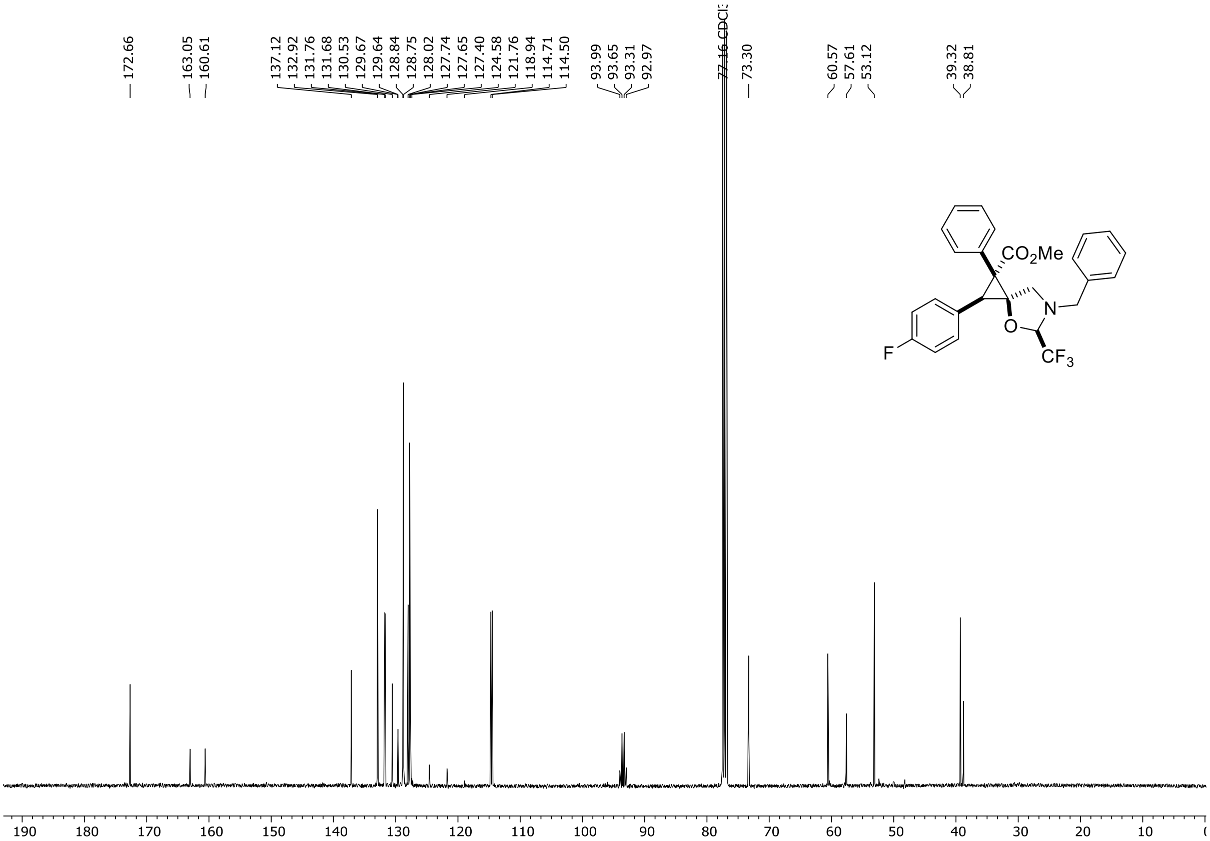

^{19}F NMR Spectrum of 6f (376 MHz, $CDCl_3$)

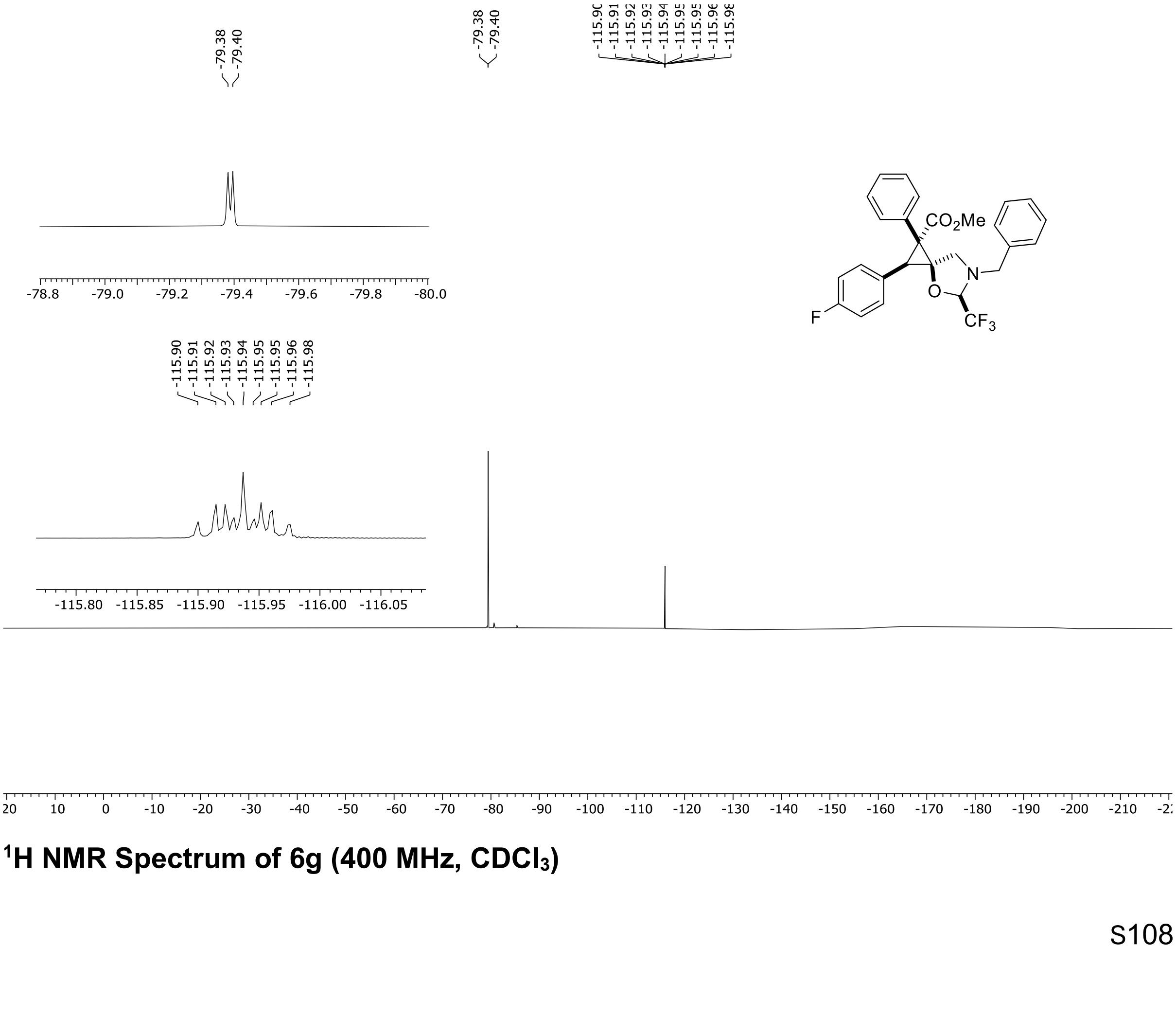

^{1}H NMR Spectrum of 6g (400 MHz, $CDCl_3$)

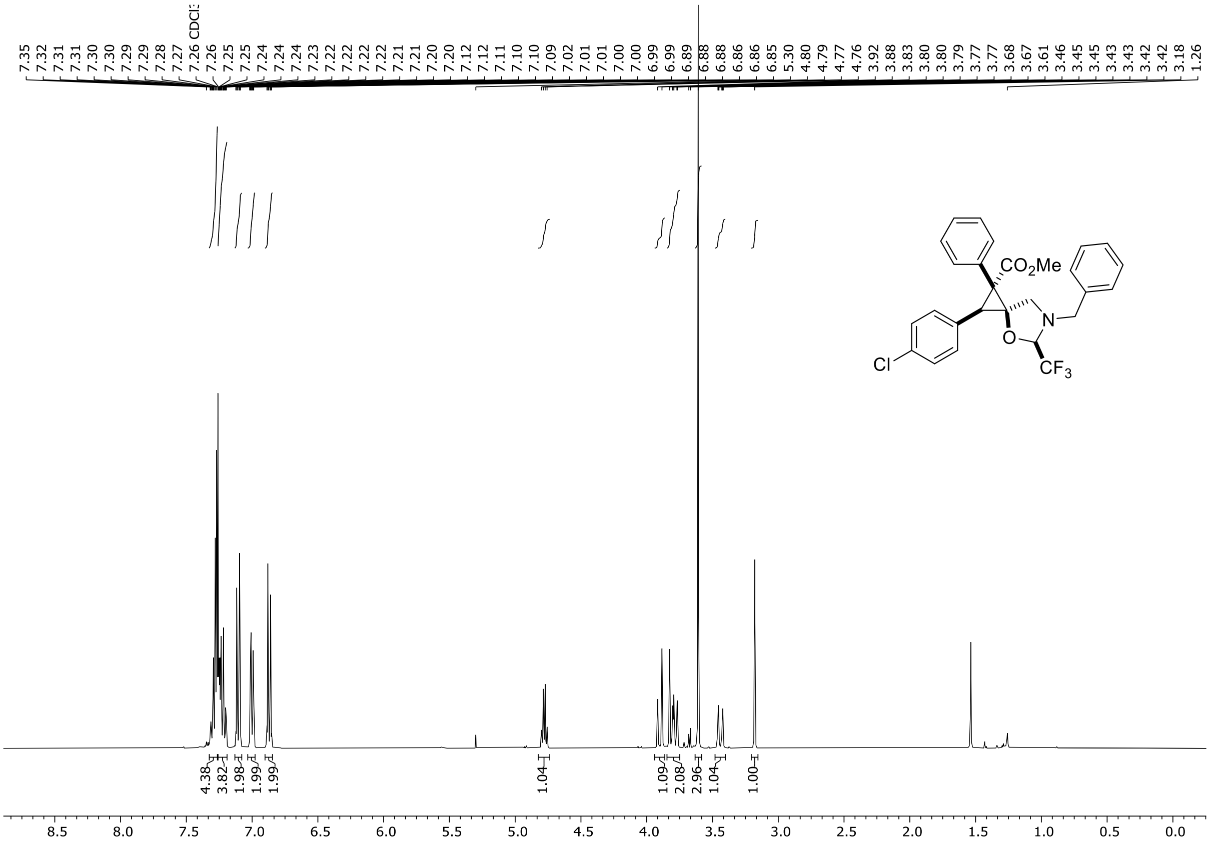

^{13}C NMR Spectrum of 6g (101 MHz, $CDCl_3$)

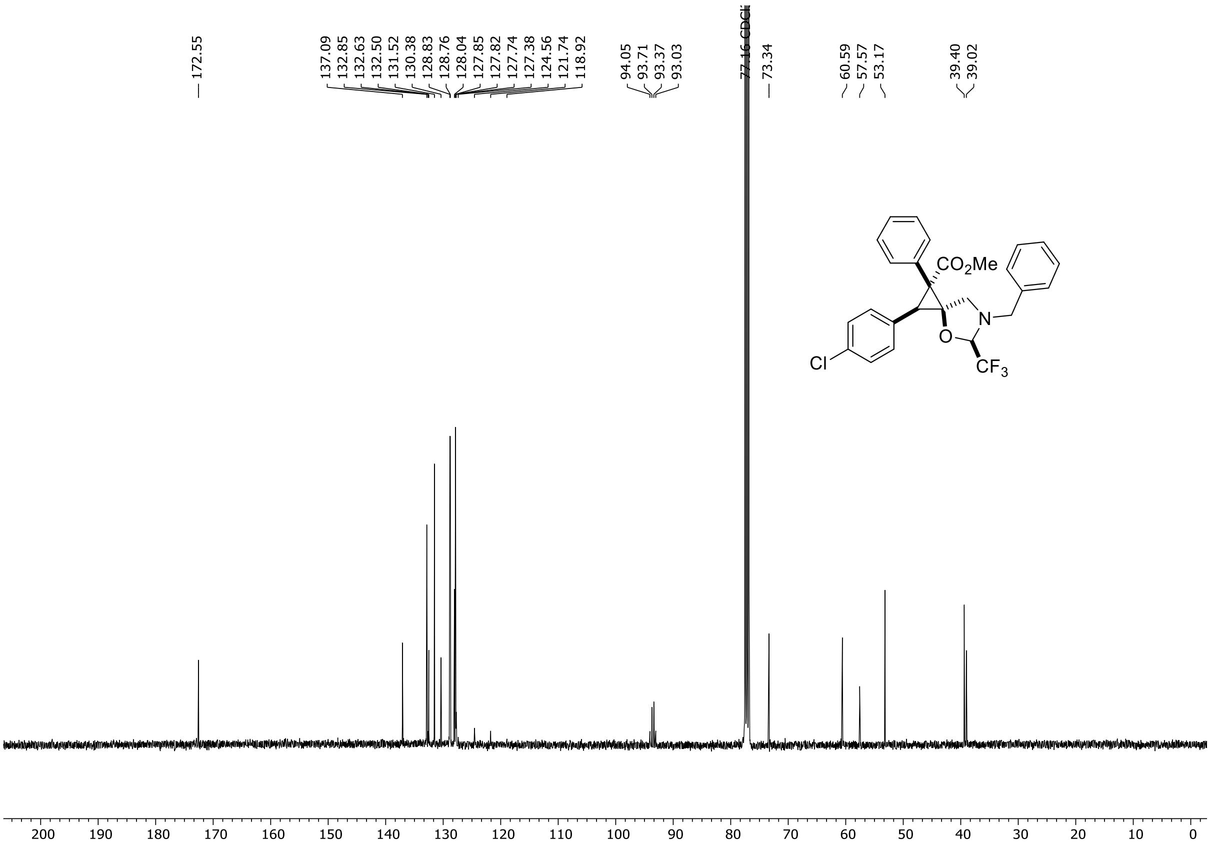

^{19}F NMR Spectrum of 6g (376 MHz, $CDCl_3$)

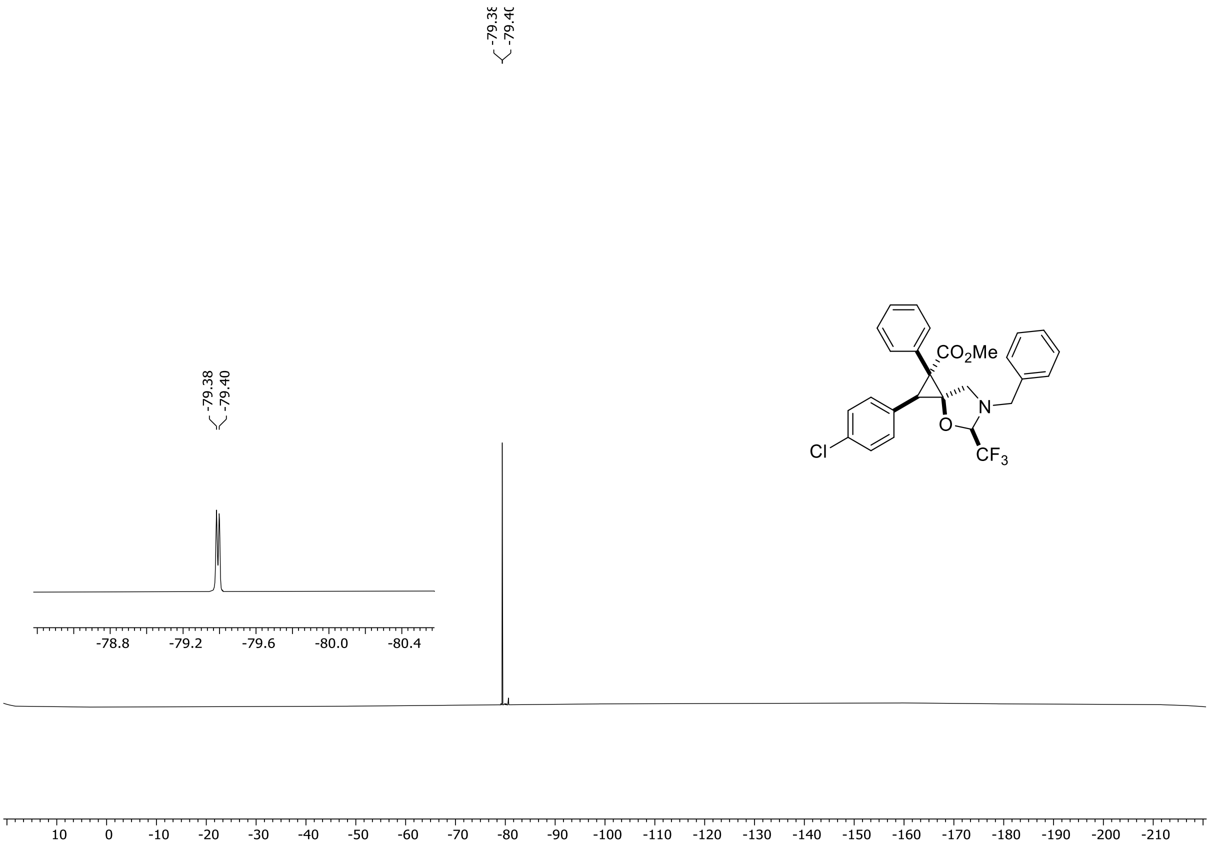

^{1}H NMR Spectrum of 497 6h (400 MHz, $CDCl_3$)

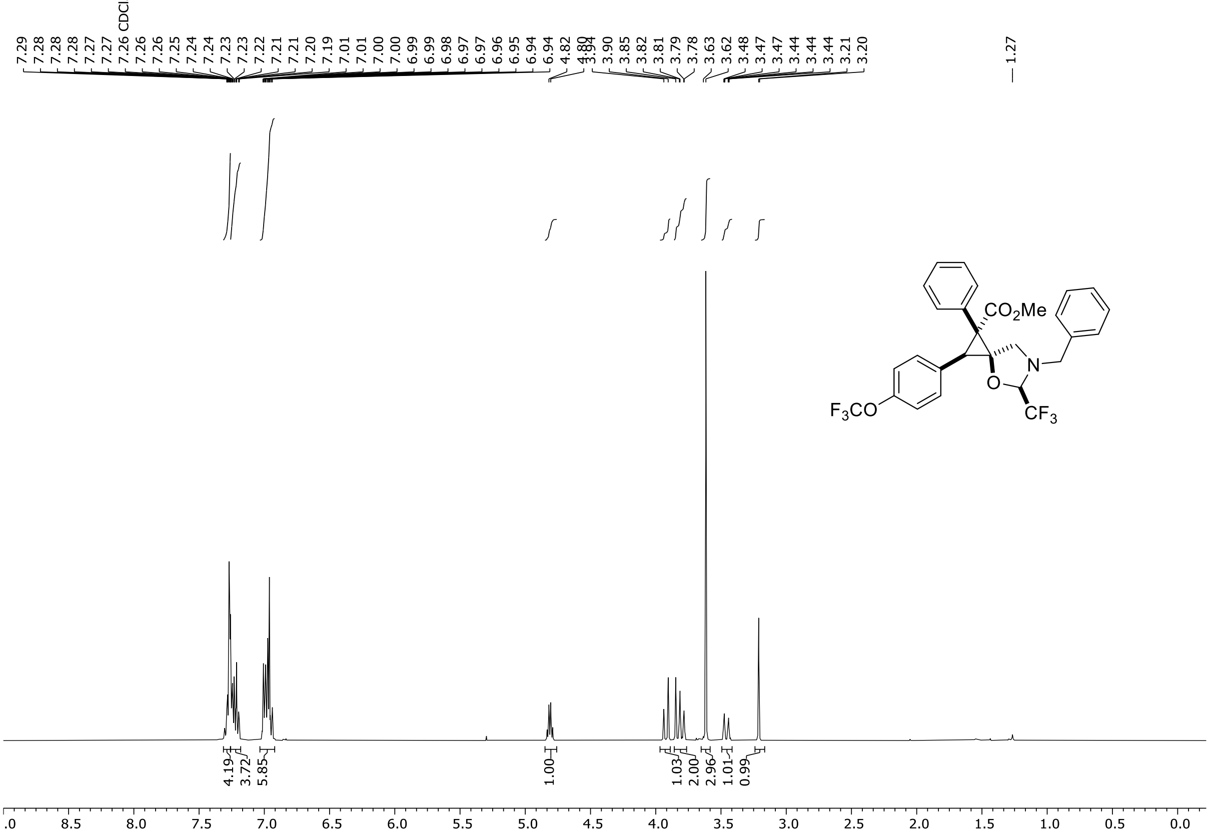

^{13}C NMR Spectrum of 6h (101 MHz, $CDCl_3$)

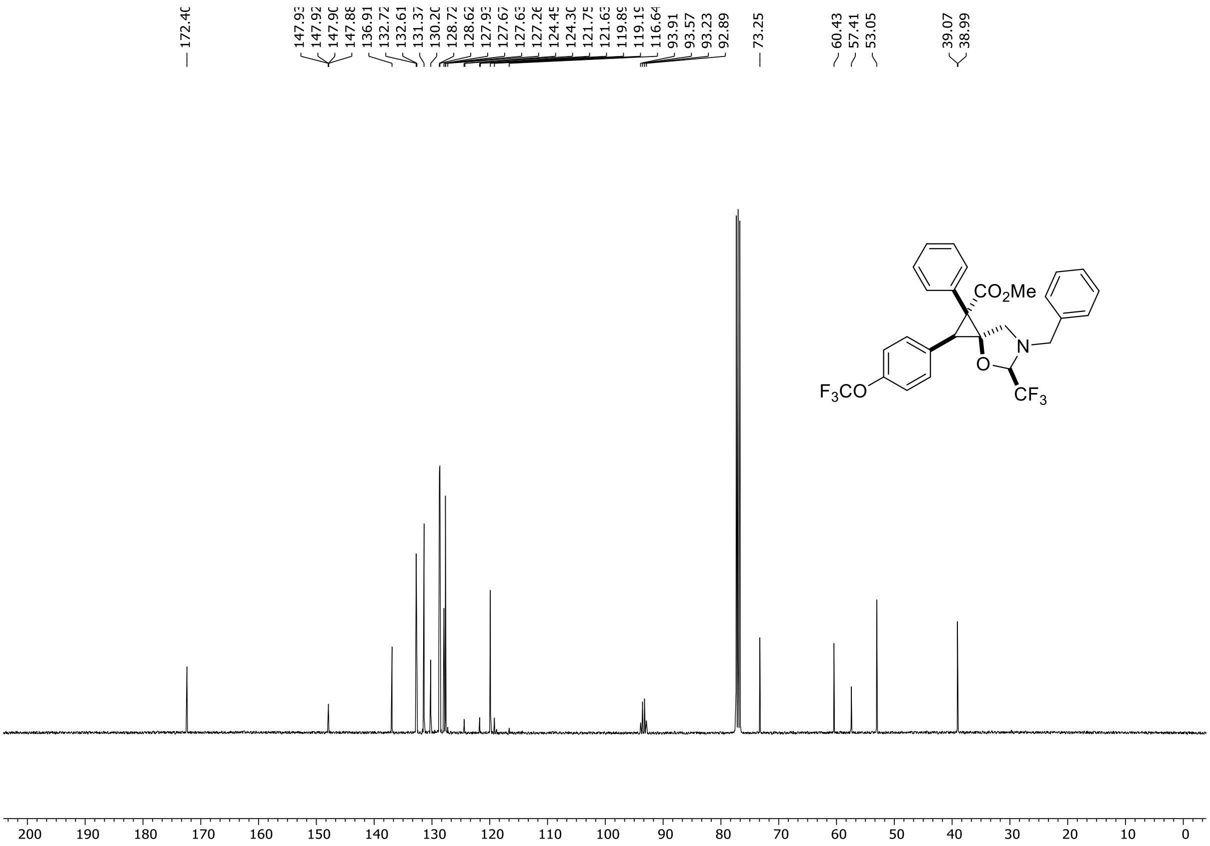

^{19}F NMR Spectrum of 6h (376 MHz, $CDCl_3$)

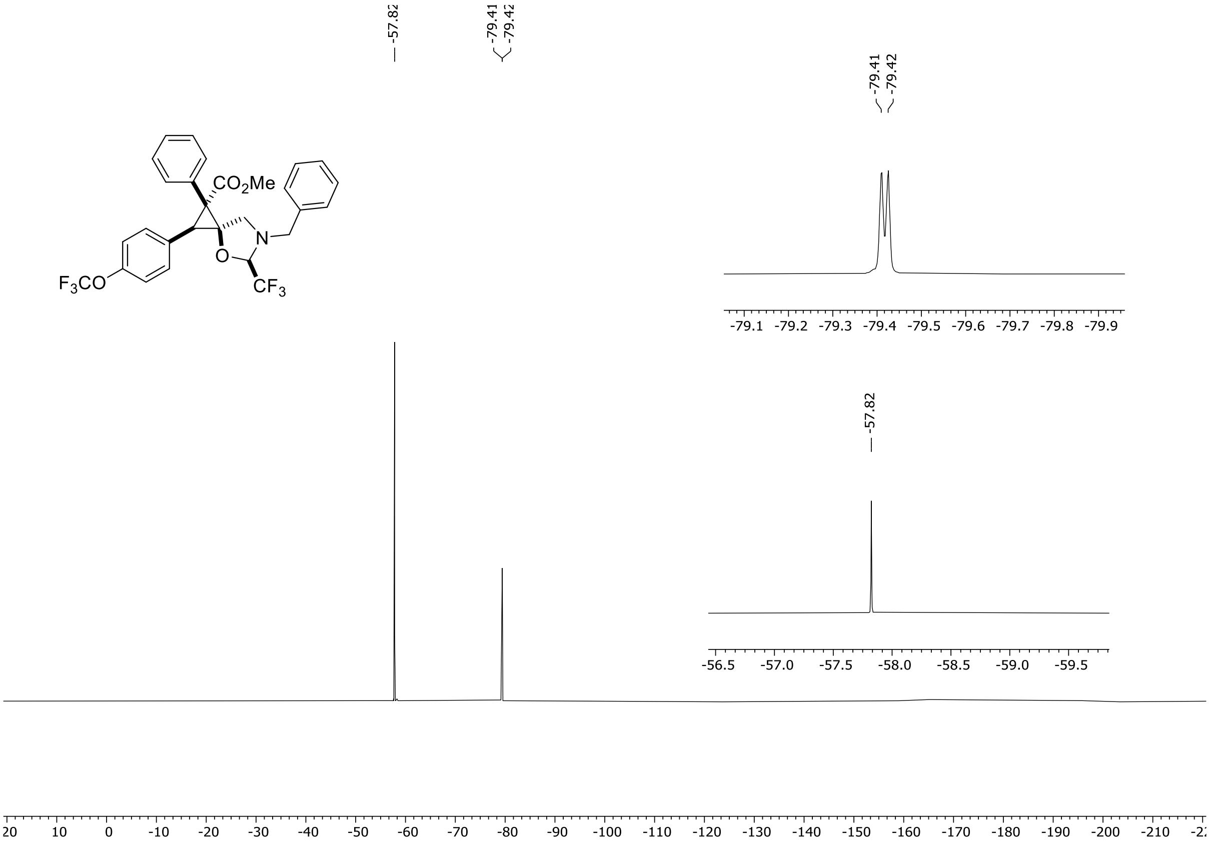

^{1}H NMR Spectrum of 450 6i (400 MHz, $CDCl_3$)

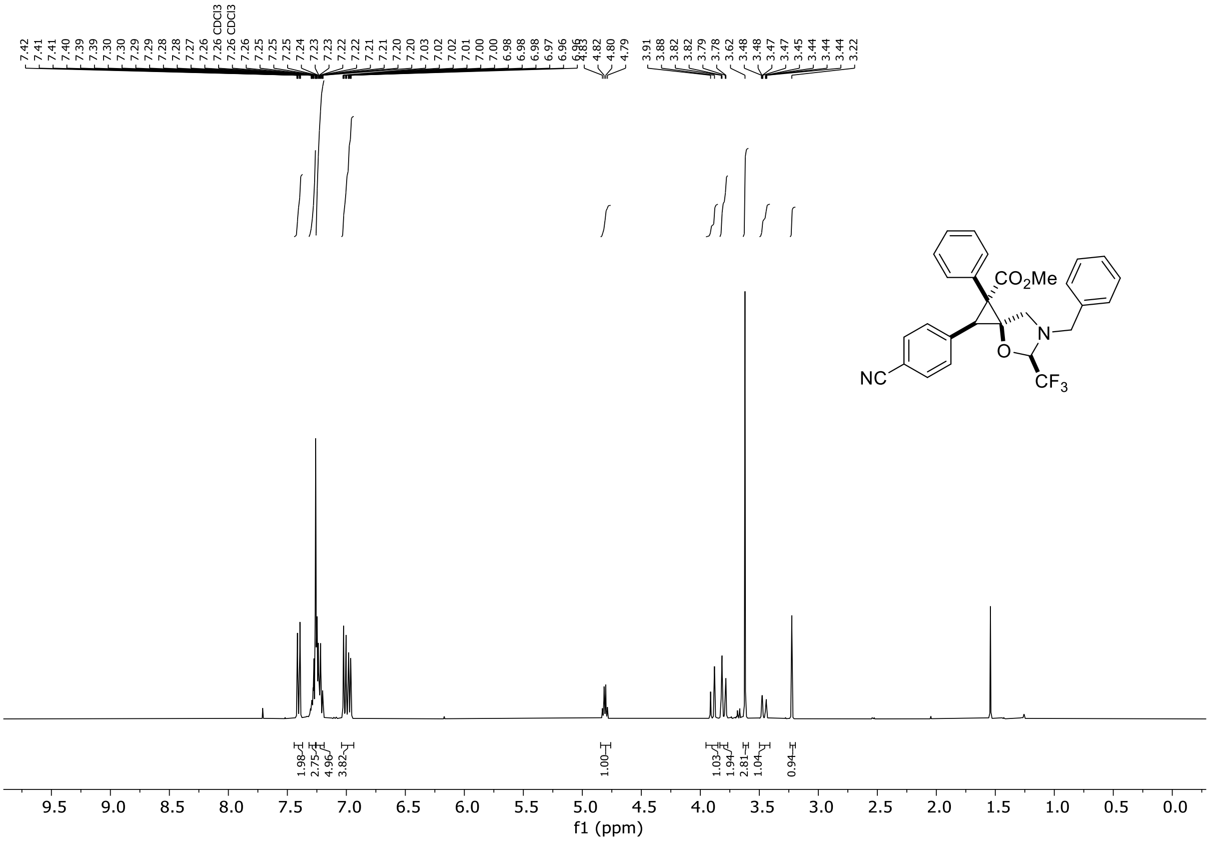

^{13}C NMR Spectrum of 6i (101 MHz, $CDCl_3$)

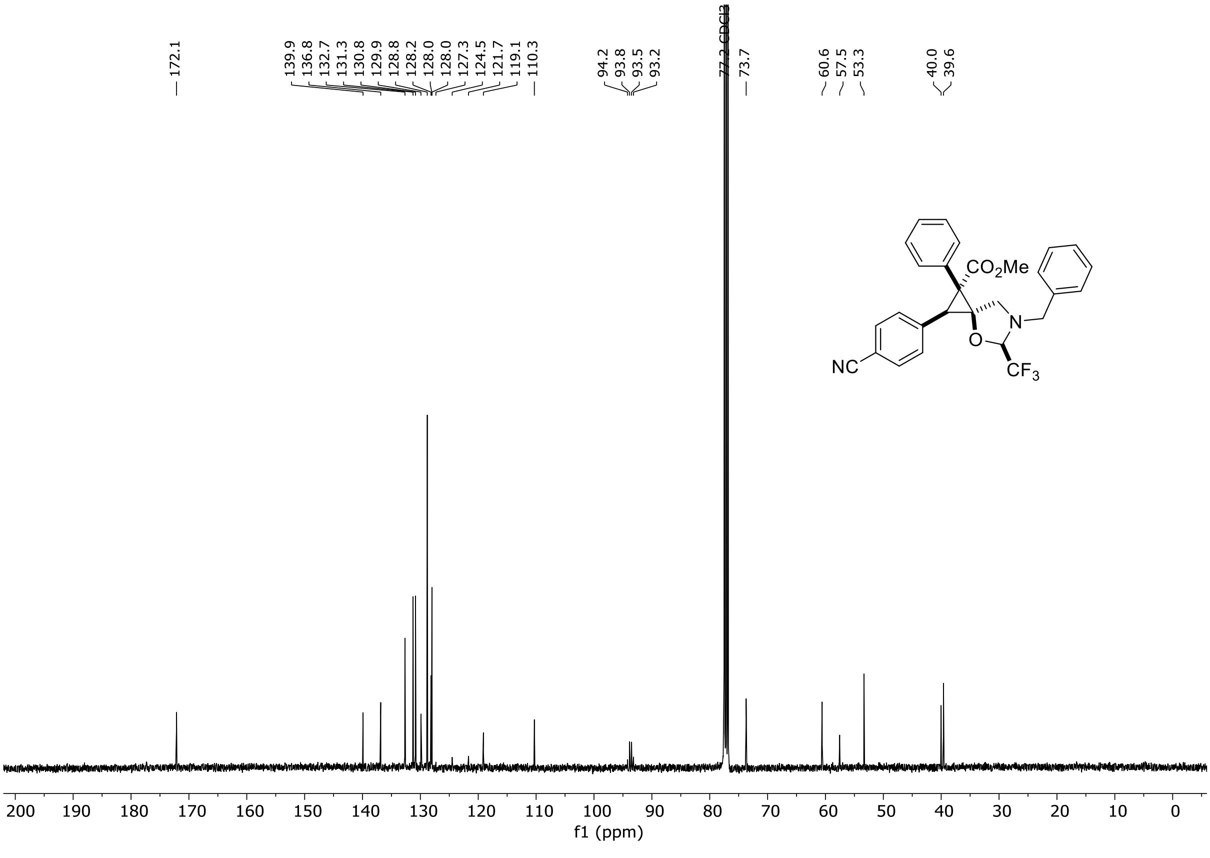

^{19}F NMR Spectrum of 6i (376 MHz, $CDCl_3$)

DKB-01-450.15.fid

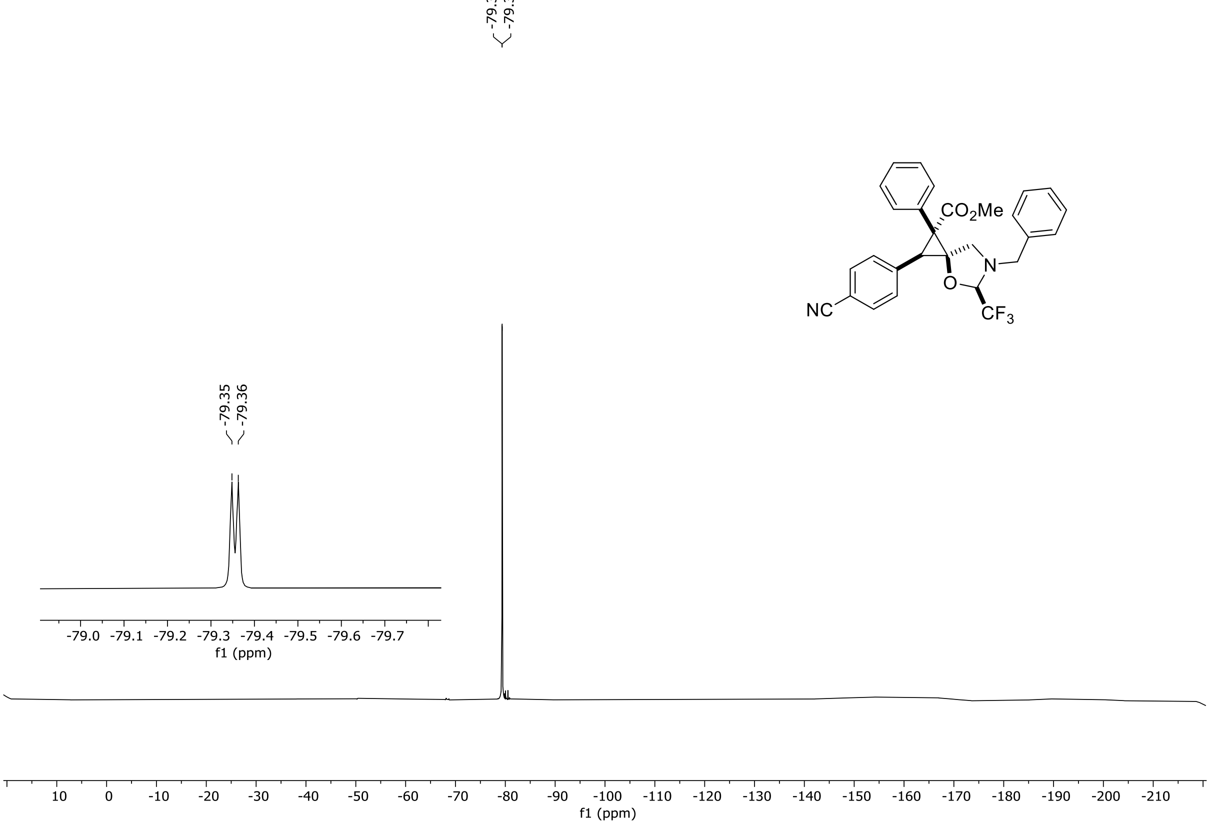

^{1}H NMR Spectrum of 521 6j (400 MHz, $CDCl_3$)

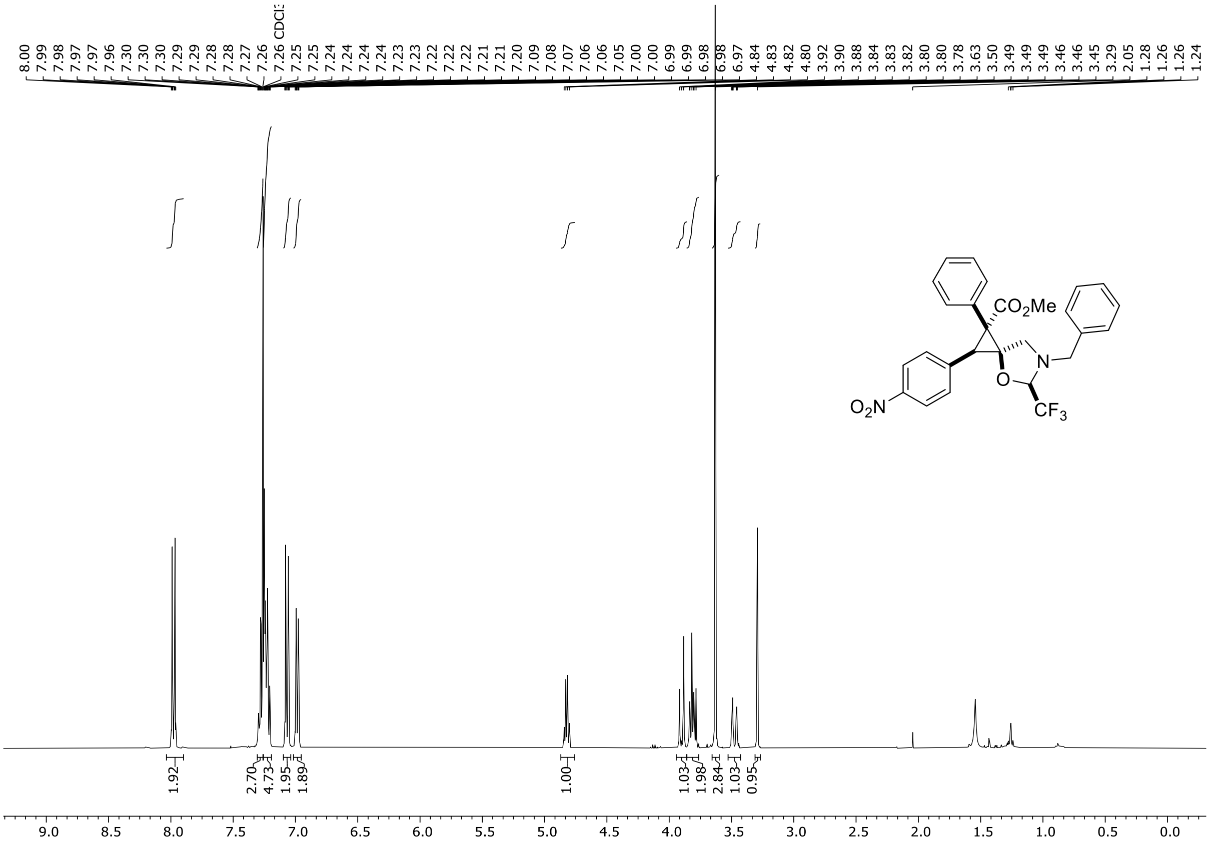

^{13}C NMR Spectrum of 6j (101 MHz, $CDCl_3$)

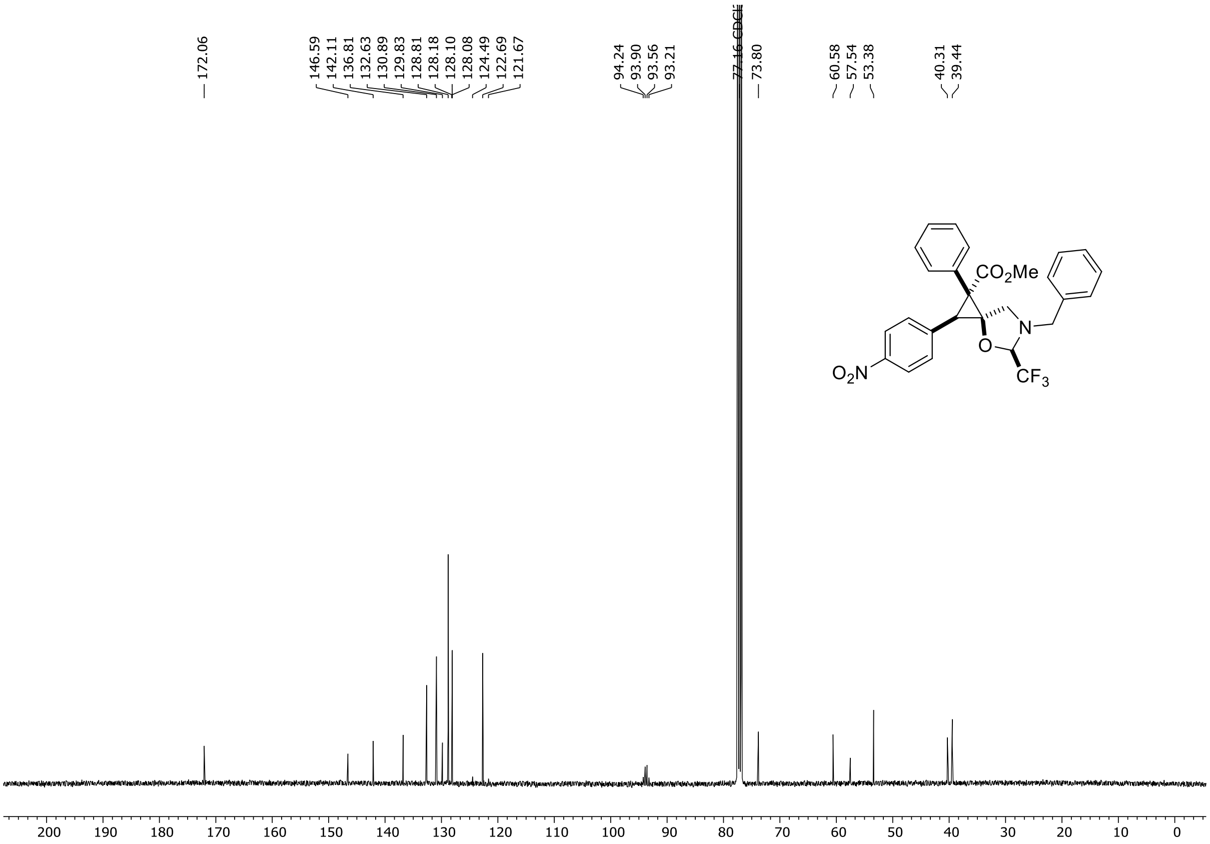

^{19}F NMR Spectrum of 6j (376 MHz, $CDCl_3$)

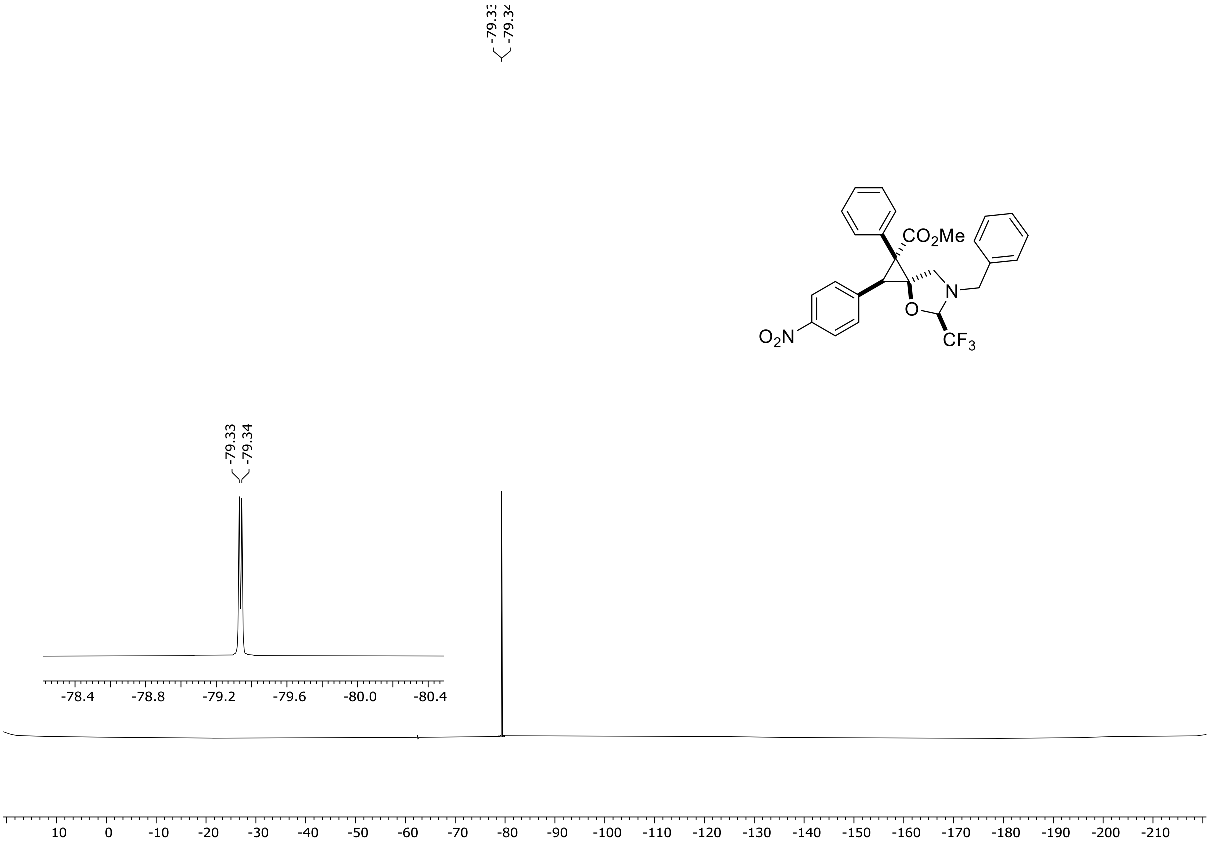

^{1}H NMR Spectrum of 6k (400 MHz, $CDCl_3$)

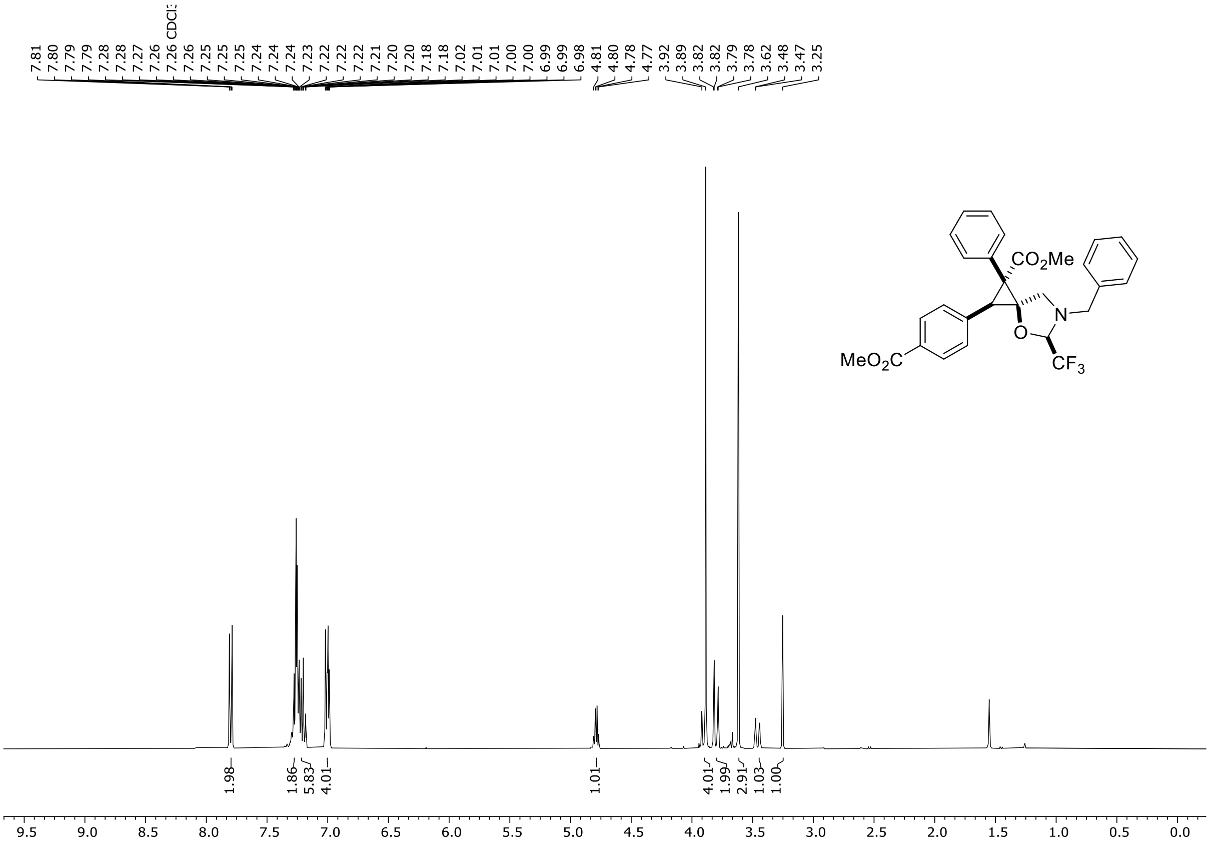

^{13}C NMR Spectrum of 6k (101 MHz, $CDCl_3$)

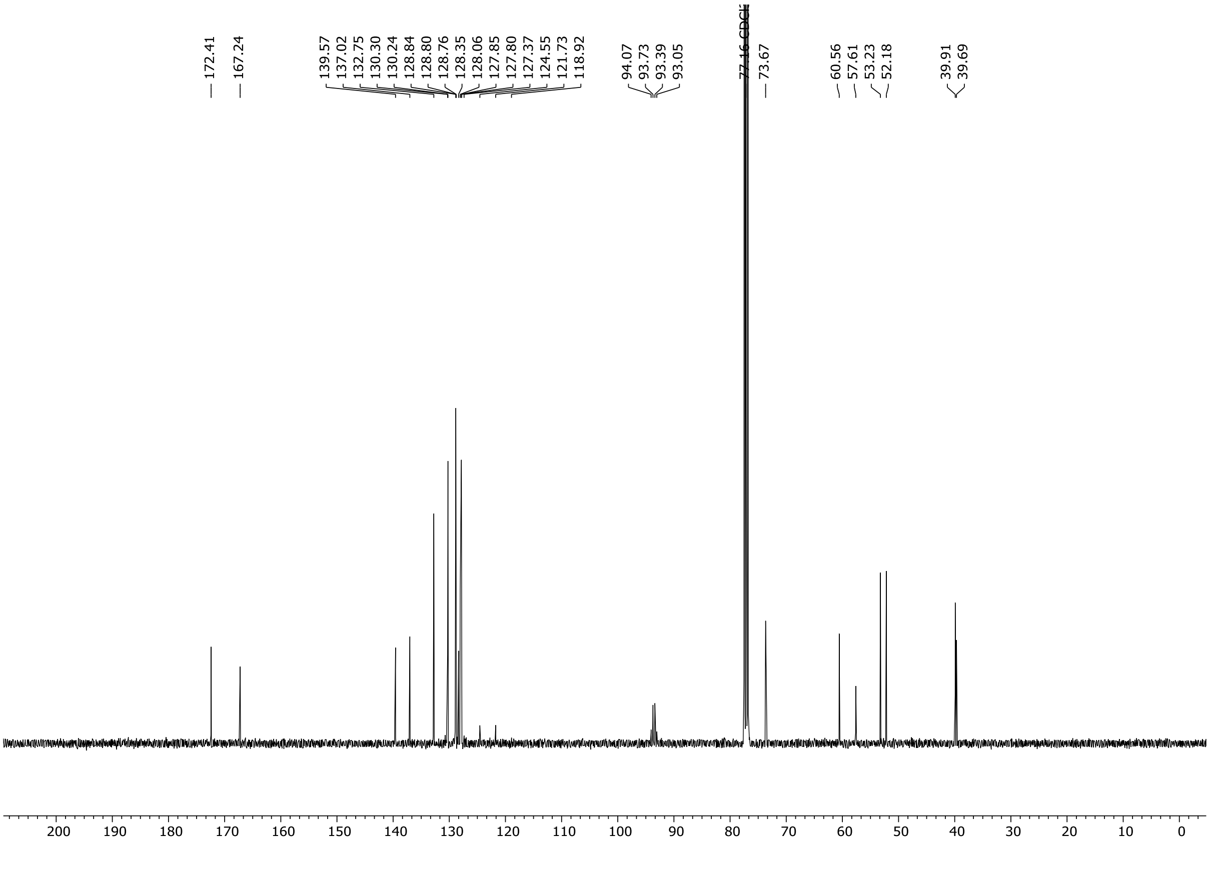

^{19}F NMR Spectrum of 6k (376 MHz, $CDCl_3$)

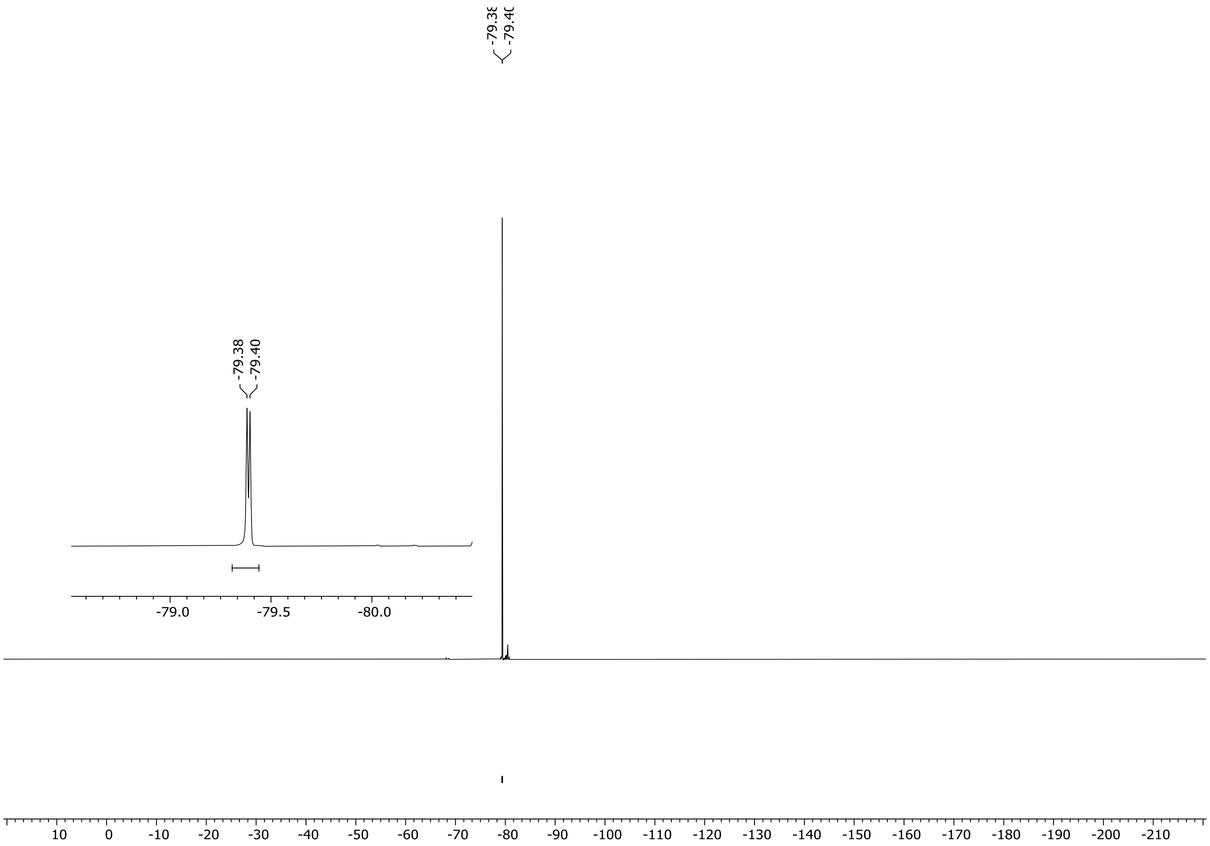

^{1}H NMR Spectrum of 492 6l (400 MHz, $CDCl_3$)

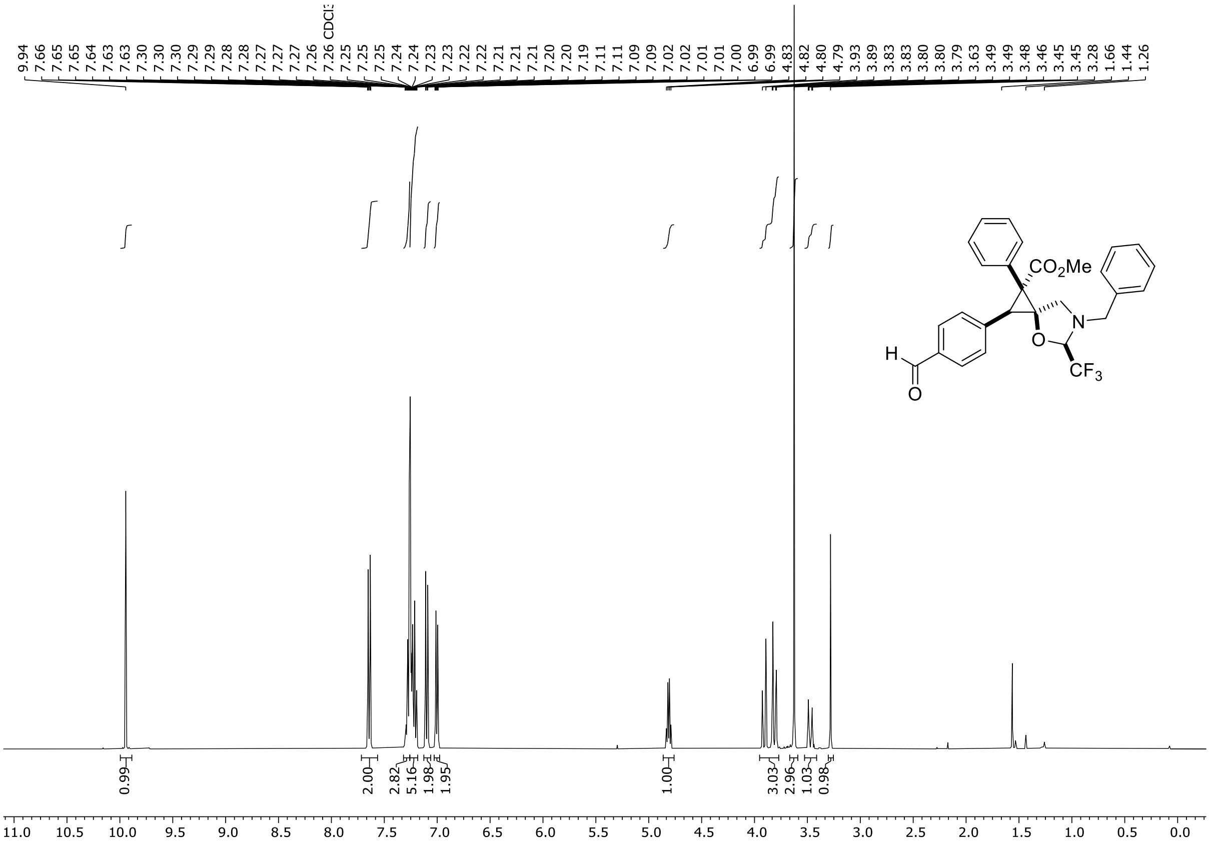

^{13}C NMR Spectrum of 6l (101 MHz, $CDCl_3$)

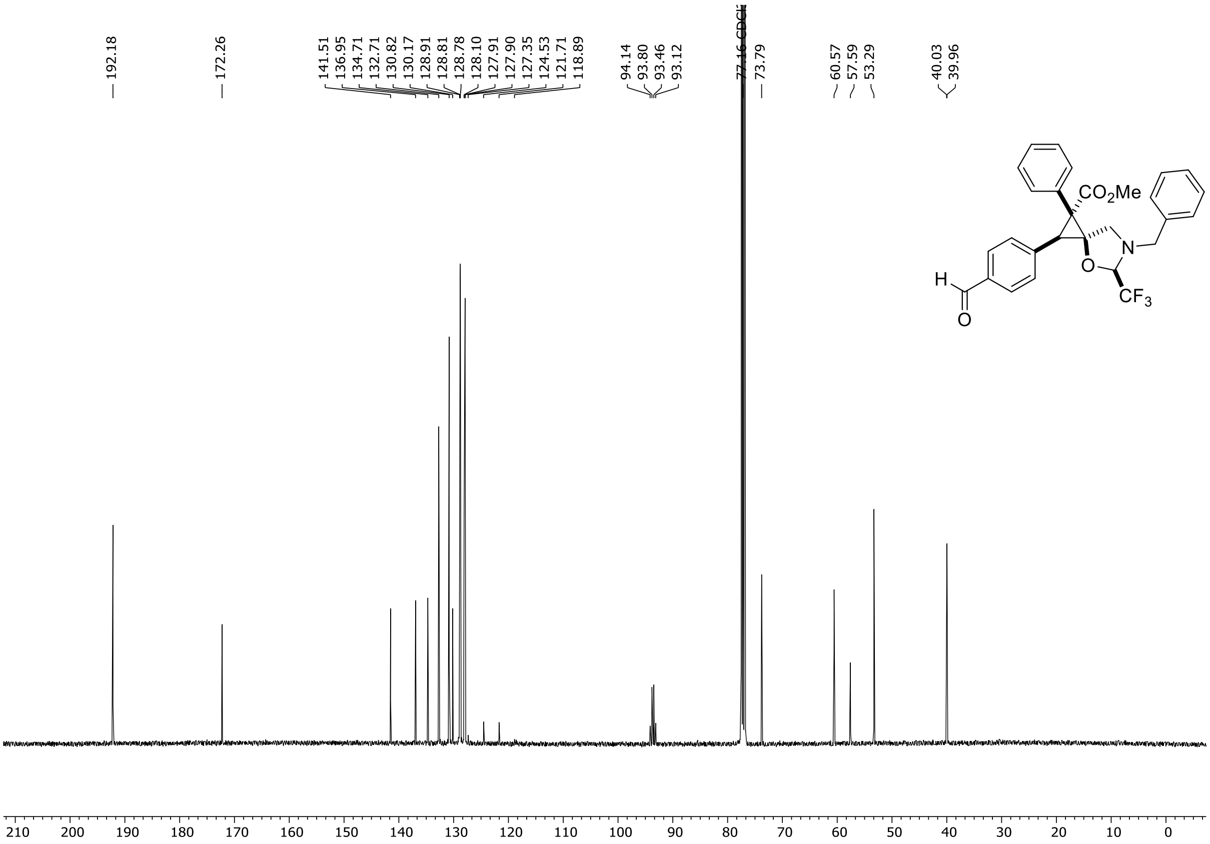

^{19}F NMR Spectrum of 6l (376 MHz, $CDCl_3$)

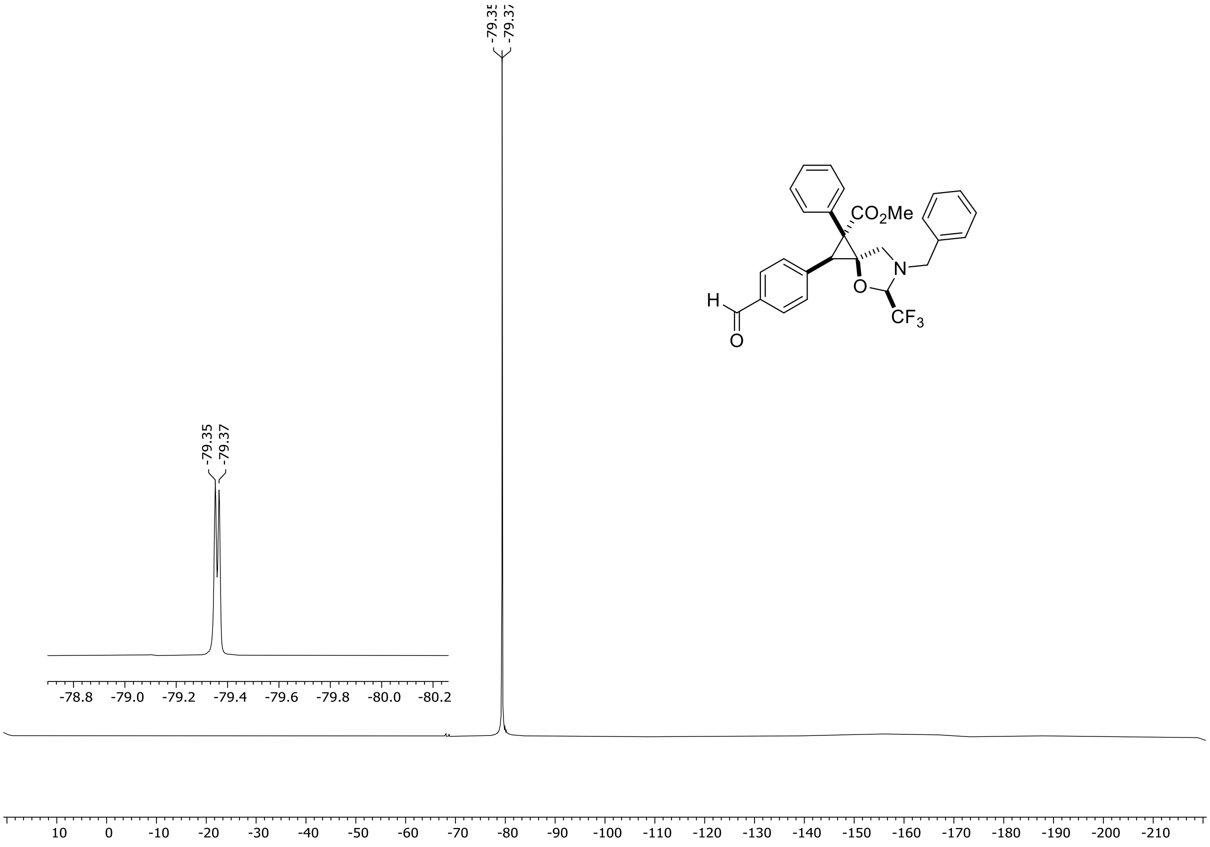

^{1}H NMR Spectrum of 6m (400 MHz, $CDCl_3$)

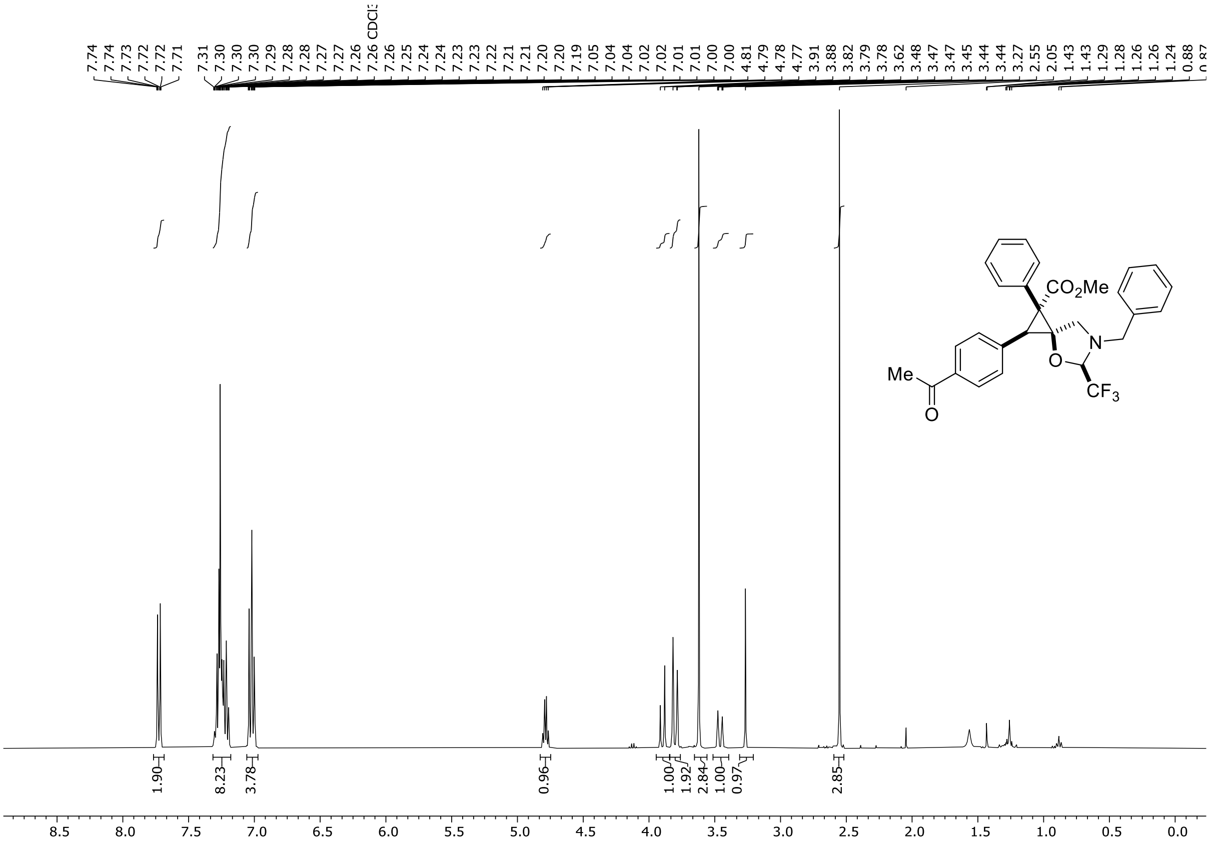

^{13}C NMR Spectrum of 6m (101 MHz, $CDCl_3$)

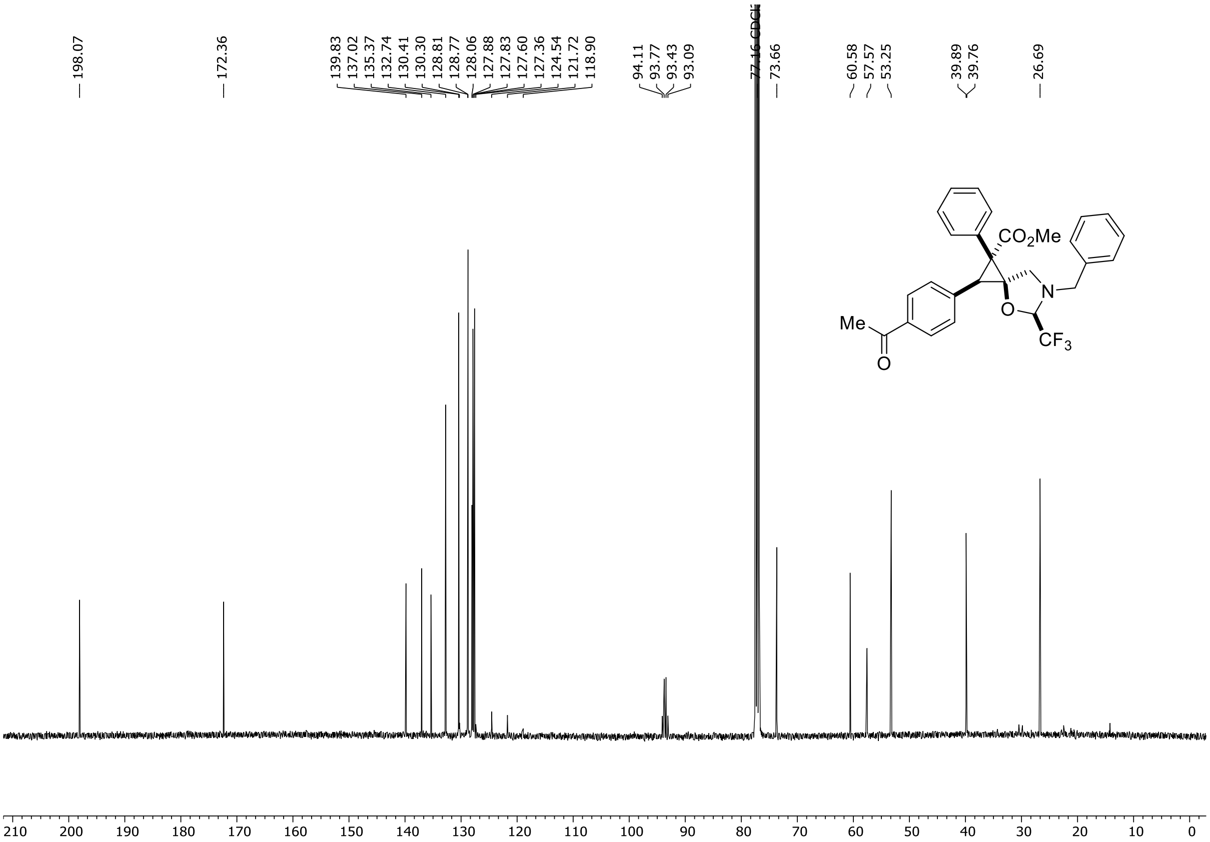

^{19}F NMR Spectrum of 6m (376 MHz, $CDCl_3$)

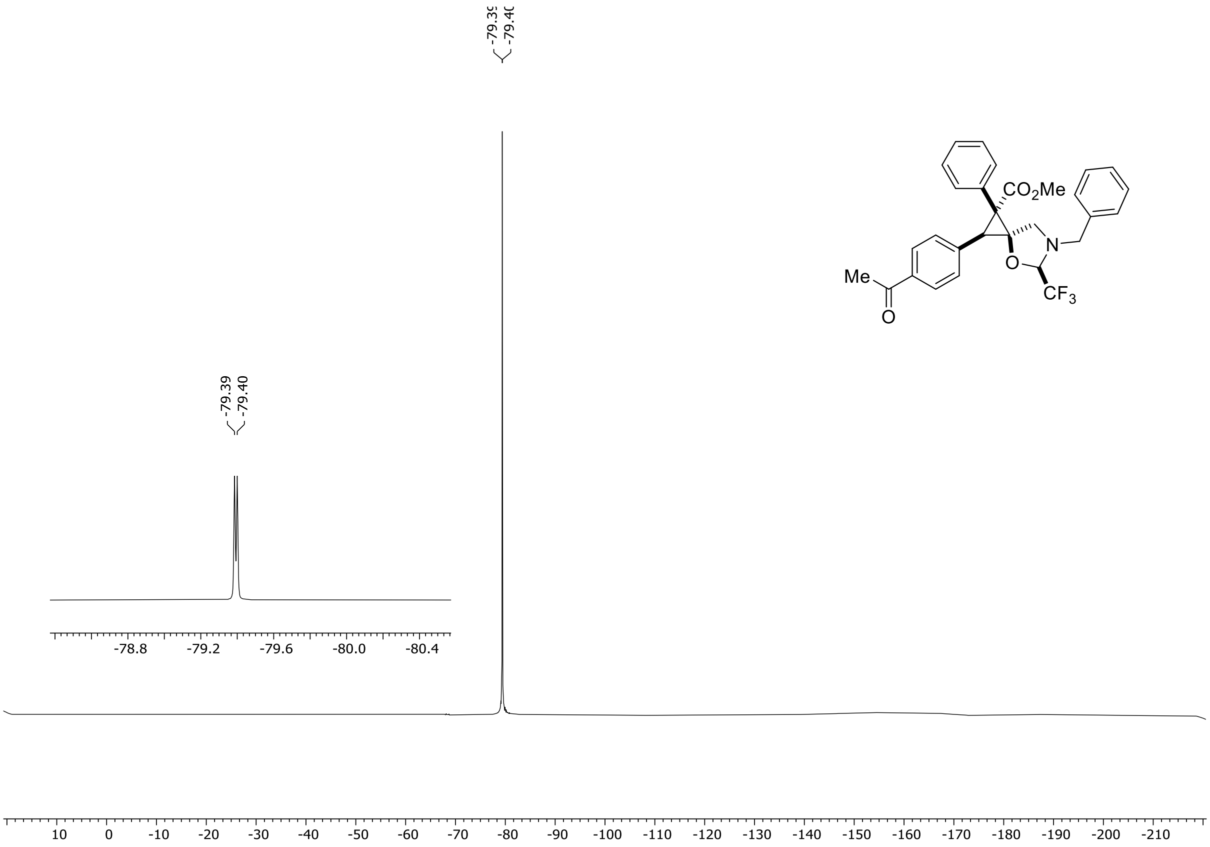

^{1}H NMR Spectrum of 6n (400 MHz, $CDCl_3$)

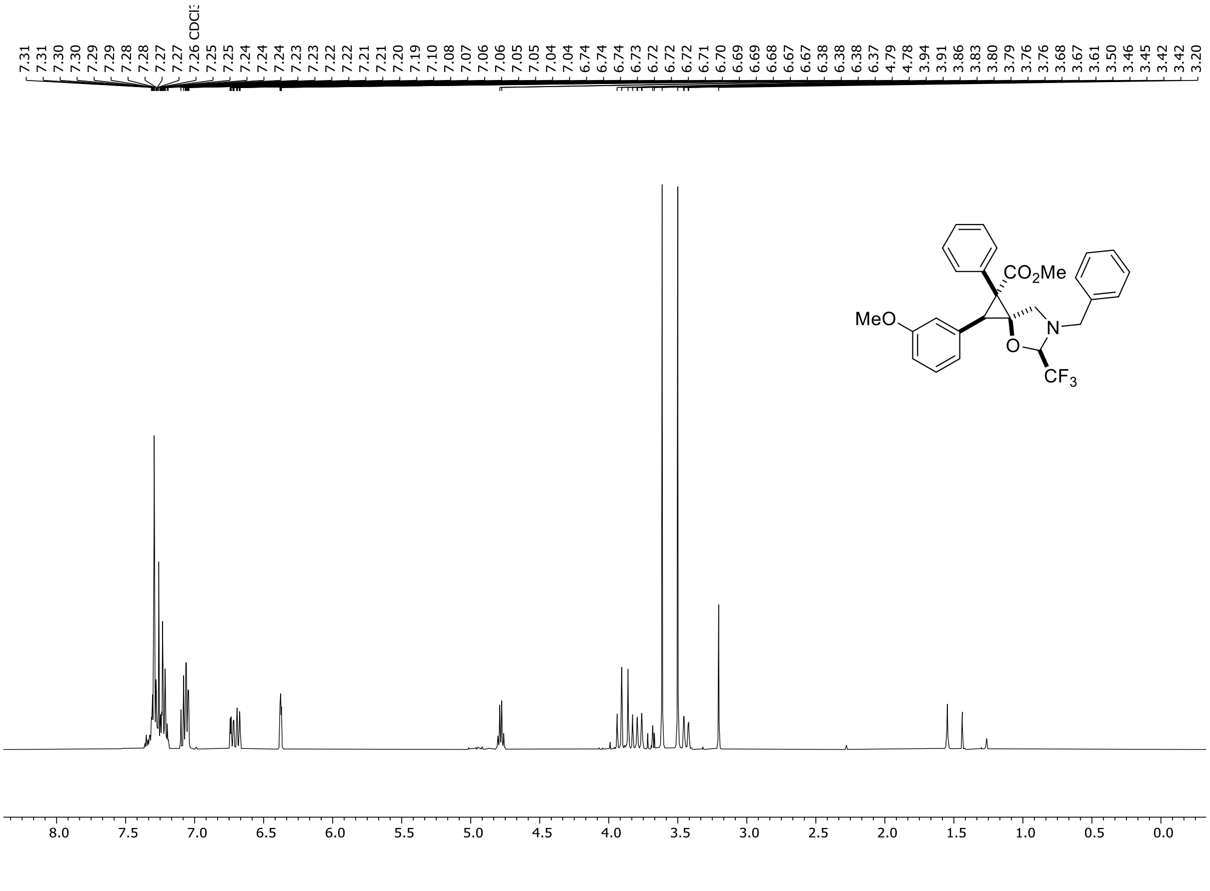

^{13}C NMR Spectrum of 6n (101 MHz, CDCl$_3$)

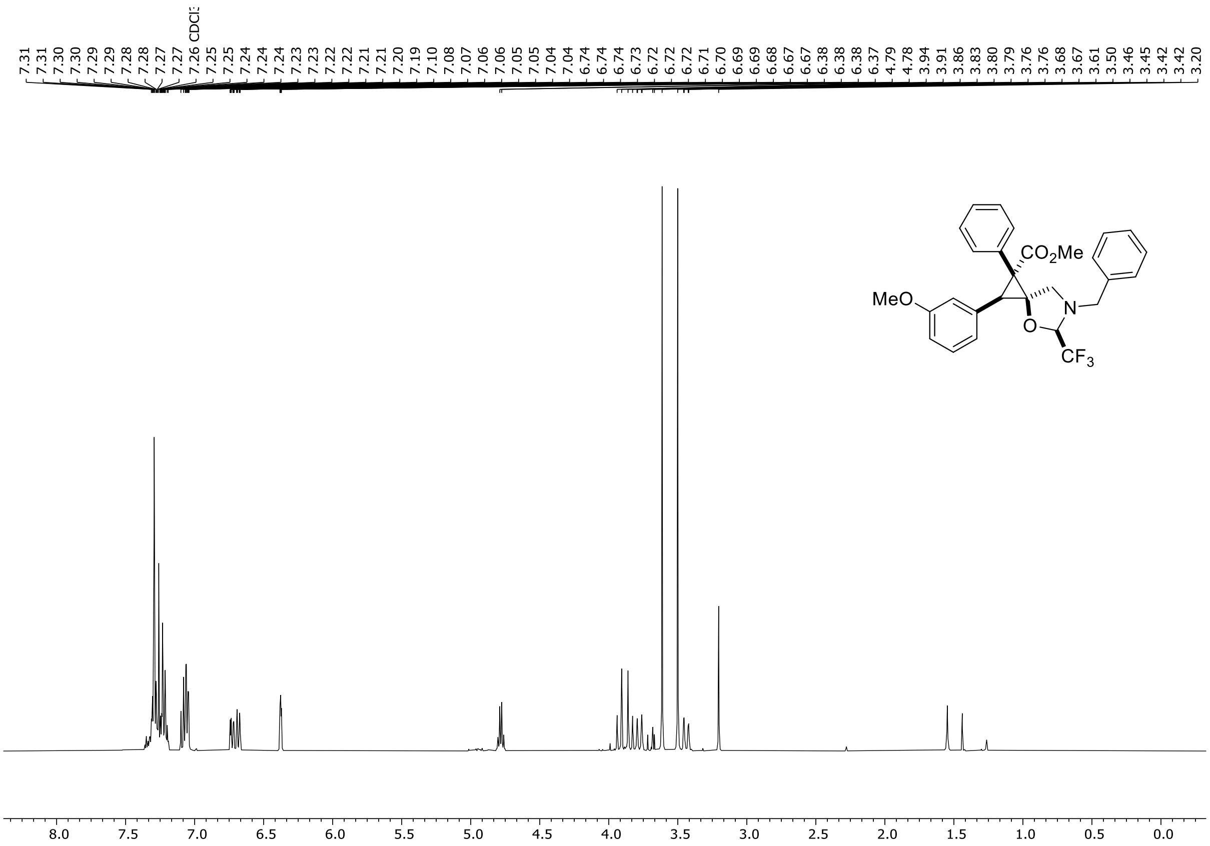

^{19}F NMR Spectrum of 6n (376 MHz, CDCl$_3$)

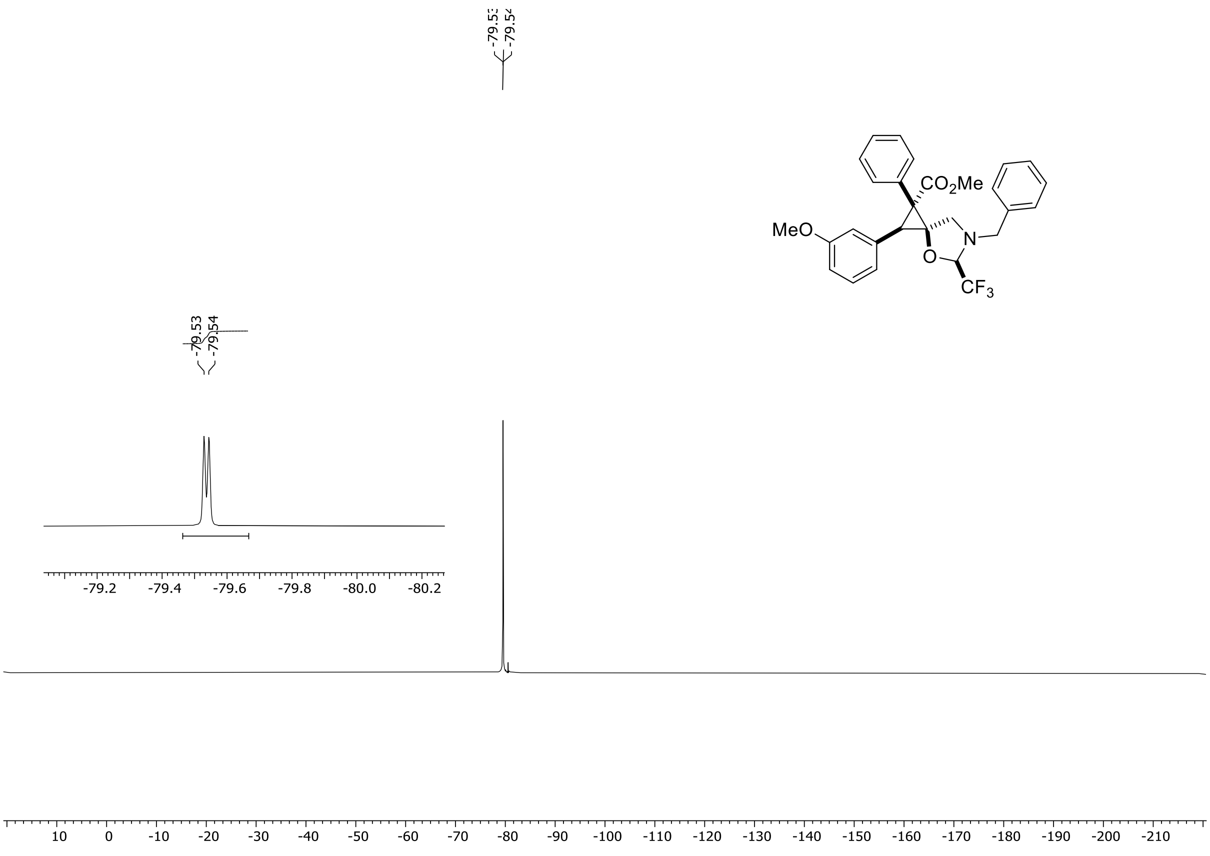

^{1}H NMR Spectrum of 6o (400 MHz, CDCl$_3$)

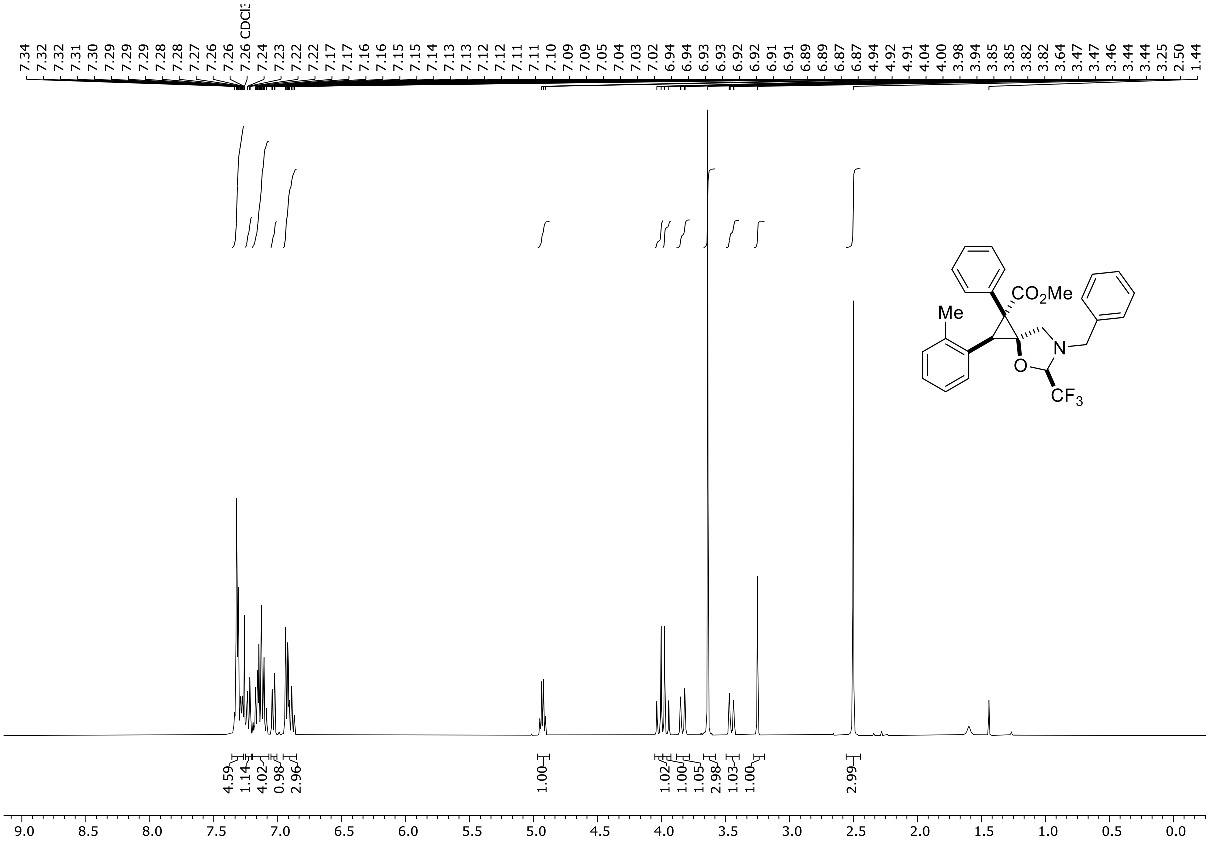

^{13}C NMR Spectrum of 6o (101 MHz, $CDCl_3$)

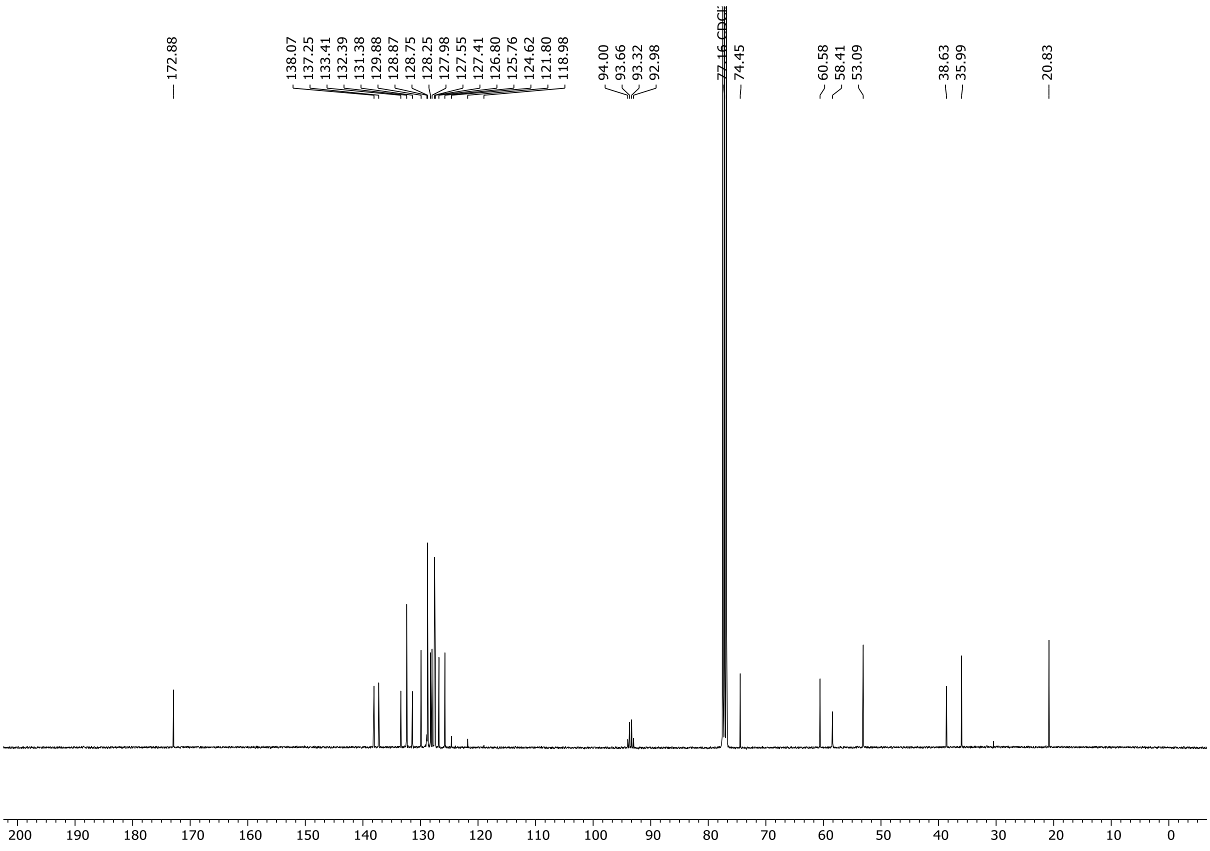

^{19}F NMR Spectrum of 6o (376 MHz, $CDCl_3$)

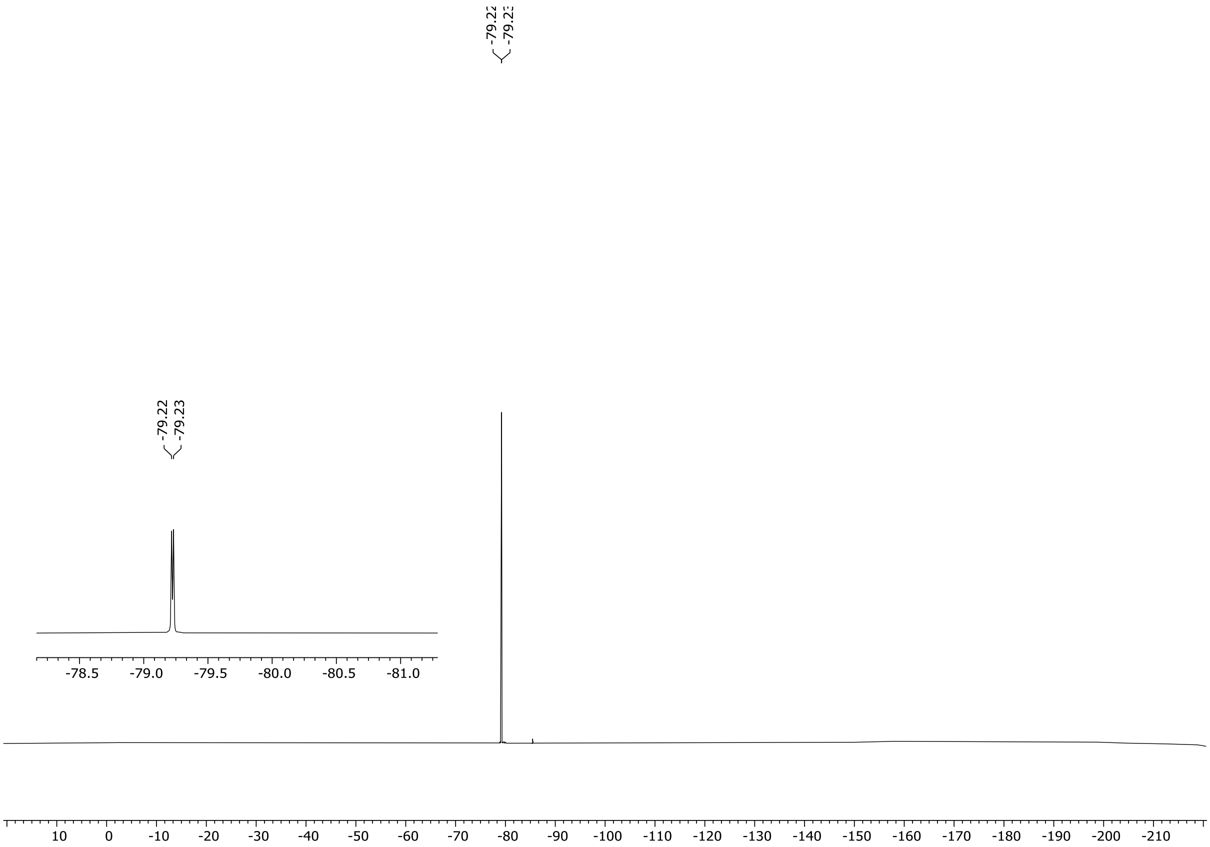

^{1}H NMR Spectrum of 6p (400 MHz, $CDCl_3$)

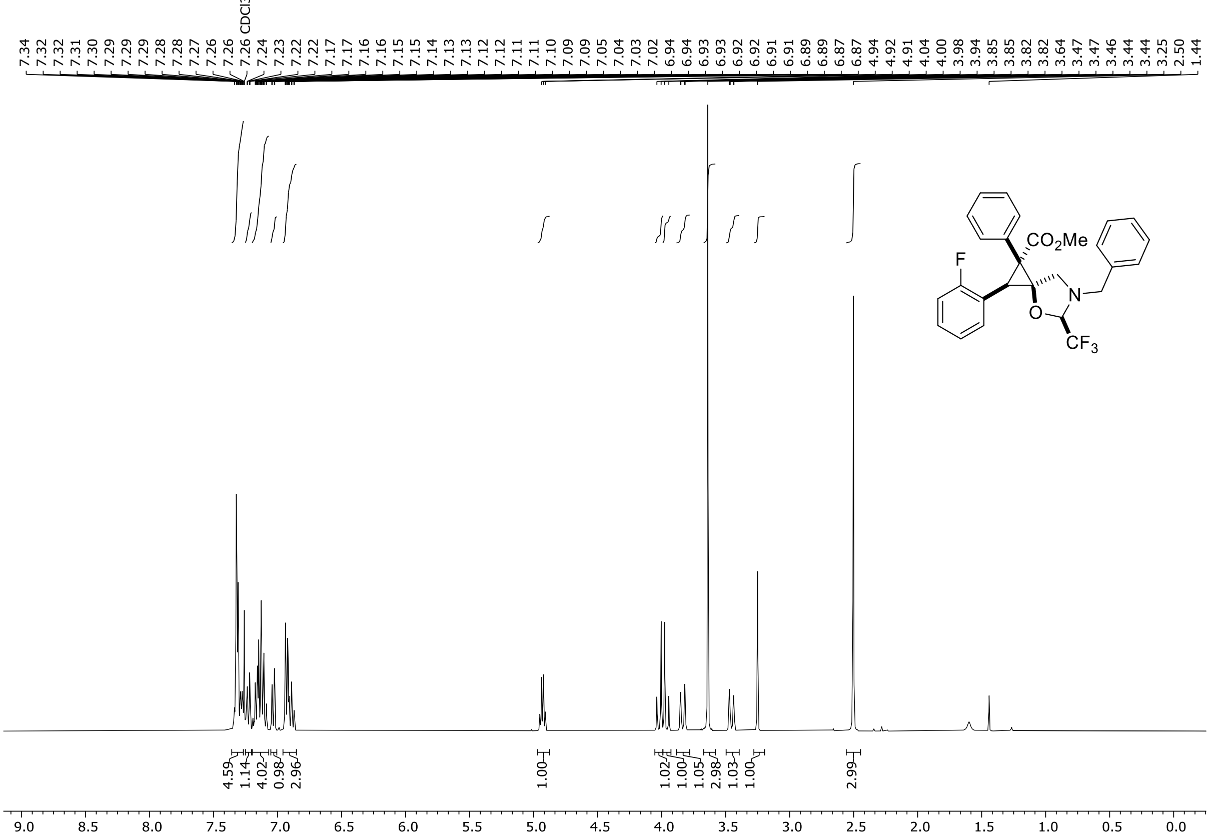

^{13}C NMR Spectrum of 6p (101 MHz, $CDCl_3$)

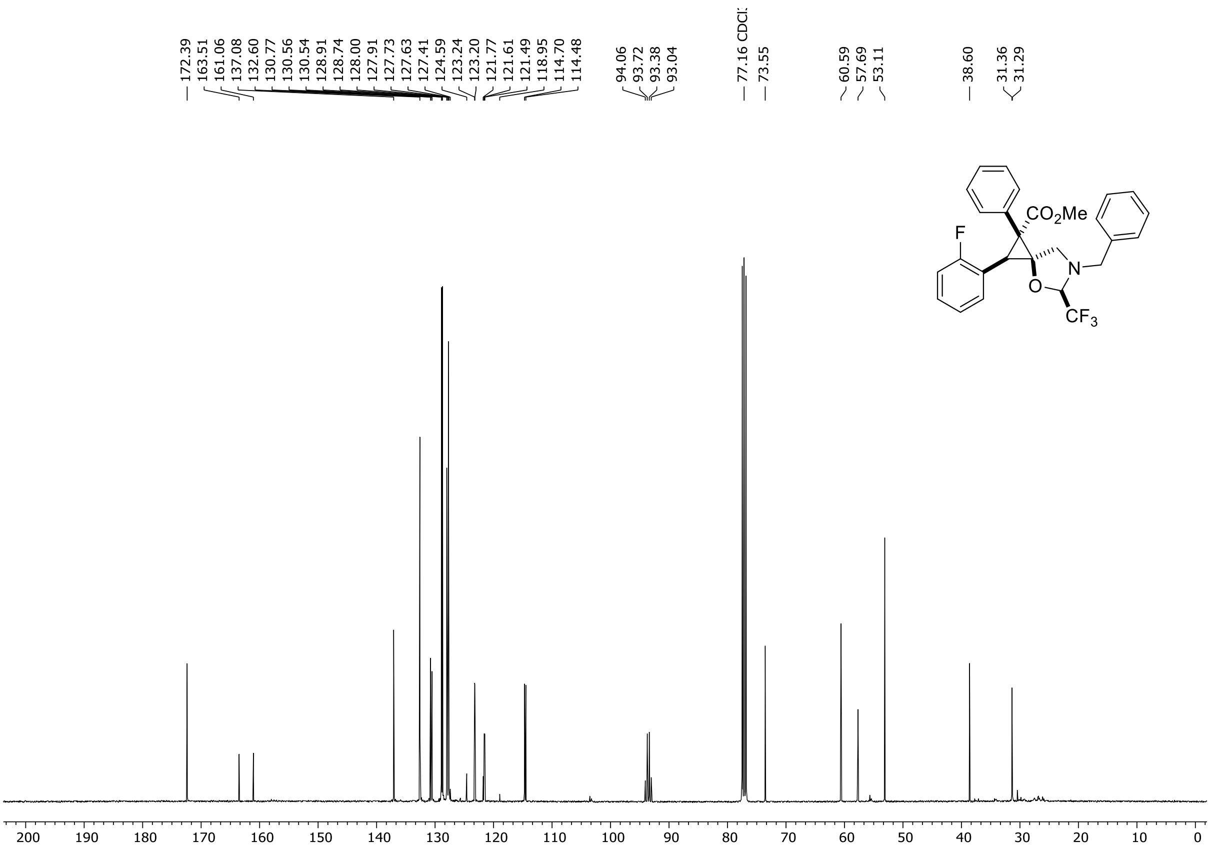

^{19}F NMR Spectrum of 6p (376 MHz, $CDCl_3$)

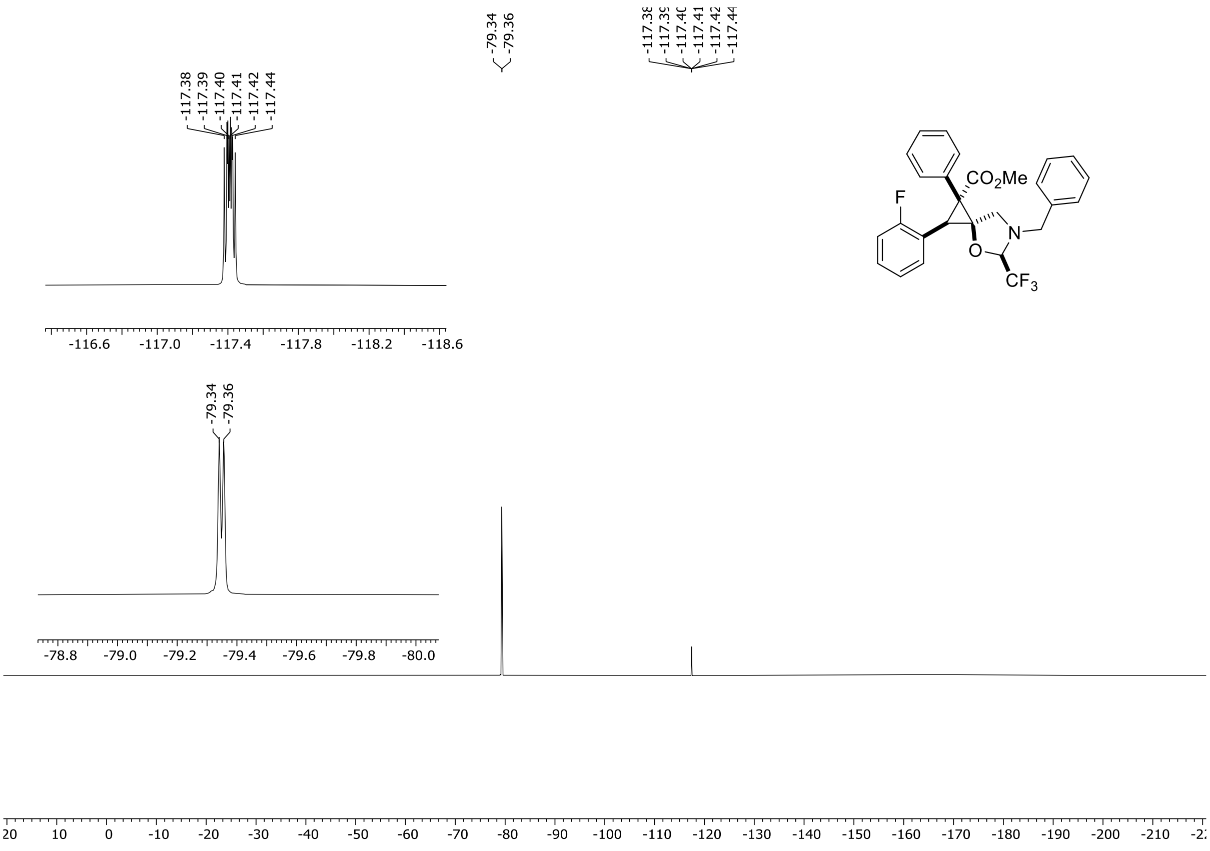

^{1}H NMR Spectrum of 6q (400 MHz, $CDCl_3$)

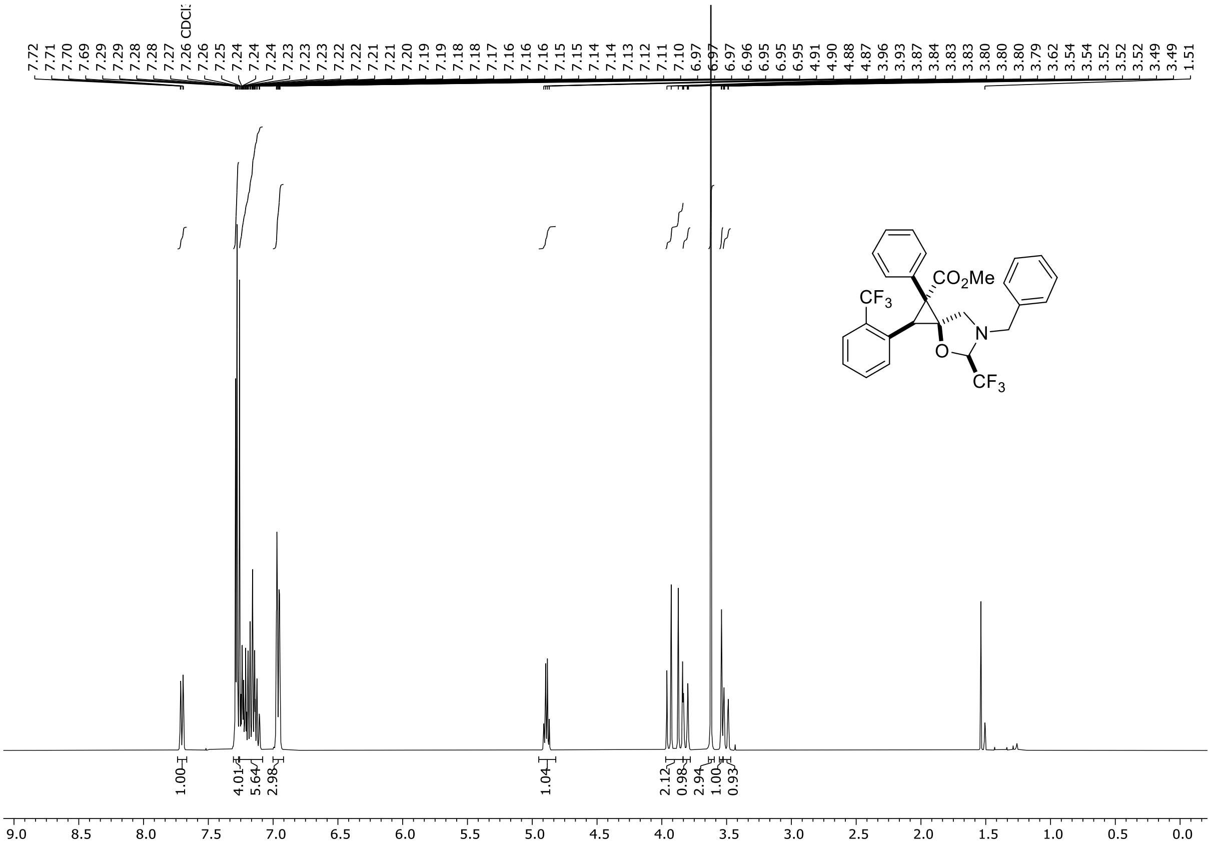

^{13}C NMR Spectrum of 6q (101 MHz, $CDCl_3$)

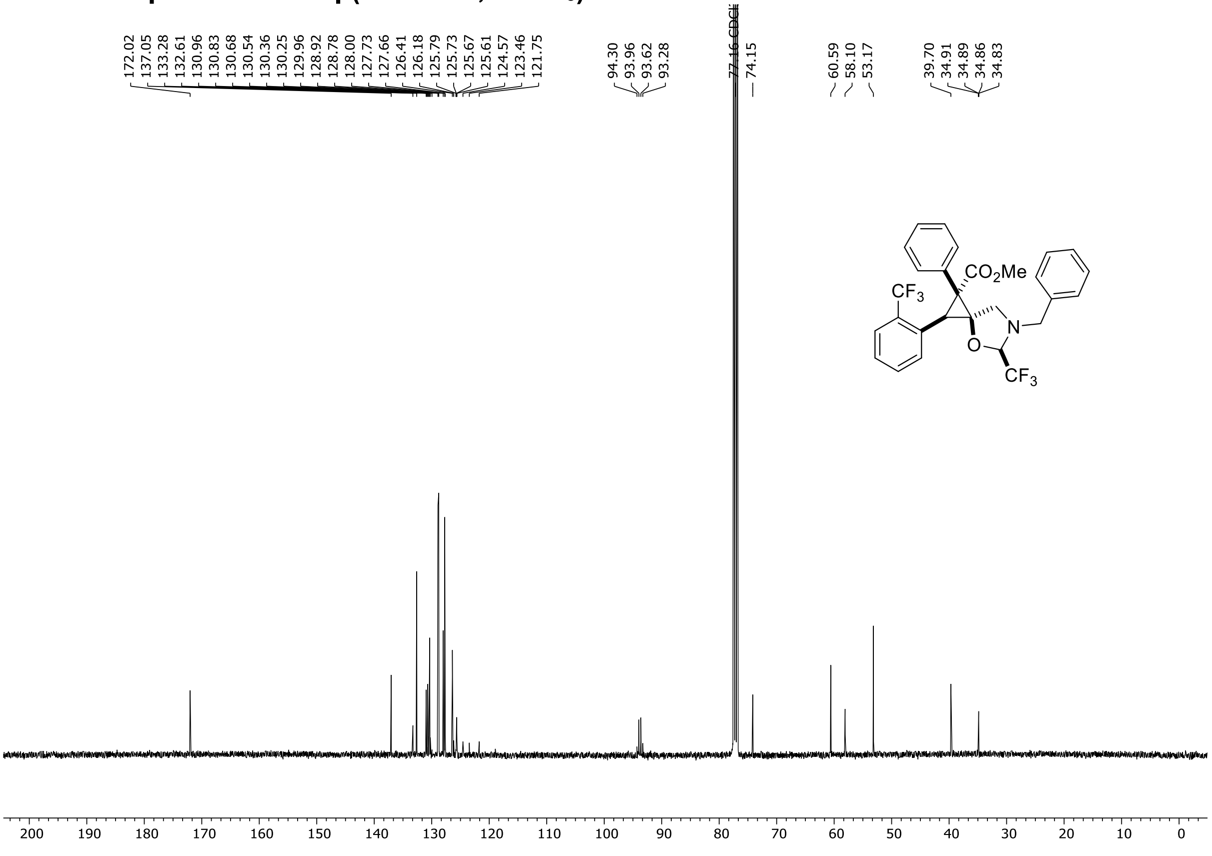

^{19}F NMR Spectrum of 6q (376 MHz, CDCl$_3$)

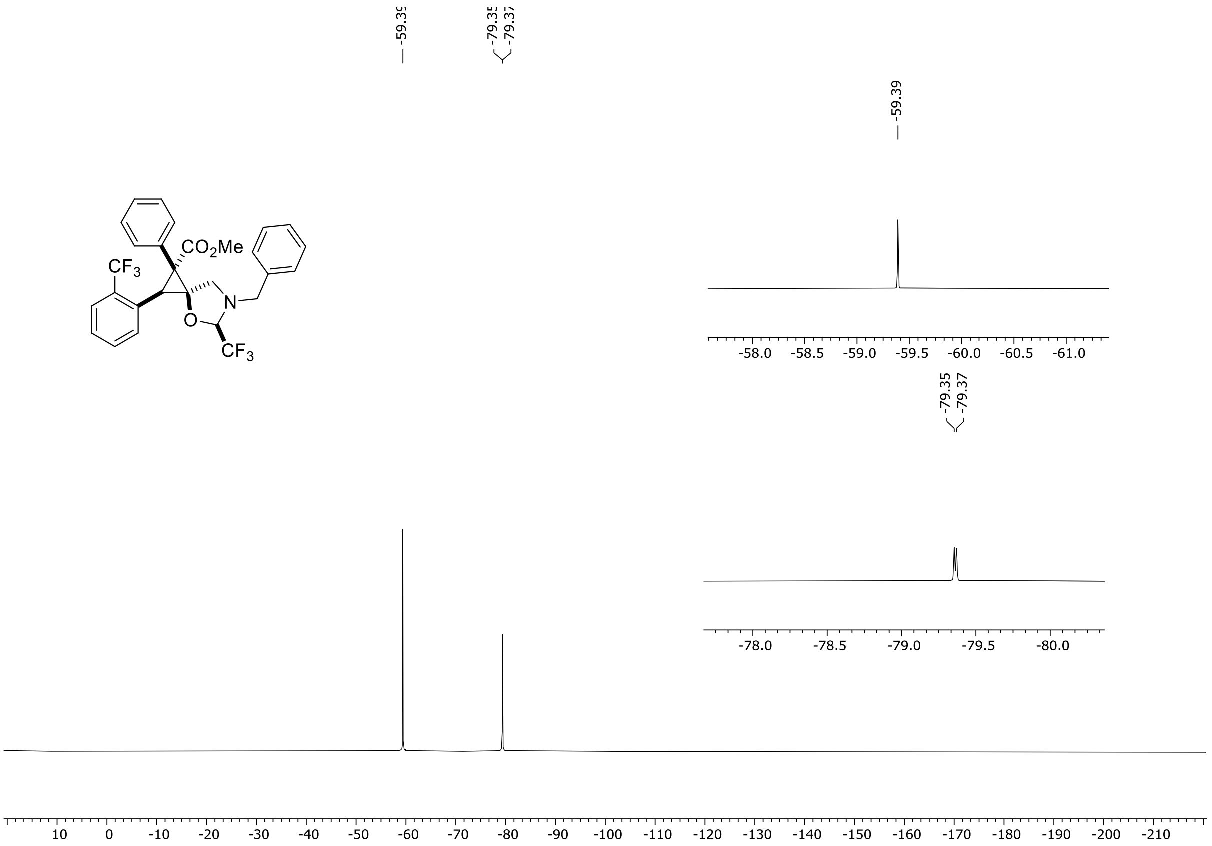

^{1}H NMR Spectrum of 6r (400 MHz, CDCl$_3$)

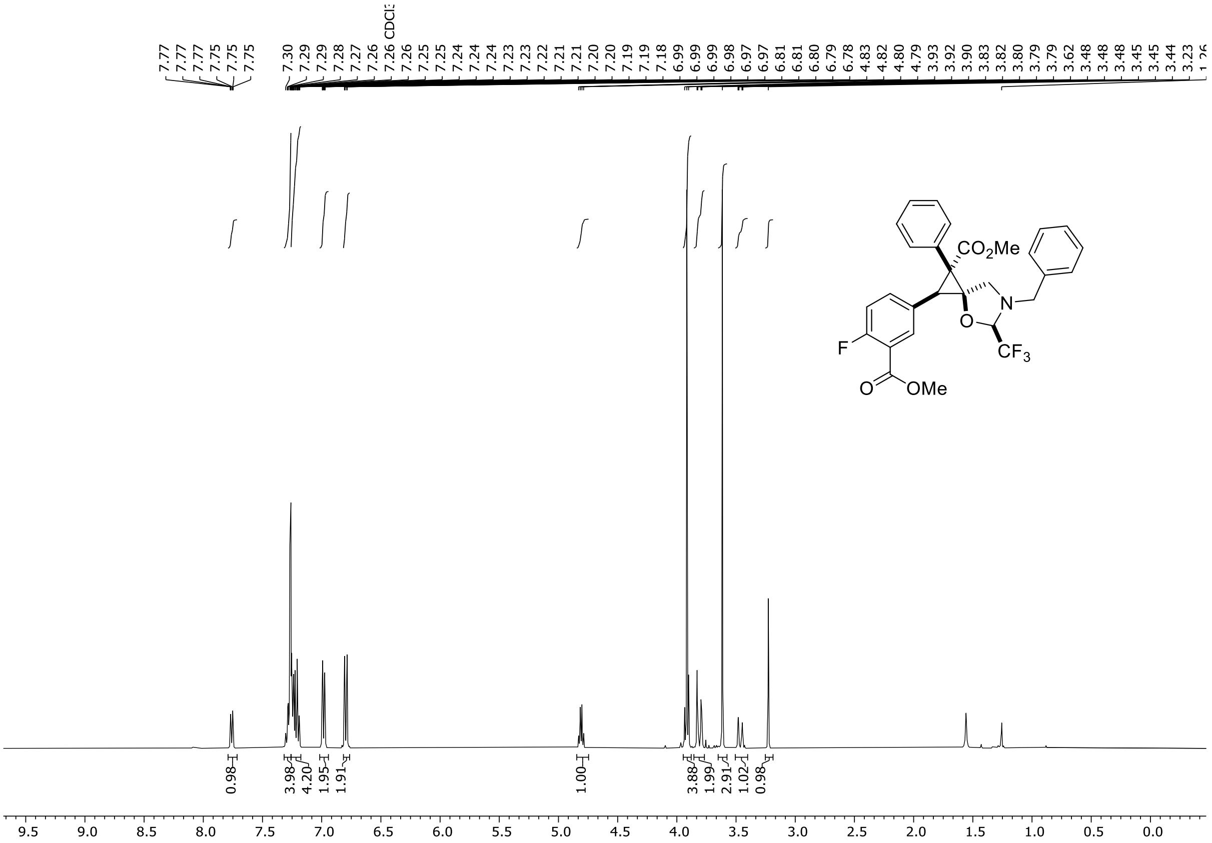

^{13}C NMR Spectrum of 6r (101 MHz, CDCl$_3$)

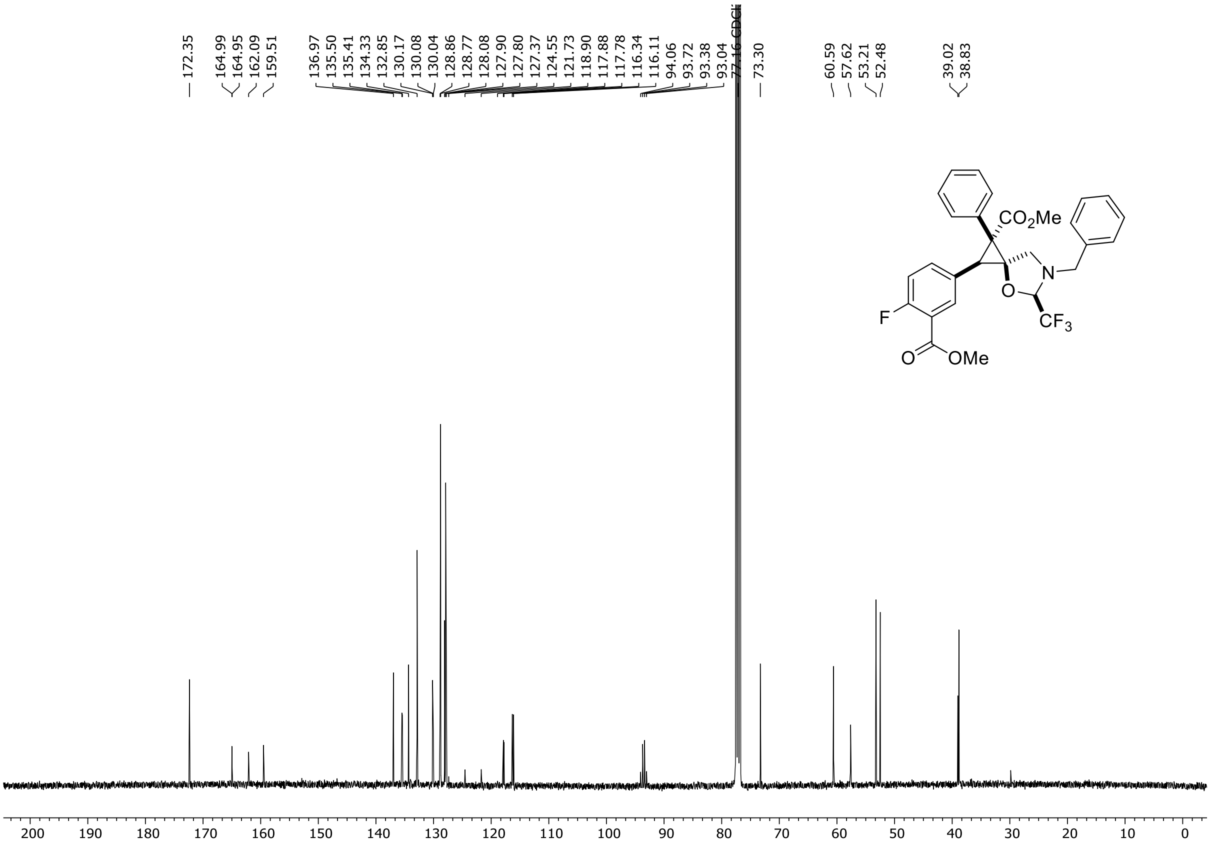

^{19}F NMR Spectrum of 6r (376 MHz, $CDCl_3$)

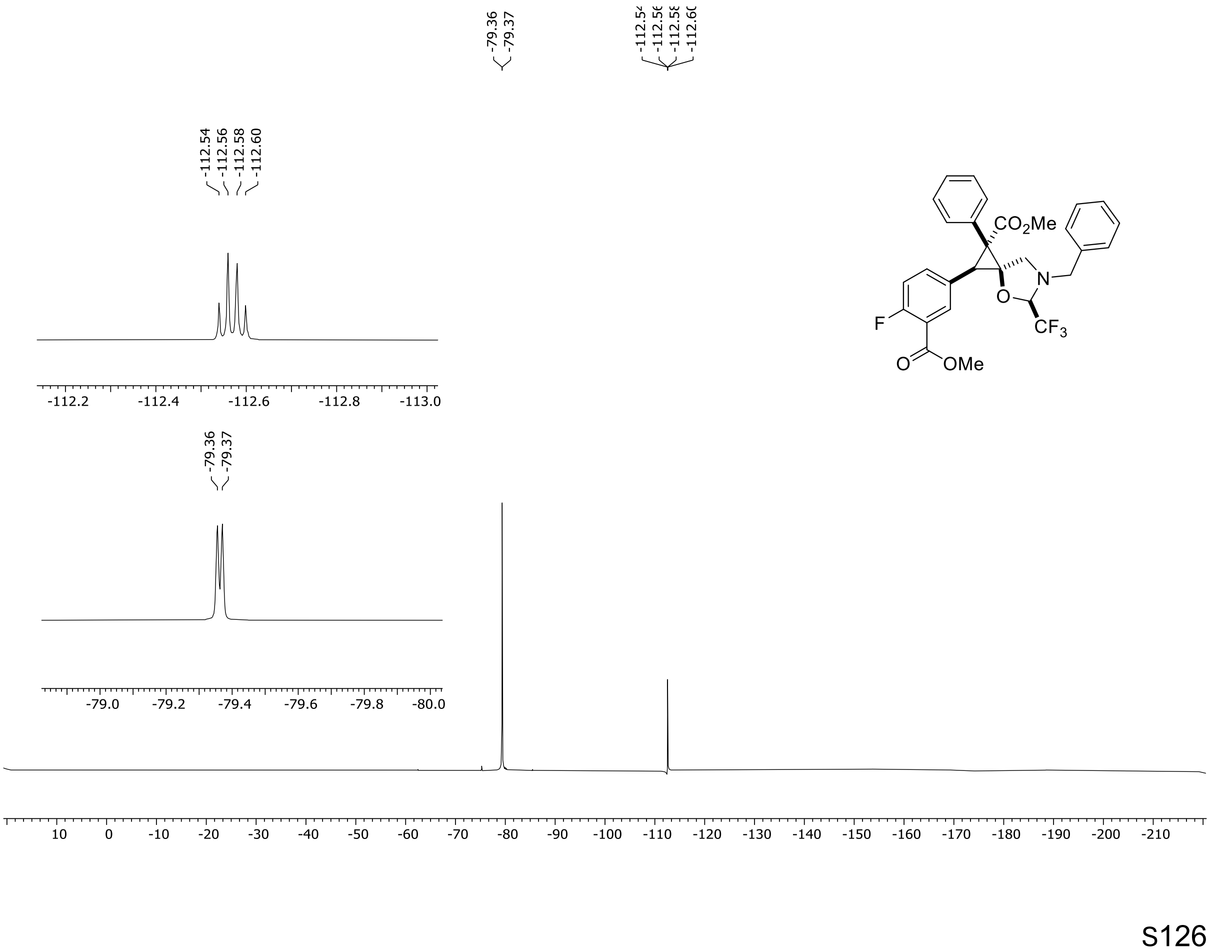

^{1}H NMR Spectrum of 6s (400 MHz, $CDCl_3$)

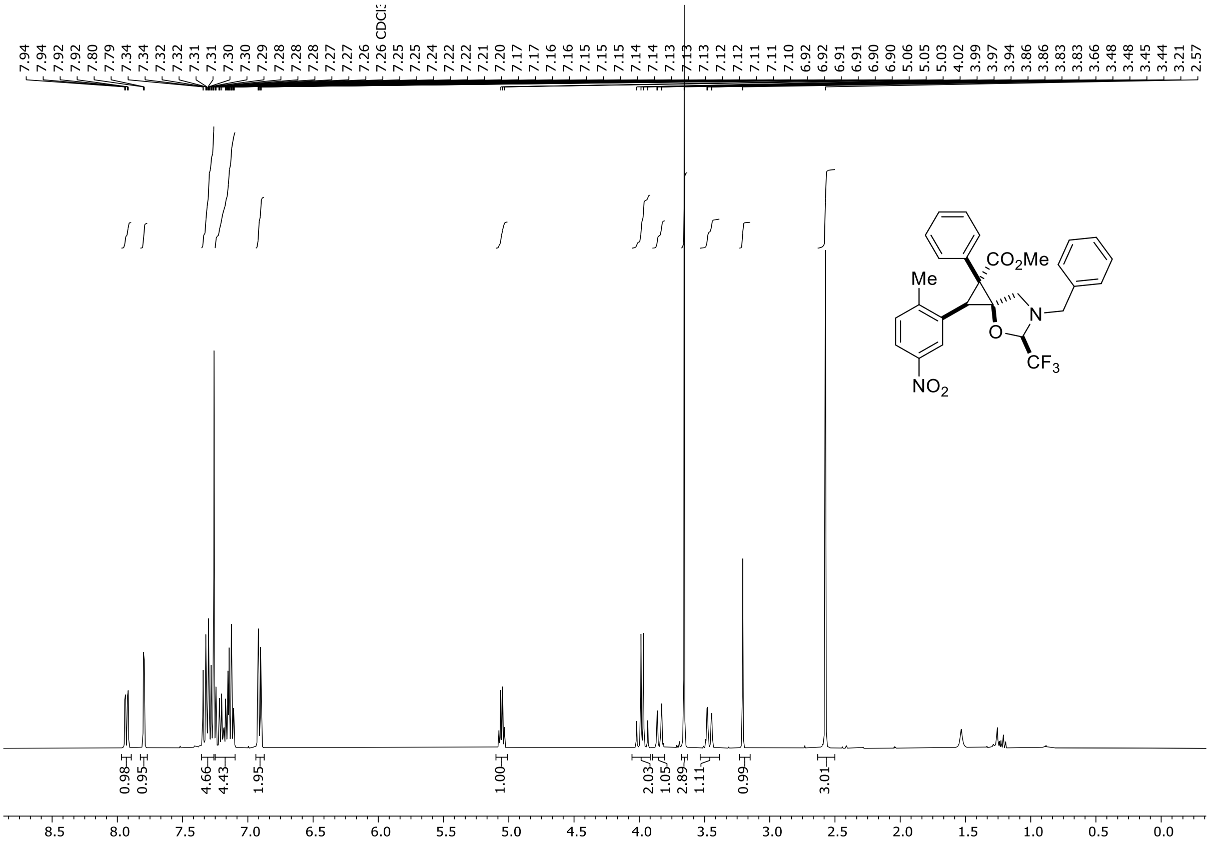

^{13}C NMR Spectrum of 6s (101 MHz, $CDCl_3$)

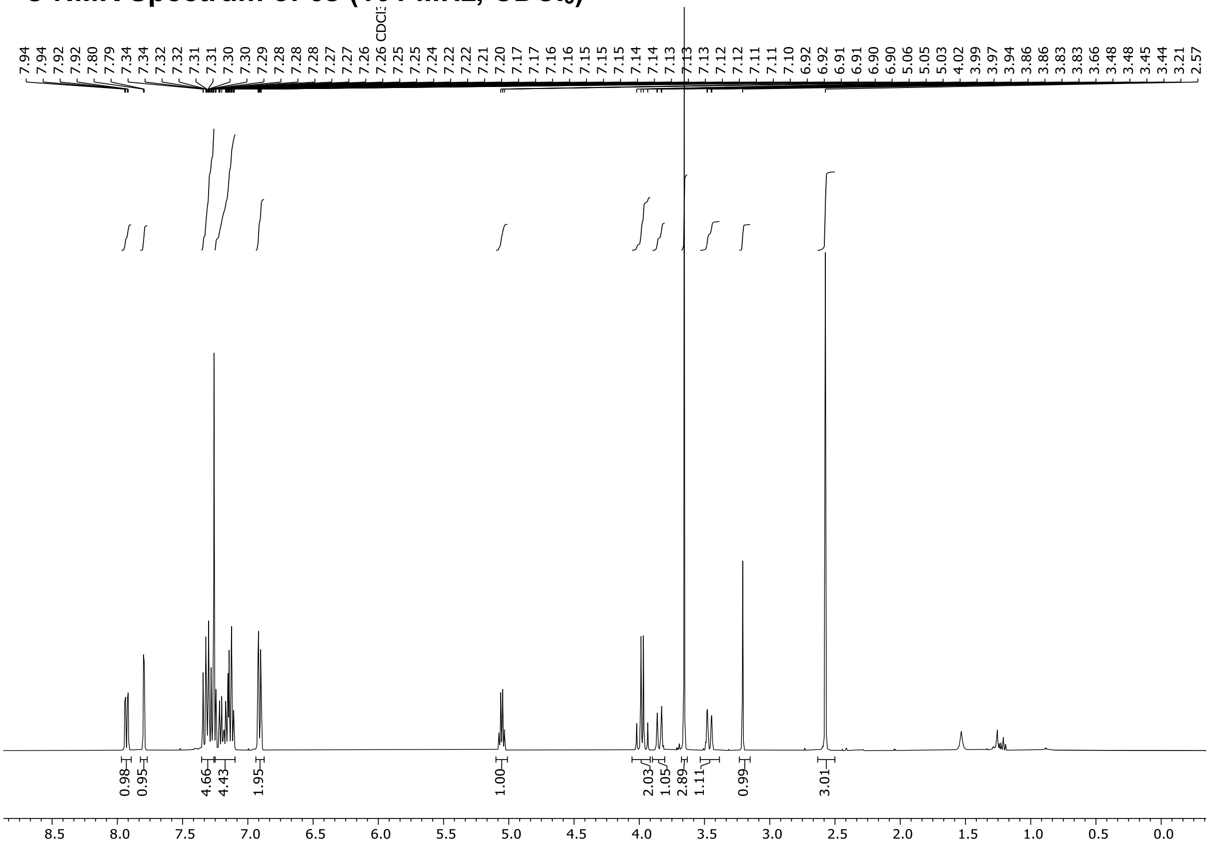

^{19}F NMR Spectrum of 6s (376 MHz, $CDCl_3$)

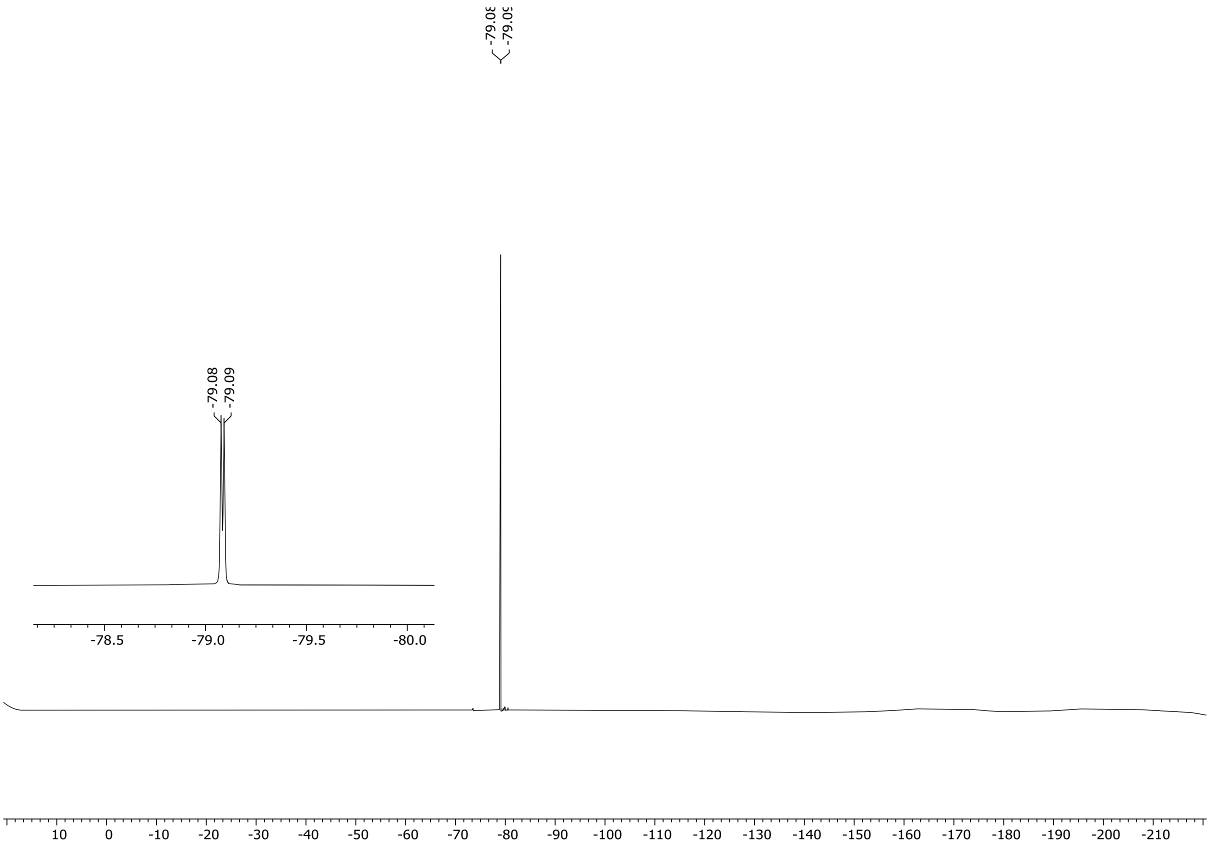

^{1}H NMR Spectrum of 500 6t (400 MHz, $CDCl_3$)

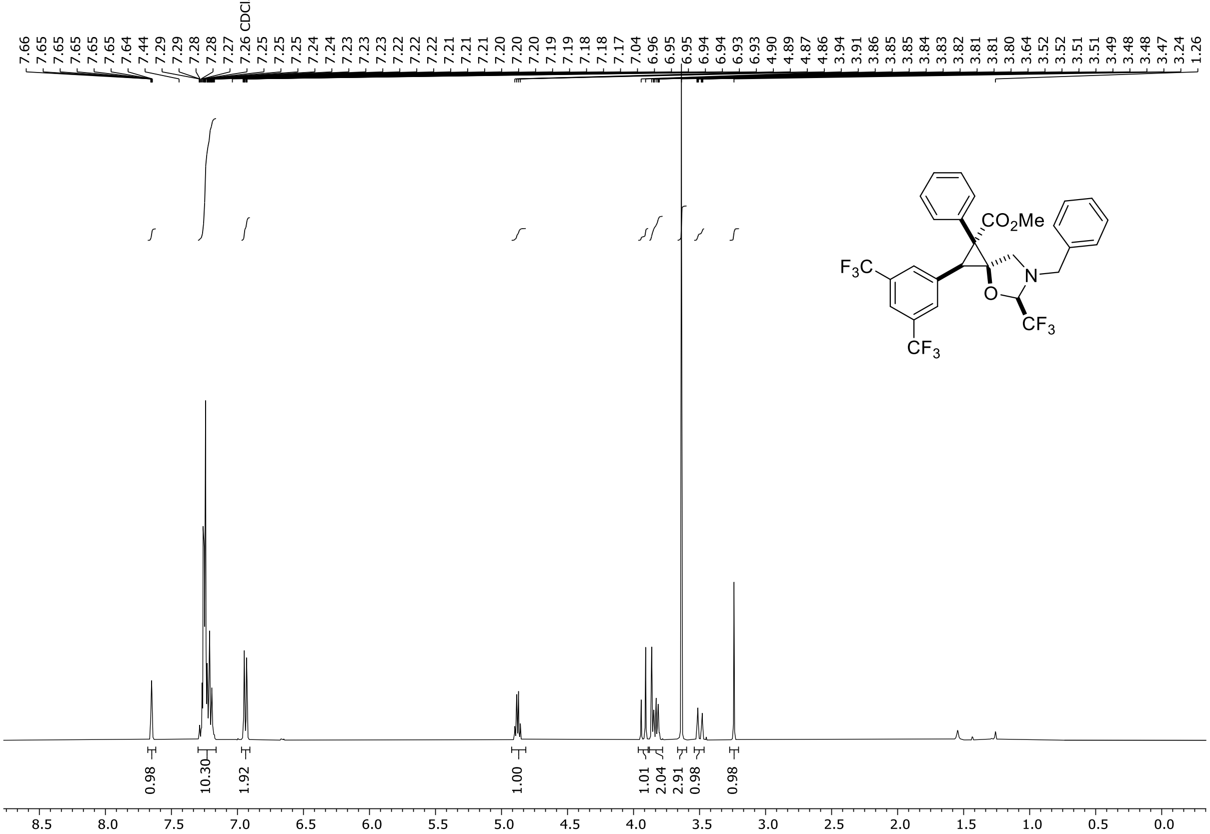

^{13}C NMR Spectrum of 6t (101 MHz, $CDCl_3$)

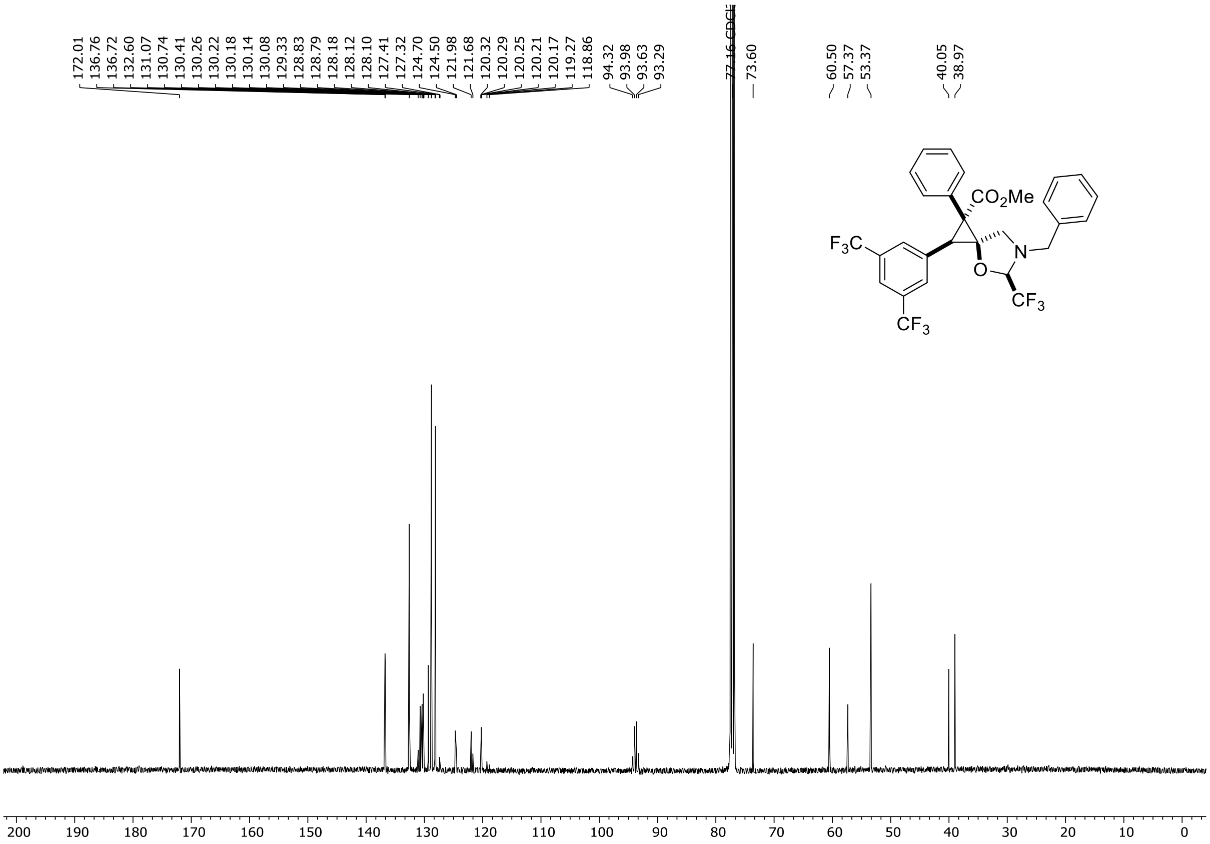

^{19}F NMR Spectrum of 6t (376 MHz, $CDCl_3$)

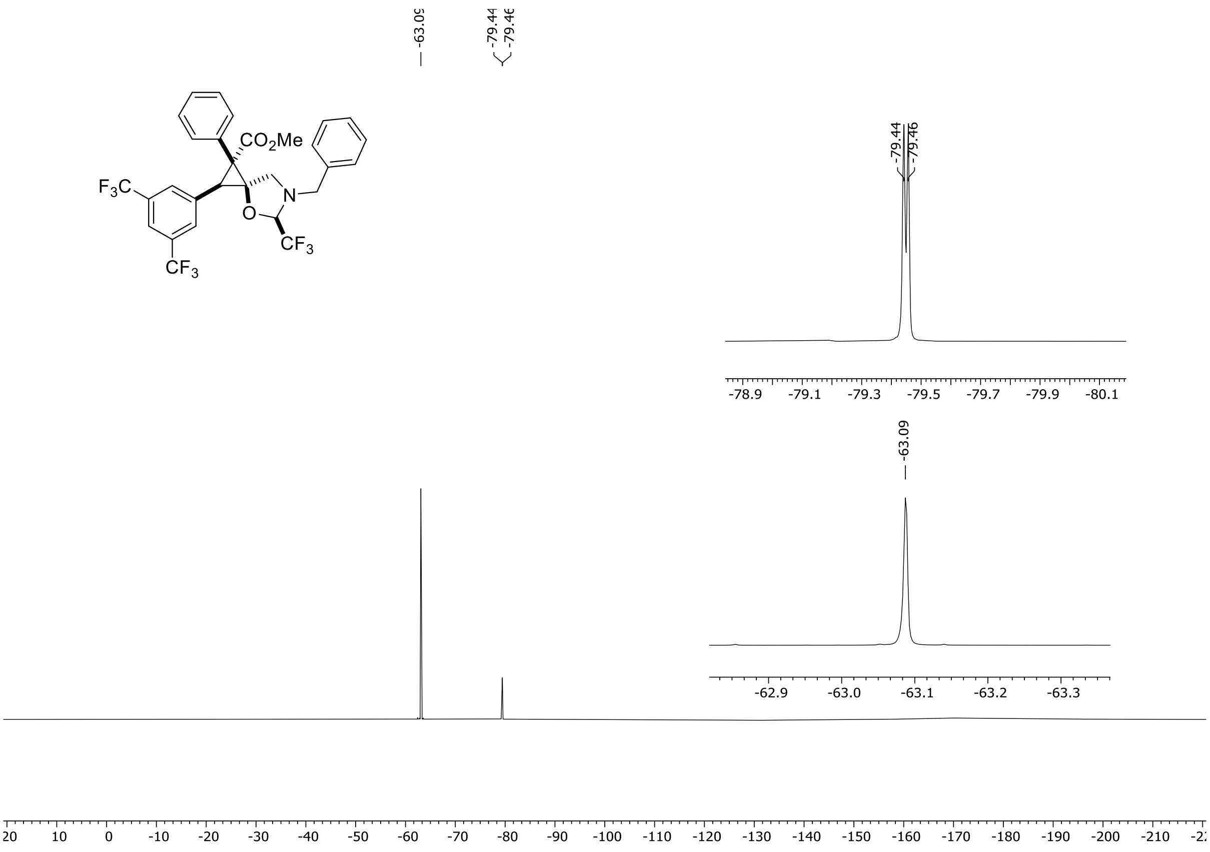

^{1}H NMR Spectrum of 453 6u (400 MHz, $CDCl_3$)

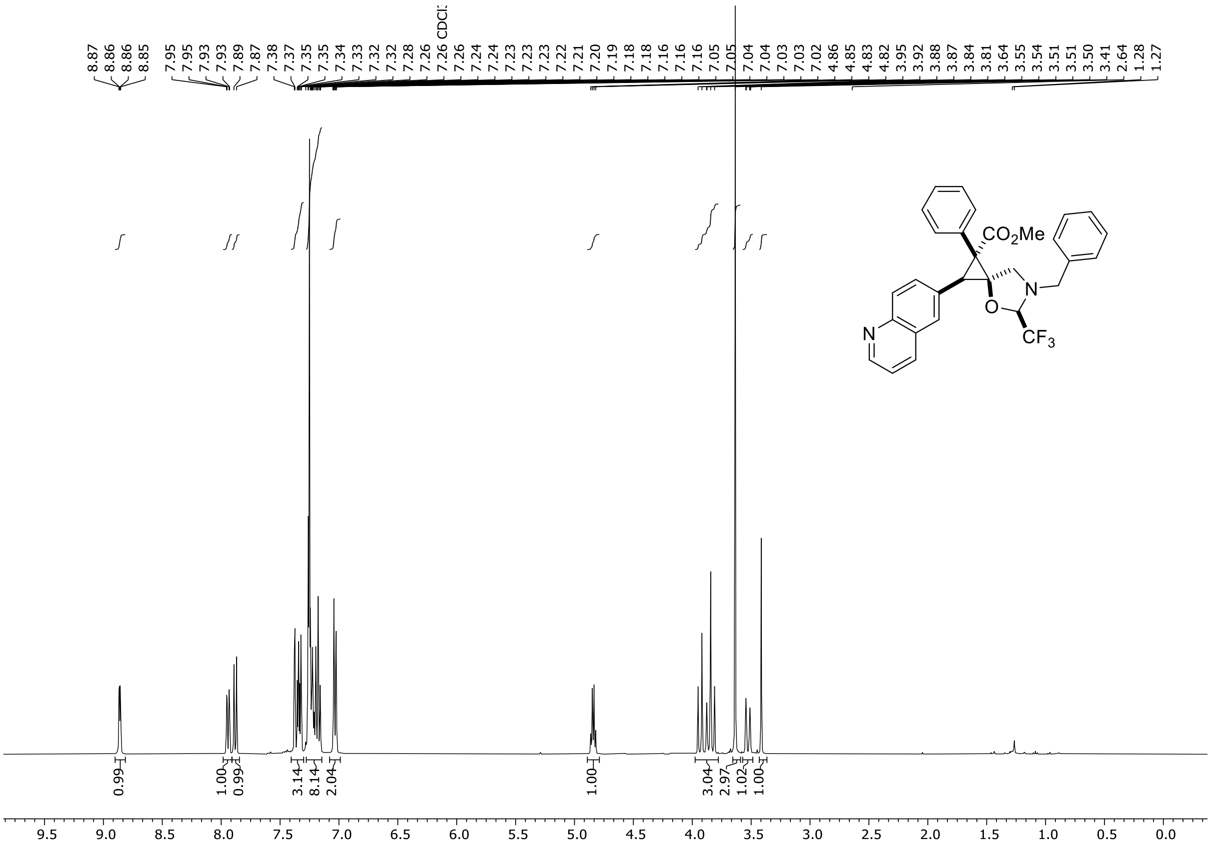

^{13}C NMR Spectrum of 6u (101 MHz, $CDCl_3$)

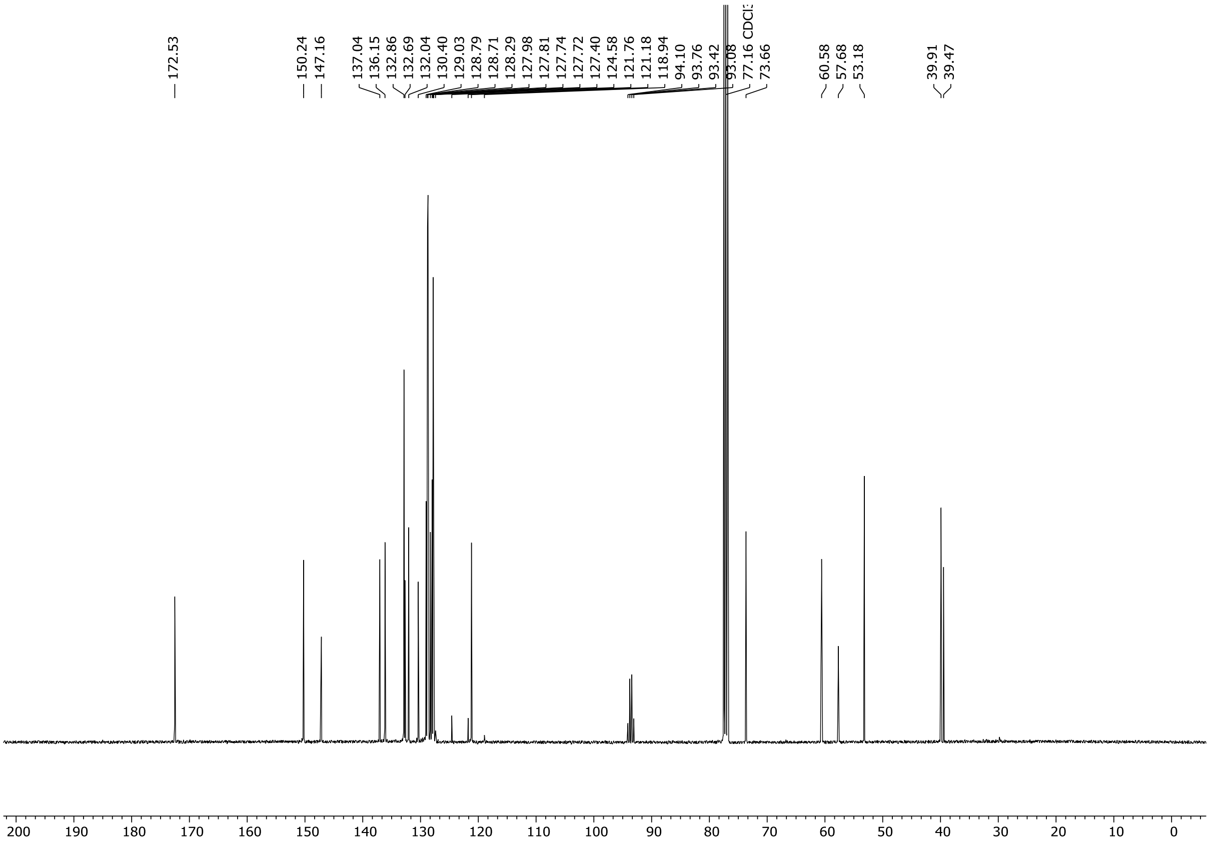

^{19}F NMR Spectrum of 6u (376 MHz, $CDCl_3$)

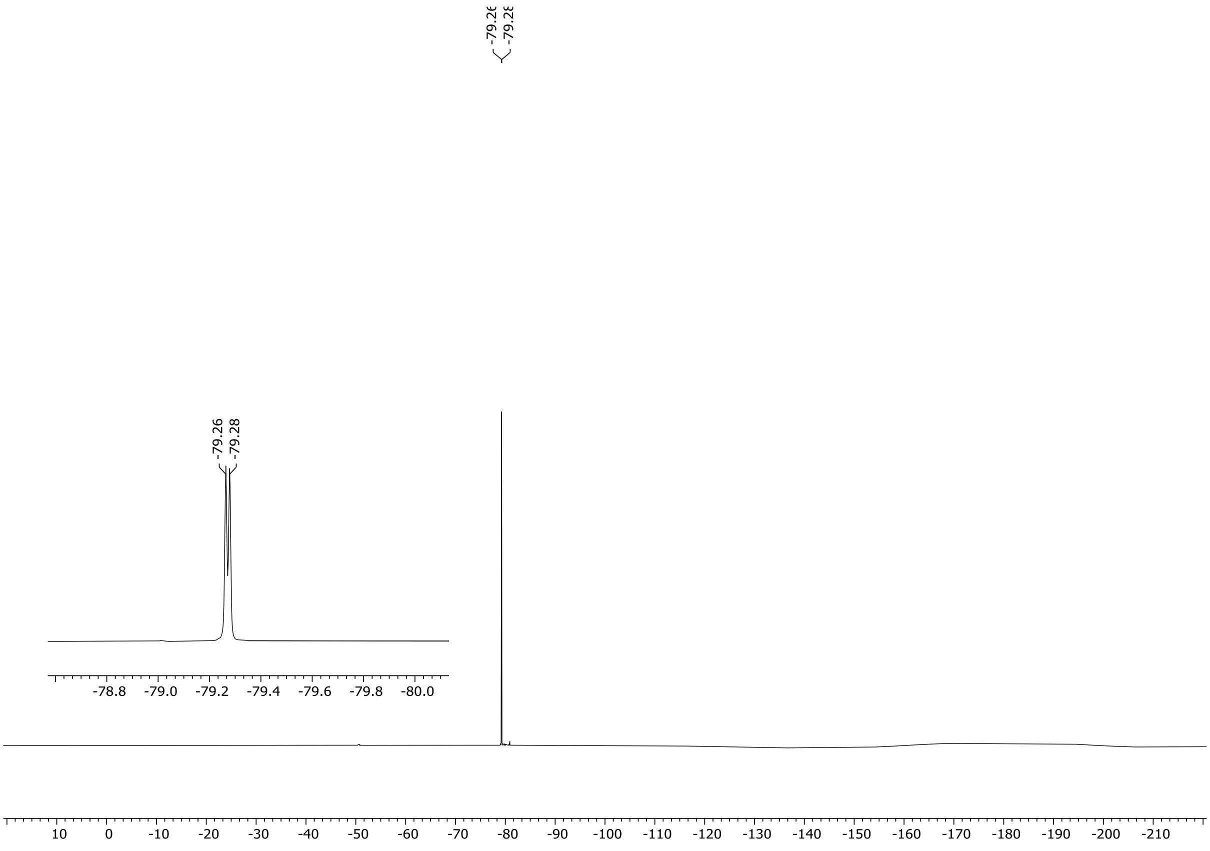

^{1}H NMR Spectrum of 6v (400 MHz, $CDCl_3$)

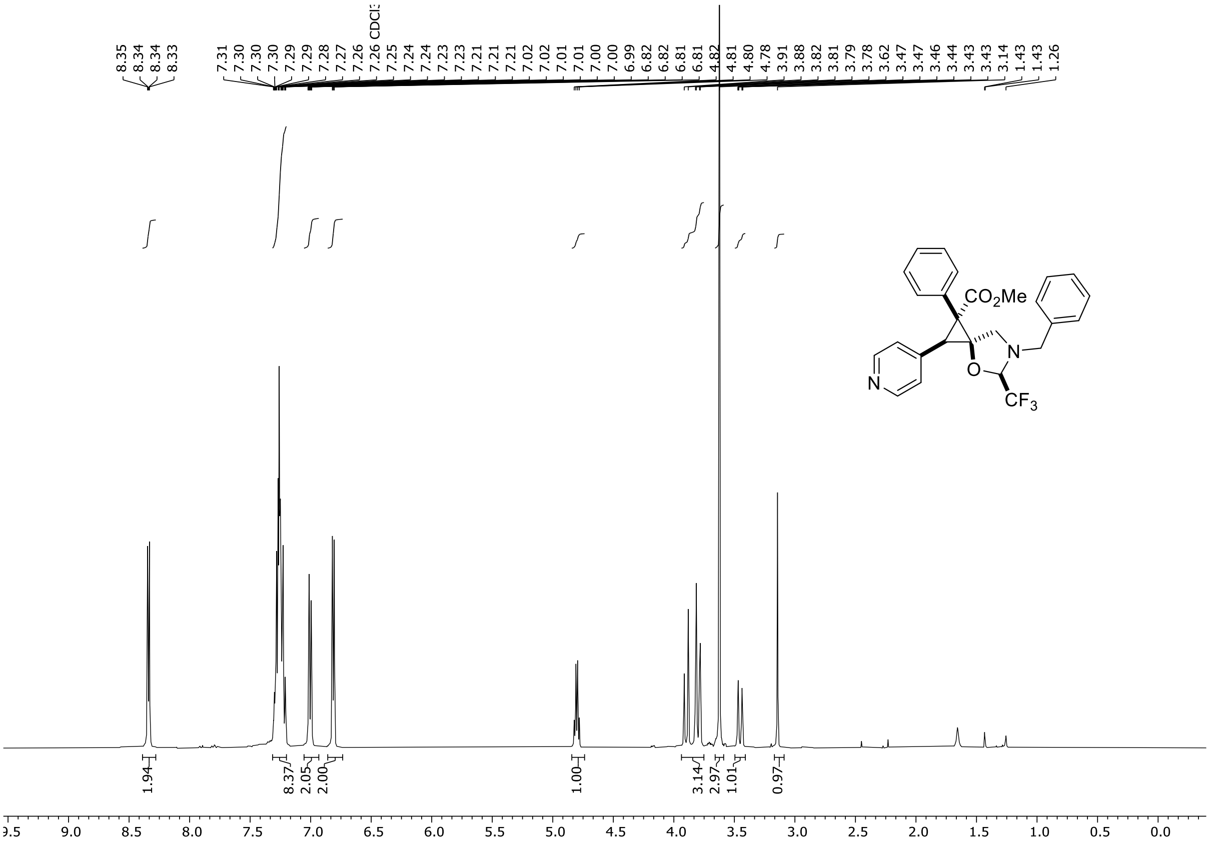

^{13}C NMR Spectrum of 6v (101 MHz, $CDCl_3$)

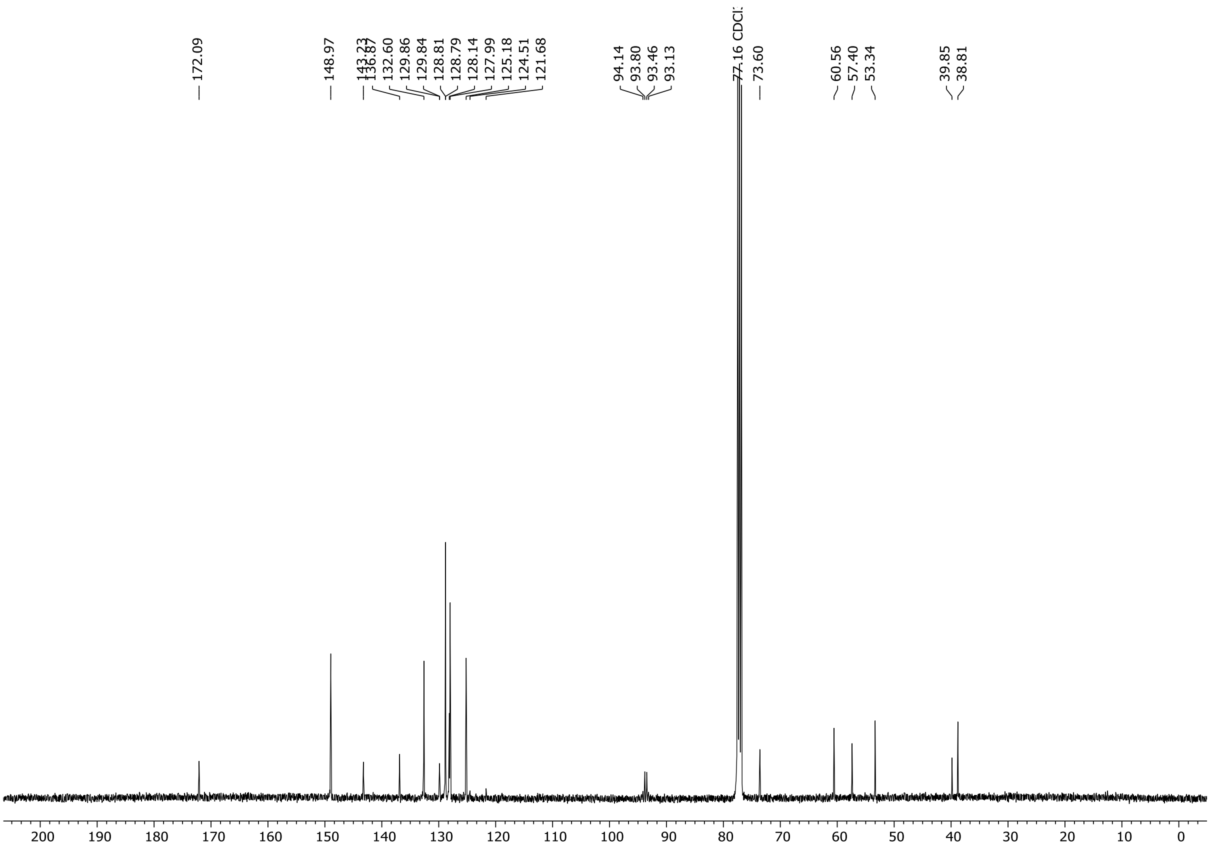

^{19}F NMR Spectrum of 6v (376 MHz, $CDCl_3$)

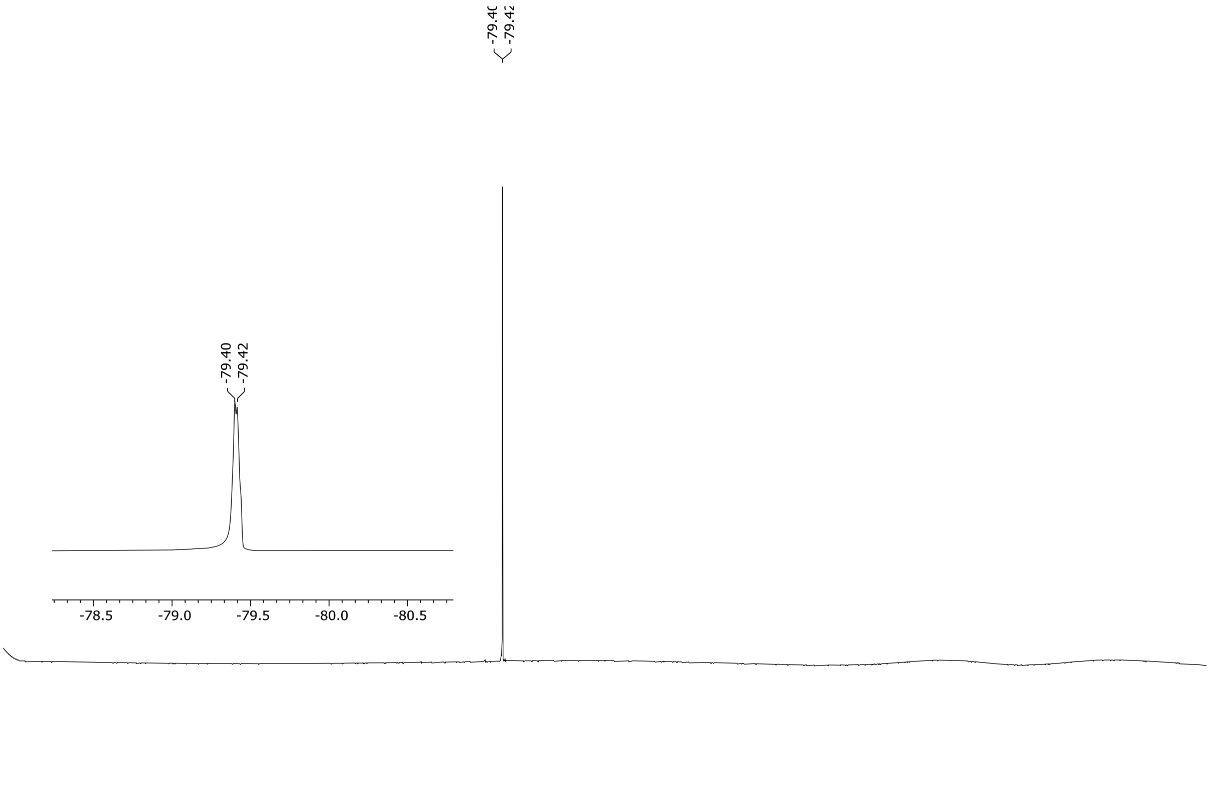

^{1}H NMR Spectrum of 6w (400 MHz, $CDCl_3$)

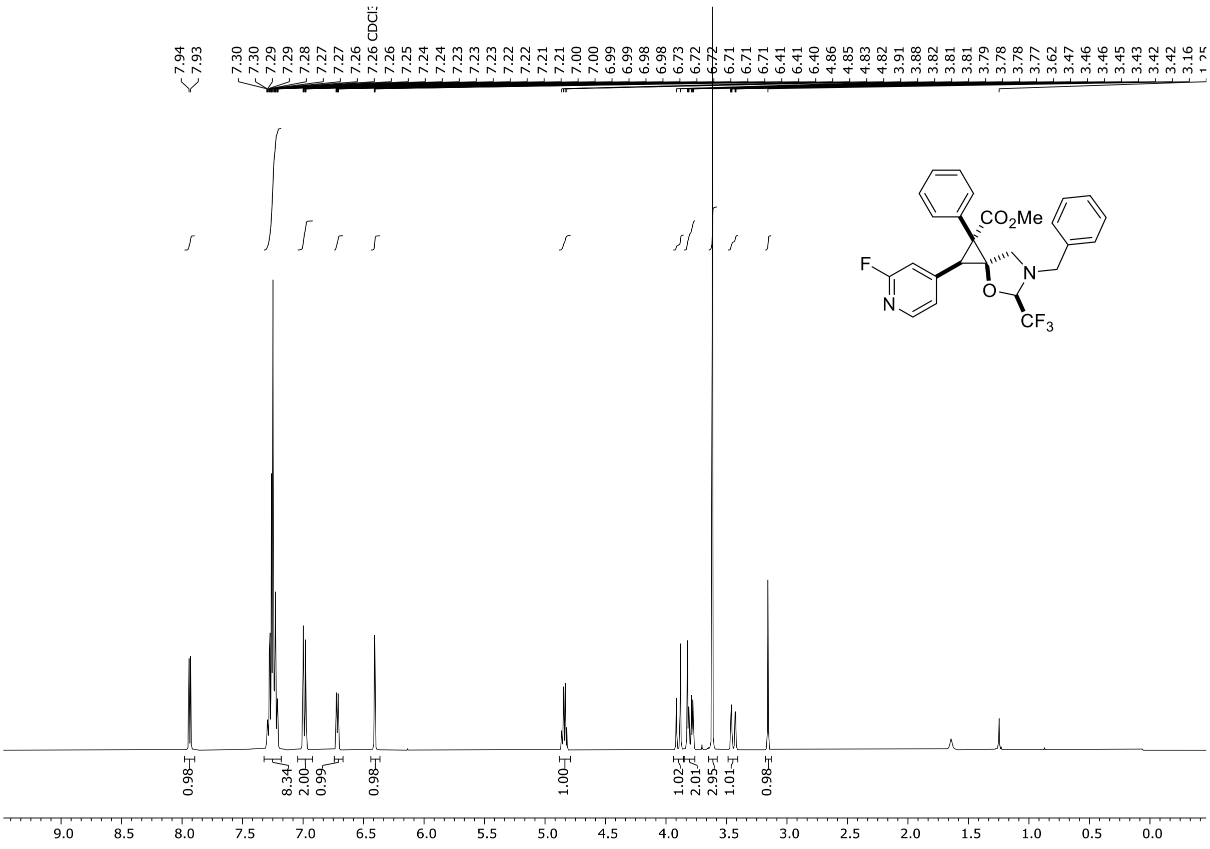

^{13}C NMR Spectrum of 6w (101 MHz, $CDCl_3$)

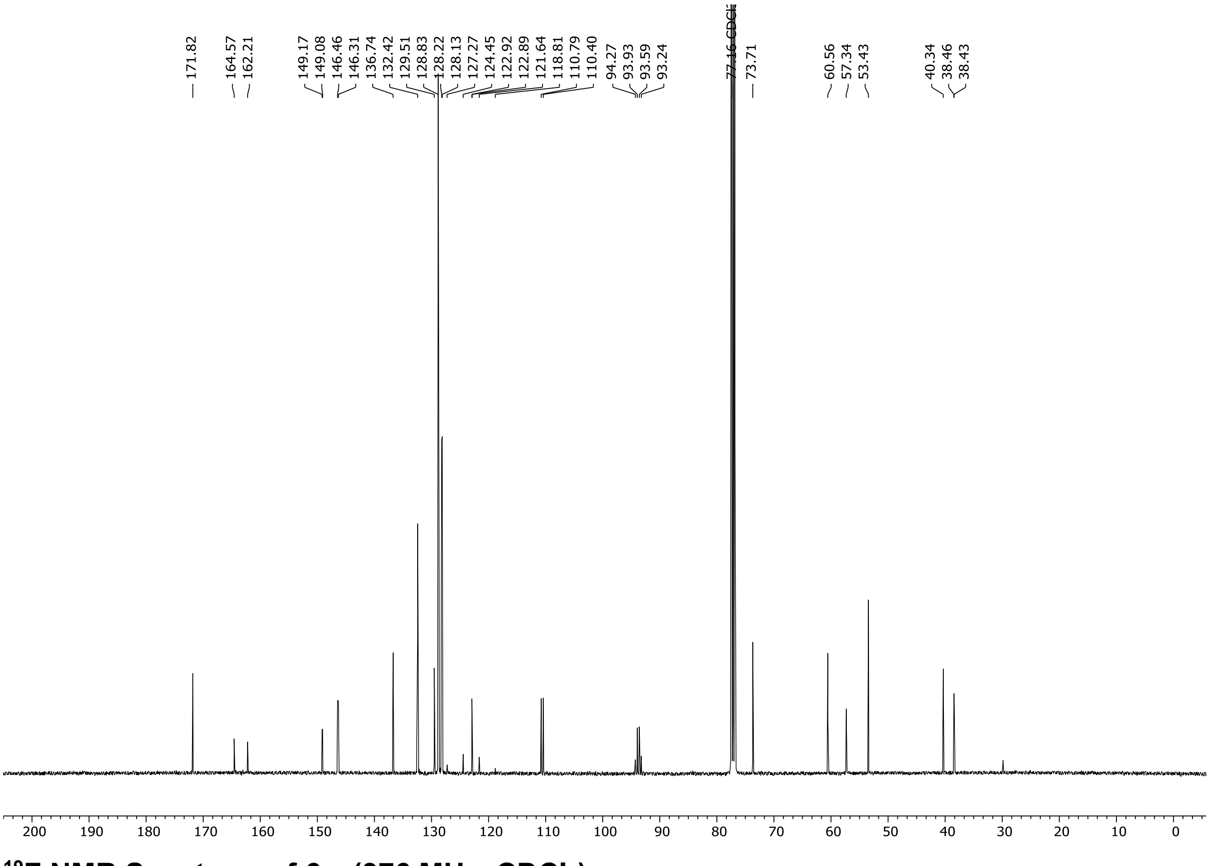

^{19}F NMR Spectrum of 6w (376 MHz, $CDCl_3$)

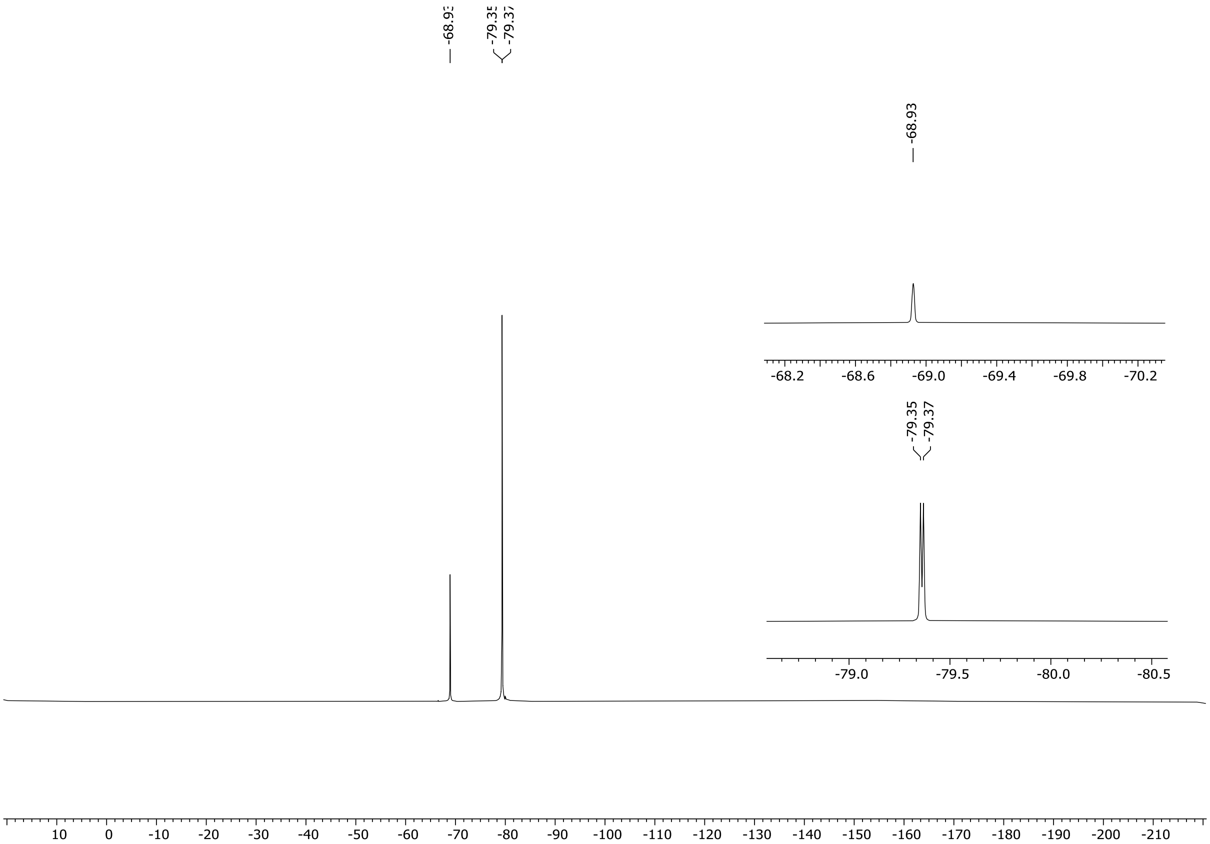

^{1}H NMR Spectrum of 6x (400 MHz, CDCl$_3$)

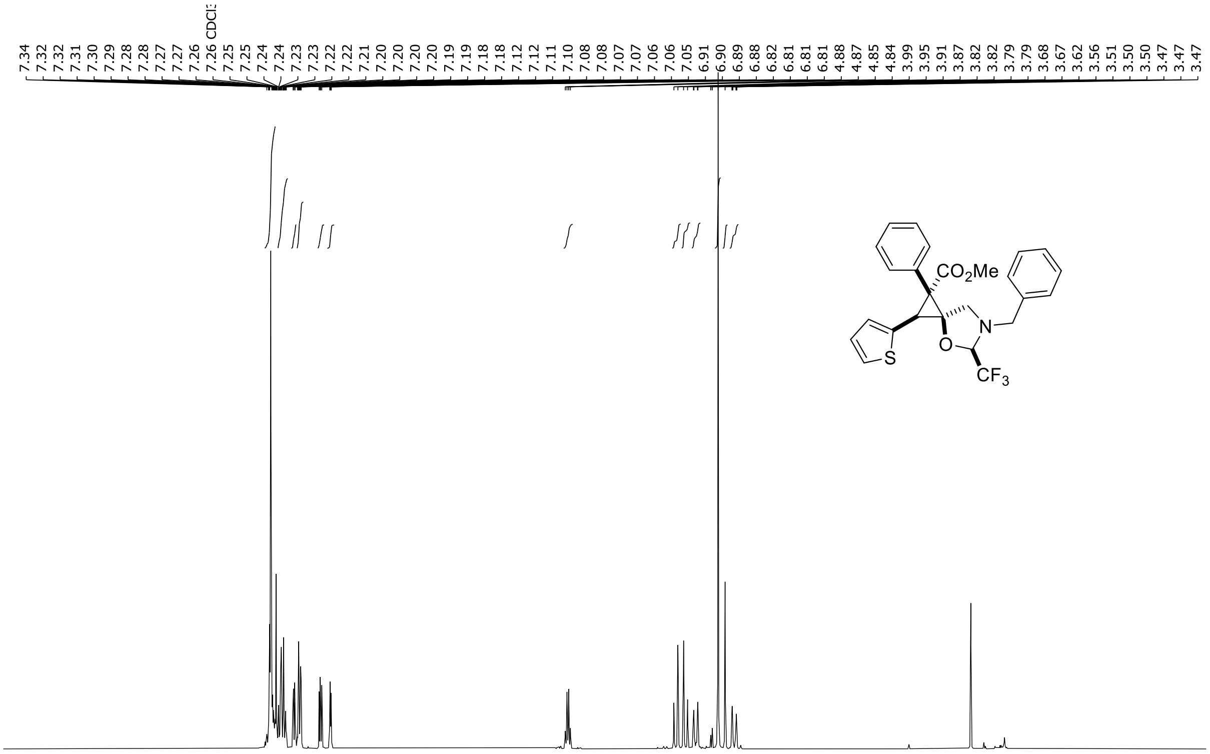

^{13}C NMR Spectrum of 6x (101 MHz, CDCl$_3$)

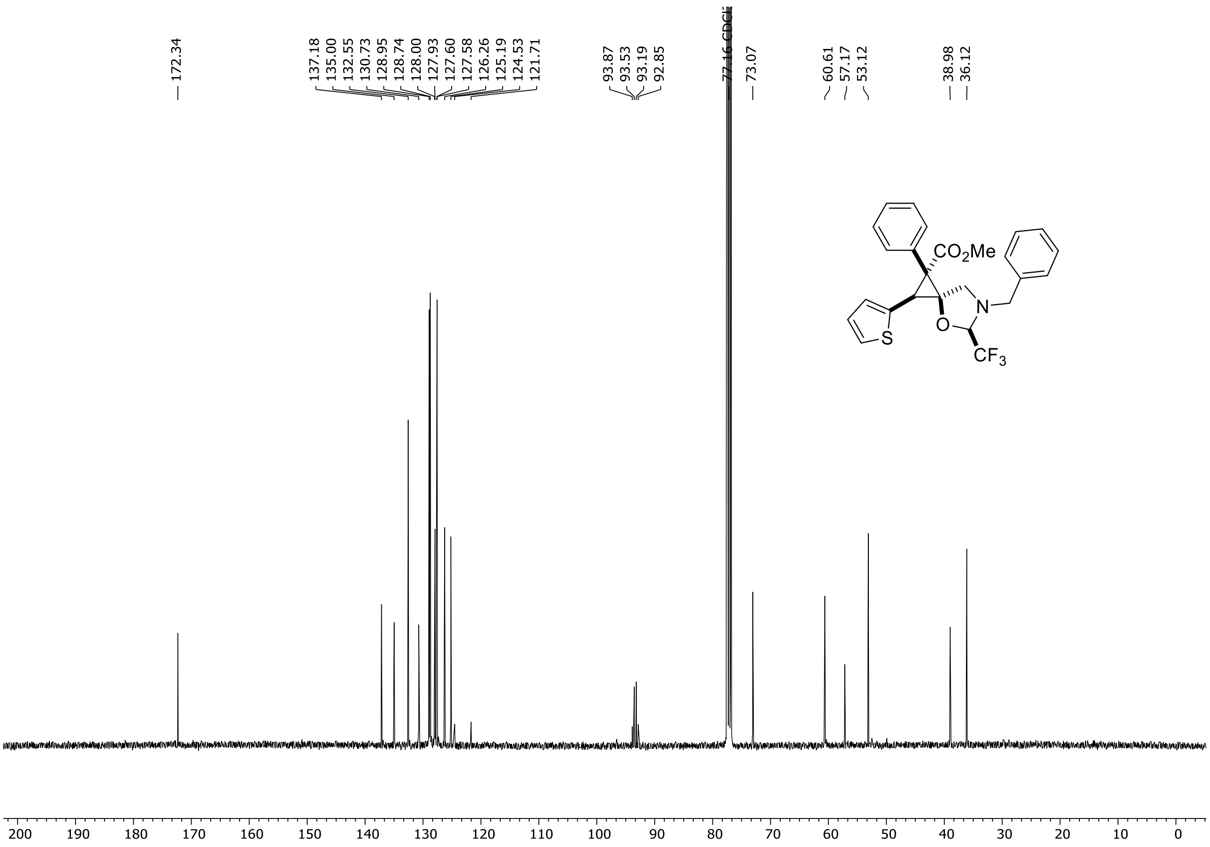

^{19}F NMR Spectrum of 6x (376 MHz, $CDCl_3$)

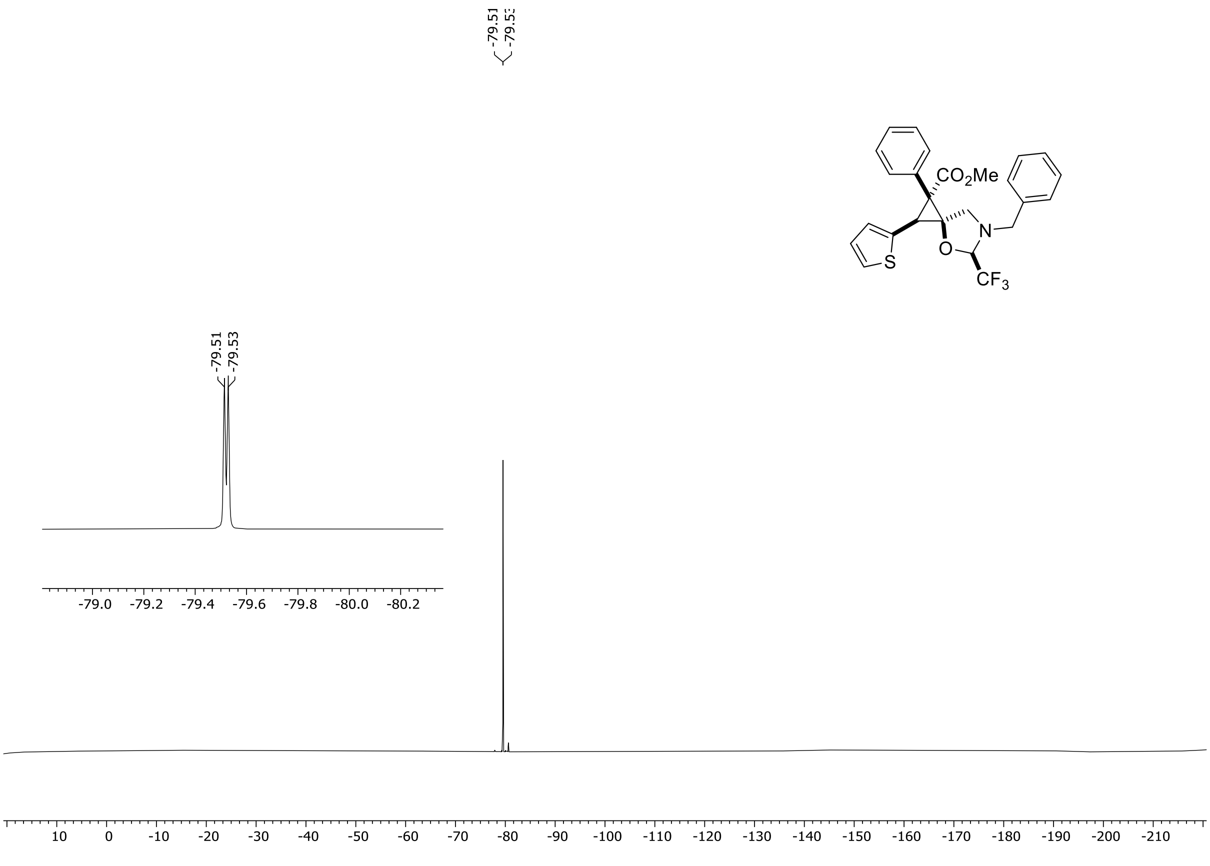

^{1}H NMR Spectrum of 7aa (400 MHz, $CDCl_3$)

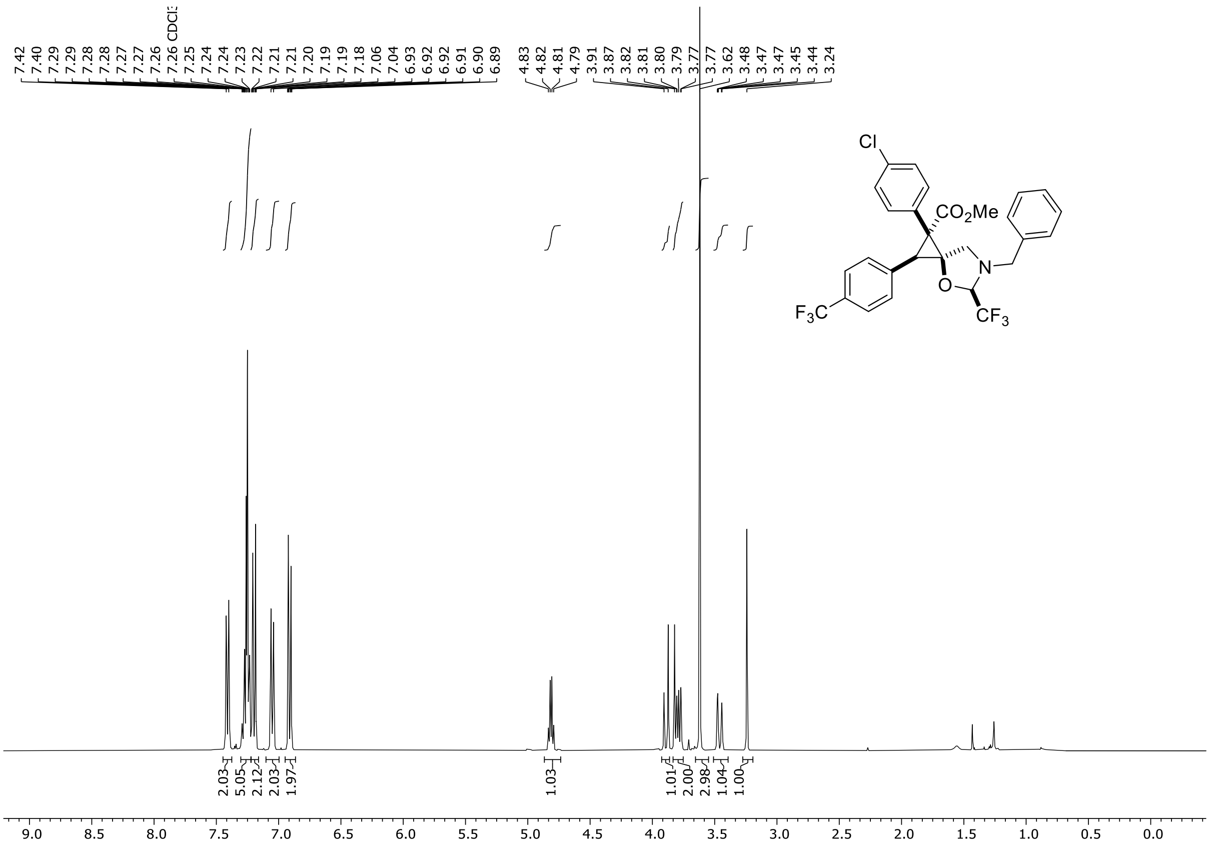

^{13}C NMR Spectrum of 7aa (101 MHz, $CDCl_3$)

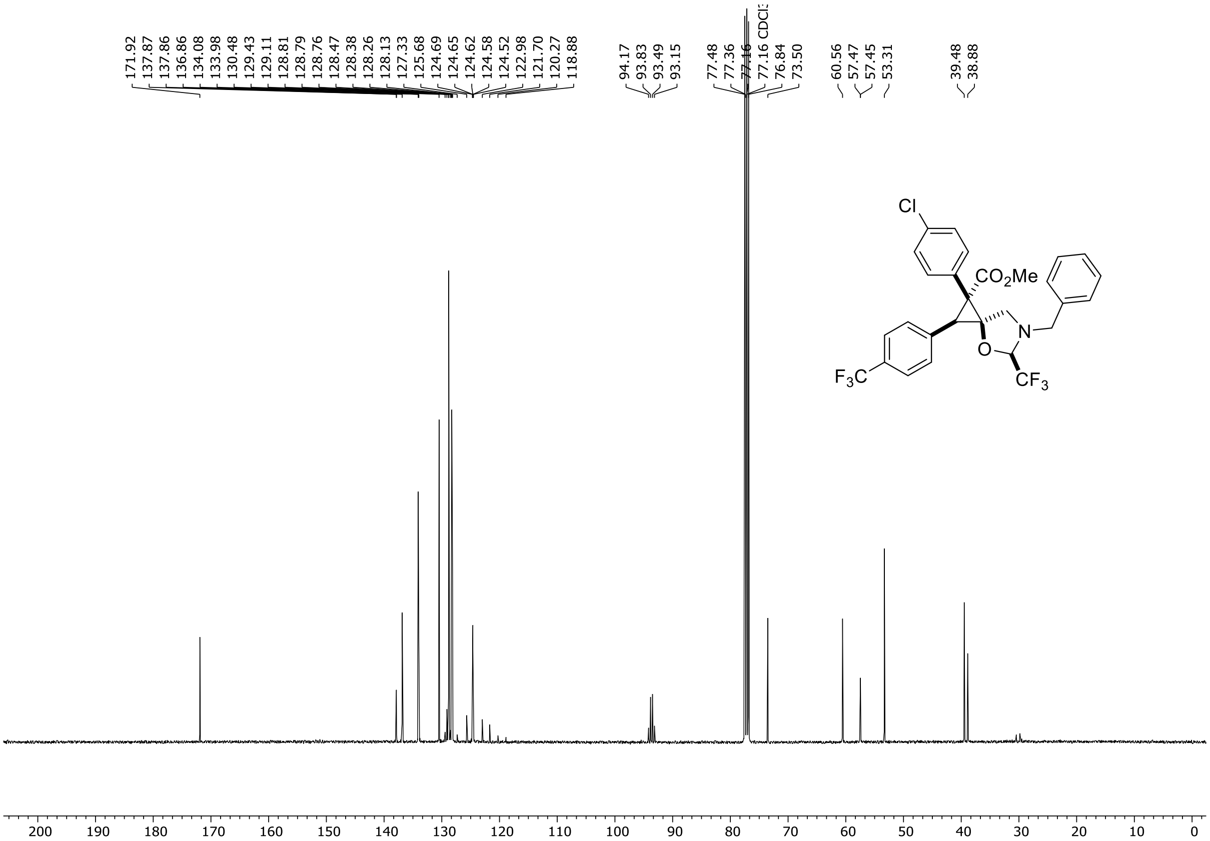

^{19}F NMR Spectrum of 7aa (376 MHz, $CDCl_3$)

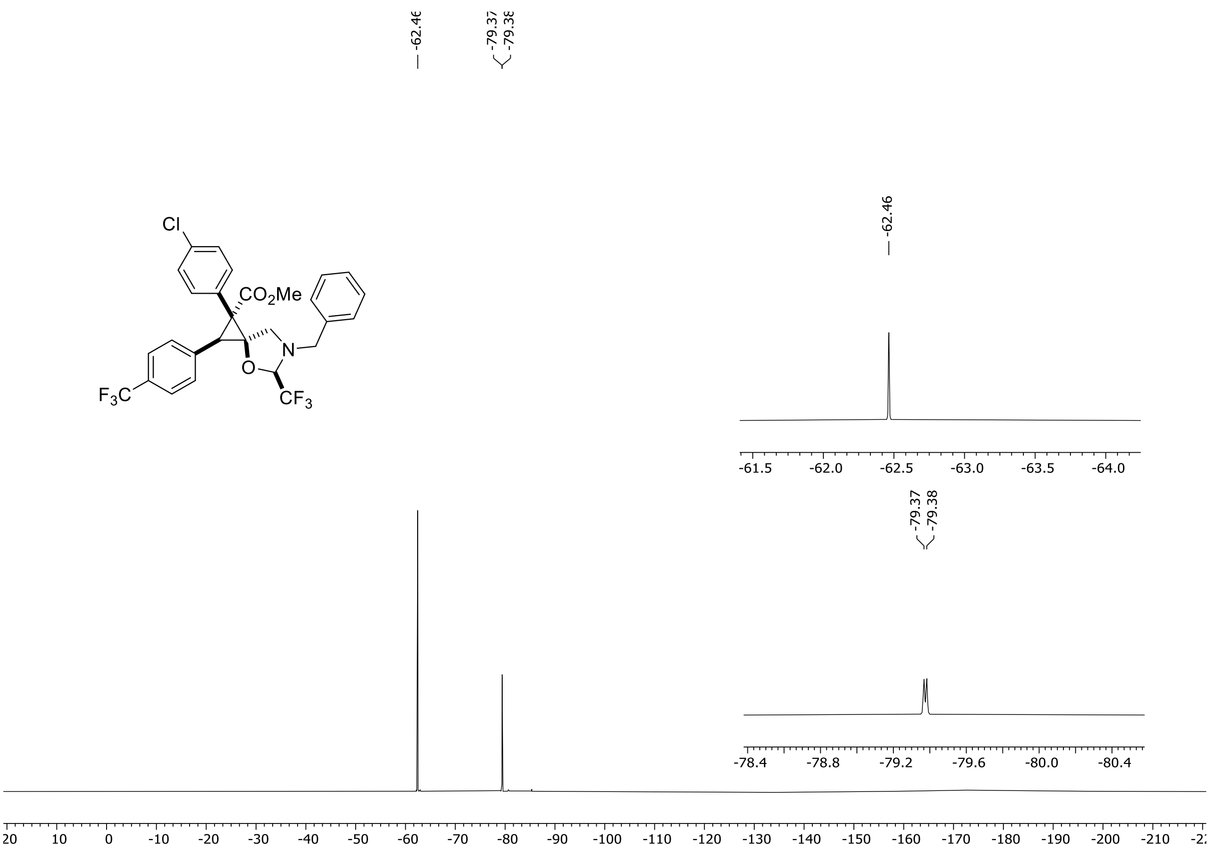

^{1}H NMR Spectrum of 7ab (400 MHz, $CDCl_3$)

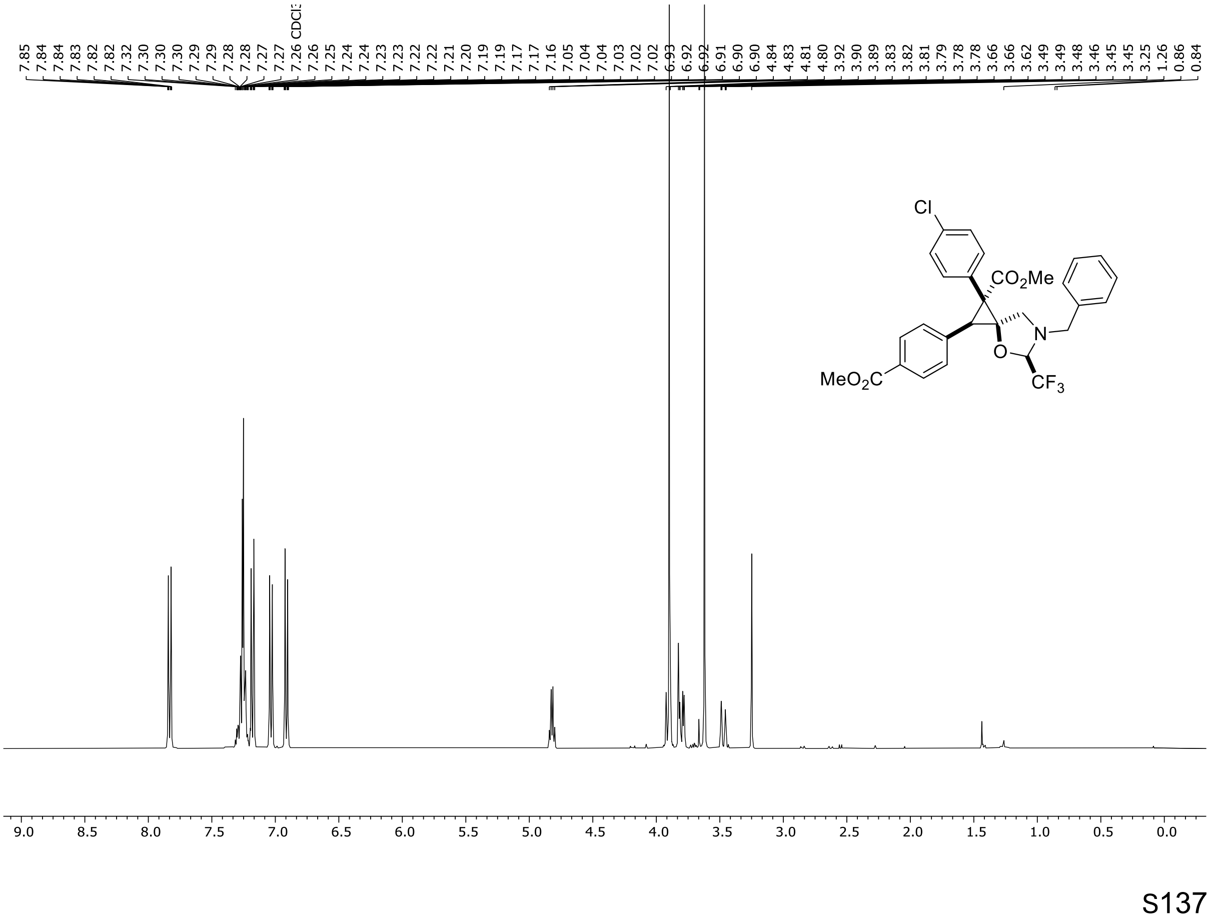

^{13}C NMR Spectrum of 7ab (101 MHz, $CDCl_3$)

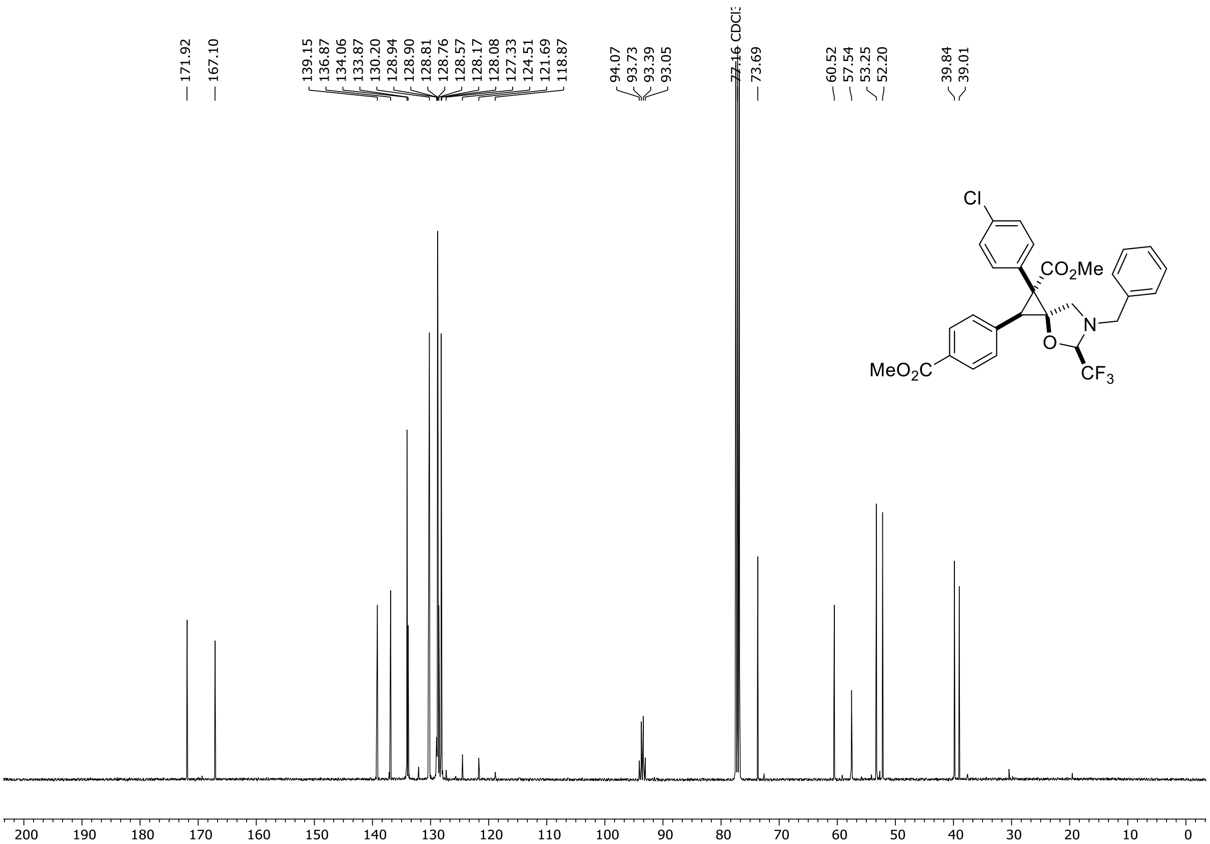

^{19}F NMR Spectrum of 7ab (376 MHz, $CDCl_3$)

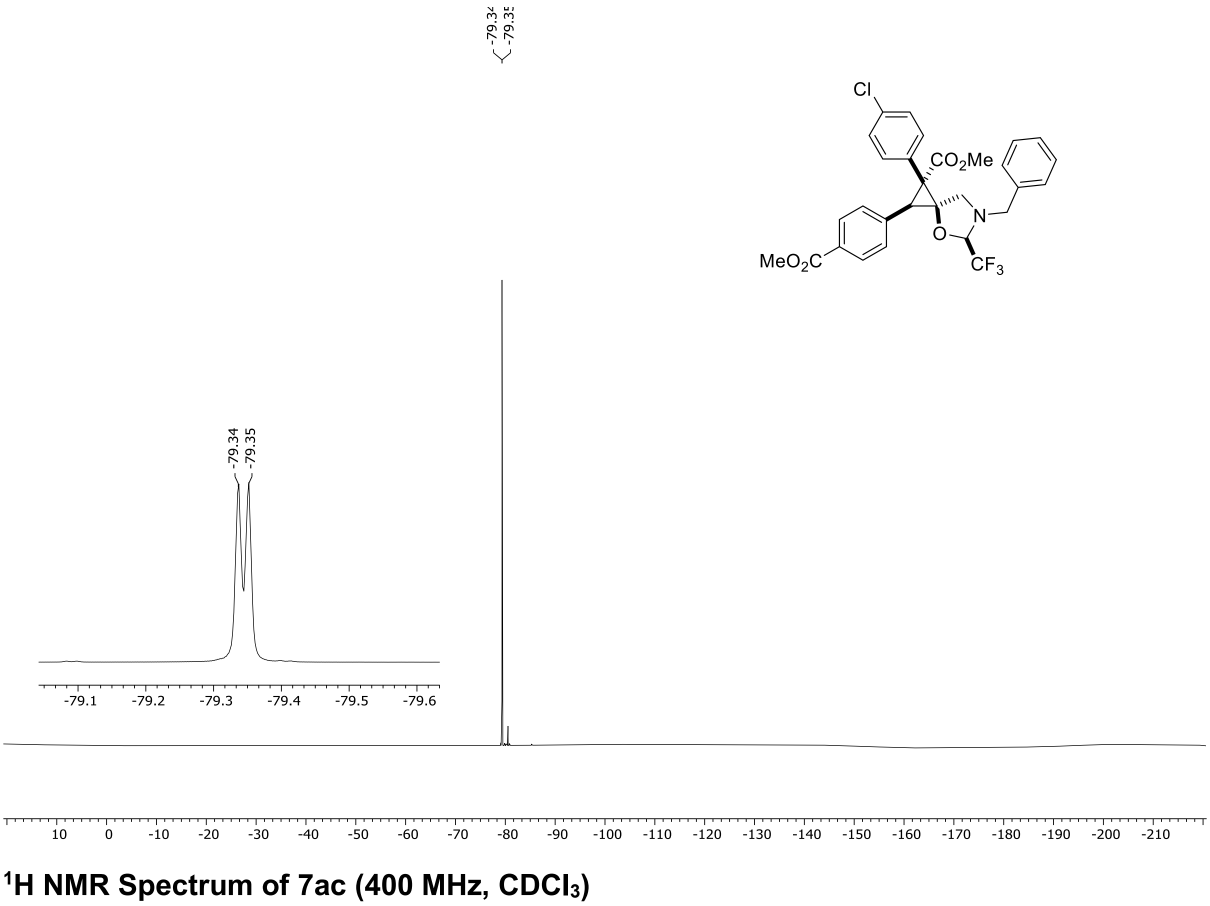

^{1}H NMR Spectrum of 7ac (400 MHz, $CDCl_3$)

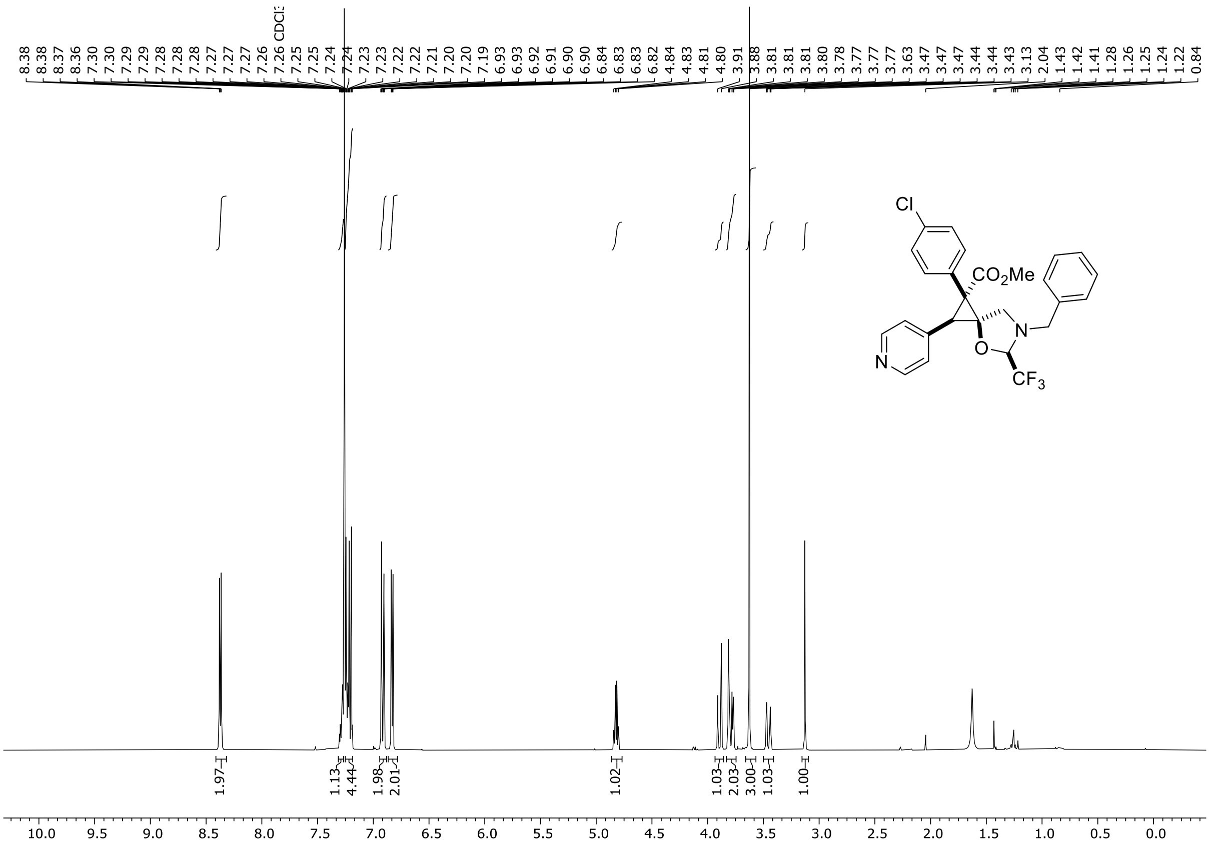

^{13}C NMR Spectrum of 7ac (101 MHz, $CDCl_3$)

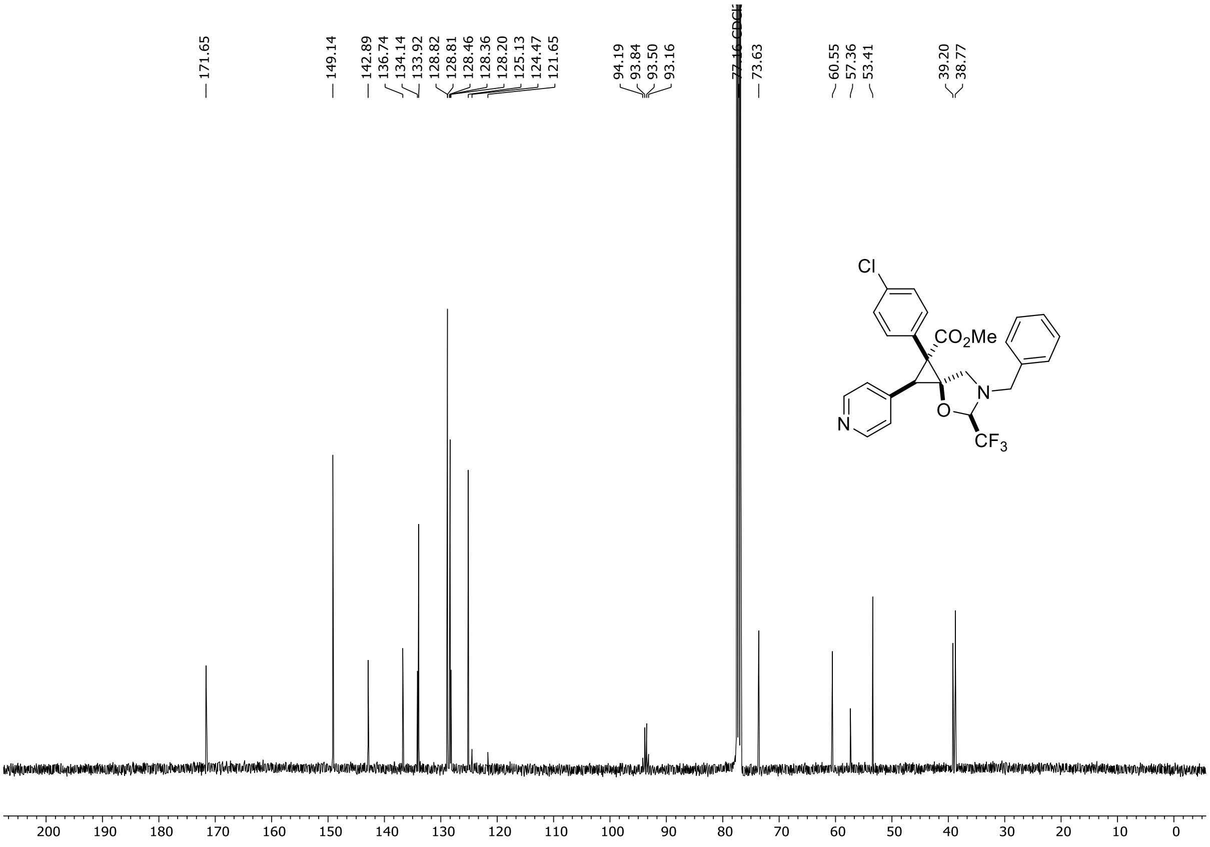

^{19}F NMR Spectrum of 7ac (376 MHz, $CDCl_3$)

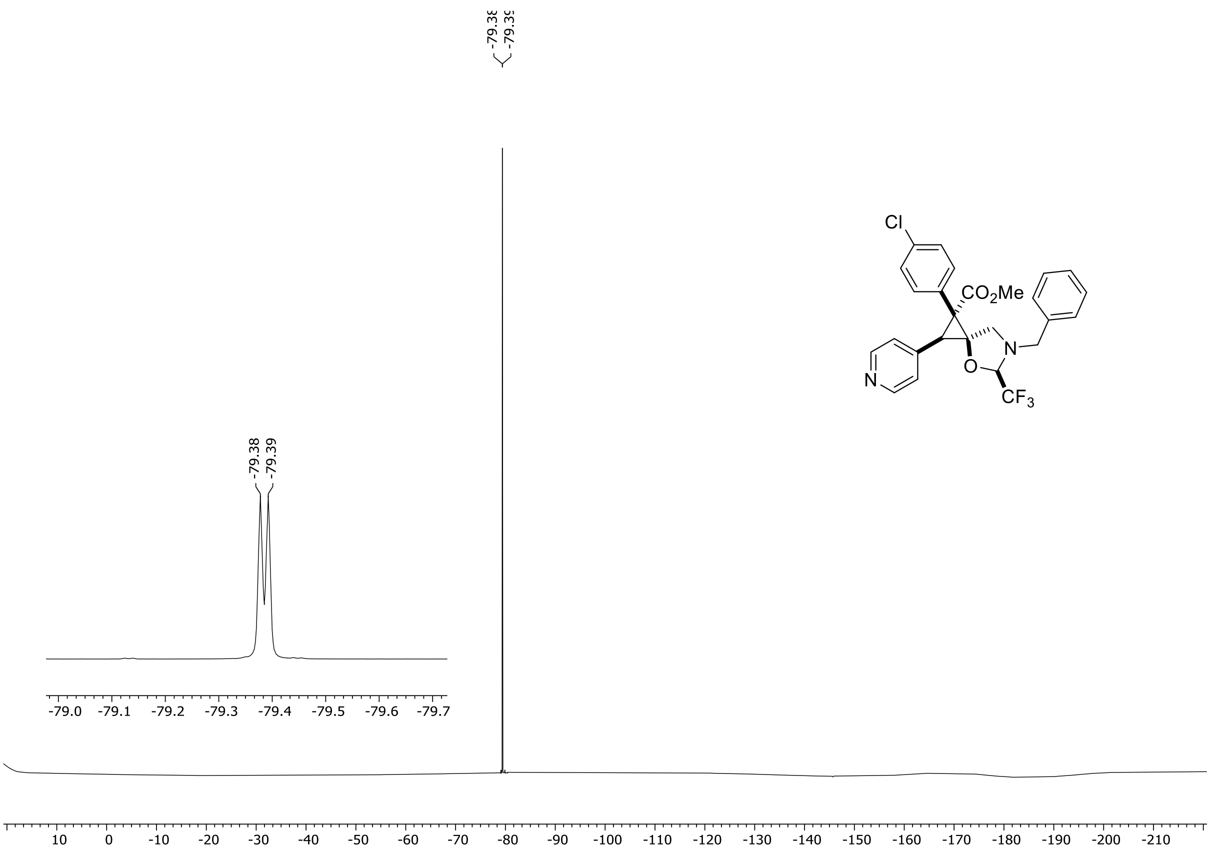

^{1}H NMR Spectrum of 7b (400 MHz, $CDCl_3$)

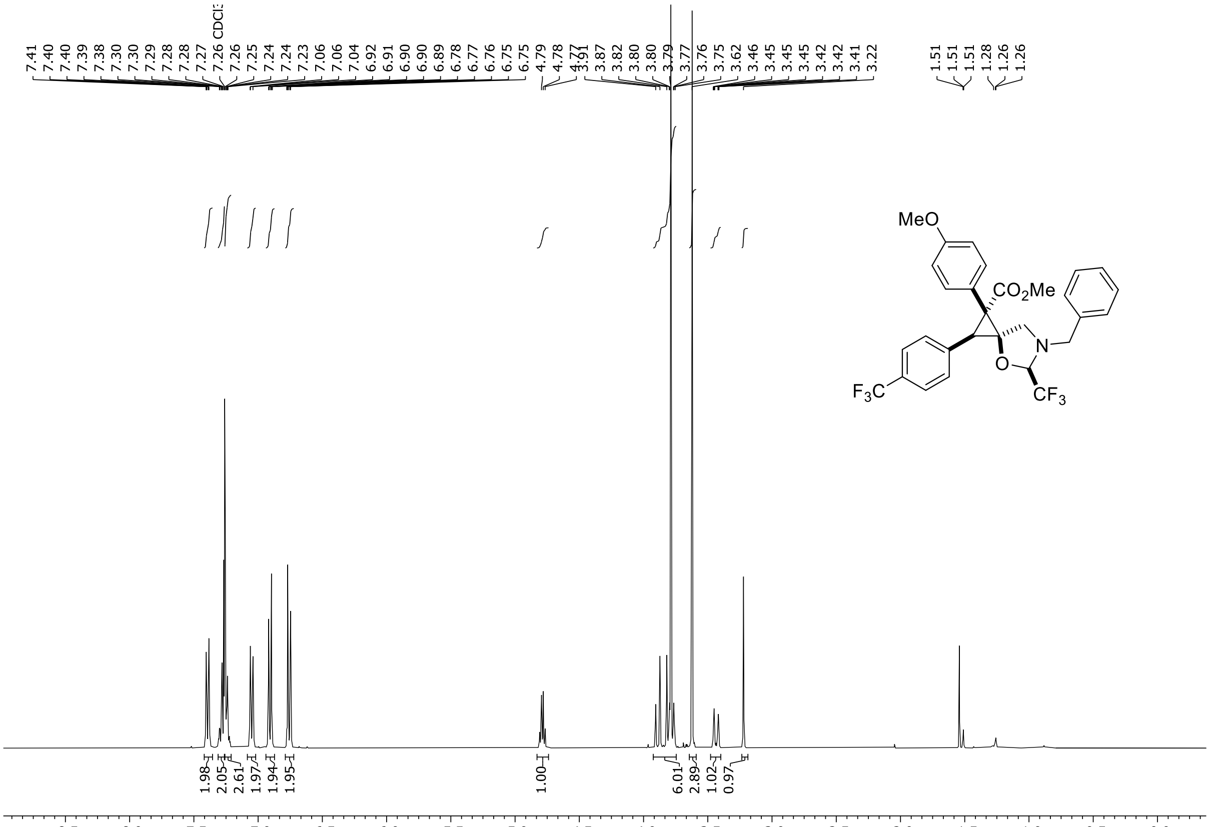

^{13}C NMR Spectrum of 7b (101 MHz, $CDCl_3$)

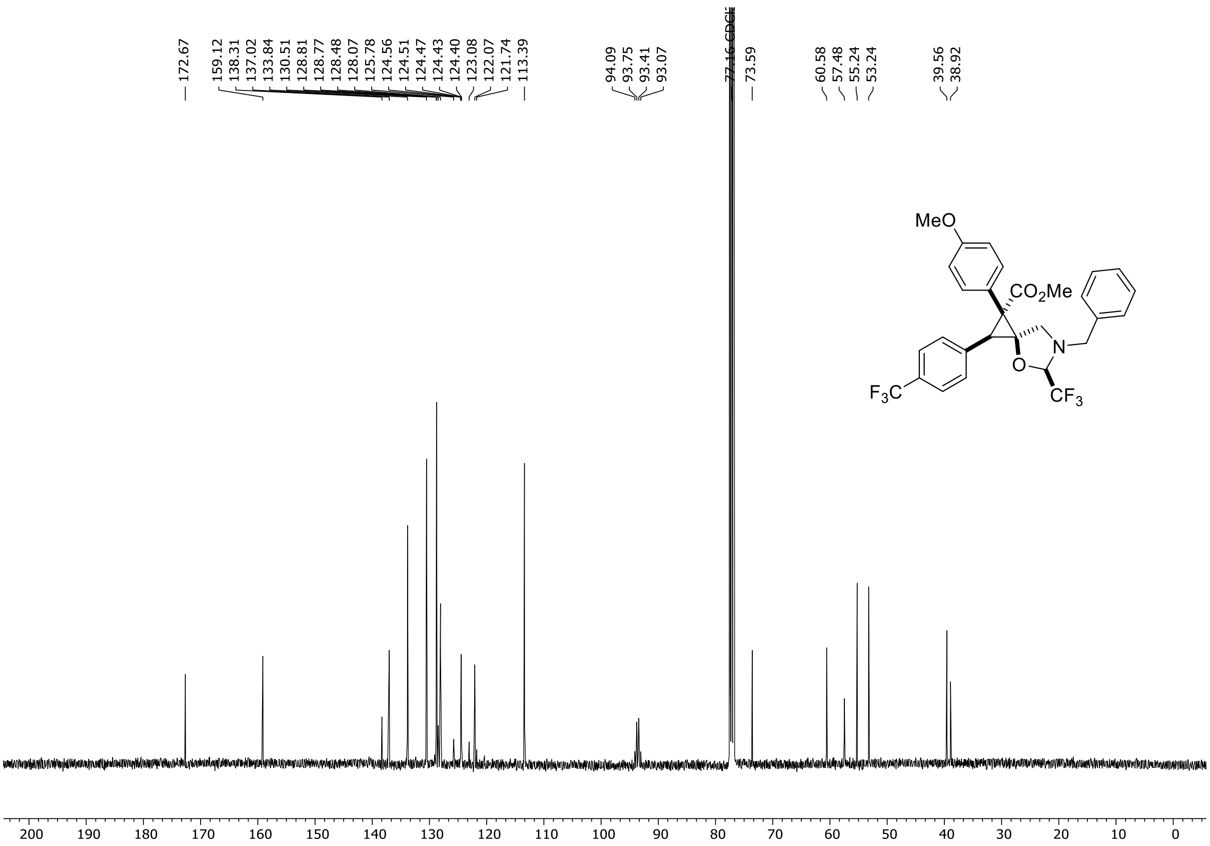

^{19}F NMR Spectrum of 7b (376 MHz, $CDCl_3$)

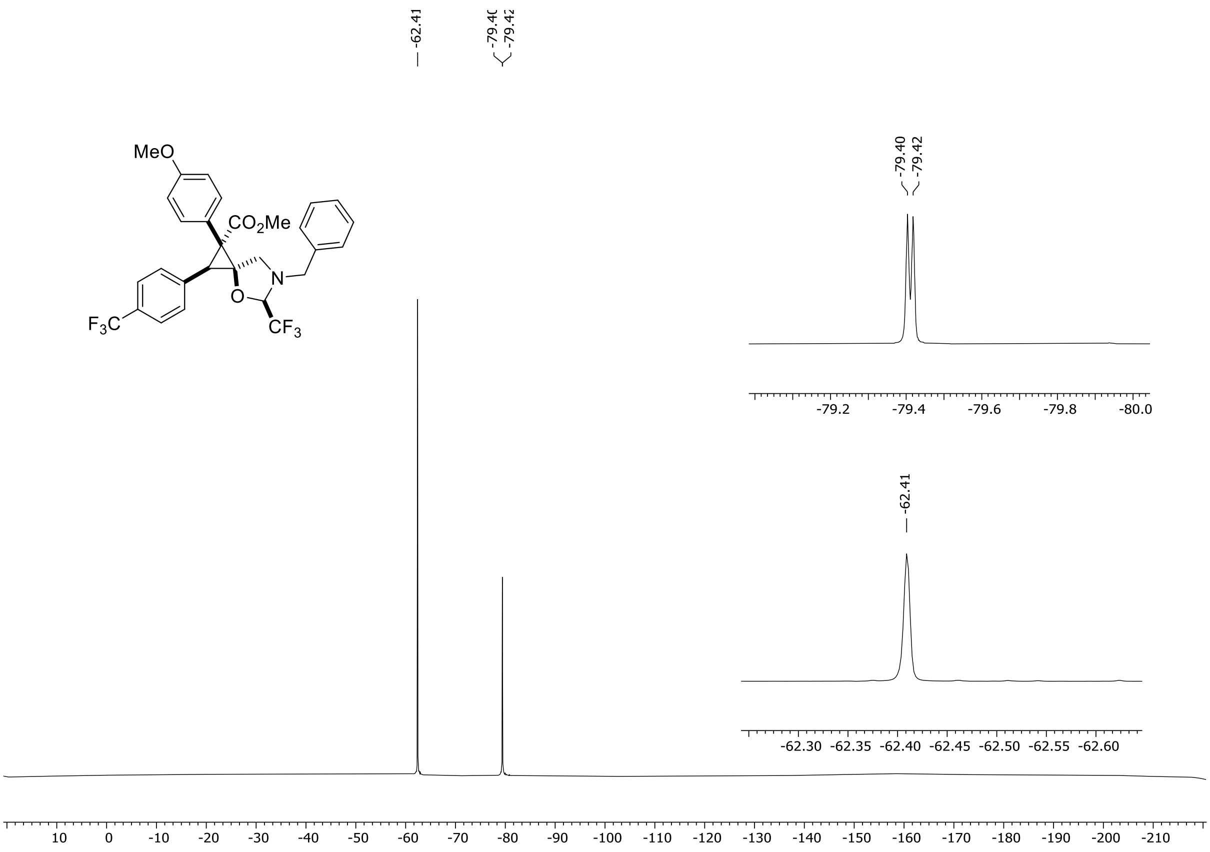

^{1}H NMR Spectrum of 7c (400 MHz, $CDCl_3$)

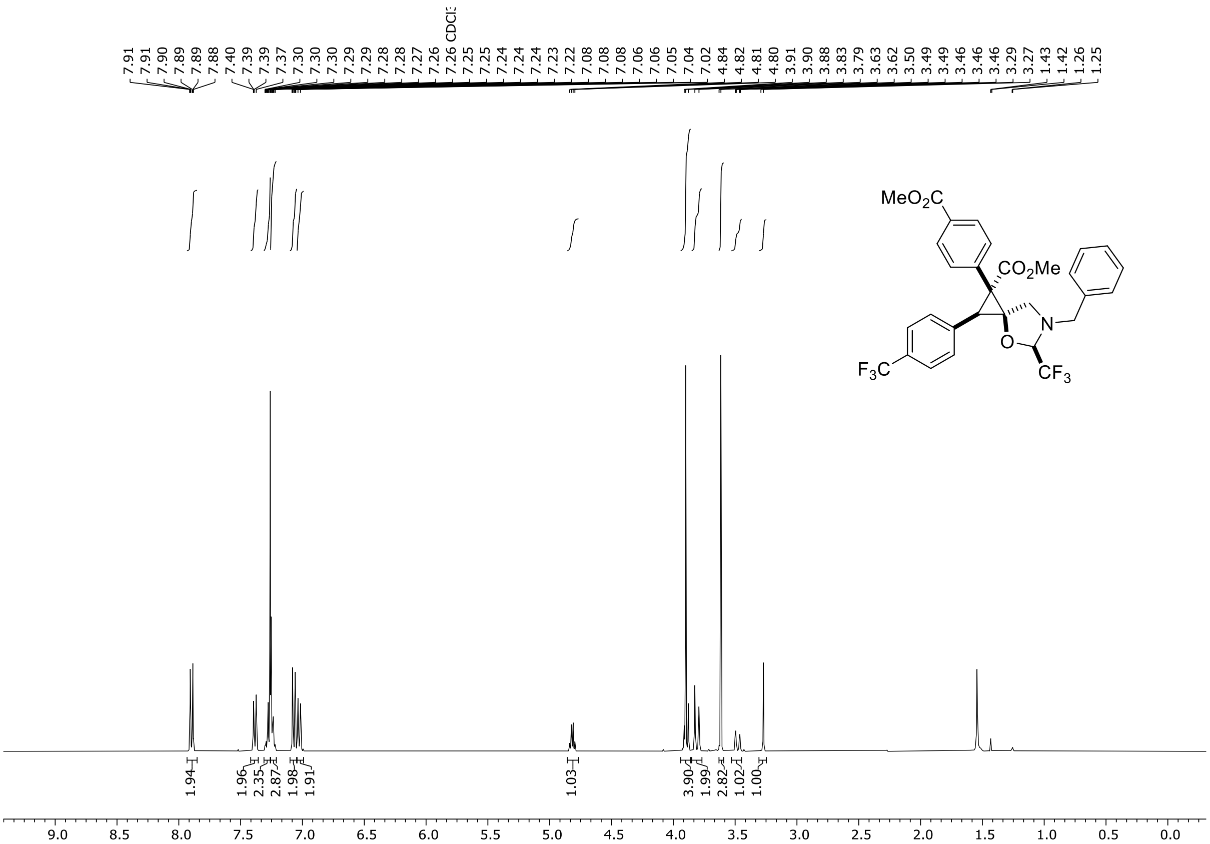

^{13}C NMR Spectrum of 7c (101 MHz, $CDCl_3$)

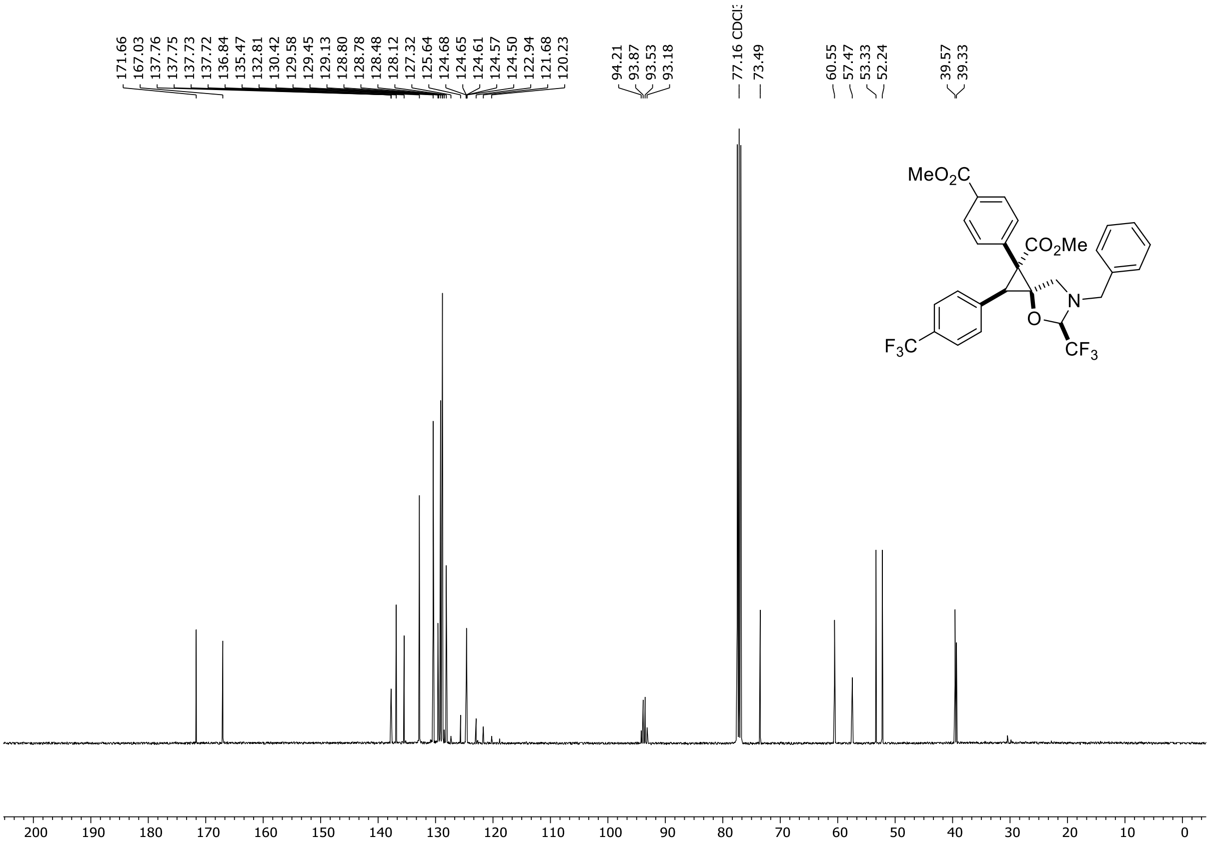

^{19}F NMR Spectrum of 7c (376 MHz, $CDCl_3$)

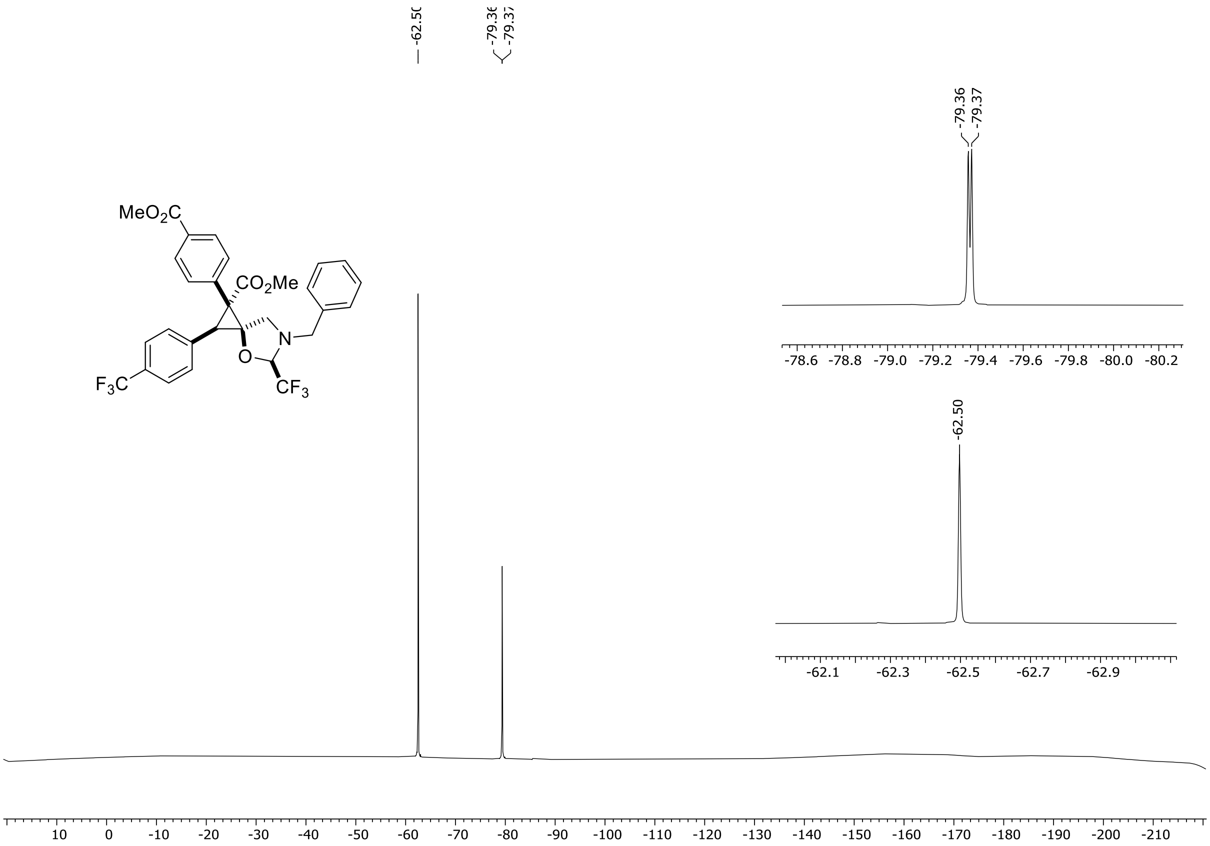

^{1}H NMR Spectrum of 7d (400 MHz, $CDCl_3$)

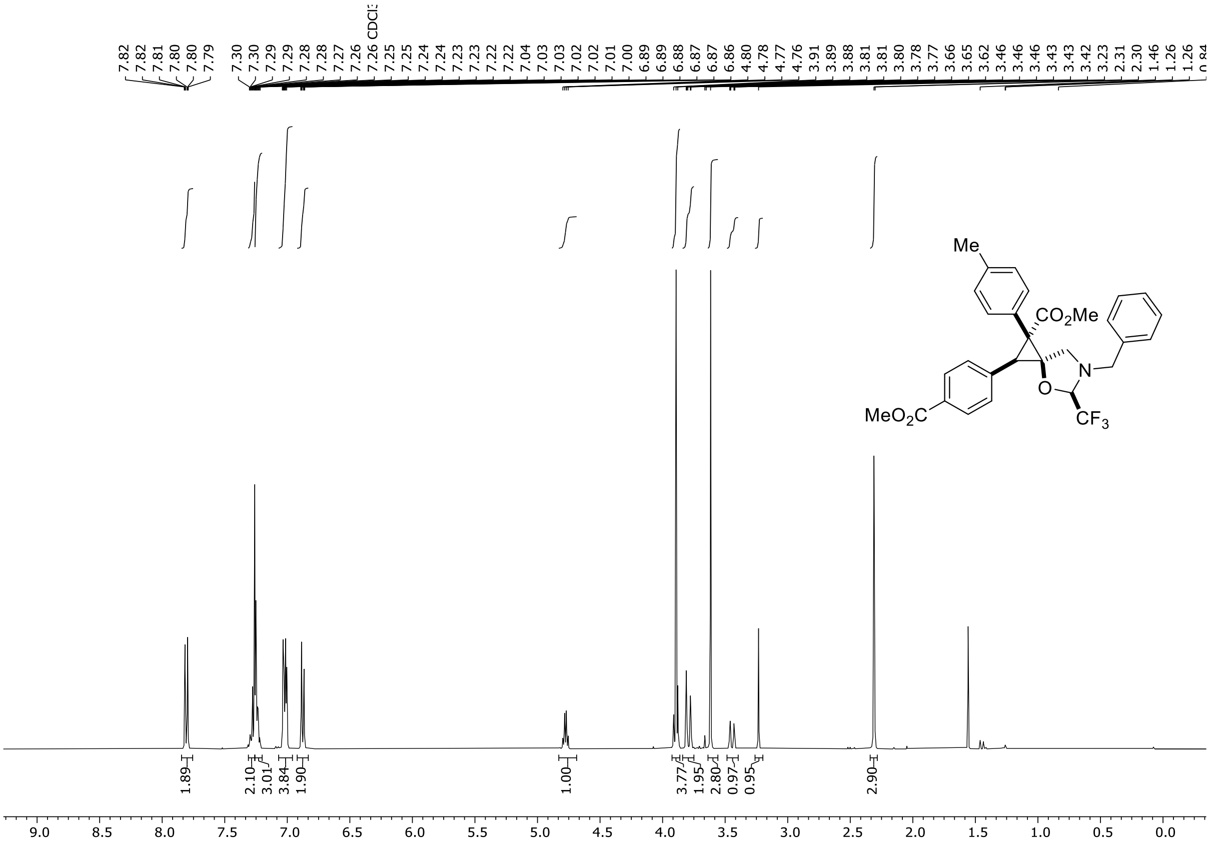

^{13}C NMR Spectrum of 7d (101 MHz, $CDCl_3$)

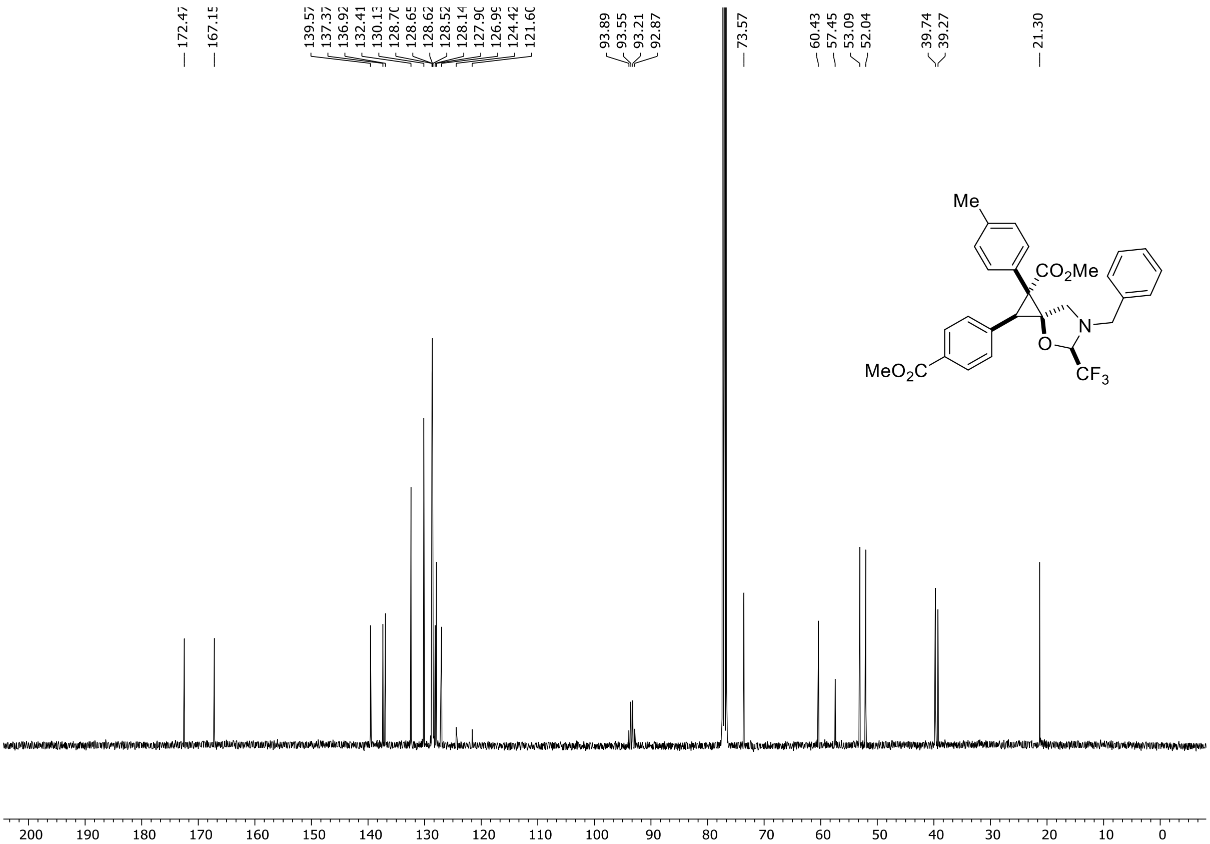

^{19}F NMR Spectrum of 7d (376 MHz, $CDCl_3$)

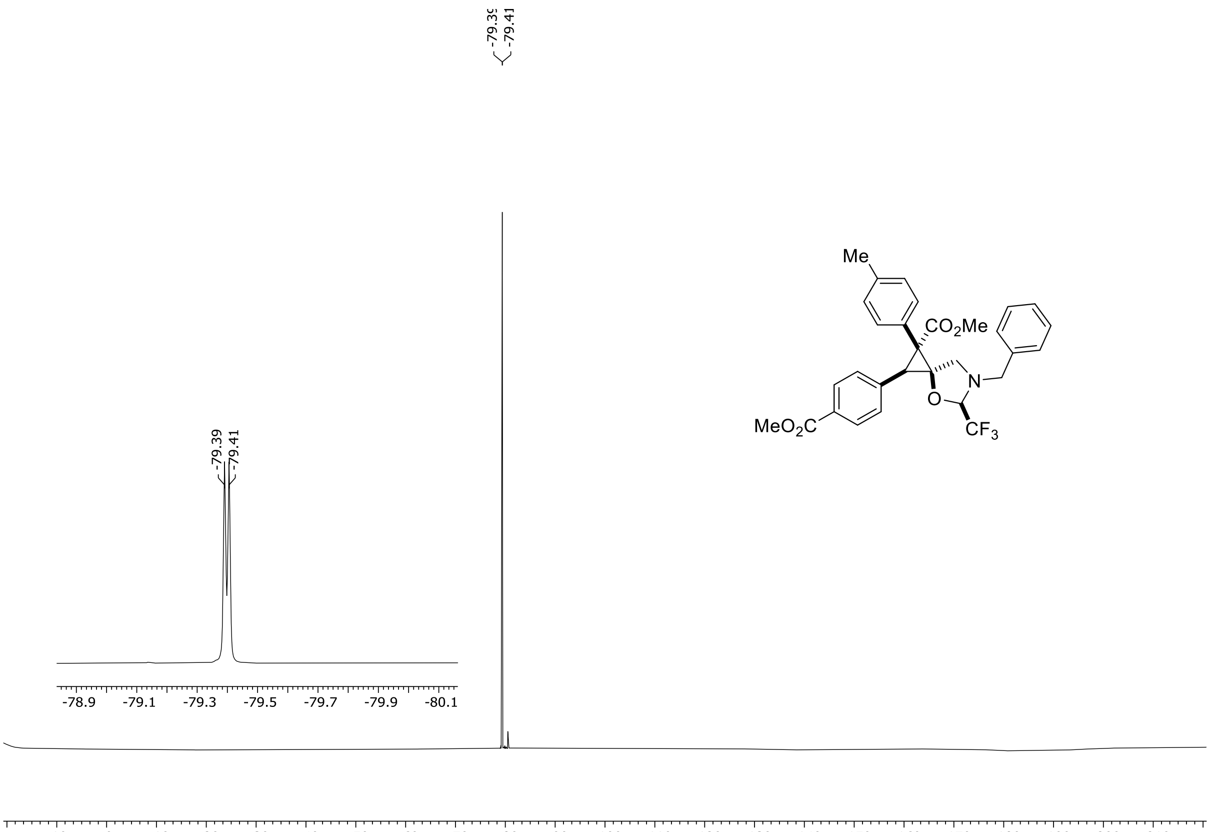

^{1}H NMR Spectrum of 7e (400 MHz, $CDCl_3$)

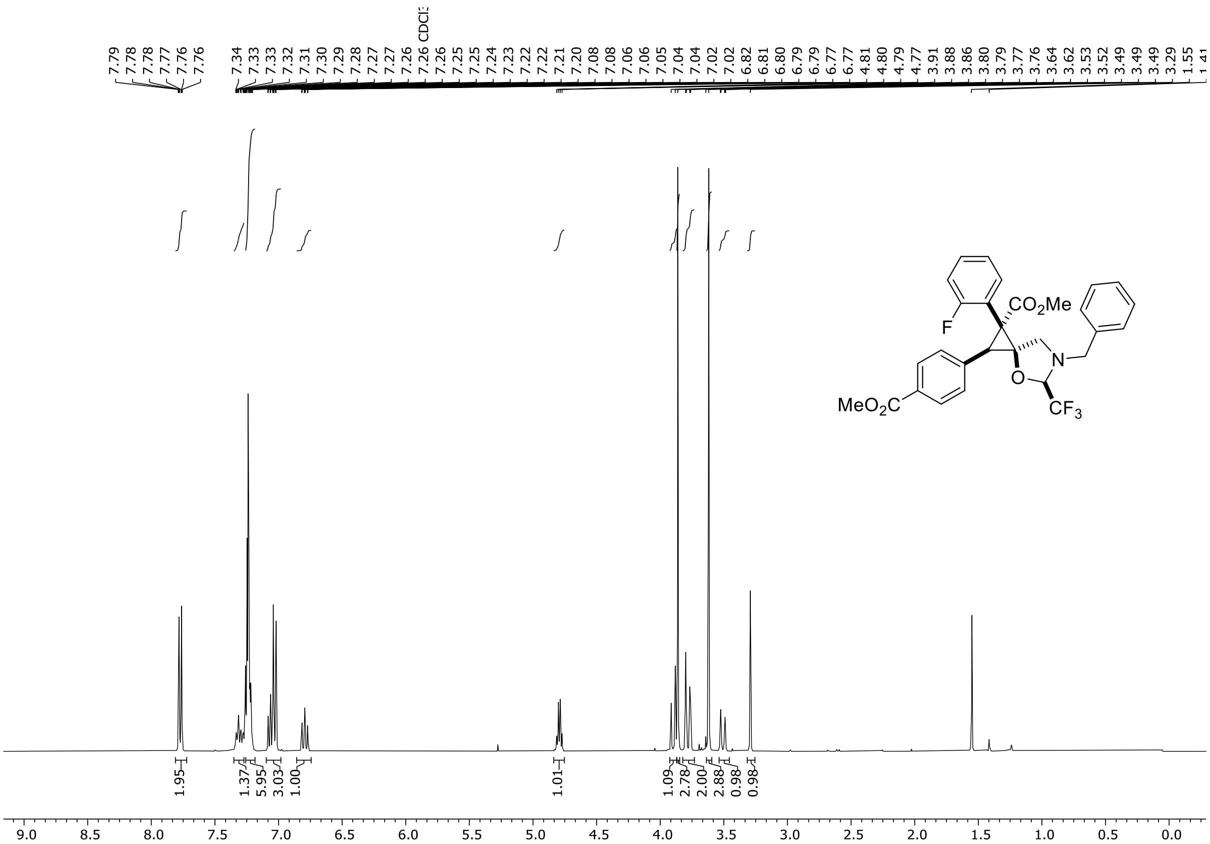

^{13}C NMR Spectrum of 7e (101 MHz, $CDCl_3$)

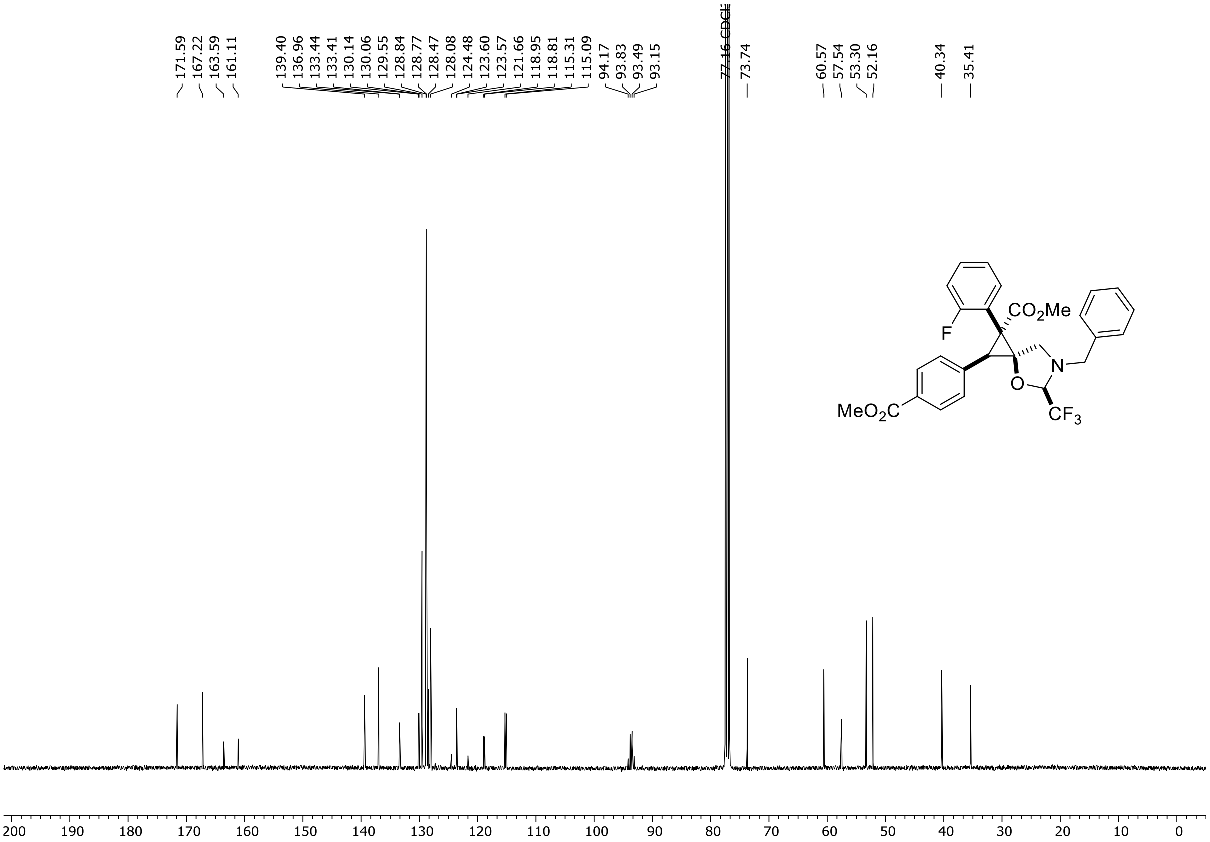

^{19}F NMR Spectrum of 7e (376 MHz, $CDCl_3$)

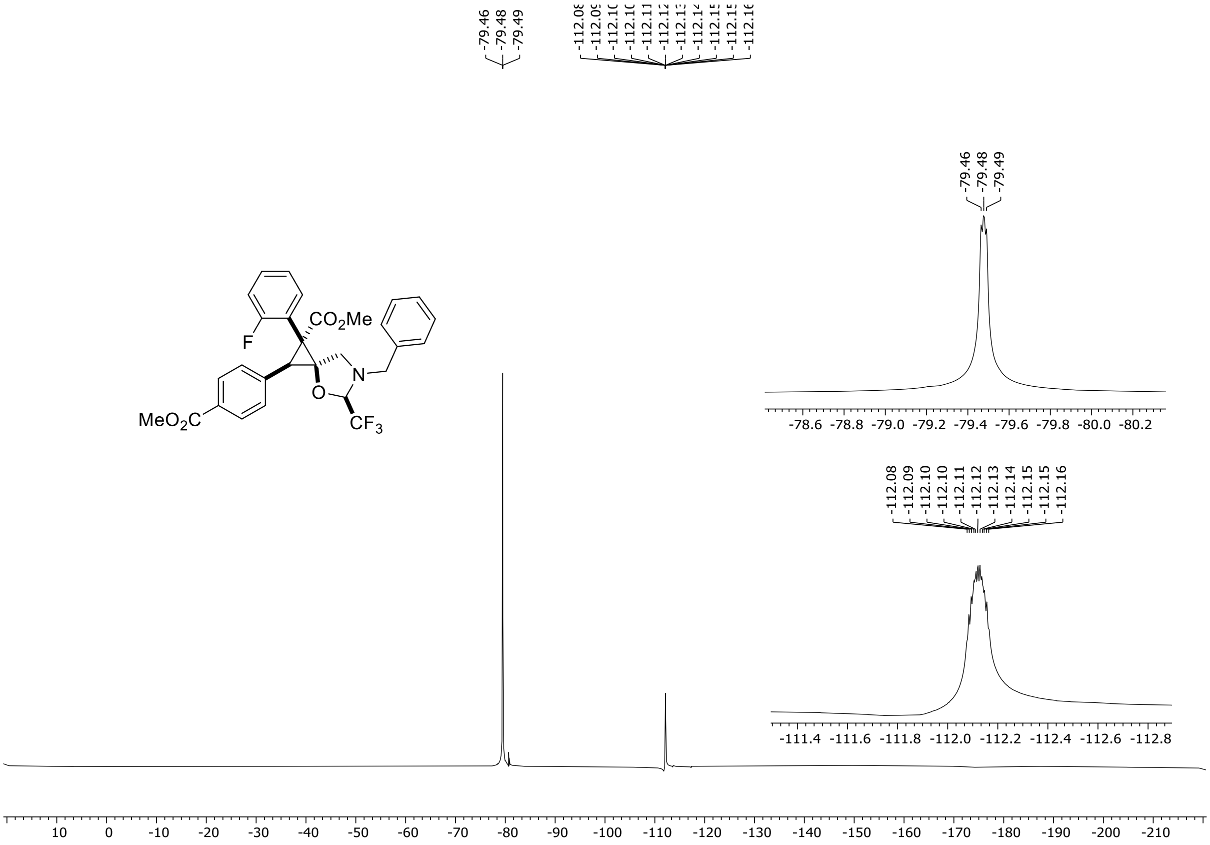

^1H NMR Spectrum of 7f (400 MHz, $CDCl_3$)

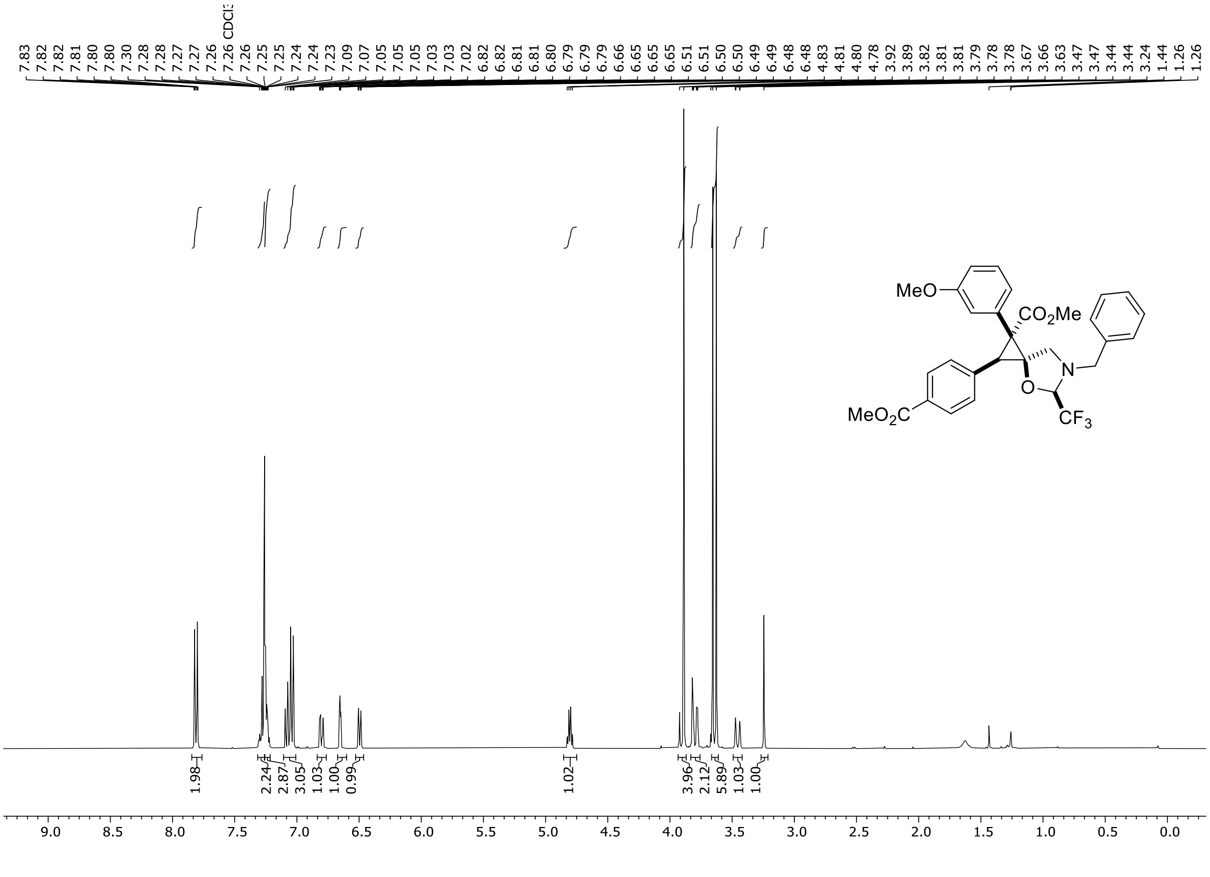

^{13}C NMR Spectrum of 7f (101 MHz, CDCl$_3$)

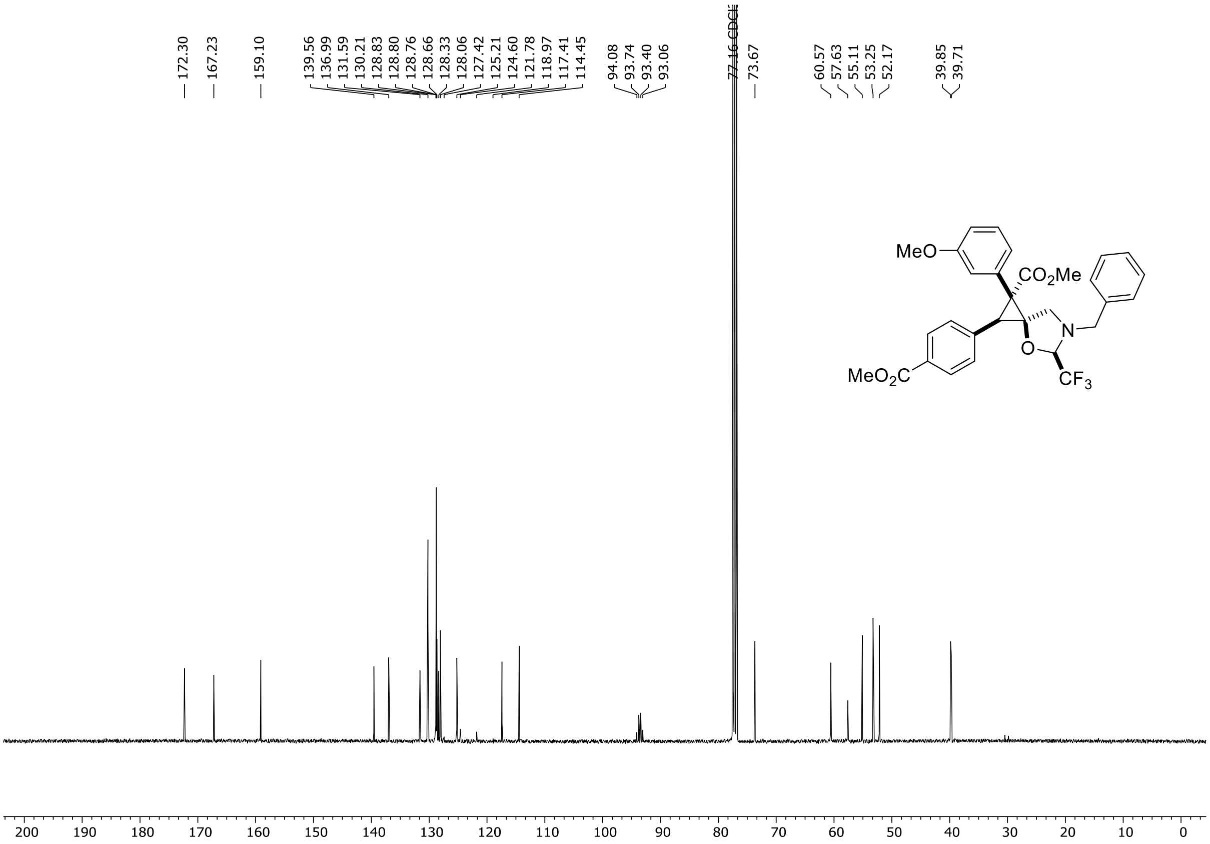

^{19}F NMR Spectrum of 7f (376 MHz, CDCl$_3$)

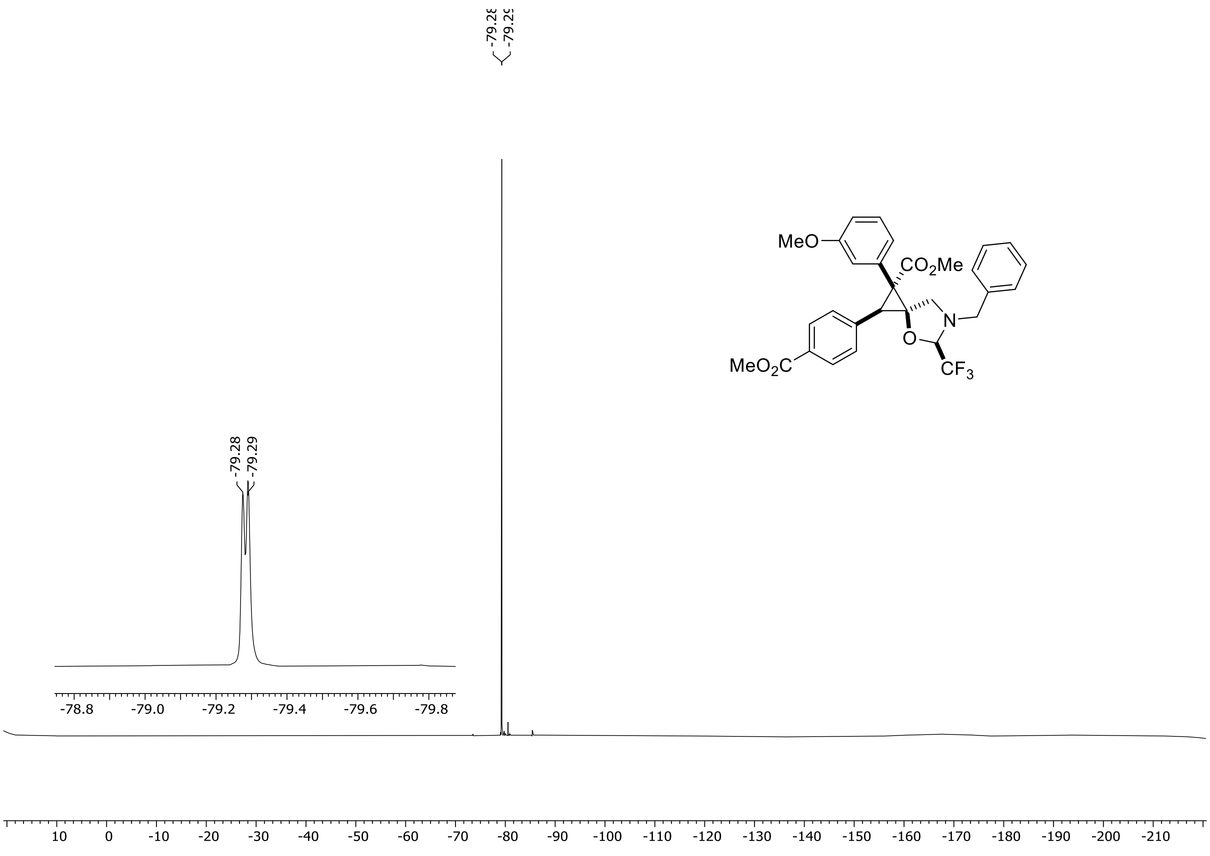

^{1}H NMR Spectrum of 7g (400 MHz, CDCl$_3$)

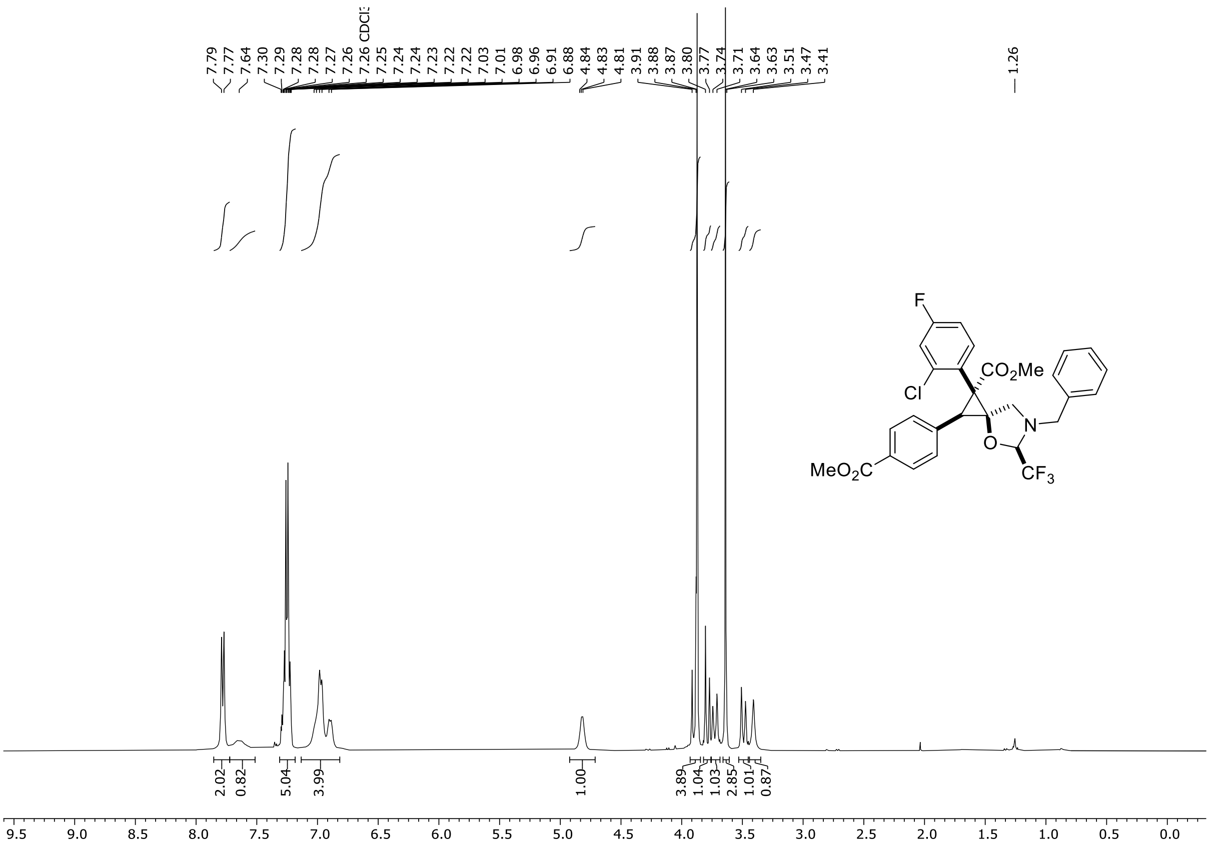

^{13}C NMR Spectrum of 7g (101 MHz, $CDCl_3$)

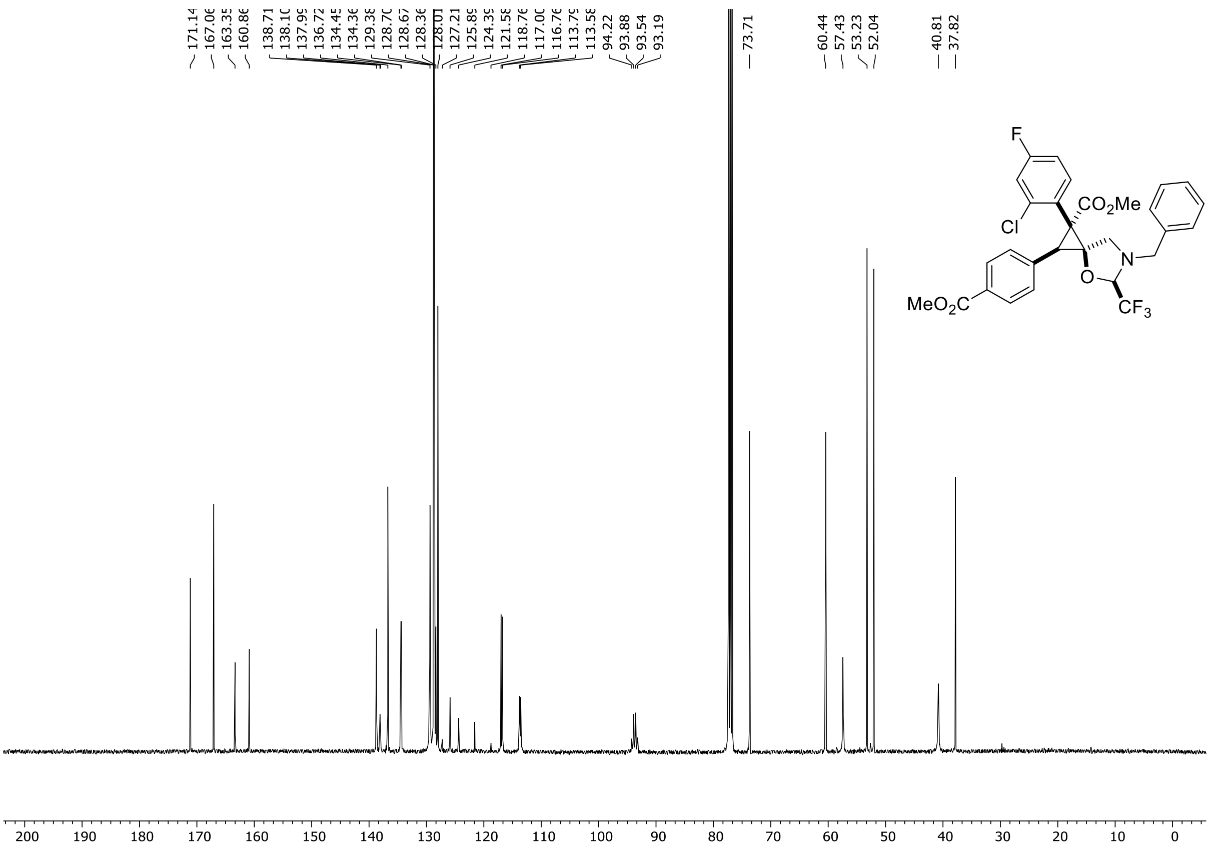

^{19}F NMR Spectrum of 7g (376 MHz, $CDCl_3$)

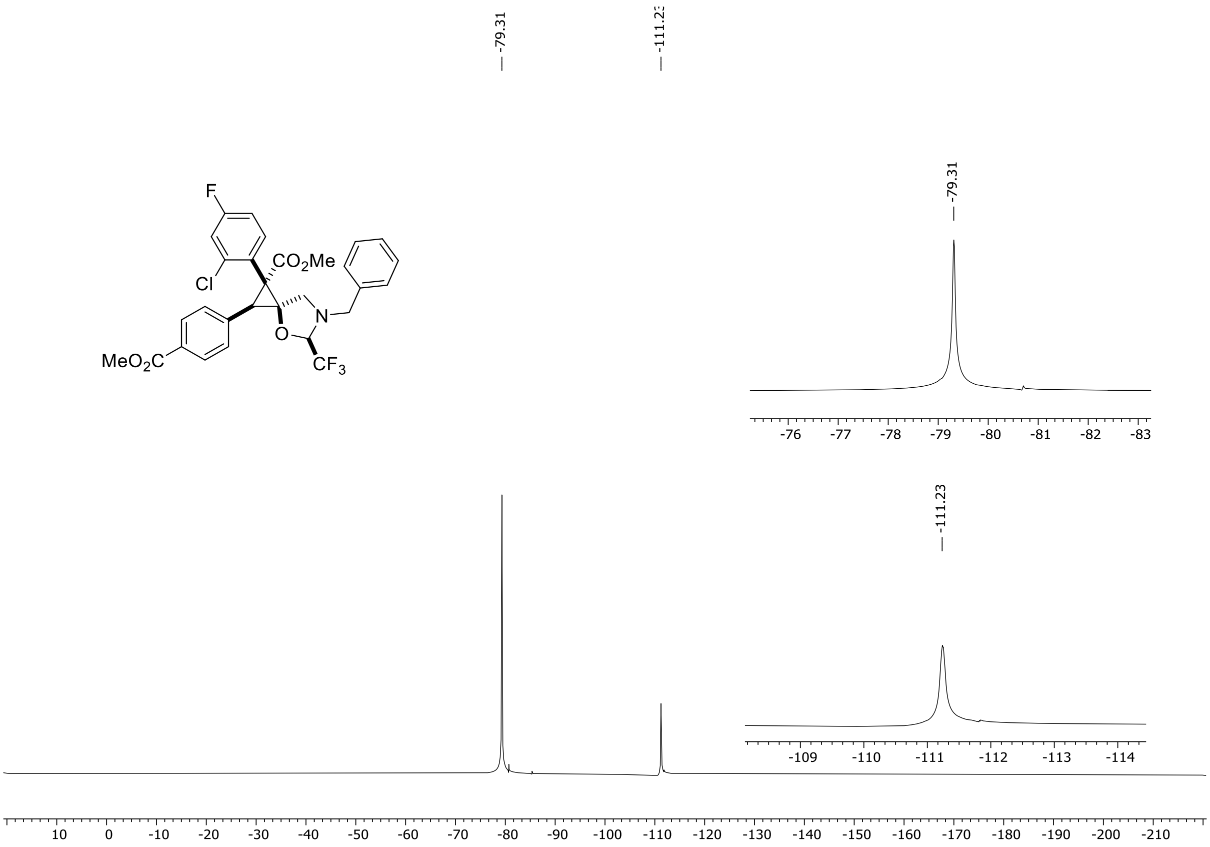

^{1}H NMR Spectrum of 7h (400 MHz, $CDCl_3$)

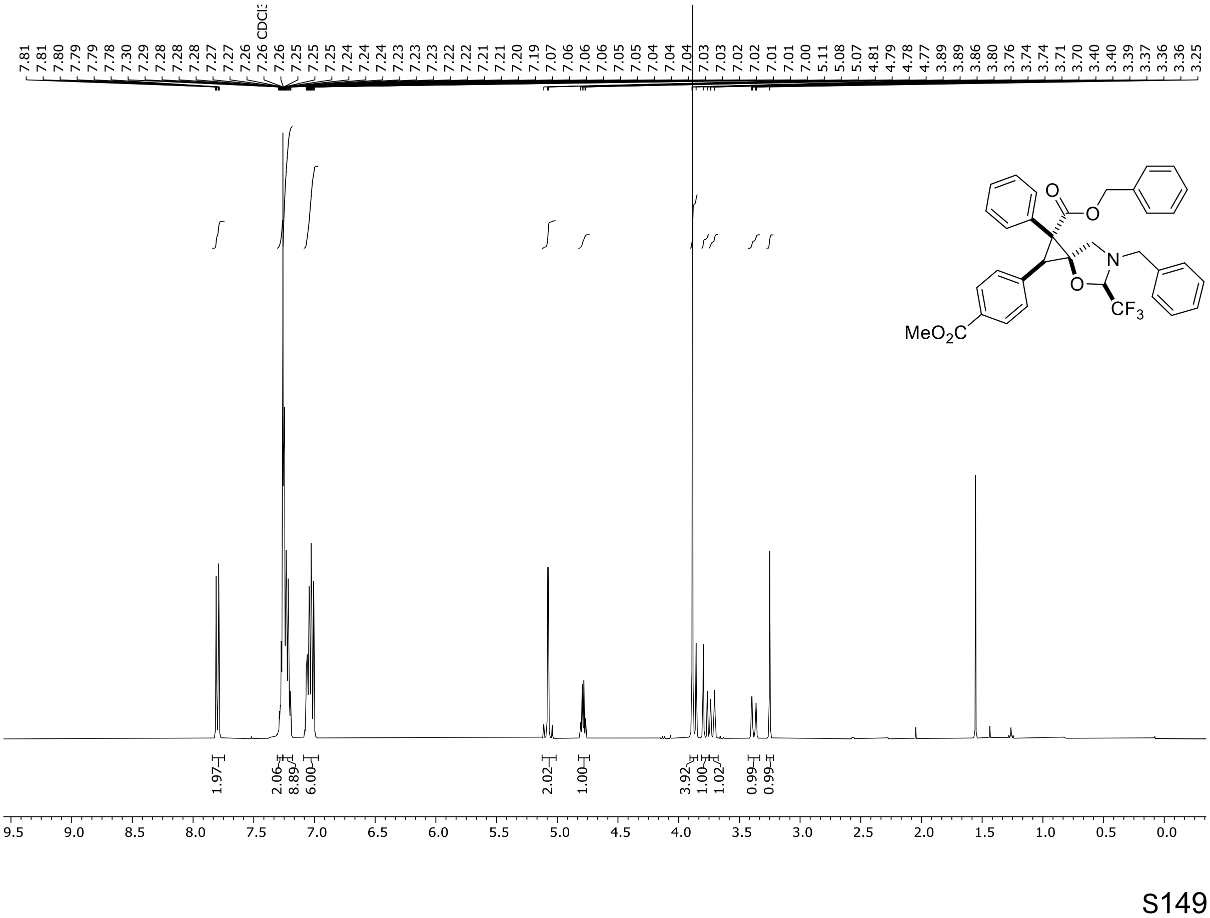

^{13}C NMR Spectrum of 7h (101 MHz, $CDCl_3$)

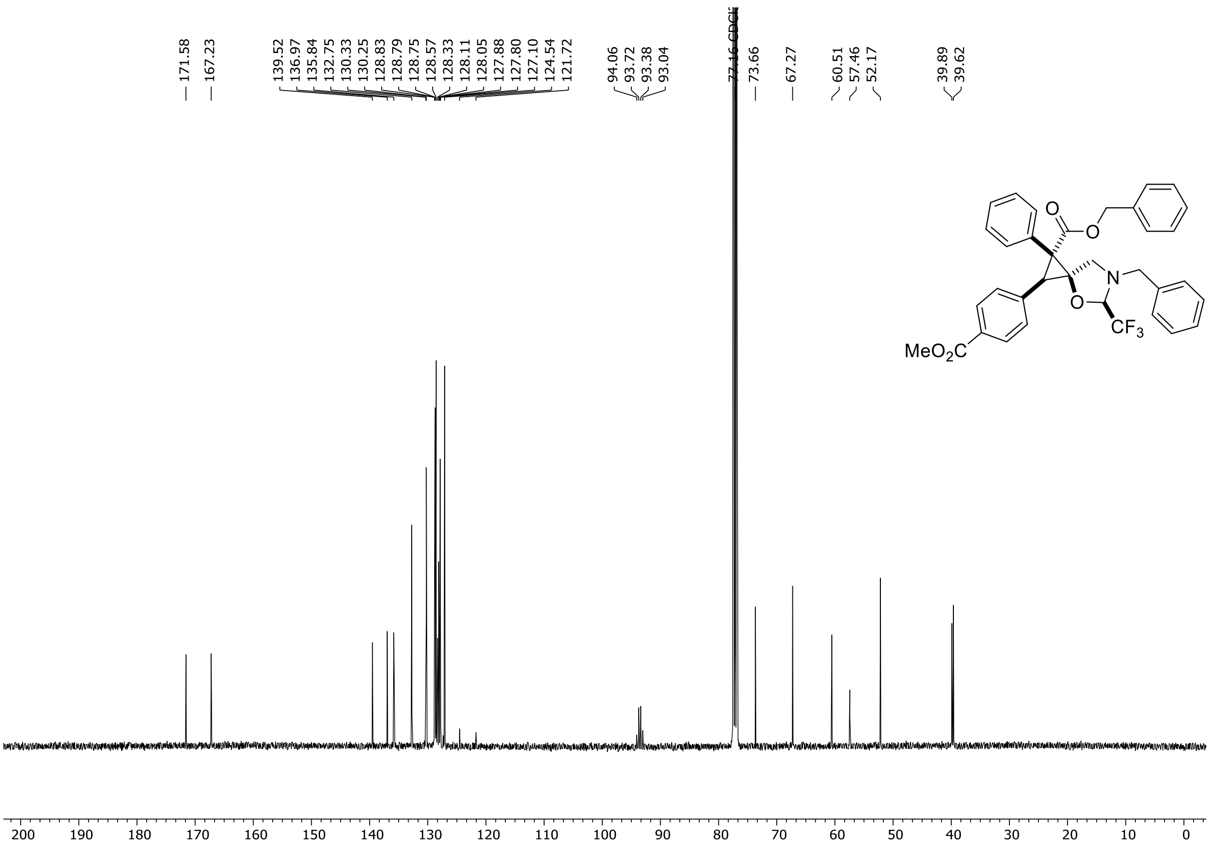

^{19}F NMR Spectrum of 7h (376 MHz, $CDCl_3$)

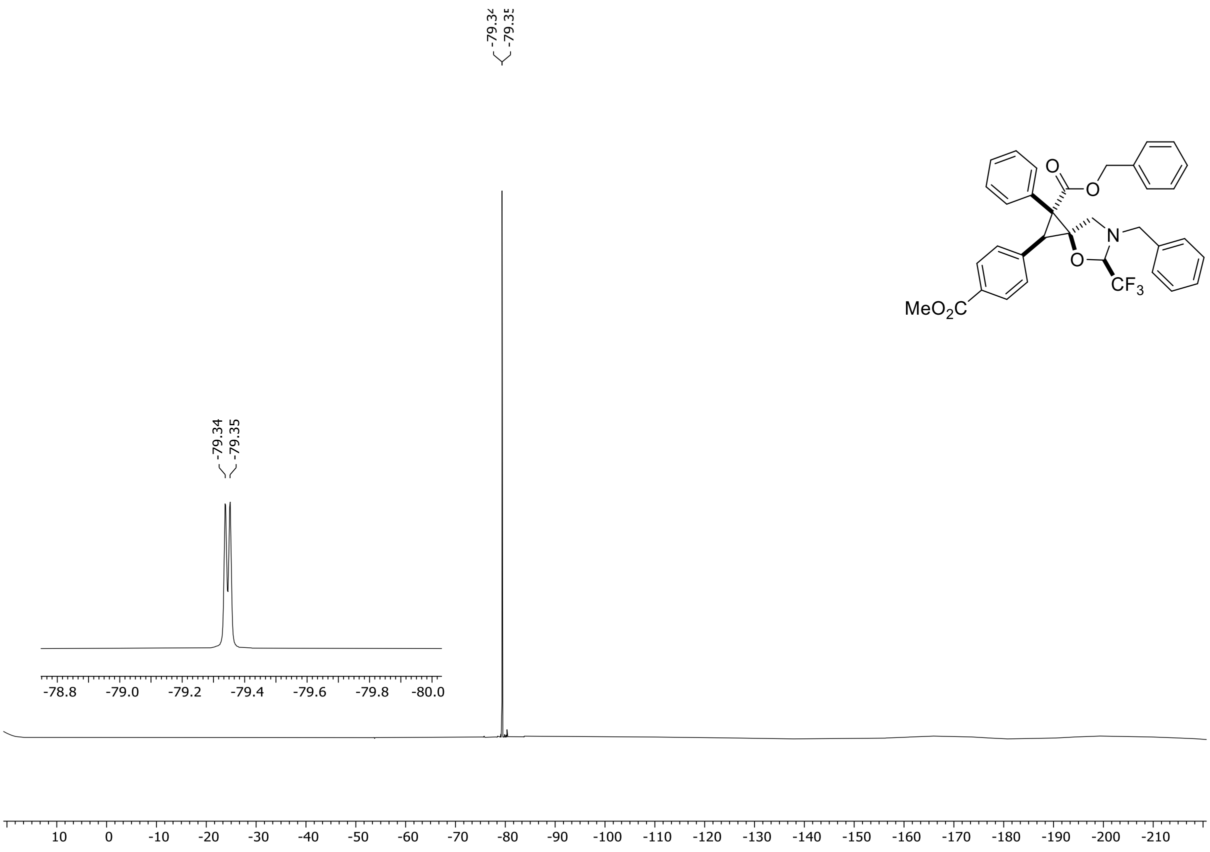

^{1}H NMR Spectrum of 7i (400 MHz, $CDCl_3$)

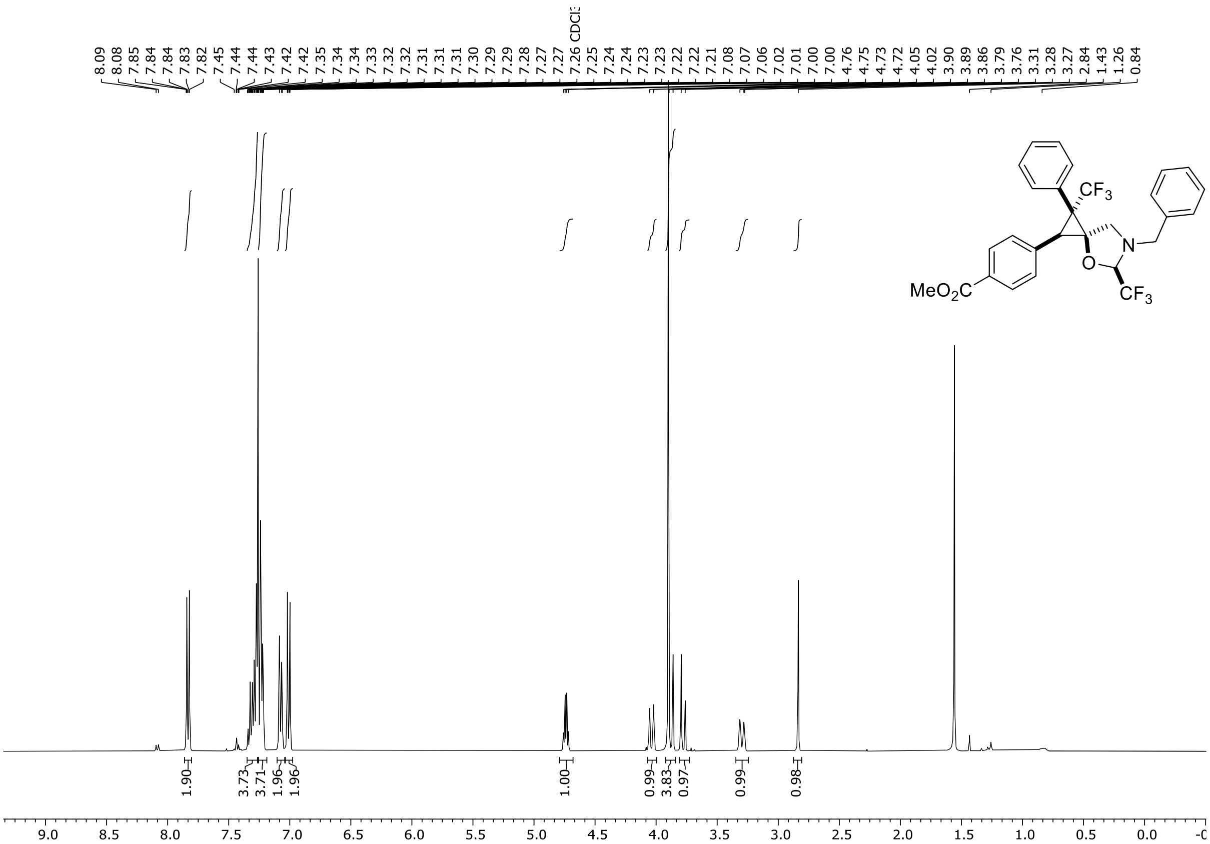

^{13}C NMR Spectrum of 7i (101 MHz, $CDCl_3$)

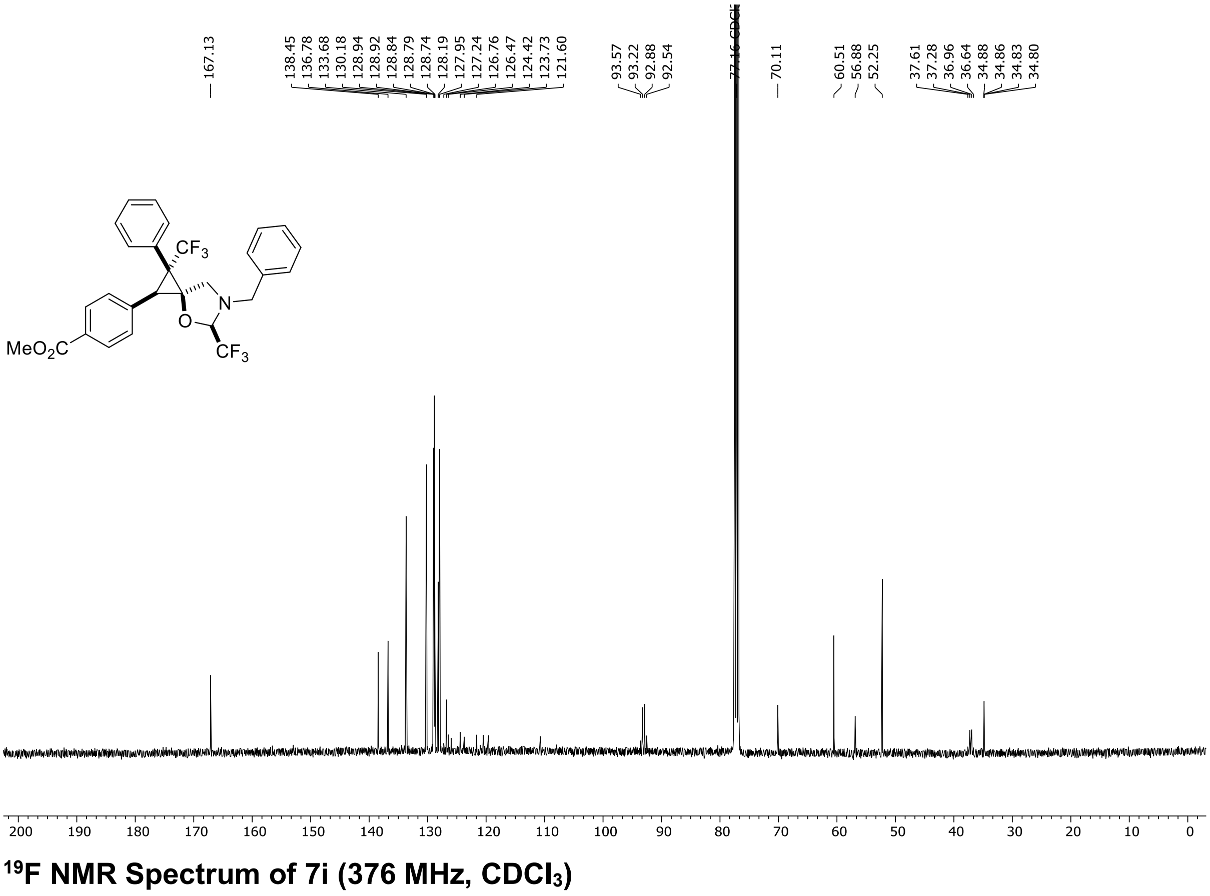

^{19}F NMR Spectrum of 7i (376 MHz, $CDCl_3$)

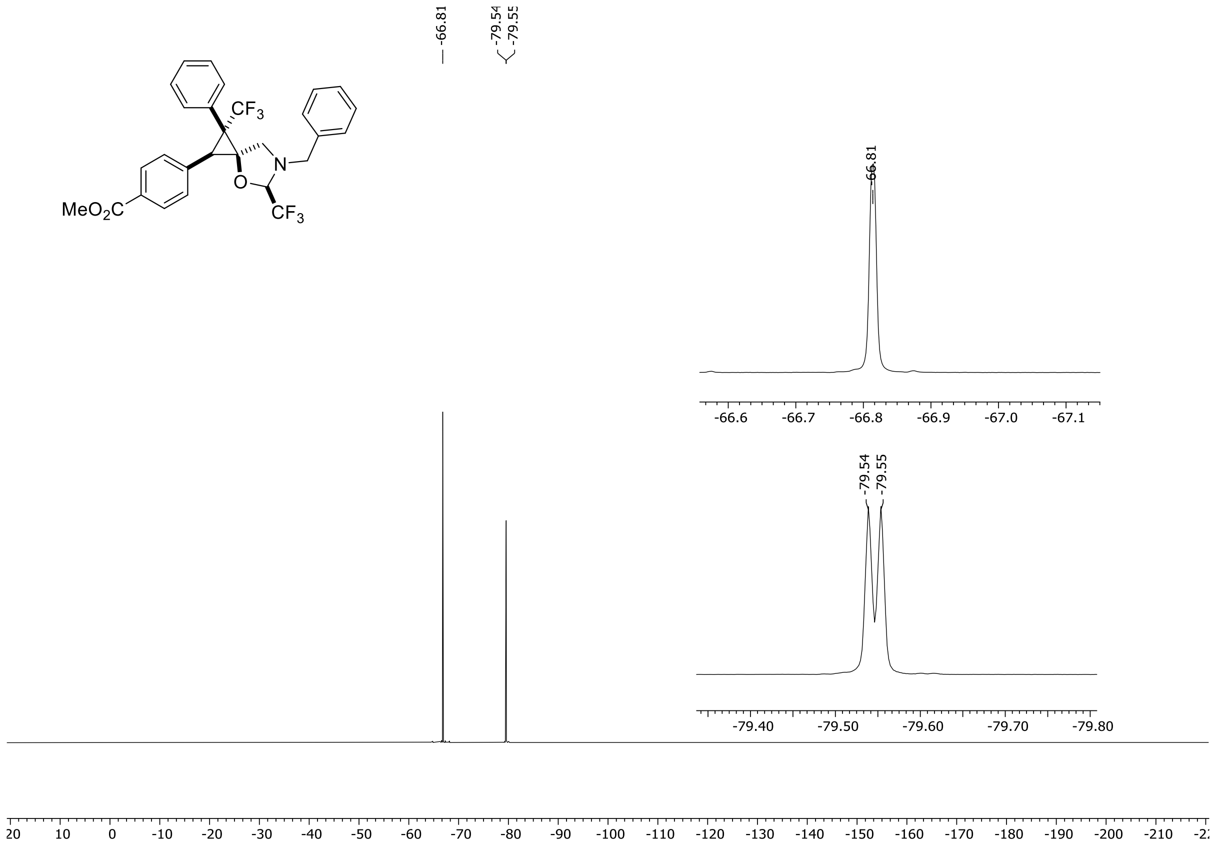

^1H NMR Spectrum of 7j (400 MHz, $CDCl_3$)

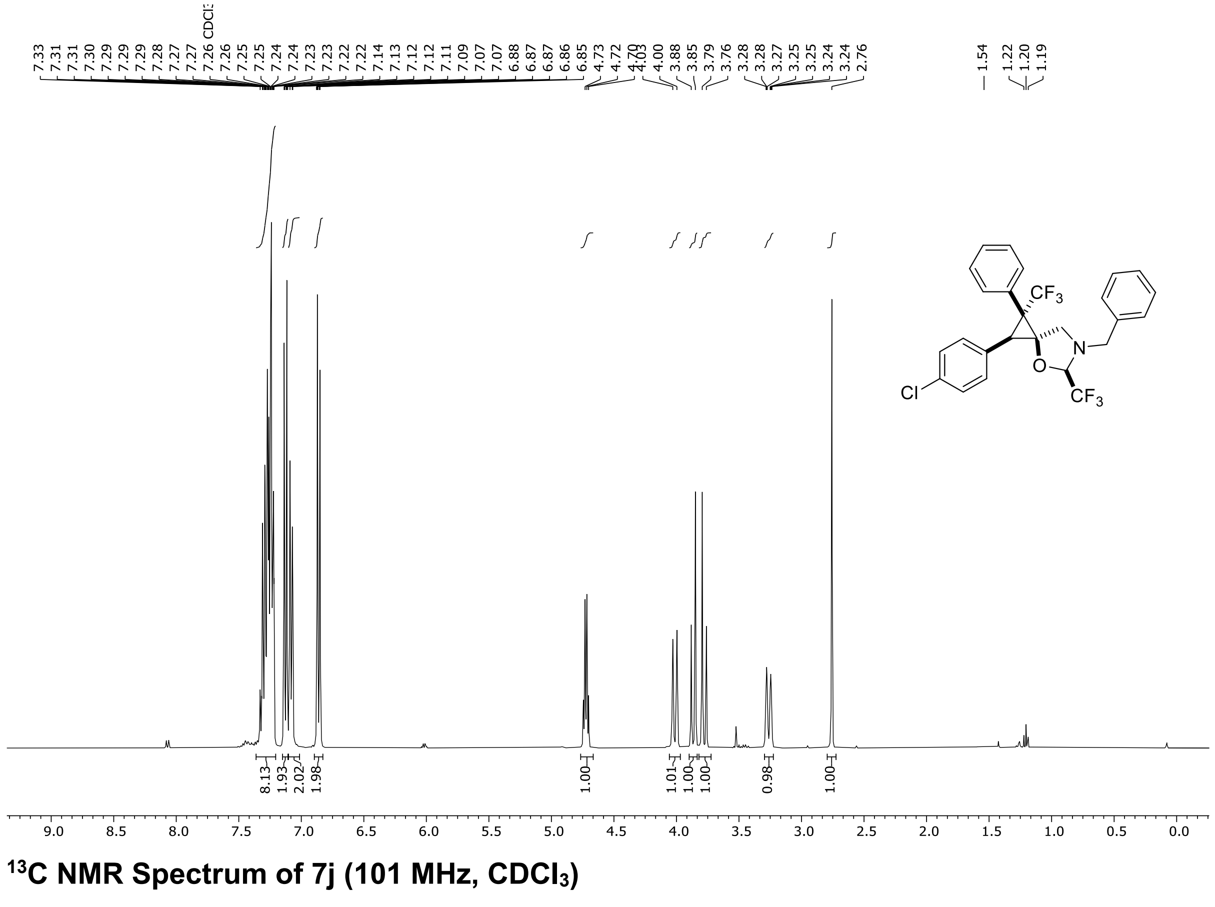

^{13}C NMR Spectrum of 7j (101 MHz, $CDCl_3$)

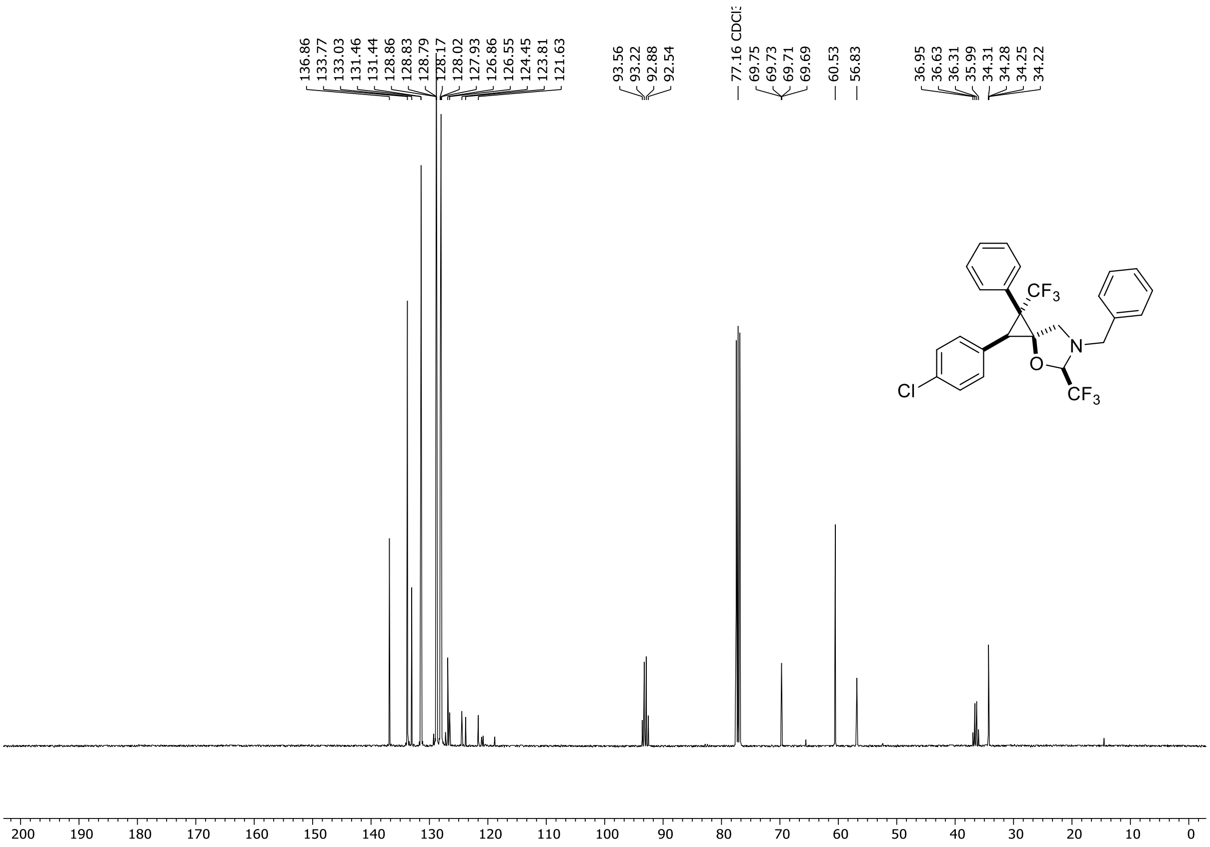

^{19}F NMR Spectrum of 7j (376 MHz, $CDCl_3$)

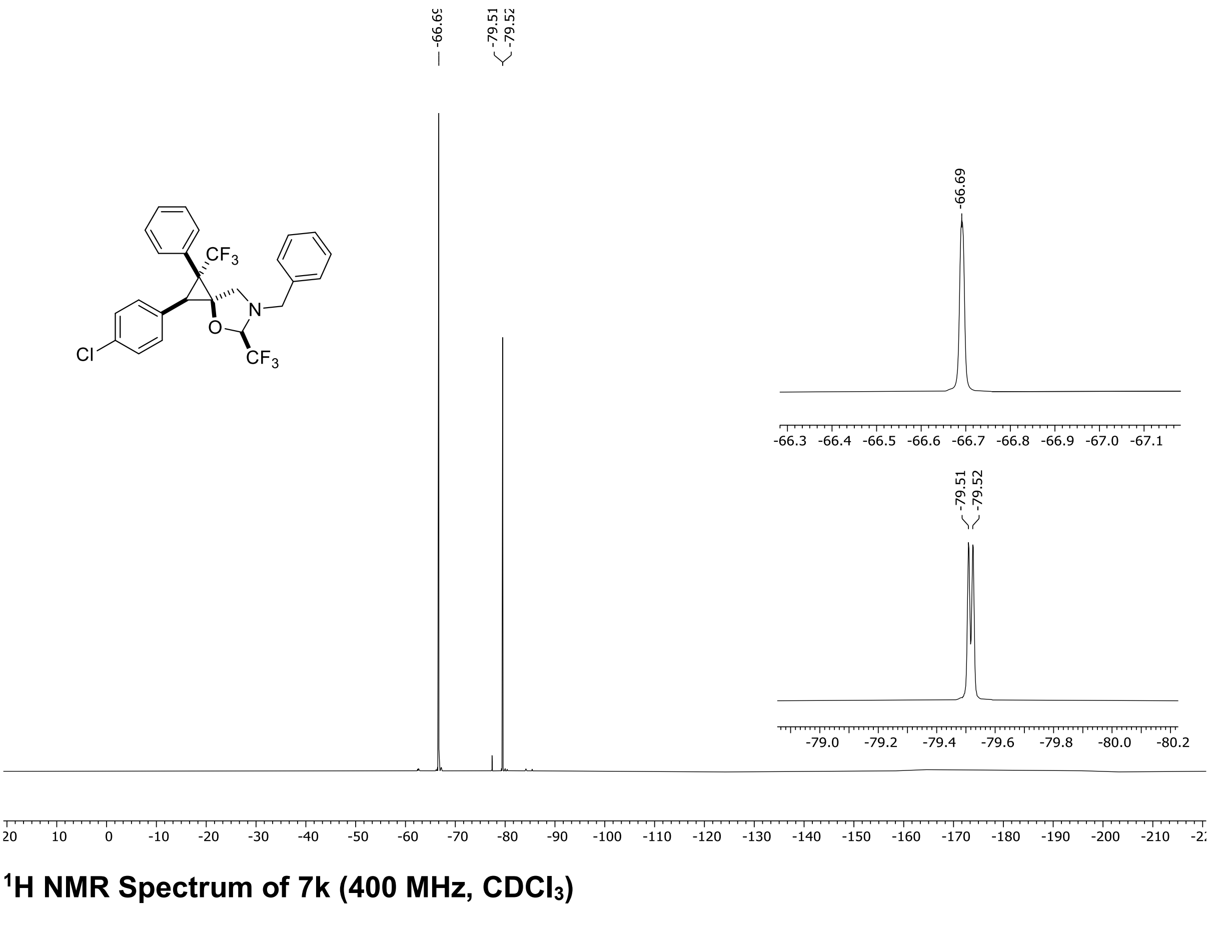

^{1}H NMR Spectrum of 7k (400 MHz, $CDCl_3$)

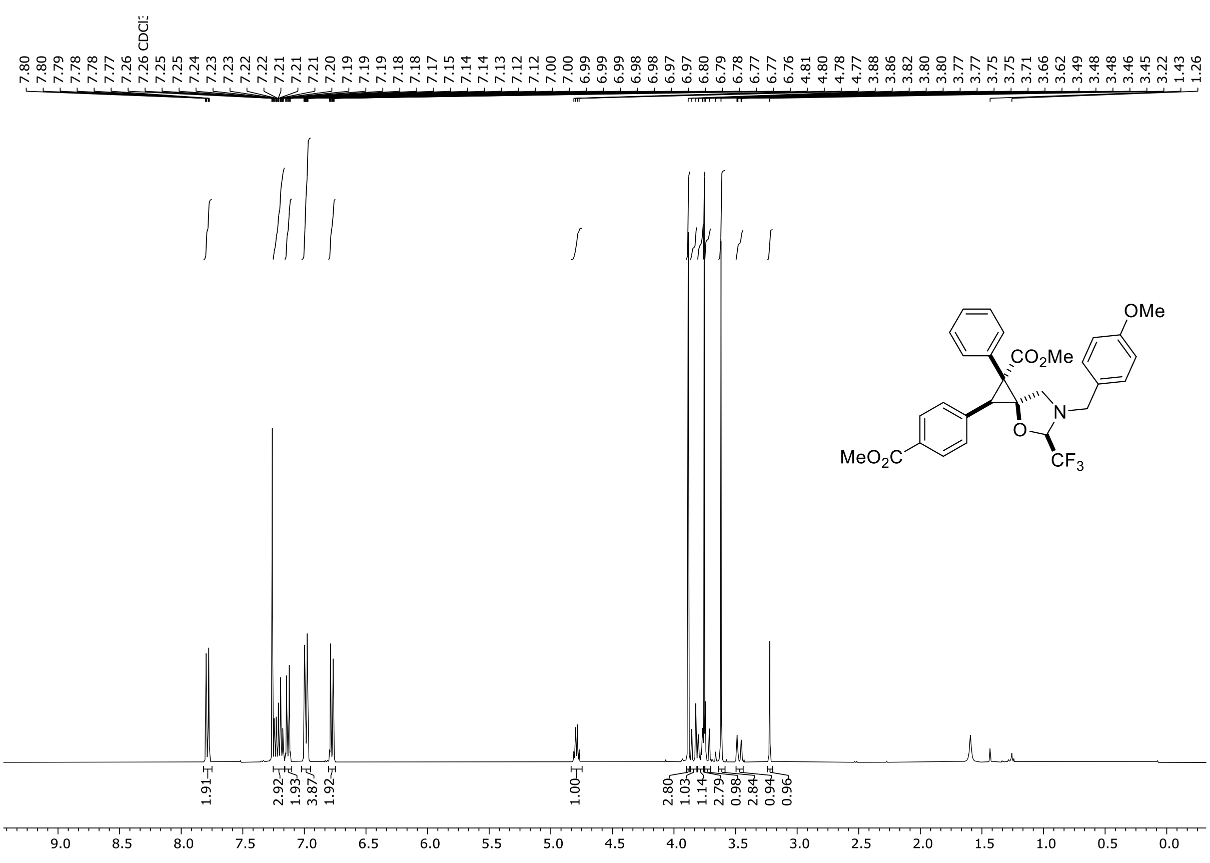

^{13}C NMR Spectrum of 7k (101 MHz, $CDCl_3$)

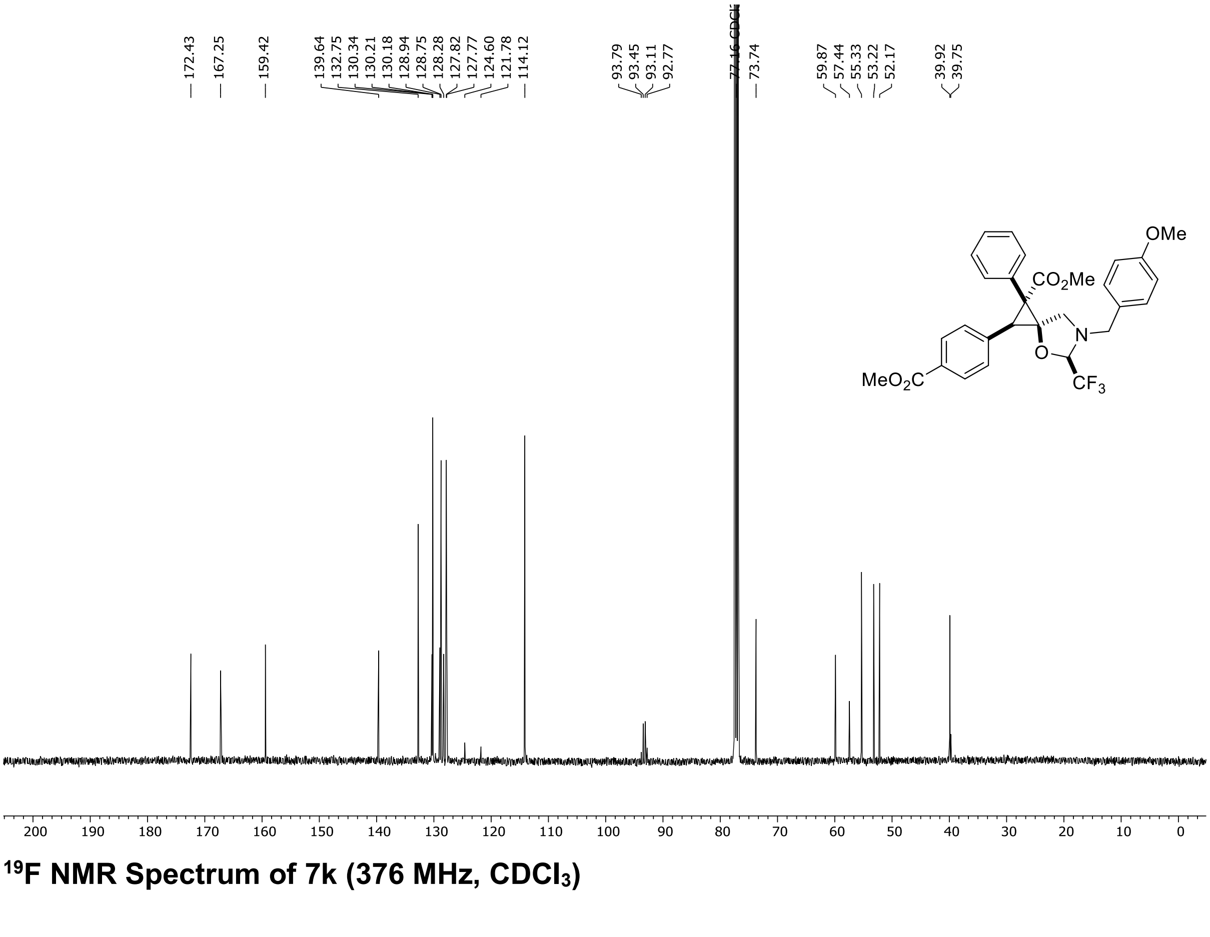

^{19}F NMR Spectrum of 7k (376 MHz, $CDCl_3$)

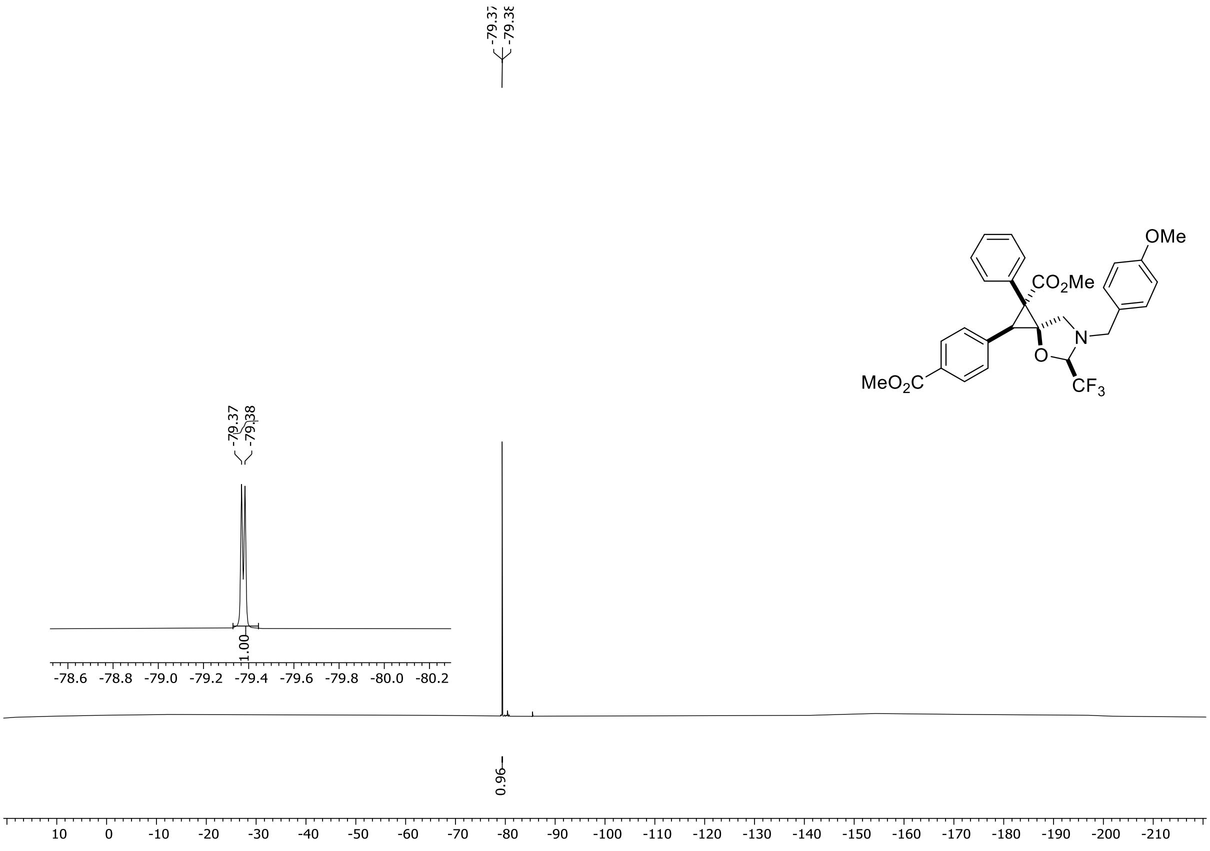

^{1}H NMR Spectrum of 7l (400 MHz, $CDCl_3$)

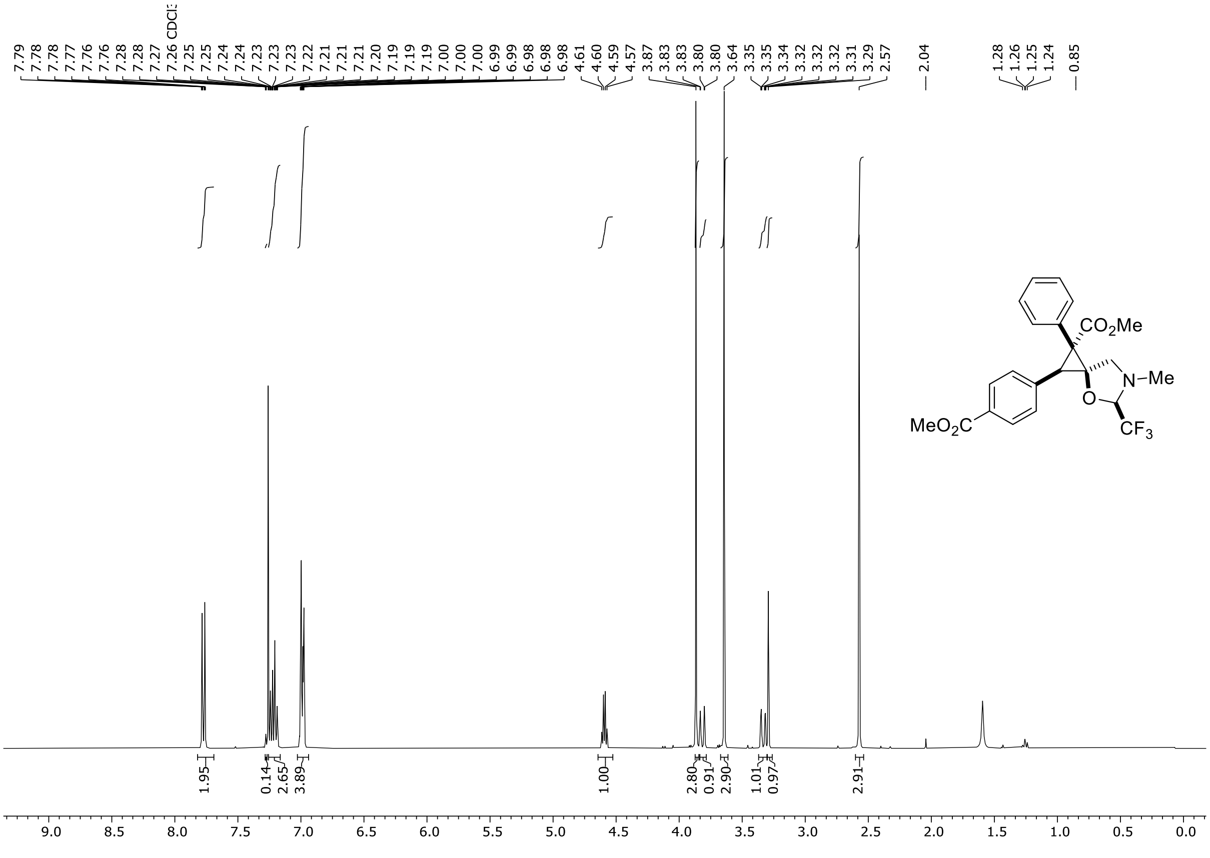

^{13}C NMR Spectrum of 7l (101 MHz, $CDCl_3$)

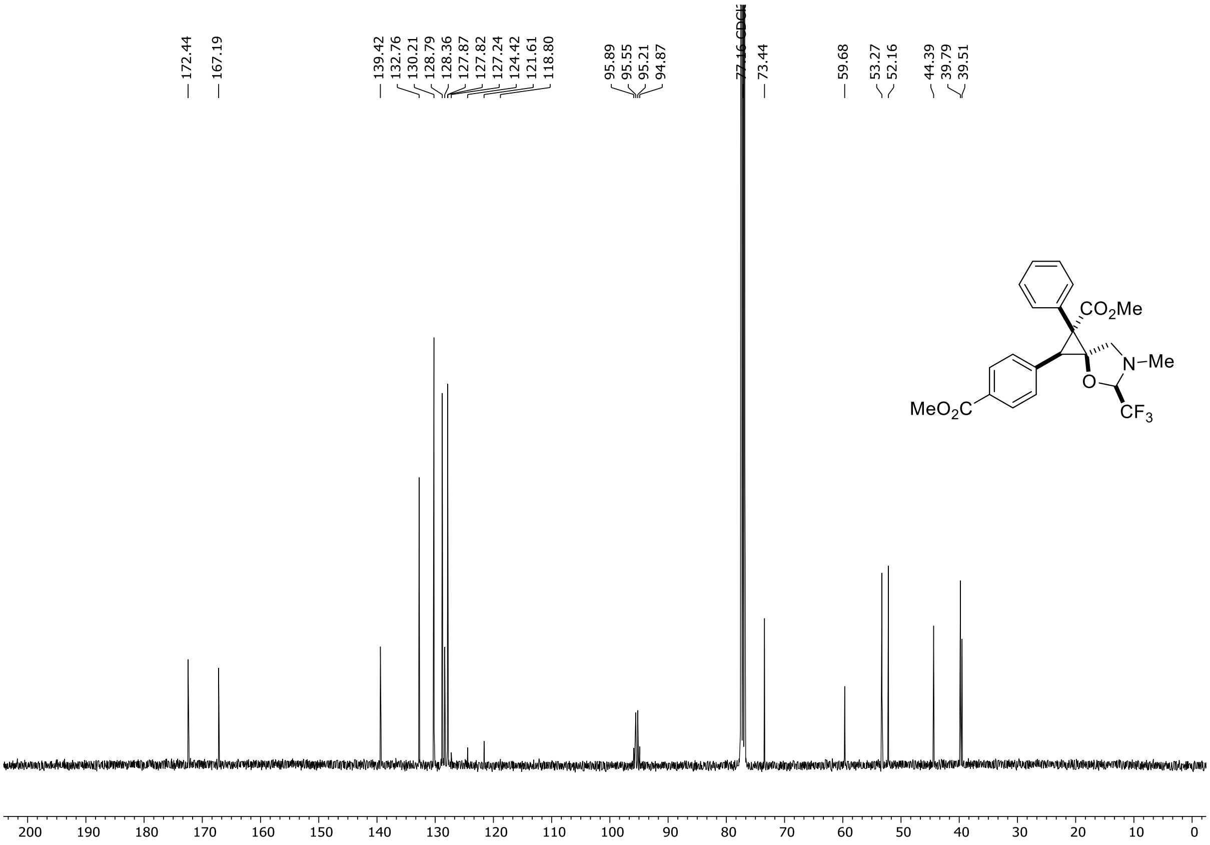

^{19}F NMR Spectrum of 7l (376 MHz, $CDCl_3$)

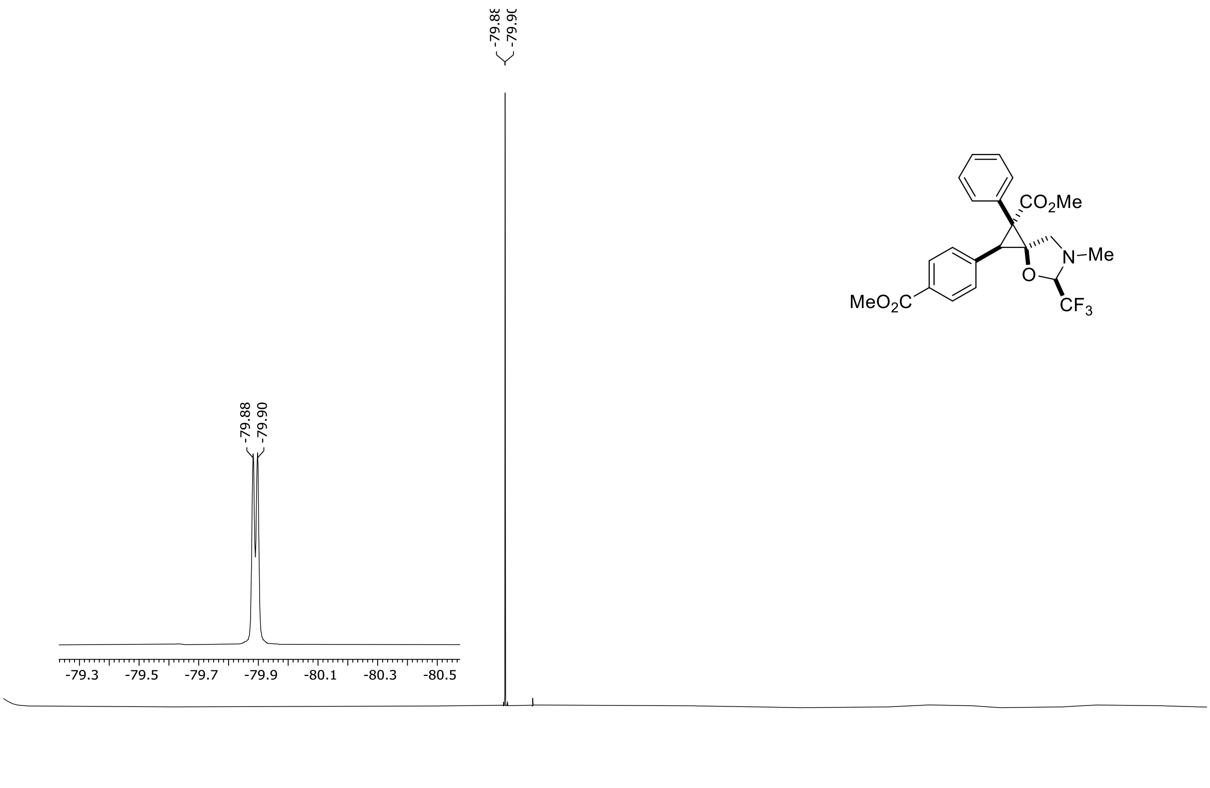

^{1}H NMR Spectrum of 7m (400 MHz, $CDCl_3$)

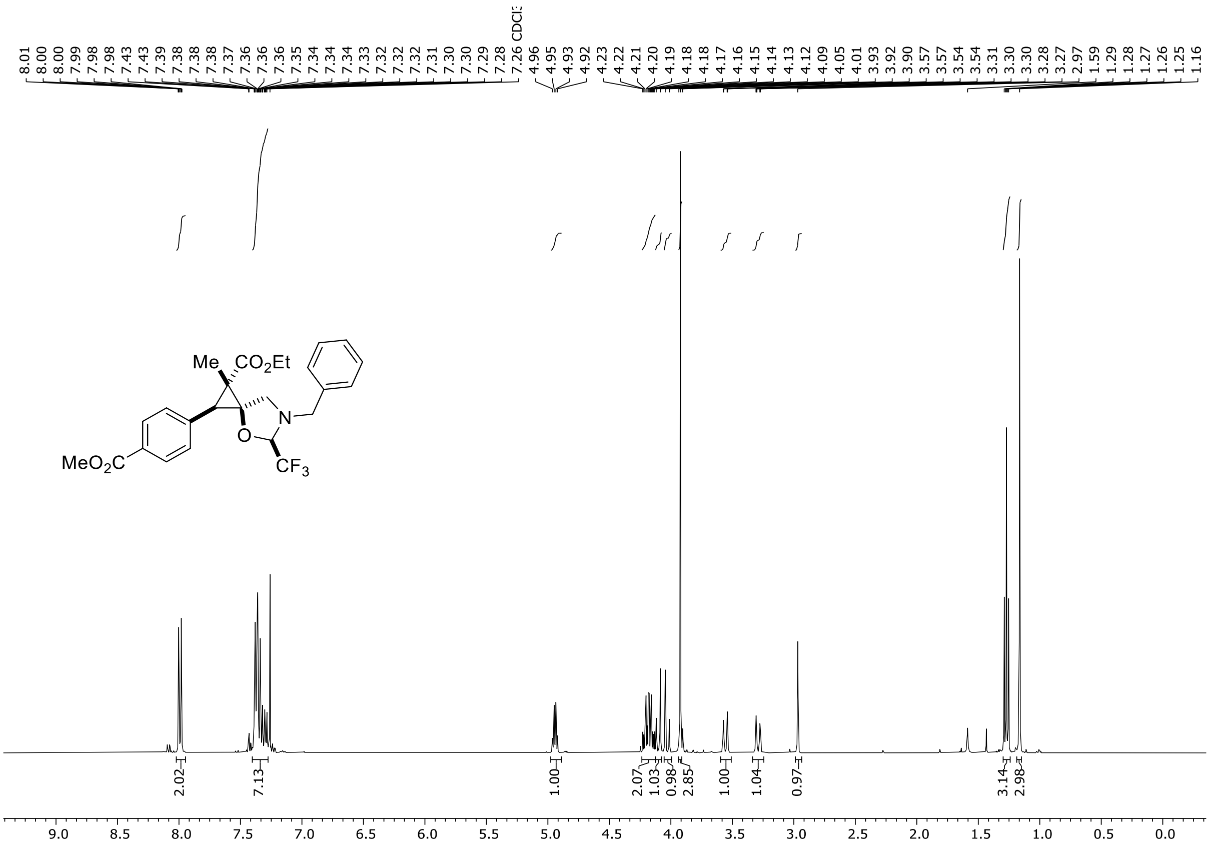

^{13}C NMR Spectrum of 7m (101 MHz, $CDCl_3$)

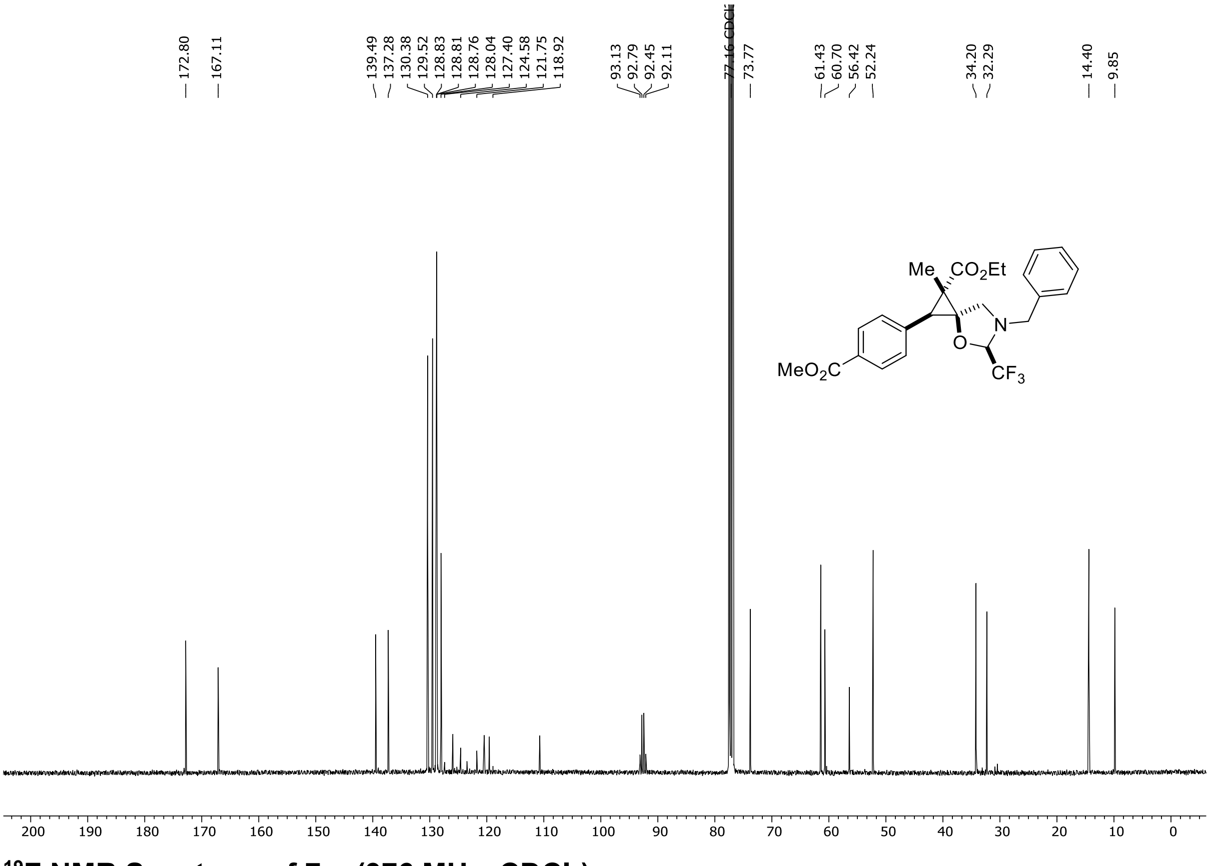

^{19}F NMR Spectrum of 7m (376 MHz, $CDCl_3$)

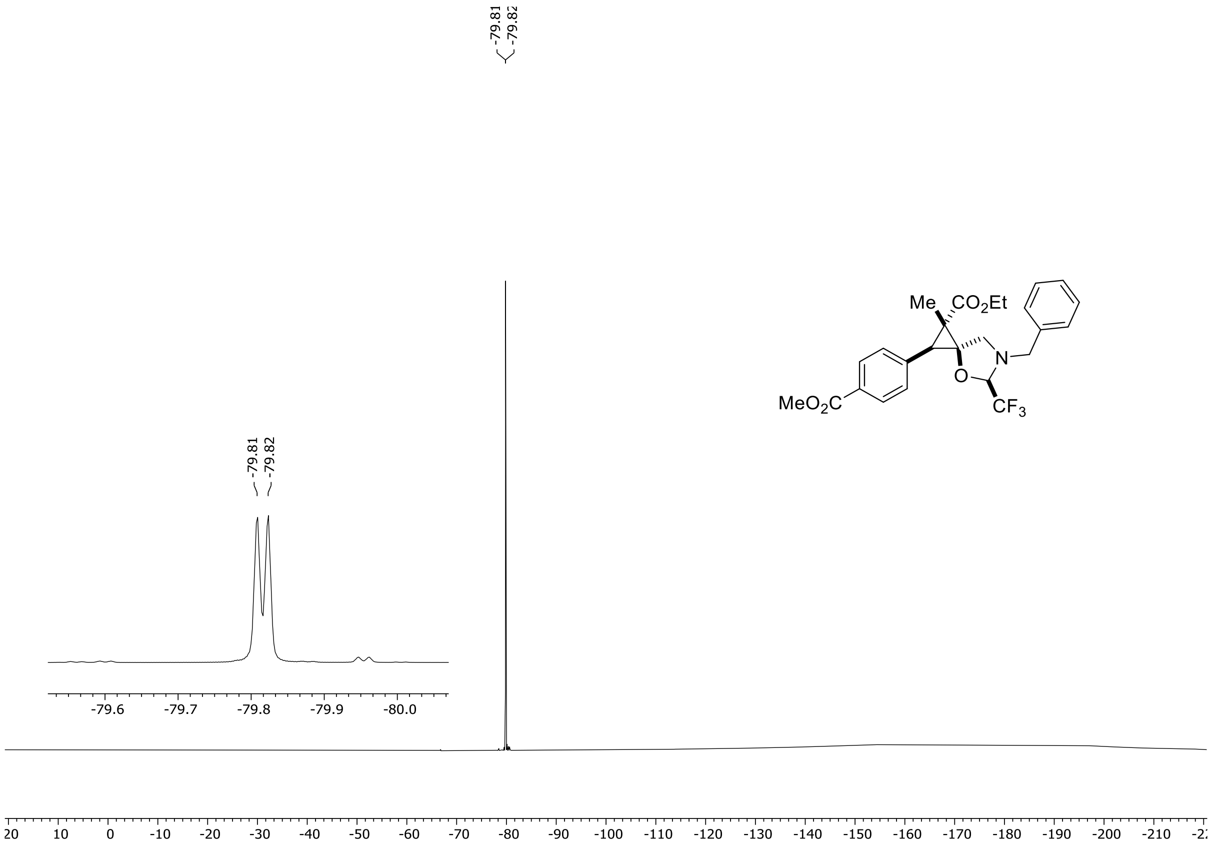

^{1}H NMR Spectrum of 7n (400 MHz, $CDCl_3$)

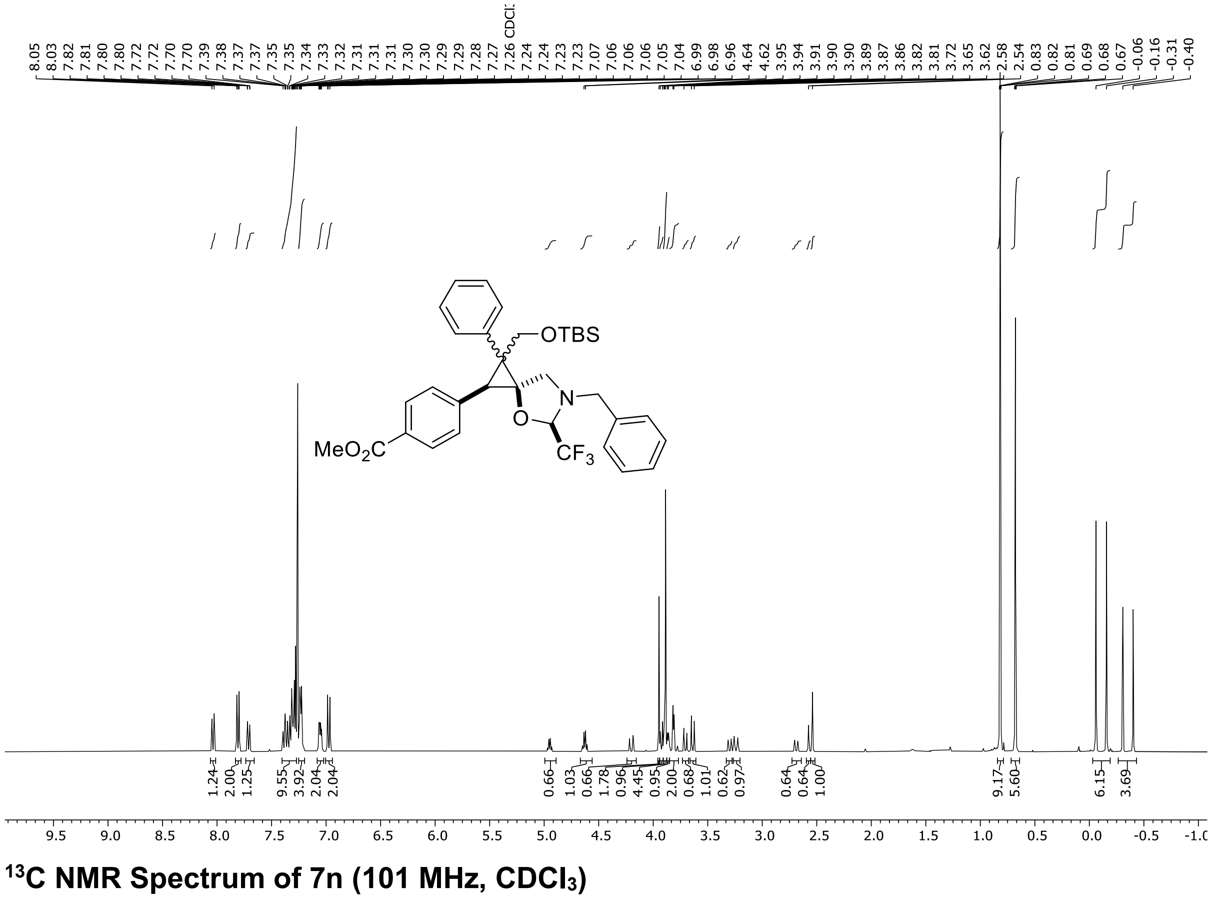

^{13}C NMR Spectrum of 7n (101 MHz, $CDCl_3$)

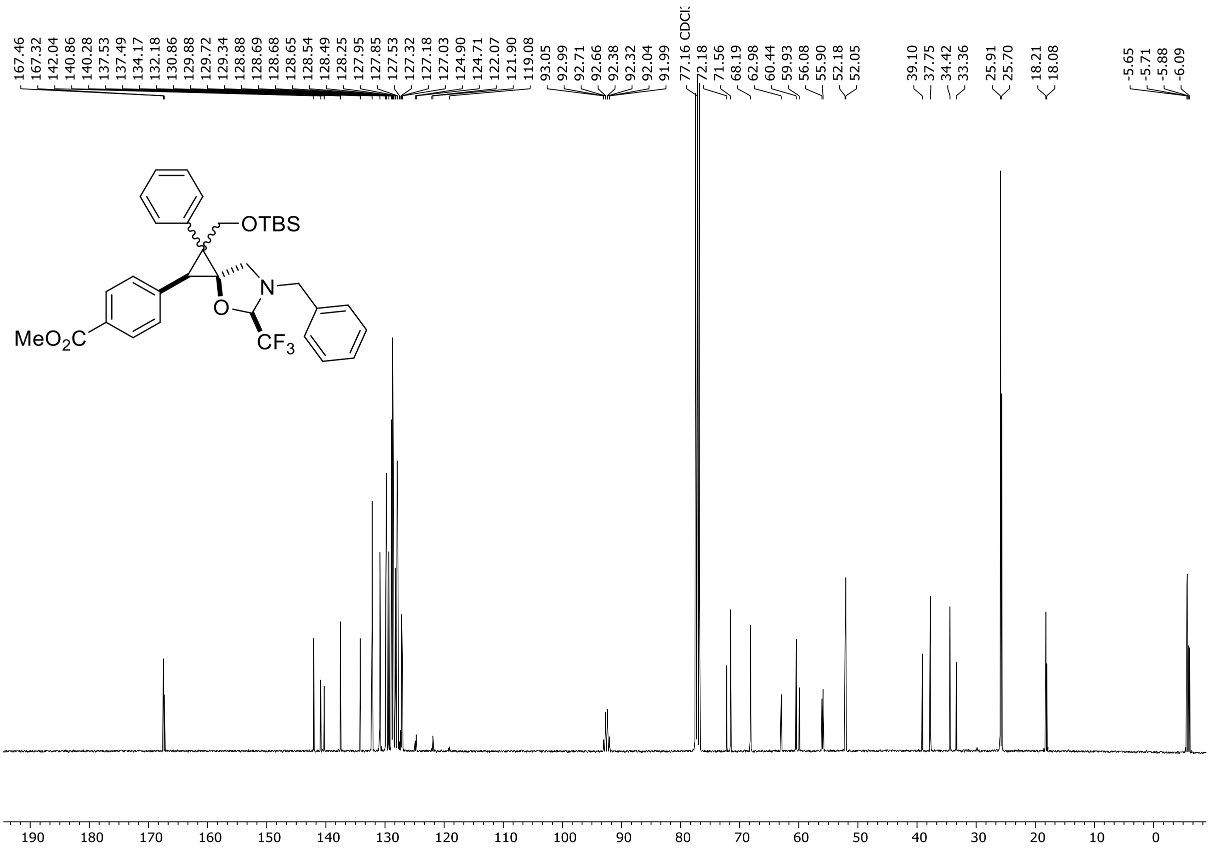

^{19}F NMR Spectrum of 7n (376 MHz, $CDCl_3$)

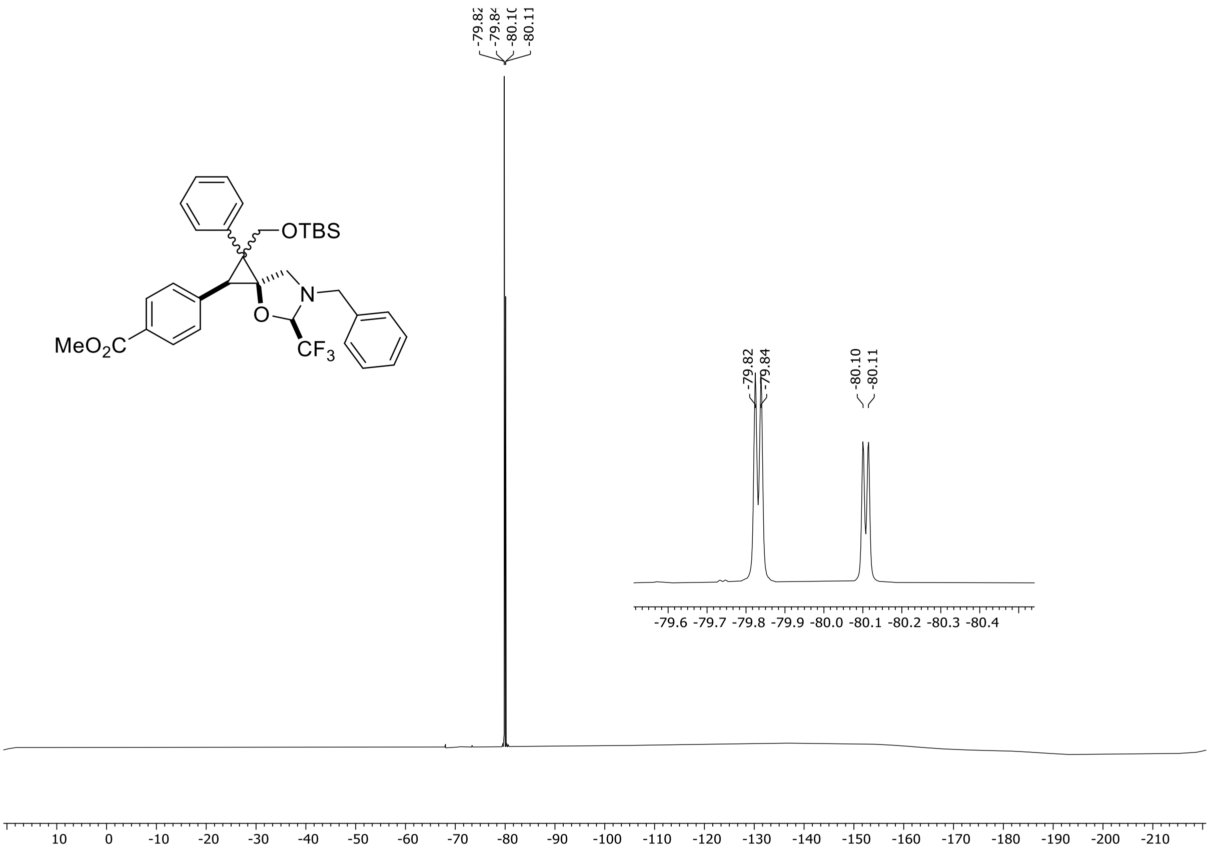

^{1}H NMR Spectrum of 9a (400 MHz, $CDCl_3$)

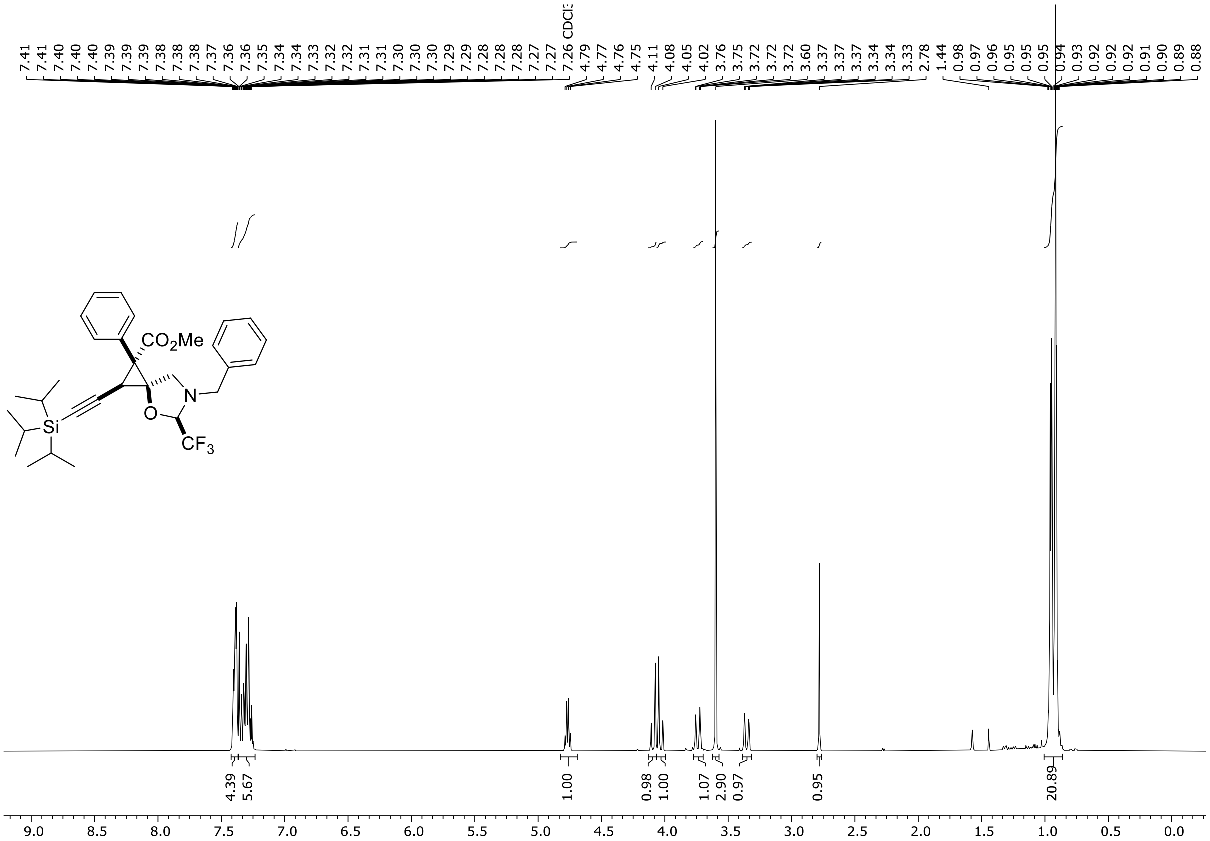

^{13}C NMR Spectrum of 9a (101 MHz, $CDCl_3$)

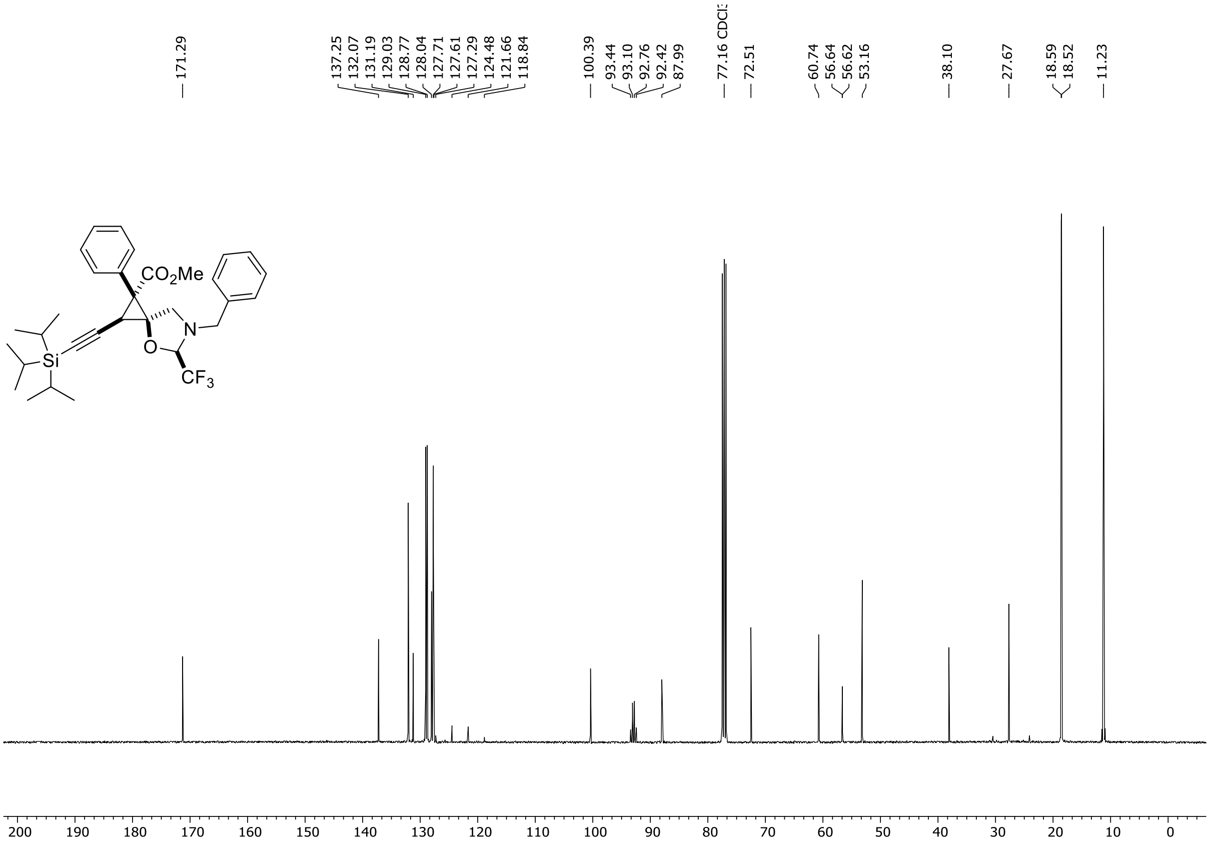

^{19}F NMR Spectrum of 9a (376 MHz, $CDCl_3$)

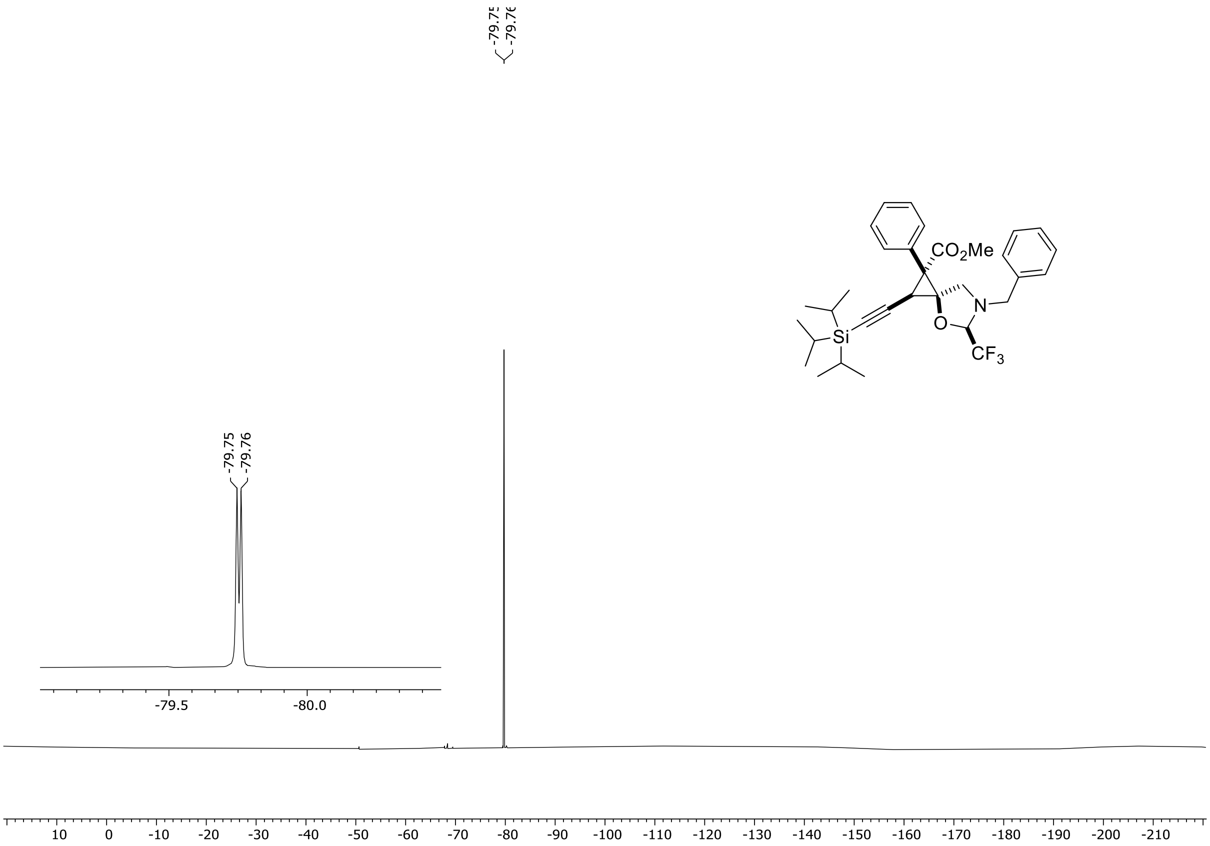

^{1}H NMR Spectrum of 11a (400 MHz, $CDCl_3$)

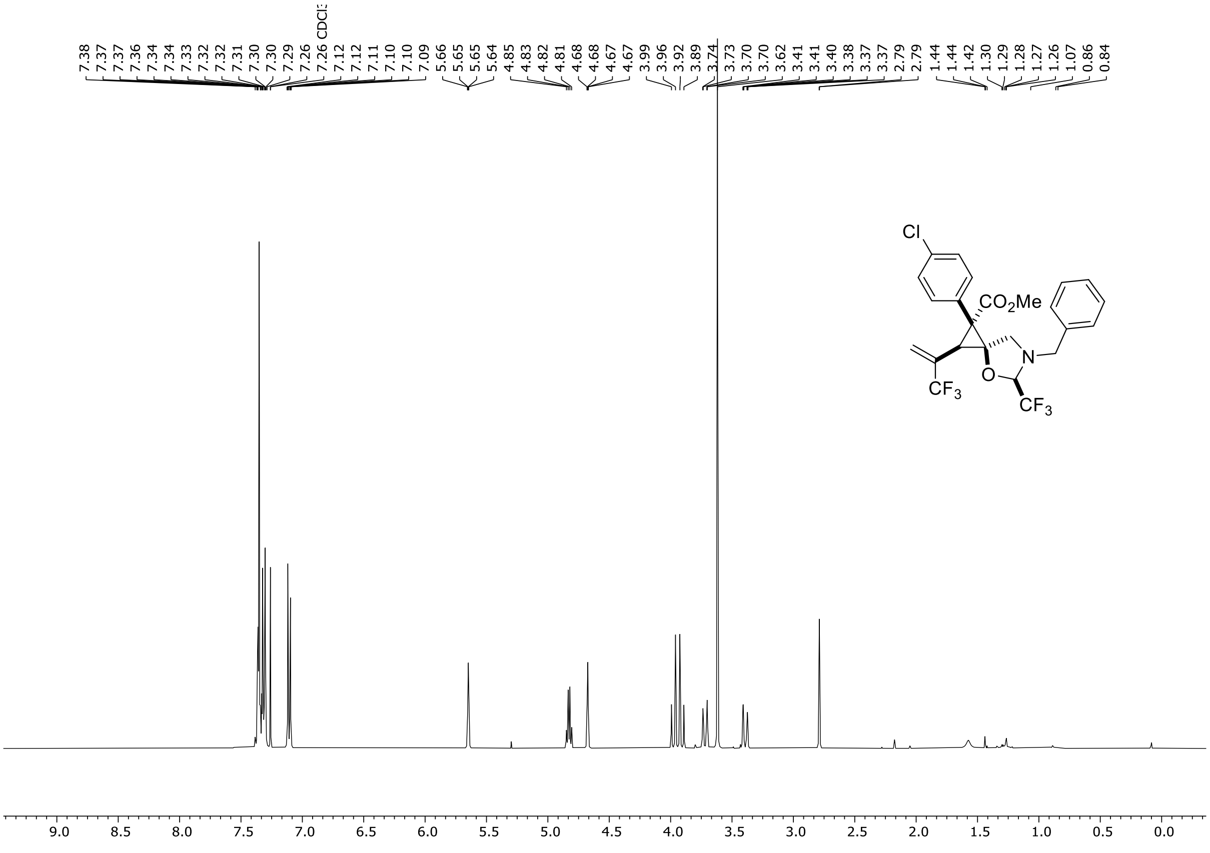

^{13}C NMR Spectrum of 11a (101 MHz, $CDCl_3$)

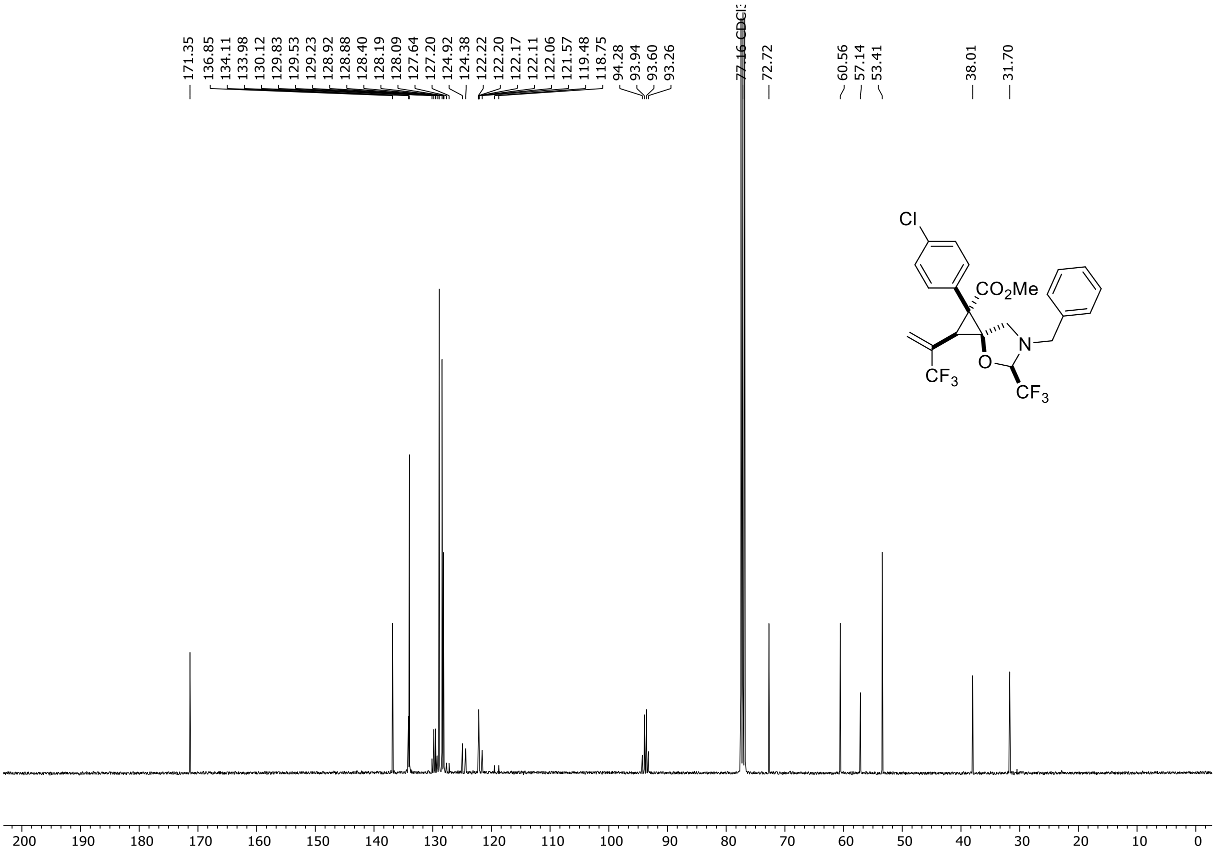

^{19}F NMR Spectrum of 11a (376 MHz, $CDCl_3$)

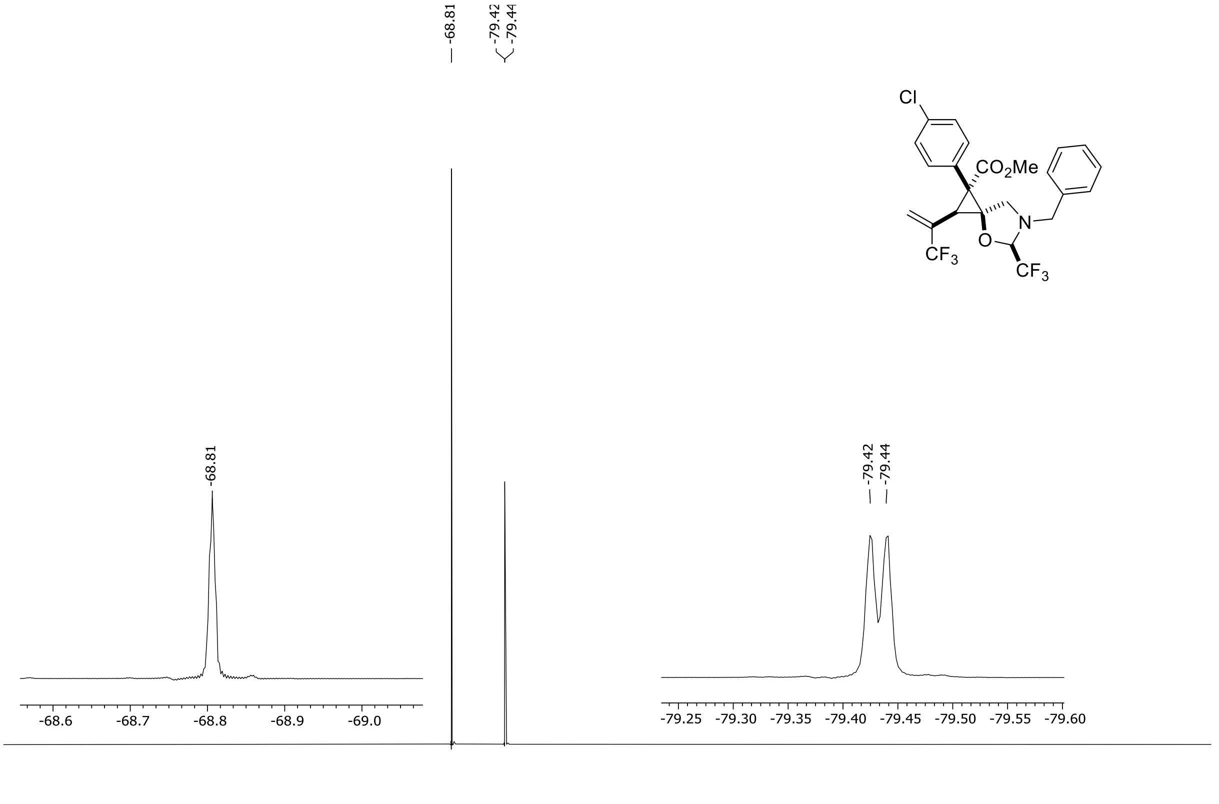

^{1}H NMR Spectrum of 11b (400 MHz, $CDCl_3$)

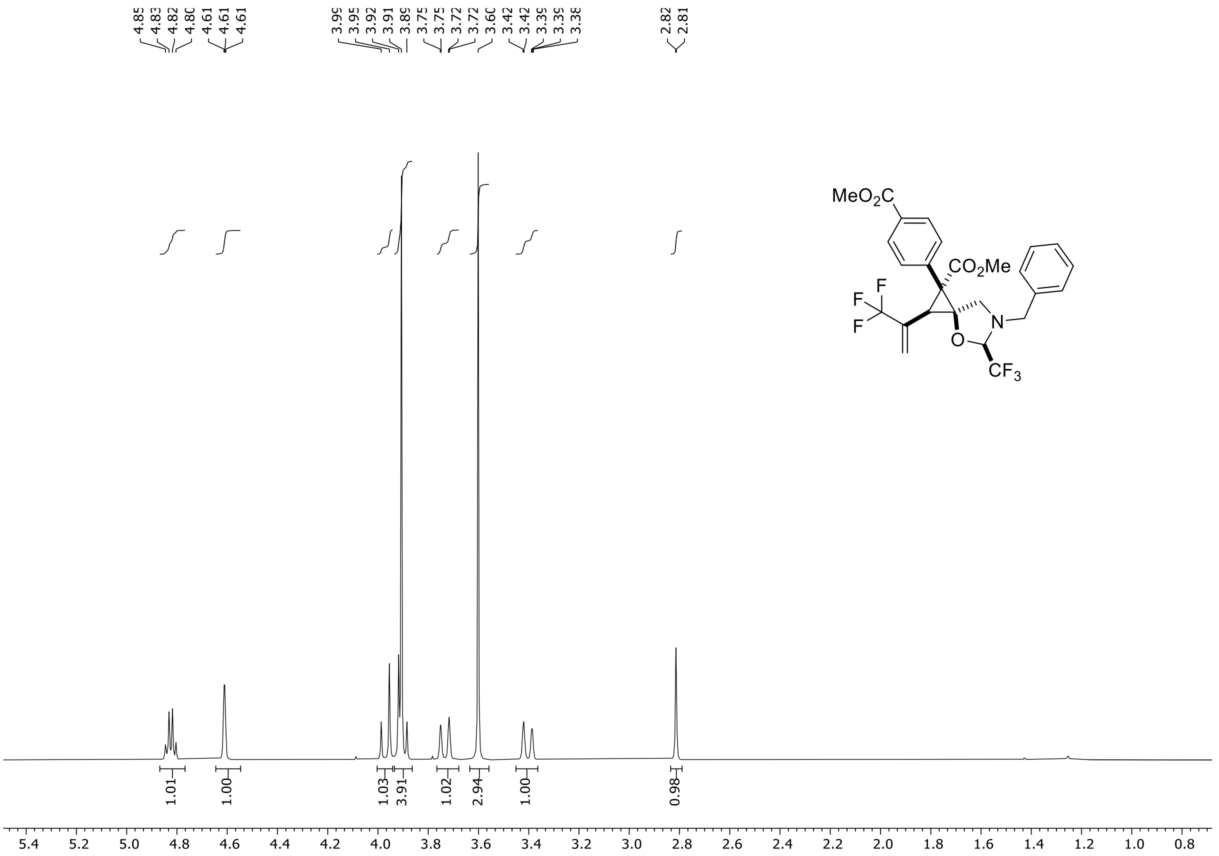

^{13}C NMR Spectrum of 11b (101 MHz, $CDCl_3$)

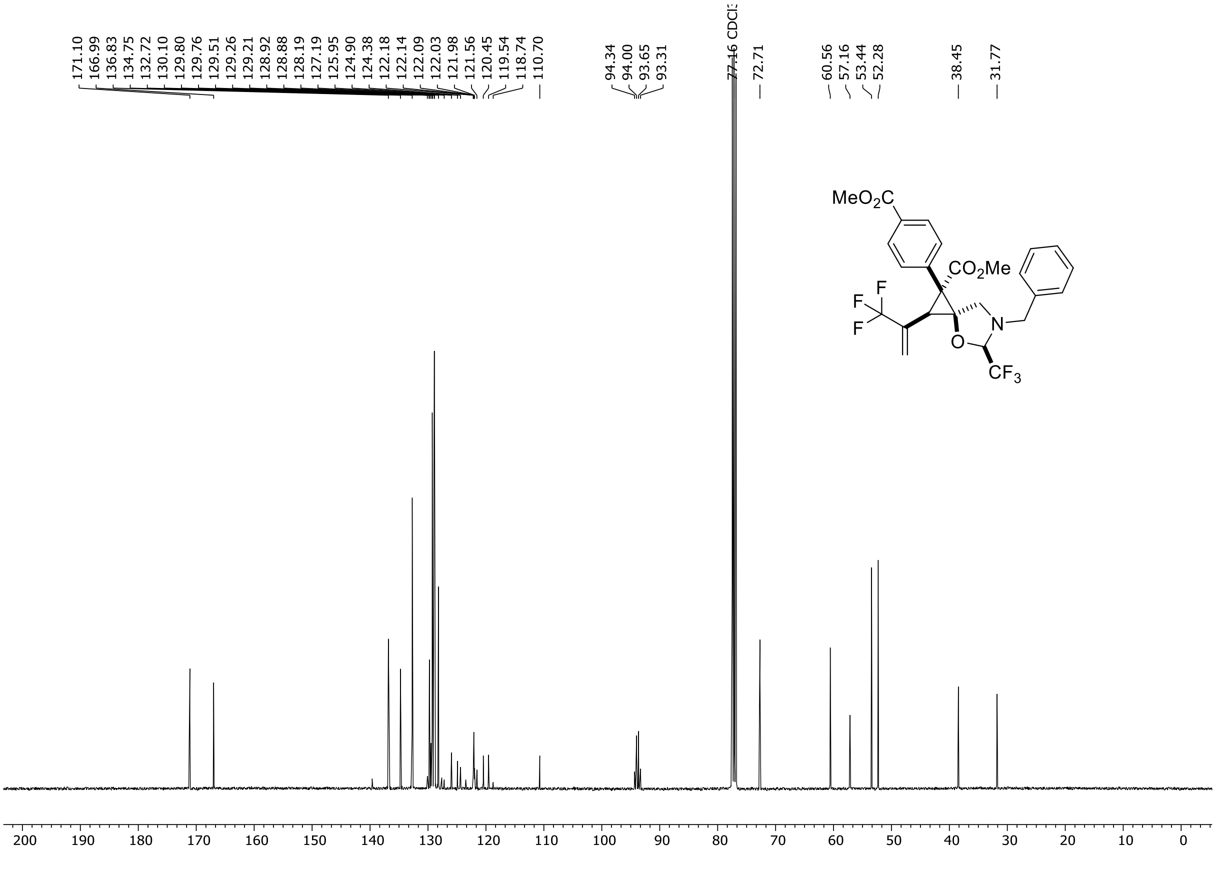

^{19}F NMR Spectrum of 11b (376 MHz, $CDCl_3$)

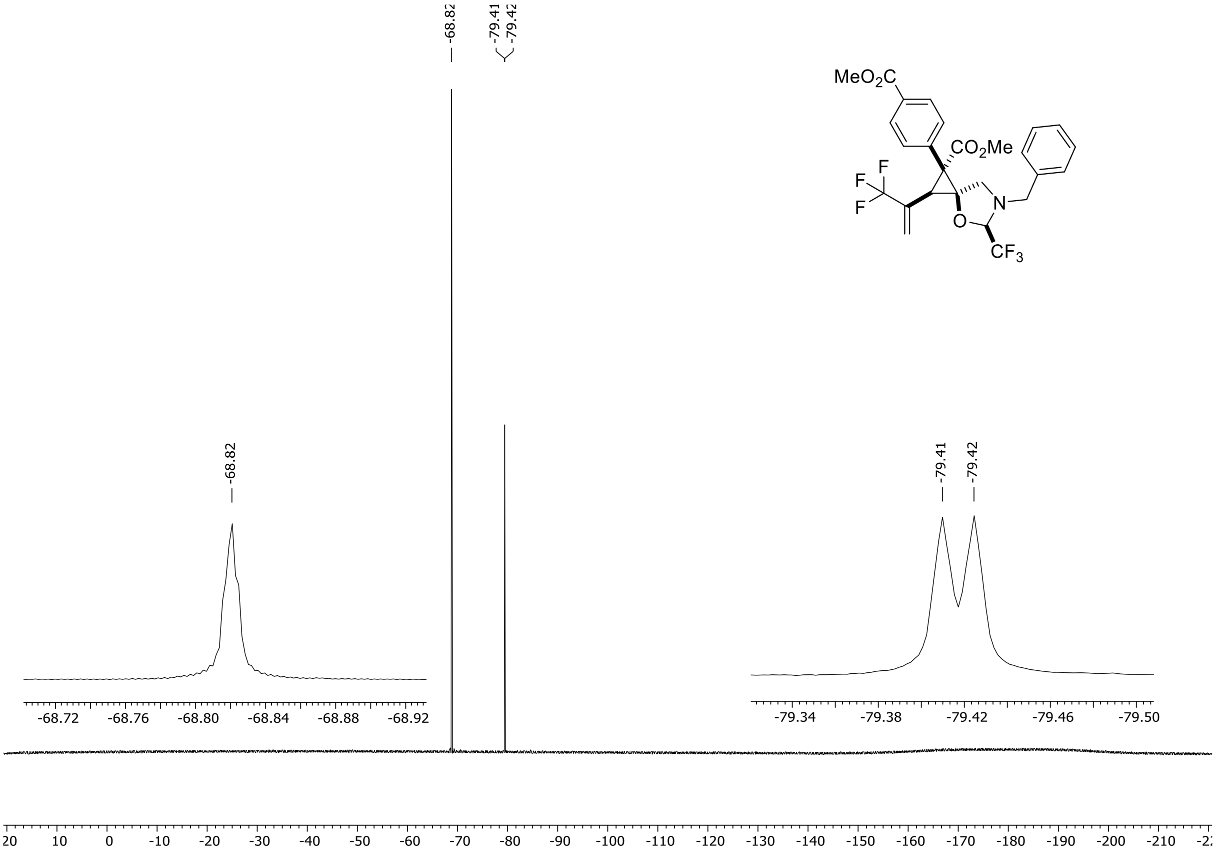

Product Modifications

^{1}H NMR Spectrum of 12 (400 MHz, $CDCl_3$)

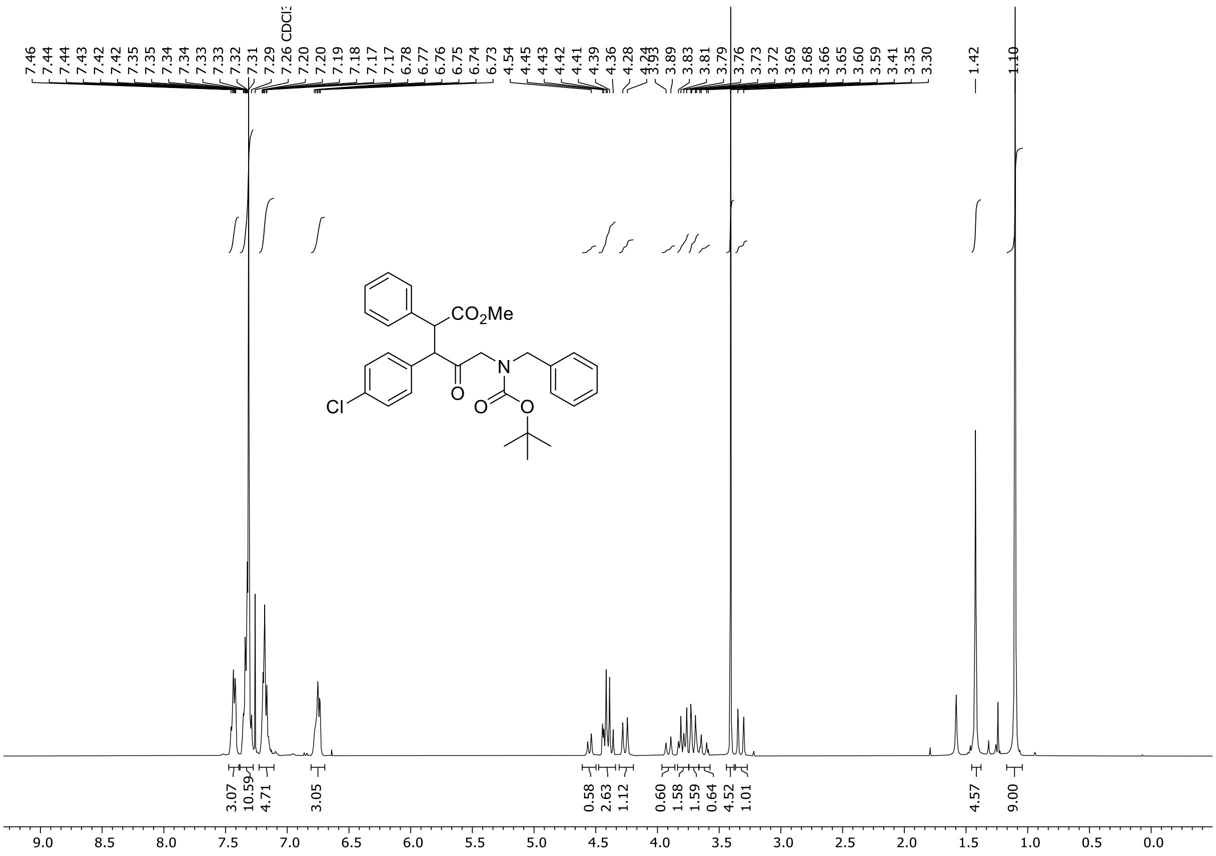

^{13}C NMR Spectrum of 12 (101 MHz, $CDCl_3$)

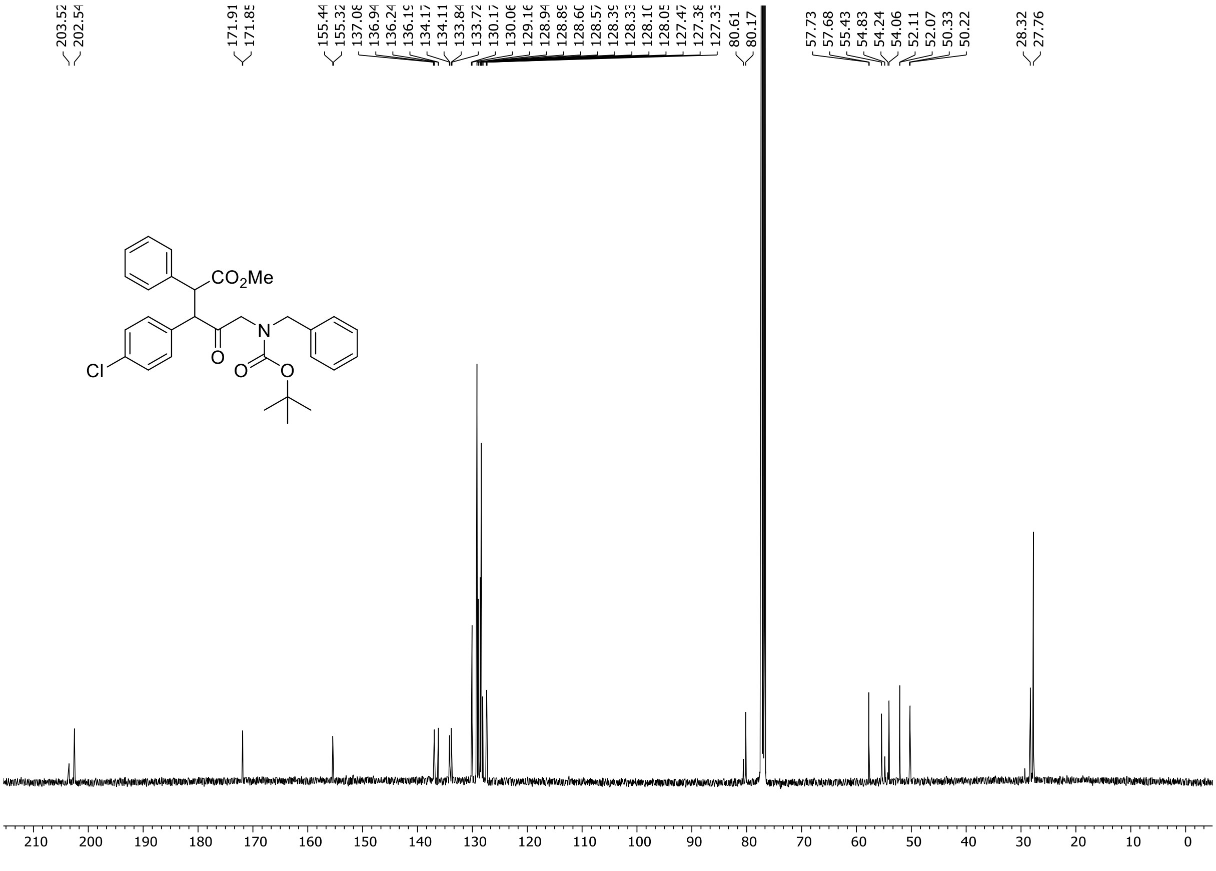

^{1}H NMR Spectrum of 13 (400 MHz, $CDCl_3$)

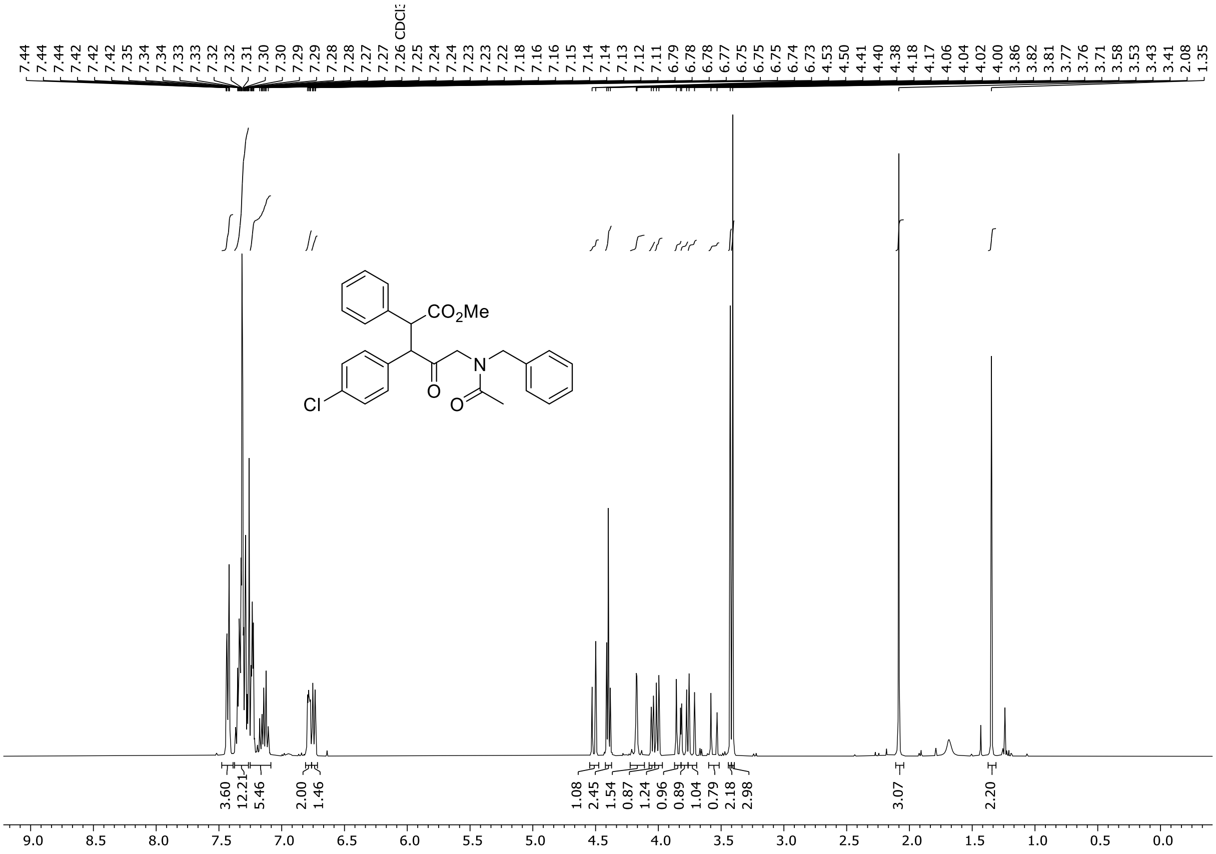

^{13}C NMR Spectrum of 13 (101 MHz, $CDCl_3$)

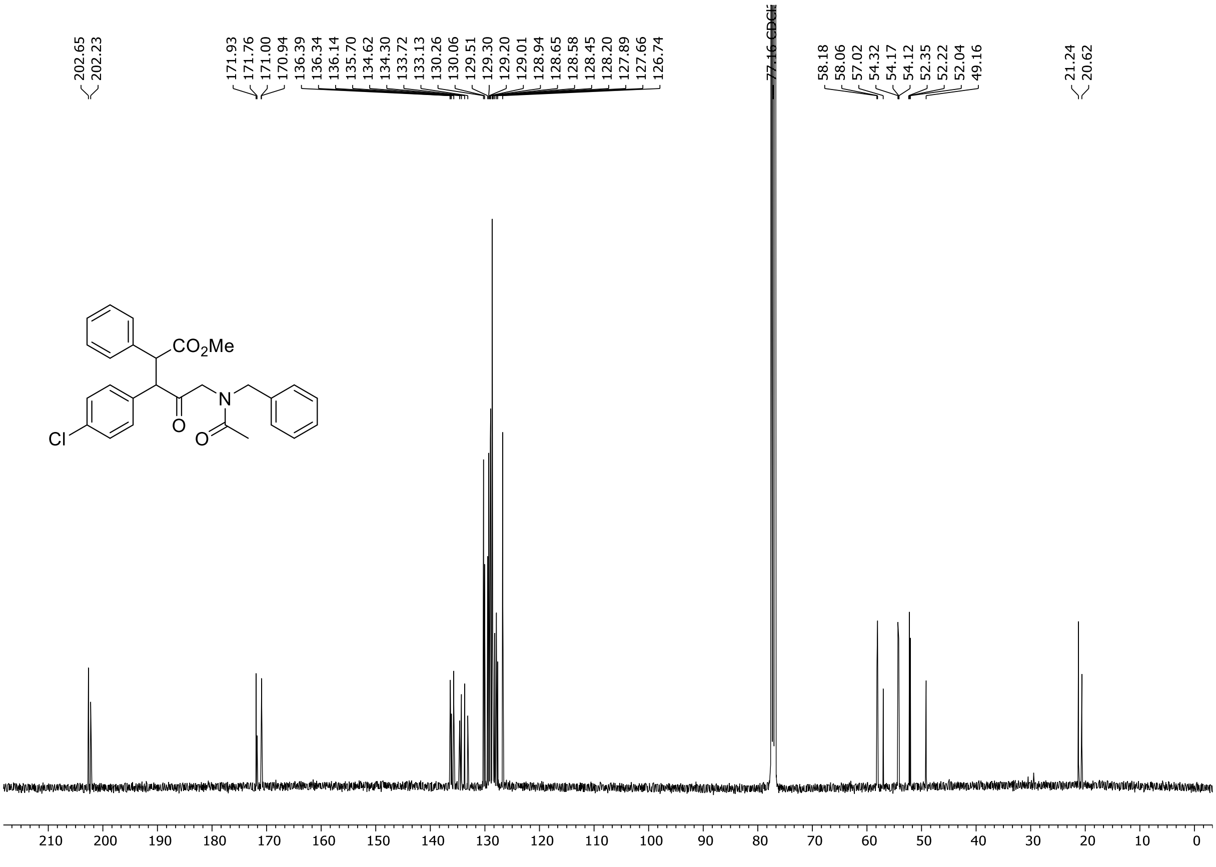

^{1}H NMR Spectrum of 14 (400 MHz, $CDCl_3$)

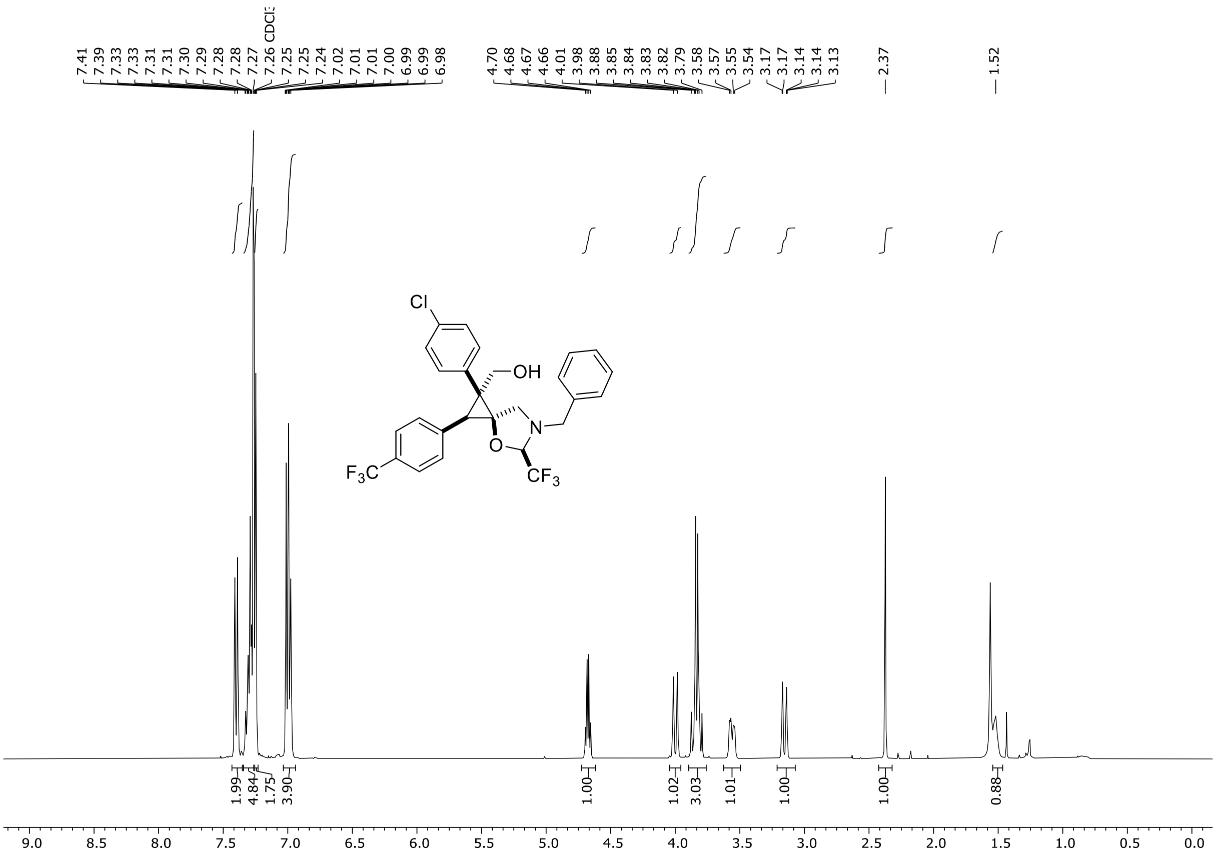

^{13}C NMR Spectrum of 14 (101 MHz, $CDCl_3$)

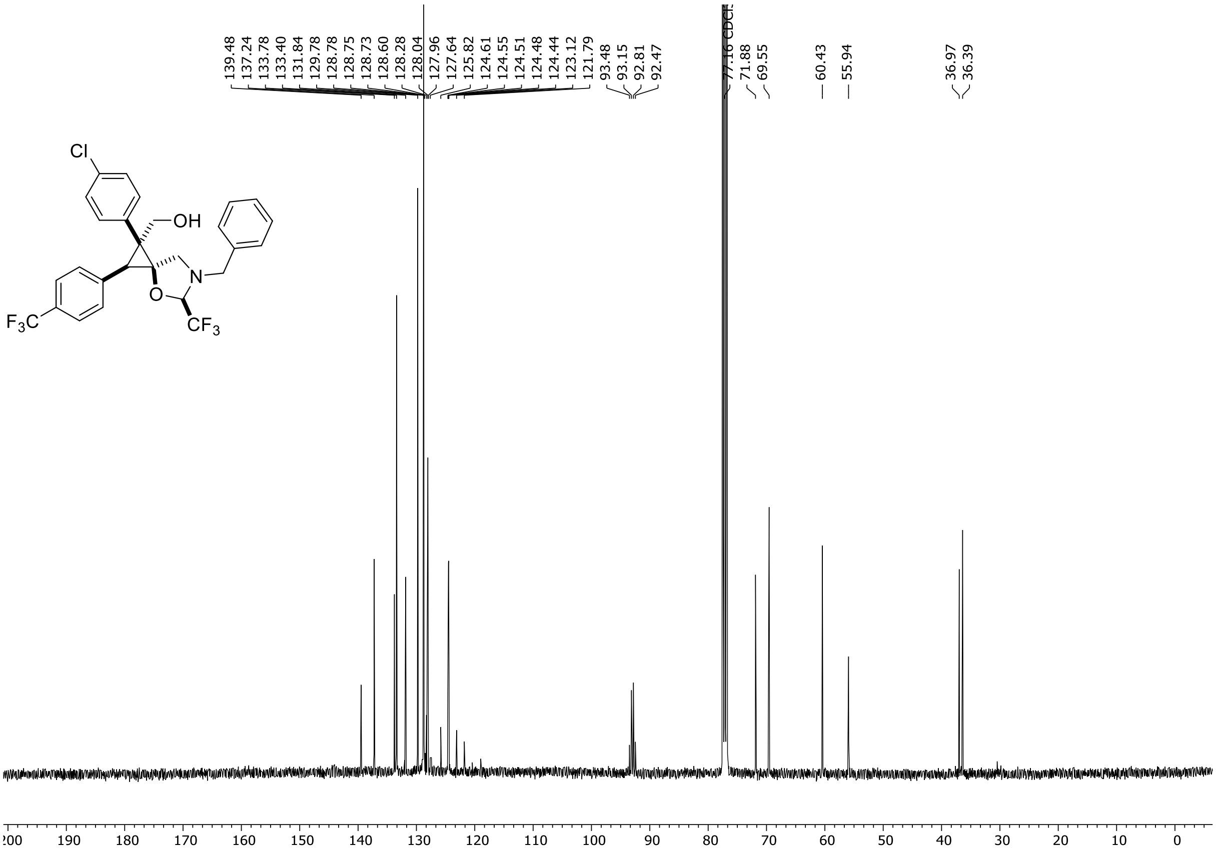

^{19}F NMR Spectrum of 14 (376 MHz, CDCl$_3$)

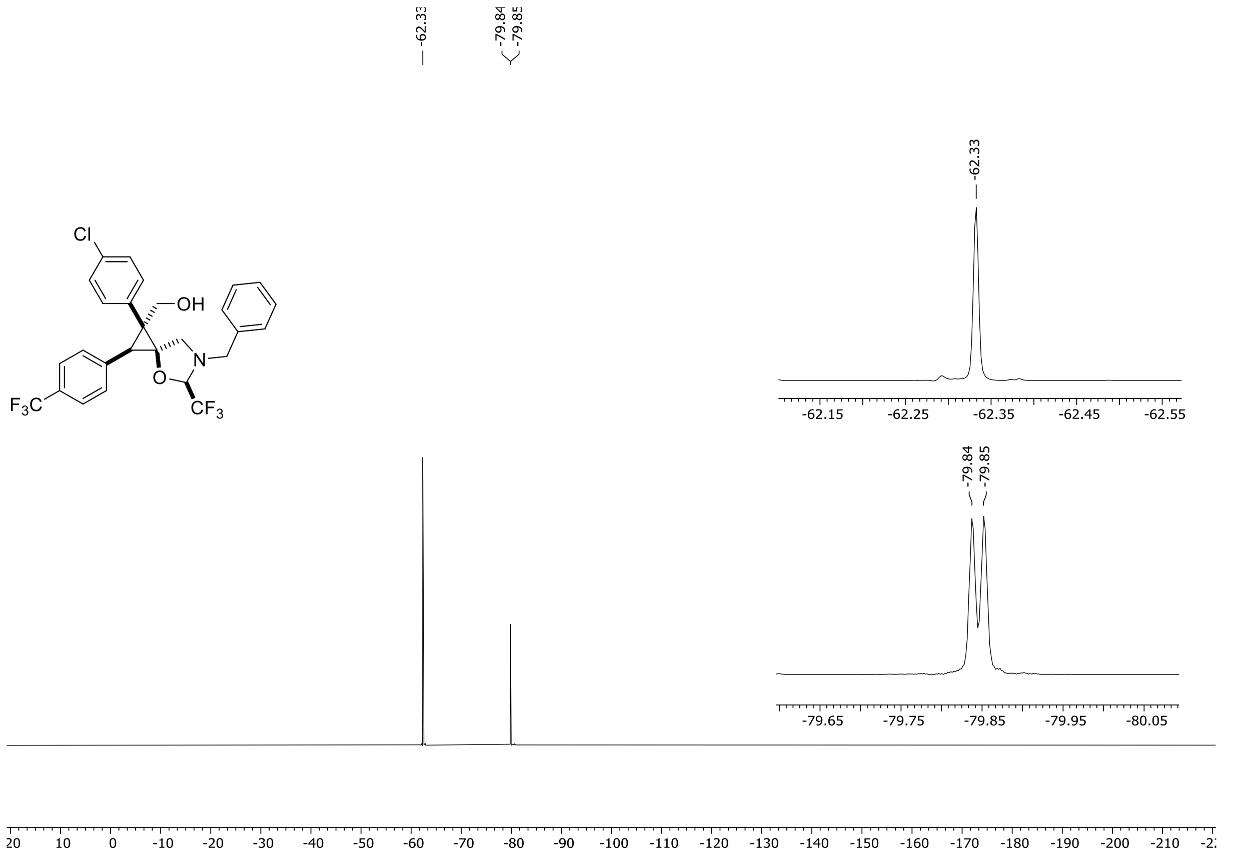

^{1}H NMR Spectrum of 15 (400 MHz, CDCl$_3$)

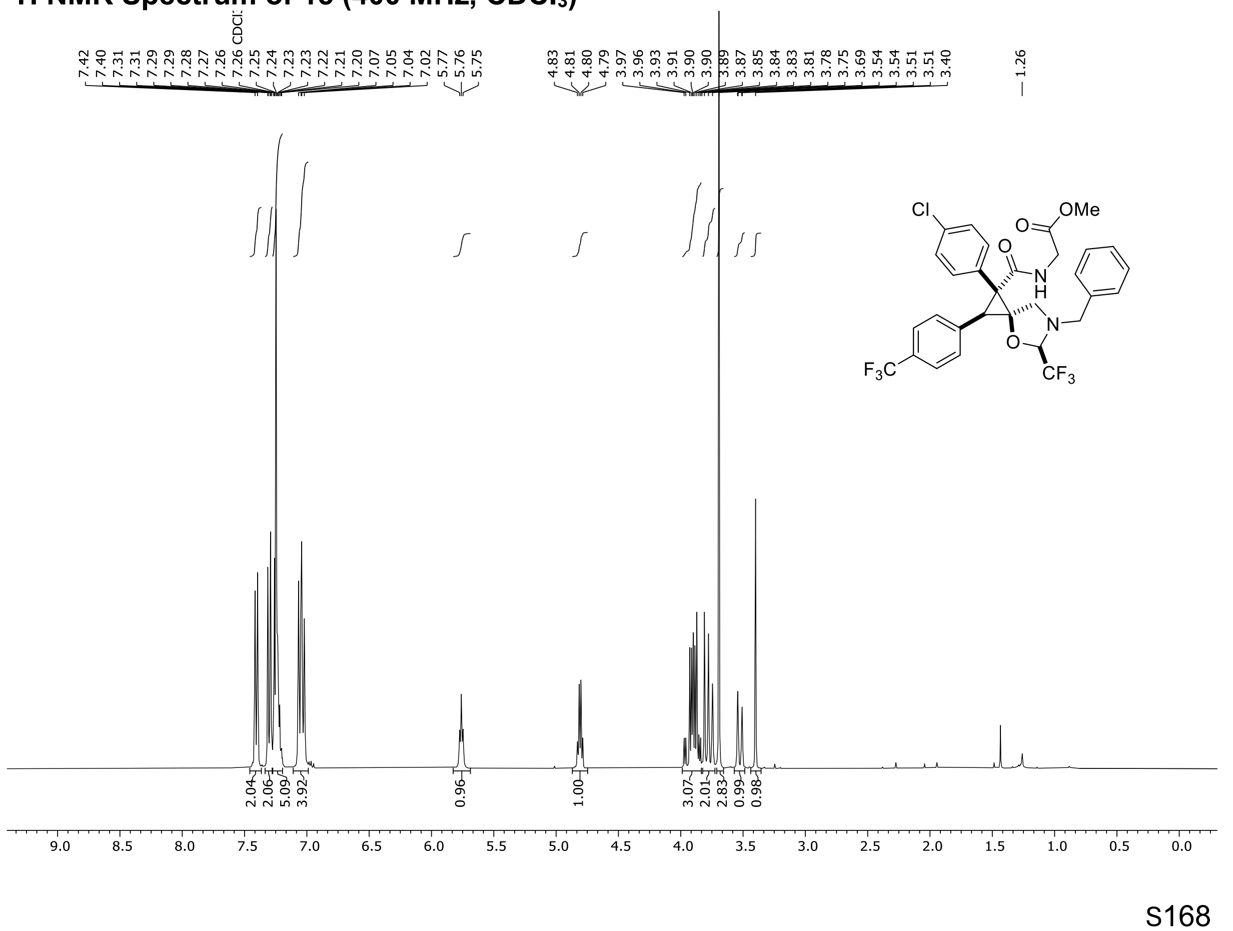

^{13}C NMR Spectrum of 15 (101 MHz, $CDCl_3$)

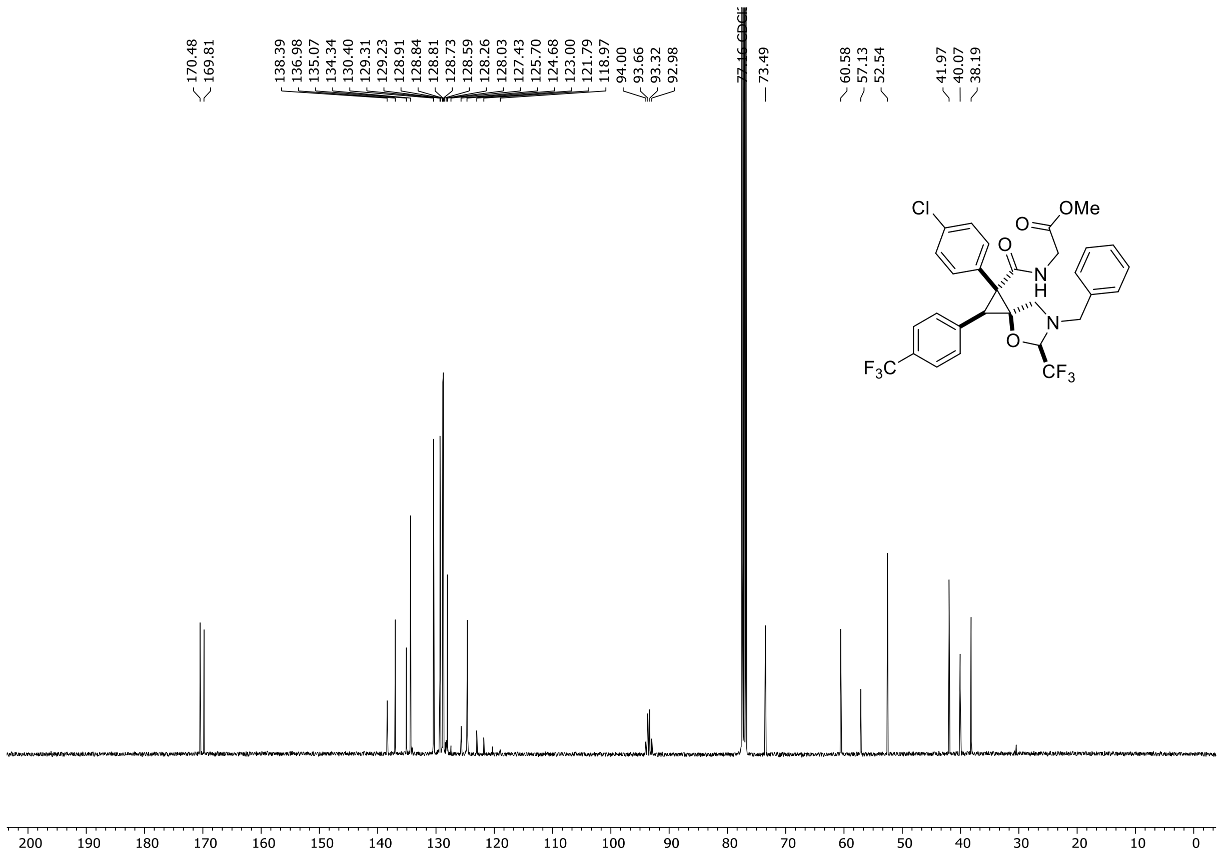

^{19}F NMR Spectrum of 15 (376 MHz, $CDCl_3$)

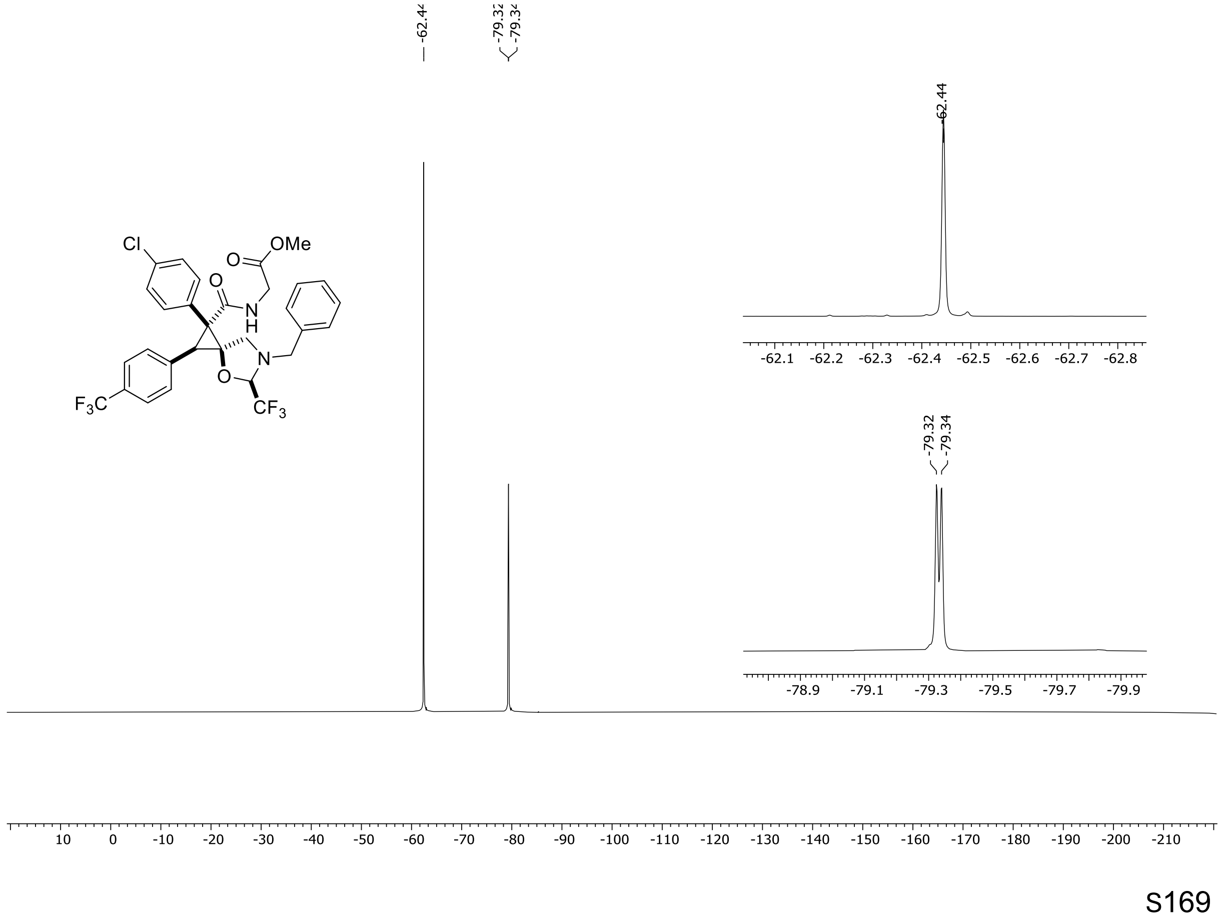

^{1}H NMR Spectrum of 16a (400 MHz, $CDCl_3$)

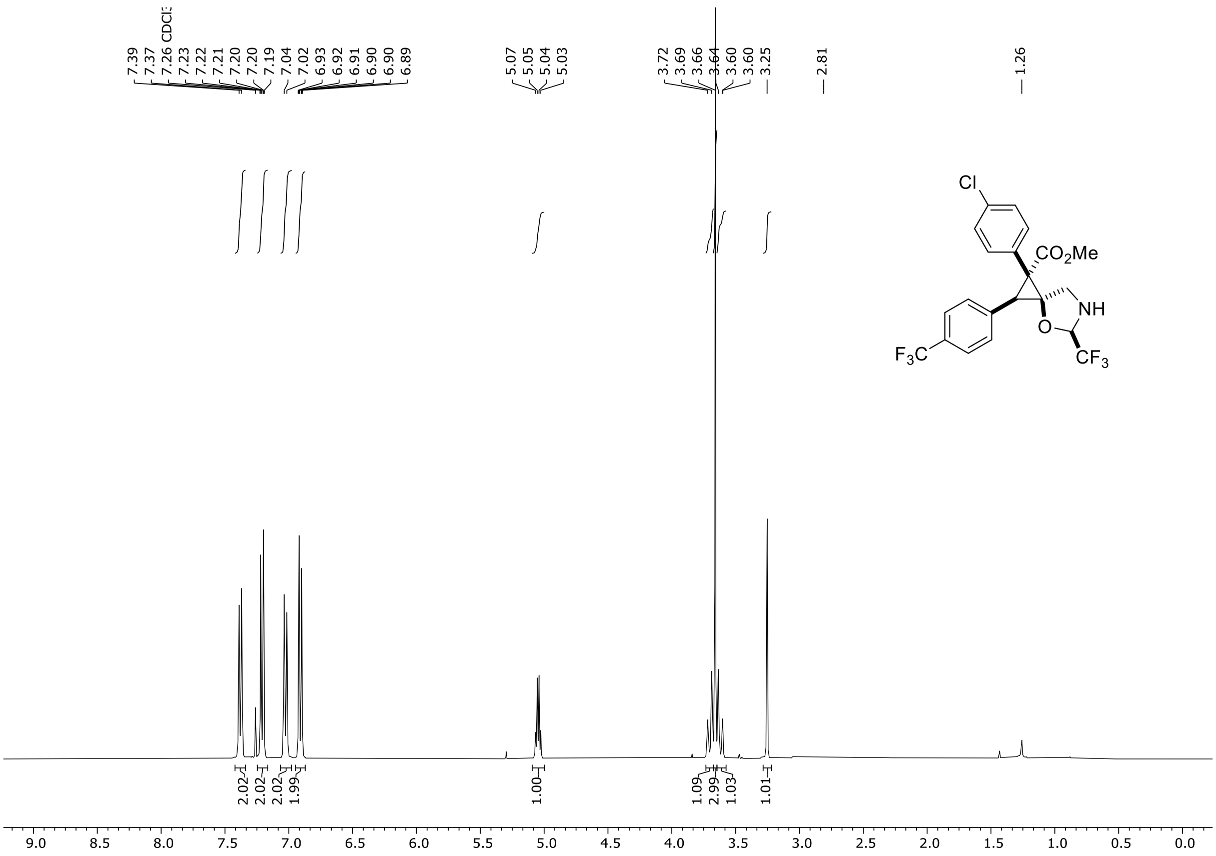

^{13}C NMR Spectrum of 16a (101 MHz, $CDCl_3$)

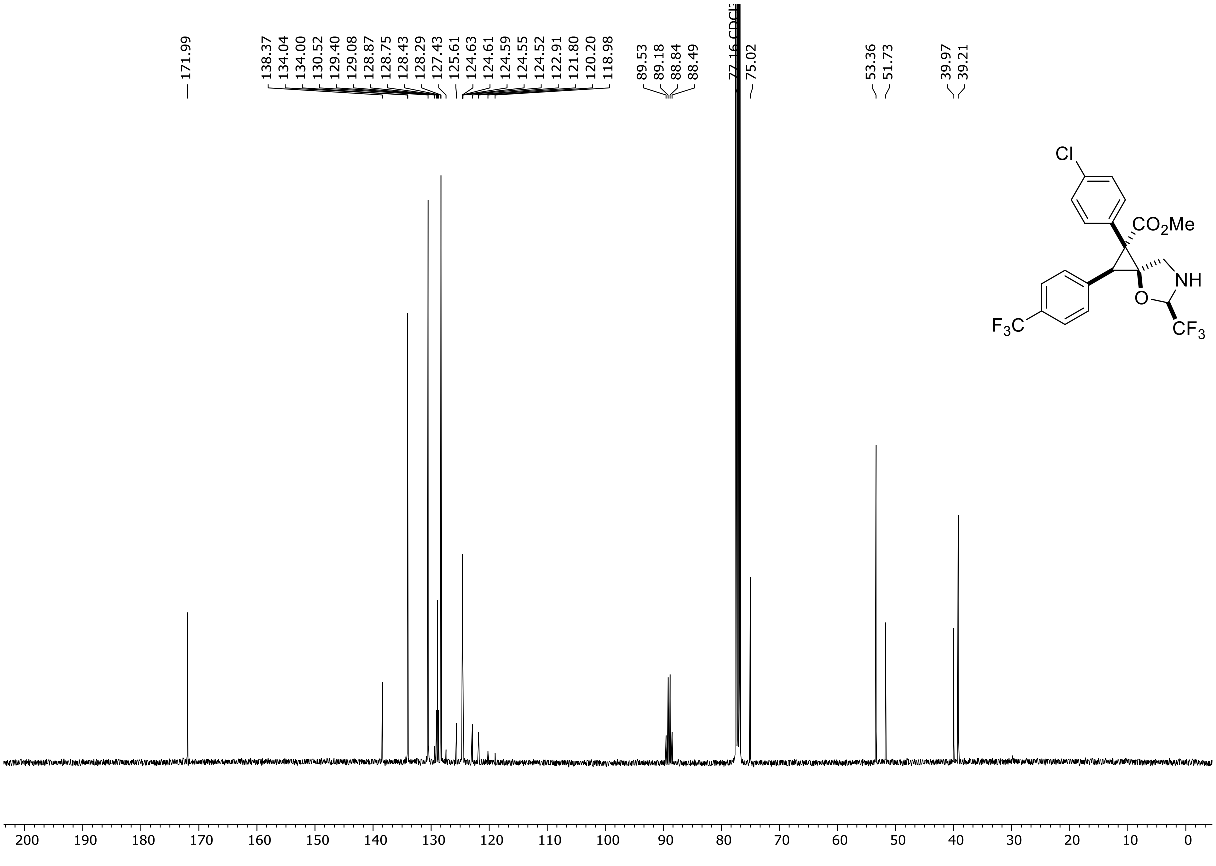

^{19}F NMR Spectrum of 16a (376 MHz, $CDCl_3$)

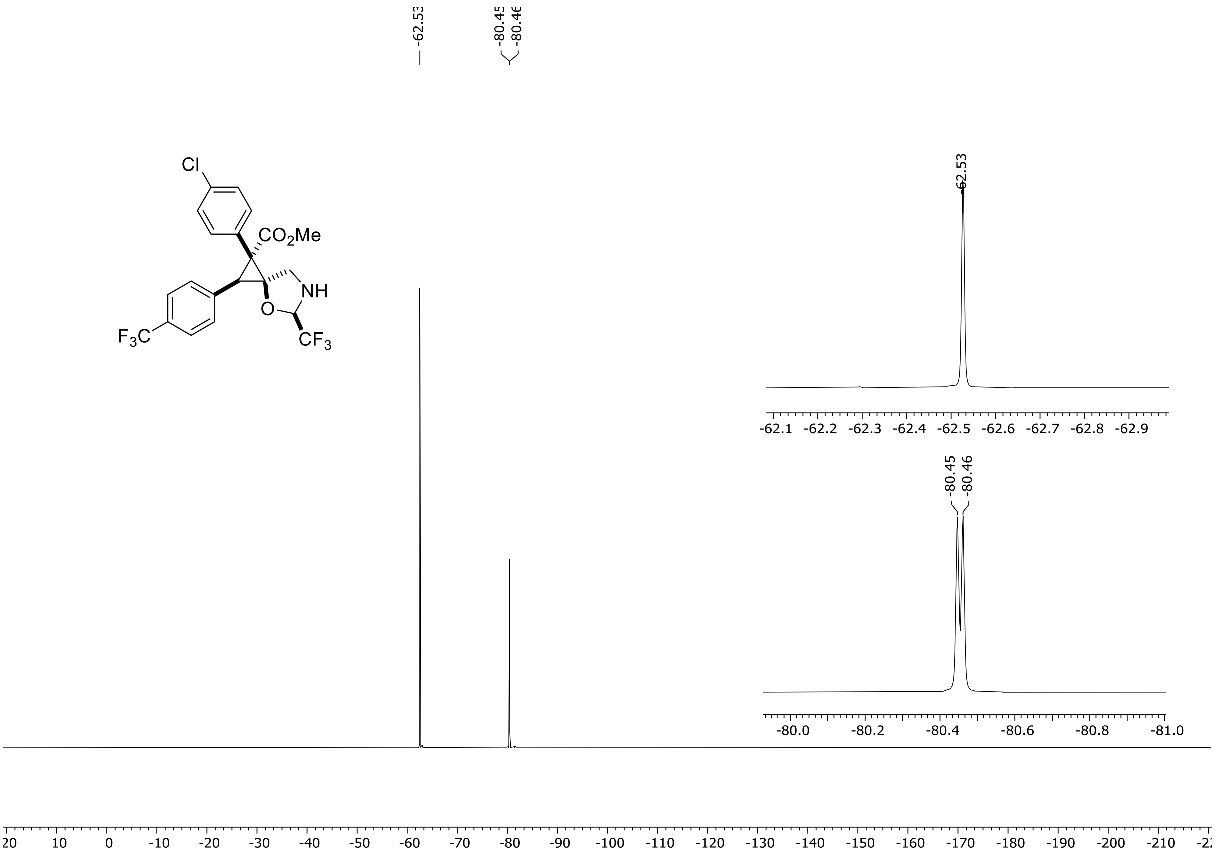

^{1}H NMR Spectrum of 16b (400 MHz, $CDCl_3$)

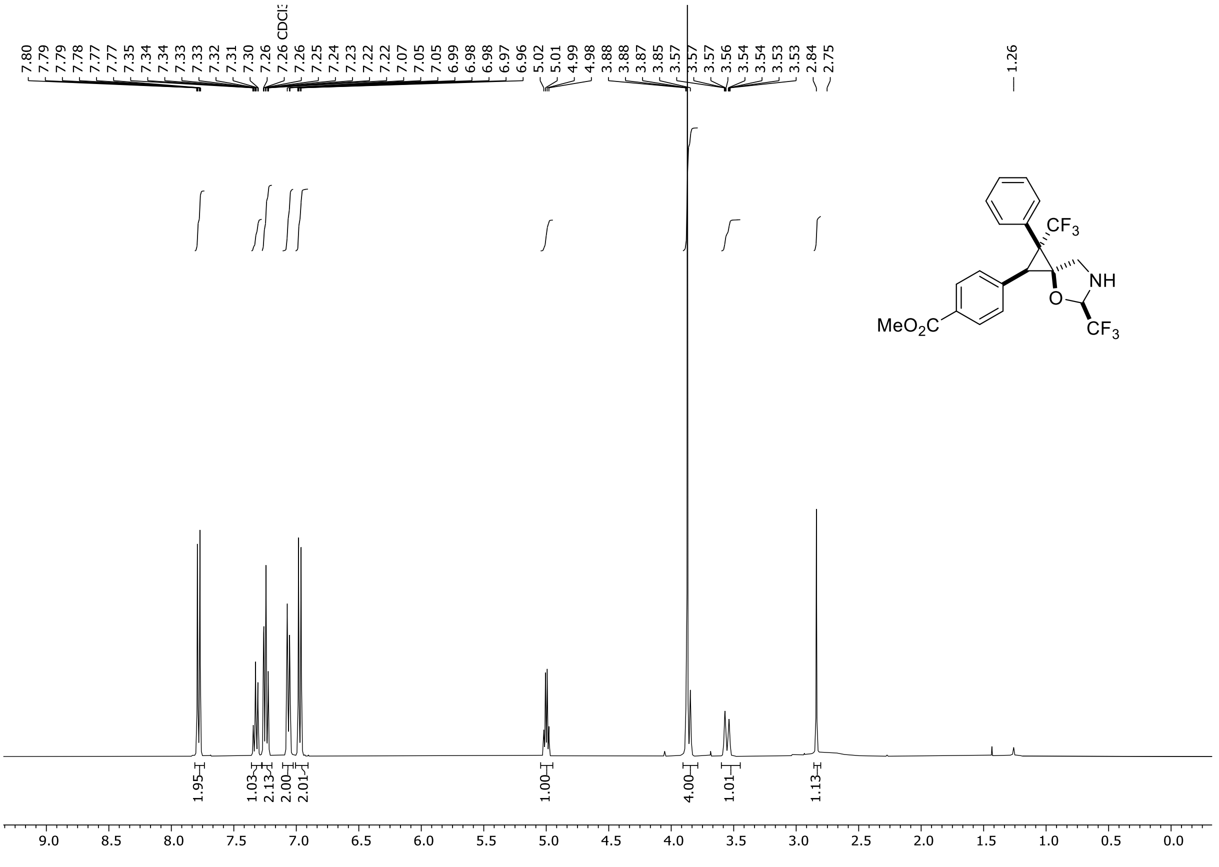

^{13}C NMR Spectrum of 16b (101 MHz, $CDCl_3$)

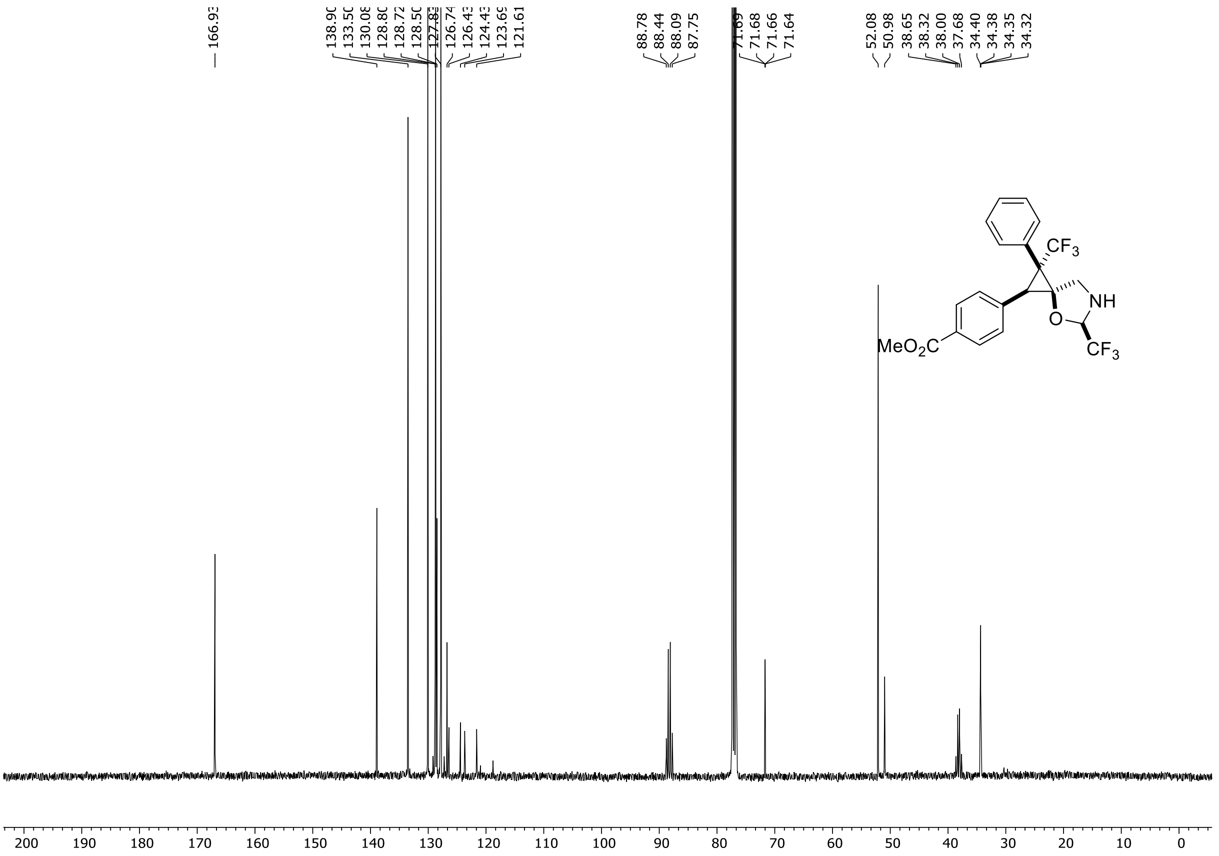

^{19}F NMR Spectrum of 16b (376 MHz, $CDCl_3$)

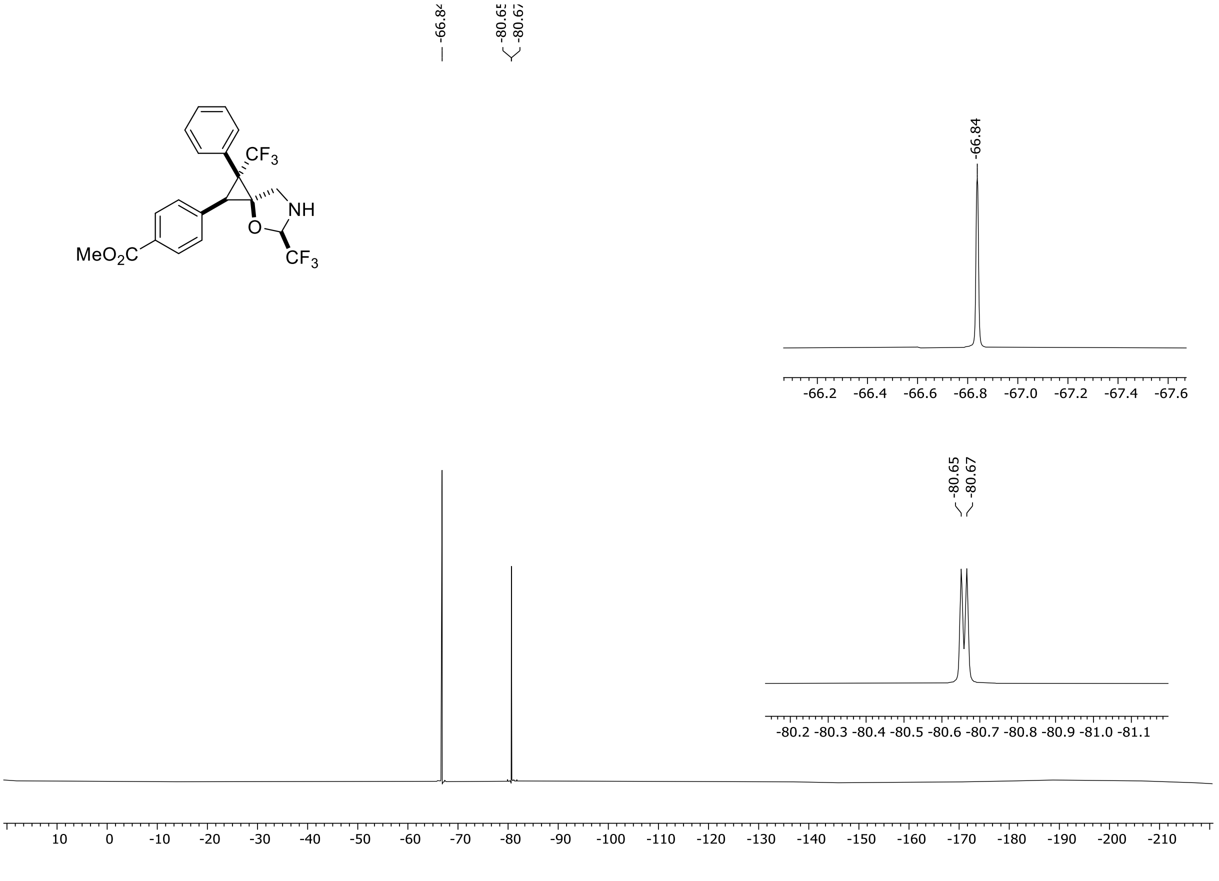

HPLC Traces

Methyl 2-((benzylamino)methyl)-1-phenylcycloprop-2-ene-1-carboxylate (*rac*)-3a

Chiral HPLC Daicel Chiralpak IB N-5 column: 80:20 hexane/IPA, flow rate 1 mL/min, λ= 254 nm

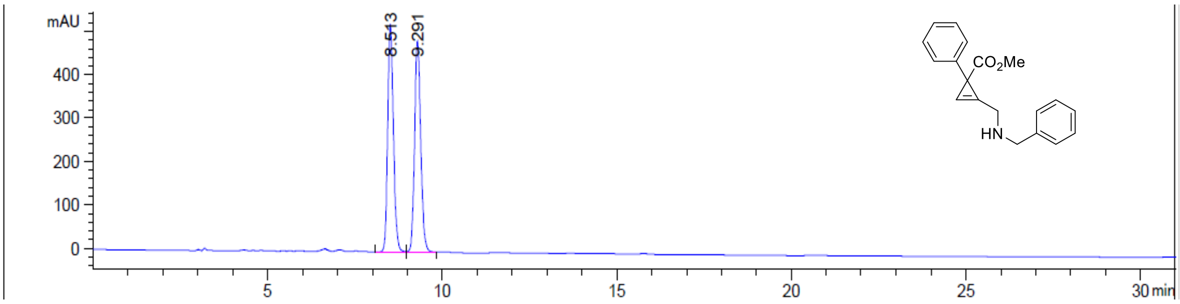

Signal 3: DAD1 C, Sig=214,4 Ref=360,100

Peak #	RetTime [min]	Type	Width [min]	Area [mAU*s]	Height [mAU]	Area %
1	8.513	BB	0.1742	5961.85791	526.12244	50.0003
2	9.291	BB	0.1893	5961.78467	485.47464	49.9997

Methyl 2-((benzylamino)methyl)-1-phenylcycloprop-2-ene-1-carboxylate (+)-3a

Chiral HPLC Daicel Chiralpak IB N-5 column: 80:20 hexane/IPA, flow rate 1 mL/min, λ= 254 nm

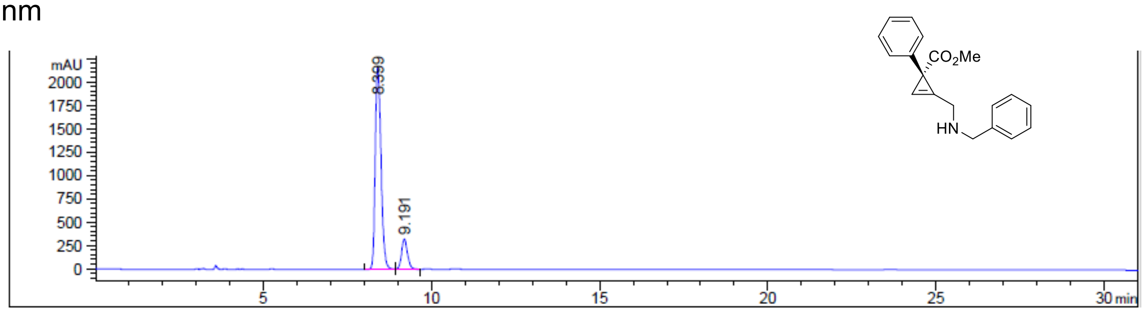

Signal 3: DAD1 C, Sig=214,4 Ref=360,100

Peak #	RetTime [min]	Type	Width [min]	Area [mAU*s]	Height [mAU]	Area %
1	8.399	BV	0.1884	2.60819e4	2167.81714	86.8593
2	9.191	VB	0.1867	3945.87305	322.54572	13.1407

Methyl 6-benzyl-1-phenyl-5-(trifluoromethyl)-2-(4-(trifluoromethyl)phenyl)-4-oxa-6-azaspiro[2.4]heptane-1-carboxylate (*rac*)-3a

Chiral HPLC Daicel Chiralpak IB N-5 column: 99:1 hexane/IPA, flow rate 1 mL/min, λ= 254 nm

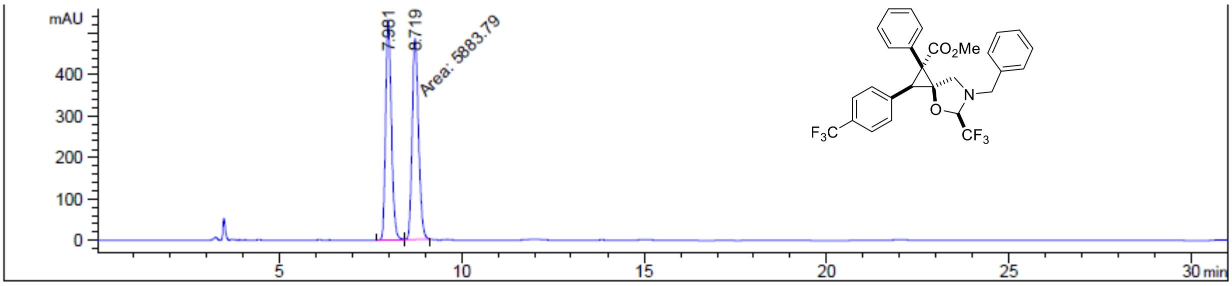

Signal 3: DAD1 C, Sig=214,4 Ref=360,100

Peak #	RetTime [min]	Type	Width [min]	Area [mAU*s]	Height [mAU]	Area %
1	7.981	BV	0.1711	5879.17920	531.14740	49.9804
2	8.719	MM	0.2026	5883.79443	483.96506	50.0196

Methyl 6-benzyl-1-phenyl-5-(trifluoromethyl)-2-(4-(trifluoromethyl)phenyl)-4-oxa-6-azaspiro[2.4]heptane-1-carboxylate (+)-**6a**

Chiral HPLC Daicel Chiralpak IB N-5 column: 99:1 hexane/IPA, flow rate 1 mL/min, λ= 254 nm

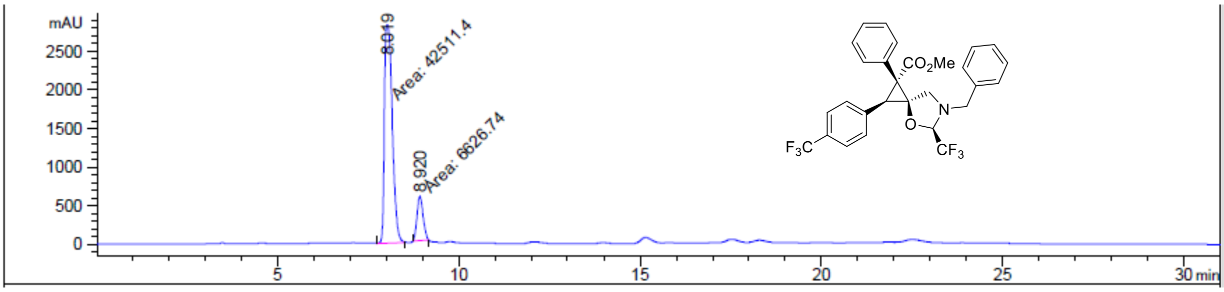

Signal 3: DAD1 C, Sig=214,4 Ref=360,100

Peak #	RetTime [min]	Type	Width [min]	Area [mAU*s]	Height [mAU]	Area %
1	8.019	MM	0.2505	4.25114e4	2828.15918	86.5140
2	8.920	MM	0.1941	6626.74463	568.93341	13.4860
